# Predicting Secretory Proteins of Malaria Parasite by Incorporating Sequence Evolution Information into Pseudo Amino Acid Composition via Grey System Model

**DOI:** 10.1371/journal.pone.0049040

**Published:** 2012-11-26

**Authors:** Wei-Zhong Lin, Jian-An Fang, Xuan Xiao, Kuo-Chen Chou

**Affiliations:** 1 Information Science and technology School, Donghua University, Shanghai, China; 2 Computer Department, Jing-De-Zhen Ceramic Institute, Jing-De-Zhen, China; 3 Gordon Life Science Institute, San Diego, California, United States of America; Instituto de Ciências Biomédicas/Universidade de São Paulo - USP, Brazil

## Abstract

The malaria disease has become a cause of poverty and a major hindrance to economic development. The culprit of the disease is the parasite, which secretes an array of proteins within the host erythrocyte to facilitate its own survival. Accordingly, the secretory proteins of malaria parasite have become a logical target for drug design against malaria. Unfortunately, with the increasing resistance to the drugs thus developed, the situation has become more complicated. To cope with the drug resistance problem, one strategy is to timely identify the secreted proteins by malaria parasite, which can serve as potential drug targets. However, it is both expensive and time-consuming to identify the secretory proteins of malaria parasite by experiments alone. To expedite the process for developing effective drugs against malaria, a computational predictor called “**iSMP-Grey**” was developed that can be used to identify the secretory proteins of malaria parasite based on the protein sequence information alone. During the prediction process a protein sample was formulated with a 60D (dimensional) feature vector formed by incorporating the sequence evolution information into the general form of PseAAC (pseudo amino acid composition) via a grey system model, which is particularly useful for solving complicated problems that are lack of sufficient information or need to process uncertain information. It was observed by the jackknife test that **iSMP-Grey** achieved an overall success rate of 94.8%, remarkably higher than those by the existing predictors in this area. As a user-friendly web-server, **iSMP-Grey** is freely accessible to the public at http://www.jci-bioinfo.cn/iSMP-Grey. Moreover, for the convenience of most experimental scientists, a step-by-step guide is provided on how to use the web-server to get the desired results without the need to follow the complicated mathematical equations involved in this paper.

## Introduction

Malaria is a potentially fatal tropical disease caused by a parasite known as Plasmodium. Four distinct species of plasmodium that can produce the disease in different forms: *Plasmodium falciparum*, *Plasmodium vivax*, *Plasmodium ovale*, and *Plasmodium malaria*. Of these four, *Plasmodium falciparum*, or *P. falciparum*, is the most widespread and dangerous. If not timely treated, it may lead to the fatal cerebral malaria, which remains one of the most devastating global health crises. Nearly half of the world's population is still at risk from its infection. According to the World Health Organization's 2010 World Malaria Report (http://www.who.int/malaria/world_malaria_report_2010/worldmalariareport2010.pdf), there are more than 225 million cases of malaria each year, killing around 781,000 people, corresponding to 2.23% of deaths worldwide. Malaria is more dangerous for women and children. It was stated in the World Health Organization's 2011 World Malaria Report (http://www.who.int/malaria/world_malaria_report_2011/9789241564403_eng.pdf) that 81% of cases and 91% of deaths occurred in the African Region, mostly involving children under five and women with pregnancy. Malaria was usually associated with poverty; actually it was a cause of poverty and a major hindrance for economic development. The situation has become even worse over the last few years with the increase in resistance to the drugs normally used to combat the parasites that cause the disease. Therefore, one strategy to deal with the growing malaria problem is to identify and characterize new and durable antimalarial drug targets, the majority of which are parasite proteins [1]. Parasite secretes an array of proteins within the host erythrocyte to facilitate its own survival within the host cell. These proteins can serve as potential drug or vaccine targets. However, it is difficult to experimentally identify the secretory proteins of *P. falciparum* owing to the complex nature of parasite. With the completion of *Plasmodium* genome sequence, it is both challenging and urgent to develop an automatic method or high throughput tool for identifying secretory proteins of *P. falciparum*.

Actually, some efforts have been made in this regard. In a pioneer study, Verma et al. [2] proposed a method for identifying proteins secreted by malaria parasite. In their prediction method, the operation engine was the Support Vector Machine (SVM) while the protein samples were formulated with the amino acid composition, dipeptide composition, and position specific scoring matrix (PSSM) [3]. Subsequently, Zuo and Li [4] introduced the K-minimum increment of diversity (K-MID) approach to predict secretory proteins of malaria parasite based on grouping of amino acids. Meanwhile, various studies around this topic were also carried out [5], [6], [7], [8], [9].

In the past, various predictors for protein systems were developed by incorporating the evolutionary information via PSSM [10], [11], [12], [13], [14], [15], [16], [17], [18], [19], [20]. In the above papers, however, only the statistical information of PSSM [3] was utilized but the inner interactions among the constituent amino acid residues in a protein sample, or its sequence-order effects, were ignored.

To avoid completely lose the sequence-order information associated with PSSM, the concept of pseudo amino acid composition (PseAAC) [21], [22] was utilized to incorporate the evolutionary information into the formulation of a protein sample, as done in predicting protein subcellular localization [23], [24], 25, predicting protein fold pattern [26], identifying membrane proteins and their types [27], predicting enzyme functional classes and subclasses [28], identifying protein quaternary structural attribute [29], predicting antibacterial peptides [30], predicting allergenic proteins [31], and identifying proteases and their types [32].

The present study was initiated in an attempt to develop a new and more powerful predictor for identifying the secretory proteins of malaria parasite by incorporating the sequence evolution information into PseAAC via a grey system model [33].

According to a recent review [34], to establish a really useful statistical predictor for a protein system, we need to consider the following procedures: (i) construct or select a valid benchmark dataset to train and test the predictor; (ii) formulate the protein samples with an effective mathematical expression that can truly reflect their intrinsic correlation with the target to be predicted; (iii) introduce or develop a powerful algorithm (or engine) to operate the prediction; (iv) properly perform cross-validation tests to objectively evaluate the anticipated accuracy of the predictor; (v) establish a user-friendly web-server for the predictor that is accessible to the public. Below, let us describe how to deal with these steps.

## Materials and Methods

### 1. Benchmark Dataset

The benchmark dataset 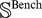 used in this study was taken from Verma et al. [2]. The dataset can be formulated as
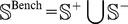
(1)where 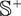 contains 252 secretory proteins of malaria parasite, 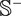 contains 252 non-secretory proteins of malaria parasite, and the symbol 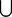 represents the union in the set theory. The same benchmark dataset was also used by Zuo and Li [4]. For reader's convenience, the sequences of the 252 secretory proteins in 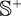 and those in 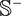 are given in Supporting Information S1.

### 2. A Novel PseAAC Feature Vector by Incorporating Sequence Evolution Information via the Grey System Theory

To develop a powerful predictor for a protein system, one of the keys is to formulate the protein samples with an effective mathematical expression that can truly reflect their intrinsic correlation with the target to be predicted [34]. To realize this, the pseudo amino acid composition (PseAAC) was proposed [21] to replace the simple amino acid composition (AAC) for representing the sample of a protein. Ever since the concept of PseAAC was introduced in 2001 [21], it has penetrated into almost all the fields of protein attribute predictions, such as predicting protein submitochondrial localization [35], predicting protein structural class [36], predicting DNA-binding proteins [37], identifying bacterial virulent proteins [38], predicting metalloproteinase family [39], predicting protein folding rate [40], predicting GABA(A) receptor proteins [41], predicting protein supersecondary structure [42], identifying protein quaternary structural attribute [43], predicting cyclin proteins [44], classifying amino acids [45], predicting enzyme family class [46], identifying risk type of human papillomaviruses [47], and discriminating outer membrane proteins [48], among many others (see a long list of references cited in [49]). Because it has been widely used, recently a powerful software called PseAAC-Builder [49] was proposed for generating various special modes of PseAAC, in addition to the web-server PseAAC [50] established in 2008.

According to a recent review [34], the general form of PseAAC for a protein 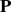 can be formulated as
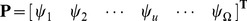
(2)where 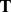 is a transpose operator, while the subscript 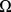 is an integer and its value as well as the components 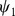, 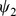, … will depend on how to extract the desired information from the amino acid sequence of 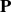.

The form of **Eq.2** can cover almost all the various modes of PseAAC. Particularly, it can be used to reflect much more essential core features deeply hidden in complicated protein sequences, such as those for the functional domain (FunD) information [51], [52], [53] (cf. Eqs.9–10 of [34]), gene ontology (GO) information [54], [55] (cf. Eqs.11–12 of [34]), and sequence evolution information [3] (cf. Eqs.13–14 of [34]).

In this study, we are to use a novel approach to define the 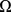 elements in **Eq.2**. As is well known, biology is a natural science with historic dimension. All biological species have developed starting out from a very limited number of ancestral species. It is true for protein sequence as well [56]. Their evolution involves changes of single residues, insertions and deletions of several residues [57], gene doubling, and gene fusion. With these changes accumulated for a long period of time, many similarities between initial and resultant amino acid sequences are gradually eliminated, but the corresponding proteins may still share many common attributes, such as having basically the same biological function and residing at a same subcellular location. To incorporate this kind of sequence evolution information into the PseAAC of **Eq.2**, let us use the information of the PSSM (Position-Specific Scoring Matrix) [3], as described below.

According to [3], the sequence evolution information of protein 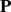 with 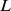 amino acid residues can be expressed by a 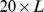 matrix, as given by
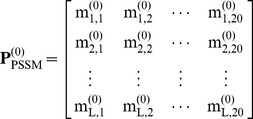
(3)where 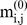 represents the original score of amino acid residue in the *i*-th 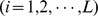 sequential position of the protein that is being changed to amino acid type *j* 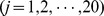 during the evolution process. Here, the numerical codes 1, 2, …, 20 are used to denote the 20 native amino acid types according to the alphabetical order of their single character codes [58]. The 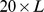 scores in **Eq.3** were generated by using PSI-BLAST [3] to search the UniProtKB/Swiss-Prot database (Release 2010_04 of 23-Mar-2010) through three iterations with 0.001 as the 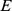-value cutoff for multiple sequence alignment against the sequence of the protein 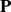. In order to make every element in **Eq.3** within the range of 0–1, a conversion was performed through the standard sigmoid function to make it become
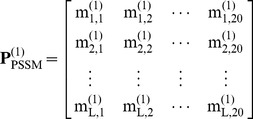
(4)where
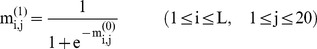
(5)


Now, let us describe how to extract the useful information from **Eq.4** via a grey system model. According to the grey system theory [33], if the information of a system investigated is fully known, it is called a “white system”; if completely unknown, a “black system”; if partially known, a “grey system”. The model developed based on such a theory is called “grey model”, which is a kind of nonlinear and dynamic model formulated by a differential equation. The grey model is particularly useful for solving complicated problems that are lack of sufficient information, or need to process uncertain information and reduce random effects of acquired data. In the grey system theory, an important and generally used model is called GM(1,1) [33]. It is quite effective for monotonic series, with good simulating effect and small error, as reflected by the fact that using the GM(1,1) model has remarkably improved the success rates in predicting protein structural classes [59]. However, if the series concerned are not monotonic, the simulating effect of the GM(1,1) model would not be good and its error might be quite large. To overcome such a shortcoming, in this study we are to use a different grey system model called GM(2,1) [33], which can be effectively used to deal with the oscillation series.

To extract the serial information of **Eq.4**, let us consider the 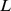 components in its 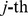 column, i.e., 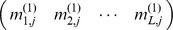, as an initial series. Obviously, the *j*-th column of the **Eq.4** is an oscillation series but not monotonic as in the case investigated in [59]. To deal with such a problem, instead of the GM(1,1), let us adopt the GM(2,1) model here. According to the GM(2,1) model [33], we have the following 2nd-order grey differential equation with one variable:
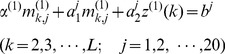
(6)where
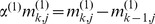
(7)and
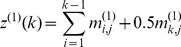
(8)In **Eq.6**, the coefficients 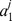 and 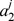 are associated with the developing coefficients, and 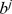 the influence coefficient. Actually, 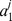, 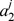, and 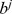 can be expressed as the components of a 3D vector as given by
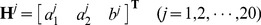
(9)in which the components 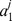, 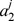, and 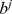 can be directly derived from the following equation
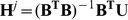
(10)where
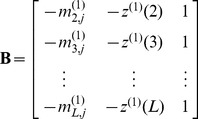
(11)and
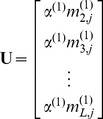
(12)Accordingly, the Ω elements in **Eq.2** are given by
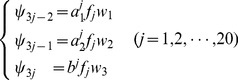
(13)where 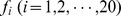 are the occurrence frequencies of the 20 different types of amino acids in the protein sample concerned, and 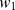, 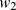, and 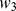 are the weight factors that will be determined by optimizing the performance of the predictor, and their concrete values will be explicitly given in the footnote of **Table 1**. Substituting **Eq.13** into **Eq.2**, we immediately obtain a feature vector with 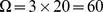 components. The 60D feature vector thus derived will be used to represent the samples of protein sequences for further study.

**Table 1:** A comparison between iSMP-Grey and K-MID by the jackknife test.

Predictor	Sn (%)	Sp (%)	Acc (%)	MCC
iSMP-Grey^a^	93.25	96.46.	94.84	0.90
K-MID^b^	81.75	99.60	90.67	0.83

a The parameters used: 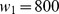, 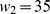, and 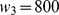 for **Eq.14**; 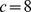 and 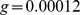 for the LIBSVM operation engine.

b From ref.[4].

### 3. The SVM Operation Engine

In this study, the Support Vector Machine (SVM) algorithm was adopted to perform the prediction. The SVM software was implemented from the LIBSVM package [60]. The software thus obtained provided a simple interface by which the users can easily perform classification prediction by properly selecting the built-in parameters 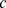 and 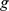. In this study we searched the optimal parameters 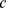 and 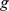 by the grid arithmetic built in the LIBSVM software, and their optimal values are also explicitly given in the footnote of **Table 1**. Meanwhile, the MATLAB windows were adopted in developing the classifier.

The predictor thus established is called **iSMP-Grey**, which can be used to identify whether a protein of malaria parasite is secretory or non-secretory according to its sequence information alone.

### 4. Web-Server and User Guide

To enhance the value of its practical applications, a web-server for **iSMP-Grey** was established. Moreover, for the convenience of the vast majority of experimental scientists, here let us provide a step-by-step guide to show how the users can easily get the desired result by means of the web-server without the need to follow the above mathematical equations for its development and integrity.

#### Step 1

**Figure 1:**
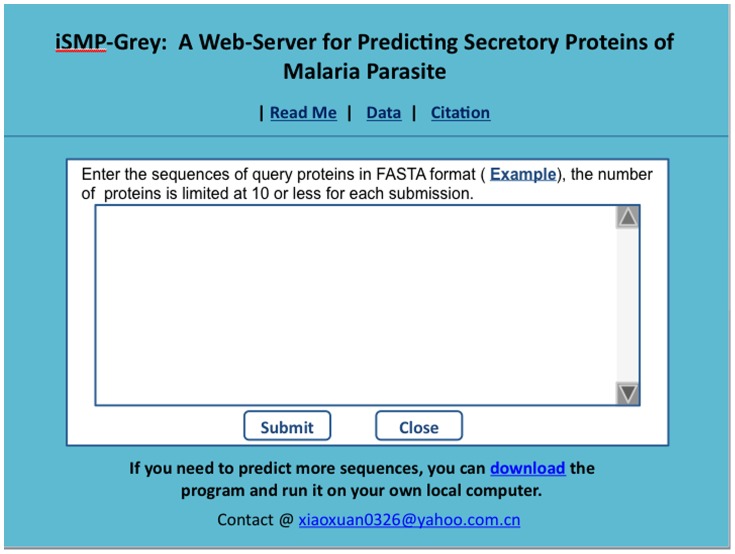
A semi-screenshot to show the top page of the iSMP-Grey web-server. Its web-site address is at http://www.jci-bioinfo.cn/iSMP-Grey.

Open the web server at the site http://www.jci-bioinfo.cn/iSMP-Grey and you will see the top page of the predictor on your computer screen, as shown in **Fig. 1**. Click on the Read Me button to see a brief introduction about **iSMP-Grey** predictor and the caveat when using it.

#### Step 2

Either type or copy and paste the query protein sequence into the input box at the center of **Fig. 1**. The input sequence should be in the FASTA format. A sequence in FASTA format consists of a single initial line beginning with a greater-than symbol (“>”) in the first column, followed by lines of sequence data. The words right after the “>” symbol in the single initial line are optional and only used for the purpose of identification and description. The sequence ends if another line starting with a “>” appears; this indicates the start of another sequence. The example sequences in FASTA format can be seen by clicking on the Example button right above the input box. The maximum number of query protein sequences allowed for each submission is 10.

#### Step 3

Click on the Submit button to see the predicted result. For example, if you use the two query peptide sequences in the Example window as the input, about 2–3 minutes after clicking the Submit button, you will see on your screen that the 1^st^ query protein is a “**Secretory Protein of Malaria Parasite**”, and that the 2^nd^ query protein 2 is “**Non-Secretory Protein of Malaria parasite**”. All these results are fully consistent with the experimental observations.

#### Step 4

Click on the Citation button to find the relevant paper that documents the detailed development and algorithm of **iSMP-Grey**.

#### Step 5

Click on the Data button to download the benchmark dataset used to train and test the **iSMP-Grey** predictor.

#### Step 6

The program is also available by clicking the button download on the lower panel of **Fig. 1**.

### 5. Performance Evaluation

In statistical prediction, the following three cross-validation methods are often used to examine a predictor for its effectiveness in practical application: independent dataset test, subsampling (K-fold cross-validation) test, and jackknife test. However, as elaborated by a recent review [34] and demonstrated by Eqs.28–32 therein, among the three cross-validation methods, the jackknife test is deemed the least arbitrary and most objective because it can always yield a unique result for a given benchmark dataset, and hence has been widely recognized and increasingly used by investigators for examining the accuracy of various predictors (see, e.g., [36], [38], [39], [41], [44], [47], [61], [62], [63], [64], [65], [66]). Accordingly, the jackknife test was also adopted in this study to examine the anticipated success rates of the current predictor.

Also, to use a more intuitive and easier-to-understand method to measure the prediction quality, the rates of correct predictions for the secretory proteins of malaria parasite in dataset 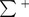 and the non-secretory proteins of malaria parasite in dataset 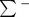 are respectively defined by [67]

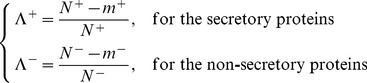
(14)where 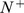 is the total number of the secreted proteins investigated and 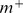 the number of the secreted proteins missed in the predicted result; 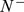 the total number of the non-secreted proteins investigated and 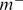 the number of the non-secreted proteins missed in the predicted result. The overall success prediction rate is given by [68]

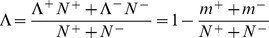
(15)


It is clear from **Eqs.14** **–** **15** that, if and only if none of the secreted proteins and non-secreted proteins are mispredicted, i.e., 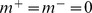 and 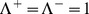, we have the overall success rate 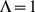. Otherwise, the overall success rate would be smaller than 1. It is instructive to point out that the following equation is often used in literatures for examining the performance quality of a predictor
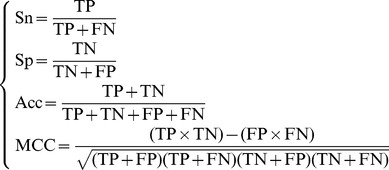
(16)where TP represents the true positive; TN, the true negative; FP, the false positive; FN, the false negative; Sn, the sensitivity; Sp, the specificity; Acc, the accuracy; MCC, the Mathew's correlation coefficient.

The relations between the symbols in **Eq.15** and those in **Eq.16** are given by
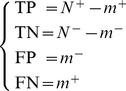
(17)It follows by substituting **Eq.17** into **Eq.16** and noting **Eq.15**

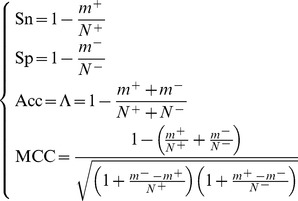
(18)


As can be obviously seen from the above equation, when 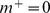 meaning none of the secreted proteins was missed in prediction, we have the sensitivity 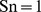; while 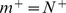 meaning all the secreted proteins were missed in prediction, we have the sensitivity 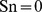. Likewise, when 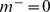 meaning none of the non-secreted proteins was incorrectly predicted as secreted protein, we have the specificity 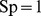; while 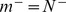 meaning all the non-secreted proteins were incorrectly predicted as secreted proteins, we have the specificity 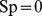. When 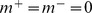 meaning that none of the secreted proteins in the dataset 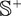 and non of non-secreted proteins in 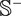 was incorrectly predicted, we have the overall accuracy 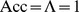; while 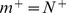
and 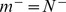 meaning that all the secreted proteins in the dataset 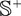 and all the non-secreted proteins in 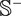 were incorrectly predicted, we have the overall accuracy 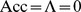. The MCC correlation coefficient is usually used for measuring the quality of binary (two-class) classifications. When 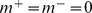 meaning that none of the secreted proteins in the dataset 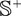 and none of the non-secreted proteins in 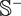 was incorrectly predicted, we have 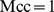; when 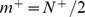 and 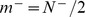 we have 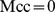 meaning no better than random prediction; when 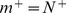 and 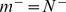 we have 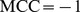 meaning total disagreement between prediction and observation. As we can see from the above discussion, it is much more intuitive and easier-to-understand when using **Eq.18** to examine a predictor for its sensitivity, specificity, overall accuracy, and Mathew's correlation coefficient.

## Results and Discussion

The results obtained with **iSMP-Grey** on the benchmark dataset 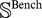 of **Eq.1** by the jackknife test are given in **Table 1**, where for facilitating comparison the results obtained by the **K-MID** predictor [4] on the same benchmark dataset with the same test method are also given. As we can see from **Table 1**, the overall success rate by **iSMP-Grey** was 94.84% with 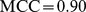, which are remarkably higher than those by the K-MID predictor [4].

Moreover, a comparison was also made with the **PSEApred** predictor [2]. Although the results by **PSEApred** as reported by Verma et al. [2] were also based on the same benchmark dataset 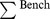 of **Eq.1**, the test method used by these authors for **PSEApred** was 5-fold cross-validation. As elaborated in [34], this would make the test without a unique result as demonstrated below. For the current case, 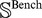 consists of 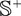 and 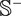, where 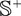 contains 252 secretory proteins of malaria parasite, and 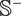 contains 252 non-secretory proteins of malaria parasite. Substituting these data into Eqs.28–29 of [34] with 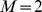 (number of groups for classification) and 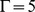 (number of folds for cross-validation), we obtain
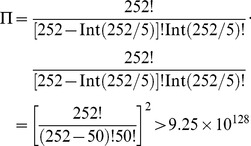
(19)where the symbol Int is the integer-truncating operator meaning to take the integer part for the number in the bracket right after it. The result of **Eq.19** indicates that the number of possible combinations of taking one-fifth proteins from each of the two subsets, 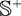 and 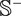, for conducting the 5-fold cross-validation will be greater than 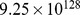, which is an astronomical figure, too large to be practically feasible. Actually, in their study [2], Verma et al. only randomly picked 100 different combinations from the possible 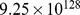 combinations (cf. **Eq.19**) to perform the 5-fold cross-validation, yielding 100 different results located within a certain region. Therefore, in their report, rather than a single figure but a figures region was used to show their test result. For example, according to their report (**Table 2**), 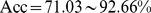, meaning that the lowest one of the 100 overall success rates obtained by the **PSEApred** predictor [2] was 71.03%, while the highest one was 92.66%. To make the comparison of **iSMP-Grey** with **PSEApred** [2] under the same condition with the same test method, we also randomly picked 100 different combinations as done by Verma et al. [2] to perform the 5-fold cross-validation test with **iSMP-Grey**, and the corresponding results thus obtained are given in **Table 2** as well. As we can see from the table, not only the average rates obtained by the **iSMP-Grey** predictor are remarkably higher than those by the **PSEApred** predictor [2], but the corresponding region widths by the former are also significantly narrower than those by the latter, indicating the success rates by the **iSMP-Grey** are not only higher but also more stable than those by the **PSEApred** predictor [2].

**Table 2:** A comparison between iSMP-Grey and PSEApred by 5-fold cross-validation test.

Predictor	Sn (%)^c^	Sp (%)^c^	Acc (%)^c^	MCC^c^
iSMP-Grey^a^	90.48∼92.46	94.05∼98.02	92.86∼94.84	0.87∼0.90
PSEApred^b^	73.41∼97.22	44.84∼100	71.03∼92.66	0.49∼0.86

a See footnote a of **Table 1**.

b From ref. [2].

c See the discussion in the text and **Eq.19** for why the results obtained by the 5-fold cross-validation test were not unique.

All the above results have indicated that the novel pseudo amino acid composition formulated via the grey system model GM(2,1) can more effectively incorporate the protein sequence evolution information so as to remarkably enhance the success rates of the **iSMP-Grey** predictor in identifying the secretory proteins of malaria parasite. It is anticipated that **iSMP-Grey** may become a useful high throughput tool for both basic research and drug development in the relevant areas.

## Supporting Information

Supporting Information S1The benchmark dataset 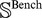 includes 504 proteins, classified into 252 secretory proteins of malaria parasite and 252 non-secretory proteins.(PDF)
