# Supplementary material for: Predicting Secretory Proteins of Malaria Parasite by Incorporating Sequence Evolution Information into Pseudo Amino Acid Composition via Grey System Model
Source: PLoS One. 2012 Nov 26;7(11):e49040. doi: 10.1371/journal.pone.0049040 (PMC3506597; doi:10.1371/journal.pone.0049040)
Supplement: Supporting Information S1 — The benchmark dataset includes 504 proteins, classified into 252 secretory proteins of malaria parasite and 252 non-secretory proteins. (PDF) [file pone.0049040.s001.pdf]

Online Supporting Information S1. The benchmark dataset $\mathbb{S}^{\text{Bench}}$ includes 504 proteins, classified into 252 secretory proteins of malaria parasite and 252 non-secretory proteins. See the text of the paper for further explanation.

---

(1) $\mathbb{S}^{+}$: 252 secretory proteins of malaria parasite

```
>PFE0065w, 337 bases, D1F677AA checksum.
MCSAARAFDFFTDLADEPTQLQDAVPETTEKLAEVVSDAATNVTDAVSDTASGIGSLV
GEAASSLGNLVGEAASGIGNIVGGAASGIGNIVGGAASGIGSLVGDAASGLGNLVGDA
AEALATTELKDVIPENTESTTDLVPSEVSPPVDDYLDDDGFSSFREFLESTPCWQRRM
AQEALLNEYEVESPAESMSPILRVQFFADFAKQAVHVAKQNYLYVVIFLFFVINILLF
INFYNLGKRKGYYLAKKQKKEQMLEQNPEQNPEQNAQQNAQQNAQQNAQQNAQQNAQQ
NAQQNTQQNTQQKTQQNPQQNAQQNTQQNTQQQSTTKSTTKTVARET
>PF14_0142|serine/threonine, 304 bases, DD282E30 checksum.
MALEIDIDNVISKLIEVRGTRPGKNVNLTENEIKILCLSSREIFLNQPILLELEAPIK
ICGDIHGQFYDLLRLFEYGGFPPDANYLFLGDYVDRGKQSLETICLLLAYKIKYPENF
FLLRGNHECASINRIYGFYDECKRRYSVKLWKTFIDCFNCLPVAAIIDEKIFCMHGGL
SPELNNMEQIRKITRPTDVPDNGLLCDLLWSDPEKEINGWGENDRGVSFTFGQDVVHN
FLRKHELDLICRAHQVVEDGYEFFAKRQLVTLFSAPNYCGEFDNAGAMMSVDETLMCS
FQILKPVEKKKAAN
>PF11_0014, 218 bases, B689E139 checksum.
MFKKNNNKIKNGVENGTYKSSYHNGGIQFRMLAQNNTNKKSNGNTLTNILLKDKGEKG
GKKKNPDDQISDLVNLVDNMNITQEKKNEIKNLTLKYMNSDDIKEKNKSINELKKYSN
NEECKEHMDNYLMYLRMQNDIKCLKRKNFWNNIWINSINFFVIIIMIACVVVGLIEAS
SISYPLCIFPVFMLYMLVRFFPDMKMGFKKIKETCTNFFQKKKK
>PFI1820w, 1315 bases, BE84B9BB checksum.
MAPKNGSRNGKLLSLRDVLENIGSGIKDKRKNQSKYTDKLKGILTKAKFVDGLSSRYG
YVRDSDGISCNLSHKFHTNITIEAARDPCYGREQNRFDENVESYCNNDKIRGSGKIFD
GRVCVPPRRQHICDHNLEYLNNNNTDDTDDLLGNVLVTAKYEGQSIVNNHPHKETSDV
CTALARSFADIGDIVRGIDMFKPNDQDEVWNGLRSVFKKIHDNLSSEVKNAYPDDGSG
NYFKLREDWWTANRDQVWKAMTCVAPENAYFRKTEADGIGISSLILPYSKCGRDTDPP
VVDYIPQRLRWMSEWSEYFCNVLNKEIDEMNNQCKDCEMSRRCNDDSEGGKCKKCKEQ
CQIFKELVSKWKNQFDKQSMKYMELYNKASTNITKQNSSAPERGYRRNHRRRGYDDDT
NVQLFLKKVIENNECKVESLGKYLDKTSHCGNYNFNYDNTPGSNRSNAFEITPEKFKK
ACKCKIPNPLEKCPNEENKNVCTRFDKVYSCTSLSFKNDLSEWNNSGVKNKENDNNGV
LVPPRRRNLCINLFSKKDYKMKDENDFKEDLLNAAFSQGKLLGKKYSNYSNEAYEAMK
FSYADYSDIVKGTDMMNDLKKLNKELNTLLKETEKGDISVDRKTWWDDNKNVVWNAML
CGYKTENENQQLNSSWCNVPDDDYIDQFLRWLTEWAQQYCKEKLIKAHIINTKCKDIV
EGRKHKSMVDITDVECKRLFIDYEEWFRYRYNQWKGLSEKYIKIKKSKNSGVNIPSEE
CAASYVTKHCNGCICNLRDMEDIHKNINNQNELMKEMINIIKFDTDQYRTQLQNISNS
MEINPKSVKTAVDTTKDIVSYGLAGTMGVAAIGLQAGDFLGKKIQDLYNEFMKPVEKK
LDTSSKNLNIYEDPNIMVPAGIGVALTLGLLLFKMRRKAKRQVDMIRILQMSQNEYGI
PTTKSPNKYVPYGSQRYKGKTYLYVEGDTDEEKYMFMSDTTDITSSESEYEEMDINDI
YVPGSPKYKTLIEVVLEPSKRDTQNDIPSDNTPSYKLTDEEWNQLKDDFISQYLPNTE
PNNNYRSGNSPTNTNNTTTSHDNMGEKPFIMSIHDRNLYTGEEISYNINMSTNTMDDP
KYVSNNVYSGIDLINDSLNSGNQPIDIYDEVLKRKENELFGTNHVKQTSIHSVAKNTY
SDDAITNKINLFHKWLDRHRDMCEKWENHHERLAKLKEKWENDNDGGNVPSGNHVLNT
DVSIEIDMDNPKPINQFSNMDINVDTPTMDNMEDDIYYDVNDNDDDNDQPSVYDIPMD
HNKVDVDVPKKVHIEMKILNNTSNGSLEQQFPISDVWNI
>PFI1830c, 2264 bases, C8CE2A8E checksum.
```

MALGRPGGEDKYKNAKDAKHLLDSIGEEVYKEIVEKDDAEKYKEALKGKLSQASIWRE
TVSSLKTCDIIKYEGHKLLAARGDPCKKDTNGNDVDRFSVKEQAEYDNKKMKCSNGSN
GSNGKNEGACASFRRLNLCKKNMEKIPTSSTKHDLLADVCMAANYEAQSLIRDHPKYK
LKYSDSQLCTELARSFADIGDIVRGRDLYLGNPQEKGKRDELDKKLKDIFKQIHDDVT
TNGKNKDALKTRYNDDAKGGHFYQLREDWWIANRHTVWEAITCDDDDKLGGYSYFRAT
CGSGNNATLARDKCTCNNGDVPTYFDYVPQYLRWFEEWAEDFCRKKKKYVDIVKTYCR
GKDKNSEERYCSRNGFDCEKTKRAIGKLRYGKQCISCLYACNPYVEWIDNQRKQFLKQ
KQKYINVINGAPVSRRQKRDARNENYDGYESKFYKILKIDYQNVEDFLKKLSNEEICT
KVQDTEGGKINFENVNSGASGTNVESQGTFYRSKYCQPCPHCGVKMKDGKWETKGEDD
KCNIKLYRPKGGKDGTPIKILKSGEKQKEIETKLKAFCKTQNGGGGSDDCGGNSDSSL
CEPWQCYQPEELTKDDQGEEDVDDLEYENEVINAGGLCILKKKKEKKTDNDPDEIQKT
YNDFFNFWVAHMLKDSIYWKKKLQRCLQNGNRIKCGNQQCNNDCECFQKWIKQKKDEW
DRIKQQFRKQDFRKQGENIAGGMLGKEIESPDFVLQEVLKLEFYKEKSEDGSAQDTEN
SLDEEEAEELKYIREIIEKKNQEPAGANGQKTIMDKLIDYEKDEAKKCKDCQETQPQP
PGGDGVAKIGQPRSEVQPEEEEEEEEEEEEDKEDDDDLDRKDAEDQEAEVETAKEGD
VVTTKETTVEDPKVCDIVKTALENRENLQDACNLKYVKGKNYGWKCVPTTSGGDNNTP
GETTGSESERAEPTRAKRAAPSSPSGKDTGSVCVPPRRRKLYIHDLQSLIGKDSTTPT
QEDLLKWFIETAAIETFFLWHRYKKIKEKEIEEKRKRENGELPFLARSTSVEMKALSG
AGGGPELTGSGSDDPNDPNNIYSGKIPPPFLRQMFYTLADYKDILFGDKGEKNGYSDI
INGDKEMAQKEQNIKTAIESVFKPSGSSPPAPGKKNPVQTPKDWWTKYGPSIWEGMVC
ALTYKENSSGGEGKTIEKVKTADDKDLFDTLKGKYSDYEKVKLEDTSGAKPKDPKASP
SGEKTTLVDFISRPPYFRYLEEWGQNFCKKRTEMLGKIKEECRGDRGGHQYCSGDGHD
CTREDIKHNDMFSNLDCRPCYKQCRKYRKWIDIKFEEFRKQEKKYEEEHGKVITSSNN
ADDKNIYEKLKDYTSAANFLEALKHCKDGQTGGEKKSNDQDNKINFNDPKTTFGPLEY
CKTCPPNKVNCNGRRRTNPCTPDNGKGNKWIEVFDKIPKNNGKTTTIDVHMIDRRGPF
IKEYLKNSEKSFKDSYLFKSLRTEEWECRFNKEKKMDVCKLNKFDANIDLNEYTTFKV
LIVYWLEDFLYSYYILKKKIELCTQNKEQTCSEGNSKNGCVCVKKWVEKKKKEWDQIK
KHFNNREQKEGDDDMKSSVKMFLEDLQHLTELNKIMKPCTELYHFLKSLGCTETDSSE
NSKEDAIDCMLKKLEKKTESCSDEHSDQTHQTSCDSPPLVEDEDDTLHEEIEVKMPEI
CKNVVDTKKENDETGETCTSEDTITKETVETDSTDGPKQEEERIAPSAGDDGATRGPQ
EPSPKADSGDDNPETPQPKPPSNVFDNPHVKTALMSSTIMWSIGIGFAAFTYFYLKKK
TKSTIDLLRVINIPKSDYDIPTKLSPNRYIPYTSGKYRGKRYIYLEGDSGTDSGYTDH
YSDITSSSESEYEELDINDIYVPGSPKYKTLIEVVLEPSGNNTTASGNNTTASGNNTT
ASGNNTTASDTQNDIQNDGIPSSKITDNEWNTLKHDFISNMLQSEQPNDIPNDYTSGT
IPTNTNITTTSRHNVDNNTNTTMSRDNMEEKPFITSIHDRDLYTGEEYNYDMSTNSGN
NDLYNGKNNLYSGQNNVYSGIDPTSDNRGLTSGKHDSYSGIDLINDALSGNQPIDIYD
ELLKRKENELFGTNHTKKNTSTNSVAKNTNSDPIHNQLELFHKWLDRHRHMCEKWENH
HERLAKLKEEWENETHSGNTHTSDSNKTLNTDVSIQIHMDNPKPINQFTNMDTILEDL
EKYKEPYYDVQDDIYYDVNDHDASVVDSNAVDVPSKVQIEMDINTKLVKEKYPITDVW
DI
>PF10_0001, 2241 bases, 319C507C checksum.
MAAQSSGGGGGCGEEDKDAKYMFDRIGKEVHDEVKNAANVFKDYLKGNLTISTIFGEE
TVAFTDPCELIKEKRDELLAARGDPCGKGKEHRFSKERGAECDDNKIEGNVRSKGKGG
KSAGACAPYRRLSLCNKNFPNMNSKDSLKAKNDLLVDVCYAAKYEAESLIPYHDQYKL
TYGDSQICTVLARSFADIGDIVRGKDLFLGHNQRKKKLEERLEQMFKNITHSNAAKLS
VLSSKEIREYWWALNRDQVWKALTCDEENKLGGNAYFHATCSERNGGCSQAHEKCRCP
MTSDGKPNDQVPTYFDYVPQYLRWFEEWAEDFCRKKKIYVDIVKKYCRGERDGEKYCS
LNGCDCTKTVRARGKLRYGNRCTDCLFACHRYENWIDNQRKQFLKQRNKYTEEINGTS
TSSRTRRSARGGSDHKGYEKIFYEKLEGEYRTVDGFLELLNKEKACQEVKDSEGGKIN
FSEKHSGNSNDETKGTFYRSEYCQPCPYCGVRKTKGNQWENKSKNDQCNIKLYRPNPG
ESGTPIEILKSGEGETEIENKLNAFCAQTKNGSVVGGGSGGGNSDSKELYDEWQCYEF

KQLTTDGQEDDDDEDYDKDVRTGGGLCILEKKKEEGEAKSQNEPDEIQKTYNDFFYYW
VAHMLKDSIHWRTKKLDKCINNTNKSKACKNSNKCNNDCGCFQKWVDQKKEKEWDAIK
QQFRKQKDIGDETNCDPIVTLEGVLQIEFLKGDSEDSSEEKSENSLDAKEIQHLRQML
KQAGVAIGLDAFVALRGRCTEGGADSQNKTTIDKLLDEEAKEAKDCQSKHKDPCPKPQ
DGVAGRSAEHPEEQEEEEEDDDEEIEEKSEENDEESEKTVEDKETEAENVAEVPPTTQ
DNAEKPCDIVAELFKKPDTLKQACPTKYGSKAPTSWKCIPTKPNSDSSTTGGGSDTGV
PTTKSGATGKSGGSDTGSVCIPPRRRKLYVGELTKWAESKQVAQAQTTDTQTQVDAAS
TSTSQTSLLRDAFIQSAAVETFFLWHRYKKEWELRQKEAEQGQSGLPGIGVPGAGIPV
VPGAGIPGVVGANGLSQPVLGVDNDNPQTSLQNGTIPTDFLRQMFYTLGDYRDILFSG
SNDTTSGSKDTSSGSNDNLKHIVLNAGGDQKSKDEMQTIQTAISSYFSNSGKPSTSVT
PVTQPSGTTPQALWSKYAESIWNGMVCALTYRDSGDKGGKPQKVEAANGKLFQQLKDK
YGEYDKVKLEEEDSENQAKGSSSQHPENPSTTSENKPTHLTDFISRPPYFRYLEEWGQ
NFCKKRTEMLGKIKDNCTEDGGKTKKCSGDGHDCTDGKRRYNNMFDDLDCRPCYEQCR
KYRKWIDIKFVEYQNQKNKYGEEHEKLKTNSNGDNNCCTEIKEKTSAADFLKALKHCK
DGQTGGEKNKTHFENPETTFGPLDYCKTCPPNKVSCNVIRGRSGGRNGCNVKDNEDKW
KEVFDGISGNGGKSSTIEVEMIDRRGPYMKEYMKENSKNPFKTSRLFKGIRKEQWECK
VTNDDMHICKLDQFKENINLNPYTTFKVLLHYWLEDFLYGYYISKKKIEKCTQKEKNA
CDEETKKNCVCVKTWVEQKKKEWKEIKKHFKNREQKYGQGNDIKSKVKMFLETLIPLM
DLVNNKGKHESLDAFLKSYECKCAESSGKKGGKENNIVLCLLDMLEKKATPCLSSTSD
SSETPCENTPTTLDDDDPLEEENPVIHPQICGDIPTTKETVDEDACKRAEEPPKEPAP
TGPKKPAPTAGGEEDQTEKDTEVNPLAPAPADETFDPTILQTTIPLGIALALGSIAFL
FLKKKTKASVGNLFQILQIPKSDYDIPTKLSPNRYIPYTSGKYRGKRYIYLEGDSGTD
SGYTDHYSDITSSSESEYEEFDINDIYVPGSPKYKTLIEVVLEPSGNNTTASGNNTTA
SGNNTTASGNNTTASGNNTTASDTQNDIQNDGIPSSKITDNEWNTLKDEFISQYLQSE
QPKDVPNDYKSGNSSTNTNITTTSRDNMEEKPFIMSIHDRDLYSGEEYSYNVNMSTNS
MDDPKYVSNNVYSGIDLINDSLNSNNVDIYDEVLKRKENELFGTNHVKQTSIHSVAKL
TNSDPIHNQLELFHTWLDRHRDMCEKWNNKEEVLDKLKEEWENDTSTSGNTHPSDSNK
TLNTDVSIQIHMDNPKPINQFNNMDTILEDLDKPFNEPYYYDMYDDDIYYDVNDDNDT
STVDSNNMDVPSKVQIEMDVNTKLVKEKYPISDVWDI
>PF10_0406, 2215 bases, 274CBE87 checksum.
MVRTLDPEEELRGIEDTTAKHALDKIGEKIYEKAKKNAEQYRSQLHGRLSDARFEKAP
KEQQTPSGPCGLNHEYHTNATNGKSYPCRTGKEERFSQVHGGECDKNKISGNKDDEGA
CAPYRRLNLCVRNLENISNYGKINNDTLLADVCLAALHEGAAISSDHGKYQETNNDVN
ANICTMLARSFADIGDIIRGKDLYRGVNGNDKLEKNLKKIFGIIYEGLTKANGKKGQK
PAKDHYGDDENYYKLREDWWNNNRLMVWYAITCGAPKEAQYFRKTCGSGERTKDNCRC
AIHGVPTYFDYVPQYLRWFEEWAEEFCRLRKHKLQNAIKNCRGENNEKYCDLNGYDCE
KTIRGKKKLFEGADCKKCTVTCDNFVPWIKNQKQEFDKQKNKYAEEIKKAKADEETSS
RNINNIYEKDFYERLKTHYGSVNEFLKKLNEERICKDEPKVKEEKADAVDFTKDETNG
TFYRTTYCEACPWCGAEKVNGQNGKGGKWEPKNEACSQEEERIFDEQNITEIPVLTPE
EGQSGILKKYSKFCKNGATGEKSKNGNQIETWKCYYDENENKKDGNGAINFCVLQNDK
IGKKEEKSMHYHPFFWKWVTEMLIDSMYWRKELKSCINNKRGKCKNKKCNNDCKCYES
WVQQKENEWKLILQHFKKQGGFSIFGNDYNYALKALLDVKEILTNIKDTYGNVKELEG
INNMLEKENENNEQEASGGNNSQKKNTIDLMIDHEQKEAQNCKENNPDKCENTPGVRS
QTPPANQDDVHDDVHDDLHDNEITRRDLNIKVDPQEVHHEEEVVEEEPEATKAVKEDT
DGEGGSPKEDEEAAKRPSQEDPKVCETVDKALTETNLKDACPTKYGKNAPVSWKCIPT
SGSGVTATGGGSGEPKGRHRREANPAKASDSNQGAICVPPRRRRLYIQKLHDWATAVS
PQASGDKATQAAEPQASDKAAQVTEASASSSSSESNSVQTTPASTSSPSNSRDVDLVK
AFVESAAVETFFLWDRYKKENTKRQGGGAGGLGGVPGVELPELPVSNSVEKTPQTQLA
SGDIPTPFLRQMFYTLGDYRDILVRGGNTSDSGNTNGSNNNNIVLLASENKQEMENIQ
EQLKVFFSNSGNQSSTVGRNPSQSRVTPASLWGDFAQYIWNGMICALTYEEKTSGSDD
KGVKIEQNEGLKEALLEDKTNEPKKPQYQYKTVELKEENSDTQPITPGSSSPSGGDPI

NNPKLTQFVERPPYFRYLHEWGQNFCKKRKGMLENIKKECMKDSNSGSTGKKVQKCSC
YGEHCDDQLKDDPSIFPSLNCPSCGRHCSFYKKWIKKKRTEFEKQSNAYEQQKTKYQR
ETKGAENTDHDNGFYTRLQNLPDAAAFLQKLGSCSKKVSEKDNEKIFEDTEQTFKDAD
NCKPCSQFKINCKSGDGHCDNNKGNHCQSKSSIEAKHIKKEGGLAEDIDMLVSDNGTT
GFYDLSICISSGIFKGIRKDIWECGNVCGYNVCKPKNVNGETFEGQANGKNQIIFIRA
LFKIWLEYFLKDYNKINAKISYCKENGGTNICIKNCADKWIKLKKEEWGKIKEHYLEK
KHEDGDNDMTSLVTNFLRDVQPQTDVNKAIEPCKGLENFEKSCGLHGDANEQNKNGYQ
DAIDCLLDKLRKEATSCPGKTSDKTQAKCQESTPPDDDESLEEEENTVGKQQPSFCPK
PPEPKPEDEGGCETAQTTPKEPTADGGEGTENQPPVIKPEKEDTKSKDIQPQPPTAPP
EEKNNLPQPSHPLPSDNTSDILKTTIPFGIALALTSIVFLYLKVIYIVVYMYIYIYLW
MYMYVWKKTKSTIDLLRVINIPKSDYDIPTKRSPNRYIPYTSGKYRGKRYIYLEGDSG
TDSGYTDHYSDITSSSESEYEELDINDIYVPGSPKYKTLIEVVLEPSGNNTTASDTPS
DTQNDIQNDGIPSSKITDNEWNTLKDEFISQYLQSEQPKDVPNDYSSGDIPFNTQPNT
LYFDKPDEKPFITSIHDRNLYSGEEYSYNVHMVNTMDDIPKYVSNNVYSGIDLINDTL
SGNKNIDIYDEVLKRKENELFGTNHVKQTSIHSVAKLTNSDPIHNQLELFHKWLDRHR
DMCEKWENHHERLAKLKEEWENETHSGNIHPSDSNKTLNTDVSIQIHMDNPKPINQFT
NMDTILEDLDKYNEPYYDVQDDIYYDVNDHDASTVDSNTMDIPSKVQIEMDVNTKLVK
EKYPIADVWDI
>PF11_0007, 2182 bases, 8B10CD8D checksum.
MEPHGGSGGGGDVIDHQSAKHLLDSIGKIVHDQVEKEAADYRSSLQGRLKDATYSKKP
DNQEKPSDPCELKHEYRTNATNGTSYPCRAGKEERFSDTLGGQCTDQQIEGNDRNNGG
ACAPYRRLHLCNKNMVKMDTNNDDSSKAKHNLLLDVCMAAKYEGYSINTHYPKHQEKY
KDTGTASQLCTVLARSFADIGDIVRGRDLFRGNKKKSENKREKEKLEENFKKYFQQIH
EDVTSTSGKNRKALRDRYHKDGPDYYQLREDWWNNNRKMVWIAMTCEAGGSQYFRRTC
SNGISETNNKCRCTIETVPTYFDYVPQYLRWFEEWAEDFCRKKKHKLKDAIDKCRGQY
QDADRYCDLNGYDCEKTKRGINMYRWDHKCTGCFLSCSHFRTWIDNQKEQFHKQKKIY
DKEITRGGSDHKGYEKKFYGKLKGTDYKDVGKFLEKLSDEDVCTKFREDEGIIDFKNV
NSSSGSGGGEGSNKTFSHTEYCQACPLCGVEKESNGGGGNTKWKRKEDMDKCPPINLY
KPIDDKGGITINFLYSGDEPTEIGKKLNAFCLTHSGNSVVSSSSARGNGASRDKNGGS
DSQDLYQKWTCYEIDELEKDEKEDGVDDPQYEKDVKTGGGLCILEKTNGKEKVNKQKT
SHEMQKTFNPFFYYWVVHMLKDSIHWRTKKLDKCINNSNESKACKNNKKCNKECECFQ
RWITQKQQEWEQIKKHFRKQKNIDNVGGFFKLSHDDVLQQVLDKDLLLKSLQEAYGKP
EDIKHIKDLLEKEAAAVAVVLGGKDNTTIDKLLNHEDKDAKDCLKKQEECKEQERGGV
ARSGTGPRPPSAGDTVHEVEEKEEEEQEEEEEDEGDDGDGDGKGDVDQDNTVDVGAKE
APKVVEGPTTTQNEVNPCQIVDNLFSNPEQFKDACGLKYGPKAPTSWKCVTPSGKPGE
APSGDSTATGSSGSICVPPRRRRLYVTPLTRLAGDNTAASVSPQVRGETPLASTSAST
PSGSHRDPLLAAFVESAAIETFFLWDRYKKEWEAQKKAEQEQSGLLLGESSEALGMTA
ITSGAGAPKTPLTQQPVASSDDPQSKLQKSGDIPPPFLRQMFYTIADYKDILYSGSND
DNTKSSTYNDILKGDKEMKAKEEKIKEAIQKFFENGDKKPDGGTTPSSWWNNNAKHIW
NGMICALTYTDNTNGGPPTQDGEVKNALLDDNNKPKTNAGPKSNPHDYTYEKVVLKED
EENGPKTGSSTQPLTLKNFVERPPYFRYLEEWGEEFCRKQKHKLYIIEKECKVEANGR
DGKKNPKCSCYGENCNDNLIADPSIFPDFNCPKCGKHCSSYRKWINTKKTQYEKQKEA
YGEQQKKCQKENNGAQGNNGGNGVCGTLEATYTEAKDFLQKLGSCKNNSEEANIDFGD
KTKTFGPSENCKPCSEFKINCRNGNCGSDPKVECNGKNKNSISANHIIDDKNGNGNIE
MLVSDDSTTEFEVDELKVCKGAGIFEGIRKDVWKCGNVCGYNVCKPKNVKGEKGNGNQ
IIIIRALFKRWLEYFLQDYNKINAKISYCKENGGTNICIKNCADKWIRKKKEEWKKIK
DHYLEKKHENGDNNMKSLVTDILRSLQPQTELNKAIKPCGSLDAFESFCGLNGDENSK
KNNDNQDAIDCMITNLQKKIGECKKNHAKTSGSDCNTAPTSDTTLDDEDLSLEEENTV
EQPKFCPKPPEPKAEEKGACDPAPTTPKETASPADSGEGTKEHPSPPPTPDPAAPTSP
PLPPLTTALVTSTLAWSVGIGFAAFTYFFLKKKTKASVGNLFQILQIPKSDYDIPTKL
SPNRYIPYTSGKYRGKRYIYLEGDSGTDSGYTDHYSDITSSSESEYEEMDINDIYAPR

APKYKTLIEVVLEPSKRDIPSDDIPNNDTPSSKITDNEWNTLKHDFISNMLQNTQNTE
PNMLGYNVDNNTHPTMSRHNVEEKPFIMSIHDRNLLNGEEYSYNVNMVNSMNDIPMSG
KNDVYSGIDLINDSLSGGEPIDIYDEVLKRKENELFGTNHVKQTSIHSVAKPISDDPI
HNQLELFHTWLDRHRDMCEKWENHHERLAKLKEEWENETHSGNTHPSDSNKTLNTDVS
IQIHMDNPKPINEFTNMDTYPENSTMDTILDDLDKYKEPYYDVQDDIYYDVNDDNDIS
TVDSNNMDIPSKVQIEMDVNTKLVKEKYPIADVWDI
>PF11_0008, 2994 bases, 7EBE2595 checksum.
MGSQTSKFSKTVVGNETHNSARNVLEGFAKDIKRDVSNNAKRHGKVLKGNLRDAKFYH
DYSKLRDIPRSPCDLDFWFHTNVWRDKAYERDPCYGRQAKNNYNLEGAVCTNSKIKGN
ENKINDIGACAPYRRRNICDYNLEHLNERNVLNTHDLLGNVLVMAKREGESIVEKHPN
RGSSEVCIALARSFADIGDILRGKDMYVGYDEKEKNRRKQLENKLKDIFDNIYKDLTK
KKGRNGKKSALQERYNDPKGDFFQLREDWWALNREDVWKALTCSADDSEDYFIQSEGV
TKSFTNPKCGHGDNEVLTNLDYVPQFLRWFTEWAEEFCRIRKIKLGKVKNECRGETSG
KRYCSGDGYDCTKTDISRNIFYMDLDCPRCEEECRKYDEWIENKENELDKQKNKYTKE
IEKLKDNSKSNYDKNFYLTLTKKYGSINLFLDTLKEGSHCSYNTIEDKIDFNKANQTF
TSSKFCGACPFYGVKCNWKTCTEVKENEYKKKNKVDSTHTTEQPTAIDVLVTHIRGTN
IPEDLKDCKKYGLFKGMRKQAWKCQYINPYDECKLSPFVKDIDVDDRILFKVLFERWL
KYFIQDFNNVKDKINRCTKFEKGKDNTCIKGCKHKCECVEKWIKIKEAEWKKINQHYN
QQKKHYTYSVPRWVNSYLTHQHFSSDFINALEAFKNIRGLENLKECSSDTCKIEKIRT
IDDDLIKELISKLKDKCAMCKNQHKATKGKECCGKLPKTLNDQDDEEDEEYEAPPPPT
PPRTQKNPCVNGQNQKVRKIRSVRRVPKRMQKQASVRVPRARQGGEREQVVKNGRQDH
HLQRVLLVGEAEAEEPETAEEKKEEEKEEDTDGKVQPPPAATTPGVKPPCDIVEKHFK
DKHDNTGAIDHCNPKKDYPPWKNDKSLVDEDGVYMPPRRQKLCVINLEHFKENTSDDL
REAFIKCAAAETYLLWQKYKEDNNGGEDLQNQLKSGKIPEDFKRQMFYTFGDYRDFLF
GTDISKLNKHTEAVKTNIDRIFPPTERTNDTIRKEFWEKNAESIWQGMLCALSYNSND
KKMDPDVQKELNSTYNYDTIKNNLEDFANRPQFLRWFIEWSDEFCRERKKKEEKVGSA
CKNDYEGCANTKDNGNGNCVNACNAYKKYITDKKEQYEKQAKKFDIDKSQNKPGYEDY
SGKKASEYLKEKCINSSCDYMLKLKDNSNYWEKPHTTYDDNSLQNKCSCPLSPCEIVD
KTLGDKTSKSYAEGCKWKYGKMPLGLGWLCNDKEGEKGKEDGLCIPPRRKRLYVKDLE
TFSDHTTVGLREAFIKCAAVETFFAWHEFTKEKEREYKEEKQRNGELGFIDENDQIPK
DPDNPQNKIRKNGEIHEEFKSQMFYTLADYRDILFGNNIGIGNDMGKVKSNIDKVFAN
SSGKTPTAKKTTPKEWWEKNAKDIWEGMLCALSYDTKTKIKNEELRKKLIDPKNSNYM
YEKVTFSSDNNTNLSKFTERPPFFRWFQEWGEEFCRKKKIKIDKIEKECRGPYGRNHC
DGDGFDCSEIGPNENGSFAIFKCPSCAISCRSYKTWINTKKDEFKKQEKLYNKEIKDN
KSNYDNIYDKEFVKNLCTDYKSVDSFLKKLKEGPCCNKNTKDSKIDFKDTEETFRNAE
YCDPCPVFGVICNNGDCSNSTEKKCDAQEFKVTYDVKNKENPNKEVNMLVSDKTAKKY
PGDLNGVCENSSIFEGIREDKWSCGYFCGLDICTPNKTTGDIHDKQNAPIRVLFKRWI
ENFLKDHNKIKDKISLCINNENRNICTDVCRKNCECIDKWIEMKMKEWKIVRDRYVKQ
YNVADSVVYEVRRFLEGLQPQNDLEKVKGDVNDLRDLEELSECTNTVSTENRKCRKKD
VVESLLNKLKNEIRHCKNERDDSMGKESCKTLPEPTDDPQTDSDTHDTPDIPPGDVAP
TFCNVPANPCGDKSATNVVNVTEVAKEMHEEAHKDMLERSVKKVESKVKDSTVESVLR
ADASKGEYKHEGNPDDLKHNMCNITKEHTNYQKRGGYNYRGPCTGKGNGKDTRFVIGT
IWKDEDEKDETIKVLLPPRRRHMCTSNLEYLLHVNKGPLLKVEPDKINHSFLGDVLLA
AKYEAEFIKTNYTRLNGQNDNGAKCRAMKYSFADIGDIIRGKDLWGIQDFKDLQTKLV
TIFGKIKEEIPDIKKKYSSENPPYTTLREHWWEANRAKVWEAMQCPTIPPVTTSCDTT
TVTPLVDYIPQRLRWMTEWAEWFCKMQSQEYEVLVKQCRNCRSGICENGKDDCVKCTQ
ACNTYKQKIKKWEDQWKEISKKYKTLYQQAKGSVNGATTSSTTDEKDKDVVDFLKMLH
QKNTDNTIYTTAAGFIHQEAHMTDCQKQTIFCKNTSYNDKKKYAFRHPPHDHDDACAC
RPPSTPVDVSRKLDTQRDPKKEESEPESEEEEDDAEEEEEPAKETATTETTQPAAPAG
PPVTPVPELPGPPAPAGPAADGPIEDDEDAENEDDDDVGSATGTEDDDDDEDDDDEDE
EDSADEGEGEGDGGDVGEEEDEDHGGQEAEGVVPQPAAPQPPTPQLLDDPLLKTALMS

STILWMVGIGFAALTYFLLKKKSKSSVDLLRVLNIPKGDYEMPTLKSKNRYIPYRSGS
YKGKTYIYMEGDSDSGHYYEDTTDITSSESEYEELDINEIYPYQSPKYKTLIEVVLEP
SKSNGNTPSKGDGNTLGDDMVPTTNTFTDEEWSELKHDFISQYIQSEPLDVPKVGVSK
ELPMNIGGNVLDDGINEKPFITSIHDRDLYTGEEIKYNINMGTNSMDDPTYVSNNVYS
GIDLINDTLSGNQHIDIYDEVLKRKENELFGTNYKKNTSNNNVAKLTNSDPIMNQLDL
LHTWLDRHRDMCEKWNKKEELLDKLNEQWNKDNDGGDIPNDNKKLNTDVSIQIDIDEN
KGKKEFSNMDTNVDTPTMDSILDDLETYNEPFYDIFEDDVYYDVYDENPFVDDIPMDH
NKVDVPKKVHIEMKILNNTSNGSLEQQFPISDVWNI
>PF11_0521, 3119 bases, 4013EA8D checksum.
MGNAIPATPDPIFINESYKSARNVLENVARLIKAKATKDVKRHRNVLKGNLRNAKFYH
PFSKERPYYKSACDLDYRFHSNIWSGDNTYRHPCAGRNRNRFSYEGEAECRISRITGN
KTEHGACAPYRRRHLCDYIFHQVNDNYIKTSDDLLGNLLVTAKYEGESIVNSYANSGM
FNVCTALARSFADIGDIIRGKDLYLGNGDYKEKVSNNLRAIFNKIYENLNDPKLKKHY
QKDAPNYYKLRDDWWNANREDVWKALTCNAPYEAQYFIKPSDKEHSFSSEYCGHYKNG
DPLTNLDYVPQFLRWFEEWAEEFCRIKKIKLENVKNACRDEKKRKYCSLNGFDCTQTI
WKKKVFGRGNDCTNCSFKCFPYEIWLGNQREAFRKQKEKYAKEIEAYVTNRGIPKSSI
NNGYYKDFYNKLKEKTYNTVDEFINLLNEGRYCKKQKPEEENIDFIKTGEKGTFYRSD
YCQVCPDCGVDCSSGTCIEKKDDINCGKKINYEPPHGVKPIDIIVLYSGNEEGEITKR
LSEFCTDSSNNKGKNYEQWKCYYKNGDDNKCKMVKNSGNNITEEKIISFDEFFYVWVR
KLLIDSIKWENELNNCIDNTSTHCNKECNKNCECFDKWVKKKEDEWKNVKNVFENKNG
TSHNYYNKLNGLFKGFFFEVMDKLNKDETKWNKLIESLRTKIDSSKENIGTGNTQDTI
KVLLDHLKETATICKDNNTNEACETSRNRKTNPCAKPHGKKLATVKQIAQYYKRKAYI
QLNERGSRSALKGDASQGQYDRGGKADDFKTKLCEINEKHSNARSNSLNPCNGKDNNK
VRFNVGTPWQSGEKIATATDVYLPPRRQHFCTSNLEYLINGGHQAILNVKNGKINHSF
LGDVLLAAKYQAQHTMKDYKSKNDKEGICRAIRYSFADIGDIIKGTDLWDKDGGEIKT
QNHLVTIFDKIKAQLPKDIKGKYTGTKHLELRKDWWEANRDQVWKAMQCGNDNPCSGE
SDHTPLHDYIPQRLRWMTEWAEWYCKEQSRLYDKLKVCEECMRKGESCTKGSGECATC
KEACEEYNKEIKKWEQQWDAISYKYLMLYAKARITAINGGPGYYNTEVQEEDKPVVDF
LYNLYLQNGGKKGPPPDTHRVKALIARVKRDAARNRVKRADGSSATRVTATTTITPYS
TAAGYIHQEAHIGDCQKQTQFCKNKNGSDVSDTEADPTYAFRDKPHDHDTACKCKDRQ
PELVTEKKKDDEGEEQEDEPPKPKPPSTPNPCVRKDQSGTHIVSVEDVAQGMQRETHD
RVTKVPGLTADAKLGQYYQNGKVNTLNNECDISLEHSNRNTSRSQRPCYGKDGYDKMF
KIENGWKSGTDINKKHPHDVFLPPRREHFCTSNLEHLNTNVSGLTGPNAIHSLLGDVL
LAAKKEAGFIEEKYKKPITPEGFKENVTMCRAIKYSFADLGDIIKGTDLWDGNGGEKN
TQSNLETIFGKIKDNLGKSVENYKDDSYPYIKLRSDWWSANRDKVWKAMTCPQNGIKC
DKDPPLDDYIPQRLRWMTEWAEWYCKYQAEAYKTLQKGCDECKNKSKKCEKDKPQCKN
CTKACDEYWKKIEKWENQWTKIKEKYEQLYQKAEKNDGDTSSGTDDVKEENDVVAFLS
QLYEKNKGSNTIYSTAAGYIHQEAHIGDCKEQHVFCDKKNGDKPTNGEEKVDNEKYAF
KHPPHEYEVACKCETNKKPEAPPPPPPPQPPPPPPQPARPAEDQIEHDHRARSDGGQR
QRPLPLPPVQQPPVQQPPVQQPPAQPPQQPPQQPPQPPPPAPEGGGLGRSLPRADRNG
EITDSEEEEEEEKETPKVDEETVEEETTKEEEKAPQDQEAENPDTKVEDKVEGSGPKE
GEAPPKVDGVKPACDIVATLFNDTNKFKDACTLKYGPKAPTSWKCVPTTSGGSGVTTT
AGSSVTTTPPSNSGAICVPPRRRRLYVKDLEKIGDGEVTQVQLRDAFIKCAAIETFFS
WHKFKKDKEKKKKPQQQHALHPLFVQDDDEEEEEEEQENPQEQLKRGKIPDDFLHQMF
YTFGDYKDILFGKDMGNDVGDVENNINKILPNNSAKSANGHSRQEWWKKYAEDIWKGM
VCALSHSVSASDKDSMQKNLTDKYKYKEVKFTSKSSKHNIPLSEFEKKPQFIRWFEEW
TEDFCRKRTYKLKKAKQECRGKEEGDKYCDGDGYDCTDKDKLRNKSFIYLQCSGCQKE
CIKYKKWIVNKRNEFNKQKKKFENEIKTVNGTNEDKYDEEVYKNPKLMYPLFNDFVSK
LNESPYCINSNVEDKIDFNKNGETFGSSQYCKACPVYGVNCKNSKHKCLPISIEKYNS
TKSSRGENNDDKTPSIIEVLMLDRKGDNKNNVFINDCKNAGFFEDASFQKWNCQKKNG
VDQCKLTEFDDNMDDDKEMEFNVFFQRWLRYFVYDYNILKDKIKACVITKDEKSNKCI

NGCKGKLECVEKWLNKKSTEWGDIKEHYKKNPKFVNESIPYLVKSYFDQIYFDKDSTK
AKEVVEEETERIKLWGCTGPNNCNEQEQSKNEDFITNLIEKLKNKIDICKAQPDGPDE
NCLPFPSDPEPEPEEDPDTSITSSPPDFCKNLNTPDGEKSPIDTEESAVPVDHDVTKE
EEEEKEEEKDKGDEEEEEEEEEEDEEEEEEAEEEELDDEIHDDDSDSKTEDEDQDELD
PAGPLSPSEPRPKRLSREFPSTQLKNAMLFSTILWMVGIGFAAFTYFFLKKKPKSPVD
LLRVLDIHKGDYGTPTSKSSNRYIPYVSDRYKGKTYIYMEGDTSGDDDKYMFLSDTTD
VTSSESEYEELDINDIYVPGSPKYKTLIEVVLEPSKRDIPSDDIPSSDTPMNKFTDEE
WNELKQDFVSQYIQSEPLDVPQYDVSTELPMNIVGNVLDDGMDEKPFITSIHDRNLYS
GDEISYNINMSTNTNNDIPKYVSNNVYSGIDLINDTLSGNQHIDIYDELLKRKENELF
GTNYKKNTSNNNVAKLTNSDSIMNQLDLLHKWLDRHRDMCEKWNTKEELLDKLNEEWN
KEKDGGNVPSDSNKRLNTDVSIQIDMDDGKPKKEFSNMDTILDDMEHDIYYDVNDENP
SVNDIPMDHNKVDVPKKVHVEMKILNNTSNGSLEPEFPISDVWNI
>PFL0005w, 2178 bases, 6A09F24C checksum.
MGPQPAVTDYSKATNVKYLFDLIGETVQKKAKEKADVASRKYFEELHGDLSKATYKKD
KNPEGTTPPNPCKLEYQYHTNVTKGEDKEYPCLGRKTVRFSDKEGAECYKTKIKDSTT
DTVGACAPYRRLYMCDRNLEHIEPTKITTHNLLLDVCLAAQYEGQSISQNHGKHQLSY
PDSPSQLCTELARSFADIGDIVRGRDLYRGNNRENDKLEKKLKGYFKKIYDNLVEKKK
EEAETDYKDDAPDFYQLREDWWALNRQDVWKAITCDAHDSRYRKMGADGSITESAMRQ
CRNVADVPTNFDYVPQYLRWFEEWAEDFCRKRKHKLKDAIQKCRGQDGTGKDRYCDLN
RYDCKRTISAKHELVQGEECKKCSVVCIPFGPWIDNQKQEFEKQKNKYTNEINKKHDE
TTKEISGNRRKKRSLTTKNYKGYDEEFYKIFKDEYPDVDKFLDLLSKETACESQPYDE
PRTISINFKNYKNPDIFSHTEYCQACPWCGMTCTFDGKCTKNPEELCHHKIVQKEYPD
TNTTDIPILTPDTTKSNIVEKYRNFCNSSDDNNSDQINNWQCHYDESKKSGQNDNCVE
GTWQNFKKDQKVTSYNAFFWKWVSEMLDDSIKWRAELDKCLKNDKKTCGKKKCNRDCK
CFKKWVEQKKEKEWKAIKKHFKKQKDMIETGMPPEMALKILLNDVFLQDMEKAQGDPQ
HIAKIKELLKKNDEKVNNLSNMETIFDFLLQEEEQDAQKCVSNNPEKCEETQKPPTDG
APGGAGPSPDTGTDDNLEDIDSDGEEDDDVSHVDEEEPEDNPVEGSSEEEKQEVVKDT
EAAVPKQDTQPKEEVNPCKIVEELFKSTKNFEDACGLKYGKNYGWKCVPTTSDKGSEP
TARGHSHVARSADGAPSGDKDGAICIPPRRRKLYLHKIEGVDTTDDKSLRKWFIESAA
VETFFLWDRYKKLNTPQSGSPLLGGGLPGVGVENGDDENNPEKLLQKGEIPDGFLRQM
FYTLGDYRDILFSGDKDKKNGYSDIVSGDNVIKERENTIKEKIASFFQNGNKEGTPHV
PKNPVQTPQTWWKDNAKHIWHGMICALTYEEKTSGSDGEKKIEKDDAVYKKFFGTPNG
NPLPQPGTNGTSNEPISQYQYDQVVLKEENNGAMSTSPKSTSAAPSDNTPTTLTQFVL
RPTYFRYLEEWGQNFCKERKKRLAQIYEDCRGNDKVCSGDGEDCEEVRKQDYSKISNF
NCPGCGRECRKYKNWIKTKRTEFDEQKKAYVDRKDKYKTENKGAESKHHSISDQNFVK
KLGTDYASIESFLEKLGSCSKNNKDNGDGTINFKEPDVTFKPADNCKPCSEFKVKCEN
GKCSGGGKKVNCNRKNTIAATEIANMINSTEGVFMTVSDNSDHKFEGGLEPCGSANIF
KGIRKDVWKCGEYCGVDICEPNTFDGKQNGKEYIQIRALLKRWVEYFLEDYNKIKHKI
SHCMRKGEKTICINDCVEKWINIKKKEWETIRERYVKQYTTGHSDIYKVTSFLEDPQF
HNEVLKAIKPCGDLDKFQNSTDCTVAGSSENGVTNKKDIVECLLENLKTKAKTCPNQA
NGENQTCDSLPHVEDDDDEEPLEETEENTVEQPNICPQLPKPPPQPGDEDGCKQASPA
PSEGTENQPPVIKPEEEAPAPRTPRPRPKPPQEPQPYLPPALKNAMLSSTIMWSIGIG
FATFTYFYLKKKSKSSVGNLFQILQIPKSDFDIPTKLSPNRYIPYTSGKYRGKRYIYL
EGDSGTDSGYTDHYSDITSSSESEYEEMDINDIYVPGTPKYKTLIEVVLEPSGNNTTA
SGNNTTASGKNTPSDTQNDIPSGDTPNNKLTDNEWNTLKDEFISNMLQSEQPKDVPND
YSSGDIPFNTQPSTLYFDNNQEKPFITSIHDRDLYTGEEYNYNVNMSTNSMDDIPISG
KNDVYSGIDLINDTLSGNHNVDIYDELLKRKENELFGTKHHTKHTNTYNVAKPARDDP
LHNQLNLFHTWLDRHRNMCEKWKNDNERLAKLKEEWENDTSTSGNKHSDIPSGKLSDT
PSDNNIHSDIHPSDIPSGKLSDIPSDNNIPSSNKTLNTDVSIQIDMNNPKTTNEFTYV
DSNPNQVDDTYVDSNPDNSSMDTILEDLDKPFNEPYYDVQDDIYYDVHDHDTSTVDTN
AMDVPSKVQIEMSVKNHKLVKEKYPIGDVWDI

>PFL0020w, 2860 bases, D43EDE93 checksum.
MTSCSPENTKMAPKTRASDYKDAQDAKHLLDMIGEVVQKQAYSEALTRGRSALQGRLS
DARFLHGTSWEQANQNVCSLKHTHDTNVRWGVIHPCDNRLGNLYSEESASQCSTSRIS
GNNSDSGSCAPYRKLQLCDYNLEKITDANVTNTHNLLVDVLLAAKHEGDSLSKYMKEN
PKIIPKSNVCTVLARSFADIGDIIRGKDLYVGNRKEKEKEKLQKNLKNIFAKIHSGLT
KKGAEARYKDNDENYYQLREDWWEANRAKVWEAMTCSVEDAYYFRQTCGGEKTASTNK
CRCVNTDPPTYFDYVPQYLRWFEEWAEEFCRIKELKLENVKTRCRGEKDGEKYCSRNG
YDCTGTIIKRNIFRPDPECTNCLFECNHYQDWIDNKKKEFEKQKKKCEKEIYNTSTTN
KRTNNNVNVMYYDDFYKELQGRYRTINELLNSLNEETKCKSTENTDKESKIDFNDSEK
TFSASKYCKPCPDCGVVKQDDGNFKVREETDAECQVKNDATPLDGVKPTDIEVLYSGV
ERKHISEKLSEFCSKPDDQYGIKNEKWECYYKSSVDNKCIMQTNNQKVESHSKIMKFY
EFFIFWVTYMLNDCIDWKKKITKCINNGTKWRCKNKCKNNCKCFEKWVKKKQVEWNTM
KEQYEKQPDLVNNRHFTTLEWFLEGQFLKTIEKAYGNEDAIEKIQEFLQKKSKQEDDE
IKDKRDIIDILLEHELDEAQECKDNNREEKNCSKEPHDDLDDEDDLYEDETHYNPCSA
QPGGRYTVRVKDIAKQMHRRAKTQMRNNSVVDDDNKLEGDIFKVTFRNGGKGSELQGE
NICNINTTHSNDSRGSKGEPCKGKDGSGERMKIGTEWSYIKEKEISYKDFYLPPRRQH
MCTSNLENLDVESVTKEDKASHSLLGDVQLAAKYEAENIKKLYVENNDRKDQEAICRA
VRYSFADIGDIIRGRDMWEHKDQTTLQNHLKSVFKNIKEKLPGIQGKYADDERNIPAY
KLLREDWWEANRRQVWKAMTCENNGIKCDAHNAKHPPPDDYIPQRLRWMTEWSEWYCK
MQSQEYEKLKEGCKKCMENKGKNCIKDTPECNDCKQACEEYNKKIKEWEKQWHKIQVQ
YLMLYYEANTTARYGIHAYAYAVGEKDKPVVAFLQKLQEANKSSASKRSKRSTDGTTT
DTLTPTTPYSTAEGYVHQEATMNCDTQTQFCEKKHGGTTPTGTNDTDAPYTFKQPPPE
YKDACECDGKSPQAPKKEEEKKDACTIVNSLLQNKKATDPIDGCNQKSDANWDCDPST
FVNDNNNGACMPPRRIKLCIYYLENHTQKGNIKKEFQLREAFIKCAGAEIFLLWQKYK
TDNNSDTKLQTQLESGQIPEEFKRQMFYTFGDFRDLCLGNDIGKADNTKDISTTVNRI
LSVTNGETQTTAENWWEKNAESIWQGMLCGLSHAVSARDKETVQKTLTNNPSYKYDLV
KFSGSDNPPTLEKFAQTPQFLRWFIEWSDEFCRERKEKLDKLLKECKEYECNEENMDE
KKKKCEDACKVYEEWLQGWKDQYKKQSKKFTTDKEKKEYKDDPDLDGATYAYQYLSKK
LKPICQNGTTTDKCDYNCMENASRQPQTSACSQEQQQQGNTSSTQNHFPEAFDCPPKE
IGDRCNCPKLPEPKYCVDKTAYDIRKDAEKNIKDIYSSIKGNGEIYKGKCNPTETKID
ENGINTCEFKKRYPNANNLLDTSCDNKGNERFKIGQKWNYKYIYKIGKNLYIPPRRQH
MCINHLKQIIKYTDTDSTTLLKKVQEVAKKEGDDIIKKLLPKYPCNEDVICKAMKYSF
ADLGDIIRGRDIYIGNNNQIENKLQQVFKNIYDSNKHKLSNYNDNGDSKYTKLREAWW
DANRKDIWKAMTCAAPEEAKIYITKEGGYISPLTSTKNHCGHKDDPPDYDYIPQPLRW
ISEWGEQFCLYQKHLLESMKICENCKKNSGECKQSVHGACMDCKKKCEEYKKFIEIWK
KQFETQNEAYKEIYKKATSNGRYFNGIDENTKKFVKKLKDTCKTGDLASADKYLENGS
VCRRFKFVKTDTHIKNYAFHNTPLSYKEHCECAKNFDPLDECPVDKDECKKYGRYSCR
KNHYNKNPIEWTNHFVKKSIRNYEAVMVPPRRRQLCLIGNRRFVGRVKDEKMFKEYLL
RDASSEAKMLSQYYNFDNEKALQAIKYSFADIGNIIKGDDMLDDGISEKIENIFEHKI
NKRTHSSSLSSSSSSGPNITPSTWWEKNKEKIWYVMMCYYTGEHKTATSCPSHNDIDN
EDQFLRWMTEWAEYFCKEKKKEVEELIEKCKTEITTKTYPTSNQNKQSSCYKVLEKYN
HWLYNRKLEWNDISKKYQTYYNENSKSTQLKRSAQEYVDQRCKECTCNFDEIIEKYDK
SGNGISIVHSLLKDLNPGRTCGPDTSHKSDTKPPLPPPLPPAVDPPQAEEPFNRDILE
KTIPFGVALALGSIAFLFLKKKTQTPVDLFSVINIPKSDYDIPTLKSKNRYIPYKSAQ
YKGKTYIYMEGDSDEDKYAFMSDTTDVTSSESEYEELDINDIYVPGSPKYKTLIEVVL
EPSKRDTQNDIPNDIQNDIQSDGIPSSKITDNEWNTLKDEFISQYLQSEQPNDYKNGN
IPLNTQPNTLYFDKPEEKPFITSIHDRNLYSGEEYSYNVNMVNSMDDIPINRDNNVYS
GIDLINDALNGDYDIYDELLKRKENELFGKNYKKNTSNNSVAKLTNSDPIMNQLDLLH
KWLDRHRDMCEKWKNNHERLAKLKEEWENETHSGNTHPSDSNKTLNTDVSIQIHMDNP
KPINQFTNMDTILEDLEKYNEPYYDVQDDIYYDVNDHDASTVDSNNMDVPSKVQIEMD
VNTKLVKEKYPIADVWDI

>PFL0030c, 3056 bases, 7E8A44CD checksum.
MDKSSIANKIEAYLGAKSDDSKIDQSLKADPSEVQYYGSGGDGYYLRKNICKITVNHS
DSGTNDPCDRIPPPYGDNDQWKCAIILSKVSEKPENVFVPPRRQRMCINNLEKLNVDK
IRDKHAFLADVLLTARNEGERIVQNHPDTNSSNVCNALERSFADIADIIRGTDLWKGT
NSNLEQNLKQMFAKIRENDKVLQDKYPKDQNYRKLREDWWNANRQKVWEVITCGARSN
DLLIKRGWRTSGKSNGDNKLELCRKCGHYEEKVPTKLDYVPQFLRWLTEWIEDFYREK
QNLIDDMERHREECTSEDHKSKEGTSYCSTCKDKCKKYCECVKKWKSEWENQKNKYTE
LYQQNKNETSQKNTSRYDDYVKDFFKKLEANYSSLENYIKGDPYFAEYATKLSFILNS
SDANNPSEKIQKNNDEVCNCNESGIASVEQEQISDPSSNKTCITHSSIKANKKKVCKH
VKLGVRENDKDLRVCVIEHTSLSGVENCCCQDFLRILQENCSDNKSGSSSNGSCNNKN
QEACEKNLEKVLASLTNCYKCDKCKSEQSKKNNKNWIWKKSSGKEGGLQKEYANTIGL
PPRTQSLCLVVCLDEKGKKTQELKNIRTNSELLKEWIIAAFHEGKNLKPSHEKKNDDN
GKKLCKALEYSFADYGDLIKGTSIWDNEYTKDLELNLQKIFGKLFRKYIKKNNTAEQD
TSYSSLDELRESWWNTNKKYIWLAMKHGAGMNSTTCCGDGSVTGSGSSCDDIPTIDLI
PQYLRFLQEWVEHFCKQRQEKVKPVIENCKSCKESGGTCNGECKTECKNKCEVYKKFI
EDCKGGDGTAGSSWVKRWDQIYKRYSKYIEDAKRNRKAGTKNCGPSSTTNAAENKCVQ
SDIDSFFKHLIDIGLTTPSSYLSIVLDDNICGADKAPWTTYTTYTTTEKCNKETDKSK
LQQCNTAVVVNVPSPLGNTPHGYKYACQCKIPTNEETCDDRKEYMNQWSCGSARTMKR
GYKNDNYELCKYNGVDVKPTTVRSNSSKLDDKDVTFFNLFEQWNKEIQYQIEQYMTNT
KISCNNEKNVLSRVSDEAAQPKFSDNERDRNSITHEDKNCKEKCKCYSLWIEKINDQW
DKQKDNYNKFQRKQIYDANKGSQNKKVVSLSNFLFFSCWEEYIQKYFNGDWSKIKNIG
SDTFEFLIKKCGNDSGDGETIFSEKLNNAEKKCKENESTNNKMKSSETSCDCSEPIYI
RGCQPKIYDGKIFPGKGGEKQWICKDTIIHGDTNGACIPPRTQNLCVGELWDKRYGGR
SNIKNDTKESLKQKIKNAIQKETELLYEYHDKGTAIISRNPMKGQKEKEEKNNDSNGL
PKGFCHAVQRSFIDYKNMILGTSVNIYEYIGKLQEDIKKIIEKGTTKQNGKTVGSGAE
NVNAWWKGIEGEMWDAVRCAITKINKKQKKNGTFSIDECGIFPPTGNDEDQSVSWFKE
WSEQFCIERLQYEKNIRDACTNNGQGDKIQGDCKRKCEEYKKYISEKKQEWDKQKTKY
ENKYVGKSASDLLKENYPECISANFDFIFNDNIEYKTYYPYGDYSSICSCEQVKYYEY
NNAEKKNNKSLCHEKGNDRTWSKKYIKKLENGRTLEGVYVPPRRQQLCLYELFPIIIK
NKNDITNAKKELLETLQIVAEREAYYLWKQYHAHNDTTYLAHKKACCAIRGSFYDLED
IIKGNDLVHDEYTKYIDSKLNEIFDSSNKNDIETKRARTDWWENEAIAVPNITGANKS
DPKTIRQLVWDAMQSGVRKAIDEEKEKKKPNENFPPCMGVQHIGIAKPQFIRWLEEWT
NEFCEKYTKYFEDMKSNCNLRKGADDCDDNSNIECKKACANYTNWLNPKRIEWNGMSN
YYNKIYRKSNKESEDGKDYSMIMEPTVIDYLNKRCNGEINGNYICCSCKNIGENSTSG
TVNKKLQKKETQCEDNKGPLDLMNKVLNKMDPKYSEHKMKCTEVYLEHVEEQLKEIDN
AIKDYKLYPLDRCFDDKSKMKVCDLIGDAIGCKHKTKLDELDEWNDVDMRDPYNKYKG
VLIPPRRRQLCFSRIVRGPANLRNLKEFKEEILKGAQSEGKFLGNYYNEDKDKEKALE
AMKNSFYDYEYIIKGSDMLTNIQFKDIKRKLDRLLEKETNNTEKVDDWWETNKKSIWN
AMLCGYKKSGNKIIDPSWCTIPTTETPPQFLRWIKEWGTNVCIQKEEHKEYVKSKCSN
VTNLGAQESESKNCTSEIKKYQEWSRKRSIQWEAISEGYKKYKGMDEFKNTFKNIKEP
DANEPNANEYLKKHCSKCPCGFNDMQEITKYTNIGNEAFKQIKEQVDIPAELEDVIYR
LKHHEYDKGNDYICNKYKNINVNMKKNNDDTWTDLVKNSSDINKGVLLPPRRKNLFLK
IDESDICKYKRDPKLFKDFIYSSAISEVERLKKVYGEAKTKVVHAMKYSFADIGSIIK
GDDMMENNSSDKIGKILGDGVGQNEKRKKWWDMNKYHIWESMLCGYKHAYGNISENDR
KMLDIPNNDDEHQFLRWFQEWTENFCTKRNELYENMVTACNSAKCNTSNGSVDKKECT
EACKNYSNFILIKKKEYQSLNSQYDMNYKETKAEKKESPEYFKDKCNGECSCLSEYFK
DETRWKNPYETLDDTEVKNNCMCKPPPPASNNTSDILQKTIPFGIALALGSIAFLFMK
KKPKTPVDLLRVLDIPKGDYGIPTPKSSNRYIPYASDRYKGKTYIYMEGDTSGDDDKY
IWDLSSSDITSSESEYEEVDINDIYVPSFPKYKTFIELVLEPSKRDTFNTSSGDTFTN
KLTDDEWNQLKQDFIEQYLQNIQKDFILHDSMDEKPFITQIQDRFLDSSHEEVTYNID
WNVPENINRITNNMDDPKYCSNNMYTGTDLINDSLNGNQYIDIYDEMLKRKENELFGT

YHTKYTTFNSVSKQTPSDPIINQLDLYHKWIDKHRDICEQWKTKEDMLYKLNEVWNME
RKEYLLDIQPSTLDDIHKINDETYNIISTNNIYDHPSQETPLQLLGSTNIIPSYITTE
QNNGLRTNISMDTYIDETNNNNVVATSIIGDDQMENSYNC
>MAL7P1.5, 235 bases, 6472CB67 checksum.
MFHYIYKIYIFTIILCASNLFNNNGVENGTYKLSYHNGGIQFRMLAQNNTNKKSNGNT
LTNILLKDKGGKGSKKKNPDDQISDLVSLVDNMNITQEKKNEIKNLTLKYMNSDDIKE
KNKSINELKKYSNNEECKEQMDSYLMHLRMQNDIKCLKRKNLWNNIWINYITLSLIII
MIALLFLLPGSQPYYIFYSAFSLLIFIIYIVASYFPDMKIGFKKLKTKLNTFFQNKKQ
ITK
>PFL0935c, 2256 bases, D24E0D92 checksum.
MVAAAKGGGSSQDAKHVLDEIGKDVYETVEKDAKIYKEALKGNLQHAKGMGERASSNK
TCTLVKEYYEHFNGDANSNRYPCKELSGKMGENRFSDTLGGQCTNKKIEGNKNNCGAC
APYRRLHLCHHNLESIDTTSTTSDTLLAEVCYAAKFEGETLTTQHGQHQQTNPGTASQ
LCTVLARSFADIGDIVRGKDLFLGNDEEKKKRDELEENLQKIFAKIHSGLTSTRGKQN
GELQKRYKGDAKKNFYQLREDWWDANRHTVWEAITCGAGESDKYFRQTACGTGTPTHK
QCRCDDKANVDPPTYFDYVPQYLRWFEEWAEDFCRKKNKKLKDVKTNCRGDSGTDRYC
SGNGYDCTQTIRAIGKLVIGEHCTNCSVWCRLYEKWIDNQKLEFLKQRNKYQTEISGG
GASGGASGRSRKTRAATIKYEGYEKKFYEEFKKKGNYGTVDAFLGLLNNEKACKDINE
EKEKIDFTKNVEDDKNINKEGTFYHSQYCQPCPGCGMKKTKNGNGWEEKDTNQCNIKL
YKPTSSAKPTEIKILKSGEKHDDIKEKIEQFCKTQNGSDGSGGGNSEKKELYDEWKCY
QIGELTKDGQEGMDDPDYDRLVETGGGLCILENQKKKEKSDPKSQNEPEQFQKTFNEF
FYFWIGRFLNDSMYWRGKVNSCINKSKREKCEEECKKECECFQKWIGKKKEEWDKIKE
HFKTQDFGNDALLGPEMKSPSYVLKFVLNIDELFQNIKSGYGNVKELKGINKILDEEK
KREEEERADGVVGVASGGDNQNNTTIDKLLDHEDKDATKCKNCQEPTKPASKPEDLAR
SGASPDTPREDSPPADLDDEEHDEDDEDEDDDAEEEEEEEKKEEEEEHKEEENEAAPE
EKKEEGSSSPEEVEEKAKEAPAGPDACNIVQTLFSSIDKFSDACTLKYGKTAPTGWKC
VTPSGDKAATSSEGKGSSDGGAKDGVGVNGGALQRNKRDLATPSAKSGDTTGGKDGAT
GKSDGSICVPPRRRRLYIQKLHDWAEKVGDTATQPQVDTPSQSDKLRDAFIESAAVET
FFLWHRYKKIKDKEKLEEQQRQRENGELPGLSSSGDGDSNDPQSKLEKGEIPDGFLRQ
MFYTLADYKDILYSGSNDNTKSSTYNDIINGDKEIQEREKDIKGAISTYFSNSGTTPT
PPVKPSVTTPQTWWELHGPHIWNGMICALTYKENGIDKPEVDTAVRAQLWDSGKNTPQ
NEKYQYTNVKLEEEASGAKINIPDTSGDNTPTLNTPTLKNFVEIPTFFRWLHEWGSDF
CRQRKRMLKNVKHNCRNIERGGHEYCGGDGHDCTRDGIQHNNMLADPDCRDCHIQCRK
YRKWIEKKFEEFHNQKNTYGKEKQKLNGNSNDGDNTKFFKEIENRSFDQFLTSLKHCK
NNEGDGSDPKNKIDFKEPLKTFGPLEYCKTCPPNKVNCNGPSRRSGGNDQCTAVNGNE
WEKIFSENGGNSTTIDVHMIDRRGPYMEKKSQKLENSENPLFKTSRLFKGIREQKWTC
KFKDKNTDVCKLDKFDQEVDLNQYTTFKVLLIYWLEDFIEGYYLLKKRKIIEKCTQKG
EKTCDDESKNDCACVKKWVEKKTTEWEQIKEHFKNRNQKDGDGNDMKSSVRQLLDPLI
YRMDLANGKGKINELKEFLKSYECKCVDNAGNSEKDVVECLLQKLETKAKKCKDPSSG
ENQKTPCQEYTPPDDDEDLLLEEENTVKAPTFCPKPPKKEEQTEETCDKAADEKVEET
VAQNEENNIPPAGPAPPADSPPAGPATDSGKENVVPEPPAPAAPPSPPIPPLATSTLA
WSVGIGFAAFTYFYLKKKTKASVGNLFQILQIPKSDYDIPTLKSKNRYIPYRSGTYKG
KTYIYMEGDADEDKYTFMSDTTDITSSESEYEELDINDIYVPGSPKYKTLIEVVLEPS
GNNTTASGKNTPSDTQNDIQNDGIPSSKITDNEWNTLKDEFISNMLQNQPKDVPNDYS
SGDIPFNTQPNTLYFDNNQEKPFITSIHDRNLYTGEEYSYNVNMVNNDNIPINRDNNP
YSGIDLINDSLNSNKVDIYDELLKRKENELFGTEHHPKHTNIYNVAKPARDDPITNQI
NLFHKWLDRHRDMCEKWDTNNKVDILNQLKEEWENDNSNSGNKTSGNITPTSDIPSGK
LSDIPSTNKMLNSDVSIQIHIDKPNQVDDNIYLDTYPDKYTVDNINPVDTHTNPNLVG
NINPVDQNSNLTFPSNPNPAYDNIYIDHNNEDLPSKVQIEMSVKNGEMAKEK
>PFL1950w, 2646 bases, 66BA7C93 checksum.
MVRSSSRASRPKYYQATSAKELLDQIGQSVHAEVQRDAKKYVSELKGNLSRATYPKDE

SPKGTTSPDPCHLDYRYHTNVTKGHGKEYPCEDRPEVRFSDTEGAQCDKSKIKDNKGK
SEGACAPYRRSSLCDHHLSYMNAGKTNTTDNLLLEVCMAAQYEGQSIRGQHDKHKLDN
NNSSSQLCTVLARSFADIGDIIRGRDLYRRDKGEETKLEKNLKEIFKNIYNELTTKNG
TKERYNDTDNYFQLREDWWEENRETVWKAITCHVVSGNNYFRHTCSDENHPTATQGNC
RCIGATVPTYFDYVPQYLRWFEEWAEDFCRKKKKKLPNVKTNCRGENNKKYCSGDGFD
CTKTIRAKYIYAIGDECTKCSFWCGFYKKWLENQKQEFLKQKKKYETEISGGGGRKKR
AARSSGSNSNYDGYESKFYNILKGIPEGGLDKFLDLLNKEEVCTKFSEDEGTIDFTKH
DNKNNDQKGTFYYSKYCEICPECGVRKGTFEEKPKNESGECDGKKLYTITDYAESTDI
NVLSFGDERDQIKKKIDEFCDKNDINKQELTEQWKCYEEQDIENDGQDDYKDDVNGSG
GICILEKTNGDKNGKKQKTFNDFFHFWVRHLLNDSIEWRDKLKKCIEDPEKKCKNGCN
KKCECYERWVDKKKGEWKNIKDHFDKQPGFDQTFPPYYVLEDVLEESYFPIIQEAYGD
STAIQGIKKTLEKKKKEKGANISEEKTILDEFLNHELKDAETCKNCEPRKFKNPCSGD
TSGDSNKQYEAVANTVAQILQGKAQKQLHGNGSRNALKGNIQNAKINNGRKPNPLTDA
CQITKNHSNGKGDSNNPCNNKGNRLKIGQVWSIKNDTSYTDVYMPPRRQHMCTSNLEK
LNYASVIGSNNVNDKFLVEVLHAAKSEAEFIKKKYNEKQNDGKNGLRKDQATTCRAIR
YSFADIGDIIRGKDLWDDNNDAKSLQTNLKAIFKKIKEKHPGIEGNDKYVKDNENKQL
RSDWWEANRRQVWNAMTCETPNGDNIKCDVHDVPVDDYIPQRLRWMTEWAEWFCKAQS
QEYDKLFMQCAKCMGNGQGCTKDSSDGECEKCKEACTSYANFINTWKPQWVPMQIQYA
LLYSYVGKSGTIGLGGYPDYKQVVHFFEELQKEYENATRSSSTTKVSSTASPITPYSS
PEGYIHQELPITGCQKQKEFCYYKNGLTSRSSDAKENKNYAFKNPPHGYDLACTCNTR
DQQTDGRGRVAVNPDDNIITPGRIDNGEDDEESDEEDFEEEELENDDAGGGSDVGEEE
DVSNHQEDKGPQKEDTPQIDVCAIVKTALTTPGNLTQACKQKYGPKAPTGWKCVPSGD
STTTGEARARRVARSAEPRGSEPTSDKGSICVPPRRRKLYLGGFKRLTDGTAVSSETT
SATSSHASNGDALLTAFVESASVETFFAWHKYKMEKKQPAQEGAGLGLSLLEQEVSPE
DDPEKQLQTGTIPPDFLRLMFYTLGDYRDILDGKNMEVVNLLKDGSPSDKEMYTRENK
INQAIDNHFSKNENQATTGVNQSSSGGVPSSSGHSSSSTVTPPGPPQTSGTTPKTWWQ
KNGEHIWNGMICALTYEDNGAKGTSNALQQNEEVKKALWDETTKKPKDEKYQYDQVKL
DENSVTDGPRTTSPGTSGDTPPTLSQFISRPPYFRYLEEWGETFCRQRTRMLKNVKDN
CTKDDKQKYSGDGEECEKVLVEEANTFKDLEGRSCADSCRSYKKWIGRKKDEYDEQES
AYGQQQKEYVNGSKGAGRNNDDKEFCTKLEKTWTTAGDFLKSLGPCKNNDNGEGTIKF
NGGQTFQHTNLCDSCSEFKIKCENGVCSGDTKVECNGKTPIAATEIAKMITSTEGVFM
TVSDNSDHKFDDGLKDCQHAGIFKGIRKDVWTCGNVCGYVVCKPKEGNRETASGENKD
QIITIRALVTHWVQNFLEDYKKIKHKFLNCTKNGQSKCINGCNNKCTCVETWISTKKG
EWKNIKERFIEQYKGEPSDEYFNVRSCLETFIPQIPVADVKNEVIKLSQFDNSCGCSF
SAHKQKDSNQDSIECMIKNLEKKIDECKTQHYPSGKPEEQCKEPPPEDDYEDENEKKV
ESPTICPKETVDQKTKVEDEKCDKTRPVVPKEDRQEEEESKKKKDETALPPPEPPADS
PPPAAPTKPLPSDNTSDILKTTIPFGIALALTSIALLFLKKKTKSSVGNLFQILQIPK
SDYDIPTPKSSNRYIPYVSDTYKGKTYIYMEGDSSGEEKYAFMSDTTDVTSSESEYEE
LDINEIYPYQSPKYKTLIEVVLEPSGKNTTASGNNTTASGKNTPSDTQNDIQNDGIPS
DTPNTPSDIPKTPSDTPPPITDEEWNQLKHDFISNMLQNTQNTEPNMLGYNVDNNTHP
TPSRHTLDQKPFITSIHDRNLYTGEEYNYNVNMSTNSMDDTKYVSNNVYSGIDLINDS
LNSGNQPIDIYDELLKRKENELFGTNHPKHTNTHNVTKSSNSDPIDNQLDLFHTWLDR
HRDMCEKLKNDNERLAKLKEEWENETHSGNTHPSDSNKTLNTDVSIQIHMDNPKPINE
FTYVDSNPNQVDDTYVDSNPDNSSMDTILEDLEKYNEPYYYDMYDDDIYYDVNDHDAS
TVDSNNMDVPSKVQIEMDVNTKLVKEKYPIADVWDI
>PFL1955w, 2287 bases, 354FD5D3 checksum.
MAPSTTYSSAKNAKELLDMIGKDVYNEKVKSEAQTYKEALKGDLNTANGYNSETRGTT
DPCRLVEDYRSKAIGDANSERHPCGIGKDAKGEEVPRFSDTLGGQCTDHRIKGNNRNV
TGGACAPLRRLHVCNKNMEKMDSTKIKDKNVLLAEVCYAAKYEGKSLVEKYKEYKQPN
NDSDTDICTALARSFADIGDIVRGKDLYLGYDDEEKKQRDELEENLKTIFKNIYGNLD
KKDRYEGDTKNYYQLREDWWYANRRQVWKAITCDAKAFNYFRNTCNGESPTKGYCRCN

DDQPNADKPNTDPPTYFDYVPQYLRWFEEWAEDFCRKKNKKLNDVKKQCRGDNGTDRY
CSGNGYDCTKTIRAIGKYAIGDECTKCSFWCRMYETWIDNQKKEFLKQKRKYDKEIEK
YKNGTSNGTTTTKKTSNGPINNFYVKEFYEKLKGGYEKVDDFLELLNNEKECKGINEQ
EEKINFKTADISLNKNKNDEGTFYHSEYCKPCPHCGMKKKDGKWITKADDDKCNIKLY
KPKKGATSTEIKILKSGDEPTEIGKKLKDFCTKTQNGTGGGGGGSGGNSEKKELYDEW
KCYEFKHLDKVEEGEDDEVYEKDVKNGGGLCILKKEEEEKKEVKKTNSEKEPKEIQKT
FHDFFYFWIRRFLNDSMYWRGKVNSCINKSKREKCKEECKKECECFQKWIDKKEKEWG
QIKIHFNTQDFGSKGGLLGGVMTADFVLKTVLNIEDLFKDIKDGYGNAKELKGIENML
EKEKQKNKEEEAGASGGEDNTTIDKLLQHEGDEAGECLDTHKETCPDEGLGRTAAGYR
PPSVTKGGDEEEELSTDEEEEEDEDDTEESPQAEPEEAESEDPDGESSTAETTQQEAV
APTTQDNAEKPCDIVDKLFQNPEQFKEVACKQKYAKNNSRLGWKCVTPSGDQKATSEG
NGDANRRVARQTSESGEKSGDKDGAICIPPRRRRLYIQKLQEWAKTVGNTVVSGEPQT
QGEASSPSDKESSQSDKLREAFIQSAAIETFFLWDRYKKEWALQKLAELQRNGDLPFF
TSGAGYGMTAVVNGAQPTASPGHSNGLISLPSLVTDSDNPQNKLNDGTIPPDFLRQMF
YTLGDYRDILFGDTTMIDTLKKSGDKDMLQREQNIKEAIDKILNSGNKEPSDEQRKTW
WGDFAQYIWNGMICALTYTDSEKKDGKPQHNESVKKAFFGENNTDNPVTTSTQNGSPR
LPTGTTGNTEGTYEKTYKYDIVKLDENSGTGDPLNNPKLKEFVEIPTFFRYLHEWGQN
FCKERKKRLKQIKVDCRGDENTNRSNDGDGFNCEQIVPDKDVFWGDFIGSSCATSCSY
YRKWINTKKAEYEKQEKIYVHQKAIAKNNKNDSAFSRTLDTYTTAGDFLENLKNGPCS
KINSAEDNGNGNEEDKLNFRQPNVTFRPATDCKPCSLNELKCNSDVCGGNTKRKCDGK
TDISAEDIETMGQPTEDIGMLVSDNSTTEIKDEGLKEACKDADIFKGIRKDEWKCGKF
CDVDVCVLENFNKDIHDKKNVLIRTLFKRWLEYFLQDYIKINAKFSHCTNNGEGFACK
NKCKDKCKCVEHWINQKRTQWGKIKERFFKQYNVTDSNDYNVRSFLQDLIPRIALTND
KKHFKTLDDLENIYGCNCAWKSGNREENDLVKCLLEKLKDKIGECNRFQTSANDCTTT
PTAFEEDDDYIPLEEEEENQVVQPNICPAQTPAPTPAPAPAPAPAPAPAPAPAPAPAP
SARPNHDQNPPGTGTRKITKRRQKSDKTKKTSNPTPSNVLDHPLMKPALMSSTIMWSV
GIGFAAFTYFYLKKKTKSSVGNLFQILQIPKSDYGTPTPKSKNRYIPYVSDRYKGKTY
IYMEGDSDSGHYYEDTTDVTSSESESYEELDINDIYVPRAPKYKTLIEVVLEPSGNNTT
ASGKNTPSDTQNDIQNDGIPSSKITDNEWNQLKDDFISNMLQSEQPNDIPNDYSSGTI
PTNTNNTTPSHDNVDNNTHPTMSRLNVDQKPFITSIHDRDLYTGEEYNYNVNMSTNIM
DDIPISGKNTVYSGIDLINDSLSGNQHIDIYDEMLKRKENELFGTNHVKQTSIHSVAK
LTNSDPIHNQLELFHTWLDRHRDMCEKWENHHERLAKLKEEWENETHSGNTHPSDSNK
TLNTDVSIQIHMDNPKTKNEFKNMDTTPNKSTMDTMLDDLEKYNEPYYYDFYKDDIYY
DVNDDDKTSMDNNNNLVDKNNPVDSNNSTYNHRNPADINKNFVDKNNQNQHPIEKPTK
IQIEMNSNNREVVEQQYPIADIWNI
>PFL1960w, 2359 bases, F2654D48 checksum.
MGPPSTAPDYSSAKDAKELLDKIGQQVHDKVKSESNGFKDELEGSLSLAKVSGVELAD
TLDPCKLIKEKHENLIGARGDPCGKDGTGKEDDSKRFSKESGAECDEKKIKDNKSKGG
NNEGACAPYRRLSLCNKNMEKMGRTSTTKHDLLLDVCMAANYEAESLIPYHDQYEATY
KDYGSTMCTMLARSFADIGDIIRGRDLYSGNKVKKKKLDDSLKTIFGKIYEGLTGGVK
ERYTNDGGDFFQLREDWWTANRETVWKAMTCSEHLKNSAYFRVTCSDKQGESIANHKC
RCPMTSDGKPNDQVPTYFDYVPQYLRWFEEWAEDFCRKKKIYVGIVKKYCLDETEEKY
CSLNGCDCTQTVRAKGKLRYGNRCTDCLFACHRYEKWIDNQRKQFLKQRNKYADEIKK
YINEASSSSGSGRQRRGARNENYDGYESKFYDILKNKRYENVDAFLGLLNNEKACQAV
TDDKGGRINFKNVNSGKNSGGGESGDRGKGASSTSDTSGTNDETKGTFYRSKYCQPCP
ICGMKKKGGKWEAKNDDKCKRGKLYEPTSSAKPTEIKILKSGENHDDIEKKLEAFCET
QNRSDGSSSVHVGGGSGGGGGSGGGGGSGGGGGSGGGGGSGGGGGSGGGGGSGGGGGS
GGGGGSGGGGGSGGGGGSGGGGGSGGGGGSGGGGGSGGGGGSGGGGGSGGGGGSGSKS
DSQKLYEEWKCYKHDEVQKVQGQGEEEDEDEVEEEDDLKSAGGLCILPNPKKKEESEA
KSQNEPEQFQKTFYDFFTYWVAHMLKDSIHWRTEKLDKCINNTNGKQTKCRKGCKGDC
DCFLKWVNEKKTEWGKIKDHFKTQEGLDNEGENGIPESTRFGFTHDVVLGEFLELQFL

NENNEENSKNSLDSEEIKHLKNLRDIIKKKNQQAGAGATGKRTIMDELIDYEEGIAKD
CQQKCQETQKPQQVDRDLARSATGPRDPSSPAAPTAGPVESAAVDDEDDDNEEEEEEE
EEEDEKEEVGGSDVVEEKEEKEEEKEPEEQVEPQPAATTTQNDVKVCDTVDKLFKDTT
ALQDACNLKYVKGKNYGWRCVAPSHTTSETTGKSESATSSSGATTAPAPSLTSPQATA
SANSANSVSSAVCIPPRRRRLYVGKLEQWASGGNTQGGGGNTQVTPQAGGSEAQTQPQ
GKDAASTSPQVALLRDAFIQSAAIETFFLWHKYKMEKKKEKEETQGQVYKQTDDEDKT
PQELLNSGKIPDGFLRQMFYTLADYKDILFSGDKDKKNGYSDIINGDKEMKAKEEKIK
EAIQKFFEQTRGAIPPPPSGTTPSSWWEQHAESIWNGMICALTYTDNSDTVAKGKASI
TQDTDLKDALWDENGATNEPIKYKYTEATASGDNTPLTQFVLRPPYFRYLEEWGETFC
RQRTRMLAKIRGECVKSGSERCSGDGEDCNDQLDADPTIVRDFLCSTCGRHCSFYKKW
IQRKKEEFTEQYNAYGGQKTKCQTESETAKEFCGTLNTFNDAAKFLERLKSGPCKNNE
NVEDNGKDKLDFSKPNETFVPATNCKPCSSFKIDCKKAKCSNRDDTNVGFNCKKNGNG
YITSDHIKNEDNFTDTIDMLVSDDNKNKSETVLEACEGADIFEGIKENKWKCGNVCGY
VVCKPENGNGKIVNGKPNGENQIIIIRALFKRWLEYFLEDYNKIRKKLKPCMNNSDGS
PCIDNYKKKYQCVLQWISRKEEEWKKIKEHYEKQKPKNGDNNMKSLVTDILSGLYPQT
DVNKAIKPCKGLTKFESFCGLNRTESSKIKDGTPKDVVECLFQKIQKKIEECKQKHPQ
PSANNCTTSPTSDTPPDDEEDLLHEEEENQVKAPEICPTPQQEPEAEDEGECKAASPA
TSEEQTNQTSNPHETPVLKPEEEATAPTSPPRPPLVTSTLAWSVGIGFAAFTYFYLKK
KTKSSVGNLFQILQIPKGDYDIPTLKSTNRYIPYASDRHKGKTYIYMEGDTSGDEDKY
AFMSDTTDVTSSESEYEELDINDIYVPGSPKYKTLIEVVLEPSKRDTQNDMPSDDIPS
DIPNSDTPPPITDDEWNQLKKDFISNMLQNTQNTEPNILRDNVDNNTHPTPSRHTLDQ
KPFIMSIHDRNLLNGEEYNYDMSTNSGENNVYSGIDPTSDNRDPYSDKKDPISGTKDP
ISDNHHPYSGIDLINAALNGDYDLYDEILKRKENELFGTNHVKQTSTHSVAKNTNSDP
VMNQLDLFHKWLDRHRDMCEKWDTNNKKEELLDKLKEEWNKDNNKHNGENTINKTLNT
DVSIHIHMDDPKPTNEFSNMDTYPNNSSMDNILDDLEKYNEPYYDVQDDIYYDVNDHD
ASTVDSNNMDVPSKVKLEMSVKNTQMMEEKYPIGDVWDI
>PFL2665c, 2268 bases, 264CB658 checksum.
MGGSNGGGGSSQEQDESVKHMFDRIGQQVHDEVKNGGADAKKYVGELEGSLSQVSINL
ESAGTTDTCNLVKEYYKHPNGGGDVSDKRYPCKGLSEKYVERFSDKIGGQCTKEKISG
STNTCGACAPYRRLHLCHHNLESIQTKNYNSSNAKHDLLAEVCMAAKYEGETLTTEHG
KHQQTNNDSQICTVLARSFADIGDIVRGRDLFHGNPQESAQRKVLDEKLKEIFKEIHS
GLTKKDAQTYYDENGGNFFKLREDWWTANRHTVWEAMTCHAGDSDEYFRKTCGSGNNA
TQAKDKCRCSDNQVPTYFDYVPQYLRWFEEWAEDFCRLRKHKLKDAIDKCRGGSSNDK
YCSGNGFDCVETVRGDEHFVEKDCHDCSYSCSPFVKWIDNQKLELDKQKKKYKSEITR
GASGKSPKRTKRGARASDDNGYESKFYKILKEKNNYGTVGEFLRLLNNEKTCKDQPQV
GNEKASPVDFTKGKTKETFSRTEICEPCPWCGAEKDNTGNGKWKDKELNCGNKKHYHP
EKTTTIEILTADKKQSGIVKKYSKFCTSANGGESDENVQKVARSEKSKNGDQIVTWEC
YYDKEKGSSKNNNNCVEGTWKDFEKGKNVMPYHPFFWKWVHDMLHDSVEWKTELSKCI
NNKKGSNCIGGCKKTCECFQKWVVQKGKEWDEIKVHFNKQEDIVQPGSFIEFSPYGVL
DLVLKGGNLLQNIKDVHGDTEDIKYIKDLLNDEEAAGVLVVGSGGENKTTIDKLIEHE
KEQAEQCKKCEEAQKPQQEREDLARSLPTPEDSSSRPTQDADTGTDDIDDDDEEDEED
DVDPEEEADLGEENHQEESEDKDDANEAAVVPQPEEPQEPVPTDDVNVCETVATALTG
DDLKQACSTKYGPKAPTSWKCVPSGVSTATSGEGGDAKSRERREAGVPTATSSGNTTG
GGKDGATGGSICVPPRRRRLYVGGLTKWAKKYTGNTGESKSQEGVLQTKAVVDGKANA
EGGGQKGARGPNGGTEGANSGKGAQQQQEQQQEQQQEQQQQQQQQQQQQPHSTDSSSS
PPSSNPRDVDLRNAFVESAAIETFFLWDRYKKEKEKEKEKEDIEKNGQDTVAYTSSVEKD
PQEELQRGDIPDGFLRQMFYTLGDYRDILYSGDKENGNKYMLVDDIKDISDKIKSILN
SDVGQKTTAKQWWDDNGQHIWNGMIYALTYNTDTPSGDKPTQIDEVRAQLWDEKEKKP
KKTDNDHDYTYENVELKEDDDQSGAKTPSASSGSNDPINNPQLSDFVEIPTYFRWLHE
WGSDFCGTRKRMLGKIKHECRGDKVCSGYGENCDDQLKDNPSIFPSLNCPSCGTPCRY
YKKWINTKKTEYDKQKSAYEQQQGKCEKENNGAEGNDHDKKFCTRIQNCNEAKDFLKT

LGPCRTNDESGKGTLYFDDDTFKHADNCKPCSSFKIYCKNCKSSGGTENKCPKGKISA
DDIPSLGNSTHKLDMRVSDKNAKDFAGDLSVCADADIFTGIKEHKWKCRNVCGYVVCK
PENGNTETTSGENNDQIITIRALVTHWVHNFLEDYNRIKQKISHCKENSEQTICKKDC
KDKCKCVDEWINKKRTEWDNIKKLFIEQYKMDSDEYYYVRSCLEDFESRPELNKAIKP
CPNLNNFKTSCGLNDTESSKKKDGDKRDLVVCLLNKLEKKAEKYKDNHETSGEEKECD
SLPHVEDDDDPLEEVDQNPEEAKKNMMPTFCEIQEKKEKEEGTCEKAVDPGGDGKPKG
DQDGGTAAGPEADPEADPETEPAPAGDQKEASTSKVAPKPKPPRPQPQPPTQLLDNPH
VQTALMSSTIMWSIGIGFAAFTYFYLKKKTKHPVDLFSVINIPKSDYDIPTKLSPNRY
IPYTSGKYRGKRYIYLEGDSGTDSGYTDHYSDITSSSESEYEELDINDIYVPGSPKYK
TLIEVVLEPSGNNTTASGNNTTASGNNTTASDIPSDNTPTPQPITDDEWNTLKDEFIS
QYLQSEQPNDVPNDYTSGNSSTNTNITTMSRDNVDEKPFITSIHDRDLYTGEEYNYNI
NMSTNSMDDPKYVSNNVYSGIDLINDSLNSGNEHIDIYDELLKRKENELFGTENTKRT
STYSVAKNTNSDPILNQINLFHKWLDRHRDMCETLKNDNERLAKLKEEWENDTSTSGN
THPSDSNKTLNTDVSIQIDMDNPKPTNEFTYVDSNPNQVDDTYVDSNPDNSSMDTILD
DLEKYNEPYYDMYDDDIYYDVNDDNDISTVDTNAMDVPSKVQIEMDVNTKLVKEKYPI
SDVWDI
>PF13_0003, 3346 bases, 23006AE1 checksum.
MGNTQSSEEEEAKSPSLTESHNSARGVLEEIGKKIKDKTEKESKHVRQLKGKLSNAKF
ADRLYKESGGDLRSAYSDACSLTYKFHTNITTDGGDGRHPCHGRENNRFSESQEYGCS
NVYIKGNENNSNGTACVPPRRRHICDQNLEFLDNPHTDDTDDLLGNVLVTAKYEGNYI
VSNHPDKNSNGNKSGICTSLARSFADIGDIVRGRDMFKSNEKVEIGLKKVFEKINNGL
KKIGINDYNDISGNYYKLREAWWTANRDQVWKAITCRAPNGANYFRKGLDGKIIFSDN
GPCGRKELIVPTYLDYVPQFLRWLNEWSEEFCRIKNIKIGNIKKSCTGESNNKHCSRE
GYDCNKTNLRLNEIFMDLECPRCADDCKSYETWVEKKKKEFNKQKKKYEKEVDATQNN
DNNENGIYNKKFYDELKSSYKEVNSFFELLNKGPICEHIDKKIPMDYNNTEKTFSRSE
YCKSCPITDILCDDNECKTINEFKCREIKSMPNIRKNENETPIDIDILVNVNNKKVIT
HDLKNNYENCDLFKKLGEQKWKCKYKCYLDVCEPRNLDSNIYNERYISIKVLFKRWLE
YFLEDYNKLKEKLNPCMYNVQEIVCINECKQNCECVEKWIKEKREEWKKIKDRYVQQY
ESKDEDVSSKLKKFLKQELFTNYVKNALDKDETLDSMKESTECIDPNKPKGKPCNNND
VINILLNRLEKQIDNCKKKHEEKGEKPCVDIPKLLNDEDEDEDDDETPRAHNPCVDKN
DSQPTKTVSYIARQMHRRAKAQMTKNSVVDGDNKLEGDIFKVTFRNGGVGKNLNGDIC
KIDKTYSNDSRGTPTDGPCEGKGDRFKIGTDWQGDSFVNPQYRGIYMPPRRQHFCTSN
LEKLDVSRVIRNGNASNSLLGDVLLAAKYEAERTKNHYVSKKEEHSEACRAVRYSFAD
LGDIIRGKDMWDKNHGEKKTQENLERIFAKIKEQLLNSSIKDKYKDDDKATPKYKQLR
EDWWEANRSQVWEAMQCPPKNGTFPCKSDHTPLHDYIPQRLRWMTEWAEWYCKEQSRL
YGELVETCGKCMHKGKCKQGNGHCVTCKPACEKYKKFINTWQPQWKQMEQKYSQLYEE
AKKYNDSSRKDTTNKDDYVLQFLNKLLTQNKGNKTYDTAEGYVHQEAHISDCQKQTQF
CKKRNGEIPSSDTETDNNYAFRPQPHDHDEVCECNTRQKTKVRKKKKKVDACEMAKTL
LHNNDGTIRIGQCKRKDEGNAEYPKWDCNSQIHTTHNGACMPPRRQKLCVYFFANPSQ
IGSINKQDNLRKAFIISAAAETFRSWQYYKSKNGGENLQTQLKDGTIPDDFKRQMFYT
YGDYRDFLFGTDISKGLGEGTALEKQINILFPNGVRKIPNEKTREKWWTDHGPEIWKG
MLCALTNGLSESEKKTKIFDDYSHDKVNQSKNGNPSLEDFAKKPQFFRWFIEWSDEFC
RERKKKEEEVERDCKDEYEGCEKEKNGKCVTACKAYKEYITNKKEEYDSQKGKFDVEK
TEKKQGYEDYSEKQASEYLKEKCIKSSCNCMKKVTEISNYWTNPHKTYDTENLGIKCE
CPPSPCTIVDGILSPQNSSSYAEGCKWKYGKMSQGGTEWDCSKKSGGEGGNEDGDVVC
IPPRRRRLYVKNLQDLTGEESLVDLRKAFIKCAAIETFFAWHEFKKEKEREEKEKNEQ
DVQYKSSVLENLQKQLKNGEIDDEFKRQMFYTFADYRDICLGKDIGNDVDGINEKIDT
ILQKNGKPNNIEEYKKWWQKHGHEIWEGMLCALSYNTETKEMDKELRNKLTEQKNGNK
NTYDTVTISGGPIGNTKLEKFASRPPFFRWLEEWADEFCRKRTHKLEKIQNECKGVSG
TNQCDDDGFDCDEMCPKKDGSFETFKCLSCAKSCRFYKKWISRKKEEFDKQSKKYENE
IDDVKHNSDNIYGKDFLETLDQQYKSVELFLEKVKGPCSINNNNNEECKIDFNKPKDTF

GHAKNCGPCSEIRFKCIEDNSNWVTTNTCNKTTFKFTEDNKDTKEDSEQLGMLISDNT
VQNFADGLQNDCKDADIFKGLRKDQWSCGYFCNLDICSLKTSHGENNYKQNILIRALF
KRWLEHFLEDYNKINDKISHCMKNGEGSTCIKGCEIKCNCVSNWIKKKTLEWEIVRDR
FFKQYNVDSEKSFTVKSFLEQAPFDSDVQKAIKPFEKLRDFEDSIVCNGTTSARKEKG
TEKDVVICLLDKLQKQIETCQTKHKETSGNTCSPPPNPDTQTDTPLPLESFPPPFCNV
PPNPCGDKDATNVVGVEVLAKEMQEAAHKSMLSRSAVDSGKGDKGESSSGKSSLEGDI
SLAEFKNGFNPSGLKNVCQITEKHSYANGASKDPCNGKGNGKDQRFKIETQWKDTGKS
GKHVDVYLPPRREHICTSNLEYLLKGNSDQIMKVGNNKINHSFLGEVLLAAKYEAEFI
KTNYTRLNGQNDNGAKCRAMKYSFADIGDIVRGRDLWEHNDFKKLERDLVKIFGKIKE
GITDETTKKQYEKDDTDNKQLRCDWWEANRDQVWEAMQCKTTIPPVTTSCDTTTVTPL
VDYIPQRLRWMMEWAEWYCKYQSKAYSELRKGCEDCRSWKCMKGDSKCENCTKACKDY
NSKIEPWKQQWTKIKEKYEELYKKAQNSDTSNSGTTYPKDEKDVVSFLSKLHEKNKDN
KIYYTAAGYIHQQAKYLDCTQQTHFCDKKNGETLPSGRDNDKYAFKKPPKKYERACKC
HEKQEPPPPKVPEDSEDDRERSEPGEDALPVLPPEEIEQEEEPEETSVDTTQDEEEPA
SEGGGPSGSPTEESGEPRENSDSSDPKPDQNPEANPEQTPILKPEEEAPPKSKPPDGD
RGVGRSLGPTPRSEVEPEESENEDVEDEDDEEEEEDPDDDPEAESEEEDEDHGGQEAE
AVPPQPQAPAPLPPPPPPLPPLKTALMSSTIMWSVGIGFAAISYFLLKKKPKSPVDLI
RVIDIHKGDYGIPTLESKNRYIPYVSDTYKGKTYIYMEGDTSGDEKYGFMSDTTDITS
SESEYEELDINDIYVPGSPKYKTLIEVVLEPSKSNGNTLGDDMVPTTNTFTDEEWNEL
KHDFISQYIQSEPLNVPQYDVLKELPMNIVGNVLDDGINEKPFITSIHDRDLNSGEEI
SYNINMSTNSMDDPKYVSNNVYSGIDLINDSLSGGKPIDIYDEVLKRKENELFGTNYK
KNTSNNNVAKLTNSDPIMNQLDLLHKWLDRHRDMCEMWNNKEEVLDKLKEQWNKDNDG
GDISSDSNKRLNTDVSIEIDMDDPKGKKEFSNMDTILDNIEDDIYYDVNDENPSVNDI
PMDHNKVDVPKKVHVEMKILNNTSTGSLEQQFPISDVWNI
>PFA0015c, 1327 bases, E44FF520 checksum.
MALKKGVINESKLSARNVLENIANEIKVKRENERKYNDKLKGSLWEARFSDGLSSSFG
DIRSGYYDSCSLDHKFHTNINNGYPPARNPCDGRNQERFSNDGESKCGSDKIRGNENN
SNAGACAPFRRQNMCDKNLEYLINKNTENTHDLLGNVLVTAKYEGESIVNNHPDKDIK
GNKSSICTSLARSFADIGDIIRGKDMFKRNKHDNIEKGLREVFKKIYEGLKNNGAREH
YKEVKNGNYIKLREDWWTANRDQVWKAITCEAPENAYIIKRRIDGGDIENLILTHPKC
GHDTDPPVVDYIPQRLRWMSEWSEYFCNVLNKEIDEMNNQCKDCEMSRRCNNDTEGEK
CKKCKEQCQIFKELVSKWKNQFDKQSMKYMELYNKASTNITKQNSSAPERGYRRNHRR
RGYDDDTNVQLFLKKVIENNECKVESLGKYLDKTSHCGNYNFNYDNTPGSNRSNAFEI
TPEKFKKACKCKIPNPLEKCPNEENKNVCTRFDKVYSCTSLSFKNDLSEWNNSGVKNK
ENDNNGVLVPPRRRNLCINLFSKKDYKMKDENDFKEDLLNAAFSQGKLLGKKYSNYSN
EAYEAMKFSYADYSDIVKGTDMMNDLKKLNKELNTLLKETEKGDISVDRKTWWDDNKN
VVWNAMLCGYKTENENQQLNSSWCNVPDDDNIDQFLRWLTEWAQQYCKEKLIKAHIIN
TKCKDIVEGRKHKSMVDITDVECKRLFIDYEEWFRYRYNQWKGLSEKYIKIKKSKNSG
VNIPSEECAASYVTKHCNGCICNLRDMEDIHKNINNQNELMKEMINIIKFDTDQYRTQ
LQNISNSMEINTKSVKTAVDTTKDIVSYGLAGTMGVAAIGLQAGDFLGKKIQDLYNEF
MKPVEKKLDTSSKNLNIYEDPNIMVPAGIGVALTLGLLLFKMRTKPKHEVDMIRILQM
SQNKYGIPTTKSPNKYIPYASQRYKGKTYIYMEGDSGDEDKYIGNISSSDITSSESEY
EEMDINDIYPYTSPKYKTLIEVVLEPSKSNGNTPSKGDGNTVGDDMVPTTNTFTDEEW
SELKHDFISNMLQNQPNDVPNDYKSGDIPFNTQPNTLYFDNNQEKPFITSIHDRDLYT
GEEISYNIHMSTNSMDDPKYVSNNVYSGIDLINDTLSGNQHIDIYDEVLKRKENELFG
TNHPKNTSNNSVAKLTNSDPIMNQLDLLHKWLDRHRDMCDKWNTKEDILNKLNEQWNK
DNNVGGDIPNDNKMLNTDVSIQIDIDENKGKKDLSNMDTILDDMEDDIYYDVNDNDDD
NDQPSVYDISMDHNKVDVDVPKKVHIEMKILNNTSNGSLEQQFPISDVWNI
>gi, 3006 bases, 6298CD77 checksum.
MAPAQGGTNKTAKEVLDEIGKEVQEKATEDALTYRNDLQGNLSQAKFHGVPIDVKNPC
DLNYEIHTNVVKGRKKENPCRGREEKRFSDVLSGQCAKNTIKDSVTNSVGACAPFRRL

HVCDRNLELIKTDENTSTHDILVDVLLTAKHEGESLVKHYKEYIKKNRNFNICTVLAR
SFADIGDIVRGKDLYLGYDDEEKEKRKQLEKNLKKFFQKIHDDVMKTSGRTNGKKSAE
AQKRYNDATGNYYKLREDWWNANRDQVWKAITCDAADNDEYFENSSDGLYVFSNGQCG
RNEGKVPTNLDYVPQHLRWFDEWAEDFCRKRNITLKSAKEKCGGEGNTKYCSLNGYDC
TKVFEKKDSCSSDGNCTACSNICIAYDAWLRNQRNEFEKQKIKYTKEIEKYKSSSDKS
NSNISNKYYNEFYENFGKKEYETLQNFLKLLNKGMYCQEKIEEEEVIDFNKDEDMVFH
RSEYCQPCPDCVVQCKGGKCTEDKKNDKCRSKIIKKILQSEEPTEIHVLNSDDKQGDI
TKKLEVFCSSTTNYEGRNVQKWKCYNKNSDYNNCEMNISSYKDSTDANVMLSVECFHS
WAKNLLIDTIKWEHQLKNCINNTNVTYCESKCIKNCECYEKWIKRKEHEWEKVKNVFG
NNNRMSYIYYNNLSRVFDSFLFQVMFALDQDEKGKWDQFTEDLKKKFEPSKTNTPTGK
SQDAIEFLLDHLKDNALTCRDNNSNESCDVSKKVKTNPCGKNPSASNNLVRVKRLAEM
MQRYARKQLEKRGGEINLKGDATKGTYRQGGPADGFKNVCSINQNHTNVQNNNRAYTY
QGPCTGKDGSNGGVRMKIGTPWKPGRQIQMSAEDIYMPPRRQHMCTSNLEYLQTKDGP
LKQGDGKLVNNSFLGDVLLSAKMDAGKIIELYKKQNNKSNLTDPEDNESACRALRYSF
ADLGDIIRGRDLWDKNSDAKRLQTNLKEIFTKIKEELPEDIKKKYDKDGTDHKLLRED
WWEANRHQVWRAMKCAIENDKDMKCNGIPIEDYIPQRLRWMTEWAEWFCKEQSRLYNK
LVADCKSCKGKAKSCTQKDGDCTKCKAACDNYNKKIKPWEEQWEKIKNKYAQLYKKAL
DSVNGKEESKKKTASDAKDQQVVHFLAELIRKSGGGKGGKNVKTTVSPTTTPNTLYSS
AAGYIHHELGRTVGCNTQKEFCYSKNGKYAFKDPPKGYEEACKCNDRNPKPQPAPKKE
DEDACDVVKPLLKDKGETDDIDGCNQKYKAGKDKYPGWDCNSQIHTTHNGACMPPRRQ
KLCVSGLTKTDRIKAIEYIRTEFIKSAAIETHFAWDRYKEDNGEAEAELKNGNIPEGF
KRQMYYTFGDYRDIFFGRDISTHAYISGVSPKVITILEKENDAKYAAKQNSNNELLDD
WWDQHGKDIWEGMLCALTHKISDEEKKKEIKNKYSYKKLNESPKGSNKVEDFAKKPQF
LRWFIEWGDEFCAQREEKEAKVKVSCSDAKDYDGCKNTKSNASCVSACKVYEDYITKK
KVEYTKQKGKFDAEKITDKEGYEGFSTKDASEYLKKKCLDDTCNCMQKVKNNTEYWNT
PNKTYTNSNLEKRCECQPPQEPPPGPEGGARSDSGPRDTTPRPAGSDARSNTVPSPPP
RPAGDTVHEVAEVQEEEEEDEDGDLPEDQDEDVEVAGAEEEEDLDVGVARILRGRTNS
PDEDEDEASEEEDDDDDDAQDTTEVTGQGEEETAEDHQDTTEETVDQEKAEEDKDGGG
ETPQKETQPKVEVNPCDIVKTLFTTTETLKEACPTKYVNGREKFPNWKCISSGSDASG
SICIPPRRRKLYLHKIEGVDTTVSSDGETTTPITHDALREAFIQTAAVETFFLWHRYK
KIKEKERQEELQNGTFLLPPAQKVSPEDNPEHPQKKLKEGKIPEEFKRQMFYTLGDYR
DLCVGVKDDVAQALEASGDNKSGDKNIKDISEKIKSVIEKSGEQTPPGPKPGQTTTKP
EEWWQKNGEHIWNAMICALTHNTDTRQVDDQVKGQLFENGKNTPKNSQYQYKNVTISS
VSSNGGPIGNIKLEQFASRPTFLRWLEEWGEEFCRKQYHKLERIKEECHKDGNRNCDD
DGFECKEMCPCKDGSFETLKCPSCAKSCKSYKKWISRKKDEFTKQKGAYEKQKKDAEG
NNNDYKEFSKTLRNYNDAAAFLNSLKNGPCSKNDDDSVQDEIKFDDERKTFGHETYCK
PCSKITVKCKENNHCDNSKPNDCRNINSISAEDIEKRSNSTQDVTMSVSDSNTNGNKF
YDLNDCIKAGIFKGIREDVWKCGEYCGVDICTLEKTNNEERVSAKENDNKNQIIILIRV
LFKRWLESFLEDYNKINDKISHCMKNDKKSPCINGCQNKCNCVEKWIEKKKSEWGKVR
ERYINQYRDKNSNEAFEVKSFLETLIPQIPVVTDKGKHDSLTQLKKLLKCNCSEKSEN
SNEKDVVLCLLKKLEDKAKNCKDQASGEPCPQTTSENPDDEDILLEEENPVEAPNICP
KVEEPEPVVEEEKCDLAEAPSKESSTEENSGEGSNSEQNPRSKPEEEPPPPTSSETDT
PPPAPPTIQPSQADQPTNSISDILSSTIPFGIAIALTSIVFLFLKKKTKSSVDLLRVL
NIPKGEYGMPTLKSSNRYIPYASDRYKGKTYIYMEGDSDSGHYYEDTTDVTSSESEYE
ELDINDIYVPGSPKYKTLIEVVLEPSGNNTTASGKNTPSDTQNDIQNDGIPSSKITDN
EWNTLKDEFISQYLQSEQPNDVPNDYTSGNSSTNTNITTTSRHNVEEKPFIMSIHDRN
LYTGEEINYNVNMVNTMDDIPINRDNNVYSGIDLINDALNGDYDIYDEVLKRKENELF
GTNHVKQTSIHSVAKPARDDPIHNQLELFHKWLDRHRDMCEKLKNHHERLAKLKEEWE
NETHSGNTHPSDSNKTLNTDVSIQIHMDNPKPINEFTNMDTILDDLDINNEPYYDVQD
DIYYDVNDHDTSTVDSNAMDVPSKVQIEMDVNTKLVKEKYPIRDVWDI
>gi, 2182 bases, D3DE6998 checksum.

MEPGGSGGRGSGGSSSGKGKKDTSEYIYVSDAKDLLDRVGEKVYEEKVKNGDAKKYIE
ALKGNLNTANGRSSETASSIETCTLVKEYYERVNGDGKRHPCRKDAKNEDVNRFSDTL
GGQCTYNRIKDSQQGDNKVGACAPYRRLHLCDYNLESIDTTSTTHKLLLEVCMAAKYE
GNSINTHYTQHQRTNEDSASQLCTVLARSFADIGDIVRGKDLYLGYDNKEKEQRKKLE
QKLKDIFKKIHKDVMKTNGAQERYIDDAKGGDFFQLREDWWTSNRETVWKALICHAPK
EANYFIKTACNVGKGTNGQCHCIGGDVPTYFDYVPQYLRWFEEWAEDFCRKKKKKLEN
LQKQCRDYEQNLYCSGNGYDCTKTIYKKGKLVIGEHCTNCSVWCRMYETWIDNQKKEF
LKQKRKYETEISGGGSGKSPKRTKRAARSSSSSDDNGYESKFYKKLKEVGYQDVDKFL
KILNKEGICQKQPQVGNEKADNVDFTNEKYVKTFSRTEICEPCPWCGLEKGGPPWKVK
GDKTCGSAKTKTYDPKNITDIPVLYPDKSQQNILKKYKNFCEKGAPGGGQIKKWQCYY
DEHRPSSKNNNNCVEGTWDKFTQGKQTVKSYNVFFWDWVHDMLHDSVEWKTELSKCIN
NNTNGNTCRNNNKCKTDCGCFQKWVEKKQQEWMAIKDHFGKQTDIVQQKGLIVFSPYG
VLDLVLKGGNLLQNIKDVHGDTDDIKHIKKLLDEEDAVAVVLGGKDNTTIDKLLQHEK
EQAEQCKQKQEECEKKAQQESRGRSAETREDERTQQPADSAGEVEEEEDDDDYDEDDE
DDDVVQEEEEGKEEGTVTEVTEVTEVVEETVTEQEGVKPCDIVGKLFEDDKSLKEACG
LKYGPGGKEKFPNWKCVTPSGVSTATSGKDGAICVPPRRRRLYVGGLSQWASRGGDET
TEVSSEATSAPSQSESEKLRTAFIESAAIETFFLWHKYKEEKKPPATQDGAGLGVSLP
EPSPPGEDPQTQLQQTGVIPPDFLRQMFYTLADYKDILYSGSNDTSDTTGKQTPSSSN
DNLKNIVLEASGSTEQEKEKMKQIQAKIKKILNGATSGVPPVTKNSVKTPQQTWWENI
AKDIWNAMVCALTYKENDARGTSAKIEQNKDLKKALWDEANKNTPIEKYQYTNVKLED
ESGAKSNDTIQPPTLKNFVEIPTFFRWLHEWGNSFCFERAKRLAQIKHECMDEDGEKQ
YSGDGEYCEEIFSKQYNVLQDLSSSCAKPCRLYKTWIEKKKTEYEKQQKAYEQQKSNY
ENEQKDKCQTQSNNNANEFSRTLGASPTAAEFLQKLGSCKNDNGYENGEDNKIDFKNP
DKTFKEAHSCDPCPITGVKCQNGHCVGSANGKECKNNKITAEDIKNKTDPNGNIEMVV
SDDSTNTFEHLGDCKSSGIFKGIRKDEWKCANVCGVDICTLEKKIKNGQEGDKKYITM
KELLKRWLEYFLEDYNRIRKKIKLCTKKEDGCKCIKGCIEKWVQEKTKEWQKINDTYL
EQYKNDDGNTLTNFLEQFQYRTEFKNAIKPCDGLDQFKTSCGLNSTDNSQNGNNNDLV
LCLLNKLQKKISECKEQHSGQTQTPCDNSSLSGKESTLVEDVDDYEEQNPENKVEQPK
FCPDMKEPKKENDEEVGTCGGDEEKKKVEDSVIEQKEEEAASAPEESPPLTPEAPKKE
ENVVPKPPPPPKKRRIKTRNVLDHPAVIPALMSSTIMWSIGIGFAAFTYFYLKKKTKS
SVGNLFQILQIPKSDYDIPTLKSSNRYIPYASDRHKGKTYIYMEGDSSGDEKYAFMSD
TTDITSSESEYEELDINDIYVPGSPKYKTLIEVVLEPSKRDTQNDIHNDIPSDIPNSD
TPPPITDDEWNQLKKDFISNMLQNTQNTEPNILHDNVDNNTHPTMSRHNMDQKPFIMS
IHDRNLFSGEEYNYDMFNSGNNPINISDSTNSMDSLTSNNHSPYNDKNDLYSGIDLIN
DALSGNHIDIYDEMLKRKENELFGTQHHPKNITSNRVVTQTSSDDPITNQINLFHKWL
DRHRDMCEKWKNNHERLPKLKELWENETHSGDINSGIPSGNHVLNTDVSIQIDMDNPK
TMNEFTNMDTNPDKSTMDTILDDLEKYNEPYYYDFYKHDIYYDVNDDKASEDHINMDH
NKMDNNNSDVPTNVQIEMNVINNQELLQNEYPISHM
>gi, 3078 bases, D06F10CA checksum.
MVELAKMGPKEAAGGDDIEDESAKHMFDRIGKDVYDKVKEEAKERGKGLQGRLSEAKF
EKNESDPQTPEDPCDLDHKYHTNVTTNVINPCADRSDVRFSDEYGGQCTHNRIKDSQQ
GDNKGACAPYRRLHVCDQNLEQIEPIKITNTHNLLVDVCMAAKFEGQSITQDYPKYQA
TYGDSPSQICTMLARSFADIGDIVRGRDLYLGNPQEIKQRQQLENNLKTIFGKIYEKL
NGAEARYGNDPEFFKLREDWWTANRETVWKAITCNAWGNTYFHATCNRGERTKGYCRC
NDDQVPTYFDYVPQYLRWFEEWAEDFCRKKNKKIKDVKRNCRGKDKEDKDRYCSRNGY
DCEKTKRAIGKLRYGKQCISCLYACNPYVDWINNQKEQFDKQKKKYDEEIKKYENGAS
GGSRQKRDAGGTTTTNYDGYEKKFYDELNKSEYRTVDKFLEKLSNEEICTKVKDEEGG
TIDFKNVNSDSTSGASGTNVESQGTFYRSKYCQPCPYCGVKKVNNGGSSNEWEEKNNG
KCKSGKLYEPKPDKEGTTITILKSGKGHDDIEEKLNKFCDEKNGDTINSGGSGTGGSG
GGNSGRQELYEEWKCYKGEDVVKVGHDEDDEEDYENVKNAGGLCILKNQKKNKEEGGN
TSEKEPDEIQKTFNPFFYYWVAHMLKDSIHWKKKLQRCLQNGNRIKCGNNKCNNDCEC

FKRWITQKKDEWGKIVQHFKTQNIKGRGGSDNTAELIPFDHDYVLQYNLQEEFLKGDS
EDASEEKSENSLDAEEAEELKHLREIIESEDNNQEASVGGGVTEQKNIMDKLLNYEKD
EADLCLEIHEDEEEEKEKGDGNECIEEGENFRYNPCSGESGNKRYPVLANKVAYQMHH
KAKTQLASRAGRSALRGDISLAQFKNGRNGSTLKGQICKINENYSNDSRGNSGGPCTG
KDGDHGGVRMRIGTEWSNIEGKKQTSYKNVFLPPRREHMCTSNLENLDVGSVTKNDKA
SHSLLGDVQLAAKTDAAEIIKRYKDQNNIQLTDPIQQKDQEAMCRAVRYSFADLGDII
RGRDMWDEDKSSTDMETRLITVFKNIKEKHDGIKDNPKYTGDESKKPAYKKLRADWWE
ANRHQVWRAMKCATKGIICPGMPVDDYIPQRLRWMTEWAEWYCKAQSQEYDKLKKICA
DCMSKGDGKCTQGDVDCGKCKAACDKYKEEIEKWNEQWRKISDKYNLLYLQAKTTSTN
PGRTVLGDDDPDYQQMVDFLTPIHKASIAARVLVKRAAGSPTEIAAAAPITPYSTAAG
YIHQEIGYGGCQEQTQFCEKKHGATSTSTTKENKEYTFKQPPPEYATACDCINRSQTE
EPKKKEENVESACKIVEKILEGKNGRTTVGECNPKESYPDWDCKNNIDISHDGACMPP
RRQKLCLYYIAHESQTENIKTDDNLKDAFIKTAAAETFLSWQYYKSKNDSEAKILDRG
LIPSQFLRSMMYTFGDYRDICLNTDISKKQNDVAKAKDKIGKFFSKDGSKSPSGLSRQ
EWWKTNGPEIWKGMLCALTKYVTDTDNKRKIKNDYSYDKVNQSQNGNPSLEEFAAKPQ
FLRWMIEWGEEFCAERQKKENIIKDACNEINSTQQCNDAKHRCNQACRAYQEYVENKK
KEFSGQTNNFVLKANVQPQDPEYKGYEYKDGVQPIQGNEYLLQKCDNNKCSCMDGNVL
SVSPKEKPFGKYAHKYPEKCDCYQGKHVPSIPPPPPPVQPQPEAPTVTVDVCSIVKTL
FKDTNNFSDACGLKYGKTAPSSWKCIPSDTKSGAGATTGKSGSDSGSICIPPRRRRLY
VGKLQEWATALPQGEGAAPSHSRADDLRNAFIQSAAIETFFLWDRYKEEKKPQGDGSQ
QALSQLTSTYSDDEEDPPDKLLQNGKIPPDFLRLMFYTLGDYRDILVHGGNTSDSGNT
NGSNNNNIVLEASGNKEDMQKIQEKIEQILPKNGGTPLVPKSSAQTPDKWWNEHAESI
WKGMICALTYTEKNPDTSARGDENKIEKDDEVYEKFFGSTADKHGTASTPTGTYKTQY
DYEKVKLEDTSGAKTPSASSDTPLLSDFVLRPPYFRYLEEWGQNFCKKRKHKLAQIKH
ECKVEENGGGSRRGGITRQYSGDGEACNEMLPKNDGTVPDLEKPSCAKPCSSYRKWIE
SKGKEFEKQEKAYEQQKDKCVNGSNKHDNGFCETLTTSSKAKDFLKTLGPCKPNNVEG
KTIFDDDKTFKHTKDCDPCLKFSVNCKKDECDNSKGTDCRNKNSIDATDIENGVDSTV
LEMRVSADSKSGFNGDGLENACRGAGIFEGIRKDEWKCRNVCGYVVCKPENVNGEAKG
KHIIQIRALVKRWVEYFFEDYNKIKHKISHRIKNGEISPCIKNCVEKWVDQKRKEWKE
ITERFKDQYKNDNSDDDNVRSFLETLIPQITDANAKNKVIKLSKFGNSCGCSASANEQ
NKNGEYKDAIDCMLKKLKDKIGECEKKHHQTSDTECSDTPQPQTLEDETLDDDIETEE
AKKNMMPKICENVLKTAQQEDEGGCVPAENSEEPAATDSGKETPEQTPVLKPEEEAVP
EPPPPPPQEKAPAPIPQPQPPTPPTQLLDNPHVLTALVTSTLAWSVGIGFATFTYFYL
KKKTKSSVGNLFQILQIPKSDYDIPTKLSPNRYIPYTSGKYRGKRYIYLEGDSGTDSG
YTDHYSDITSSESEYEEMDINDIYVPGSPKYKTLIEVVLEPSGNNTTASGNNTTASGN
NTTASGKNTPSDTQNDIQNDGIPSSKITDNEWNQLKDEFISQYLQSEPNTEPNMLGYN
VDNNTHPTTSHHNVEEKPFIMSIHDRNLFSGEEYNYDMFNSGNNPINISDSTNSMDSL
TSNNHSPYNDKNDLYSGIDLINDALSGNHIDIYDEMLKRKENELFGTKHHTKHTNTYN
VAKPARDDPITNQINLFHKWLDRHRDMCEKWKNNHERLPKLKELWENETHSGDINSGI
PSGNHVLNTDVSIQIDMDNPKTKNEITNMDTNPDKSTMDTILDDLEKYNEPYYYDFYE
DDIIYHDVDVEKSSMDDIYVDHNNVTNNNMDVPTKMHIEMNIVNNKKEIFEEEYPISD
IWNI
>gi, 2664 bases, C27740EA checksum.
MVLQRAAGGGGDGIDKRSAKHLLDSIGKKVYDKVHGDALQPSNGKLKGTLSLAIFEKA
PEGKQTSEDPCDLNHEYHTTVTSGYDKENPCKDRPEVRFSYTEGAECDKSKIRGSNSN
KDGACAPFRRLHLCDQHLEHIKHDKITRHNLLADVCEAAKFEAESLEKYRGQYQLNNS
DVNINICTELARSFADIGDIVRGRDLYRGNDKEKDRLEENLRKIFKKIYDNLNDAHVQ
EHYKDDDKGTKNYYKLRNAWWEANRQTVWKAITCGAAGGTYFRQTCGTGTWTNEKCRC
PINDVPTYFDYVPQYLRWFEEWAEDFCRKKKKYVDIVKTNCRNYSRNLYCSGNGLDCQ
ETIRVIGHHVIGSECSKCSVWCRRYKKWIDNQKEEFLKQKKKCENEMLSKSKKKQSTK
YNVYEGYDKEFYKILKSENVGGLDKFLELLNEERECQEFSNDLGKIDFKTVDGGGVGA

IGGGASDSNNSNKTFSHSQYCEECPGCGVELIGNEWKEKNKGECKGGKRYNIPKGTKH
NVIPVLSFGDEHKEIIEKIEQFCAESNSDSSKLTEQWKCYYGDKEYEVCTLENRNKSE
EDPEEIQKTFHNFFYFWIRHLLNDSIEWRDKINNCIEKAKEGKCKNECKTDCGCFQRW
IGKKKEEWGEIKKHFKTQDGFSIFGNNYDFVLENVLNIDELFQDITEAYGNSQKIQGI
KDTLAKKKTQAADDATEQKNTIDLLFEYDSEEAEKCKKIQEECQPKKPTKVRNPCYGN
NTYDALAGKVAQKLQQEAKEQLDRNDSRSALKANASQGKYSNQGDPDDFKKNLCGITQ
KHSNAIGDSKNPCNNKGKERFNVGEKWKNGGEVKMSHTDLYLPPRRQHFCTSNLEHLN
TKSTGLTSDKAIHSLLGDVLLAAKKEGEDIKTKLTENDNRSSICRTMKYSFADIGDII
RGTDLWDINGDATGVQNNLKDIFSKITEELKKQHPDKFNDNDKYTNDSKHTKLRSDWW
EANRDQVWKAMTCPTKNGNIQCGATPHDDYIPQRLRWMVEWAEWFCKEQSRLYEELLR
DCGSCTTGKCNNDKCAKCDKQCQEYKTKIQPWADQWNEISNKYQILYWQAKIAAINGG
TEKSTTTKDDKDKNVIDFLQKLHEANYGTRGPPPEAHPDRRPRRAATSKSDVYETTAG
YIHQEARTRECLGQNVFCNNNGNNEYAFSLTPHEYKHACKCNENKASSPEELGRSDSF
DDHQTPRPEEDEVHSSEEGEEDESEDEEKEEEVEEVHDGADEKAGAVSQPEASPTTKD
VVKPPCDIVKELFSNVDTLQKACSTKYGPGKNYGWRCIPTKTSNDVTGEDGQGSRRVV
RSTPESGSNSDKNGATCIPPRRRRLYVGKLEQWANKHNTETSVSQGEATEARGSEAPA
PGGKESSSGGKETPSDKLRTAFIESAAVETFFLWDRYKKEWLAQKKAELQNGGLDLYS
SGDGDPDNPQNKLLNGVIPPDFLRLMFYTLGDYRDILVHGGNTSDSGNTNGSNNNNIV
LEASGNKEDMQKIQEKIEQILPKNGGTPLVPKSSAQTPDKWWNEHAESIWKGMICALT
YTEKNPDTSARGDENKIEKDDEVYEKFFGSTADKHGTASTPTGTYKTQYDYEKVKLED
TSGAKTPSASSDTPLLSDFVLRPPYFRYLEEWGQNFCKERKKRLKQIKEECMDGSDKK
YSGDGEQCDRRDTSNEVSADLEGRSCGNSCRFYKKWIKRKRKEYDKQANAYSKQKTKY
EEGSKGAGLNDHNKEFCVKLGTCTDAAAFLNRLKNGPCKKDNENGGNDINFGNTEETF
RPAENCKPCSSFKINCRNGNCRSGDGDTKEKCNGGTITTGNFNTMGTCTEDVVMHVSD
KNANEFEGDGLDEACENAGIFTGIRKDEWKCRKVCGLHICKQEKGNGAINDQQIILVR
ALLKRWVEYFLEDYKKIKKKLKPCIENGNGSTCINGCNKKCNRVGEWIKLKKDEWTKI
KNHYLEKNKEGDKNVTSLVTNVLETLVTQIAAANDKREQTSLDKLKTSLGCNCPENSR
KNDGNENDAIDCMLNKLETKIHECKTQHENSVENSDQPHPNCGGNPPPDEEDLLLEEE
NPVEQPGFCPTPQQEPEPDDKCGKLEEKKDEKKEQPEQPAEEDGGAIVPSGPPGSEPE
ADKGPVKPAEIPKPQEPPDLSHPAVIPSLVTSTLAWSVGIGFAAFTYFYLKKKTKSSV
GNLFQILQIPKSDYDIPTKLSPNRYIPYTSGKYRGKRYIYLEGDSGTDSGYTDHYSDI
TSSESEYEEMDINDIYVPGSPKYKTLIEVVLEPSGNNTTASGNNTTASGNNTTASGKN
TPSDTQNDIQNDGIPSSKITDNEWNQLKDEFISQYLQSEPNTEPNMLGYNVDNNTHPT
TSHHNVEEKPFIMSIHDRNLFSGEEYNYDMFNSGNNPINISDSTNSMDSLTSNNHSPY
NDKNDLYSGIDLINDALSGNHIDIYDEMLKRKENELFGTKHHTKHTNTYNVAKPARDD
PITNQINLFHKWLDRHRDMCEKWKNNHERLPKLKELWENETHSGDINSGIPSGNHVLN
TDVSIQIDMDNPKTKNEITNMDTNPDKSTMDTILDDLEKYNEPYYYDFYEDDIIYHDV
DVEKSSMDDIYVDHNNVTNNNMDVPTKMHIEMNIVNNKKEIFEEEYPISDIWNI
>PFC1120c, 2169 bases, 2B420EDA checksum.
MGRPKRAPAAKEPDYSSAKDAKELLDMIGQTVHSKVHREDANYRGKLYGLLTQAQFSN
KERVHINNPCLLDYNYDTNVTSNVIDPCEHKSVERFSEVSGGECDEKKIKGSNGGACA
PFRRLHVCDRNLEQIKPHTITATHNLLVDVCYAAQFEGKSISGYYPRYQTKYKDSGST
ICTVLARSFADIGDIIRGKDLYEGYDQKDKEQKVKLENKLKDIFKNIYNELTSTNGKK
GKKQALQARYQDDGSGNYYQLREDWWDANRAKVWYAITCGAGTSDKYFRKTCSNDTSD
TNEKCRCVSTDPPTYFDYVPQYLRWFEEWTEEFCRKKKKKLEDVIKKCRYDENNERKY
CSRNGFDCKDTIRAQEKLVKGYDCHKCSVACDDFEPWIKNQKQEFEKQKGKYTKEMQK
YANGTTTKETSNGPINNLYADDFYKKLQEHYPSVDEFLEKLNEQQICKDEPKVKEETI
SRVDFKNVDAFSYKEYCDTCPWCGTEKKQDGTLRYKEDVECRNQPTTPLDNTKSTDIQ
LSFTDKGNPKILEKFKNLCEENNEKTINWKCHYKEKNVYKDDSDKDYCVLQDGNQNIK
DQTIMSFESFFWSWVSRMLNDSIKWRNQHSKCINNKKETKCIEGCKKTCECFEKWVGQ
KKTEWRKIKHHFDQQENLKGRDRNITLKYYLDILFKEKIKEAYGDERESKELEEKLNN

IEGSQQAGDTEHSEFAVDVLLKHEEEIAENCIKYNPEDTCPTSDTGSRARSDHHDPPP
VIPRNDFEDEKDKQPEFKDPGRESEDDDDDEDELPPEDEAKEQEEEEKGAGEDEAASE
AEASGPKVEVEGKPPCDIVQTLFESTKNLSDACGLKYGPGGKEKFPNWKCISSGSDTG
STTKQNDSEGSEGGHRSKRHTESSDSTTTSSGSVCVPPRRRKLYVGELTKWAEEARKS
STSPQPGESGVANASASSTSSPTDATQLLRDAFIQSAAIETFFLWHKYKAENTRDNKS
PLGNGGVAQFFGSYSGSESEEKTPQQWLQSGTIPTDFLRQMFYTLGDYRDLCVGVKED
VINALKASGDNPTNKLTIQQISEKIKNVIEKSGDTPSRTPPGQQPSDNDPKSWWKLHA
PYIWNGMIYALTYDTNTASGEKKIEKDDAVYKKLWDEANNKPKKDNGQQDYTYEKVEI
KEEDSGQKASTASQTPSPRASGENKPTTLDSFVKRPTYFRYLEEWGETFCRERKKRLE
KIKVDCEVEENTGARGGTTKQKYSGDGEECSKIVENKDKIFKDLEKPSCATPCGLYKR
WIRRKKDEYNKQKSAYNEQKTKYENGNNKGGGGNGVCGTLQENAADFLKKLGPCSKTY
NENGVGKTDFDDDKTFKHTKHCDPCPKFNVNCKNCNSSGGGTKVECNGRNSGTTTITA
SDIKNGGDSTEINMLVSDKFTTKFEGDGLKACKNANIFKSIRKDVWKCVNICGVDVCG
LKKGDNNGELDDKQIILVRALIKRWLEYFLEDYNKIKKKLKSCTEKGEGSPCIKECVD
TWITEKRKEWKTIKERYVDKYTKENDGSNDLTTFLQQGPFYSLVEEAKKVVKCKDEQE
KLWGCTGNTTGHAQDKCENGDFITNLISKLQEKISECTSQSSGSDCTLSTENPSTTLD
DEEPLEEVDQNPEDAQKMIPKICGDVIPKEEAKEEGGCTPDIKKEEEKKEEKKKPEQT
AEKPAAETKAKEEKPAQDEAGPPPADPLPAREPFDPTILQTTIPFGVALALGSIAFLF
LKKKTKSPVDLFSVINIPKSDYDIPTKLSPNRYIPYTSGKYRGKRYIYLEGDSGTDSG
YTDHYSDITSSSESEYEELDINDIYAPRAPKYKTLIEVVLEPSGNNTTASGNNTTASG
NNTTASGKNTPSDTQNDIQNDDIPSSKITDNEWNTLKHDFISQYLQSEQPNDVPNDYS
SGDIPLNTQPNTLYFDKPEEKPFITSIHDRNLYTGEEYSYNINMSTNSMDDIPINSHN
NIYSGIDLINDSLNSNNVDIYDEVLKRKENELFGTNHVKHTNTHNVTKSSNSDPIDNQ
LDLFHTWLDRHRDMCEKWNNKEEVLNKLKEEWENETHSGNTHTSDSNKTLNTDVSIQI
DMDNPKPINQFTNMDTILEDLEKYNEPYYDVQDDIYYDVHDHDTSTVDSNNMDVPSKV
QIEMDVNTKLVKEKYPIGDVWDI
>chr4.glm_42, 117 bases, AAECA4F7 checksum.
MYTGDDLPYTKLREHWWEKNRGKVWEAMQCGYKHGRDPTDSEKDLAICDRKDNDYPVG
NDRKEGTEYQFFRWFSEWSEDFCQKQKKQLEELEELEKGCPTDMCTSENEKKQQMHTS
M
>PFL1970w, 2188 bases, 6948D14 checksum.
MAPGSGGAASSGEEDKDAKHVLDEFGQKVHDEVKKEAKKYIDELKAGVSFASILGEES
AHTTEPCGLDYSKLIKGSGSGGVAARGHPCGNGSASASDKRFSKERVDEYDEKKIGCS
NSEGACAPYRRLHLCNKNFPNMNSKDSSKAKHDLLVDVCMAAKYEGESLKVYHEQYEV
QYPSSGSTMCTELARSFADIGDIVRGIDLYGGNNKRRKQLDDKLKEIFKKIHENLGTH
EKKHYENDTANYYKLREAWWALNRQDVWKALTCDAHGTYFRATCSERNGGCSQANDKC
RCPKTSDGKANDQVPTYFDYVPQYLRWFEEWAEDFCRKKNKKVENVKKQCRKKDNSSD
DRYCSRNGYDCEKTKRAIGKLRYGKGCTECFFACNPYVEWIEKQKEQFDKQKKKYDEE
IKKYENGAPRSSGGRAKRAAPSNINYEGYESKFYKILKGDYGSVDAFLEKLSKENVCT
AITDGGRISFEKVNTGSTGGSVRDASGGDSGTNNEKEGTFYRSEYCQPCPYCGVKKKN
GNEWKKKDTDQCKNIKLYKPRDDKGGTPIEILKSGEKHDDIETKLKAFCKTQNGSDGG
RGGSGGSGGKNSDSSLYDPWQCYQIGELTKDNKAGGEDDEDDDYDGLVTNSGGLCIL
KNNINKKEKPERSSQKEPDEIQKTFHDFFYYWVAHMLKDSIHWRTKKLDKCLQNGNKK
CGKKICNGDCECFKRWVEQKETEWGKIKEHFKTQKGFDKEDNSSPSGFTLRMTHDVVL
DGVLKEEFLKGDSEDGSAQDTQNSLDAEEIQHLREMLKETGFDGGSGIGVAKEQKTLM
DKLIEYEKGIATKCLQKCQETQPENPARSLDSVPGSPSPPADPSLDTARGPNHEEEEE
DDDSGDDDEVDLQEDQEDQDEAEAEDPDGESSTAETTQQEAVAPTTQDNAEKPCDIVD
KLFQNPEQFKEVACKQKYAKNNSRLGWKCVTPSGDQKATSEGNGDANRRVARQTSESG
EKSGDKDGAICIPPRRRRLYIQKLQEWAKTVGNTVVSGEPQTQGEASSPSDKESSQSD
KLREAFIQSAAIETFFLWDRYKKEWALQKLAELQRNGDLPFFTSGAGYGMTAVVNGAQ
PTASPGHSNGLISLPSLVTDSDNPQNKLNDGTIPPDFLRQMFYTLGDYRDICIGVKED

VIKALKASSDKNIETIKKAIDEILSKQSRNNQQSGQKSGTTPQTWWDENAQHIWEGMV
CALTYEDNGEKKIEKVNDANGTDLFEKLKTKYQYKNVKLDENSGTEAKTNEPSSPSSD
NTPTTLTDFISRPTYFRYLEEWGQNFCKERKKRLEKIKEDCYRNGGREGPIKFDDQDE
TFRPAKNCAPCSEFKVKCNGRVCTGGVTKGNCNGGRITAENIQNKRDGNGNIHMRVID
SNTAVFNGLDEACKTSGIFKGIRKEQWKCDNVCGYEVCKPENGNENENKNQILLFNAL
FKRWLEYFLEDYKKIKHKISHCMKNGNGSKCTNDCPNKCKCVEKWVEKKREEWYIVKK
RFNGQYTKENDDAISSNLNSFLEPLVTQIAAATDKAEHETLEKLEKSLGCNCPDSSQK
KGDTPKDIVECLLNKLQQKATSCQEQHSDVDPKTSCETSSPVEDEDDTLDEETEVKAP
EICKNVVDTKKENDETGETCTSEDTITKETVETDSTDGPKQEEPPAATRPTKPKGRKP
RQPKEIDFPTPALKNAMLSSTIMWSIGIGFATFTYFFLKKKTKASVGNLFQILQIPKG
DYDISTLKSSNRYIPYASDRYKGKTYIYMEGDSSGDEKYAFMSDTTDVTSSESEYEEL
DINDIYVPGSPKYKTLIEVVLEPSKRDTQNDIHNDIPSDIPNSDTPPPITDDEWNKLK
KDFISNMLQNTQNTEPNILRDNVDNNTHPTTSHHNVEEKPFIMSIHDRNLFSGEEISY
NVNMVNSMNDIPMSGKNDVYSGIDLINDSLNSDQHIDIYDEVLKRKENELFGTNNTKK
NTSTNSASKLTNSDPITNQLELFHKWLDRHRHMCDQWNKNKKEELLDELKEEWNKENN
NSSAKTYNSDNKPSHNHVLNTDVSIQIDMDNLKPKNEFTNMDTNPDKSTMDTILDDLE
KYNEPYYYDFYKDDIYYDVNDDDKTSMDNNNNLVDKNNPVDSNNSTYNHRNPADINKN
FVDKNNQNQHPIEKPTKIQIEMNINNGELVKEKYPISDIWNI
>PFF1550w, 296 bases, CC6E1856 checksum.
MLLFNFLINVLVLPHYENYQNNHYNIRLIPNNTYRITIKSRLLAQTQIHNPHYHNDPE
LKEIIDKLNEDAIKKYQQTHDPYEQLQEVVEKNGTKYKGGNDAEPMSTLEKELLQTYE
EVFGNESDMLKSGMNTNVDEKSSTCGCTDINGVKLAKTKGRDKYLKHLKHRCTRGICF
CSVGSALLTMFGLAVAKKAAVDAILPVYVAAIKKCVSSSSLFHIFHGGSLTTALKATE
ACASVAGPDIVIPATGAAIGAFPPYGIAALVLLILAVLLIIILYIWLYRRRKNSWKHEC
KKHLCK
>MAL13P1.7, 308 bases, B14DA43F checksum.
MKMHYLKMLLFTFLINILGSPHNENYLNNHYNISIVQNNTKRTTINSRLLAQTKNHNP
HYHNDPELKEIIDKMNEEAIKKYQKMHDPYKQLKEVVEKNGTKYTGGNDAEPMSTLEK
ELLETYEETFGDKKHIMLKSGINTNQYDKSIDESSTCGCTDNNKAKLETTKGKDKYLK
HLKEGCTRSICFCSVSTVFLTLIALAFAKKAAVASLVYYGEAFQNCVSSSSLFYIFDS
VSLTTAIQTASKCASLAGATDAGGTAAGAAMGIFYPCGIAALVLLILAVVLIIILYIWL
YRRRKNSWKHECKKHLCK
>PFA0090c, 289 bases, EEB69262 checksum.
MKMYNLKMLLFTFLINTLALPHYDNYQNSHYNINLIQYRAQRTTINSRLLAQTKNHNP
HYHNDPELKEIIDKMNEEAIKKYQQTHDPYKQLKDVVEKNGTKYTGGKDAEPMSTLEK
ELLETYEEMFGNESDMLKSGMSPNVDEKSSTCECTDINGVKLAKTKGRDKYLKHLKHR
CIHGIGFCSIGSTLLTLIGLALAKKAAVDALASYATSHKICVDSINIFNMIAGSAVCA
ADLTVPAATSAATIFVPCGITALVLLILAVLLIIILYIWLYRRRKRSWKHECKKHLCK
>PFA0750w, 301 bases, 5FA740FA checksum.
MNMYYLKMILFTFLINTLVLPHYENYLNNHYNVSLIQSKTKRTTIKSRLLAQTQIRNP
HYHNDPELKEIIDKMNEEAIKKYQNTHHPYKQLKEVVEKNVTKHVGGNDTEPMSTLEK
ELLETYEEMFGNERDIMLKSGMYQNDDDGSDDSSTCECTDTNNSELAKTKGKDKYLKH
LKHRCTHGIGYCSIGSTFLTLIGLALAKKAAFDTLNVTFHGVSYSKCASSISIFNMLD
GPSMFAGGTACSADLTGNAAFAAMGALYPWGIAALVLLILAIILIIILYIWLYRRRKNS
WKHECKKHLCR
>MAL6P1.15, 231 bases, 9AE3A1AF checksum.
MFHYIYKIYIFTIILCASNLFNNNVVEIGTYKLSYHNGGIQFRMLAQKNTNKKPFGNT
LTNILCKDKKKKNLDPQISSLVSLVDNMDITQEKKDKIKNLSLKYINSRDVKEKNESI
NELQKYSNNEECKEQMDSYLMHLRMQNDIKCLKRKNLWNNIGINSITLSLIIIMIMIA
CMFAIYSTAQYIYVPASFLLIFIIYIVARYFPDMKIGFKKLKTKLNTFFQNKKQITK
>PFB0025c, 304 bases, 54C3D0F0 checksum.

MKMYYLKMLLFTFLINTLVARHYENFVNNHYNVSLIQNKTKRVTIKSRLLAQTQIHNP
HYHNDPELKEIIDKMNEEAIKKYQQTHDPYKQLKEVVEKNGSQNRSGHVAEPMSTLEK
ELLETYVETFGEESNIMLKSGRYQNGDDVSDDSSSCDCTDINNAKLEKTKGRDKYLKH
LKGRCTRGIYFCSAGSALLTLIALIAAKKAALSAVASYAGFKNCMSSIATFKLLDSST
LLSSFLSMKACVVGATDMAGTIATPAMAAFYPYGIAALVLLILAVVLIILYIWLYRRR
KHSWKHECKKHLCK
>PFB0065w, 298 bases, FC694BAB checksum.
MLLFTFLINTLVLPHYENHKNNHYNVSLIQNNTQRTTINSRLLAQTQNKNPHYHNDPE
LKEIIDKLNEEAIKKYQQTHDPYEQLKDVVEKNGTKHVGGHVSEPMSTIEKELLETYE
DVFGDKNHVMLKSGRYPNDDDKSDDSSSCECTDVNNTKLEKTKGKDKYLKHLKHRCIG
GICSCSVGSAFLTILGCAFAKSAALTAFASSESTKTCISSVAIYNLFQNSTMLSALKT
VGGTCANGAPDIAGTVSTLASAAFPPYGIAALVLLILAVALIILYIWLYRRRKNSWKH
ECKKHLCR
>PFB1020w, 297 bases, 48BE8E16 checksum.
MLLFAFLINTLVLPHYENYLNNHYNVCLIQNKTKRTTINSRLLAQTKNHNPHYHNDPE
LKEIIDKMNEEAIKKYQKSHDPYEQLKEVVEKNGTIYTGGNGAEPMSTTEKDLLETYK
EVFDDESDMLKSGMSQNVDEKSSTCECTDINGAKLTKTKGKDKYLKHLKGRCTRGICV
CSVSSVFLTLIGLITAKNAAVAAVTSSFNEASKICASSISVLHMFTHESVTLSMPSVT
AAGGVECFSDLAGTISSAAMGVFEPCGIAALVLLILAVVLIILYIWLYRRRKNSYKHE
CKKHLCK
>PFC0025c, 296 bases, 8AE79126 checksum.
MKMYYLKMLLFTFLINTLILPHYENYLNNDYNVSFIQNNTKRTTIKSRLLAQTQIHNP
HYHNDPELKEIIDKMNEEAIKKYQQTHDPYEQLKEVVEKNGTKYSGGNDAEPISTLEK
ELLETYEEIFGNESDMLKSGMSPNVDEKSSTFECTDINGVKLAKTKGRDKYLKHLKGR
CTRGIYFCSLGSVILTYIGTTAAKGAAISTAGAGSKNCIYAISIFHMLNHKSMSSALT
ALGTVGCAADYASGATSASAIFLPCGIAALVLLILTVLLIILYIWLYRRRKRSWKHEC
KKHLCK
>PFC1105w, 301 bases, E53AEAD5 checksum.
MKMYNLKMLLFTFLINTLALPHYDNYQNSHYNINLIQYRAQRTTINSRLLAQTKNHNP
HYHNDPELKEIIDKMNEEAIKKYQQTHDPYKQLKDVVEKNGTKYTGGKDAEPMSTLEK
ELLETYEEMFGNESDMLKSGMSPNVDEKSSTCECTDINGVKLAKTKGRDKYLKHLKHR
CIGGICSCSVGSFLLTMFGLHAAKVAAVAAVLSTYGKTSTCSSVLSITNMFNPATLQS
VLCATNGCTIGASDVVLPATSAAMGTFQPCGIAALVLLILAVVLIILYIWLYRRRKNS
WKHECKKHLCK
>PFD0035c, 289 bases, EC93E16B checksum.
MKMYYLKMLLFNYLINTLVLPHYENYLYNPYNVSLNPNNTQRTTIKSRLLAQTQIHNP
HYHNDPELKEIIDKMNEEAIKKYQKTHDPYEQLKDVVEKNGTKYTGGNDAKPMSTLEK
ELLETYEEMFGNESDMLKSGMSPNVDEKSSTCECTDLNGVKLAKTKGRDKYLKHLKHR
CIGGICSCTVGSAILTMIGLAATKATIIKFLTVPPLSYNASTSPVTIYNMFVGITAWG
PDAVGSIPTYSIVALQPWGIAALVLLILAVALIILYIWLYKRRKNSWKHECKKHLCK
>PF07_0130, 296 bases, 6DA97408 checksum.
MNMYYLNMLLFTFLINTLVLPHYENYLNNHYNVGLIQNNTKRATINSRLLAQTKNHNP
HYHNDPELKEIIDKMNEEAIKKYQQTHDPYKQLKDVVEKNGTKYTGGKDAEPMSTLEK
ELLETYEEMFGNESDMLKSGMSPNVDEKSSTCECTDINGVKLAKTKGRDKYLKHLKHR
CIGGICSCTLGSALLTYIGTVAAKAAIIAKFAEVPKNCITSISILHMLTYDSMSSAIK
SVGTAVCVADLAGEATTVSAIFVPCGIAALVLLILAVVLIILYIWLYRRRKNSWKHEC
KKHLCK
>PFI0045c, 293 bases, 40F75134 checksum.
MLLFTFLINTLALPHYDNYQNSHYNINLIQYRAQRTTINSRLLAQTKNHNPHYHNDPE
LKEIIDKMNEEAIKKYQQTHDPYKQLKEVVEKNGTKYTGGNDAEPMSTLEKELLETYE
EVFGNDNHMLKSGMHPNDDDISDTSSSCECTDIDNAKLAKTKGRDKYLKHLKGRCTRG

IYFCSVGSALLTTIGVCATKAALIAKFAEVPKNCIYTISILDMLTYESMSSALKALSI
QACASDLAGTAASAAMGIFYPCGIAALVLLILAVVLIILYIWLYRRRKNSWKHECKKH
LCK
>PFI0080w, 303 bases, 8F509C3 checksum.
MNTYYLKILLFSFLINILVLPQYENCEKGHYNGSLVLNNTQRTTINSRLLAQTQNHNP
HYHNDPELKEIIDKLNNEAIKKYQQTHDPYEQLREVVEKNGTKIRGEHAAEPMSTLEK
DLLDTYEEIFHEESHMLKSGRYPNDDDKTNEKSSSCECTDINNTKLTKTKGKDKYLKH
LKHRCSRGICTCSVGSLFLTLIGLAAAKKAALAYFSGTTKTCVSSIPIFNMFNCSSVA
TSLQAGGATCATGISDIAGTVSTAATTTIFPFSIAIYVLIAITVILIIILYIWLYRRRK
NSWKHECKKHLCK
>PF10_0395, 305 bases, F88CFD2B checksum.
MKMYYLKMILFNFLINTLLLPHYENSQNKHYNISLIQNNTQGTTIKSRLLAQTQNHNP
HYHNDPELKEIIDKLNDEAIKKYQKTHDPYKQLKEVVEKNGTKIRGGNSAEPMSTIEK
DLLEKYEDVFGDKNHAMLKSGRYPNDDDESDDSSSCGCTDINNAELEKTKGRDKYLKH
LKGRCTRGIYSCSVISAFLTMLGLTAAKTAAKGALAEYAAYETCLSSIPIFSLPGNST
VFSALQAGTEICATHASDLAGIISTPAMAAFEPYGIAALVLLILVVVIIILYIWLYRR
RKNSLKHQCKKHLCK
>PF11_0516, 308 bases, 5DEC1408 checksum.
MNMYYLKMLLFNILINTLVLPHYENYLNNHYNVGLIQNKTKRATIKSRLLAQTQIHNP
HYHNDPELKEIIDKMNEEAIKKYQKTHNPYKQLKEVVEKNGKKYTSGNDTEPMSTLEK
ELLETYEEVFGDEKDITLKSGINSNDDNRSDNSSTCECTDINNAKLAKTKGRDKYLKH
LKWRCTRAICFCSVGSLFLTFIGLAAARATDVAALNITFNGINYSIYATYVTILNMFN
EASMIAAIQAGVGATVDGLADMLTPAAASASAIFGGFGIAALVLLILAVALIILYIWL
YKRRKRSWKHECKKHLCK
>PFL2610w, 295 bases, FDA89D8A checksum.
MNMYYLNMLLFTFLINTLVLPHYENYLNNHYNVSLIQNKTKRATINSRLLAQTKNHNP
HYHNDPELKEIIDKLNEEAIKKYQQTHDPYEQLKDVVEKNGTKYTGGNDAEPRSTLEK
ELLETYEEMFGDECHMLKSGKYQNVDEKSSTCECTDINGGKLSKTKGRDKYLKKLKHR
CIGGICSCSVGSFLLTMFGLHAAKAAALAEFTKYGTTYSACKSSITIYSMLSSDSMIA
GSTACFTDLTVPAATSAGAIFDPCGITALVLLILAVILIIILYIWLYRRRKNSWKHECK
KHLCK
>PF14_0007, 293 bases, D4D81FE2 checksum.
MNIYHVKMLIFMFLINALVLPHYENCQNDHYNKSLLQKNTQRTTIKSRLLAQTQNHNP
HYHNDPELKEMIDKMNEEAIKKYQKTHDPYKQLKEVVEKNVTKHVSGHVAEPMSTTEK
DLLETYEEMFGHKNDIMLKSGMYPNDDNGSDKSSTCEYTDTNNTKLEKTKGKDKYLKH
LKHRCIGGICSCSVGSFLLTMSGLYFAKAAVLKYIASVGPPYNACASTINIYNMIAGG
SACVSDITGTAASAATAIFEPCGIAALVLLILAVVLIILYIWLYRRRKNSWKHECKKH
LCK
>PF14_0767, 299 bases, 614309B0 checksum.
MKMYYLKMLLFTFLINTLILPHYENYLNNDYNVSFIQNKTKRATIKSRLLAQTQIHNP
HYHNDPELKEIIDKMNEEAIKKYQETHDPYKQLKEVVEKNGTKYTGGNDAEPMSTLEK
ELLETYEEIFGNESDMLKSGMSPNVDEKSSTCECTDINNIKLGKTKGRDKYLKHLKHR
CIGGICSCTLGSALLAYIGTVAAKVAVIGSFGDTVKNCISSISILHMLTYDSMNLAMQ
SVNAAGSVTCVSDLAGATGTAYAIFVPCGITALVLLILAVVLIIIYIWLYRRRKRSWK
HECKKHLCK
>PF14_0771, 297 bases, 5ADD9117 checksum.
MLLFTFLINTLLLPHYENYLNNHYNISLIQNKTKRATIKSRLLAQTQIHNPHYHNDPE
LKEIIDKMNEEAIKKYQETHDPYKQLKEVVEKNGTKYTGGNDAEPMSTLEKELLETYE
EIFGGQKDIMLKSGMNTNDDNISNKLSTCECTDTNNMKLESRKGKDKYLKNLKHRCIG
GICSCSVGSAFLTILGFAFAKAAAMKAVADYTAFKTCVSSISTFNLLDSSTLLSSFQA
ISTCVEGATDMAGTIATPAMAAFYPWGIAALVLLILVVVLIILYIWLYRRRKNSWKHE

CKKHLCK
>PFL2635w, 295 bases, C0913FCC checksum.
MLIFNFLINILVSPHNENYLNKHYNVSLIQNNTKRTTIKSRLLAQTQIHNPHYHNDPE
LKEIIDKMNEEAIKKYQQTHDPYKQLKEVVEKNGTKYTGGNDAEPMSTLEKELLETYE
EMFGNDSDMLKSGMSPNFDEKSSTCEYIDIHGVKLAKTKGRDKYLKHLKGRCTRGIYI
SSLATVILTTIALYAAKTAALNYIVGIKGSYSACASFVTIFNMLSRESIIAVLETSAG
FCASGAGDLGGTVAANALVAIYPCGIAALVLLILTVVLIILYIWLYRRRKNSWKHECK
KHLCK
>PFD00655w, 343 bases, F13392CB checksum.
MNTYYVKLLLFTFLINILVLPHNVCTKIHHYYYAVNIKNYYIFNIYLLIFFFFENYIN
NHYNIILIQNNTQRTTIKSRLLAQTQNHNPHYHNDPELKEMIDKLNEEAIKKYQQTHE
PYEQLKELVEKSETKLTGGNDAEPMSTLEKELLEIYEEMFGHKNDIMLKSCMYQNDDE
KSDKSSSCCTDTNNAKLATTKGKDKYLKHLKHRCTRGICSCSVGSVFITLIGLLVAKA
AAVDAFKIVTASIATCSKCASSITISNMFNNASIVAALQKCGVLVATNGVSDGVGTAV
GTATTAFNPYGIAALVLILVAVALIIILYIWLYRRRKNSWKHECKKHLCK
>PFF1590w, 376 bases, 2F309D2A checksum.
MKIHYINILLFELPLNILIYNQRNHYITRTPKATTRSLSECELYAPSNYDNDPQMKEV
MDNFNRQTQQRFHEYDHRMKTTRQKCKEQCDKEIQKIILKDKLEKELMDKFATLHTDI
QNDAIPTCVCEKSVADKVEKNCMKCTQNLGGIVAPSSGVLAGIAEGALYVWRDAEIVA
AIAAAKEAGAAKGAAVGIKEGIKVLLNRLNTDFGLSPVRIKELESVINGTNYTDVTFI
YEAIYTTYKRSCVPVDVSVRFTVADTDLTFCESVWNQTLAVSQRNMGTSPLPIIQKTA
QKIVSDANFTAAATAETATEEATTTLTAKNTGEVNATYMGYQTPIIASIVAILVIVLV
MIIIYLILRYRRKKKMKKKLQYIKLLEE
>PFF1545w, 360 bases, 78C68906 checksum.
MQNYKKCKRLYYIIFLFFLFISFSFFFGIKGKNMVTAHSKNKLYMKPHTRTTTSRVLS
ECDIEKSIYDNNPDMKYVKENFDRQTSQRFEEYKERMIKNRQKCKEQCEKDIKQIILK
DKIDKPVEEKIEKVCLRCGCGLGGVAASVGVLGTAVVNVWKTAAMDAAIGDAITKGLT
EGAAMGATEGVAEVIAGLKALNIDKLFPETLGSFISATNYDKASYIFNIVNMKYKGTC
KILVPGGLSNPSNSICDSIQTWSLVQGSNHVNVTTDVAIGKEVVKVVTGAKTIAEKTA
KMATENVTKGAIKTNVAEVNATYASCQTAIIASVVAILVIVLVMVIIYLILRYRRKKK
MNKKLQYTKLLN
>PFA0065w, 192 bases, D8D323C5 checksum.
MLAQKNTNKKSNGNALKNILLKDKSKKGSKKKNPDAEISDLVNLVDNMNITQEKKDKI
KNLSLKYINSRDVKEKNESINELQKYSNNEECKEYMDSYLMHLRMQNDIKCLKRKNLW
NNIWIVSTTLLLIIIMIACVYWLASTPPPGLFCLPFIVLIFIIYIVARYFPDMKIGFK
KLKTKLNTFFQNKKQITK
>PFF1565c, 377 bases, B7EB3A8C checksum.
MKIHYTNILLFPLKLNILVNTHQKPHTTARHTQKIPTTRSLSECELYAPVNYYSDPQM
KEVMDNFNKQTQQRFHEYDERVQNTRQKCKEQCDKEIQKIILKDKIEKELNEKFSALH
TDIQSDDIPTCICEMSMADKVEKGCLRCVGVFGGGIAPSVGLLGGLGIYVWKPGALKV
AITAALNANSVKIAAAANAAGEAMGVKTVIEGLKALNVHGLCPDLFESIGTKIHYTNA
EEIAKIIVAKYRATCNLSTGTSSTQAMCKQFDYTFGMRIRLGSPVEYGPPPASAIPDT
VKKVVAGAEQAAEAKAANVRTTISSKIITEETDVINTIYMSNQTAIIASIIAIVIIVL
IMVIIYLILRYRRKKKMKKKLQYIKLLEE
>PFF1575w, 377 bases, F5B3BE9A checksum.
MKVHYINILLFALPLNILIYNQRNHKSTTHHTLKIPTTRLLCECDLYIPNYDNDPQMK
KIMENFDRQTSQRFHEYDERMKTTRQKCKDKCDKEIQKIILKDKIDKELTEKFATLQT
DIQNDAIPTCVCEKSLADKVEKTCLKCGGVLGGGVTPAWGLISGIVYTGWKAAALAAA
KKLAAEAGAAEGASQGAAAGATRLIELIQSTFQVQNIAGQSLESIFTAQTYTDVSNIT
KALFNEYAEICLPIFTDSVPVRGVRYNISSPICTFVEEGILATSRDKGGSPITFIEKK
VETMVSKAEGVATARAADVAAAKTAEFEATKVGAVEATYAGYHTTIIASIIAILIIVL

IMVIIYLILRYRRKKKMKKKLQYIKLLEE
>PFF0025w, 372 bases, 9A7E10FA checksum.
MKIHYTNILLFALPLNILVNTHKKPHTTARHTQKIPTTRSLSECELYAPVNYYSDPQM
KEVMDNFNKQTQQRFHEYDERMKTTRQKCKDKCDKEIQKIILKDKLEKELMDKFATLD
TDIQSDAIPSCVCEKSIAEKAEKGCLRCGYGLGSVAPMIGLTGSVAVNVWKTAELAAA
MELAKQAGAAAGIKAGHLAGTKVVIDQLHTLGIYFVGGKPLESIIHVTNYMNVSVIYD
KVYSHYTTLCTPRFVIDRPVGDFIFSGPVCNLVQPNHQGIWVKSSAQAIIKKKVEEAV
AEGTQAADVVAKNTADEVTKAAIKTSTEAIDAATTTYYTPIIASIVAIVLIVLIMVII
YKILRYRRKRKMKKKLQYIKLLEE
>PFF0035c, 349 bases, A3929809 checksum.
MKIHYINILLFELPLNILIYNQRNHNSTTPHHPPNTRLLCECELYAPATYDDDPQMKE
VMVKFSKQTQQRFEEYDERMKTTRQKCKDKCDKEIQKIILKDRLEKQMEQQLTTLETK
IDTNDIPTCICEKSMADKLEKECLKCAQNLGGIVAPSSGVLVGIAEGALYAWKPTAIT
AAKKAALAEATDAAIEAGMNAVSLKIEELGTVFKPSEGFVNLSSIVNKLTYNNGDALV
ESAKNVIGGLYSNGKGGNTIFYNTTIHTKSGTLYVGNFGDIGRAAHDAKLASETTALT
KAKVGAVESTYGGCQTAIIASIVAIVVIVLIMVIIYLILRYRRKKKVNKKLQYIKLLN
E
>PFA0010c, 331 bases, B68A13E0 checksum.
MKIHYINILLFELPLNILIYNQRNHKSTTPHTPNHTQTTRLLCECELYSPANNDNDAE
MKRVMQQFEDRTTQRFHEYDERMKTTRQKCKEQCDKEIQKIILKDKLEKELMDKFATL
QTDIQSDSIPTCICEKSLEDKVEKGCLRCAGVLGGGIAPGWSLVSGLGYAVWTNYVTQ
TALQKGIEAGVKAGIEGLRDFSGLGKLIPISVIQNLINHTNYDIAKTYITFVKSVNST
KCAVKEHSFCFSTYISNENALSKRAAGIAEYAADMAKITERGVLDAATPGLTTYSNAI
TASVVAIVVIVLVMIIIYLILRYRRKKKMKKKLQYIKLLEE
>PFA0020w, 372 bases, 9BA3921 checksum.
MKIHYTNILLFPLKLNILVNTHKKPSITSRHIQTTRLLCECELYTPNYDNDPEMKSVM
QQFHDRTTQRFHEYDENLKEKRQKCKDKCDKEIQKIILKDKIEKELTEKFSSLQTDIH
SDAIPTCICEKSLADKVEKNCLKCTQNLGKIVAPSSGVLAGISEAALSVWKTTEIAAA
MELAKQAGAAAGLKAGHLAGTNAVIEQLRTLGIYFVGDKLLETIIDVTNYMNVSFIYD
KVYSHYTTSCTPSLVNDQLVGTFNTSDPFCNLVHSNLQGSFYRSSAQTIIYEKVEEAV
AGAEQAATTKTAVMTPIYTTEFTAKNIAEVEAATTSYYTPIIASIVAIVIIVLIMVII
YLILRYRRKMKLKKKLQYIKLLEE
>PFA0030c, 359 bases, 22FD456E checksum.
MKLHYTKLLLFFFTLNILLTSYHAHNKNKPSITPHHTRSTTSRLLSEYDTESSIYDSD
DEIDSVKEIFERQASQRLREYDERLQEKRQKRKEQRDKNIQKIIHKDKMEKNLAEKIE
KGCLRCGCGLGSVAGSIGLFGAVAINIWKPAALDAAITAALNANAVKIAAAANAAGEV
IGKALVIAELQKMGISTLKGQTLKSFFRTISYKNVSSITKAVYGQHYEICVYDPSRNL
LSSFGDVNRHIGICNSVWKQISAVSQRGQYISHEEVIKRTVETMMSEAEVSAKAAAKT
AEAANKLAIEEAQEQVMEATIYNWYTTIGYTILAILIIVLIMIIIYLILRYRRKKKMK
KKAQYTKLLNE
>PFA0040w, 344 bases, DFBE05D6 checksum.
MKFHYINIFLFALPLNILVHNKRNHMKTIICTPKTKQTKTHRTLCECELYAPSNYEND
SEMKEVMEIFDRQTSERFREYDERMQHKRKQCKEQCEKDIQKIILKDKIEKELTEKFG
ALQTDIRTEDIPTCVCEKSVADKTEKVCLNCGKTMGAVAPAWGFVSGIGYVAWTQYVA
AKILEVGIKKSVQVSLDKVMEIVTQIHSLTTAEIPKFSAAQILSSGNFTNNISLIDMV
QYLRNNMYGVIESQKCERFGFILDTMGKNTLKVFNRNYHIQVEAVKNAVDAAEAAESL
KLATNTSILTNTIIASVIAILVIVLVMVIIYLILHYRRKKKMKKKLQYIKLLKE
>PFA0045c, 350 bases, F099FA87 checksum.
MKIHCINILLFALPLNILVNTHKKTSITPLHTPKRPTTRLLCECELYSPANYDNDPQM
KEVMENFIKQTQERFYEYDERMKTTRQKCKEQCEKDIQKIILKDKLEKELMDKFATLH
TDIQSDAIPTCVCEKSIADKMEKGCLRCAGVLGGGVMPGMGLIDGSLLGAISVLQPLE

IAAAKDAAIAKATDAAIEAGINTVVSKIKGLLASFTEKEVLVDLTKIVTPSTYNNGAI
LHKSAMALANKSCDFEGRGINSSFCNTLYNGEKTTFEPFAKAGIAEYNATYTLQKEAL
ETANVDMVKATYASYQTAIIASIVAIVVIVLIMVIIYKILRYRRKKKMKKKLQYIKLL
KE
>PFB0960c, 80 bases, 5048123B checksum.
MLAQKNANKKSLGNTLMNLLLKENKKTNLDPQTSSLVRLVDNMNITQEKKDKIKSLTL
QYINSDDILEKNKSINELKKIQ
>PFA0050c, 358 bases, A3347A0 checksum.
MKVHCYNILLFSFTLIILLLSSSQVNNQMNHYNTAHMKNTEPIKSYRSLCECELYTSM
YDDDPEMKEILHDFDRQTSQRFEEYNERLLENKQKCKEQCEKDIQKIILKDKLEKELM
DKFATLHTDIQSDAIPTCVCEKSIADKMEKECLRCAQNLGGIVAPSSGVLAGIAEGAL
IVWKPAAIKAAKAAAAKAASDAATQAGMNAVRLEIKKLLEMFTGKPGYVDLLPIVKES
TYKNGSALVDSAKKLFVESGKLEGLDRMPVFYNTVIDYPGPSNIKGFGKIGSDAYEAA
FTSQKGTLEATKVGEVNTTYGGCQTAITASVIAIVVIILIMVIIYLILRYRRKKKMKK
KLQYIKLLEE
>PFA0080c, 369 bases, 3EEC82A9 checksum.
MKDHYINILLFALPLNILVYNQRNYYITPRHTETNRSLCECELYSPTNYDSDPEMKRV
MQQFVDRTTQRFHEYDERMKTTRQKCREQCDKEIQKIILKDKIEKELAQQLTTLETNI
TTKDIPTCVCEKSLADKVEKTCLKCGGVLGGGIAPSVGILGGIGEAIMSAWKVAALKA
ATEYALTEGAAKGAIAGNAQGMEVVINFLKYWGVNEFFSEIFESILKISHYSKVKDFA
DAIITKKAQICADGLLKNPAMCRKIDIKFGLSDEYGTPIAGPPNIEIPQKISGLVEQA
DQAAVEVAKDTSQSVAAKITEEQTAVINATYTSWQIAITASVIAIVVIVLIMVIIYLI
LRYRRKKKMKKKLQYIKLLEE
>PFA0095c, 343 bases, B857FA0E checksum.
MKLHYSKILLFSLPLNILVTSSYAHNKNKPSITPHHTPITTSRVLSECDLYMPKYDND
ADMKSVKGTFDRQASQRFEEYEERIKDKRQKRKEERDKNIQEIIEKDRKDKSFAEKVE
KGCLRCGCGLGGVAASIGLFGGLGIYAWRSAALVTAIQKGIEASVEAGEAAGTKAGKE
FVIAALQKMGIFTVGDQRLVSYFSTTDYTSALKISSDIIQKYDPSSCFFDFSGARETF
CTWVKVKSDAAVNVERSTVSTNALVEKAVGTIVTDAKTVVERAVETATDEAIQRSIGV
VDAKYAICQTAIIASVVAILVIVLVMMIIYLILRYRRKKKMKKKDQYTKLLNE
>PFA0740w, 368 bases, DB5AFB4 checksum.
MKDHYINILLFALPLNILVYNQRNYYITPRHTETNRSLCECELYSPTNYDSDPEMKRV
MQQFEDRTTQRFHEYDEKMQSKRIQCKDRCDKEIQKIILKDKLDKELTEKFATLHTDI
QNDAIPTCVCEKSLADKVEKTCLKCTQNLGGIVAPSSGVLGGIGEFELSVWKPAALAA
AKEFAEKAGAAQGAIAGNAHGMKIVIYYLKDWGIQQCCPEIFNPFVAKNIYTEVANIS
GDIIAKYSAKCADTVTGGNSMCKAFCLKLGTNIAHGGRTLSVDHIVQLKIKGLVERAD
QAVAHVTKTTSETVTAAIKARETALIEGRFESSITSINASIIAIIVIVLILVIIYLIL
RYRRKKKMKKKLQYLKLLKE
>PFA0745w, 336 bases, CF2C6B28 checksum.
MKLHYTKILLFFFPLYILVYSKNKPSITPHHTQTNRSLCECDTQSTNYNNDEDIKSVK
EIFDRQTSQRFEEYEERMQEKRQKRKEQRDKNIQEIIEKDRMDKLLAEKVEKGCLRCG
CGLGGVAAGVGIFGTVAVKELTKASTVAAIAAAQEAAAAKGAVAGAEAGIKTVISGLQ
KLDISTLNGQTLVSYFDTTDYTNFKTIAHAINTQYDPSPCVLGRSGASESFCSWVRAN
FFAPQEISGKVSSTYESIEIGVTSIVSDAKKAAAAAVKKATDEVIKNSTAAAESTYAG
CQTAIIASVVAIIIIALVMIIIYLVLRYRRKKKMNKKAQYTELLNQ
>PFA0760w, 379 bases, 6A7A4EE6 checksum.
MKVHYINILLFALPLNILIYNQRNHKSTTHHTLKIPITRLLCECDIYTSIYDNDPQMK
EVMDNFNRQTQQRFHEYDERMQGKRQKCKDKCDKEIQKIILKDKLEKELMDKFATLHT
DMQSDSIPTCVCEKSVADKVEKNYMKCTQNLGGIVAPSSGVLAGIAELGLSAWKTTAL
KTAIAAAEQAGAAKGLAAGAAKGATRLIELIQSTFKIQNIAGKSLGTFIDATNYNNGP
FIYQAIYTKFEMSLCLPVFPGVDPVPGAVRDPTFCNLFEKFVPTNGSSNRDSIINAIE

TYVQPFVSDAKFTAAATAETATEEATAVLITKKTGEVTTTYASYQTAIIASIVAILVI
VLVMIIIYLILRYRRKKKMKKKLQYIKLLEE
>PFB0030c, 370 bases, 95750CA1 checksum.
MKVHYINILLFALPLNILEHNKNEPHTTPHHPPNTRLLCECELYSPANYDSDPEMKRV
MQQFVDRTTQRFHEYDERMKTTRQKCKDKCDKEIQNIILKDKLEKQMEQQLTTLETKI
DTNDIPTCVCEKSLADKTEKFCLNCGVQLGGGVLQASGLLGGIGQLGLDAWKAAALVT
AKELAEKAGAAKGLAEGNAHGMKIVIHHLKELHIDKLVPGICEKISSTGHYANITNFA
NTIIQQRGTMCGASGKNLGKDMCTKISIKLGTLKPDGIRPGLPDKDAVTKVLNGLVEQ
ADKAAAHVTKTTSESVTAAIKARETALIEGRFESSITSINASIIAIIVIVLIMVIIYL
ILRYRRKKKMKKKLQYIKLLEE
>PFB0035c, 387 bases, B17C84C checksum.
MLFLKILINVFFLFVSNEREKYDCSKKYFYNGKIIAHNKNKQYISARTPTITSRMLSE
CDINTSIYDDDTEMKFVKENFDRQTSQRFEEYNERLLENKQKCKEKCDKEIQKIILKD
KLEKELMDKFATLQTDIQNDAIPTCVCEKSLADKTEKFCLNCGVQLGGGVLQASGLLG
GIGAVAVNAWKDAALEAAIDFATEAGAAAGVAAGEAAGKAVVIKSLKYFRVDVFFPKI
FNSIGNAIPYYDAKTIGAAIAEKHAQNCALVSTNEGAMCYPFEVNLGIREAITFTQTG
PPAKYAIPDTVSEIVEGAEQAAKAAAKAAEKGVTAAIKAKETRLLEAGFNSSISSINA
SIIAIVVIILIMVIIYLILRYRRKKRMKKKHQYIKLLEE
>PFB0040c, 330 bases, 1744E1 checksum.
MKDHYINILLFALPLNILVYNQRSYYITPRHTETNRSLCECELYSPTNYDSDPEMKRV
MQQFEDRTSQRFHEYEERMQSKRMQCKEQCDKEIQKIILKDKLEKELMDKFDTLHTDI
QSDAIPTCVCEKSLADKVEKGCLRCGYGLGTVAPTVGLIGAIAVNEWTKAATAAATQK
GIEAGINVVIDTLKRLFNIEVVTDLKWKTLITAQNYTDKILVGDVIRKLGNTLCGGSE
DTAGGFCLFTVKANTLPQAINGHVTKAISEGTAEVVKVTEAEMGKVTTSAGAYSTGII
VSVVAIVVIVLIMIIIYLILRYRRKRKMTKKMQFMKLLNE
>PFB0060w, 361 bases, DC6A3F8 checksum.
MKVHYINILLFTLPLNILVTLYHVNGQGHYSSTKHPISSTKSSKYHRSLCECEIYTSI
YDNDPEMKKVMQDFDQQTSQRLREYDERLIKNRQKCKDQCDKDIQKIILKDKIEKELT
KQLEALEVDITTEDIPACVCKKSVEDKVGKNCLKCGGILGGGIPGLGVLGAYAVNSMV
QVAMDAAKKAAIAEGAEAGIAEGIKVAIQGVPKKFLLYTLNGKELQAVINANNFQNPS
FFYGEIMAEYVSWKKSDMVNSYGLFSFIEESCENNPDKIMKFILANSNDIAKDAGKAA
TKMTTQTTEALTLKKTAEATSTSAIFSNPIVISFIVLVIIVLILLIIYLILRYRRKRK
MKKKLQYLKLLKE
>MAL7P1.58, 231 bases, E657A87D checksum.
MFHYFYKVYIFTIIICASNLFNNDGVEIGTYKLSYHNGGRQFRMLAQKNENEKSNGNT
LKNTLLKDENKKGSKTKKLDPQITSLVNLVDNMDINEEQKDKIKTLTLEYINSDDIKK
KNKSINELKKYSNNEECKEHMNNYLMHLRMQNEIKYLKRKNFWNNIWIVVITLLSIIL
LIAAMAMNTFPGFSAFLVTFVLSNLMIYMFARFYPEIKVQFKKFKETCTNLFKKKSK
>PFB1005w, 316 bases, A74C5597 checksum.
MKMHYSEILFFSLSLNILITSSYAHSENKQYITPYTPNTSSRVLTECDIKMSIYDNDG
DMKSVKENFDRQTSERFEEYDERMKDKRRKCKEQCDKDIQEIIVKDKMEKSLAKKVEK
GCLRCGCGLGGVAASVGIIGPIAVNEVKKAALVAAAQKGIEVGMAKAIEELGKIVGLS
DFSYLNWSAMITATTYYKPMKLVNIVNSANSMCTDSNPAFTSLFCKASYRINSEVSSS
RFTEVISQEAAKAASAAGEAAKNAEKAQIALVNEESAHLYSAIGYSVIAILIIILLVMV
IIYLILRYRRKKKMNKKLQYTKLLNQ
>PFB1010w, 368 bases, 5AD29E74 checksum.
MKVHYMNILLFALPLNILEHNERDHNNTTLHTSITRSLCEFELYEPANYDNDQEMKEV
MQQFEVRTSQRFHEYDESLQSKRKQCKDQCDKEIQKIILKDKLEKHMAQQLSTLETRI
TTDDIPTCVCEKSMADKVEKGCLRCGCILGAAMPELGSVGGSLLYALNTWKPVALKAA
IAAANKAGMAAGIKAGDAAGMNVVIVQLGKWGINEFCPEIFESILKINHYSKLKDFAS
AIVAEHDKICAITTSGENSMCLPFDIALGLSDAKGTPIGPPASQAIPKMMNQLVGKAK

GTADFMANKVNSETYSKIITKQADLIEAGFNSCTTSIYASIIVILIIVLIMVIIYLIL
RYRRKKKMKKKLQYIKLLEE
>PFB1015w, 348 bases, A329AFE3 checksum.
MKLHYTKILLFFFPLNILLTSYHAHNKNKPYITSRHRQTSTSRVLSESDPYMLNYDND
DDMKSVKENFDRQTSQRFEEYEGRMKDKRRKCKEQCDKDIQEIILKDKMEKSLAEKVE
IGCLRCGCGLGGVAASVGIFGTVAVKELAKTATAAAVAAAQEAVKDAAMAATIKAVGA
AAGKEFVIAGLKQMGVSTLDGKELGTYITATNYTNVKNIAHAINTQYEPSSCLITVPV
DSKPICTWVRAKEGAARVIQGKQFSTQETIKVAVTSIVSDAENVAAAAEQQATKDAIK
ASTLAVDSKYAICQNAIIASVVALLIIVLIMIIIYLVLRYRRKKKMKKKAEYTKLLNQ
>PFB1040w, 345 bases, 69DAC979 checksum.
MKLHFPKILLFFFPSNILLTSYHVHSKNKPYITPRHTPTITSRVLRECDIHKSIYDND
EDMKSVKENFDRQISQRFEEYEERMKGKRQKRKEERDKNIQEIIEKDRMDKSLAEKVE
KCCLICGCGLGGVAASVGIFGGIAISELKKAAMIAAIASAQKTGVLAGEAARIPAGIK
AVIAGLKRMGISTLGGKDLGSYFATTDYTNFKTIARVINSEYQTDSCLIGGPATDKSK
TICNWVRANFVAPQDSPGKGGSVYKSIETAVKSIVTDAETVAQRAVENATEEVIKNST
AAAESTYAGCQTAIIASVVAIIIIALVMIIIYLVLRYRRKKKMKKKAEYTKLLNQ
>PFB1050w, 327 bases, 64F55E9 checksum.
MKVHYINILLFALPLNILIYNQRNHKSTTHHTLKIPITRLLCECELYAPTNYDSDPEM
KRVMQQFVDRTTQRFHEYDNRMKDKRQKCKDKCDKEIQKIILKDKLEKELMDKFATLQ
TDIQNDAIPTCVCEKSLADKVEKVCFRCGGLLGGGIAPGWGLVSGLGYVGWTNYVTQT
ALQKGIEAVISYLEQIPGIKGLPGFNLANIVNPNNYSSGGLLTTAIDAAARPICSVNH
SKTPAFCSYATQNGGSIIAKVSVDAENAANAGIDAASAEAANLAPKTLTLTNTIIVSF
VAIVVIVLVMLIIYFILHYRRKKKMKKKLQYIKLLKE
>PFC0030c, 359 bases, F6F19D39 checksum.
MKLLYSNILLFALALNILLTSYYAHNKNKPSITPHHTRTTTSRLLSECDTESSIYDSD
EEINSVKEIFERQTSRRFEEYEERMITQRQKYKEQRDKDIQKIIHKDKMEKNLAEKIE
KGCLRCGCGLGSVAGSIGLFGAVAINIWKPAALDAAIAKAITEGTANISAAGVKAGEA
TGKVLVISGLKKMGISTLGGKNLESYFATTSYKKVASIAQAVYEQHFEKCEFGYLKEG
FTPVGDPSRDIHFCQSVWQQTSVVSKTGHYISPKDVIKRTVETMVSKAEGPANAAAEF
VKATETATIKAAEEKTIETASTQLYSAIGYSILAILIIVLIMLIIYLILRYRRKKKMK
KKAQYTKLLNE
>PFC0035w, 374 bases, A00E6632 checksum.
MRITIKMKVHYINILLLLALSLNILANTHQKPSSTPRHIQTTRLLCECELYMHNYDNDP
EMKRVMQQFHDRTTQRFQEYDERLQEKRQVCKDTCDKEIEKIILKDKIEKELNEKFAT
LQTDIHSDAIPTCICKTSMADKVEKTCLKCTQNLGGIVAPSSGVLAGIAEGALYAWKP
NALQTAIEAALKAAADDILVGGIEAGKKVVIGGLDALEIEKLGIGSWEPYFTEGYCIN
VKSLASIIYEKRQTLCGATKSTLDKATCEQIGISIGTMQHDGTYGAPGTTPIETVLNG
IVEGTKEAADVAAEAARESATNAIKVQETRLLEAGFNSSISSINASIIAIVVIILIMV
IIYFILRYRRKKKLKKKLQYIKLLEE
>PFC1100w, 317 bases, AF4895F6 checksum.
MKLHYTKILLFFFPLYILVTSYHVYSKNKPSITSHHTPITTSRVLSEKDLQSSIYDND
ADMKSVKENFDRRTSQRFEEYEERVKDKRQKCKEQRDKDIQKIVLKDKMEKNLAEKVE
IGCLRCGCALGGGVAPIWGLVSGLWYATWSQYVSAKVLEEATNAAIKAGIIKAIEGLK
ELQDLGKLVGDKWSNIVTPETYSNPNLLSRALQRVNIAMCYSESNENVLFCNAIQRRP
ESVFVPASKVAADAGEAGKAVYLATEKAELLKIAPATNALTTAIIASVVAIIIIALVM
IIIYLVLRYRRKKKMKKKLQYIKLLNE
>PFD0015c, 370 bases, BB7AC3A7 checksum.
MKIHYINILLFELPLNILIYNQRNHKSTNLHTQNNRSLCECELYAPATYDDDPQMKEV
MVKFSKQTQQRFHEYDERMVEKRMQCKDKCDKEIQKIILKDKMEKELMDKFATLQTDI
QSDAIPTCVCEKSLADKVEKGCLRCGYGLGTVAPTVGLIGSVAVHVWKPKALEAAIAK
AIAEGTADIAAAAEAAGKARGMEFVIKALKHFGVENFFPGICDTISSTGNYTKVTEFV

NTIYSKYNGTCNLMRSSINPTACYTIETELSIKTGGAGTGDHPPLYAIRQMIKGLAEE
ATEAAKAAEAAKNAKLTAAIKEKQTALIEAGFNSSITSINASIIAILIIVLIMVIIYL
ILRYRRKKKMKKKLQYIKLLKE
>PF14_0761, 820 bases, EC7D59F8 checksum.
MGIILNIYIFFIYLIYVRSHFSQPTSSEYEGYSEICEKARNANESSVYCMKDYKRNSS
KYMYKHIMRMFIEKHNLNDNKIALIEHECGEPQNYMTYDTFLRKILSFNNSLNKYDGI
NIPEKIYNEEMNNGKFKLLGLYGSNSINWLVADLGAMLSGVTTLVMHSKFSMDVIVGI
LNETKLEWLCLDLDLVEGLMERINELPHLKNLIILDTVAKQGIINSTIEKNKNINLKG
NGNLNNMNNKKGNDLSKNLEDVRLGPIKYDKEKLEKFKSLKERYHKYLEKFLLLDDVI
NNENTNFKIKNEDPNFVTSIVYTSGSSGMPKGAMLSNKNLYNQLYSLYNHSVRKTYNL
QYHLSYLPISHVFERTIAYSIIFLRGTVHIWSKNLNYFSKDILNSNSVIMTGVPKVFS
RIYTNIMTEINNLSPFKRCIIKKIISLKKSNKKRWLVNFLENLFHISSKIKEKVNPNL
EIILNGGGKLSPDVASELCNLLNIKYCQGYGLTETGGAIFGKHVEDLNFECIGGPICP
NTKYKVRSWETYKATDTLPKGELLIKSDSIFRGYFLEKEYTKNAFTNDGYFKTGDVVQ
INKNGTLTFLDRSKGLVKLSQGEYIETDLLNNLYSQISFINNCVVYGDDSMDGPLAII
SVDKYLLFLSLKDDNMLEMTGVNEQNYLDKLTDDNINNNIFLDYVKEKMLEVYKETNL
NRYNIINNIYLTSKVWDTNNYLTPTFKVKRFYVFKDYAFFISQVKEIYNNKLKGCAPI
SVNSENKDEEKKNDSKKKEEKDSEKLSNESTSNQNKNDMGKHEKYVENKKVKLGATYG
TRQKEMNK
>PFD0025w, 382 bases, 9FE01EB5 checksum.
MKIHYTNILLFPLKLNILVNTHKKPSITPRHIQTNRLLCECQLYAPQNYDNDPEMKRV
MQQFHDRTTQRFQEYDERLQEKRQVCKDTCDKEIQKIILKDKLEKELMDKFATLQTDI
QSDAIPTCICEKSLADKVEKGCLRCAQNLGGIVAPSSGVLAGIAEGALYAWKPKALEA
SIKTAIAEGTANILAAGVEAGEVTGKELVIAGLKKMGISTLDNKSLESYFATTSYKNI
TNIAQAVQKLYFETCACDSSGKVVYLYGDANRHIPICNSVWNQTPAVLTTKKGISTKE
VIEKTVQTMVSDAEGVAADASAAKSAELTAAIKAREAEVINTIFMSKQTAIIASVVAI
LIIVLIMLIIYLILRYRRKKKMKKKLQYIKLLNQ
>PFD0030c, 380 bases, 2856DA57 checksum.
MKIHYINILLFELPLNILIYNQRNHKSTTHHTLKIPTTRLLCECELYSPANYDNDPEM
KEVMEIFDRQTSERFHEYDERMVEKRMQCKDKCDKEIQKIILKDKLEKELAEKFVTLQ
TDIQNDAIPTCVCEKSIADKVEKGCLRCVGVFGGGVMPGFGTIGGTALYALNQLKPAV
FKAAIKAALEEGAAEILAAGIEAGDAAGMNVVRYGLRYLHVHELFPVIFDSFVKTRPY
NEITSIANSILLKYGPTCTGLDNNSPPAACTKFQLNLGIHKKIGAMIDTHGTPASTAI
RQGLEGILEEATQTAEAAAKIAEKGVAAEITARETALIEAGFNSSITSINASIFAIVV
IVLIMVIIYLILRYRRKKKMKKKLQYIKLLEE
>PFD0040c, 329 bases, B7ECC245 checksum.
MKIHCINILLFSVPLNILVNTHKKSSIRRLHTQTTRSLCECELYSPANYDSDPQMKEV
MENFIKQTQQRFHDYDDRMKEKRKQCKERCDKEIQKIILKDKLEKELMDKFATLHTDI
QSDSIPTCVCEKSLADKTEKFCLNCGKTMGGVAPGWGLVGGLWYATWSQYVTKTAIQK
GIEAGVKWGIQELKVFHSLYRLIEVSQIQSFINPANYAEKTTYFSFVKSVNSTKCVGK
AVNTEPFCNFVSLNGESALSDRAAVIAKDAAYMAEVAKEGVLKEGASVTSSLTTGITA
SLIAIVVIVLIMIIIYLVLRYRRKKKMKKKLQYIKLLEE
>PFD0045c, 309 bases, 912265AF checksum.
MKLHYTKILLFFFPLNILLTSYHEHNKNKPYITSHTPTTTSRVLSECDLYMPNYDKDA
DMKSVKENFDRQTSQRFEEYEERMKDKRQKHKEERDKNIQEIILKDKIERSLEEKVEK
CCLICGCGLGGGVAPFVGLFGGLAVNELKKDAAVAATEAGIKKAIEGVGSIFYLEEGS
PIPWMNKIHAGNYNQKMPLVTIVNSIYNNCEDDPSSAGSLFCQASKSFIEERQRPVFT
KTISEMAGNAAEAAGKAANEKYAEMTSVGTICSDPIVISAIVVVTIAVILLIIYLILR
YRRKKKMNKKLQYTKLLNQ
>PFD0050w, 367 bases, C055BE31 checksum.
MKIHYTNILLFPLKLNILVNTHKKPSISPHHIQTTRLLCECELYMSNYDNDPEMKSVM

QQFHDCTTQRFQEYDERLQEKRQICKDKCDKEIQKIILKDKIEKELTEKFSSLHTDIQ
GDDIPTCICEKSLADKVEKGCLRCAQNLGGIVVPSSGVLGEIAAFAVNAWKTTEIAAA
TELAKQAGAAAGKIAGEAMGVKTIIRIFEKIFSIDKLKGTTLKSFFTTTRYNDVKTIA
SVIDTEMNTSCVLNSFGNHAICGLRTKLDLVAKPGQVMVIQKDAITRMITDVVHKAEI
TAEAAKTQVAASKTAAAIKTNIEAIEAATTPYYTPIIASIVAVVVIILIMVIIYLILR
YRRKKKMKKKLQYIKLLEV
>PFD0055w, 375 bases, DADBDCAE checksum.
MKVHYINILLFALPLYILDHKQRNHNSTTYHASKTKSIKTHRSLCECELYAPSDYDND
AEMIRVMQQFHDRTTQRFQEYDERLQENRQIYKDTCDKEIQKIILKDKIEKELTEKFS
ALHTDIQSDGIPTCICEKSLADKVEKVCLRCGSILGAAMPEVGSIGGGLLYALNAWKT
DAIAAATKAAMIEGAAQGAVAGEAAGRNAIIGALKRYFHIDNLNGTSLKSFFNSTSYS
DVTTIASAIDTQMTASCDAFSGKIVNQAFCDVRKTLRIVADPGKSFVKQKDAITGAVT
QLVEKAKDTASFKATEVSSATSSKIITKQNALIEAGFDSSTTSIYASIIVILIIVLIM
VIIYLILRYRRKKKMKKKLQYIKLLEE
>PFD0060w, 329 bases, 768BE855 checksum.
MKVHYINILLFALPLNILVINQRNHNNSTYHTSNTKLTKTHRTLCECELYAPSNYEND
PEMKELMENFNHQSSERFREYDERIQDKRKQFKEQCEKDIQKIILKDKIEKELTEKLS
TLQTDISTNDIPTCVCEKSLADKMEKTCLKCGGVLGTAVPELGLIGGSVIYSAAQAAA
AKLGVAKAIELMKKIYNLGNVSFIDWTNLINVGNYSHRMSLVGIVNKVNNMCQIKDPE
GNVVFCFAKQNMRGGAGKFAQTISEQAGNAAIKAGETANVKFAEMTSVGTIFSDPIVI
SATVVVTIAVILIIIYLILRYRRKKKMKKKLQYIKLLEE
>PFD0070c, 347 bases, 17A82B13 checksum.
MLLCLFLLNKLLLLLLLLPLLLSCKNFQSNGYISPHTRSVTKITTSRTLSEFDKYKNN
YDDDPEMKELMKRFNERTAVRLKEYDEQKKKKQKIYKEKKDKDIKEIIVKDKIQKQLT
KQLSKLEKVTDTDDLFKGKNEKKVATKGKKGRKKNKKTLGQTLSEWNILPNIDMYEWI
PFSSKEAKVDCNIKNIKQALTRVGYIGPNKFVTLGSKDGKDAIDMDNLFSSIMSSSNA
LYQKYIDKDDTSNGSSFKKQSGFLSFALYALWEIFEHIVLPVVTSMLLNGNDSESQVS
GVDGHGHEVAHGVSVLYESFIALYTIASILLILYYILKYYRKIRMEKKEKYMKILQD
>PFD0640c, 376 bases, EE86FAAE checksum.
MKVHYISILLFSHPLNILLTSSQVYNQKNPYLTPHKTNPKSVKTCRSLCECELYAPSN
YDNDPEMKKVKQQFDDRTSQRSHEYHKRIQENRQKCKEQCEKDIQKIILKDKIEKELT
EKLGALQTEIRSDAIPTCTCQKSVGYKVEKTCLKCGGILGVGVAPSLGLLGEIGGLVI
NNWKNTPFYEAFVAFAQKEGIAAGKIASDAARIDTVISGIISNFEVHTINGSTLANAI
TLETLKDDTILTKALHFEYGSMCVNTPTDDKLICAYGMRAGLVQGKSASPEAVIRSSV
KTLLKNADNVASQAAQTTANETTSGMIKAELSKIASAGANTYSAITYSVTAILVIVLV
MVIIYLILRYRRKKKMKKKLQYIKLLKE
>PFD0645w, 346 bases, BE191C17 checksum.
MKLHYFKILLLFAHPLNILLLSSSHVHNKKNPYIITSHTPSQESLKTCRSLCECDLYT
SIYDNDPEMQKVIQEFDNRTSQRFEEYDRRMVKNRQKCKERCDKEIEQIILKDKIEKE
LTEKLGALQTEIQSDAVPTCTCQKSVADKVEKTCLKCGSVLGGGIAPGWGLVSGIGYS
TWTHYVATTVAKAATDAGIKEATEALGRIYTLNEVTVINWTSKITATNYYKPMELVEI
VNGVNNMCGETGVAGNTSFCLAAESIKPSDVFTRIISRQAQEAATYAASKAKDVTTAE
FANSASSTATLTNTIIASVVAILVIVLVMIIIYLILRYRRKTKMKKKLQYIKLLNE
>PF10_0323, 355 bases, 5404EC34 checksum.
MKVGKIFFLLNILVVCHFIISCLCRNGQTTRGNLLALKAIEQDLQQKKNRKRNLILYS
LGSAALIAALVVTGIGLNMYMKKKNVDSEVQEIIDEKDEKVKEKPAEKKKTTVKIVSK
RVPVKSKSSNGKSKARTVNSEVSPKLDDEKKEDLLKFNDNDLLLAAESLKELNPKYDE
NTQGNDSFKNINEPRKLASFSLYDALADASEQNKNKDAESSTGQIPTPTESSHGISDG
KKDTSTNDMDPLNPYGSSKRNSSEDKPTSESKGTTPESNFDSKTPEIKEINEPIIVPS
YYPTTGPNPNTHGPPSRRISTSRSSGSSNRSSSGTSTRSKGPSSPLRDSSGRSSGRST
TPRVRKE

>PFD1010w, 346 bases, 523A1C6 checksum.
MKLHYTKILLFFFPLYILVTSYHVYSKNKPSITSHHTQTNRSLCECDTQSSNYDKDTG
MKSVMQQFVDRTSQCFEEYDERMNEKRQKSKEQRDKNVQKIIEKDKIEKSLAEKVETG
CLKYGCGLGGVAGSVGIFGTVAVKELTKAAITTATELAKEAAKDGAMAATIKAAGAEA
GKKFVIAGLGKLHVSTLDNQILESYFATTKYTNVTKIARAINEQYNPSSCLTGGSGAD
NSICPWAMENFFAARKIPGKVSSTYNSIEVAVKSIVSDAEPVATAAAQQATEEAIKNS
IAVVDAKYVICQNAIIASVVALLIIVLIMIIIYLVLRYRRKNKMNKKAHYTKLLKE
>PFD1020c, 261 bases, 82EAD003 checksum.
MKVHYINILLFTVPLNILVTLCHVYNQISLHIRNHTPTTKSRLLCECDVYTSIYDNDP
EMKNVMENFNKQTEERFHEYNERMQEKRKECKDQCEKDIQKIILKDKIEKELTEKLEA
LETNIKTEDIPTCVCEKSVADKVEKACLRCGGILGGGLEPTVGLLGTVVVNQLTKTAT
VASIEFVTQEGIKAGIKAVVHNLINVLHLFDVTRDIWLTLINSKNYNTVSGLTTAAKT
AKEAVGTTCLRNTPRLKPSCDAIFNKSKV
>PFD1230c, 324 bases, 18130BCD checksum.
MKIHYTNILLFPLKLNILVNTHKKPHTTARHIQTNRLLCECELYMSNYDNDPEMKSVM
QQFHDRTKQRFQEYDEMLQEKRQICKDTCDKEIQKIILKDKIEKELNEKFSALHTDIQ
SDAIPTCICEKSVTDKFEKTCLKCSGIFATAVPELGLIGGTVVYDAAVKAATKAAIKK
AIEEVGKIFFLADESTMQWTNMINAGNYSDKMSLVSILTSLNNGCQDGEAVAGPLFCT
ASNALTESGNPYVFTRTISEKAANAADAARKAASGKFAEMTSVGTIFSDPIVISAIVV
ISIAVILLIIYLILRYRRKIKMNKKLQYIKLLKE
>PFD1240w, 324 bases, 1702F03A checksum.
MKVHYINILLFELPLNILIYNQRNHYITRTPKTNTRTLCECELYAPSNYDNDQEMKEV
MQQFEVRTSQRFHEYDESLQSKRMQCKDKCDKEIQKIILKDKLEKELMDKFATLQTDI
QNDAIPTCVCEKSLADKVEKTCLKCGGLLGGGVAPGWGLISGLGYVGWTNYVTQTALQ
KGIEAVISYLEQIPGITDLPGFNLANIINPKNYSSSSLIIQAIDAAAGPICDVPVNES
LKFCVLSSYNESTIIKQVSGGAESAAAFGEETASAEVAKFVPKTTILTNTITTSFIVI
VVIVLIMVIIYLILRYRRKKKMKKKLQYIKLLEE
>PFE1630w, 318 bases, EBEF7208 checksum.
MKVHCYNILLFSLLLHRLLLSSSKVNIEMNHHNTPHIKNREPTKSYRSLCECELYTYI
YDDDPDMKEIMNDFDRQTSQRFEEYNERVNKNRQKCKEQCDRDIQNIILKDKVEKSLS
EKVEKGCLRCGCGLGGVAAGVGIFGALGTYGWKVAATATAIEFATQEGIKAGIQAAIE
QIKITVFNSLLNVEWLNFINASNYNSIAGLVEAVKAAVVSTERTSELSSNTMDRVRNA
LSEAENWFSPAVREGTQTTASTITTVQRTQLVDVTATSTYSYMAIAYSVIAILIIVLV
MIIIYLILRYRRKKKMNKKAQYTKLLNQ
>PFF1555w, 370 bases, 70CBC0B0 checksum.
MKIHDINILLFALPLNILVGSPQKNPSIITCHTPTNRSLCECDLYMPDYDNYPQMKKV
IQQFEDRTSQRFHEYDDRMVEKRKQYKDKCDKEIQKIILKDKLEKQMAEQLTTLQTDI
QSDALPTCICERSIADKVEKNCMKCTQNLGGIVAPSSGVLAGIAEGALYAWKPTALKV
AIDKAIAAGAAMGKAAGDAAGATEVIKLMKTTFYINELNGKLLESVFTAQNYTNFPNL
PHVIYKQYKTTCDLFAINTSSDPICKISKTFNFIAESGQAPVSEEAVIEAKVTEIFTK
ATDVAATETTNVTTTQTTILETAKKGAIETTCMGYHTTIIVSIIAILVIVLVMVIIYL
ILRYRRKKKMKKKLHYIKLLKE
>PF07_0003, 331 bases, AC58A1A5 checksum.
MKFHYFNILLLNIPLNILIFSSHVNDQKNAYNSSIHHAPKTKTTKIPNIRLLCECELY
AAPNYDNSEMKVVMQNFDRQTEQRFNEYEERMLKNRKKCKEQCDKDIQKIILKDKIEK
ELEEKLAALETNINTNDIPTCICKKSVADKVEKTCLKCGGILGTTVPQLGILGGISTH
MLTTAATSAAIDAGMKAVVDKLKDFVIGFRGNTVDLTPIVNPSSYNCGNLLLENAKNL
TKTTCMPNSAGKVSTLCAQIQNSGSISTFNGFAEAGTDAYNTTWATKTAEINSFNIAM
MASIIAIVVIVLVMVIVYLILRYRRKEIIKKKLQYIKLLNQ
>MAL7P1.57, 346 bases, B8831DE9 checksum.
MMFHYFNILLCSLPLNILLTPSYEINMKSHNRSTLHTLNAKPIKIHRSLCECDLYTST

YDNDPEMKEVMQQFDDRAAQRFKEYEEMLQYKRKQCKKQCDKDIQKIILKDKIEKELT
ENFSALQTDISTNDIPTCICEKSVADKVEKTCLKCGGILGGGIAPGWSLVSGLGYMAW
TRYIAAKVLEEGIKKGLEVGLVKVTEIATQMIGDVNKVPSIDILQKITIGNFSDGVSL
YDIFKTIDSTMSTELETQGYYDFSLSVQSIADDPAKLKWCSQKVADVTNAVADGEASV
LTEAAPVTSGLNTAIIASIVAIVVIILVMSIIYLILRYRRKKKIHKKLQYIKLLQQ
>PF07_0132, 366 bases, 91AD532D checksum.
MKDHYINILLFALPLNILVYNQRNYYITPRHTETNRSLCECELYSPTNYDSDPEMKRV
MQQFVDRTTQRFHEYDERMKTTRQKCKDKCHKEIEKIILKDKMEKQMAQQLTTLETKI
GTDDIPTCVCEKSMADKMEKDCLRCTYGLGTLAPTVGLIGSVAVGAWKPTALKAAIVA
AQKAGDAAGVAAGEAAGKKAVILALQHFKLDNLFPEIYNAIVKIRHYADVKNFSVAIV
EEHSLKCQSLDLKVTTNPTCETFEFNIGMRIPDSSFVEPVDQVVPEVLDSLVGNIKEV
AEAKAAEVAAAKTAEFKIANVGAVESTYGSCQTAIIASIVAIVVIVLIMVIIYLILRY
RRKKKMKKKLQYIKLLKE
>PF07_0134, 382 bases, 5FE87B17 checksum.
MKLTYLNILSCYIILNILLLSSQINIQWNLYNTTQIQKTKLTITNTRLLCECDLYTPS
NYDDDQEMKELMQQFEDRTSQRFHKYEERMKTTRQKCREQCDKDIKKIILNYKIEKEL
SQKLVTLETNIDTTDISTCVCEKSLSDKLEKTCLRCGGVFGGGVMPGFGAIGGTALYA
LNQLKPTAIATAIEEAIKAGASNISAAAFKASEAAGLAKVITELKALSVEKLIPGILN
SIFSETHYTNATKIAEIILTKKSVTCGLSSPGNPICHEFGINFNIIDPGNGFFFPDKT
GITQKVTEVVEGAKGAAANASKVASERVTAAIKAHETNVINATYAGYEITIIASIIAI
LIIVLIMVIIYLILRYQRKKKMKKKLQYIKLLKE
>PF10_0023, 276 bases, AF946D51 checksum.
MIKDHNNNEMSMNFYTYKKMINDNSTSYSSSKMEPESNRYIKGKNINIFPLLKFTVFT
LLIWIVTFINNYGNYMNDGCRMTDLSTSRFLSEPLLEFDTVFDVFKDTFLKNMGCSDE
ETKNIRSTMKQYFDNIDMNALSKEMKENGNFFEQIGNNENTLERFMKKDISQLKCINV
ENAEEGEEIPKPYTVEEVDPNNPNKGEIGNMKFNSNFIRRSVITLKKIGTSPTFLITM
TAILLKNDNFKTALLLMVALFLKGVNFALDLKAYNDKFHFIKFK
>PFB0120w, 106 bases, C401A844 checksum.
MKLSKILYFFAALLALNFIAPRDYNSMVEAKPAKKLTPAERKKRNQNIMIYSSIASAV
ALLIGGAVGLGIHLHKNNKGDNKKGTPGAKKNDNKAVNPSISSTMYRA
>PF07_0138, 339 bases, FF0904D0 checksum.
MKIHYINILLFELPLNILIYNQRNHNSTTHHTLKIPITRLLCECELYAPSNYDNDPEM
KEVMEIFDRQTSERFHEYDERMKTTRQKCREQCDKEIEKIILKDKLEKELMDKFATLH
TDMQSDAIPTCVCEKSVADKTEKVCLNCGKTMGAVAPAWGLISGLWYATWSQYVSAKI
LEVGISEGIKEGLTQIMKFTISLYPKANLPNITVTQMLSSGKFTNNVTLFDMVQHINN
TMYTTLEAEEYSKFCGVVSSMAKYKNITFNRTYGKYSTAVTEAVTQGKTNAINTLTPA
TNTLTTAIIASMVAIVVIVLVMIIIYLILRYRRKKKMKKKLQYIKLLEE
>PF08_0138, 375 bases, 469C0E39 checksum.
MKVHYINILLFALPLNILVSSKKNTYIKYNTLNTKLIRAHRTLCECELYALSNYDNDP
EMKEVMQQFDRQSSQRFLEYDERMKTTRQKCKDKCDEAIQKIILKDKLEKELMDKFAT
LHTDMQSDAIPTCVCEKSLADKVEKGCLRCGGVLGGGVMPGFGAIGGTALYALNQLKP
TALKAAIAAANEAGIAAGKIAGDAAGMKVVSFGLKHFRVDELFPDIFKNFVNTKPYNE
ITTIANSILENYRAICTGVENIKAPTACKTFEYKFGIFIEGSRKPGPPAYNAIPQGLK
EISIKAEQAAATASKNASETVSTAIKARETALIEAGFNSSITSINASIFAIVVIVLIM
VIIYLILRYRRKKKMKKKLQYIKLLDE
>PF08_0105, 388 bases, 66769F checksum.
MKVHYINILLLYAVNLSILLISSHINIQKNPYINALHTLNTTKIPTNRFLCECDLYTS
IYDNDPEMNMVMQQFDERTSERFHEYHERIQEKRKKCKEQCERDIQKIILKDKIEKEL
TEKLDALQTDINTEDIPTCVCEKSIADKTEKYCLKCGYGLGSVSPNVGLIGAIAVNVW
KTGALIAAKNAAIAEGAAAGKAAGEAAGIATAIDELYNNFFLTILGRKSVQDVITAKN
FWDPAFIYKVVKLESNNVCFITPQPQTHASFCSLESLRNTPDFFEKYVVAGTQTVVSK

SSNAAAQATKDITEQVTTAAIESSKNTIEAACFTYHTAIIASIVAILIIVLIMVIIYL
ILRYRRKKNEEKTTIYKIIKGIVMFCCRYIKRNLIFYVIS
>PF08_0104, 332 bases, CB5FD5D3 checksum.
MRIHYINMFLFSLMFNILLNVNKNHYNTTLHTPNTTKIPTTRLLCECELYAPSNYNND
PEMKQVMDNFDRHTQQRFHEYEQRMIKNRKECKEQCEQNIQKLILKEKIQKELKEKFS
ALQIDISTHDIPTCVCEKSVADKTEKFCLKCGYGLGGALTAWQIFGYTGIYGWANYAA
LLAHEAGVKAGIKVVIDMFSTYPGLISLPGVDLTKMINGSNFNIPMELVNIVKGLSNG
LCKTPNKHLFCAFTNMEKGQSLVSFANSASQSGIMEAASVQGAKVAIIKTTTADFSYN
MIVSGITIFVIVLVMVIIYFILRYRRKKKMKKKLQYIKLLKE
>PFI0010c, 376 bases, C63A9389 checksum.
MKVHYINILLFALPLNILIYNQRNHNSTTHHTLKIPITRLLCECELYELANYDNDPEM
KEVMQQFEDRTTQRFHEYDERMKTTRQKCKDKCDKEIQKIILKDKLEKELMDKFATLH
TDIQSDAIPTCICEKSLEDKMEKECLKCAQNLGGIVAPSTGVLGEIAALAVNAWKTEA
IAAATKAAIAKGTAKGLAAGAAKGVAEVIAQVESQFRLSTIGVKELGSIFNASNYTNE
TFISGYIYAQYQGSQCGSLSMLLGKSKPFCTFVEGRIFATSVRVGRSFSPEDFIKTTV
QTIVKNAKTTAEATKAQVASAEKAAVLETSKKAIEATTTPYYTPIIVSIVAIVVIILI
MVIIYKILRYRRKKKMKKKLQYIKLLKE
>PFI0015c, 359 bases, 9D20BEC3 checksum.
MKLNYTKILLFFFPLNILLTSYHVHSKNKPHTTPHHTPTTTSRVLSECDTQSSIHDND
EEINSVKEVFDRQTSQRFEEYQERMKEKRQKRKEQRDKNIQKIIQKDRMEKNIAEKIE
KGCLMCGCGLGGVAGSIGLFGGVAINIWKPAALKAAIKTAIANSADKIAEAANSAGIQ
AGKEFVIAGLKRMGISTLGGKDLGTYFATTSYKNITNIAQAVYEQHFEKCADYPLWSV
RVPSSAAKHDFLFCQSVSNQIPSVSQPKQYISRIDVVQKTVQNIVTKAEGVAADASAV
KSAELTAEITKEQTTAINTIFMSKQTAIIASVVAILIIVLIMVIIYLILRYRRKKKMK
KKAQYTKLLNE
>PFI0020w, 343 bases, 3C03B2D5 checksum.
MKIHYTNILLFPLKLNILVNTHKKPHTTARHTQKIPTTRSLSECELYAPVNYYSDPQM
KEVMDNFNKQTQQRFHEYDERMKTTRQKCKDRCDKEIQKIILKDKLEKELTEKFATLQ
TDIQSDAIPTCICEKSLADKVEKGCLRCAQNLGGVAPGWGLLSGFDYVTWSQYISGIA
AKAAADAGLKAGVKVGLVNVVKIVRNTLGSAGEVPPMDWEKLIIFGNFSDGVTLHAIF
KNLDNMMNGYLDSRKYSQFSMVVQKFAGNFKPITAKYSKEVAEVTKAVADAKTGVLTK
AGNATSSLSTGITASIIAIVVIVLIMVIIYLVLRYRRKKKMKKKLQYIKLLEE
>PFI0030c, 367 bases, 56182295 checksum.
MKIHYINILLFVIPLNILIYNQRNHNSTTPHHPPNTRLLCECDIYTSIYDNDPQMKAV
MENYNRQTSDRFKEYDERMQGKRQKCKEQCDKEIQKIVLKDKIDKELTEKFATLQTDI
QSDSIPTCVCEKSLADKTEKFCHNCGYGLGSVAPSIGLLGGPGIYGWKTAALAAATKD
AITKGLAAGEAARIKEGINAVIAGIKSTFSIDKLGGEALKSVITAQKYNKVALISESI
YSEFSRSGCGDITSSSFLKNPFCTSVYEGIDAISGGNGVSPEDFIKRTVQSMVSDANG
VANAAAEIAEATEKAKAIKTSTDAIEAASTQLYGAIGYSILAILIIVLIMLIIYLILR
YRRKKKMKKKLQYIKLLKE
>PFI0035c, 381 bases, BA6B98F checksum.
MKFNYNNILLFSLSLNIFLLSSQVYNQRNHYITRTPKTNTRTLCECELYAPSNYDNDP
EMKAVMQDFDRQTSQRFEEYNERMMKNRQKCKEQCDKEIQKIILKDKLEKELMDKFAT
LHTDIQSDAIPTCVCEKSLADKVEKTCLKCGGVLGGGVTPAWGLISGIVYTGWKAAAL
AAAKKLAAEAGAAKGLAAGAEVGKDAVILGLNSEFGLSTQAVQKIGLVINGTNYTNVS
MITEAIFTKYKGSCMLSVPGASPGSFRILVTDPKFCNLFLKKFVPIGSGLDRNAITNA
IEKKVETMVLQAEGVAEATKTQVTSETTAELTAKQTTAINTIYMGKQTAIIASIVAIV
VIVLVMVIIYLILRYRRKKKMKKKLQYIKLLDE
>PFI0050c, 309 bases, 967AAE89 checksum.
MKLQYYKILLFSISLSIFFSSSYAHNKNKRYITPQTRTTTSRVLGECDLYMPNYDKDA
DMKSVKENFDRQTSQRFDEYEERMQEKRKKRKEQCDKDIQEIILKDKIEKSLEEKVEK

CCLICGCGLGGGVTPFIGLFGGLAVNEMKKVAAVAATEVGIKEAIKELGKIFELEAGS
NMQWTKMINAGNYSQKMSLVEIVTILKNKCEEDEALAGPLFCKASKAATESGEVFEFS
GNISRMAADAADAARKAANEKLAEMTSVGTICSNPVVISAIVVVIIAVILLIIYLILR
YRRKKKMKKKEQYTKLLNQ
>PFL1945c, 105 bases, 3AE846D5 checksum.
MKLFKILYLIAALLAINLIAPSVCNENVEGKKKKKGCPLRNFNFQEFRKKHHKAILIS
SVVSAIALLFGTAYGIGLHLNNKTFIKSILDLGKKRSASRSPHLKLK
>PFI0065w, 378 bases, C08A936C checksum.
MKIHYTNILLFPLKLNILVNTHKKPHTTARHTQKIPTTRSLSECELYAPVNYYSDPQM
KEVMDNFNKQTQQRFHEYDERMKTTRQKCKDKCDKEIQKIILKDKLEKQMEEQLTTLE
TKIDTDDIPTCICEKSLADKVEKGCLRCGSVLGGGVMPGFGAIGGTALYALNQLKPAV
FKAAIKAALEEGAAEILAAANAAGNAKGMEIVIHGLKLFGVDKLFPEIFNPFVAKNIY
TEITNLASTINSKYSTTCTWLKSGATAHPACNDFQLGLGIHLPNGSTLGIPPYTAIRQ
GLKGILQKATQTADAVADAKSAQVTAAITKNQTALLEAGFNSSITSIYASIIAIVVIV
LIMVIIYLILRYRRKKKMKKKLQYIKLLEE
>PFI0070w, 350 bases, 2AE7E7B0 checksum.
MNVHYINMLLFAFPLNILVTLYHEYNQRNHKSATFHTSNTKPTKTHRTLCECESYSPS
NYENDPEMKEIMEIFNHQTSERFREYDERVQNKRKQCKEQCEKDIQKIILKDKIEKQL
AQQFSPLQTNIDTNDIPTCVCKKSLADKTEKVCLNCGKTMGAVAPAWGLLSGLGYAGW
SHYVAASILEEGIKKGIEASLIKITEIATHIYQVSTADIPKITTTQILSSGHFTNNVS
FFDMVNYINNYMYNTLESEGYSQFCWAISSMASRKPITAFNKTYAIYSEAVTKAVTGA
ENGVIADLAPSTSSITTTIIASIVAILVIVLVMVIIYLILRYRRKKKMKKKLQYVKLL
KE
>PFI0075w, 365 bases, 8E5CCF5B checksum.
MKVHYINILLFAFPLNILVTLYHVNTHKKLSTTTCDIQTTRLLCECELYAPANYDNDP
QMKEVMDKFSKQTQQRFEEYDERLQEKRQICKDKCDKEIQKIILKDKIEKQMQEQFAT
LQTDIQSDSIPTCVCEKSLADKFEKTCLKCGGVLGGGVAPELGLIGGTALYGISVWKP
KAIASAIVEAQKAGEAAGKAAGDIAGVAEVISGLKSKFSLDTLFGTTLDKFITTKNYV
NETVISGAVKLQYQVSCATDPLGETSVLCFYKTIGEPNATAAVVENAKIVVTDAIQEA
SEVAFKVTASKTAEFKEINIATVESICNSYNTAIIASIATIVVIILVMIVIYLILRYS
RKKKMKKKLQYIKLLNQ
>PFI1805w, 371 bases, 794CF712 checksum.
MKLLHYCKVLLFSVPLNILVHNKNKSYITPQNTRNTRLLCECELYTSIYDNDTEMKAV
MGNYNQQTSQRFEEYNKRMNKNRQKCKDECDKEIQKIILKDKLQKELMHKFATLHTDI
QSDAIPTCVCEKSLADKVEKTCLRCTQNLGGIVAPSSGVLAGIAEGALIVWKPAAIKA
AKAAKAAAAKAASDAATQAGIEAVRLEINKLSVYFNGTTGFVDLTPIVTSSTYDNGLV
LVECAKKLIGDLSSNGRGGYTGFYNTAIHTESGELYVGNFGGIGKAAHDAKLASETTA
LTEAKVGAVDTTYGGFQTYITASVIAIVVIILIMVIIYLILRYRRKKKNEEKTPVHKI
IRRINIMCLAFFIDIFVYEILLV
>PFI1815c, 369 bases, EF13EBCB checksum.
MKIHYTNILLFPLKLNILVNTHKKPSITARHIQTTRLLCECELFSPQNYDNDPEMKRV
MQQFHDRTTQRFHEYDERMKTTRQECKEQCDKEIQKIILKDKMEKQMAEKLSTLETKI
NTDDIPTCVCEKSMADKTEKFCLNCGKNMAAIAPWWGLVCGSGYAGWLHSAMAAAIDK
AIAEGAAAGIKAGHLAGTNAVIEQLRTLGIYFVGNKQLETIIDVTNYMNVSFIYDKVY
SHYITLCTPRPVNGHLVSNFNFSDRFCKLFHQKDLVSLDIKSVKAIIKKNVEEAVAGA
EQAAKAEVSNVTATKTTEFTTKNIAEVEAATTSYYTPIIASIVAIVIIVLIMVIIYKI
LRYRRKKKMKKKLQYIKLLEE
>PFI1825w, 367 bases, 9E7FDB7A checksum.
MKVHYINILLFELPLNILIYNQRNHNSTTPHHPPNTRLLCECEWYAPSNYDNDPQMKA
VMQDFDRQTSQRFEEYNERLLENKQKCKEQCDKEIQKIILKDKLEKELAEKFVTLQTD
IQSDAIPTCVCEKSVADKVEKTCLKCGGVLGGGVTPAWGLLSGIVYTGWKAAALAAAK

ELAEKAGAAEGASQGAAAGAVEVIKSIKTTFGIESLGVNPLQSLFNAENYMNAKFISE
SIHSEFISSGCGAILKPPNKPICTSVYKGIEATSRGKGVKPIEFIGKKVETMVSQAKD
VAEATKAQVTSETTATLTDLKTGVVQTTYMGYQTVIIASIVAILLIVLVMIIIYLILR
YRRKKKMKKKLQYIKLLEE
>PF10_0003, 345 bases, 361D0614 checksum.
MKLHCSKILLFLLPLNILVTSLSNVHNNNKLYNTPHHIPTTTSRMLSECDLYIPKYDN
DADMKSVKENFDRQTSQRFEEYDERMKGKRQKRKEQRDKNIQEIIEKDKMDKSLAEKV
EKGCLRCGCGLGGVAASVGLFGGLGIYVSKSAALATAIAEGAETAKAAGEAARIPAAI
DAVIKGITKVFGVSTLDGKELGTYITATNYTNFKTIALAINEQYNPLSCIIPDPGADK
SICPWVMRNFFAAKDSPRNVVSAYNSIEVAVKSIVLEAQSVTERAAKKATEDVIKSSI
AAVDAKYVICQNAIIASVVALLIIVLVMIIIYLVLRYRRKKKMNKKQQYTKLLNQ
>PF10_0006, 361 bases, 32F0C016 checksum.
MKLHYSNILLFFFPLNILVTSYHVYNKNKIYITPHHTTTTTLRMLSEYGVHTSIYKND
ENMKSLKENFDRQTSQRFEEYEQRMIRKRKKYKEQCDKDIQKIIEKDKMDKSLAEKVE
TGCLKYGCGLGGVAGSVGLFGGFGIYGWKTAALAASKNAAVAEATAKGVAKAIDLVKS
TFDVQNIAGQPLETVLNVINYTDVSNIYHLIYSQYKTTCISTSSTPVTGGPETFCISI
WNKSLSGLGSNVVFVEETKVIEATVKTIVSSAEKVAGEALEKATEGVIKTSTATIEST
YASCQIAIIASVVALLIIALVMIIIYLVLRYRRKKKMNKKAHYTKLLNQKIYSFKILN
PFLCCMNSRCLNT
>PF10_0393, 359 bases, E94B9A9C checksum.
MKVHCYNILLFSFTLIILLLSSSQVNNQMNHYNRAHMKNIEPTKSYRSLCECELYTSM
YDDDPEMKEIMHDFDRQTSQRFEEYNERMNKNRQKCKEQCNRDIKNIILKDKIEKELK
QQLATLETDISTDDIPTCVCNKSVADKVEKTCLKCGGVLGGAVPELGLLCGYGAYELV
KVAIGAAEKAAIAEGAKAGIAEGIRVAIKGIKGAFNIEFLDGKTLAEVITGKTFNNST
FFVEKFVQEYNTVCLSSTTYQDTLFCDYGSMFGGKVDNITAISLNAKNTAIKAGQAAA
KMTTETTKALTAEKTGEVTSTSAIFSNPMVISFIVVVIIVIILLIIYLILRYRRKKKM
KRKLQYIKLLE
>PF10_0396, 378 bases, 59FE4D31 checksum.
MKFNYTNIILFSLSLNILLLSSRVYNKRNHKSIILHTSNENPIKTHRSLCECELYSPT
NYDSDPEMKRVMQQFHDRTTQRFHEYDERMKTTRQECKEQCDKEIQKIILKDRLEKEL
MDKFATLHTDIQSDAIPTCVCEKSLADKTEKFCLNCGVQLGGGVLQASGLLGGIGQLG
LDAWKAAALVTAKELAEKAGAAAGLKAGDIHGMKIVIEGLKALKVDTLKSGIFNSFVN
NSHYTEVTGLAIAIDTEMNEVCSATYIGIHPICVVREKLGVIPKAGGTMVKQKDAITN
VLKQALEKATQSAEALSETTAEDVAAKLTAQKTGAINTIFMSNQTAIIASIVAIVVIV
LIMVIIYLILRYRRKKKMKKKLQYIKLLEE
>PF10_0397, 348 bases, E777225B checksum.
MKLQYSKILLFSLPLNILVSSSYAHNKNKSYIAPHIPTTTTRVLSECNLYIPKYDNDA
DMKSVKENFDRQTSQRFEEYEERMIKNRQKRKEQRDKNIQKIIHKDKMEKNLAEKVEI
GCLRCGCALGGVAASVGLFGGLGTYGWKIAATATAMELAKEAAEQAGAAAAADAGKNA
VIAGLKELGVQNIAGQPLVSYLTATNYHNATFISSAINNQYSPSSCIFVSSGTRSGPV
ARETFCTWVMQKSTAAANVQRSSVSFNDLIIKTIENIVTDAKKAAGEATEKAIEEVIQ
RSTAAVESTYASCQSAIIASVVAIVVIALVMIIIYLVLRYRRKKKMNKKAQYTELLNQ
>PF10_0398, 350 bases, 41D60D38 checksum.
MKVHYINILLFVIPLNILINDQRNHKSTTHHTLKIPITRLLCECELYTPANYDNDPQM
KEVMDNFNRQTQQRFHEYDERMVEKRMQCKDKCDKEIQKIILKDKLEKELMDKFATLH
TDIQSDAIPTCVCEKSVADKMEKGCLRCGSILGAAMPEMGSIGGSLLSALSAWKPVAI
EAAEKAAIAKATDLATQAGMREVVLKIEQFLKNFTEKEGLVNFTSVVNKSNFKCPTAL
FQNANELLSDSCIPDEVTNRTSTFCSTIAYGEKTTFEPFAQAGATTFQETLTAKTPVL
QARYTAAVKTAYGGYQTAIIASIVAIVVIVLIMVIIYKILRYRRKKKMKKKLQYIKLL
EE
>PF10_0399, 326 bases, F0FF1EC6 checksum.

MKLHFPKILLFFFPSNILLTSYHVHSKNKPHTTPHHTPTTTSRVLSECDTQSTNYNND
EDIKSVKEIFDRQTSQRFEEYEERMQEKRQKRKEKCDKNIQKIIQKDKREKSLEEKVE
KCCLICGCGLGGVAASVGIFGTIAVNEWTKAAIEIAKGIGMNKATSAGVAKGIEATID
GLKGMLGFGNLRGVDIPAMVTEKSFNDANLLVPSIQKAYYNSCSQIPTGSAKPPFCGA
VETNMQIVFSSAHSIAMESGELATKTTATLSPQFISDEITLXKTASTNLYNVITYSVI
VIILIVLVMLIIYLVLRYRRKKKMNKKQQYTKLLNQ
>PF10_0400, 372 bases, A7F45BD7 checksum.
MKFSYFNILLFSIPLNILINDQRNHKSTTHHTLKIPITRLLCECELYSPDNYDNDAEM
KRVMQQFEDRTSQRFHEYDERMQSKRMQCKDRCDKEIQKIILKDKIEKELSQHLSTLE
TNIDTNDIPTCVCEKSLADKVEKGCLRCGYGLGTVAPTVGLIGAVAVNELKKAAMAIA
IKDAIAEGLVAGETARIQASIKAVILGIKSKFRIDTLGGEVLESIITAQKYDDVSLIS
ESIYMQYQSTCLPQYVGHGADLSKPICHTVYTLDFVQGKVHVPGSLQGSIKKALEKIV
AEAKSNAVSETANVTTRQTAVFESRNIAAVDATYASYQTAIVASVVAILVIVLVMLII
YLILRYRRKKKMKKKLQYIKLLKE
>PF10_0401, 295 bases, 57990E94 checksum.
MKLYYFKILLFSLLLNILAHNKNKSYIAPHIPTTTTRVLSECNVYIPNYDNDPDMNSV
RENFHKKTEQRFHEYDKRMIKNRQKRKEERDKNIQEIIEKDRMDKSLAEKVEKGCLKC
GCGLGGVAASIGIFGTIAVNEWTKAATAAAIDLAIQEGIDAGVKVVIAEIKATNAFKS
IWYVELSSFINGSNYNTVDGLSAATKAALDSIGKRCTSSIVDRACNGILNNSDGWFSS
IAEAGKQAATAATESAKTTKLVDVTTTSTHLYSAIGYSVLAIFIILLVMVIIYLILRY
RRKKK
>PF10_0403, 370 bases, BF2901C3 checksum.
MKLHYTKILLFSLLLNILAHNKNKSYTTIHTPITTSRVLIECNVYIPNYDNDPDMKSV
KENFDRQTSQRFEEYNERMIDKRQKCKEQCEKDIQKIILKDKIEKELAEKFVTLQTDI
SINDIPTCVCEKSVADKVEKTCLKCGGVLGGGITPGWGLISGIVYTGWKAAALAAAKK
AAIAEGAAKGLAEGIKEGIQEVMKGLCRDFRLSEVDVKKLGLVFDGKNYNNTEYIFKA
IFSKFDESCMPSSTGAVRGASEPICNSVWSKFVFTNRSSSRNSIINAIKENVETIVSQ
AETTAGATKEMVTQEVTSAAIKTNTTAVNATYASCQTVIIASVVAIVVIVLVMIIIYL
VLRYRRKKKMKKKLEYIKLLKE
>PF10_0404, 334 bases, 3EF5E9E3 checksum.
MKLHYTKILLFFFPLYILEHNQNKPYITQHHTPTTTSRVLSECDIPTSNYNNDEDMKS
VKENFDRQTSKRFDEYEERMKDKRQKRKEERDKNIQEIIEKDRMDKLLAEKVEKGCLR
CGCALGGVAASVGLFGGLGIYVSKSAALATAIAEGAETAKAAGEAARIPAAIDAVIKG
IETKFGVSTLGGQKMETFFNTTLYTEVSKIADAVYNQYAATCITSGNGARGPICTLAW
QYSESRGNVVSAYNSIEVAVKPIVSQAETVAERAVKTATDEAIQRSIGVVESTYAICQ
TAIIASVVAIIIIALVMIIIYLVLRYRRKKKMNKKLQYTKLLNQ
>PF11_0009, 340 bases, 4B8DCDDF checksum.
MKIHYTNILLFPLKLNILVNTHKKPSITPRHIQTTRSLCECELYAPANYDSDPEMKRV
MQQFHDRTSQRFHEYDDRMKTTRQKCKDKCDKEIQKIILKDKLEKQMAQQFSTLHTDI
QSDAIPTCVCEKSLADKVEKGCLRCAQNLGGVAPGWGLLSGFGYVTWSQYISGIAAKA
AADAGLKAGVKVGLVNAVKIVTNTLGSAGKVPTMDWEKLIIFGNFSDGVTLHAIFKNL
NNVMNGHLDFGKYSEFSTVVQKFAEKFKPITAKYSTEIAEVTNAVADAKAGVLTKAGN
ATSSLSTGITASIIAIVVIVLIMVIIYLILRYRRKKKMKKKLHYIKLLEE
>PF11_0010, 323 bases, AE4AA79C checksum.
MKIHYINILLFALPLNILVNNQRNHNNSTYHTSNTKTIKSHRSLCECELYAQSNYEND
QEMKDVIKEFNDRTAQRFEEYNERMQVKKDQCKEQCDKEIQQIILKDKIEKELTERFS
ALETKIDTNDILTCICEKSVTDKFEKTCLKCSGIFATAVPELGLIGGTVVYAAAVKAA
TKAGMEAALVGLESVNGLRGLLGEKIKDLVTTTNFQCPNALMGLVQNVKDTQCVGAAA
QSQVFCKGLLPESTSRIIQKAAAAGREGAEAYNTTFSDSTTITAFLTDPIVISAIVVI
SIVVILLIIYLILRYRRKIKMNKKLQYIKLLKE
>PFI1445w, 1364 bases, A5B7A430 checksum.

MIKVTIFLLLSIFSFNLYGLELNEKVSIKYGAEQGVGSADSNTKLCSDILKYLYMDEY
LSEGDKATFEKKCHNVIGNIRNTFSNKNTIKEGNEFLMSILHMKSLYGNNNNNNAGSE
SDVTLKSLYLSLKGSQNTEGESEVPSDDEINKTIMNFVKFNKYLLDNSNDIKKVHDFL
VLTSQSNENLLPNKEKLFEQIVDQIKYFDEYFFASGGKIKVKKGYLKYNFLDIYKQPV
CSAYLHLCSRYYESVSIYIRLKKVFNGIPAFLDKNCRKVKGEEFKKLMDMELKHNHIV
ERFDKYIISDDLYYVNMKVFDLKNVDKIQVSKIDDINNLNIYEHKETMHLSAKNLSRY
IDIKKELNDEKAYKQLMSAIRKYVTTLTKADSDITYFVKQLDDEEIERFLIDLNFFLY
NGFLRITEDKHLINADDVSPSYINLYRSNNIVALYILKTQYEENKLSEYRAHKFYRRK
RVSNITNDMIKKDFTQTNALTNLPNLDNKKTTEYYLKEYENFVENFQPDLHDIMKLQL
FFTMAFKDCNVNQNFTETSKKLWFDLLYAYDKFGWFYIHPNEVINSINKTDFVRHVLV
SRNFLLKNNDQLTFLETQVAKIVEIINLSLEVDKSPDSLDFSIPMNFFNHKNGYHVMN
DDKLKLLTSYEYIDSIANNYFFLSEYKNDVFRTGNNFKLYFNLPNIYSLAYQLFNELA
ININVITNVPLKKYLKYNASYAYFTLMNMIGKNHDIYSKGSRFVYASYILGLVFFIES
HIDIARLKPKDFFFMKQSLPIIDHVYHKDLKTLKKNCTLLTDFMKINKNSQNYSLTHT
EEMIKILGLLTVTLWAKEGKKSVYYDDDVSLYRKLMVSCVFNGGETIQEKLANNIEKS
CDISQYGIKSKNLKDMIDINLSIHKWNPAEIEKLAYSFVLSCKMQKLMYKPMNVEKLP
LEDYYKLSLAPDMVKTYHCYKLGKQAAELLESIILKKKFVRFRVTDAIDVYDFFYIKK
VLSSRIKKEYNEFLQDKRAFEKKELETILNNSPFSEEQTMKLINSYECHWFTSYENFR
ILWMHASSNLGTGTYLKNFFSELWQNIRFLFKSKLKIRDMEYFSGDISQMNLLDYYSP
MVHSESHCQEKMQVLFITLRDSKEENRSEIAQKVKSAYYQCKLDYYKNHHSDFIHRIH
PNDFLNNKVYVLKQPYYLMSNVPLNNPKKVSRLFVTEGTLEYLLLDKINIPECFGPCT
KLHFNKVVIKESKQRIYDMTINNALVPEIQPYNRRKYMTIYINEAYIKNIVSDALTSE
EIKRHDIQKGNIKICMGKSTYLTEPILTEEHFNLTHKPVYDFSSVKHNLKVFHMKNEH
LVSEDPNDDCFINYPLATINLGKHKKIYNK
>PF11_0011, 327 bases, 3D96A0A3 checksum.
MKLHYTKILLFSLQLNILMLSSYAHIKKKIYITPQIPITTSRVLSECDVQTSIYDYDE
DMKSVKDNFDRQTSHRFEEYEKRMNVKRQKCKEQCDKEVQKIILKDKIEKELTVKFGA
LHTHITIEDIPTCICDKSLSDKVEKTCLNCGGILGTVVPGWGILGSIGFYGFVNSIAI
DVAVKEGIAKVLHELKEITALEILLNNKLEALVTPETYACTNALNKSIMAAKLTICKA
SPQPAPCSPKLFDLSVIAPKVHNATMEGINTAKAAEVNTWNTAFSSPAFFSNPIVISA
IVLICIAVILLIIYLILRYRRKKQMNKKLKYIKLLKE
>PF11_0021, 330 bases, D3A17289 checksum.
MKVHYINILLFALPLNILVRLCNVGSPHKNPSITPKFPPNTRLLCDCELYSPANYDSD
PEMKAVMQGFDRQTSQRFEEYNERLLENKQKCKEQCDKEIQKIILKDKLEKELAEKFV
TLQTDISINDIPTCVCEKSLGDKVEKSCLKCGKNLGGFIPGLGLIGGTAVYAAAVNAA
TKAGMKAALDELKSVNGLFQLLGENIKDLVTATNFNCKDALLRRIDTITTPLCKSDEA
VNNLYCSLKSGKQPMGFSKIKSKISNAAEWAASASDEAKAEALNATFTWETFFSSPLG
ISLLVTVCIIIILSIIYLILRYRRKKKMKKKLQYIKLLEE
>PFL0010c, 378 bases, BFE5F602 checksum.
MKIHYINILLFALPLNILIYNQRNHNSTTHHTLKIPITRLLCECELYSPSNYDNDPQL
KKIMENFNKQTQQRFEEYDERMKTTRQKCKDKCDKEIQKIILKDKLEKELMDKFATLQ
TDIQSDDIPTCVCEKSLADKVEKNCMKCTQNLGGIVAPSSGVLAGIAELGLNAWKTTE
ITAAIAAAEQAGAAKGLAAGAAKGVAEVIAQVESQFHLSTIGVNELGSVINVTNYNNS
QFIFKAIFTKYRGSCLPSPPDRLTVAAGEETFCRTVNALGFTRRNVFDPSLLQDSIKS
VGNQIVTKAETAASTETAKVTASETATLKAAKVGEVNATYMGYQTPIIVSIAAIVVIV
LVMVIIYLILRYRRKKKMKKKLQYIKLLKE
>PFL0015c, 345 bases, D391267A checksum.
MKLHCSKILLFFFPLNILVTSYHVYSKNKPSITSHHTQTNRSLCECDTQSTNYNNDEE
IKSVKENFDRQASQRFEEYEERMKEKRQKCKEQRDKSIQEIIEKDKREKSLAQKVEKG
CLICGCGLGSVAGSVGIFGTVAVKELTKAAIAKAVVVAKEAGMAAAKAEGAAAGKEFV
IAKLQEMGISTVGVQGLQSYFVTTDYTNALTIFRAINNQYSPSSCIFVSSGTRQAFCT

WVTEQSKSVANIREMLQGNSVSYTKVTEKAVGTIVTDAKKAAGEATEKAIEEVIQRST
AAAESTYAGCQSAIIASVVAIIIIALVMIIIYLVLRYRRKKKMKKKAEYTKLLNQ
>PFL2585c, 375 bases, C547831B checksum.
MKMKHVYFKVLLLFSLPLNILVTLHHVDKKTNDYRPYTLTYTKLENTITSSRLLCECD
TYAATNYDNDPEVKKVMQQFDEHTTQRLLEYNEHMIKKRKECKEQCDKDIRKIILNDK
IEKQLKEKFSSALQTDIGANYLPKCTCEKSLSDKVEKTCLKCGGLLGGGITPTVGFFS
TIVTYLLTDAAAKQAGILAAKKVSVEAGIQTFIQQLSDMSNLHVLIGHKLVNLITPKT
VGNIMELSKTIHETLQSTCFAAEPRINDVMTILCSTDSYIFDAQYLQQQVANIATKAA
EEGAKAGALASQNAEANTWSLGTISNFFFTNSAGIAFTTIVLIAIILLITYLILHYRK
KKENEEKTTIYKIIKGIDMFYYVDVYR
>PFL2605w, 346 bases, BF2F294B checksum.
MKLLHYCKVLLFSLPLNILVYSKNKPYITPRHTPNTTSRVLSECDINTSIYHNDPDMK
SVKENFDRQTSQRLREYDERLQDKRQKRKEQRDKNIQKIIHKDKMEKKLAEKIEKGCL
MCACGLGSVAGSVGLLGGFGIYVSKSAALATAIAEGAETAKAAGEAARIPAAIDAVIK
GITKVFGVSTLGVQRLESLFTANIYNNVTMIARAINEQYNPSSCILPIGGSGADKSIC
PWVMEKYLPAQNIPEMTRGGALSMNDVIETAVKSIVTDAKTVAETAAKKATEEAIKAS
TDAVESAYAACQTAIIASVVAILVIVLVMMIIYLILRYRRKKKMNKKQQYTKLLNQ
>PFL2615w, 351 bases, D0F6F148 checksum.
MKVHYINILLFALPLNILIYNQRNHKSTTHHTLKIPITRLLCECELYTPANYDNDPQM
KEVMDNFNRQTQQRFHEYDERMVEKRMQCKDKCDKEIQKIILKDKLEKQMEQQLTTLE
TKIDTNDIPTCVCEKSMTDKVEKGCLRCGRNLGVAVPGLGVLGAYGAHSIVKVAMATA
EKVGIQLGIDAGNAAGIKAVIEALNSSLNIDNLGGITLDTVLKGNNFKNIDFLVYILT
DKYNTTCTVSNTEVETLLCYIGKEKPTLPYTLIQSNVRKAVAEATEVATSTTEEMTTI
YTTQELSKVTSTGAILSNPIIISFIVIVIVVIIFLIIYLILRYRRKKKTKKKLQYIKL
LKE
>PFL2625w, 379 bases, C8627C7E checksum.
MMLNYTNILLFYLSLNILSSSSEVYNQRNHFITYTPKRSTRLLCECELYTSIFDNDPE
MKSLIEHFNKQTQQRFHEYDERMKTTRQKCREQCDKEIQKIILKDKLEKELAEKFVTL
QTDIQSDAIPTCICEKSLADKVEKTCLKCGGVLGGGVTPAWGFLSGIVYTGWKAAALA
AATKEAIAEGAAKGAAAGTKAGIKAVMDVLYSDFGLSIEGVQKMGLVLSATNYKDVPM
ITKALYSKFQVSSCLRGGPVPGVPPVRPTDGTFCSAMLEKILAQENVVKQNSLEGSIK
SVVNQIVTEAKSAAVSETAKVTASETETLKATNIAAVNATYASSQTAIIASIIAIVVI
ILIMVIIYLILRYRRKKKMKKKLQYIKLLEE
>PFL2640c, 326 bases, F23A4A4C checksum.
MKVHYINILLFALPRNILVNTHKKLSITPPHIQTTRLLCECELFAPQNYDNDPEMKSV
MQQFEDRTSQRFQEYDERLQEKRQICKDKCDKEIQKIILKDKLEKELTEKFATLQTDI
HSDAIPTCICEKSIADKVEKTCLNCGKNLGGFVPGLGLIGGSALYVAAVKSSVELGIK
MGMQSVISDLKVLWDLTSLIKAKVIENFVTPTNYCNITSIIEFLQNINKSSCAAKTKI
TPLFCSTVEYQGPEELAKRVAGIVEQAQFAGAQAANEKFLELTTSYAILSHPIVISLI
VVVAIAVILLIIYLILRYRRKKKMKKKLQYMKLIKE
>PFL2645c, 317 bases, DC255E02 checksum.
MKIHYTNILLFPLKLNILVNTHKKPSITPRHIQTTRLLCECELYMSNYDNDPEMKRVM
QQFHDRTTQRFHEYDDRMIEKRQKCKDRCNKEIEKIILKDKIEKELTETFATLNTNIT
NEDIPTCICKKSVADKIEKTCLKYGGALGGGVMPGLGLIGGNSVYILANYETINAFIA
KTIEELEGIPGITKLFGAKISQFVTPAVFRKPMSLVETILSEKKKLCLCAANKNELLC
RGMNPNVPETLPKKIEVAVNEVLSSVNDTWATATTPTTFFTNPIILSAIAILVIVIIM
VIIYLILRYRRKQKIKKKLQYIKLLKE
>PFL2660w, 350 bases, 8C4AED86 checksum.
MKIHYINILLFPLKLNILIYNQRNHKSTTHHTLKIPITRLLCECELYTPANYDNDPQM
KEVMDNFNRQTQQRFHEYDERMVEKRMQCKDKCDKEIQKIILKDKLEKQMEQQLTTLE
TKITTDDIPTCLCEKSVADKMEKTCLRCAGVLGGGVMPGMGLIDGSLLGAISVLKPAA

IIAAKDAALAEATALATQAGMREVVLKIEQFLKLFSEKEKIFDLKLIVNKSNFSCGSS
LFQNAKELANKSCVAKPNGSYTSFCNSITYSRVEPFNGYAQAGITKYNETLPLQKALL
EKAKVDAVNTTYAAYHTSIIASIVAVVVIVLIMVIIYLILRYRRKKKMKKKLQYIKLL
EE
>PF13_0004, 343 bases, D2CDA707 checksum.
MKIHYTNILLFPLKLNILVNTHKKPHTTARHTQKIPTTRSLSECELYAPVNYYSDPQM
KEVMDNFNKQTQQRFHEYDERMKTTRQKCKDKCDKEIQKIILKDKLEKQMAQQFSTLH
TDIQSDDIPTCICEKSLADKVEKGCLRCAQNLGGVAPGWGLLSGFGYVTWSQYISGIA
AKAAADAGLKAGVKVGLVNAVKIVTKTLDGFGEVPTMDWAKLIAFGDFSDGVTLHAIF
KNLNNMMNCYLDSGKYSQFSTVVQKFAENPRSYATPYSTEVTEVTKAVADAKTGVLTK
AGNATSSLSTGITASIIAIVVIVLIMVIIYLVLRYRRKKKMKKKLQYIKLLEE
>PF13_0005, 331 bases, 1A2D1D55 checksum.
MKIHYINILLFALPLNILVRNQRNHRKSILSTTKSELTKTHRTLCECELYAPSKYEND
PEMKEVMENFDRQTSERFRKYDERIQDKRKQFKEQCEKDIQKIILKDKIEKQLSQQFS
KLQTNIETNDIPTCICEKSVAEKVEKTCLKCGEILGTVVPELGLIGGTVIYASAQSAA
VKVGVSKAIELMKDIYFLGQVPFFDLVAKITPSNFNDKMSLIGIVHDASYTVCETTNA
QDTLVFCLSKYSIGRRNPSMFLSVTANQAKDAAEAAGEAATGTFSEMTSVGAIFSDAL
VISAIVVVIIAVIILIIYLILRYRRKKKMKKKLQYIKLLEE
>PF13_0006, 345 bases, 25C777B1 checksum.
MKLHCSKILLFLLPLNILVTSLSNVHSKNKPYITSRHTATTISRVLSECDIRSSIYDN
DEDIKSVKECFDRQTSQRFEEYEERIQEKRQKRKEERDKNIKKIIEKDKMDKSLAEKV
EKGCLRCGCALGGVAASVGLFGGLGIYGWKTAALATAIAEGAAKGAVAAEAARIAEGI
KAVIKGIETKFGVSTDGLQGFKSFFTANTYNNVKNIARAINNQYEPSSCPITVPVDSK
PICTWVRANFFAAKDSPGNFTSTYEVVETAVTSIVSDAEPVATAAAQQATEEAIKAST
DAVESAYAACQTAIIASVVAILVIVLVMIIIYLVLRYRRKKKMKKKAQYTKLLNE
>PF14_0004, 337 bases, EB7EF59A checksum.
MKVHYINILLFAFPLNILVNTHKKPSITPHHTPKIPTTRLLCECELFEPANYDSDPQM
RAVMDNFSKQTQQRFHEYDESLQSKRMQCKDKCDKEIQKIILKDKLEKQMKQELTTLE
TKITTDDIPTCICEKSVADKMEKGCLRCAGVFSGGVAPSVGLLGGIGVYGWKMGAPTV
AMELAKQAGIEEGVKTVIAQIKGISIFNPYLESVECSKYITKLNYDNVSGLMKAVQAA
IKSTGETCEANSGDIVCETIYNKVERFLPVVQAGKQATTTTTETVKNAALDTIESTAT
TCTTAITASIIAIVVIVLIMVIIYKILRYRRKKKMKKKLQYIKLLEE
>PF14_0005, 339 bases, 7520AA74 checksum.
MKVHYTKILLFSLPLNILVTSSSNAHSKNKTYITLRHTPPIKSRVLSECDLYIPKYDN
DAEMKSVKGTFDRQASQRFEEYEERIKDKRQKRKEERDKDIQKIILKDKVEKSLAEKV
EKGCLKCGCGLAGVATSVSIIGPIAVNEWTKAALLSAKSSAIAEGNIKGIEAGVNAGI
KAVIDGLKSKFSLDIVAGKALEDLVTKTTYLNKNLLSEPFHIQYQSMCVGPTADIDKP
LCAFNIKNDTTWALKAIDGNVEKIISEAIKTTDTVTSNVTASEIPSIEALEKAAIEIT
CSNFHTAIIVSVVAILVIVLVMVIIYLILRYRRKKKMNKKQQYTKLLNQ
>PF14_0006, 367 bases, 8345C648 checksum.
MKDHYINILLFALPLNILVYNQRNYYITPRHTETNRSLCECELYSPTNYDSDPEMKRV
MQQFVDRTTQRFHEYDESLQSKRKQCKDQCDKEIQKIILKDKIEKEFTEKLSTLQTDI
TTKDIPTCVCEKSLADKMEKVCLKCAQNLGGIVAPSTGVLGEIAALAVNAWKTTALKN
AIAAAQKAGDAAGKIAGESKGVETIIGILEQYYSIYELKGTPLKSFFATTHYTDISNI
ATVIDTELNTSCGLNSLANQAICGLRTKLGLVAKPGQVMVTQKEAITKMITNVVHKSE
ITAEAAKTEVAATKTAAAIKMNTEAIEAATTPYYTPIIASIVAIVVIVLIMVIIYLIL
RYRRKKKMKKKLQYIKLLN
>PF14_0008, 316 bases, 8FD37C07 checksum.
MKLLYSKILLFALALNILLTSYYAHNKNKPYIIPRYTPTATSRVLSECDIHAPIYYND
EDMKSVKENFERQTSRRFEEYNERMNKNRQKCKEQRDKDIQKIILKDKIEKSLEEKVE
KGCLRCACGLGGVAAGVGIIGAIAVNEWTKVATAAAVQKGIKAGIAKAIDDLGNIVGL

IQFDLIDWAAKIDGINFLKQNSLVSIVNEVYYKCIDIENSSDFLFCSATKAWDQQKST
LGLQIISNQAAEAAVAAGKAAETAEKAEIVLVNAKSSLLYSAIGYSVIAILIIVLVMI
IIYLILRYRRTKKMNKKAEYTKLLNK
>PFC0120w, 1417 bases, D30708CA checksum.
MVSFFKTPIFILIIFLYLNEKVICSINENQNENDTISQNVNQHENINQNVNDNDNIEQ
LKSMIGNDELHKNLTILEKLILESLEKDKLKYPLLKQGTEQLIDISKFNKKNITDADD
ETYIIPTVQSTFHDIVKYEHLIKEQSIEIYNSDISDKIKKKIFIVRTLKTIKLMLIPL
NSYKQNNDLKSALEELNNVFTNKEAQEESSPIGDHGTFFRKLLTHVRTIKENEDIENK
GETLILGDNKIDVMNSNDFFFTTNSNVKFMENLDDITNQYGLGLINHLGPHLIALGHF
TVLKLALKNYKNYFEAKSIKFFSWQKILEFSMSDRFKVLDMMCDHESVYYSEKKRRKT
YLKVDRSNTSMECNILEYLLHYFNKYQLEIIKTTQDTDFDLHGMMEHKYIKDYFFSFM
CNDPKECIIYHTNQFKKEANEENTFPEQEEPNRQISAFNLYLNYYYFMKRYSSYGVKK
TLYVHLLNLTGLLNYDTRAYVTSLYLPGYYNAVEMSFTEEKEFSKLFESLIQCIEKCH
SDQARQISKDSNLLNNITKCDLCKGAFLYANMKFDEVPSMLQKFYVYLTKGLKIQKVS
SLIKTLDIYQDYSNYLSHDINWYTFLFLFRLTSFKEIAKKNVAEAMYLNIKDEDTFNK
TVVTNYWYPSPIKKYYTLYVRKHIPNNLVDELEKLMKSGTLEKMKKSLTFLVHVNSFL
QLDFFHQLNEPPLGLPRSYPLSLVLEHKFKEWMNSSPAGFYFSNYQNPYIRKDLHDKV
LSQKFEPPKMNQWNKVLKSLIECAYDMYFEQRHVKNLYKYHNIYNINNKLMLMRDSID
LYKNNFDDVLFFADIFNMRKYMTATPVYKKVKDRVYHTLHSITGNSVNFYKYGIIYGF
KVNKEILKEVVDELYSIYNFNTDIFTDTSFLQTVYLLFRRIEETYRTQRRDDKISVNN
VFFMNVANNYSKLNKEEREIEIHNSMASRYYAKTMFAAFQMLFSTMLSNNVDNLDKAY
GLSENIQVATSTSAFLTFAYVYNGSIMDSVTNSLLPPYAKKPITQLKYGKTFVFSNYF
MLASKMYDMLNYKNLSLLCEYQAVASANFYSAKKVGQFLGRKFLPITTYFLVMRISWT
HAFTTGQHLISAFGSPSSTANGKSNASGYKSPESFFFTHGLAAEASKYLFFYFFTNLY
LDAYKSFPGGFGPAIKEQTQHVQEQTYERKPSVHSFNRNFFMELVNGFMYAFCFFAIS
QMYAYFENINFYITSNFRFLDRYYGVFNKYFINYAIIKLKEITSDLLIKYEREAYLSM
KKYGYLGEVIAARLSPKDKIMNYVHETNEDIMSNLRRYDMENAFKNKMSTYVDDFAFF
DDCGKNEQFLNERCDYCPVIEEVEETQLFTTTGDKNTNKTTEIKKQTSTYIDTEKMNE
ADSADSDDEKDSDTPDDELMISRFH
>PF14_0766, 359 bases, 59B06ECF checksum.
MKLHYTKILLFFFPLNVLLTSYHAHNKNKASITPHHTRSTTSRLLSEYDTESSIYDSD
EDINSVKEIFERQTSRRFEEYEERMITQRQKYKEQRDKDIQKIILKDKMEKNLAKKIE
KGCLRCGFGLSGVAGSIGLFGAVAINIWKPAALKAAIAKAITEGTADIAAAGVKAGEV
TGKVLVISGLKRMGISTLDGKDLGTYFATTSYKNITNITQAVSSEYVQKCISASSGSV
RFRLVDAQRDIHFCHSVWKQTSAVSTPKKGISYKEVIERTVETMVSKAEGPANTAAEI
AEAANKLAIEEAQEKVMEATIYNWYTTIGYSILAILIIVLIMLIIYLILRYRRKKKMK
KKAQYTKLLNE
>PF14_0769, 348 bases, 671F7E7B checksum.
MKVHYINILLFALPLDILEHNKNEPHTTPNHTQTTRSLCECELYSPANNDNDPEMKRV
MQQFEDRTSQRFHEYDERMVEKRMQCKDKCDKEIQKIILKDKLEKQMVEQFSTLQTDI
QSDAIPTCVCEKSIEDKVEKGCLRCGSILGAAMPELGSVGGSLLYALNTWKPAAIIAA
KEAALAEATDLATQAGIDTVVAQLKIEGLLASFTVKQRLVDLSSIVTSSTYNNGAILH
KSAMELASSYCHFEGTQSTPPFCSTIKYGQTTNFVRYAKAGSAAFKTEFASKSATLTK
AKVGAVEATYGGYHISIISSIVAIVVIVLIMVIIYLILRYRRKKKMKKKLQYIKLLEE
>PF14_0770, 344 bases, 3759350F checksum.
MKLNYTKILLFFFPLNILVTSYHGNNNNKTYVTSHIPTNTSRLLSEKDLQSSIYDNDP
DMKSVKENFDRQTSQRFEEYEERMIPQRQKYNEQRDKDIQKIILKDKMEKSLEEKVEK
GCLRCGFGLGGVAAGVGIFGSIAVNELKKAAIAKAVVVAKEAAEKAGAAAAAEAGKNA
VIAGLEEMGISTLRGNVFETLFTANTYPNASEIALAINEQYNPSSCILPIGGSGPSET
FCTWVKVKSDAAPKIPGKVSSTYEVIETAVKSIVTDAKTVAETAAKKATEEAIKASTD
AVESAYAACQTAIIASVVAILVIVLVMMIIYLILRYRRKKKMNKKLQYTKLLNQ

>MAL6P1.251, 302 bases, C1223008 checksum.
MNIYYVKILIFTFLVNTLILSHNENCQNNQYNISLIKNNTQRTTIKPRLLAQTKNHNP
HYHNDPELKEMIDKLNKEAIKKYQKIHEPYEQLQELVEKKGTKPVGEHGTEPMSTIEK
ELLETYEKMFGDKKDIMLKSGMPPNDDDRSDKSITCECTDINNPDLTKAKSKDKYLKR
LKEGCTRGICTCSVGSAFLTLIGLAAAKKAAVAAFSAPYDACVSSTSLFYIFDSGTLA
SALKVGSTCASVATDMAGTVSSAATTAIIPFSISIYVLIVITVLLIILYIWLYRRRKN
SWKHECKKHLCK
>MAL6P1.315, 371 bases, 5440266 checksum.
MKIHYINILLFELPLNILIYNQRNHNSTTPHHPPNTRLLCECELYAPATYDDDPQMKE
VMQQFEDRTSQRFHEYDERMKTTRQKCKDKCDKEIQKIILKDKLEKELMDKFATLQTD
IQNDAIPTCVCEKSLEDKMEKGCLRCGGVLGGGIAPTFGLIGSVAINMWKTTEIAAAT
KAAIAAGKAAGKIAGEAAGKKAVIEALKYFGVDDFFPEIFKSILKMSRYTDVTKFGAA
IAEKHVLNCAMSARGGSVNDSTCNAFEIKLGLFEAETGKPNGPPAYQAIPQKINELAE
EATQAAAEAAKKASESATAAFETAEKEAIEAASMQLYTTIAYSILAILIIVLIMVIIY
LILRYRRKKKMKKKLQYIKLLEE
>MAL6P1.8, 323 bases, B5DE572E checksum.
MKIHYINILLFALPLNILVNNQRNHNNSTYHTSNTKTIKSHRSLCESKLYAQSNYEND
QEMKDVIKEFNDRTAQRFEEYNERMQVKKNQCKEQCDKEIQQIILKDKIEKELTERFS
ALETKIDTNDILTCICEKSVADKVEKTCVKCGRILGTAVPELSLIGGIAVYAAAQSAT
MKTFISETIDVLNRIGGMSQLFGAKISQFVTPSIYGKPMNLVIPLQEAINNACKCPDM
GDKILCGGIERSFEQNLPARIAYAVNQGVDTANDTWATATTPTTFLTNPFVASSIAIM
IIVAIVLIIYLILRYRRKQKIKKKLQYIKLLKE
>MAL7P1.2, 371 bases, 1F5B9110 checksum.
MKIHYTNILLFPLKLNILVNTHKKPYITHHTQKIPTTRSLSECELFSPQNYDNDPEMK
RVMQQFHDRTTQRFQEYDENLKEKRQKCKDRCDKEIQKIILKDKLEKQMAQQFSTLHT
DIQSDAIPTCVCEKSLADKVEKGCLRCGSVFGGGIAPSVGLLGGIGEAAISAWKVAAL
KAAARYAASKGAAQGLAAGIQAGKGVAIKSLEKLGVKYFWTGMSSEILKMNHYKEVAN
LTDVIYTAKLKVCDELTFDDFVNMCEQFDIKIGVYTEEVKNALLPKYAVPNALNEIVS
NAETTAKEVFEAESTRIAAEITEQQTAVINATYSSWQIAITASVIAIVVIVLIMMIIY
LVLRYRRKKKMKKKLQYIKLLEE
>MAL13P1.8, 422 bases, BAF51114 checksum.
MKLLHYCKVLLFSLPLNILVTST*NVCMK**KIYVSFYYLFNIPYTYLLQIYKIINYN
*I*HTYIYVMNSL*VHNKNKLYITPQHTRTNRLLCECELYTPIYDDDPEMKKVLHDFD
RQTSQRFKEYNERMQEKLKKCKEQCDKDIEKIILKDKIEKQMVDKFSA*ETNIDSNDI
PTCICKKTLADKVEKSCLKCTQNLGGIVTPSSVVLGGIAELGLSAWKTTALKVAIEAA
KEGGAAKGLAKVQAMGFANFFEGMKSGFCIKELDIKLLKAVFIQQKRENFTKLAHSIY
VQYNNKCGLAVAKNSYHTCNFGKALKCTELCSQANCSNEATIADGVNKILSTAKSAAA
NETANVIASETAAIEATKKGAIETTCMNCHTPIIASIVAIVIIVMVMVIIYLILRYRR
KKKLKKKLQYIKLLKE
>PF07_0001, 359 bases, 8ED8230C checksum.
MKLHYTKILLFFFPLNILLTSYHAHNKNKPSITPNHTRTTTSRLLSEYDTESSIYDSD
EEIDSVKEIFERQTSRRFEEYEERMITQRQKYKEQRDKNIQKIIHKDKMEKNLAEKIE
KGCLRCGCGLAGVAGSIGLFGAVAINIWKPGAITAAIEKALELKSAYISATAEAARIL
AGKEAVIEGLKKMGISTLEGKDLGTYFATTPYEKVASIYVAVNEQHFEKCAFGSLKKE
FIPVGDGSRDSLFCQSVWKQTSAVSQPRQYISPEEVIEQTVQNIVNKAEGPANTAAEV
ARESATNAIEVQQTNVINAIFMSKQTAIIASVVAILIIVLIMLIIYLVLRYRRKKKMN
KKAQYTKLLNE
>PF13_0002, 348 bases, EF586AB7 checksum.
MKVHYINILLFAIPLNILIYNQRNHKSTTHHTLKIPITRLLCECELYSPVNYDNDPQM
KEVMENFNKQTQQRFEEYDERMKTTRQKCKEQCDKEIQKIILKDKLEKELMDKFATLH
TDVQSDAIPTCICEKSVSEKVEKGCLECGGILGAAMPELGSIGGSLLYALSKWQTTEI

ATAIAAAQKAGIDAATQAGMNAVRLKIKEWYVYFSTEKIVDFTSVVKESNFSSASALH
ESAMNLLNNYCNFNGNLRRGYFCSTIKYGDKTTFPPFAQAGTEAYNATLPTKTAAFET
KYIDGVNTAYGGYQTAIIASIVAIVVIVLIMVIIYLILRYRRKKKMKKKLQYIKLLEE
>PFI1730w, 1340 bases, E26DCF16 checksum.
MIIWFIQPTIFYIIFILARNIQCTYKGDNINEIKSILDNDELYNSLSNLENLLLQTLE
QDELKIPIMKGDLDKYLNMSNFKILNELNADGAEKPYIIPTSNCSANDIVKYEHTLKT
QITLEYKPEISDMLKRKNIVVRTLKIIKFMQTPMSAYKNTNNIKQSLLEMNKLFTNKE
KKLNEHTINALRLRDRIFNTNNLTHRSIKKGISTYMPIDTKSDIIDYDDLLFTNHPSI
ELMENLDKLANYYHIGIFNMIGSHYIAVGHFITLKLALKNYKKYFEIGSLKYLNWQSI
LKFNQSDRFKVLDLICDESSWYEGQMKRREQYLKNNIFSTSEECSVLEFLIHHMNKYQ
MELYSKMHKLSLNVQIYLENKHLKEKFLQFMCRSRKECNIYESDRFKQEQEKGIEFHD
NNNYKFSQENDPVSKVVDPFNLFTNYFYFIKYYSVFNSDHIIYMHLLNFVGVLNGNNN
AYVSSLYLPGYYNAIQLSYKDQVGLKELYQNLVKCVEKCYIRNRKNRSFSHRIASIFR
HKKFDSSKCSICEGTLLYINDQSQDKMSMAQKFYIFVTKILKVNNISSFITNMNIYED
YSNYLMHDLNWYTFLFLFRMTTYKDIPNYSISNAMYLNIKDEDDTKRTMVTFQWMPST
IKRMHNYRIRKYVSIYLLEELEKLIDNKLIEKLKKCITFLIHLNAFLQLDFFSYLNET
PANLQHPFPISMMIEARFKDWFIQYLTGFFFINYDDANTRYNMPENMKRGTFIPPKYS
KWNIHLKRFIDEAFLMYFNQKHALTLFKYHNPYNISNKIMLMRDTFELYTKNYDQLIF
GADIMLLRKTFSCTPMSTKVWDRVKYYLHNIIGNPINFYKHGLIYAYTLNKAMLKEVV
NDFFVIYKMNKDLFSETSFLQTVYLLFKKIQGTYFSHRRNDDVSMNNIFMFNVEKNYS
KMSQADREKEIHESMASRFFAKNLFTVFQMMFVIQISNDVDKLDRIYGKADMLRLSVH
DEPFLRFAYAYYGSMYDKLTNVFFPMNIKKPTIQLKYGKTFIMANLYYLCSVLFSMYN
LNNLGLLCEYQAIGSANFHSYKKMSQFIDKKFIPLVFYTLKARTEGIIGKEWYKMVFN
DFDGKSMANTWPYFGYYMGGNMLYRNILYFPNHLPEELRKQTKGVELQQPEYEPSVHS
IDWQVGYAISHGLSLSFFTFGMMKAYAYFENVIFFLRNSIRIFDRFYSILENYVCMYI
KRLFNKLTVDKLLKAMSRAYTSTKKEGAYEEAMVSRVRNKENVVQEVQEDKGTDITPL
PTFDIMDSKQNTNYMYNDNEDYFDDLDDNEQFLNSKDLLYYDDGIDRTKRYELIPLQR
YRYDPF
>PF07_0136, 342 bases, 605765EB checksum.
MKLLYSKILLFALALNILLTSYYAHNKNKIYIKSHTPTTRSRLLSECDLYMSSNDNDP
DMKSVKENFDRQTSQRFEEYDERMKDKRQKRKEQRDKNIDEIIKKDRMDKSLAEKVEK
CCLICGCGLGGVAAGIGIIGGITISELKKAAMATAIVSAKEAGMAAAKAEGAAAGKEF
VIAGLKRMGISTLGGKDLGTYFATTDYTNFKTIARVINSEYQTDSCLIGGSGAEKPIC
TWVRAKEGAARVIQGKQFSTQETIKTGVENIVSNAETVAGEASKKATEDVIKSSIAAV
DAKYVICQNAIIASVVAILVIVLVMIIIYLILRYRRKKKMTKKLQYTKLLNQ
>PF11_0515, 314 bases, 8E73441A checksum.
MNLHYTIILFFCLPLNILEISSYEINKNKPYISPHTPTTLRVLSECDLHTSIYDNDSD
MKSVKENFDRQTSQRFEEYEERMIKNRKRCKEQCDKDIKKIIVKDKIEKSFAEKLEKG
CLRCGFGLGGVAASVGIIGPIIVNELKKTALVAAAQTGTEAGIDKAIEVVISKYGVNK
LYGVALEKSITSNNFKNVMFYIQAIQHRYNTMVCSAEPIDDIGPLCFLKDSLNDGVLF
TKSISASAQKVVADATEKATLVTKAEVSAAEATSVNLYYAIAYSVIAILVIVLVMVII
FLILRYRRKKKMNKKAQYTKLLNQ
>PF11_0529, 349 bases, BA3A9B4C checksum.
MKIHYINILLFELPLNILIYNQRNHKSTTHHTLKIPITRLLCECELYAPSNYDNDPEM
KEVMEIFDRQTSERFHEYDERMKTTRQKCKDKCDKEIQKIILKDKLEKELNEKFLTLQ
TDIQNDAIPTCVCEKSLADKVEKGCLRCGSILGAAMPEVGSIGGGLLYALNAWKPKAL
EAAIAAAKELAITEATNAGVKTVVSEINKLLAKFKQHEILFELKPIVNKSNFSCGSSL
FQRAEELASKSCVAQPNGSYTSFCNTILNGEKTTFKPFAQAGANTYEKTLTTETPVLQ
ARYTAAVKTAYGGYQTAIIASIVAIVVIVLIMVIIYLILRYRRKKKMKKKLQYIKLLE
E
>PFD0120w, 329 bases, 49EF20B1 checksum.

MKLHYFNILLFSLPLNILLLSSSQVYVHRNHYSITLYTSKTPKPIKSNRSLCECDLYK
PNYNNDPEMKNLMENFSKQAQQRFNEYDEHMNEKLQKCKEQCEKDIQNIILKDKIEKE
LTEKLSALQTDISINNIPT*ICEKSVADKMEKNCLKCGGIIGTAVPELALLGGVSTHM
LTTAATYAAIEAGMREVVSHIKEFLTNFNEYLVDLTPIVNESNYNCGTALFEAANKAV
STSCLTTKPGIISPLCSAIKTTGKNTFNHYAQYGSAAYNEKLNAEMVGVTSFNNAMMA
SIIAIVVIVLVMVIIYLILRYRRKKMKKKVQYLKLLNQ
>PFD0125c, 301 bases, D75BE51E checksum.
MLLFAFLFNAFLLSQRVKCINKIGNISLIKNNTQSISTKSRYLAEIERPNNPHYYNDP
ELKEIIDKLNKEAIKKYQKNHDPYEQLKEVVEKNQTKVTGGNDAEPMSTLEKDLLEIY
EKLFGDKSHIMIKSNIYINQDDKSNDKNDKSCECANYKKYNQLLSSYSVDDAYLHNLK
SGCVACVGTSALSSIITGLYGISAASTAAIEAIKTTSSATIITKLTKSLFGINFFSQT
SIESAYITAGITSVEGELYAGLVTSTFLPYGIVAIVLILVIVALIMLYIWFYRRRKSS
WKYAYKNHLST
>PFD1220c, 304 bases, 950FA7E1 checksum.
MLLFPFLFNTFLLSQNVNCQNNLRNKSLINNYTQRKILKSRCLAEIERPLNPHYQNDP
ELKKIIDQLNEEAMKKYQQSNDTYKQLHELLEKKETKSRSRNSEQEMSKIEKELLKIY
EKMSGDENHIMQKSDMYTNENHKSNDKNDKSCECINKNSTDKLSSTNKVHDNYLDNLK
TGFVGGLVVCALSSALVGKYGIAAAASAAKGVLQAFINALPSISSSQLTNILNGINFF
YETSIKSAFTFGGITSVEYEGAASLASSTFLPYGIAIIAIIVLTIIVIILYVWLRKRR
KNSWKHECKKHLCT
>PFE0015c, 347 bases, 41B4B257 checksum.
MKLYYFKILLFSLLLNILVTSSYI*IKKMIIIYIYIYVIFYYLYKYTIYIYIKNIYNH
K*LIITYMYTLHSL*SRNKNKTYVR*RRPTTASRVLSECDIETSIYDNGADMKSVKEN
FDRQTSQRFYEYEERMSKKRQKCKEQCEKDIKQIILKDKMEKSLAEKVEKGCLMCGCG
LGGVAASIGIFGPIAISELKKAALLAAIASAQKTGVLAGEAAGIEAGKEVIIGGLNAL
KIEILSIGPWETYFAEGYSINVKQLATVILRRRDAVCSATGLTPPGDTCNRISISIGT
KLPNGQYAAPGPDPVINVLNGIVERTKDAADAAAKTARETVTTGITEKETAAINTIF
>PFI0265c, 897 bases, 1EE18E4 checksum.
MRSKHLVTLFIITFLSFSTVKVWGKDVFAGFVTKKLKTLLDCNFALYYNFKGNGPDAG
SFLDFVDEPEQFYWFVEHFLSVKFRVPKHLKDKNIHNFTPCLNRSWVSEFLKEYEEPF
VNPVMKFLDKEQRLFFTYNFGDVEPQGKYTYFPVKEFHKYCILPPLIKTNIKDGESGE
FLKYQLNKEEYKVFLSSVGSQMTAIKNLYSTVEDEQRKQLLKVIIENESTNDISVQCP
TYNIKLHYTKECANSNNILKCIDEFLRKTCEKKTESKHPSADLCEHLQFLFESLKNPY
LDNFKKFMTNSDFTLIKPQSVWNVPIFDIYKPKNYLDSVQNLDTECFKKLNSKNLIFL
SFHDDIPNNPYYNVELQEIVKLSTYTYSIFDKLYNFFFVFKKSGAPISPVSVKELSHN
ITDFSFKEDNSEIQCQNVRKSLDLEVDVETMKGIAAEKLCKIIEKFILTKDDASKPEK
SDIHRGFRILCILISTHVEAYNIVRQLLNMESMISLTRYTSLYIHKFFKSVTLLKGNF
LYKNNKAIRYSRACSKASLHVPSVLYRRNIYIPETFLSLYLGLSNLVSSNPSSPFFEY
AIIEFLVTYYNKGSEKFVLYFISIISVLYINEYYYEQLSCFYPKEFELIKSRMIHPNI
VDRILKGIDNLMKSTRYDKMRTMYLDFESSDIFSREKVFTALYNFDSFIKTNEQLKKK
NLEEISEIPVQLETSNDGIGYRKQDVLYETDKPQTMDEASYEETVDEDAHHVNEKQHS
AHFLDAIAEKDILEEKTKDQDLEIELYKYMGPLKEQSKSTSAASTSDEISGSEGPSTE
STSTGNQGEDKTTDNTYKEMEELEEAEGTSNLKKGLEFYKSSLKLDQLDKEKPKKKKS
KRKKKRDSSSDRILLEESKTFTSENEL
>PFD0080c, 560 bases, 92E64A7A checksum.
MKIISNLGSMNITLFHLCFFGFLFVILNFTLDNSNFSNGGDILLNRNLSEEKAEDDYK
KSSSCSSLASTTKRSPSTASTSTSLRSASTASTASTLRSASTASTASTASTASTASTA
STASTASTASAASTTRSASAASTTRSASAASTTRSASAASTTRSASTASTARSASTAS
TASAASAASTTRSASAASTTRSASAASTTRSASAASTTRSASAASTASTASTGSTSTT
QSPSTSTSTTQSPSTSTSTTQSPSTSTSTTQSPSTGSTSGSTTRLPSTGSTIRFPSTG
SSRLEDYEKEGSNVDITEEEEKEAESVDDNNKEGNESDNIYCMLCSSNTRGFGCDCKD

AANYILKNLDLYDEHEAKKHASDRTKTFSKELSKLFKKLRTKDPNMFNKNKIVFQDLS
KKHTWDKYELHLPGLAHGKYNNERDVELNKKLIDLEDGFTDTQYIRKLYSLINENEKE
KFDDIKRKILCYCASKNKSLHRTPEESEQAWKYVQKLIKLYYNDFDKHFEKVFLIWLA
LSEGFDVFEYKMLIAANRLLWRKLSDKVQEESKVMIQD
>PF08_0036, 759 bases, 7130638B checksum.
MDIHAQENQTGIRFSWNLWPPTKAEAAKIEVPLGCLYTVLKRTDDSSVKLVEYEPLKC
KTSNCILNPYCNIDFRNKTWTCPFSNIKNPFPLHYAEHISEKNLPADVMYSNIEYIQP
SNVGDIPPPTFLFVIDTCLLEEELEQLKDSIQQCISLMPGDAYIGIITFGNMCYVHEI
GFNDCLKSYVFKGNKEISAQDLQKQLNLGSRNDPRSSTTSASARRFLQPVSECEYNIN
MLLEDIQKDNWPTPPDQRAKRCTGVALSVAIGLLECCCNQLSGRVMMFIGGADTTSPG
KIVDTPLSESLRHHLDLQKENSNARHVKKALKYYVSLANRAVASGHAIDIFACSLDQI
GLYEMKVCCEKTNGFMVMADSFSMNVFKDSFKKIFETDSTEYIKHGYNAKLTVICSKE
FRVCGAIGACSSNKKTANYVSDTCVGEGGTCEWTICALDRQSTIAFYFEIVNQNLASL
PPDRQAYLQFQTLYQHPSGRRRLRVTTISYRFAEPNIAEISQGFDQETAAVIMARFAV
LKAETDEPIDVLRWLDRKLIRLVSTFADYQKDDINSFHLSSEFSIYPQFMYHLRRSHF
LQTFNASPDETAYYRSILLRENVMNSLIMIQPALLQYSFDSPTPIPVLLDAQSLKSNV
ILLLDSYFHIVIWYGEMIYQWREQGFHEKPEYEHFRQLLNAPHEDAKSILEDRFPIPK
FVLCNSGGSQSRFLLAKVNPSTTHNSLSGSTFGTSSNESYIINTDDVSLKIFMDHLVK
LAVQT
>PF10_0013, 231 bases, AE9D5F4E checksum.
MLFSFKYIYFSSTFLLLLVLQSKTKYIQNFVNIQETLNEKCSSKYIRLISEQTSDEST
SKSIKNCKCVNKRNNKNEDNHELQSKSRSLKGTELDVTSYNKSKHTQQNNLGDSLKRT
KNNITNISSKKNETYLFDENTNEYKLRNTLRLMNERYADSIVDTMDLNDKTKDKFKKF
LHLYMAKENPDKQRKLYEQIESDIEKYKKNHVICSIVDFENIYDSFYFLKYEDRYIT
>MAl7P1.87, 1036 bases, 8866E66A checksum.
MHGSELKTCIGVCDNNNNKNYEQFRKLKSNNDTIMRREHLGEKREMNNGDGKELTILT
NKRTYSNDNYKNNMNNNRRSCSKISLLKYNEEIFKHQNDKCNDENNDMSDESHNYLYND
MYNDQYNDIHDDMPNNINNDIYNDIYNDKQSELYNDKSSDATIYNDKHCHMDKVQKHK
NDKNKKLKNVLSGISYQMKNAMNVFGEVKEHIKYTASEAKKNMNYPFCKAPCNIENLR
NTIFINEEEIVINKNIYKNIFNKKNIEKEQGKNKKENEELFSCNQSDIIIKGNYNFGI
DIEMVYVNEQNKIVVYAYDKETKKKIPCTYKWTRVYYNKECELVEKNVYPHKNVILYD
ENEYELTCEDIGTKIYVECSCINNEQINVENLSKFYLNGSTNSENCNYEEGVIDQKRI
EHKNKYNIDYNQNNRNDSHAVLHIGNDKSINDSNGLSSLKHCVTKNNKCDEEEHSRNH
VLEKKSMYLLHNYYNKNGKEKDDNNNNKKKNIYMYKEDTPPIKDLSSDAYASYESSIL
NKNSSSTIVLNNDKNIYNNKKYQGVAIGEIGPLNINEKTKKMLERIIENDTIRYPIYI
IKKVYNDEETCNDFINNDDMNRNKKDDTLKIYNDDNSLDSFTVNSNIESEHDMDIYML
YIHKNEIKIIDHNNMNHMNNVNNIMSNVKSMDKIKKNMWTHKFHYIYPYVHFMGTKHK
QNNKKDTFQLYINEKEYYICKCLYKRHRDLIAIILRYMHANLYIINDYIFNNINQNFE
KTRVKNIFENIDVNYILQNINKELLINRKITQKCYLKINKLQSEKNMLEEELKSTIQA
FQSQLDNVKKFKDENDLIQTNEKLMKEIKILQEKYRNVDLFFKNKTLISDIEKYKKLL
EENKSKVNEDDDNLLNENVQMCNIYNEEKKNKTELENKLKGLSIKLETLNNSLQEEKI
QRNINSESLKEMEELKRNNKDLQQKNVKISDEMNLLITEKNRLTKLVDSLTKDIEKSK
INKNVKSTDNKMDIDQDKEKLFKEISLLKDENELLKKRIKKLAKMSTIRT
>PFC0525c, 440 bases, 61EA65D6 checksum.
MKNWPIDEDINIYEEKNHTNNKNYVNNFEMSDQKDEEEYSHSSNRSEDEDEERTIDNE
INRSPNKSYKLGNIIGNGSFGVVYEAICIDTSEQVAIKKVLQDPQYKNRELMIMKNLN
HINIIYLKDYYYTESFKKNEKNIFLNVVMEYIPQTVHKYMKYYSRNNQALPMFLVKLY
SYQLCRALSYIHSKFICHRDLKPQNLLIDPRTHTLKLCDFGSAKNLLAGQRSVSYICS
RFYRAPELMLGSTNYTTHIDLWSLGCIIAEMILGYPIFSGQSSVDQLVRIIQVLGTPT
EDQLKEMNPNYADIKFPDVKSKDLRKVFPKGTPDEAINLITQFLKYEPLKRLNPIEAL
ADPFFDELRDPCIKLPKYIDKLPELFNFCKEEIQEMSMECRRKIIPKNVYEEFLMVDE

NDNNIINDTISNDFNESNLDTNNSNNKTHVIIES
>PFC0140c, 783 bases, 97CF3B28 checksum.
MRTNLQCCKLQSQELALTNCGFINIGLYNNLKKSVKSNDVYSEVGNMVLILRGDGNIG
REEIALNTCQREFSRIQLKELVEINILDKENKNDLVNFIPIDSIELEVNLFVKPDRLI
EMEDEKLEDVFKKYFLNHILTKGQILALKYNDILLKCIVKDLKTADFDEIKRLNNSNN
NNNNRNSSGFSGYFNKWSPGFNNADNQNLSYNRYERGILFENTECIFTSISDGGKLCI
ESKKVLKQNIIKNNFNFEELGIGALDEEFKTIFRRTFASRIYPNYIIKQLSIKHVKGM
ILYGPPGTGKTLIARQIGKTLNAREPKIINGPEILNKYVGQSEENIRNLFKDAEMEYK
QSGENSLLHIIILDEIDAICRQRGNVGSSSTGVNDSVVNQLLSKIDGVNSLNNILLIG
MTNRIDLIDEALLRPGRFELHIEISLPNKEGRIQILNIHTKNMRMSNKLSSDVNILEL
AERTPNFSGAEIEGLVRNTVSYAFERHINFNDLTKPINADDIMITKNDFMKALKETKP
AFGAEEDIIGNLLCNGIINYGKEYENIENTCKLLIKQIVDNENTKLMSILLYGENGSG
KTTISAYIAKCANFHFTKFITPENLIGYSEIGRINYINKIFEDAYKTPLSLVILDNIE
RLIDYTRIGPRFSNSILQAIMVLIKKKPKKENQKILIICTTSEYQFMKDVGLTKNFFV
NIHVPLLSTSSSIKNVLQHRNQTYHDFPNNEIEKVISSNIIKNIAIKNLLMIIDMASE
ASDDKNITSDVFLKIFNDCGLLLDDDTDY
>PFC0810w, 192 bases, 485A247B checksum.
MFIINWFRDILAHLGLSQKSARILFLGLDNAGKTTLLHMLKDDRVAQHVPTLHPHSEE
LVVGKIRFKTFDLGGHETARRIWRDYFAAVDAVVFMIDTTDRSRFDEAREELRHLLET
EELSNVPFVVLGNKIDKPDAASEDELRQHLNLFSNITVHNNMKGGSGVRPVELFMCSV
IRRMGYAAAFKWISQFLT
>PFB0640c, 1460 bases, EF4D3FDC checksum.
MALKSINISGNFEWCPFEEYKNYLLCFNSHNLLYSNNNSLNNYIYLLDINLNSEIRNL
EIVNKYNFEDALKYDNDVIKGGNKKNNKNNKNNHNNNSVNEYVTCFEWMNSNNFVDIN
NNEELSKGIIVGGLTNGDIVLLNAKNLFETNRNYDNFILSKTNIHDNGINCLEYNRHK
NNLIATGGNDGQLFITDIENLYSPTSYDPYLDKNNLQKITCLNWNKKVSHILATSSNN
GNTVIWDLKIKKSAVSFRDPHSRTKTSSLSWLSNQPTQVLISYDDDKNPCLQLWDLRN
SNYPIKEIIGHSKGINNICFSPIDTNLLLSSGKDVTKCWYLDNNNFDIFNEINNSANN
IYSKWSPYIPDLFASSTNMDTIQINSINNGNKMTSKYIPTFYKKEAGICIGFGGKICT
FDNSTNNMSNVNNMNNVNNMNNINSFNNDNSCDGEYDSNKGKNKSTQKKFLIKYHIYP
TDMELISEADNFEKYITSGNYKEFCESKINKCDDDHEKLTWQILQLLCTSQRGDIVKY
LGHDINNIVDKIMQTIGKQPGFIFKTLIDEKENNNNNNNNNSTNQMYQNDVLLHNDPN
LMNNYLLKDNMNPNIMLNNNNNNINNRTGTNVMYSNGQNLLGDTNHNEENFNGNFDID
PEKFFRELGEKTENEKIKQNEEDISGNDEHLLNSSIKGKENKTKNKKSGLGTDDNNDN
GDHNKNEGSNINGEHVSEHILNEKNNTNNWNLGIEAIIKECVLIGNIETAVELCLHKN
RMADALLLSSFGGEQLWHKTKTIYIKKQNDNFLKNINYVLDDKLENLINNVDLNSWEE
ALSILCTYAINNPNFNSLCEMLAKRLQNEKFDIRAASICYLCACNFSETVEIWNNMPS
KKTSLLNVLQDIVEKMTILKMIIKYENFNSIMNQKISQYAELLANSGRLKAAMTFLCL
IQHDQSIESLILRDRIYNSANHVLCQQIKPPISPFQIVDIKPSPNVYQNNMYNNNNNN
NNININSSSNNNNNNNNNKVLSSMHHPMQQFNQCNVNKMYTSTSNIINNNTMNSNFKS
VIPPPLPMNTQMNNSTSSIQPPPSVPPTKFHTQIINNTMNSRSSIATTTKNYPTSNLN
SVIPTSMNNMNTNISHGNNVTPPYMSQTNVAVPNMNNNNNNNNTMNPTYPSLPKFPNY
NLNSQVQQNSIIPEKQLTSPMFSSNSYGNINKTHTTNNAVPPPPNVTSSVVTPPMPSN
QLNNTRSSFADIQNVVSPPRNKNQSISSTANLNYQHDNQFNKRECMEQPVYPMTNQSS
MFSMNNTMQKKNVPGGFQDNTSQMNYGMQPTGSPPPSSLSTTSPIAGALTVTPGMPVP
WPIPTTTQQLGSTTQSTANENKKIQTATKEQNGVLMNRNHIENIKKTISNLLNIYTSQ
ESVKKKADDVSSKVYELFEKLDCGAFNEQINDSLLNLVNCINANDFKTTNKIIVDLSR
NLWDGSNKAW
>PFB0095c, 2441 bases, 3A86487C checksum.
MATIKKYHIRGRKNILIFLLKIFLFSPLIWILIYSEYFTVVKNYNKIDNVYNIFEIRL
KRSLAQVLGNTRLSSRGVRDPRTKEALKEKQFRDHKRKEALKQKTEKNEKARNALKEK

KLKEQKKNDAQKAKDLTKKESQDSSSEKSLKEKVNGEALKEKENKETLKKKELENQKE
KEEKNKIKDNNDEALKNKGNDKDDKKIVPKKPESVEKDLKEMELKEKEFIKQHLKDYE
ERKEKRRNWILRSLRRDKLREIEQLEKLNAQLESAINELKERRASRRPMMVKMQRGMK
DEVDEWIKKYDDEQAEKNGTKDEEIKDKGDGYEEIVETKFYGMRENALGELDEYEERY
EKKRYYLKEDGEGDLKDVEEKLEETGYGFREKFPTTRILVKRKRNKEQKKLKEDKEKK
LIAAEEPDDEKKIKLKDSDDKVVVPVNKNKSSFPDKFRAPDKKRTMFYRLSELFPIVP
RKDNELAVSGDCMDSKVNGKKLKSTFNPFKRRRNKLKERKMQELHKFKKNYKKYQKLL
EREKRENPDGEPLNTPEIHVIRPSDLMDKGENKSAGHPFKYQPTKGLKEYEESHVAKD
YQLEHEPPTKLPEYEKGHVSREYQLDNEVRDELPEYEKGHVSREYQLDNEVRDELPEY
EKGHVSREYQLDNEGPSTLKEYDQTELAKGKDITNKPHESVDEYDQSELAKGKDITNK
PHESVDEYDQTELAKGKEVTNKPHENLEEYNETDLAKGKEVTNKPHESVDEYDQSELA
KGKDITNKPHESVDEYDQTELAKGKEVTNKPHENLEEYNETDLAKGKEVTNKAHENLE
EYNETDLAKGKEVTNKAHENLEEYNETDLAKGKEVTNKAHENLEEYNETDLAKGKEVT
NKARENLEEYNETDLAKGKEVTNKARENLEEYNETDLAKGKEVTNKAHENLEEYNETD
LAKGKEVTNKAHENLEEYNETDLAKGKEVTNKAHENLEEYNETDLAKGKEVTNKAREN
LEEYNETDLAKGKEVTNKARENLEEYNETDLAKGKEVTNKARENLEEYNETDLAKGKE
VTNKARENLEEYEEKDYMKNNELQNKGSDGLKENAELKNKELRNKGSDGLKENAELKN
KELRNKGSDGLKENAELKNKELQNKGSEGLKENAELKNKELQNKGSEGLKENAELKNK
ELRNKGSEGLKENAELKNKELQNKGSEGLKENAELKNKELQNKGSEGLKENAELKNKE
LQNKGSEGLKENAELKNKELRNKGSEGLKENVYTNNDLKNNDIQNKDLSNKDMKNKEL
LNKDISNKDMKNKELLNKDLSNEDMKNKELLNKDIRNKDLKSIGNMEQQNTGLKNTPS
KGQQNTGLKNTPNERQQNTGLKNTPSEGQQNTGLKNTPSEGQQNTGLKNTPNERQQNT
GLKNTPSEGQQNTGLKNTPIEGQQNTGLKNTPSEGQQNTGLKNTPNERQQNTGLKNAA
NKGQQNTGLKNTPSKGQQNTGLKNTPNERQQNTGLKNTPNERQQNTGLKNTPSEGQQN
NDLKNTPNERQQNTGLKNTASKGQQNTGLKNAPNERQQNTGLKNTPSEGQQNTGLKNT
PSEGQQNTGLKNTPNERQQNTGLKNTASKGQQNTGLKNAPNERQQNTGLKNTPSEGQQ
NTGLKNSASKGQQNTGLKNTPSEGQQNNDLKNAPNERQQNTGLKNTPSEGQQNTGLKN
TPSEGQQNTGLKNTPSGGQQNTGLKNTPNERQQNTGLKNTPSEGQQNTGLKNTPNERQ
QNTGLKNAANKGQQNTGLKNTPNEGQQNTGLKNTPSEGQQNTGLKNTPSEGQQNTGLK
NTPSEGQQNTGLKNTPNERQQNTGLKNAANKGQQNTGLKNTPNEGQQNTGLKNTPSEG
QQNTGLKNTPSEGQQNTGLKNTPSEGQQNTGLKNAANKGQQNTGLKNTPSGGQQNTGL
KNTPNEGQQNTGLKNTPSEGQQNTGLKNTPSEGQQNTGLKNTPNERQQNTGLKNTPSE
GQQNTGLKNTPSEGQPNTGLKNTPNEGQQNTGLKNTPSEGQQNTGLKNAANKGQQNTG
LKNTPNEGQQNTGLKNTPSEGQQNTGLKNTPSEGQQNTGLKNTPSEGQQNTGLKNAAN
KGQQNTGLKNTPNERQQNTGLKNTPNEGQQNTGLKNTPSEGQQNTGLKNTPSEGQQNT
GLKNTPSEGQQNTGLKNTPSEGQPNTGLKNTPNEGQQNTGLKNTPSEGQQNTGLKNAA
NKGQQNTGLKNTPSEGQQNTGLKNTPSEGQQNTGLKNAANKGQQNTGLKNTPSEGQQN
TGLKNTPSEGQQNTGLKNAANKGQQNTGLKNTPSEGQPNTGLKNTPNEGQQNTGLKNT
PSEGQQNTGLKNTPNEGQQNTGLKNTPSEGQQNTGLKNTPNEGQQNNDLKNKASKGQQ
NNDLENDGLKHKPNQGQKHTELNNKNLKNKPTDGLKNVKDDELSDNESSDNEKSKKNL
RGKKN
>PF11_0507, 5507 bases, FC3EA3D3 checksum.
MKELESEEFPKEKYNIEDMEETEKENEIEKIMDENKETEQTEEGNTEEFVQEKELNQE
TLENEIIVDHIKEEEDTRNVKEQESVLEESPIEELPVENNIGKINEEVEEFILNEIPL
EEQVPKELPQEEIEEIVVEELPIDEHLSSEETTVTEEDTFKGQLINEEKPVEEKSVSE
EIPVEEKSVSEEIPVEEKSVSEEIPVEEKSVSEEIPVEEKNVSEEIPVEEKNVSEEIP
VEEKNVSEEIPVEEENVSEEIPEGGIAIEDVPVDEETVTEEITVDEKIYDKLPNEIET
VNEEMPVEDETLTEQISSEHERVPEEIIEEKPFTEGEETESLTDEIVEEGVVTDDIPE
EQIITEKVQEEEEFVTGELSEEDIINEKVQEEDESVTEELPEEDIINEKVQEEEESAY
QEIVQDGSVTKDVEYKELVNDDVRDKENFVIEEDPFKGQLINEGLPVEEEFVTKELPV
KEESVFEELTEEDQSVTKEIPVEEHSVFKEVDEIESVTDEIVEEEGSVNEEVEEEVSV

SGEVDETEYVTEEVEETELVNEEVLKEEGSASEKVVKEEGSASEKIVEEEGSVTEETA
SEEIVEEEGSVTEESSSEEIVEDEGSVTEESSSEEIVEEEGSVIEESASEVIVEDEVS
ASKEIVEDEASVTEEVVEEEGAVSDEVQVTESVEDEIINQGIVDEVIVEQEASVTDEI
VKEDESVIEEIAVEETVTEVVEETKPMDEEIVDQGSVVENVEEKKTMDEEIVDQGSVV
ENVEEKKTMDEEIGDQGSVAEKVEEEELVTEEVIEREGSINEDIVKEASITEEVEQIE
PVTGKVEKIESITDEIKEQLVPEDIKEEQLDFEEIVAQRASVNDEKVEVASITEEVEE
EEKSVSEEVLEEEGSTTEKVVKGSSTEVVVEEQGSVTENLLEEESASQGIVEKEEFVD
EEDSVKDQNVFEKEGSVTEQLVEEEKGLINEDNEKEELITEMSEEIKSVNEEIEETDL
STEEIIKQQGLATYEFVEEEKSLTDKLLEEESVTKEVGETELSTQEVVDEKVSVTEEV
IEEEKSVSEEVLEEGSATEEVVEEGSCTEVVVEEGSCTEVVVEEGSCTEIVVEKGSDT
EIVVEEGSATEIVVEEGSATEVVVEEGSATEEVVEEGSVSEEMLEEEGSATEEVVEEG
LSSDNVQKSKGVIENVGEIYSVKTAKDESMNEKIPLEKSSFVDDESFKGQGPTDNVSV
EDVNSEDIINEHTPLEETKIEELPTEYITTADIHTKGETETKYNLIYEKINEEVEKAK
FQEEKITENIPVERESVTEDIVQEPSLAQEVEQKESDTNEIEETKLANEKIIPEVSVT
ENVVEKEGLDTEEVLEEDESITEEIVEEEVSSSEEIVEEEESSSEEIVEEEESSSEEI
VEEEESSSEEIVEEEESSSEEIVEEEESSSEEIIEEVSSTEEVLEEEGSVTEEIVEEE
VSTTEEVKDIGSVSEEVLEEEGFGTEEFVGQQGSVIEEIVETESSTEKVLEDVGSNVE
EIVQEEGPVAQEIVHEEVSTTEKHDEVDRSTTEEIVEKVGSVSEEIIVEEVSASEEIV
EEGSVTEEVVEEEKLINEVGETESVTEEIVQKEVSDAEEVLGQEGSMNEEILEKESIV
EEIVGPEGSVTEEIVDHGSFAEEVKEEELVTEEAVQYEGSVTEEIKEEESITENEAIE
ESAFAEIIEEKGPNTDEIVKEEGLDTEEIVNEVSVTDEVIEEEKLVNEQIVGEERSVT
EKPVEVERSATEDLVEEEASVTEKVSVHEGSTTEQILDESVAEEIVEEEVSVDDKIIE
EEVSVDEVVEEEGSVIEEIVEEEESVPEEILEEELSGSEEVLEDEWVTDAFMGQEGSV
IEEIEEIVDGEGSITEEIVEDGSANEKIVEEEPSRVEEVLGKEGFVIEEIIEEGSVIE
QVEDTKTVSEKSEESSAIEEVKEVKEEESISEKIVEKEESVTEEIVRQEESTTEKIVK
DVSPTEDFVEQTDSVTEKVIEQEGSNTEVAEDVEEKESASDEHEQEDVSVNAQVTYEK
KSVTKEIVDEVSRTEEIVEENGSVTEGVDETGSVTEEIIEEATVTEEVVEDGSVTEEV
VEDGSVIQEVVEDGSVTEEIVQENGSVTEEIVEEEGSVNEEVEEEVSVSGEVDETEYV
TEEVEEEGSVVEEIVEEEGSVSGEVDETEYVTEEVEEEGSVVEEIVEEEGSVSGEVDE
TEYVTEEVEEEGSVVEEIVEEEGSVSGEVDETEYVTEEVEEEGSVVEEIVEEEGSVVE
EIVEEEGSVSEVVDETELVNDEIVEQAPFTEEVEEQVSVNDEIIEDASVAEAVEESES
ITESVSQEEETEKGFVIEKVEETGAVTEEIVQDGLITEEILEESESVNGEIINKESDA
EEILETEFLTEEVVGQAGSTSEEIVEEEGSVTKEVEEKESVTEELVDEGSVTEELVDE
GSVTE
EVVEQGGSIAQEIVEEESATEEIIRDETNVEEVLEKEGSATEEIVQDGSGTNDFVGKQ
GSVIEEVVEEEISTTEEKLKEEASAIEEFVEEESIREDVLEESLVTENVVGQQESVTE
EIVDGEGSFTEDIVEEEESVTEEIVVDEESVTKEIVEDEELVTEEIVEDEGSFTEEIV
EDEGSFTEEVIEERSLIEEVEDTETVAEKEEGSVIKEIIDEKSLTEKIVEEEKSVTEE
VEEKESVKEEVEEQRLVVEEEGSATEGIVEDRLATEGIVDDILVTEEIVEDGLATDEF
VEQQGSIIEEVLDDEGSVTEEIVEEEGSPNEEIVEGVSVIEEDDNIEPVSEEIVEGSV
TEEMIKEGLENEVILDEDSITEEALEKEGSVSEEIVEEMGSLTEEIVDEERSTSEDMI
EEGSASEEIIQEESQVEEVVEEVSVIDEIVEEDELDTKEVVEEIEFNTEEVVEHKEEE
GSVAEEIVQEEKEGSVNEEIIEEVGSITEEMVEQDVSDNEEIVEERSVIEEAEENVWI
EKEVEEEGLDNEEVIDEEDSVSEQAEEEVYINEEILKESSDVEDVKVENELMNEEVNE
ETQSVAENNEEDKELDNYVVEETESVTEEVVVDEVPNSKEVQEIESIIEEIVEDGLTT
DDLVGQQGSVIEEVVEEVGSDSEEIVEEASITEEVEKKESVTEDILVEESVTGDILVE
GSVTEEVVGEEKLVSEEIVTEEGSVAQEIVEEDAPATEEIDEIESVTEEVVEEEGPVD
EEIVQEEGSVTEEIIQGESKVEEVVEEQGSENEEIFVEEVSASQEIVQNESGTEEILE
KVSASQEIVQDGSVTEQIIEEQKPVTEEVVNEEESITHEIIQEESHVEKVVQQGSVAE
EVVENPVSVTEEIVEKEGSVTEDIGQEGYVAEEIVEEEEFDNEEILEEESVAEEFVEE
GFDNEEIFVEQISDSEIVKEENSVNEEVLEEEGSYTEEILEEEGSYTEEILEEEGSFT

EEILEEEGSYNEEILEEEGSYNEEILEEEGSYNEEILEEEGSYNEEILEEEGSATDYF
VGQGSDNEEIIEEGSATDYFVGQGSDNEEIIEEGSATDYFVGQGSIIEEVLEEEGLDT
EKVFENEGSATEFEETESFTEVVEETESVNENILEETSINEVQKIESITEDIKEQLVP
EEIKEEQLDSEEIKEEQLDSEEIKEEQLVPEEIKEEQLDSEEIKEEQLDSEEIKEEQL
DSEEIKEEQLDSEEIKEEQLVPEEIKEEQLDSEEIKEEQLVPEEIKEEQLDSEEIKEE
QLDSEEIKEEQLDSEEIKEEQLDSEEIKEQKGSVNEEVVEEEGSVTEEIKEQEESVNE
EVLEEVEETESIKEEIVEGGIATQEIIEEESDTKEVVEEEVIDSEKLVDAGSVTGEVM
PEEVSVTDEVVEEGSTTEEVLEEQKSVNEEVVEDGLTIDDFVGLQGSTTEEVVEEDGS
AIEKILEEETATEEIVEKQVSVTEDIVEKEGSVNEEIIEEASVAEEIIQGGSFTEEIV
GQEESATEEVIDEEGLISNEIEEEEEKSVTEEMIEEVEEVSVDDEVEEVSVAEEIVEE
ELVDDEILPEELSATEDVIEEVRSVTDEIVQEESVCEEILEQEVSASEEYVDDKSVTD
DFVGHERSVIQDVENTESVTEEIAEVDKSVIEEAVEKQGSVTEEKVQEGVSAIEEIEE
LESVTEEIAEEDKSVIEEAVEKQGSVTEEIVEEEELDTEEVLEDKSVTGDVVEQEGSG
KDESEAKESFTEEVDELKSVKEEDQETEYISREIEEESATEQHSEQELSINKEVVETE
SLTKDIEEEKSTTQEILEETQSVNEEIVEEERDTDEVLKEKVSPSEEVIEEQASTTEE
FVEERSSTDEIVEVEDLFTEEVKEREGSVTEEIVEEGSDTGEIVEEEGSDTEEILEEG
SFNEEIVEEEGSITEEILQGSVTEEILQGSVTEEFVGQQGSVIEEIVETESAIEERVE
EESATEEVDERESVTEVVEEEVSSSDEVVEGSIEEVIENEGSVTEEILEHEVSADENF
VGQAVSVIEEVEGTESVTEEVVEETESVSEEIVEVSPTENVVQQTDSVIEEVVEQKEG
SFNEEIDIRELGDDGVEEREKISTEEVVGQDKSATGDVEEVSSTEDEEEVSSTEGLEE
VSSTEGLEEVSNTEDVEEVSSTEDVEEGSVAENVKETKSITEEVSVEEDIITDKVSVE
QEVMAEASVEENILTEVPVEEEIMTEKLSVEDKALNEKIMSEEEIVIEDGNVHEVVPA
EVSVTEEIPGVEETTNNESHVKGENVVNEVVVDDNSVNDEIQFDDDSSIEIYTVDSKD
VFHKEENYDSFREEVRSDENIHIFRKKNENFVKKIENEKSSVGHVPTEYTSKENIAEE
VPSHIMFKENVTEEVPKEVKYEENTVEEVFEEVTSKENIIEEAPGDINSEENIIEEVP
EVVTSEEIVVQNDVNNINTNIDHMFDFDLDDILKIPEAQRKLNKLRTLVDVHLDVIEK
MQRDEWKKNKRDFLYICLKEINKLSEDTLMKFYGSDSNKSANDNDTVMVIKILKDKWG
TGRIVDTIANSLNKSYNSLYHNLYIEMEKDLLINKTETFNKWSKQHWNKLDNWKEEKW
FKLFKRDLKIDMRNTYESDQNDEENNEESMNELSDELIKNNTSDNMRNEQKTLEKNKE
YSNLSKDLGLIEKQKIIWKSWIVKNVNNIENWFDEMWFKNVVNELKEKNDVSNVLQEN
ASEASVENYISDVEDSKDMTNSINDSEKSKIVASSSKTTNEEYIILSRKDLIYNIIVM
VHMMVLDQFKYDELKYAKKRFLNRSIDKFIKEKKIKDKETVLDYYIDDIIKRIVEDTS
HVNNIKEKAIDHYTSHDWFRLLRQGNKAQNSISQEVDILTEKYKKLISEEDNKKENNN
NNDDEVKPELKDNIKCEENVSFNILRRSNKKDQLPLEEKKNKNGDLNNTLTESDGKKI
NINEAIEESKNGNEDKNSGNMEKSRKPRDRRTERKEKDQDLRVQLLQDYENIFEQIQN
MENQNKDKNKKETKNILKTSIDLDKNLLREYKKEKNIINQSEKEFSVNEN
>PF14_0358, 184 bases, 2046D0B3 checksum.
MDKSKSSIEKELNRIKQDVSLSAFSILFSEMVQYCLYKSKRGYRIEDCLHEMGLRVGY
KLNEYLTYKNKVKRSINIINILTFISKHVWKYLFQHSSDLLKSQDSIYEYMICDKNIL
LNKFINVPKDYGNINCAAFAAGIVEGFLCSSEFQADVTAHTIHEGDDNYNTTIFIKFY
PEVVEREKNH
>PF10_0159, 824 bases, F57509CD checksum.
MRLSKVSDIKSTGVSNYKNFNSKNSSKYSLMEVSKKNEKKNSLGAFHSKKILLIFGII
YVVLLNAYICGDKYEKAVDYGFRESRILAEGEDTCARKEKTTLRKSKQKTSTRTVATQ
TKKDEENKSVVTEEQKVESDSEKQKRTKKVVKKQINIGDTENQKEGKNVKKVIKKEKK
KEESGKPEENKHANEASKKQEPKASKVSQKPSTSTRSNNEVKIRAASNQETLTSADPE
GQIMREYAADPEYRKHLEIFYKILTNTDPNDEVERRNADNKEDLTSADPEGQIMREYA
SDPEYRKHLEIFYKILTNTDPNDDVERRNADNKEDLTSADPEGQIMREYAADPEYRKH
LEVFHKILTNTDPNDEVERRNADNKEDLTSADPEGQIMREYAADPEYRKHLEVFHKIL
TNTDPNDEVERRNADNKELTSSDPEGQIMREYAADPEYRKHLEVFHKILTNTDPNDEV
ERRNADNKEDLTSADPEGQIMREYAADPEYRKHLEVFHKILTNTDPNDEVERRNADNK

EDLTSADPEGQIMREYAADPEYRKHLEIFHKILTNTDPNDEVERRNADNKEDLTSADP
EGQIMREYAADPEYRKHLEIFYKILTNTDPNDEVERRNADNKEELTSSDPEGQIMREY
AADPEYRKHLEIFHKILTNTDPNDEVERRNADNKEDLTSADPEGQIMREYAADPEYRK
HLEIFYKILTNTDPNDEVERRNADNKEDLTSADPEGQIMREYASDPEYRKHLEIFYKI
LTNTDPNDDVERRNADNKEDLTSADPEGQIMREYAADPEYRKHLEVFHKILTNTDPND
EVERQNADNNEA
>PFE0040c, 1434 bases, 40B01CAC checksum.
MEVICRNLCYDKKNNMMENEGNKVKKVYNNSSLKKYMKFCLCTIICVFLLDIYTNCES
PTYSYSSIKNNNDRYVRILSETEPPMSLEEIMRTFDEDHLYSIRNYIECLRNAPYIDD
PLWGSVVTDKRNNCLQHIKLLEMQESERRKQQEEENAKDIEEIRKKEKEYLMKELEEM
DESDVEKAFRELQFIKLRDRTRPRKHVNVMGESKETDESKETDESKETGESKETGESK
ETGESKETGESKETGESKETGESKETGESKETGESKETGESKETGESKETGESKETGE
SKETGESKETGESKETRIYEETKYNKITSEFRETENVKITEESKDREGNKVSGPYENS
ENSNVTSESEETKKLAEKEENEGEKLGENVNDGASENSEDPKKLTEQEENGTKESSEE
TKDDKPEENEKKADNKKKSKKKKKSFFQMLGCNFLCNKNIETDDEEETLVVKDDAKKK
HKFLREANTEKNDNEKKDKLLGEGDKEDVKEKNDEQKDKVLGEGDKEDVKEKNDEQKD
KVLGEGDKEDVKEKNDGKKDKVIGSEKTQKEIKEKVEKRVKKKCKKKVKKGIKENDTE
GNDKVKGPEIIIEEVKEEIKKQVEDGIKENDTEGNDKVKGPEIITEEVKEEIKKQVEE
GIKENDTEGNDKVKGPEIITEEVKEEIKKQVEEGIKENDTESKDKLIGQEIITEEVKE
GIKENDTENKDKVIGQEIITEEVKEGIKENDTENKDKVIGQEIITEEVKKEIEKQEEK
GNKENILEIKDIVIGQEVIIEEVKKVIKKKVEKGIKENHTESKDKVIGQEIIVEEVKE
EIEKQVEEGIKENDTESKDKVIGQEVIKGDVNEEGPENKDKVTKQEKVKEVKKEVKKK
VKKRVKKRNNKNERKDNVIGKEIMKEDVNEKDTANKDKEIEQEKEKEEVKEKEEVKEK
EEVKEKEEVKEKEEVKEKEEVKEKEEVKEKEEVKEKDTESKDKEIEQEKEKEEVKEVK
EKDTENKDKVIGQEIIIEEIKKEVKKRVKKRNNKNENKDNVIVQEIMNEDVNEKDTAN
KDKVIEQEKEKEEVKEKEEVKEKEEVKEKEEVKEKEEVKEKEEVKEKDTESKDNVIVQ
EIMNEDVNEKDTESKDKMIGKEVIIEEVKEEVKKRVNKEVNKRVNRRNRKNERKDVIE
QEIVSEEVNEKDTKNNDKKIGKRVKKPIDDCKKEREVQEESEEESEEESEEESEEESE
EESEEESEEESEEESEEESEEESEEESEEESEEESEEESEEESEEESDEEKNTS
GLVHRRNCKKEKKYNNGELEEYYKEKQNEEYFDEEYIIQSKEHNTLNTFPNMALNEDF
RREFHNILSIHEDTDLMELKRILYNLFLEYNPHMNNKQKAELDKKFSEMNVVHQILNY
EERIRMYEENAARGRLNTVILDPIITFNVIFGDDTMFKFIDE
>MAL8P1.17, 483 bases, 9A2B6023 checksum.
MNRKYFSSLFLFLISFVFESFVRSHGDLFNHFVTDIHDGELDKFITKNDIVLVMFYAP
WCGHCKRLIPEYNEAANMLNEKKSEIKLVSIDATSENALAQEYGITGYPTLILFNKKN
KINYGGGRTAQSIVDWLLQMTGPVFSHVEGNIEDVLKEKKINVAFYLEYTSEDNDLYK
KFNEVGDKNREIAKYFVKKNDKHNKLFCFRTDEKKVEYDEKTPLEEFVTSESFPLFGE
INTENYRFYAESPKELVWVCATYEQYNEIKEHVRLAAQELRKKTHFVLLNIPEYAEHA
KASLGLTEFPGLAFQSNEGRYLLKNPKESLLNHNAIINFFKDVEAGKIEKSLKSEPIP
EDDKNAPVKIVVGNSFVDVVLKSGKDVLIEIYAPWCGHCKKLEPVYEDLGRKLKKYDS
IIVAKMDGTLNETPIKDFEWSGFPTIFFVKAGSKIPLPYEGERSLKGFVDFLNKHATN
TPISIDGVPEFEDGTSEEL
>gi, 339 bases, 61B6687B checksum.
MVSFSKNKVLSAAVFASVLLLDNNNSAFNNNLCSKNAKGLNLNKRLLHETQAHVDDAH
HAHHVADAHHAHHAADAHHAHHAADAHHAHHAADAHHAHHAADAHHAHHAAYAHHAHH
AADAHRAHHASDAHHAADAHHAAYAHTAHHAADAHHAHHASDAHHAADAHHAAYAHHA
HHAADAHHAHHASDAHHAGDAHHAAYAHHAHHAADAHHAADAHHATDAHHAHHAADAH
HATDAHHAADAHHATDAHHAADAHHAADAHHATDAHHAADAHHATDVHHAADAHHAAD
AHHATDAHHAHHAADAHHAPAHHATDAHHATDAHHAAAHHEAATHCLRH
>gi, 264 bases, 9E5D1B5E checksum.
MVSFSKNKILSAAVFASVLLLDNNNSEFNNNLFSKNAKGLNSNKRLLHESQAHAGDAH

HAHHVADAHHAHHAANAHHAANAHHAANAHHAANAHHAANAHHAANAHHAANAHHAAN
AHHAANAHHAANAHHAANAHHAANAHHAANAHHAANAHHAANAHHAADANHGFHFNLH
DNNSHTLHHAKANACFDDSHHDDAHHDGAHHDDAHHDGAHHDDAHHDGAHHDDAHHDG
AHHDDAHHDGAHHDGAHHDGAHHNATTHHLHH
>PF11_0039, 91 bases, B8652F37 checksum.
MKVTRILYFVFFIFALNFIAPNNEHNGYVEAKKKLTPAEIKKRNQKLMMYSAIASGVA
VLLGASIGLGVHFSKKKSPKKKVIRQVVKKTPA
>PFD1175w, 1222 bases, E51C95A0 checksum.
MNYFSKYKVIESNRLLYTSNVKYDNYFININKKNQVKNKNESYSFIKLLFRKCIIFFI
IYFLFIIPLNEVIYNKYDYSSKSIQHYCIRNLSECFRGKSALNDDSESCRCSNLLKEK
KKTEENSEEIRKYLSDKTKKRNSLYKYKLKGSKGKSVKNIVGNKEGSIHENTDLYSDS
DVEYDYGNLFYDCYYTEGLIDQGEENEEEKFYEAREDFEGEEKNKCKIKESEKSFKKE
NDCKVENVENINYERVYSWELGKEALTKLLGCSNKLSVNNVKYSDWKFKSIPTVGFSK
DGDRLQEMYKASVCSYTEECQGEVYLFVKKIPIEVWIRQYNLMNDNDGEYLLDGENFI
MEAVACAYLSEHYPGLTPKLYKVLYEPECANCNEEDKNMSENNHKKDSKHKGDSNHKS
DSNHKSDSNHKSDSNHKSGSNHKSDCNHKSGSNHKSDSNHQSDCNHMSDHNHKSDNNH
KSDSSHKSDSSHKSDSSHKSGSNHKSDNNHKSDSSHKSGSNHKSDHNHKSDSNHKSDS
NHKNESNHKNESNHKNESNHKNESNHKNESNHKNDSNHKSDSNHMSDHNHKSDNNHKS
DHNHMSDHNHKSDNNHKSDNNHMSDHNHKSDNNHKSDNNHKSDNNHKSDHNHMSDHNH
KSDNNHKSDHNHKSDSNHMSDHNHMSDHNHKSDHNHKSDHNHKSDNNHKSDSNHKSDS
NHKSDHNHKSDSNHMSDHNHMSDHNHKSDHNHKSDNNHKSDSNHKSDSNHKSDHNHKS
DSNHMSDHNHMSDHNHMSDHNHKSDHNHKSDNNHKSDSNHKSDSNHKSDSNHKSDHNH
KSDHKHMSDNNHKSDNNHKSDHNHKSDNNHKSDHNHKSDSNHKSDSNHKSDSNHKSDS
NHKSDNNHKSDHNHNSDSNHMSDHNHKSDHNHKSDHNHKSDNNHKSDNNHKSDHNHKS
DHKKNNNNNKDNKNDDNDDSDASDAVHEDIELLESYSDLNKFNEMLTEQLNKNKDGYV
VLVTELFGEDLFQYINKRNENEDTRVRDEDKKIIMFEFLKLLIKLHNAGLVHLDISPE
NILIENNGELRLCDLAKCAPMYTHNLRHIKGNGNDLYSFQSCQPCVGKIPCIPKECWD
IIREHIKLKIDNPFEHLSTITDQEERKKYYFDVHCVDKYMLGIFYIWIWNLNYIWKRA
DPPNDRTFNNFLKYNLNINVFQLAKQWPKGLKDIINKLLSLESRMKTDLNELTEHPWW
INED
>PFD1170c, 309 bases, E4AA3837 checksum.
MKFFNGSSFSNSNGFMDSKNLKNSAIFSFDEEEKKTASRSFLSSLLSKRMVIPMLGVL
YVVLLNVCLHNGSNTSTNVQFNNNVGKRILSEGQSTDEYSESSSEYVENPVIGSTFML
PNENRLNTIGNLFEGEKYNILEDTQKLIDDINKFRYNSNYSPIPYPEQYNPVDYNKAD
FVLDKYIDNLGYCKGDTIKAMEIIWEATMNNERRKYAATKRSMLRYYDDLKRKRIIRP
KTYTNKLNSCNQIMESSEKKIESHLSEMFNKWFKQKSLFLDDFNRFITACRIGWKTVS
NAVQHQCDTIMRSKNFIDI
>PFE0055c, 413 bases, CFFD1AA4 checksum.
MSILNKYEGKKNKIFLFIINIILFYTLEYVLIGSNYDKHNQSFGNEIFKNTKVFDFTS
LRSLAEFNSGSSRESSKTDETDYYAVLGLTKDCTQDDIKKAYRKLAMKWHPDKHLNDE
DKVEAERKFKLIGEAYEVLSDEEKRKNYDLFGQSGLGGTTTNDEAYYTYSNIDPNELF
SRFFSHDASSFFSQGFDDFPSFQGFASMNSRRPRSSRSNIFSRSFGRAASFEVPLQVT
LEELYTGCRKKLKVTRKRFVGLNSYEDNTFITVDVKPGWSEGTKINFHGEGEQSSPNE
QPGDLVFIIKTKPHDRFIREGNNLIYKCYLPLDKALTGFQFSIKSLDNRDINVRVDDI
INPNSKKIITNEGMPYSKSPSVKGDLFIEFDIVFPKKLSPEQKRTLKETLENTEKSYT
FFIMNVK
>PFI1780w, 383 bases, 6E404AB1 checksum.
MAVSTYNNTRRNGLRYVLKRRTILSVFAVICMLSLNLSIFENNNNNYGFHCNKRHFKS
LAEASPEEHNNLRSHSTSDPKKNEEKSLSDEINKCDMKKYTAEEINEMINSSNEFINR
NDMNIIFSYVHESEREKFKKVEENIFKFIQSIVETYKIPDEYKMRKFKFAHFEMQGYA
LKQEKFLLEYAFLSLNGKLCERKKFKEVLEYVKREWIEFRKSMFDVWKEKLASEFREH

GEMLNQKRKLKQHELDRRAQREKMLEEHSRGIFAKGYLGEVESETIKKKTEHHENVNE
DNVEKPKLQQHKVQPPKVQQQKVQPPKSQQQKVQPPKSQQQKVQPPKVQQQKVQPPKV
QKPKLQNQKGQKQVSPKAKGNNQAKPTKGNKLKKN
>PFE0360c, 893 bases, CD113355 checksum.
MIYLGKKLLSCTLFVYFLYIHFFLLKQNNFCDVKVRERILEESINNDLSSKGENLHIY
EKTNVSVQTFIKKKERKNLSDNNINDKINNNNNYNNNNNNNIEDTTYYPTGNEKKENI
FLKIFRYVKNWFPIKSSNNLKKTNINYEQVQEENEFSKYILQNNMSIETTKVCLIGSG
GDSSHDLIKQFLLHNNVKYNRNYENDNSLNNKSGSIISSYKYYSEEIIQEPYIIDRDI
CNKKKNCKESTLNYENNPKTLIGNIIIQSDILKNEKIFNMNRHFVVCKYFGSIPKTNI
KINQSTLIQHLIKCLDYCKMEGVQYIYIGYNIYAANNKLIEIMKKLREHKIIIVTSSG
KIYDDDNNNDNNNFYNDNIYNNNIYNVHNDDKEKIKNHIKKKNKQNNYLYEYQRTQKD
EEQKSNSSLYQNLENVISISGLIYTDSSKKNKNKNYIYDNEIKILDQKGNKKLNRNYI
SLFYFSYDTDIYEKIESDIIDDDHDLVSASFVNTLVLMHSINLKLSLGRLRKILNKSI
VKREELRHLSNRAYYHDMMNTFEDSLNQRKRSYKIFYLELKNNKHKVLLSDANLKSMY
QDNLPVNYNEEDHVKHNVEQETSVERDIYKNNENSNKNRKMDMDEGKGTYIQNKESHK
YNIHYPYNRIKQSLLNDNTLNHKPYVSFLNMSYYNEDIEKRNYNIYDDPSYTYDQGIT
YDDNYYIDDHDIHTRKKRKISYDGEDNNDYHMYDDRDNLFHSNLGNNKYEDDGNVHRE
KEKDLEPRFLYDPFANIENRDLETVQELSELREKKSNNFYSRNHDNSSNMKRRRKEKK
KKLKKVLRSKYDKIGNLERIRRKKKRRMIHKNKINKRRNMKRKNNELEERRNKQADKN
SSSGNGKGKINGTRNSPKIKFKR
>PFB0100c, 654 bases, C155B129 checksum.
MKSFKNKNTLRRKKAFPVFTKILLVSFLVWVLKCSNNCNNGNGSGDSFDFRNKRTLAQ
KQHEHHHHHHHQHQHQHQAPHQAHHHHHHGEVNHQAPQVHQQVHGQDQAHHHHHHHHH
QLQPQQPQGTVANPPSNEPVVKTQVFREARPGGGFKAYEEKYESKHYKLKENVVDGKK
DCDEKYEAANYAFSEECPYTVNDYSQENGPNIFALRKRFPLGMNDEDEEGKEALAIKD
KLPGGLDEYQNQLYGICNETCTTCGPAAIDYVPADAPNGYAYGGSAHDGSHGNLRGHD
NKGSEGYGYEAPYNPGFNGAPGSNGMQNYVPPHGAGYSAPYGVPHGAAHGSRYSSFSS
VNKYGKHGDEKHHSSKKHEGNDGEGEKKKKSKKHKDHDGEKKKSKKHKDNEDAESVKS
KKHKSHDCEKKKSKKHKDNEDAESVKSKKSVKEKGEKHNGKKPCSKKTNEENKNKEKT
NNSKSDGSKAHEKKENETKNTAGENKKVDSTSADNKSTNAATPGAKDKTQGGKTDKTG
ASTNAATNKGQCAAEGATKGATKEASTSKEATKEASTSKEATKEASTSKEATKEASTS
KGATKEASTTEGATKGASTTAGSTTGATTGANAVQSKDETADKNAANNGEQVMSRGQA
QLQEAGKKKKKRGCCG
>PF10_0321, 561 bases, A10BE752 checksum.
MLKHVFFCYRWRAEYGWVKSRLNFLCGASCYSSNINMDKIIDENKYEYIKNLRSQELR
ILSENCCVKKVDDVIIWSEICRHSIEKYNSFKYFDALLLLSSFDKMNIVDKSLYKTFS
DVFIKQISYLKPEHFILLINLYCRVNIFPRVLFTEIFHGIIKYCNKLYPDEYVNLLTC
FANLKIANKDLIKTLCKSIIKNINLFDYIHLTNIVGALRSLEITDDIFYYVIDQKQLK
ELKFLTVQEIFDFIKKIKLLQYSWKLYEQDLMKEFLFKVYNFKNEKDVDQLDDPFVCL
NFLVSKGLLQGNNNTNIKKKNNNKKNNNNKKNNNNKKNNNNNNNDNNNDNNNNDNNNNN
DNNNNNDNNNNNSSSDFIDDINCGGANFLVALSKWCANQVYHYPSRSTKRPTSYQLIK
LYELMKEFNIHNSDFIEKAIYRFVITRGGLENNRDKMFKPTSYQKGRKYIFTKDPQID
HINYEKNKEHSYSHHYNLKDHTTDYEQQNMHYQTYDYNDEHNMNQQKTLSEKHKRIKL
SMEKKTQNKKETHSNSRYCNFKLRQRPKRIKNSPAPIKV
>PF14_0607, 1068 bases, F6C99CD9 checksum.
MLMLYLHILLCIFLNNPVLVTNENNFFIKILRSQEKKIENEVSLYEKLNNRILFEGSD
DFSNLDKLKSLLSFQKKIDNTIRKKQKEENKLKKFQQAIKNGNNIYPVGNNPQGFEKK
EREEKEQEKTYMNNVINKINEQKGNYYEPNKNINNKYTEDLTSNGLLYNKDKIKNQFN
NPHQEKFIRKHEHEREHEGKNEGKNEHKNEHKNEHKNEHVHVHEHNSKNYEGNPPKNE
YPNINFTNILNSIDFYKKIDLVKNYFGYNNNETLNQNMKEQKNMQNHHVENEKNNMIQ
ANNQVDENQNNHQYNNNFHNQKNDNLKQDTKDNSSEQTYNNSNNNIEDNNHIKFFISK

DIEKKKNKQNDINESVYNAIKNKNLHLRRNNIKEIFDVDDLVKNIKSFLGIKSNIHEA
FENQKLIIKNCNYESFGPELCSVDEKAKEMLWKYEKKKNSAFLFIILFTLFFSLIIQN
IVYFIEKKVRNSKDQFRKDLLNTAFRQISLITIINLTIWGILQTNIAEALDEVIFNDI
LPRHRNIDGVLHNVEPLLEVIFEKILFISMNFLICYSIFIVSIHFVTRTILKWFSESD
NSDVSSVAKEVKESQRKCFRNYFFFFRNVRNSKYLAHRYDFSENVDAISIPGLDPNGY
YYYEYMRASLLKYNVKLIKIPNAVILFLIFVCISLRPFYNIRLKAEVIFLNVLSLICI
IGLISLFVYLYRIDTKLLPRDISKYLLNKYHIETCDKNKRDVTPYYKLLKQESVYPSA
LNYFFYKTTFPNKHEQLFLLWGNGPSLINFIFQTLCFCFLIILSCWIFLLRVDNITWF
QLYSYGSLSICVCILVFFFILKYIIYYNVMVTKTGYLIDTKLLEQVWEYERSDNIKRI
SEFIDAIKIKSTLHALKEGGEIFWRQLLIKSSTVPSNIQEKMFSIWIGLDEENRGIID
SSKILKFLKSQGINLTSEHDIREFLEVFDRNNKNGLNQEEFFVLIIIVKQILVELLDI
NAVQSLFEEVYGIPWKSLSSIDVNSLKKILTELNLKWPHGKIRNLIDFVCENKKTKYV
SAEYFIKQLINIEEVTLQPFHVSS
>PFE0355c, 769 bases, DD515F98 checksum.
MINRQYFIWYIFIFNIINKIYFENIRYVKNYEVVIRKKKNIERGIGNDFAFIRRYYKS
RLLSDVSYKNNSIKGKNRVDKEGDIKKYDNNDDNKMDNSYDYKNKSIKENETKIRKEQ
VISLDKRYNRNINEKEEIKKKIKDIQRKRLIIYFKQDNTILSSRNYKHIFMKVLSSCG
HIEKLTFINFYLYEFPKSINNEDMLLKICLRLLESRRINVENDNQISHTVQMKSYNNN
NNKWDNINSKNNCIYQIKDKIKDLPNVSPSASTFTSISTSPYTLKLRDRNKYANDKNH
IFKINHSNKHKNNNNNNNNNDYHNNNKSNYHSHSSAKCQTQRLNKKMIGTNILDGYDI
IQMEEGLNLSHNYELNDVNVCIIDTGIDENHIDLKDNIIEKKTFMKHSYKKYNIDGIN
NIESDNIDGINNIESDNIDGINNIESDNIDGINNIESDNIDGINNIESDNIDGINNIK
SSDNIKSSDNIKSSDNINSSDNIKSSDNNNVHTMLRNKLYLKKKKECSNYNTSNDGHG
HGTFIAGIIAGNSPKGKKGIKGISKKAKLIICKALNNNNAGYISDILECFNFCAKKKA
RIINASFASTTHYPSLFQALKELQDKDILVISSSGNCSSNSKCKQAFQECNLNIQKLY
PAAYSADLNNIISVSNIIQQSNGNIVLSPDSCYSPNYVHLAAPGGNIISTFPNNKYAI
SSGTSFSASVITGLASLVLSINSNLTSQQVIELFKKSIVQTKSLENKVKWGGFINVYD
LVRFTIDSLPKDKDE
>PFI1735c, 713 bases, B0829D2D checksum.
MADYSSNEEETPKEEKKISKLEDMQSPFDYKRFFRKYTIFAPFILVYFTLMFFVNSTV
QNGTMLLNSIKENANSKLPALLWNKIIGKGNDNEVNFEDKKMIEGNKSGNGEEHNKHK
RRQVDNQEDDYDVDEYGNPKSGAPYKSEEEHAEQQNYYGEHHGDYEDKNEYHGKSNIE
NNKDFPEQGQKDEEKEKEAKKKAEEFEKRKQLHEEEKRKARETQQALHKKLQEQLQRL
KEQEKKKVEHQKLIHKIKTQGDVDPAVQKVLNKYNQKEKDEEQQILKIRDLQVQLRHI
QQRIQSLKSTNNGQPKTKDEYLQLKDLQKEYYDKEQDEQNMIEHLEHLQGEMQYLYEE
LQIVYDENQAKTAQAKDEQHELKPQAEKDASKLTTTYDQTKEVKPQAEKDASKLTTTY
DQTKEVKPQAEKDASKLTTTYDQTKEVKPQAEKDALAKTENQNGELLPQAEKDASKLT
TTYDQTKEVKPQAEKDALAKTENQNGELLPQAEKDASKLTTTYDQTKEVKPQAEKDAL
AKTENQNGELLPQAEKDASKLTTTYDQTKEVKPQAEKDALAKTENQSGELLTQAEKDA
LAKKESEAPKGSSTSDKNTTENQLNAEAQSEVKPQWQKDYWKSKNESQTETNNTWKKD
SWKKKTEEQQNAEAQQEVKSPYEKYSYNKETKPQNDKYTLNKEVKPQNEKYTYNQKVK
AENTESEKSLLESSVFN
>PFI1740c, 119 bases, EE3E66E0 checksum.
MKMYLAEIFSSGKESLLSLKDTLGSSNFSPLKPCEGLECLPQVLFLYVIFLLLCTGIF
MHNKNKEHHHEKSHNNGQNSNVPKNSFQNNKSQNHVQKSKVENQKVPVAQGSQHKSAA
VNA
>PFI1755c, 326 bases, 3375A313 checksum.
MQTRKYNKMLSKVETKQFIYILFFLCLYLNTFNYKYTTSYEGSSFRQLSEPVVEEQDL
KKTNAESSHIEATTSQATTSQATTSQATTSQATTSQATTSQATSSQESDEQGLTAPSL
NLEETQSNKVRNKIFNFPIPSAEGTVSKEFKNQPKTEYEKKLFEEWQHLNMFEHSNWV
NITVQSCQVLVQGLTSLDDYDAKFKSWSAMVELLGEFRITLFNESNNMFEALLNELRE

ARKENPNENLTPEEEEKWDLIKQTKLEKDIEWKIYQILTWKYWNLKEFPGVDIPDPSV
PSLDFDATYDVLGDILEDDEDEEDDEDTAKPSTSSS
>PFI1760w, 214 bases, 2D32C81E checksum.
MGNFFIISYNKHCIGQIIHMLYFICLIFILYNRNTCLRRIAKCGRNLSELNLDLRTDY
DEREININKNIPSYPVKFSKLEYKLRNEWDELEIDEHDDYMDVTVECFEKLLENDKNL
ENYQENYEIRALVAYMLHELRRGYINEQKMKFKNFLHKLKEKRINEPIDTLNNDEQDE
WNKIKNGKIKSDEEWKSYQLQTWEYLIKMEYYKNKYSQNV
>PFC0435w, 1294 bases, 60D44EDC checksum.
MKVGIIFFCLFFFVVLGACNNVKERIFKNIKKRTKFIILNEPIVDLSFSENLFHTLLF
DLDVDKNLYTLDESLLNLENLNYSSIFRLLVDTYKNIKENEDDNKNIRYIFLGTSFSR
IHPLNFEYFLRKLNKYIYNGNIYEKGNVDIRGILEEYNKEIEEKKLEKQKLNKIKDKN
NNNNNNNNSKFSKDGDNEDFNNKNDLYNPSDKLYNNNDDIDVHELLEEIITKEKRFFL
NDDDDNDSNDKYILKTDEVNKYKGFFIGYGFNDDIPSVIHHYNFDKNFLFPSLNSGII
LDITLLKNIYEVSNILLSNNEKDQSIHIDYIYEVTKYIKENLRVRLTHSENVCLNEEQ
NIHLLDNDPNNFEIYKYYQVLNLFKDYNKNTEEKQYEKIGHENVRHEETSSEGNENLN
RNTKHNNDNNNDNNNYSEDAIAELLLSYFNVFYPISTCMCYSIRSKHESLMDYDKYHM
INLENDIKLKHYIKETEEIHFNSIEEYKMKLNRINYKYDTLLEEHENLVTHKNILIGI
KTSINTEEERIPHIKNTYDNKENTQIIFNTFNYDNKLKEKNTFGFYNNSLLQNALEND
NIDLDIIYMSDKESQKYDNLYFNSKVTSKEGLCEKLKHMIYYYYEEYVMKNSEKKYFF
IADDDTFVNVKNLIDVTNLTLNTCSHSKKYMYDKYIKSYDFVKENEALFLQNFPKKTL
FLYSYLKDTFAKTIQTLKKYDYVPKYCQGGILSKKHKNNDSDDDHDHHVGNKQNNDST
NHQDIEKNQVNVINNNNNNNNNKAKSIPIYLGRRYSYNTFSTNSNEYFYDYLTGGAGI
LINDETAKRIYECKECTCPSTNSSMDDMIFGKWAKELGILAINFEGYFQNSPLDYNKK
YINTLVPITYHRLNKNRTTKESRDMYFNYLVNYNRNDKEQNKDIYVDYLDRNHKNMID
NVFHYFFYVNMYDEKNKVVTKIEHNADMNSKKNKSKNPQKLNNTQGDKNVNDDENVND
DENVKGDENVKGDENVKGDEYMKGDENVKGDENVKDDENVKDDENIKGDDNNYNVDNM
ENIDDIINMVESVDDDVMERNKKGTGKEKKDDKNHNNKEKATDVKKSSVPTNNIDKNE
DTTKYVIKMNEKIYNRMQESGKYKQLFDINKFFKKEIEGHPYFQKIKKKNEKAKKEKE
KMNQLKKQKDYTNNYFHTSNMQGNFNQQKMGNYQNQENEENDFFDQRPEIEEDAINPM
DYEEYMENLSNFEDDGEPYDEYDDYDDFVNTINADKLKINDQNKHLYEQIKDIAQPPV
NFQNDQNSNTFDFDTDEL
>PFE0060w, 408 bases, 737D8C83 checksum.
MLLFFAKLVVFTFFFWLLKYGKTRSYPKSGHKGHTKLNQPVVRTLADFNDMFANQKNT
FNFLKHINHYKNEQDTNNTHTPNHDEYSHNLPKNHEESNANMNNHNSFNDKSVNKKEA
FDQFLQTLLNNYEIMHKEDESKESNQHNYKEGPSYEDKKNMYKEILKGYYNVFFENYA
NDTESNVHNKPEEVHKHEEIHKHRKLHKHEEVHKPEEFHKPEEFHKHEKVHKHEEVHK
PEEVHKHEENHKHEENHKPQMVGQAPPEKEIRQESRTLILGSFPQAGEILREDLWNKE
DNKFSYALDPNDYASIEDKLLGSIFGYFKKNHDNLVKHLLQQINTYKHKYMELKEQYI
NEVMKLKKIYNKSIMVIFIASCISILGPVMLHMHQNNPEEFFATILSFSISLGLHNLL
LT
>PFF0845c, 2394 bases, 36DC08B0 checksum.
MGPAPRTATTTYSSAKDLLEDIGESVQKEAKKQALGRSESVLHGLLSNATIKGVKNKA
TKPIQLEYEYHTNVTGGFDKNNPCANRLDVRFSDIYGGQCTDNKINGNDDETGGTCAP
LRRLFLCDQHLSHMKEGNINNTDNLLLEVSLAAKYEGDSIINNYPDNRDKKEGICTAL
ARSFADIGDIIRGKDLFLGYTKKDEKEKEKVQKNLKRIFNEIYKKMQDPAKSHYSGDS
SDFYKLREDWWALNRKEVWKAITCKAKNDAEYFRKKDSDGKHCSVQNCKCVDGDPPTN
LDYVPQHLRWFDEWSEEFCRKRKDQLKISLEKCRGKNKDKYCSLNGCNCKTTVRGKKK
FDYQQECNDCLVACDPFVHWIDNEKLEFLKQKKKYKNEIKEREPTKKTSHGTINNMYA
KEFYETLEKEHRTVDTFLKLLNEEKECKNHPEVGEGKKTFIDFNDNIETFSHTKICEP
CPWCGVKPGGPPWKDNDIDSCGKKEISFSDKDTTDISILSTDRAKKNILQKLENFCRD
TEHINHDIWKCHYENTDNDNCILQNENTGSEKQKIMPFDAFFFLWLTQMLDDSIEWRK

KLKTCINNEKPTNCIRGCKKPCECFERWVEQKEEEWISIEKHFDKQRDISEEERYITL
EYILNEFFMDKIEKAYGIEKSKELKEKLKSNKGHGIIRDTEHSQDAIKILLEHELEDA
KKCTETHNDEKCKEQEESGGRSLNPDPESDDEEETDNVKENPCAVGKKLTKTVKQIAR
QMHQAAKKQLGSSSSRALKAHAHLGTYKHTNKRDDFKTICKITKDHSNATHNYPQGPC
HGKNNDKSMFKTEEGWKPGNQINMNDEYAFMPPRRQHFCTSNLEYLETADRTLNGIGD
DPNVLNHSFLGDVLLAAKFEADFIKEKYNEQSNYKDFSTICRAMKYSFADLGDIIKGT
DLWDKNGGEQKTQGKLEKIFCKIKNKLPEDIQEKYINDDKNSPQYKKLREDWWEANRK
EVWRAMTCATTSGKIPCSIVTPLDDYIPQRLRWMTEWSEWFCKEQSKLYGELVKDCAS
CKKKGKEKCTQGDNDCTPCDKKCKEYGKKIRTWKDQWTKMDGIYQMLYLQAQTTARNA
GDTAFDNPNDQYVIDFFKKLQKANGDNNFGVNTSPYFTPAGYIHQEARVGECEVQKHF
CNNNGNQDKYSFRNQPYDHEEACACKKNTKAPEKKKEETPPAGPNVCEIVDKLFEDTT
TPHAACQQKYINGHEKFPNWKCVTPSGEKSGDKGAICVPPRRRRLYIHDLQSLDEKPS
DTALRDWFVKSAAVETFFLWHRYKKQKEKKPQEGSLLSGSTLLSQFSIDLGDEEQPPE
KQLAGGKIPDDFLRQMFYTLGDYRDICIGDENVIKTLKYSGDKDDIMEKIQEKIKAVF
PTSGGTSHIPDVKPGGALSRGDWWEQHGKDIWHGMICALTYKETSGSGEKGEKTTITQ
DGTLKDALLDTDGKKPKRDYQYTSVTLKEDDSGEKAANVPAKVEPTTLADFTRRPAYF
RWLEEWGDGFCRERKKRLAQIKKDCYEDGGTGEKQYSGYGEACDRTNTSNEGASADLE
GPSCANSCSSYRKWIERKKYEFKKQEKAYGGQKQNCKKERKAAESNDNDKQFCGTPET
TCNTAEAFLQNLGSCKKYNGEGKKIFENTEETFKPAIDCEPCSEFKVKLEKCNCGSDA
KGNTCTTGKITAENFENKTDVNEVVMRVSDNAESGFKGDLKSSCENAHIFEGIKENKW
KCRNVCGYIVCKLEEVNGEKDNGKKILLIRALVTHWVDNFLQDYNKIKKKLNTCMNSS
DATPCIKGCVDKWIKLKKDEWEEIKKPYLEQYKNGYGENYNVKTILEKFQDQPEFKKA
IGPCPTLDAFEKSKQCNATASSEKGKDGNKSYVIDCLLQELEKLQEKAKKCHDQHSDN
PQEKCDDPHPDEPDEEDLLLEEEENTANSAPEICKDVIKAPPKQEEKGGCEPASPLEP
EEVEEETASVPPGSEPEADKGPVKPAELPKPPKRNKRQPKKLYFPTPALQNAMLSNTI
MWTIGIGFAAISYFFLKKKTKSTIDLLRVINIPKGDYGIPTMKSKNRYVPYKSGQYNG
KKYIYMEGDESDDYTYIGDISSSDITSSESEYEDIDINNIYPYKSPKYKTLIDVVLEP
SKRDTFNTQSDIPSDTSTNKFTDNEWNQLKQDFISNISQNSQMDLPKNNISGNIQMDT
HPHVNILDDSMQEKPFITSIHDRDLHNGEEVTYNINLDDHKNMNFSTNHDNIPPKNDQ
NDLYTGIDLINDSISGNHNVNIYDELLKRKENELFGTNHTKHTTTNIVAKQTHNDPIV
NQINLFHKWLDRHRNMCEQWDKNKKEELLDKLNEEWNKENKNNSNVTDTNGENNITRV
LNSDVSIQIDMNSKPI
>MAL13P1.413, 249 bases, E8F5558E checksum.
MAEQAAVQPESVPTVGTVPQADVPTEGMDVPFGFFDKNTLKKLMFIFMRDVDNYARNW
FTNFMHAQTEDDDQTDGEGKHAYLLNHKRTWFEQFKASLSEALDGKNSVFLLLFLFFG
FVFCLLYHAFLYHSIKSEHKAKKLHLEQEENDDDYHHYHHAPHFYPFFDPEYMHDHDH
DHEHDHTIKPAHAHELDHGHDHGHDHGHDHGHDHGHDHGHGHGGHVCTCKNKAKKKPG
EPCDCQKAKLEQEKKDN
>MAL13P1.356, 2223 bases, FE89FDB8 checksum.
MAPQGGSGDPQDDDAKNMFDRIGKDVYDEVHGEAKKYKEALKGKLQEAVSTSPELVAF
TDPCELVKQYYNNHVNGKSNRYPCGNVKNAKNEKVKRFSDTLGGQCTDQQIEGNDRKN
GGACAPYRRLYLCDKNLETISNYNSNARHKLLAEVCYAAKEEGDLIKTHYTEHKLTNL
DTKSQLCTALARSFADIGDIVRGKDLFLGNSVESAQRIILENNLKTIFQQIHSEVTTT
NGEAAEARYNDTKNYYQLREDWWDANRETVWKAITCNAWGNTYFRTTCSDGKSQSQAN
KYCRCDGDQPGHDKSKAGKANDDVNIVPTYFDYVPQYLRWFEEWAEDFCRKKKKKLKD
VKRNCRDETVKYCSGNGYDCTKTIYKKGKLVIGSECTKCSVWCRLYEKWIDNQKKEFL
KQRNKYETEISNSGSCGGSGGVKGSSRKTRAATIKYEGYESKFYKKLKEKNNYGTVDD
FLKLLNKENECKGINEQEEIIDFSNKCDYRFDKNINNKGTFYHSEYCQPCPGCGVKRK
DNQWKEKKNGDCDSDKHYKIEDGAKPIDINVLSFGDKGNEIKSKIDKFCLTQNGSAGG
GGSGNASGSSGDCGGGNSDSSLCEPWKCYEAQYVKEDKKEDEEDEDEVKEEDYLKDAG
GLCILKNKNKEEKKEKEKKSEEEPAEFQKTFNDFFYFWIRRFLNDSMYWRGKVERCLK

NKSEKCKSGCNKDCDCFKKWIGKKKEEWDAIKKHFKTQEAFKNGGENGVIDMFGEAFR
SADFVLELALELEQLFQDIKEGYGDVKELKGIENMLEKEKKKNEEETTVGNDSQKKTT
IDKLLQHEGEEAKDCLEKQNDCNKQSPPTGGPGGAGGRSQTPHAGGATAPKKGDSEGE
DEDEVEDGEESESEEKEKEEKEKEEDRKVKEEVKETKVDGESPKETTKEVNPCQIVDD
LFKGTNKFSDACDLKYNKGKNYGWRCIPTGNTSNDNKGENSGNGALLQRSKRHTSESS
ADSAPSGDTTGGSICVPPRRRKLYLHKVDDGEFDDDKSLRDWFVKSAAVETFFLWDRY
KKENKTQNTSQLLPITAPVSNSDDPQSKLQKSGEIPPDFLRQMFYTLGDYRDILYSGD
TVNGGKENKIKTAIDNHFQKIREQSSSDNNLSPPHGTPGQPNSVKTPQQTWWKKYGED
IWNGMICALTYDTNTASGKTPTQIPEVKEKLWDSGKPQKNYQYSEVKLSDNDGDGTSA
PVAQHASRDAPHTSTANGSISLADFTSRPTYFRYLEEWGETFCRQRTRMLAKIRGECV
KSDGGRCSGDGLKCNEIVIDKEKIFGDFLCPTCAGHCRFYKKWIKIKKEEFTKQKERY
ETESDNAKSKSDDTSENEFVKKLEEYKSIDLFLGKLKAGPCSKTNNGDDDINFKENDS
KTFKHTNLCDPCSEFKVKCNGDGCRGGANGNTCNKTTFKVPGDIGNKENLTEKVDMYV
SDKYAKGFLQDLNDCNDAGIFKSIRKDVWKCGNVCGLDICSLKKINNNGQESDEHILI
KELIKRWLEYFFQDYNRIQKKLKPCMNDGNESSCRNKCKKKCDCVGKWVEEKKNEWEK
IKEHYVDKYNYKDTNNLNSFLETLIPQIPVVTDKGKHDSLDKLKTSLKCNCHGRSKKE
NDKNNDVIDCMIKKLQEKAKKCHDQHSDNPQEKCVDSTPLEDDEEDLLLEETENPVDP
PKICGDMPTQPETKEEEVEKCEEASPAPKAPEPTQPADGGEQTPVLKPEEEAPPPAQA
PDVAPPARAPADQPFDPTILQTTIPFGIALALGSIAFLFLKKKTKSTIDLLRVINIPK
SDYDIPTKLSPNRYIPYTSGKYRGKRYIYLEGDSGTDSGYTDHYSDITSSSESEYEEL
DINDIYVPHAPKYKTLIEVVLEPSGNNTTASGKNTPTSDTPSDTQNDIQNDGIPSSKI
TDNEWNTLKDDFISQYIQSEQPKDVPNDYSSGDIPFNTQPNTLYIDNNQEKPFIMSIH
DRNLYTGEEYSYNVNMVNSMDDTKYVSNNVYSGIDLINDSLNSGNQPIDIYDEVLKRK
ENELFGTNHVKQTSIHSVAKPARDDPLHNQLELFHKWLDRHRDMCEKWNNKEELLDKL
KEEWENETHSGNTHPSDSNKTLNTDVSIQIDMNNPKTTNEFTYVDSKPNQVDDTYVDS
NPDNSSMDTILDDLDKPFNEPYYYDMYDDDIYYDVNDHDASTVDTNAMDVPSKVQIEM
DVNTKLVKEKYPIADVWDI
>PFF1580c, 3954 bases, 4E8196CB checksum.
MGPPGGSGGSTLDESVKDLFDRIGKEVHDQVEKEANQYKEALKGDLSSATFPTGRRHE
KPQSESCKLNYIYDTNVTSGGGKENPCYGRQGVRFSDTKGAKCYSYKIEDNDSSIGFC
APYRRLHLCVQNLEQIKPDQITSTHNLLVDVLLAAKYEGQSITQDYPKYQATYDDSPS
KMCTMLARSFADIGDIVRGKDLFLGTNEEKKPLEENLKEIFKKIYENLGIQEKNHYND
TPDYYKLREDWWIANRDQVWKAITCNAGGYSYFRKTCGGDNEKNSTLASNKCRCKDEK
GEHDTDQVPTYFDYVPQYLRWFEEWAEDFCRKKKIKVENVKKSCRGEDKESKDRYCSR
NGYDCEKTKRAIGKLRYGKQCISCLYGCNPYVDWIEKQKEQFDKQKKIYDKEIKKYEN
GASSSSRKTRAASTTNYEEYEKKFYEELNKSEYSDVNAFLEKLSKEDVCTKVQDDKGG
RISFENVKSSSASGGAAGTSSTSGGGTSGDSGTNNENEGTFYRSKYCQPCPPCGVERN
VNDWKEKHKIQECKSINLYKPNEYAKPTNITILKSGEGHEDIETKLKAFCDKKDGTGS
GDCGGNSDPSLCEPWQCYEEKDIEKHGDVDDDDADGNPLNAGGLCILKNEKHVSETNS
QIEPDQFQKTFHDFFYYWVAHMLKDSIHWKKKLQRCLEKKNGNTCKKNNCKDNCGCFE
KWVAQKKEQEWDPIKQQFRKQDFGKQELILGQIRYDYVLDELLKKDELLTSIKEGYGD
ANEIKHIKALLEDEENEETQEEETAGADNQKKNTIDWLIQHEEDDAELCLEIHEDEEE
GGGNDECDDDHEEEVYVSNPCATPSGSYPSLANKVAQLMHHKAKTQLAIRAGRSLLRA
NASKGEYKHEGNPDDFKKEKLCEITAKHSNDSRRDGEPCKGKDGNNERFKIGTEWKIG
EKVETSYKDVFLPPRRQHMCTSNLENLDVDSVTENDKASHSLLGDVQLAAKTDAAEII
KRYKDQNNIQLTDPIQQKDQEAMCRAIRYSFADLGDIIRGRDMWNKDSGSTEMEKHLI
SIFEKINEKLPEKEQKKYSNDGKYLDLRKDWWEANRYKVWKAMKCATKNSKIPCSGIP
IEDYIPQRLRWMTEWAEWFCKEQSQAYETLQDQCGKCTGPNKDNCTRDNNDCNTCTKA
CEEYEQKIKKWADQWKVISKKYEELYLQAKTAFARTAFPDDDPDYQQVVEFFKELQKE
INRSASQRSKRSIDVTNTDPTLTSPYSSAAGYIHQEIGNVGCNVQTQFCEKKNGVIPT
TGSGTNNKNYAFKNTPKDHDEACECESRPQVPPKKKEDEEDVCEKVKGLLKGKDGETT

EINGCKPKGNGNTWNCSNQIDDSHKGACMPPRRQSLCIHNLKESNETGTEQQLREAFI
NCAAIETFWLWYKYKKDKNVDAEKKLEQGEIPSEFLRSMIYTFGDYRDLCLNTDISAK
KDPIKIVKNNIQKVFNRNNGPHKEGDKEKRKKFWETNKNDIWQGMLCALEKIANNKKT
LTETYNYSLVKFSGNNSTTLEDFAKTPQFLRWFTEWGDDFCQQQKEQLVTLQEACPNG
ICEQSEEEKKKCKSACEKYQAFIEKWKGYYDKQSKKYFYDISTGMYKDNSSAKDDVTV
SSYAYEYLNKSLKKLCPDGSCSCMEQRSQQHNEDSSDALETHNSIMPQSLDKEPKEVK
GRCKCPDPPKPCDIVKDLFENKDDNKFTEACSTKYKNGKEKYTQWKCINDSSNTTRSS
PPAPVASSTLSTSPEAGASATCIPPRRQQMYIQPLQSLSGNESQVELRRIFIEMAAIE
TFFQWHKFKKEKVREIKEKDEIDGKISLFGQDDTSIEENLQKQLKNGTIPDDFKRQMF
YTFGDYKDILFGKYIGIDMGTVKTNIGRVFNNGGNKSDEEREKWWKNHGPSIWNGMLC
GLSYDTKTKHNIQGVYTQLTDPSKNNMYEKVTFPSKTGPSVNTKLEQFARRPTYFRWL
EEWGEEFCKKRTDKLKKLEKECRGVNYSGYNKYCSGDGYHCDDEKGTYNSINANLNCR
DCMKECRNYKTWIVKKKNEYDKQKSKYVNEHENVISFLNKQSYKQLYENIKPYSSAAD
FFTSLNHCKPDKANDDKNNKLNFKNPHETFSPSTYCKACPLNGVICRGRSQCAANSEN
NLTNLGESTDFDILINDAAIHDNDNEIKKGCPTYEMYKDLRKQKWICQKKTGEVHQCK
LNNAADSKYYDNKFPFNILFHRWITDFIQYYNKSKEKIKPCTNDVNSCKQGCKGNCDC
VDKWLKNKSTEWEIIKKYYKENFGNTNEHIAYAIKIFLQEGLFDSDYKRAQEVIDQNE
WEQLWGCTGDNLKDVKDQKAENCNKGDFITNLISKLQDKITSCQNKHNPNGKTACDEI
PPHSDEEETSLLDDDTSTQEKMSPDFCPSDMPEKPKTDSDILCDDKKEPKCGNFRTLF
KTSTSKTKTNLIGLEAHNHRAGRFYPNVYISPRAHQLYLEPLKDLKENNTDKNELIKA
FTKCAYNEGKCLYEYYSKNKATLGKNGSALSNDEVKTYTLEAMERSYADYGTIVKEDI
LWDYEDKKKIDPKIMNFAKNHNISTTKTIVSSLDDDDVKRQKLWESIRIDVWKAMICG
YKDAIGGDMNSLPNDVDLCTLPTTDDEYSFLRWFVEWGQNFCIRHEKELKQLNEECAR
GTCDGTDEEKKKKCEKACKNYREFLNNFKKQYENQKKEYEIIKSSYPKYEKKDAFTFL
KDKCNSNYSCFENKTEISVLKMFEHPPDDVKDECDCKTSKAHDDKVNDLDKCPNNINN
NKNICNKYKKRRICGDLKYSNSLDHWFGTNTLIPPRRRHLCLRNIIIKKNYRKGDISK
FKDDLFYAAASEAKFLFNNYENKNESLQAIKYTFADIGDIIKGNDMMDDMTYKKIKGK
LEKVLDKTGNNPETPEKWWEQNKKHVWEAMLCGYKLAGGEIKPNDCNIPTEESTHQFL
RWLTEWGTQYCKEKQQLKLNMQIPCMTHFDKYGIIENRIDVHPNCLQGLGKYEIWSNN
RIPDWERLSSKFNEVKGTMNENVKKLTAYEYLKQNCSKCICSFKDIEQTHKKSKDEGY
HIYEDILDKAQIPSFLEDTAYRYKGLNPECPEDIECSQYGNIPCRGVSHDDDNDWNSS
FVKDNKTTNLGVLVPPRRRHLCLRIDVNKFLRLRNDINNLKTFICKSAFAEAKRLKKV
YKDDNSKLHQAMKYSFSDIGSVVKGNDMMESPTSDNIAKIFRGMKYTEINRETWWDLN
KYHVWESMLCGYREAGGDTKKSENCRFPDIERVPQFLRWFQEWTEIFCIKRKTLYDKM
VTECQKAECDTSNGTVKETKCTKACEEYKSYVLSKKKEYYIQKDKYDNQFKKVLNNKD
AEEFLNVHCLSEYFKDETRWKNPYESIADKALKGKCDCKKMIPTTPEVKPKKPAVPEA
KKPEVEPLPSDEPFNRDILEKTIPFGVALALGSIAFLFLKKKTQAPVDLFSVINIPKG
DHDIPTLKSSNRYIPYVSDRYKGKTYIYMEGDSDSGHYYEDTTDITSSESEYEELDIN
DIYPYKSPKYKTLIEVVLEPSGNNTPTSDTPSDTQNDIPSGDTPNNKLTDNEWNTLKD
EFISQYIQSEQPNDVPNDYTSGNSSTNTNITTTSRHNVEEKPFIMSIHDRNLYTGEEY
NYDMSTNSGNNDLYNGKNNLYSGQNNVYSGIDPTSDNRGPYSDKNDRISDNHHPYSGI
DLINDTLSGNAHIDIYDELLKRKENELFGTNHVKHTSTHSVAKHTNSDPIHNQLNLFH
TWLDRHRDMCEKWNTKEELLDKLNEQWNKDNNNSGNINPSGNTPPTSDIPSGKLSDIP
SDNNIHSDIPYVLNTDVSIQIDMNNPKTTNEFTYVDSNPNQVDDTYVDSNPDNSSMDT
ILEDLDKPFNEPYYYDMYDDDIYYDVHDHDTSTVDTNAMDVPSKVQIEMDVNTKLVKE
KYPISDVWDI
>MAL13P1.1, 2162 bases, 8AE29CB6 checksum.
MGPPGITGTQGETAKHMFDRIGKQVYETVKNEAENYISELEGKLSQATLLGERVSSLK
TCQLVEDYRSKANGDVKRYPCANRSPVRFSDESRSQCTYNRIKDNETDDNACGACAPY
RRLHLCDYNLEKMGKTSTTKHDLLAEVCMAAKYEGDSIKTHYTIHKHTNNDSAAELCT
ELARSFADIGDIIRGKDLYLGDIKKKQNGKKTEREKLEENLKRIFGKIHEDVTNGKKE

VLKTRYKDINDPEFFKLREDWWTANRATVWKAITCHAGESDKYFRNTCNDSEHSGTFS
QPNKYCRCNGDKPGEDKANVDPPTYFDYVPQYLRWFEEWAEDFCRIRKHKLKNAKEQC
REKYKSGTDRYCSRNGYDCTQTIRGRNILVSDSECTNCSVVCTPFVKWIENKKLEFEK
QKGKYTKEIEKANGTSNGTTIRTQYGTINNMYRKDFYQQLQSGYGDVNAFLELLNKET
TCKDHPKVEEKSDIDFNEGTEKTFSHTEYCETCPWCATKKKGIDGNWEEQKYEEGCEN
YLMKPIDESKSTDIDLLVKDTSGTTMVEKLGGLCNDSSKRTVQMENWQCHYEKKSQYE
DGFDKDYCVLKDDKKKPEHRTIKSYYTLFPNWINEMLKDSIDWSKELKTCINNEKPT
NCIRECKSKCDCFKKWVVQKEQEWKQLEEHYEKENFSGDFGPRISPYVTLEGNLQYSY
LEMIRKYYAQEKPVQEIEQIIEKNKNNFEVKEDDNSITKFLQQEKGIATKCIEKQEEC
KQQKKQQRQKQPADKVVSRSGASPDTASPDTKATEEKEEEEEEEEEEDLGEESEEPED
QAVVDQDGQGETTEKKVPATTEEGSPKETTTPEKSVDVCKTVAELFSNVDNLKEACTQ
KYGGNNSRLGWKCIPTSGGEKATGGSGESTGSDATTGGSICVPPRRRRLYVTPLTKWA
TNMEATEAQAGGDEATEASVPLGDGVSKNPKEALLKAFVESAAVETFFLWHRYKKIKD
KEKKEKEERERENAGLDPFGGSVDDEASTPDPQTQLQSGTIPPDFLRQMFYTLGDYRD
ICIGGDRDIVGDTIVSNKEGGTPTKISDKIKEILEKVDKKQPDKQNSGTTPKTWWEEN
GPHIWNAMVCALTYKDNGDKGKPQVDDTVHSQLWDTTKNKPKNRQYEYDQVKLDENSG
TGPKSNDPINNPTLKEFISRPPYFRYLEEWGETFCRQRARMLKQVEKNCTQHGKKQYS
GDGEYCDKIHKDPTTLPDLGYSCPKSCSSYRKWIERKKTQYEKQEKAYNNQKANVQNN
NDNGFCGTLEREAAAFLNSLKNGPCKKENGKDNDEYEIKFDDKEKTFKHAKYCDPCPK
FKVDCKNGKCDNDKGGDCNGIKTIDAKNFETMVKPTEEIVMRVSDNSGNKFNDLSDCQ
NAGIFEGIKENKYKCGNVCGYVVCKSENGNGEKVNGIENQNKIITIRGLVAHWVQYFL
EDYNKIKHKISHCINNGEVSKCIKDCVKKWVEEKEKEWKKLKEHYQKQYGYNNSGESY
PVRSILEQFQSGTEFKNAIKPCGTLQQFESFCGLNGDESSKKENGNEDAVQCLLKNLG
NEAKKCEEKQQTSGIPEAPCVNHTPLEDDDEEPYEDLLLQETEEKPEEAKKNMMPKIC
KDVVQEAETVVESGCVPAKTPEAPAASPAPKEPPAPSEGTKERPSPPEKKAPLPKEEK
TKPKRSLTPTDDPWEPLKNAMLSSTIMWSIGIGFATFTYFYLKKKTKRLVDLFSVINI
PKSDYDIPTKLSPNRYIPYTSGKYRGKRYIYLEGDSGTDSGYTDHYSDITSSSESEYE
ELDINDIYVPGSPKYKTLIEVVLEPSGNNTTASGKNTPSDTQNDIQSDDIPSSKITDN
EWNTLKDDFIYNMLQNEPNDIPNDYTSGDIPLNTQPNTLYIDKPDEKPFITSIHDRNL
YTGEEYSYNVNMVNSMDDIPINRDNNVYSGIDLINDTLSGNHNVDIYDEVLKRKENEL
FGINHKKHTTINRFAKPARDDPLHNQLNLFHTWLDRHRDMCEQWNNKEEVLDKLKEEW
ENETHSGNTHPSDSNKTLNTDVSIQIDMNNPKTTNEFTYVDSNPNQVDDTYVDSNPDN
SSMDTILEDLDKPFNEPYYYDMYDDDIYYDVNDDNDISTVDTNAMDVPSKVQIEMDVN
TKLVKEKYPISDVWDI
>MAL7P1.187, 2595 bases, 3B9AD333 checksum.
MARPSGSAGGGAGGKKEDESAKHMFDRIGKEVYDEIVKKDVGAEAYKEALKGKLQKAA
STISELAGITDTCKRVQKYYEHFNVGAARGKRYPCTNLKRNTNEERFSNTLGGQCTNK
KMKCSNGEGACAPYRRLHLCHHNLETIETTSKTSTDTLLAEVCMAAYYEGESLTRQHV
KHKLTNSDVNINICTVLARSFADIGDIVRGKDLFYGNTYESTQRKVLDDNLKTIFENI
KKSDTKLTKLNDEQIREYWWEANRETVWKAITCSDDLKNSSYFRTTACAGTRTNDKCR
CTKSSGAKVDDQVPTYFDYVPQYLRWFEEWAEDFCRKKNKKIKDVKTNCRDEKEKYCS
GNGYDCRKTIYKKGKLVIGEHCTNCSVWCRLYEKWIDNQKKEFLKQKNKYTKEMKKYK
NGESRSGSDGNKYDGYESKFYKILKGGYEKVNNFLELLNEEKECKGISEVKEKIDFKT
VDNGFDKNINSPGTFYHSEYCKPCPGCGVKLEDNVWKEKKGGTCDRRKLYTTITNAES
TNIDVLSFGDKREDRETKLKAFCPKTNGDTTNGVHGSGDCGGTNSDPSLCEKWKCYKH
EHVQKVKNGEDDDDDDDVDGNYVKNGGGLCILPNPKKKEEEKTKKSEKEPDEFQKTFN
EFFYFWIGRFLNDSMYWRGKVGGCLKNKSEKCKNECNTKCDCFLKWITQKKTEWDKIV
QHFKKQDFGPQVENGGSGMLGGLMSCPDFVLKTVLKLEDLFENIKSGYGNVKETEGIN
KILDEEQKKNKEEADDGVGGGGIDFAAFAVSCTEDGVAKQNTTIDKLLNQEDKDATEC
KKCEEQAARARSAETNDEPPGSPPRSEEVESEEEEEEEEEDEDHGPDDEEAKTAEGAG
EGEGAEAPKEVVPGPKDAEVPAATTTTQNDVKVCKTVAEALKTSLTDACTLKYVTGKN

YGWKCIPTSGGDKTATSEGSSESGRRIAKRSVETSGSSGGSGATGKSDGSICIPPRRR
KLYLGGFKRLTDGTSVSSEPTTATSSPSPKGDSLLTAFVESAAVETFFLWHKYKAENT
KTQSESLLLPPQPVPVVDNDNPQNQLLSGKIPPDFLRQMFYTLGDYRDILFSGSKDEK
SSTYNDILKGDKEMKAKEEKIKEKITSFFQNGDSQPPNGKHVTQTSDKTPQQTWWQAH
GPDIWNGMVCALTYKEDTSGAKGESAKIEQDNEVETKLKEKLQKDKDYHYDTVTLKDE
QSGGDSTLNNPKLKNFVEIPTYFRYLHEWGQNFCKERKKRLQKIEGDCRVEDGSKNCS
GYGEDCKDNLLNKPYDTLPSFNCRSCGIECRKYKNWIKGKRKEFEEQKQEYSKQKTDA
EGNNNGNEFYTKLEECPEVKDFLQKLEPCKKDNGEGKTIFQDEAEAFGHKKYCDPCSQ
FKIDCKNGKCKSGDTKVNCNRKNTIDATEIENIKTNTKEVTMLVSDGNKKFFHGLNKC
RFAGIFEGIRKDEWKCGEICGYNVCKPINVNDLKVNGTQNQNQIIIITAFVKLWVEYF
FEDYKKIKHKISHCINSGNKSTCTNDCPNKCKCVKEWVEEKTNEWKTLKERFNDQYKN
DSQIYPVRSILEELIPQIDVTIDKKNYTSLEELEKTLKCNGSDKSQNGTQKDIIECLL
GNLKDKIETYPSSTSGSEQCTTPPSNLDDYTHTDDDDAHEKEKQSPKFCKDGVVPEKS
KVPESEDDSTKTSESTKKGESSIEDIFKTCPYDNDTCNNYRNKNNIGCPPKTHHTNLN
HWKNTLIKFDKGKSTDMNDGILIPPRRRQLCFRNIRKFHGRIDSEQKFREYFIADVYN
EAKQLSRYYAKDNEKILEAIKNSFADYGNIVKGDDMLGDGLSEIIQKILVKLNEKKSN
AEKLALQQLWENNKKYVWYVMLCGYKQGNHSVKNIENECTLPTTESEDQFLRWFQEWG
KIFCTRKKELKEEVKQQCSNSICTKHKTIENRCQKACKKYSNFISTNQNVYLLLKSQY
DKNYKRDKTGGREAHDYLKIKCKNGKCDCIVQNFIDDDKWEKPYETLDKNLKSKCECI
KPKPTCTTNTAETEKKKEEENDQLPAPKEQTDPGSDTPPPLPTPSKPDELLPIQADQP
FDPTILQTTIPFGIALALGSIAFLFLKKKTKSTIDLLRVINIPKSDYDIPTKLSPNRY
IPYTSGKYRGKRYIYLEGDSGTDSGYTDHYSDITSSSESEYEELDINDIYAPRAPKYK
TLIEVVLEPSGNNTTASGNNTTASGNNTTASGNNTTASDTQNDIQNDGIPSSKITDNE
WNTLKDEFISQYLPNTEPNNNYRSGNSPTNTNITTTSRHNMEEKPFITSIHDRNLYTG
EEISYNINMSTNSMDDPKYVSNNVYSGIDLINDTLSGNKHIDIYDEVLKRKENELFGT
NHTKKNTSTNSVAKLTNSDPIHNQLELFHKWLDRHRDMCEKLKNDNERLAKLKEEWEN
ETHSGDINSGIPSGKLSDIPSDNNIHSDIHPSDIHSGKLSDIPSGKQVLNTDVSIQIH
MDNPKTTNEFTYVDSNPNQVDDTYVDSNPDNSSMDTILEDLEKYNEPYYDVQDDIYYD
VHDHDASTVDSNAMDVPSKVQIEMDVNTKLVKEKYPISDVWDI
>PFF0010w, 2879 bases, 44511BE2 checksum.
MAPQRAEDSNKSAKEVLDEFGQQVYKEKVEKDAKIYKGELEGNLASSSILGETASTDK
PCTFEYNKLLGARGKRHPCGNRQTVRFSDEYGGQCTFNRIKDSEHNNNDVGACAPFRR
LHLCDYNLEKMGSTKIKDKNVLLAEVCMAAKYEGQSLLKQYEEHKNNYPHTNICTVLA
RSFADIGDIVRGKDLFLGHQQRKRKLEENLKQMFENIKKNNDKLDKISIEQVREYWWN
ANRDQVWEAITCHAAHSDEYFRKSTDGVTLYFDGRCGRELSSVPTYLDYVPQFLRWFD
EWSEEFCRKRNITLKSAKEECQNDSKKLYCSLNGYNCTRLIPNKNYCSRDPICTPCSN
KCIPYDLWLRNRRDEFNMQKGKYENEIKTYESDNDISNSNINTEYYKSFYKKFAKSDY
KKVHDFLTLLNNGRYCKEGVDGKDAIDFNKTDDKDAFDRSEYCQPCPACVVECDGGKC
EEKKNSDGTCIEAQIYTVVRDETPTPIKVLFSGDHQKDITKKLSSFCKNPESENNRDY
QTWQCYYKSSDYNNCEMKGSLYKVEGDPNIIVSHECFHLWVQSLLIDTIKWETKLKKC
INNTNVTNCYNKCNKNCECFENWVEQKKKEWENVNDVYKDQKQSLGIYYEKLENLFKS
NFFQVMKALEGDEKGKWYQFKDDLKKKFEPSEKNTRTTDSQDAIKLILDHLKDNATTC
KDNNSLEEDENCPKTKINPCIKRTRIPTRGASNNLVSVKHIAELMQRSARKQLEAGAG
EINLKGDATKGKYTKKNGKAVALNDICSIDVQHSNSTYRSEKPCAGKNTGRFDIGTPW
KTGTNVKMTEDQAYMPPRREHMCTSNLEYLETDQGPLKNSDGKFVNHSFLGDVLLAAN
HEAKKIKELYTKDNGLNDLKDKETVCRAMKYSFADLGDIIRGRDMWDNETGMKHAKKH
LKDVFDNIRKSLKNKGNQKYNYDDKKLPPYKELREDWWEANRHQVWRAMKCAIKEATI
DNCNGIPIEDYIPQRLRWMTEWAEWYCKMQSQEYKKLQDACTGCKKKVDSCTKGTPDC
EQCDKQCKQYTEFITKWQPQWETMSYKYQTLYEEAERDATSGSVKKRTQLSKEDQRVV
DFLKQLLLRNSAAARNRVIRAAGSSATGGTTAMTPNTPYSTAAGYIHHELGKTVGCNV
QTKFCKHKIGSKASGTENKEYAFREKPYDHDDACACRPPKPTGGPGAGESPARSDADS

RGPATVGAGEVEEDGDEVDSEEEEDEVEEEPPAEEVCEMVDTLLDESNGGKNGINGCN
PKDQVQPYPGWDCKLSTFKDKEHGSCMPPRRIKLCVSGLTQTNNIINKEDIRTHFITC
AAIETYFAWLRYKKINTEADKELKEGKIPDEFKRQMFYTFGDYRDIFFGTDISTHNHI
PEVSSKVITILEKENGTKSEDKQKFNNVLLEDWWKEHGPEIWEGMLCALTNGLTDDEK
KNEIKTKYSYDELNNAKKGDSSLEEFAQTSQFLRWMTEWGEDFCKKRKEQVETLQKAC
KFYECNINAEDTKTKCEKECKVYEQFIKQWKPQYEQQSKKFTTDKVQPEYAVDADVKK
STHAYQYLSKKLKKICQNGTTTDKCDYKCMENASRQPQTSACSQEQQQQNKSSTENNY
PEAFDCPPKEIADKCNCPKPPEPKYCVDKTAYDIRKEREKKSDNSLKGNGTDIPLTDC
TKVDTITFFNDNIGTTTINEKEMYKVFPPNSDSCDNKSSNRFNSEKQWLCNNINLKKK
NICLPPRRQYMCLRKIERMITKDVDDKDKFFQVVMKAAKEEGIRILKNYKEQNKTDFS
EICDDMKYSFADLGDIIRGKDLWNTDKNYKNIQDKIRYVFDYMHKKLNSDDQKRYKDL
VNHYDLRSDWWDANRRDIWKAMTCAAPRNAYIYKTTENSETKIRSTDMYYYCGHKDDP
PDNDYIPQKLRWMTEWSEYFCKELNRKLEQMKTNCDSCKLNDSNCRDSNDGNNCRKCQ
QNCQEYTKLVNQGKKQFILQDNQYKEIYKKISNNSDGKAYVGTHVVEFLKKVEKNKCS
DLNSADKYLYKGSNCKNLTFTENDNEHRTRTYAFTEKPIEYKNKCTCEITNHPLDKCP
TPQNRIICNNLKLINSYKKNYTINLKEWNNNLVPKISSDNYGVLVPPRRKHICLRNIT
ANFLENKIYGKEKLRNAVLNAAYNEAYFLWTVYNKDSTTAFEAIKYSFADYGDIIKGT
DIMESSLSDKIGNIFTNTKDTNARSKWWNEIKYQVWHAMLCGYRTANDKFVIDADTCK
LPTEDEIPQFHRWLIEWAKQACKEYRIRKSAFEQFCHCSTAGGLSGLDLLKNHSCNYE
LTQYIGWNTMVKQYMDGFDIKFQKVKNASTNSSISENSAQEYIKGKIEGNECDFNDME
NIYEKITNRKNKDFQEILGILCPNKKIDKDKSKEILDETSSKPKEEDTSHVQPPPLPP
KPSTPEVEPLPSDEPFDPTILQTTIPFGVALALGSIAFLFLKKKTLSPVDLFSVINIP
KGDYNIPTLKSSNRYIPYASDRYKGKTYIYMEGDSSGDEKYAFMSDTTDITSSESEYE
ELDINDIYVPRAPKYKTLIEVVLEPSKSNGNTLGDDIPHTNKFTDEEWNELKHDFISQ
YVQRESMGVPQYDVSTELPMNIGGNVLDDGINEKPFIMSIHDRNLYSGEEYSYNVNMV
NSMDDIPINRDNNVYSGIDLINDTLSGNKHIDIYDEVLKRKENELFGTNHTKKNTSTN
SVAKNTNSDPILNQINLFHTWLDRHRDMCEKLKNDNERLAKLKEEWENETHSGNTHPS
DSNKTLNTDVSIQIHMDNPKPINQFTNMDTILEDLEKYNEPYYDVQDDIYYDVNDHDA
STVDSNAVNVPSKVQIEMDVNTKLVKEKYPIADVWDI
>PFF1595c, 2238 bases, 21C71EA0 checksum.
MAPGRGAGGDGIEDTTAKHLLDSIGKKVHDKAKNAALDRSNSDLQGFLSEAKFEKNES
DPQTPGDPCQLQYEYHTNVTLGYDKENPCKKRSDVRFSDTKGAECDNRKIRDSEKKSN
YGACAPYRRLHLCDQHLEKINRYDKVNNHTLLTDVCLAAKFEAESLKTYRGQHQLTNE
GSQICTVLARSFADIGDIVRGKDLYLGNNKEKKQLEENLKKIFKEIYDKLDGKNGKKT
LQERYKGDTTNYYQLREDWWNINRKKVWDAITCGAAGGTYFRKRACSSYYPTGEDCRC
VANVPTYFDYVPQYLRWFEEWAEDFCRKKKKYVDIVKTNCRGESGKDKYCSGDGFDCT
KTVRAKGIYAIGDDCHKCSFWCGFYKKWLANQKQEFLKQKKKCENEMLSISKKKQSTK
YNVYEGYDEEFYKILKSGEVGGLNKFLELLKEQSECNRFSTDEGIIDFTKANDKPNEE
KGTFYRSKYCEECPECGVEKKDNGEFQKKEKNNGECDGKKLYEIPIDTKHNVIPVLSF
GDERDQIKNKINTFCTKKDNNREMEELTEQWKCYKEDDIKKHVENDYKDDVNGSGGIC
ILEKTNGDKNGKKQKTFNDFFHFWVRHLLNDSIEWREGLKKCLQNDKKTCIRKCNDNC
KCYESWVQQKKTEWNAIKEHFDQQKDICQQQGILGDRIKSPYFVLELVLELEYFPLIK
EAYGDAQAIEGINKTLDKKKQEEGVLGASNEETIIDYLLDHELEEADKCVKKNPLDKC
NQQKKQKQQEQDTGPARSNTSADTPRNGPTVEEVDSEEELENEEEGEEGTPPEENGGG
GGAEAKDTDRKGDRKVKETTEVTKDVVTPACEIVDKLFQNPQQFKEVACNQKYGYPQR
HWGWRCVAPSDTTKTTGSETAGSRTTRAADGAEPTRDDGNGDGAGGAPAKSGGSGATT
TSSGSICVPPRRRRLYVGGLTKWAEKQSSQGGGAPQVSPSATASSGSQSDPLLLTAFV
ESAAIETFFLWDRYKKIKEKEIEEKKKQENEKYNILGVKQEDILSDQDHPQKKLQKSG
EIPNDFLRQMFYTLGDYRDILVRGGHKTNGVHTNSDKTNIVLLASENRGEMEKIQQEI
DKILKQSGTEATSGAQENSVTTPQQTLWKDFAPQIWNGMICALTYEDSGDKGGKPTQN
EQVKGQLLESDGKKPKKDKYGDYDKVQLKEENDTEVKGQDGLTPQTTHLSKFVLRPPY

FRYLEEWGETFCRERKKRLKQIKHECKVEENGDRRRGGITRQYSGDGESCETISNHDY
SKVSDLEKPSCAKPCSSYRRWIERKKDEFIKQKDRYQTESDKVRSDNGFSKTLRNYND
AAQFLKRLKNGPCKNNDNGEGKIEFNEKSETFQHTDYCGTCSLIGVKCKKGHCDNAAN
GKDCPTGKITAGSFGNEGDSIGNVDMHVSDNGEKGFGDLKEACNGKGIFEGIRKDEWK
CDKFCGVQICGLKKNNDIDQNQIILIRALFKRWVEYFFEDYNKIKNKISHCTKNDKKS
KCISGCEEKCKCVSKWIDQKSKEWTIVRKRYLEQYKNADSGDTFPVRSFLEELIPKIA
VVNDQDNVIKLSKFDNPCGCSFEANSQNKNGHKDAIDCMITKLQKKIEECQSKHSVEK
TEKECQEYTPPVEDDEEDLLLQEEENTVEAPKICDDVLKTPPKQEEGEEKCEPAQTAP
KKPAADSERQTPEEKLPPPPAAKEEKPPKQNAEKTKPKRSPRPIDDLTPALKKAMLSS
TIMWSIGIGFATFTYFFLKKKTKSTIDLLRVINIPKSDYDIPTKLSPNRYIPYTSGKY
RGKRYIYLEGDSGTDSGYTDHYSDITSSSESEYEELDINDIYVPHAPKYKTLIEVVLE
PSGNNTTASGNNTTASGNNTTASGNNTTASGNNTTASDTQNDIQNDGIPSDTPNTPSD
IPKTPSDTPPPITDDEWNTLKDDFISNMLQNQPNTEPNMLGYNVDNNTNTTMSRHNVE
EKPFITSIHDRNLYTGEEYSYNVNMVNSMDDIPINRDNNIYSGIDLINDTLGGNKHID
IYDEVLKRKENELFGTNHVKHTTINRFAKPARDDPIHNQLELFHKWLDRHRDMCEKWE
NHHERLAKLKEEWENETHSGNNHPSDNTPPTSDIPSGKLSDIPSGKLSDIPSGNHVLN
TDVSIQIHMDNPKPINQFTNMDTILDDLDKPFNEPYYYDMYDDDIYYDVNDDNDISTV
DTNAMDVPSKVQIEMDVNTKLVKEKYPIADVWDI
>PFA0005w, 2163 bases, FF481D5A checksum.
MVTQSSGGGAAGSSGEEDAKHVLDEFGQQVYNEKVEKYANSKIYKEALKGDLSQASIL
SELAGTYKPCALEYEYYKHTNGGGKGKRYPCTELGEKVEPRFSDTLGGQCTNKKIEGN
KYIKGKDVGACAPYRRLHLCSHNLESIQTNNYNSGNAKHNLLVDVCMAAKYEGDSIKN
YYPKYQRTYPDTNSQLCTVLARSFADIGDIVRGKDLYLGNPQESTQRIILENNLKDIF
AKIHSDVMSTSGSNGRALQKRYKDTDNYYELREDWWALNRDQVWKAITCNAGGGNRYF
RQTCGSGEWAKDKCRCKDDKVPTYFDYVPQYLRWFEEWAEDFCRLRKHKLKDAKNKCR
GDSGNDRYCDLNRYDCTQTIRGNEHFVEKDDCKGCQYSCAHFVNWIDNQKLEFEKQKE
KYTKEIKKKHPTTIIIKTANRKTTINNLYVKEFYKKLQEKYGDVENFLQKLNEEQICK
NQPYNDESSIDINFKSIKDIDIFSHTEYCQACPWCGAKRKGKGWEPKEKTCGKTKTYD
PKKTTNIPILTPYISQQSILKKYNKFCNGNGGNGAPATATGGGQIKNWQCHYEGDNND
NCVEGEWKEFKEGKNVMSYNAFFWKWVHDMLIDSMQWRNEHGNCINKDNDNTCKNSCK
RPCECFKRWVDQKKKNEWEAIKDHFKKQNIAAETQCDPGVTLQWVLILDFLKDESTED
KENKVSAEEAKEIKHLRQMLQQAGVDDPAAFARPCTEDGVAEQDTIMDKLLNREENDA
TECKKCDKPPPAPTAGDRGPGARADPHDVQQPRPPGSGPGTDANDEDDDDDDDDDDEE
DGEAKEEEEDEEKQEDVHQEEKAKKEEPQKEEVARTPKDDVNVCNIVNNVFTDGSSLQ
AACSLKYGKNAPTSWKCVTPSGNTSDTTVKSGDTTGGSICVPPRRRRLYVTPLTRLTG
GDSTTQASQASEVQTQARGSNTDKSPGSSEAAQGDGVSKDPQKALLKAFVESAAVETF
FLWDRYKKIKEKEKKEKKKTYEQIYESTDYDDEEKDPQEELKKGIIPDEFKRQMFYTL
GDYKDILYSGDTVNGGNEDKIKKAINNYFQKIREQSSSDNNPSPRSVKTPSTSDKDPQ
TWWNAHAPSIWNAMVCALTYDTNSGGEGKTTTITQDPNLKTALWDENGKKPLKTKYQY
DSVTIGASGAKPQTKAKPTGGDTPLTQFVLRPTYFRYLEEWGQNFCKKRTEMLEKIKY
ECKVGQGRGGRKQKTPQCSCYGENCDDQLDDNPSTDADLKCPGCGRECRKYKKWIEKK
KEEFTKQSNVYEEQKTKCQKESKSAKGNNHGNEFCGTQGTCDTAGDFLNRLKSGPCKK
ENGKDNQEDEINFKDEDKTFGHENYCAPCPVFKDICKKKDCRNASNNMCNGKDFITAE
DIKIMDSSSEEVNMLVSDNDTNKFDGGLDACKDAHIFKGIKENKWSCGNVCGYNVCKP
KKVNGEKGSGENNDQIITIRGLVTHWVQNFLDDYNKIRTKLKPCRNNGEVSKCIKDCV
KKWVEKKTEEWPKIRDRYLEPYKSDDGYNKKSLVRSFMETLIPLMDLTNGKEKIQELN
KFLRSYECNCADNSQQKGDTPKDIVECLLEKLEDKANKCKTQTSGTDCHPSTPLEDDD
EPLEETEENTVEQPNICPTKQPQPEKEDGCEAAPTTAEETSPTATSEGTENQSPPPPP
PAPAPAPAPAPEKSQPKEDKKVEPQPKPQPTNPPPNLFNNPAVIPALMSSTIMWSIGI
GFAAFTYFLLKKKTKSSVGNLFQILQIPQNDYGIPTLKSKNRYIPYRSGTYKGKTYIY
MEGDSSGDEKYAFMSDTTDVTSSESEYEELDINDIYVPHAPKYKTLIEVVLEPSGNNT

TASGKNTPSDTQNDIPTSDTPPPITDNEWNTLKDEFISQYLQSEQPKDVPNDYKSGDI
PLNTQPNTLYFNKPEEKPFITSIHDRDLYTGEQISYNIHMSTNTMDDPKYVSNNVYSG
IDLINDALNGDYDIYDEILKRKENELFGTNHVKQTSIHSVAKLTNSDPIHNQLELFHK
WLDRHRNMCEKWKNDNERLAKLKEEWENETHSGNTHPSDSNKTLNTDVSIQIDMDHEK
RMKEFTNMDTILDDLKTYNEPYYDVQDDIYYDVNDHDASTVDSNNMDVPSRVQIEMDV
NTKLVKEKYPIADVWDI
>PFA0765c, 2203 bases, FA7A87C0 checksum.
MVTGSGGEDKYKSAKNAKELLDMIGKDVHEIVEKDEAKKYIDELKGNLQKAKGIGELA
AFPDTCKRVEQYRSKANGDGKRYPCTELSEKYVERFSNTLGGQCTDSKMRRDGIGACA
PYRRLHLCHHNLENIKDVNNIDNDTLLAEVCMAAYYEGESLTRYNPIYQTKYKDSGST
MCTELARSFADIGDIVRGRDLFRGNDEEKKKRDELEKNLKTIFGKIHSRLTKDAQNYY
EDNDTDKNYYQLREDWWKVNRDQVWEAITCEAKSDDKYNVIGPDGKITESNKGQCRCF
SGDPPTNMDYVPQFLRWFEEWAEDFCRLRKHKLKDAIDKCRTPKGKEKYCDLNRYDCE
QTIRGDHDFVEDDVCKGCQYSCSHFVNWIDNQKLEFDKQKRKYKSEIKKYKSEITGGA
GGKGKTRKRRSASNENYEGYEKKFYDKFKSRYKEVGEFLGLLNNETTCTKKLNDQGEE
EGTINFKNVHSGKHSSGGDGNKTFYRTKYCEACPWCGAQKVEGGWKDKNKDCAKKKER
IFDEHNTTTIEILTADKKQLDILKKYSKFCDSVNGTANVEKDKKRVSNGATGKKGNQI
VTWQCYFDKEKPSKKNNNCVEGEWKDFEEGKSVKSYNGFFWKWVHDMLIDSMQWRDEH
GNCINKDNDNTCRNNKKCNKECGCFQKWVEQKKKEWQNIKKHFDKQTDIVIEGGPLGE
LSHCGVLEWNLKEEFLKDESTEDKENKVSAEEAKEIKHLKKMLQQAGVDVGDLAAFVG
PCTKGPVAGQNTTIDKLLNQEDKDAKDCQSKHNDCPQPPAEEVGVARADTPTQATGGP
RPATTKNTEDDDAEEDEEDEEEEEEEEEEKENADAGGEDGKEEKSEEKEEDPPAAVEDT
AVKRPQQETQPEEAPTATDPSLNVCTTVDKALTDQTNLTKACQQKYEKGREKFPNWKC
IPSGDNSTTREGSESGLRRSRRDADSQTPGEKTTPPSGTNQGAICVPPRRRKLYIQKL
PDVEFDDKSLRKWFIETAAIETFFLWDRYKKIKAKEKKEKEDAKGQIYESAGEDDEDK
DPQEELQRGDIPDGFLRQMFYTLGDYRDILEGKNDILIGKTGTGSAKDEMADKENKIK
EAIKKFFQNGDSQLPSGKPGDERKKFWEANEKHIWNAMVCALTYEEKTSSASGGEKNT
TITQDDGLKGALIKDGNPKNPQYHYEKVTLENSGPSPKLQTGSPGTSGDNTPLTQFVK
IPTFFRWLHEWGSDFCGTRKRMLDKIIFECRGNGKVCSGDGEDCKDQLKHNPSTVRDF
LCPTCGRHCSFYKKWIDIKKKEFEEQSNAYTGQKDKCKKDSNNGFYRKLQTYNEAKDF
LQTLRPCKTNNENNNGDDKLDFTNPKETFRPAKNCKPCSEFKVKCDYDNCTGANANTC
TTRKISPKDIDEKTDPNGNIEMLVSDDSATGFAGDLNDDCKNAHIFKAFREDVWTCGK
VCGYNVCKPVKVNGETFDAKKKGENQIIIIRALFKIWLEYFLEDYNKIKKKLNPCRNN
GEVSKCINDYDEKHKCVEQWIEKKRAEWEKIKKHYKRQNEKGDTEMISLVRNFLGDVQ
PQTEVHKAIQPCKDLDKFQDSSYCTVNGSSAKGKDGTQKDIVECLFQKLEEKAKTCST
STSEETQNTAQCQDTHPDDDLSLEETEEVKAPNICPKDPESKKDEPDDCCEQAKALPK
ETADVVADNGSGNDEVQEEEESEEKNKGDAAPLPEKPSGDSTSTDQTEPPAPAPQPLP
SDNTSDILKTTIPFGIALALTSIAFLFLKKKTHAPLDLFSVIDIPKSDYDIPTKLSPN
RYIPYTSGKYRGKRYIYLEGDSGTDSGYTDHYSDITSSSESESEYEELDINDIYAPRAPK
YKTLIEVVLEPSGKNTTASGNNTTASGNNTTASGNNTTASGKNTTASDTQNDIQNDGI
PSSKITDNEWNTLKDEFISQYLQSEQPNDVPNDYKSGNSSTNTNITTMSRHNVDQKPF
ITSIHDRNLYTGEEYNYNVNMVNSMDDPKYVSNNVYSGIDLINDTLSGNQHIDIYDEL
LKRKENESFGTNHVKQTSIHSVAKPISDDPLHNQLELFHKWLDRHRDMCEKWNNKEEL
LDKLKEEWENETHSGNIHTSDSNKTLNTDVSIQIHMDNPKPINEFTNMDTILDDLKTY
NEPYYDVQDDIYYDVNDQDVSTVDSNAMDVPSKVQIEMDVNTKLVKEKYPIGDVWDI
>PFB0010w, 1711 bases, BE834973 checksum.
MATGSGGDSSRDESVKDLFDRIGKKVYEKTEKIAKRYTTELHGDLSKATYPNDKHPEG
STENNPCKLQYDYNTNVTHGFGQEYPCETDIVERFSDTEGAQCDKKKIKDNSEGACAP
YRRLHVCVRNLENINDYSKINNKHNLLVEVCLAAKYEGESITGRYPQHQETNPDTKSQ
LCTVLARSFADIGDIIRGKDLYRGGNTKEKKKRKKLEENLKTIFGHIYDELKNGKTNG
EEELQKRYRGDKDNDFYQLREDWWDANRETVWKAITCNAGSYQYSQPTCGRGEIPYVT

LSKCQCIAGEVPTYFDYVPQYLRWFEEWAEDFCRKKKKKIPNVKTNCRQVQRGKEKYC
DRDGYNCDGTIRKQYIYRLDTDCTKCSLACKTFAEWIDNQKEQFDKQKQKYQNEISGG
GGRRQKRSTHSTKEYEGYEKHFNEELRNEGKDVRSFLQLLSKEKICKERIQVGEETAN
YGNFENESNTFSHTEYCDRCPLCGVDCSSDNCRKKPDKSCDEQITDKEYPPENTTKIP
KLTAEKRKTGILKKYEKFCKNSDGNNGGQIKKWECHYEKNDKDDGNGDINNCIQGDWK
TSKNVYYPISYYSFFYGSIIDMLNESIEWRERLKSCINDAKLGKCRKGCKNPCECYKR
WVEKKKDEWDKIKEFFRKQKDLLKDIAGMDAGELLEFYLENIFLEDMKNANGDPKVIE
KFKEILGKENEEVQDPLKTKKTIDDFLEKELNEAKNCVEKNPDNECPKQKAPGDGAAP
SDPPREDITHHDGEHSSDEDEEEEEEEEQQPPAEGTEQGEEKSESKEVVEQQETPQKD
TEKTVPTTTPTVDVCDTVKTALADTGSLNAACSLKYVTGKNYGWRCIAPSGTTSGKDG
AICVPPRTQELCLYYLKELSDTTQKGLREAFIKTAAQETYLLWQKYKEDKQNETASTE
LDIDDPQTQLNGGEIPEDFKRQMFYTFGDYRDLFLGRYIGNDLDKVNNNITAVFQNGD
HIPNGQKTDRQRQEFWGTYGKDIWKGMLCALQEAGGKKTLTETYNYSNVTFNGHLTGT
KLNEFASRPSFLRWMTEWGDQFCRERITQLQILKERCMVYQYNGDKGKDDKKEKCTEA
CTYYKEWLTNWQDNYKKQNQRYTEVKGTSPYKEDSDVKESKYAHGYLRKILKNIICTS
GTDIAYCNCMEGTSTTDSSNNDNIPESLKYPPIEIEEGCTCKDPSPGEVIPEKKVPEP
KVLPKPPKLPKRQPKERDFPTPALKNAMLSSTIMWSIGIGFATFTYFYLKKKTKSTID
LLRVINIPKSDYDIPTKLSPNRYIPYTSGKYRGKRYIYLEGDSGTDSGYTDHYSDITS
SSESEYEELDINDIYAPRAPKYKTLIEVVLEPSGNNTTASGNNTPSDTQNDIQNDGIP
SSKITDNEWNTLKDEFISQYLQSEQPNDVPNDYSSGDIPLNTQPNTLYFDNPDEKPFI
TSIHDRDLYSGEEYSYNVNMVNTNNDIPISGKNGTYSGIDLINDSLNSNNVDIYDEVL
KRKENELFGTNHTKKNTSTNSVAKELCGDPIMNQLDLLHKWLDRHRDMCEKWNNKEEV
LDKLKEEWNKDNNSGNINPSGNINPSGNTPPTSDIPSGKLSDTPSDNNIPSSNKTLNT
DVSIQIHMDNPKPINQFTNMDTILEDLEKYNEPYYDVQDDIYYDVHDHDVSTAGSNAM
DVPSKVQIEMDINTKLVKEKYPISDVWDI
>PFB1055c, 2199 bases, 46227279 checksum.
MGSGKGGDPQDESVKHMFDRIGEDVYEQVKSETVNYVSELEGKLSLAPILGVESGSTN
ETCNLVQDYYNKPVYGNSNRYPCKNLKGITNEERFSDTLGGQCTNKKIKGNEYSTKSG
KDCGACAPYRRLHLCSHNLESIDTTSMTHKLLLEVCMAAKYEGNSIDTHYPQHQRTNE
DSPSQICTMLARSFADIGDIVRGKDLFYGNSKEKEKRDELETNLKTIFGKIHEKLKDK
EGAETRYGSDTTNYYQLREDWWYANRATVWEAITCDVHGSDYFRQTCGDKETTATRVK
DKCRCKDENGKKPGSNADQVPTYFDYVPQYLRWFEEWAEDFCRKKKKKLEKLEQQCRD
YKQNLYCSGNGYDCTKTIYKKGKLVIGEHCTNCSVWCRLYESWIDNQKLEFLKQKQKY
ETEISNSGSCGGSGGVKGRNRKKRGAGVETATNYDGYEKKFYKELKESEYGKVDDFLK
LLNNEDVCKKIKDEKEKIDFTKPADKNSNNEGTFYHSEYCKPCPDCGVKRKDNQWKDK
YDGKCTRGKLYEPASGAQGTPIKILKSGEKQKEIETKLKAFCDQTNGDTTNSVARGGG
ADGSGSKSNSKELYEEWKCYNEVQKVKDDKNGEEEDEDEEDVDKVKKAGGLCILENKK
HESRNNSSNEPEQFQKTFHDFFYFWIGRFLNDSMYWRGKVNSCINNPKRKKCRNECKD
DCGCFKEWIGKKKEEWENIKKHFKTQEAFKNKRENSGIDMFSGLMDSADVVLELALEL
EQLFQDIKDGYGDVKELKGIKELLDEEKKKKQAEEAVVVVVADNQKKTTIDKLLQHEG
DDANNCLKTHKEKCEETQPKPPGAGGPGAPSETGETTTLEDEEEEEDEEEDAGDEVEE
GETVDTTEGDETETVEQPVKDTDREGEEEEAKKATDTTTSLDVCDTVKNALTNNDNLT
DACKLKYGPGGKERFPNWKCVSSGEKSVATAGSSGATGKSGDKGAICVPPRRRRLYVG
GLTKLTSAGTSSESPQGGSESSRASDVSQGNGGDDITTTESLRKWFIETAAIETFFLW
HRYKKEWEAQKKAELQRNGLLLGTGASLNLGGDDSNPQTQLQKSGTIPLDFLRLMFYT
LGDYRDILVRGVADDKNGGNNIILNASGNKDEKQKMEKIQEKIEQILPTSGNKETRGP
QNSVNDRQSLWDRIAEHVWHGMVCALTYKDDDNGLKGVVKKPQKIENPEKLWNETTKK
PKDEKYQYQTAKLEDESGEKRPDSSASGTKLTDFIKRPPYFRYLEEWGENFCKKRTEM
LGKIKEDCYKNGGRCSGDGLKCNEIVIDKEKIFGDLLCPTCARHCRFYKKWINTKRDE
FNKQSNAYSEQKKKYEEENDSAQKNNGVCGTLKDDAAEFLNRLKNGPCKNESEENKKA
EDEIDFKKPDDTFKDADNCKPCSEFKIKCENHNCSSGGNTQGKCDGKTTIAATEIENI

KTNTKEVTMLVSDDSKSATEFKDGLSECKDKGIFKGIRKDEWECGKVCGVDICNLKKK
DNIGKESDKKYIIMKELLKRWLEYFLEDYNKIKHKISHCTKNGKGSKCIKGCVDKWVQ
QKKEEWKQIKERFNEQYKSKTSDEYFNVKSFLETWIPKIAVVNDQDNVIKLSKFGNSC
GCSASAISTNGNEEDAIDCMIKKLEKKIDECKRKPGENSGQTCNETLTHPLDVQDEDE
PLEETEENPVGKQHPSFCPPVEDKKKEEEGETCTPASPAPAPAPAPASPSPTPAPADE
PFDPTILQTTIPLGIALALGSIAFLFLKVIYICVVYMYIYMCFCIYMYVWKKTKHPVD
LFSVINIPKSDYDIPTKLSPNRYIPYTSGKYRGKRYIYLEGDSGTDSGYTDHYSDITS
SSESEYEEMDINDIYVPGSPKYKTLIEVVLEPSGNNTTASDTQNDIQNDGIPSNKFSD
NEWNTLKDDFISNMLQNQPKDVPNDYKSGDIPFNTQPNTLYFDKPEEKPFITSIHDRN
LLNGEEYSYNVNMSTNSMDDPKYVSNNVYSGIDLINDSLSGNKHIDIYDEVLKRKENE
LFGTNHVKHTSIHSVAKNTNSDPILNQINLFHTWLDRHRDMCEKWENHHERLAKLKEE
WENETHSGNTHPSDSNKTLNTDVSIQIHMDNPKPINQFTNMDTILEDLDKPFNEPYYY
DMYDDDIYYDVNDHDTSTVDTNAMDVPSKVQIEMDVNTKLVKEKYPIADVWDI
>PFA0680c, 186 bases, 95132A2 checksum.
MLAQKNTNKKPFGNTLTNILFKDKKKKNLDPQISSLVSLVDNMNITQEKKDKIKNLSL
KYINSRDVKEKNESINELQKYSNNEECKEYMDSYLMHLRMQNDIKCLKRKNLWNNIWI
VSTTLLLIIIMIVCLFSVDITSSSALGPAFILLIFIIHIVARYFPDMKIGFKKLKTKL
NTFFQNKKQITK
>PFC0005w, 2209 bases, 1F149D19 checksum.
MVRTLDPEEELRGIEDTTAKHIFDRIGKIVHEKAKKNAEQYRSQLKGSLLKATFEKAP
AGQQTPGNTCELKYQWHTNVTKGGNKEYPCRNGTEKRFSEVSGGECDDSKIKGSNGGA
CAPFRRLNLCVRNLENINNYGKINNDTLLADVCLAALHEGDSIRGDHDKYKETNDSSQ
LCTMLARSFADIGDIIRGKDLYRGNNGKDKLEENLKTIFGKIHEGLKNGKTNGIEERY
GNDPDFFQLREDWWTANRETVWKAIRCSAPRDADYFIKTVCSGGKTPTQGKCRCIDFS
VPTYFDYVPQYLRWFEEWAEDFCRLRKHKLQNAKNKCREKHKDGKKLYCDLNGFDCTQ
TARGKNKYKYEHDCIECYSSCDHFVHWIDNQKKEFEKQKNKYDKEIKKAYGKNGTTTK
ETSNGPINNLYVGDFYSKLQQTYGSVDKFLKKLNDEAICKKHPEVEEKTDVNFNENLD
DIFSHTKYCQACPLCGLENDSPPWKPKPEKECRDQQIRNFDDNESNEIKLLVKDKGGQ
TMVEKLGGLCGNGAKKNNIQEKTWKCYYDKNKENSIGGGDKDYCVLKNDKKNRTQLEI
VSFNSLFWRWVTEMLKDSIDWRKEYKNCINNGDKSTCKNVCKKPCDCFQKWGVRKTKE
WQQVKAHYEKEDFGKGLTPYKTLEWVLDLSYFPIIKEAHPKEKPVQKMEEIIKKNQEN
ISRVTKQNNSITKFLQQELQEANNCLQKRKQDCKPPQQSAEEGVAKTGQPRAEDEEDS
PRPDAGAGEVDDEDDDADNDDEITPRDLNIEVDDLDSKDPEDQVEEEKAKDNTDETVE
TAKETKEDTDRKGEEKQPKEEVDNVKPCEIVKTLFTNGDNTALNEACKQKYQYGKEKF
PNWKCVPTTRGSGEPTGSSGSICVPPRRRRLYVTPLTRLAGGGNTAASQGSGEAAQPV
TVTQPQASGGNTQVAVSPGGAASSTSTTESSQLLRQAFIESAAVETFFLWHKYKVDKE
KEDKEKNGRNMVVYTSPVPNDLYEKLKKGEIPDDFLRQMFYTLADYKDILFSGDKDNK
NGYNDILSGDKELHEREKEIKGAIEKHFSNSVKTPSTSGNDAKTWWKENAPHIWEGMI
CALTYKETSGSGGEKQIEKDSDVYNKFFGSTAATQKGTYESKYKYTDVKLEENSGTGG
PRGPNESPNSKPPSSTSENKPTTLDSFIKRPPYFRYLEEWGETFCRERAKRLAQIKVD
CRGDENTNRSNDGDGFDCEKKVTNKDVFLEDFNGSSCATCCSSYRKWIERKKIEFEEQ
ENAYEEQQKKNCVNGNNKGGVNGVCGKLEENAAGFLQKLGSCKKDSGEDNGNGNEEDK
LNFRQPNVTFRPAENCKPCSLIEIKCKNGVCNGDPTKGECNGETVTAEEIEKMNDLNG
NIDMLVSDNGKNEIPEDLKSSCKDANIFKGIREDVWKCGKFRDVDVCVLKNFNKHIHD
KKNVLIRTLFKRWLEYFFEDYNRIQKKLKPCIENGKGKEQKCFKGCKENCDCVKKWVE
EKEKEWPKIRKRYLEQYKNAGGSDDYKVKSFLEDPQFYNEVNKAVKPCDDLNAFERSI
HCNGPNSSQKKDVERRDVVVCLLDKLEKEAKKCEQKHQNSGNPQQPCEGSTPPDDEEE
ELLEEDEQNTVGKEKVGNKAPAICGDVEEQKEKEEGDCDKAVTPDSDTGGNGEKEDSR
SEEEEEVSGSGDQGSPPAPPPPESPQEKAPAPAPEELPPGPERPPKPAAPTSTPEVPA
QPLPSDNTSDILKTTIPFGIALALTSIALLFLKKKTKSTIDLLRVINIPKSDYGMPTK
LSPNRYIPYTSGKYRGKRYIYLEGDSGTDSGYTDHYSDITSSSESEYEEMDINDIYVP

GSPKYKTLIEVVLEPSGNNTTASGKNTPSDTQNDIQNDGIPSDTPNTPSDIPKTPSDT
PPPITDDEWNTLKDEFISQYLQSEQPNDVPNDYKSGNSSTNTNITTTSRHNVEEKPFI
MSIHDRDLYTGEEYNYDMSNNSGIYPSSSNRDSLSGTKVPYSGIDLINDSLNSGNQPI
DIYDEILKRKENELFGTNHVKQTSIHSVAKLTNSDPIHNQLELFHKWLDRHRDMCEKL
KNDNERLAKLKEEWENETHSGNIHTSDSNKTLNTDVSIQIDMDDGKPKKEFTNMDTIL
EDLDKYNEPYYDVQDDIYYDVNDHDVSTVDSNAMDIPSKVQIEMDVNTKLVKEKYPIA
DVWDI
>PFD0005w, 2646 bases, 588715EB checksum.
MVRTGGSGGGGVDKDGIDHQSAKHLLDSIGKKVHDQVKNGADGTGASGDAKNYIDDLK
GDLQKAPNINPKLIGTDDPCKLVEDYYNNHVNGDGKGERYPCTELSGKKFQNPFSDTL
GGQCTNSKMRSGCEGACAPYRRLHLCHHNLESIETTSKTASDTLLLEVCMAAKYEGQS
INTHYTKHEHSNKDSPSQLCTVLARSFADIGDIVRGKDLFYGNTYESARREKLENKLK
EVFGKIHGGLSEEAKKKYQDGDGNYYQLREDWWTANRETVWKAITCEVKSGNNYFRAT
CGDEKNPSLTSKQCRCDKDKAGKPIKGSGNVNIVPTYFDYVPQYLRWFEEWAEDFCRL
RKHKLKDAIKKCRGKNGEEKYCDLNRYDCKNTASGKHVFFEDFDCKDCQYSCAPFVDW
IDNQKLEFLKQRKKYTKEITSGGSCGGSGRKKRDATTTNYEEYEKKFYKELKGTKYKV
VNNFLEKLNDEDVCTKNNDIKDGGNIDFKNVHSGSAKKGDGNNKTFYRTKYCEACPWC
GAEKVEGGWKAKEENCSQTKDYDPDKTTTIEILTGDTRKSDMVQKYKKFCNGNGGNGE
KSATPNATSREKGKKGDQMEKWICYYDENKEKKYGSDAINFCVLQDGKQHRKEQKVTS
YNAFFWKWVHDMLHDSVEWRERLNSCINNAKSQNCKNNEKCNKECGCFEKWVKQKKEK
EWEAIKDHFGKQKDIIEQTGCDAGVTLAAVLKLEFLNEDTEEKSEKGLDAEEAKEIKH
LRQMLEQAGVRDLAAVGGPCTEGGVAEQNTIMDKFLDEELKEAEQCKNCPKPKAQQEG
PGGARSADSPPAGTEDHPDAEDDDDEDDDDDDDEDEEEEEEEEDPQCKTVNDILSTDD
RTKQVGDCHEKNYGKNGPDWKCGDLTLVDDTKVCMPPRRQKLCLYYIAHESETKNIET
QDDLRDAFIRTAAAETFLSWQYYKIKNGADAKQLDNGTIPEEFLRSMYFTYGDYRDIC
LNTDISKTVNDVAKAKDKIGKFFSKDGSKSPSGTTTPQDWWQTYGKDIWKGMICALTH
GVTNTEKKTKIKNDYSYDKVNQSQNGNPSLEDFAKKPQFLRWMIEWGEEFCAERGKLE
QNIGKSCNGINPIQYCSDNRHPCNKACDEYKNYVETKQKEFRGQTTKFVRDANLENAD
QEYKDYKTTQGPSKQGNDYLKDKCDNKKCSCMEGNVLTDVSSKKPFGIYAHKYSEKCN
CLGAKFVPTNVPPAPPPQPPPPPAIPAPATTPGVNPCEIVNTLFSDTNKFKDACTLKY
GPKAPTSWKCIPTGNTSNEGAATDSEGSDAKSRHKRDLAPSSGSNQGSICVPPRRRKL
YVTPLTKWAEETTKGSKSQESGKAEGTSESSGSEASSPGGTSSQGEKSPQGLSTPAST
SSPSNSRDDDLLKAFVESAAVETFFLWHKYKMDKQKELDEKKKQQRESGLVGALDGNS
GNVDDEDKDPQKKLEKGDIPEEFKRQMFYTLGDYRDILVRGGNTSDSGNTNGSNNNNI
VIEASGDKQDEMKKIQKAIDEHINSLKQAASVPNPQRPGQQQQNSSLTRETLWKEHAP
SIWEGMICALTYKENDEKKIVKDNEVYEKFFGTTPGTTSGKYKEKYEYNTVKLDENSD
TEAKDTKATAPSDNTPTFLSHFVLRPPYFRYLEEWGETFCKERKKKLAQIKVDCKVDS
ADYKCSGYGEECKIEDISNIGVFADLKCPGCGRECRKYKKWIERKKIEFGEQKSAYVK
QKTKCKEESGGGGNGVCGTVKTCDTAAQFLERLGPCKNNDNGEGKIEFNEQSETFKHT
KHCDPCSSFKIDCRNGKCKSGDTKGKCDGITTIDAKEIAKMISSTPDVVMRVSDNDTN
TFEGDDLKVCEGKGIFKGIRKEEWKCRNECGLDVCGLKKGDNNGKLDDKQIIILIRALI
KRWLEYFLEDYNKIKHKISDCINNGEGNICKRDCQNKCNCVGEWIKLKKEEWEKIKKH
YLEKNKEGDNDMKSSVRNFLEKFEHRPEFNKAIKPCKGLTQFESFCGLNGDKPSQNGH
QDAIDCMIKKLEDKITSCLSSTSGEQTETECQEHTPLPDDEDLLLEETENPVGKQQPS
FCPKVEEKKETVDEGKCGEDTSEPKKTEDEESKKKKDESTPDSPEPPPPATPEAPKEE
KKVEPQPQPQPTTPQIVDKTPALVTSTLAWSVGIGFAAFTYFFLKKKTKSTIDLLRVI
NIPKSDYDIPTKLSPNRYIPYTSGKYRGKRYIYLEGDSGTDSGYTDHYSDITSSSESE
YEEMDINDIYAPRAPKYKTLIEVVLEPSGNNTTASGNNTTASGNNTTASGNNTTASGK
NTPSDTQNDIQSDGIPSSKITDNEWNTLKDEFISQYIQSEQPKDVPNDYSSGDIPFNT
QHNTLYFDKPDEKPFITSIHDRNLYTGEEYNYDMSTNSGNNDLYNGKNNLYSGQNNVY
SGIDPTSDNRGLTSGKHDSYSGIDLINDTLSGNQHIDIYDEVLKRKENELFGTNHVKH

TTINRFAKPARDDPLHNQLELFHTWLDRHRNMCEKWNNKEELLDKLKEEWENETHSGN
THPSDSNKTLNTDVSIQIDMDNPKPINQFTNMDINVDTPTMDNMEDDIYYDVNDHDTS
TVDSNTMDVPSKVQIEMDVNTKLVKEKYPIADVWDI
>PFD0020c, 3467 bases, 941CB843 checksum.
MGTGSSTPSVPKDVKNESHNSARNVLENIGIEIYNEEKKKVNGYTSQLRGDLSRARFH
DGLRKAARLGVIPGPANSCDLDHKFYTNINNGYPPARNPCDLRNQNRFGENAEAYCNS
DKIRVTGKKSAGGACAPFRRQNMCDKNLEYLDNTNTDDTDDLLGNVLVTAKYEGESIV
AKHPHKENSEVCTALARSFADIGDIVRGKDMFKRNEEDAVQKGLRAVFKKINDNLKEK
EISDYDNDPNYYKLREDWWTANRDQVWRAITCYIPYYVNYFKKTSDDTIVFTNDGKCG
HYEGAPPTNLDYVPQFLRWFDEWAEEFCRIRNHKLRKIKDACRNDKERLYCSQNGYDC
TKRIEKGSSCSRENKCTGCSNKCVDYDFWLEKQQNEFKIQKDKYDKEIETYVNKTPIS
NSNSNTKKEYYKEFYEELKKQYGSVKNFLQLLNNGRYCQEKIEEEDAIDFTKTGNKHA
FYRSDYCQPCPDCVVECDGKTCTQKTDDDKNCRSKIIQKILESETPIEIEVLYSDDKQ
GVITEKLKDFCRGPNNYNDENLQKWKCYNKNSEYNKCEMISWLYQDPKEYNLMLSVEC
FHSWAKNLLIDTIRWEHQLKNCINNTNVTDCTSKCIKNCECYEAWIERKKDEWEKLKE
VLNKKDETSHNYYNKLKDVFDRFLFQVMFALDQDEKGKWDQFTEDLKKKFGPSVESAG
TANSQDAIEFLLDHLKDNALTCRDNNSIKPCTYPPNPTPNPCGTNNNGGKLVRVKRLA
EMMQRRARKQLEKRGGEINLKADASQGKYIRGGKEKKLNGQICNIDTSYSNDSRNGNN
GGPCTGKNDKRFKIGTEWSYGEHEKKRTHPEVYMPPRREHMCISNLEKLDVVSVIKNG
NASHSLLGDVLLAAKYEAKNIKELYQQNNSKNGVIDQNDKETICRAMKYSFADIGDII
RGKDMWVQNTDATKLQAYLAKIFDKIKDNHKDIKGKLQYNGDTDHKLLREDWWEANRH
QVWRAMKCAIENDKDMKCNGIPIEDYIPQRLRWMTEWAEWYCKEQSRLYGELLEKCQS
CKGKQKCTEGDVDCGKCKAACDKYKDEINKWREQWTKIKGKYKTLYKKATKPGVTTSN
NPKDEKDVVDFLKQLLPRKSKNTPGVTAMTPNTLYSSAAGYIHQELGKTVGCNTQKEF
CDNKKGKYAFKHPPKEYEEACICDTRQKAQKPIEKKNDCNGIKTLLDRSNGGTGGIDG
CNPKIGNYPSWNCERNESKAENKGACVPPRREKFCVSLLAKEGIFKNKGEDIRETFVK
SAALETYFAWKRYNDDNKKAEEELKSGTIPENFKRQMYYTFADYRDIFFGTDITSHDH
ILDVSKNAKNKLKEKNGEQKSVIIIDDEKLLADWWKEHGHEIWEGMLCALTHEIDEEE
KNKIKSTYSYDQLKKTTNGTTPLEKFAERPQFLRWFTEWSDEFCREREKKEEVVEKKC
KKDHEGCNKPNTKGNHGCVSACKDYEEYISTKKKQYNTQKEKFDIDKNKGNEEYENYK
DKEAHDYLKDKCFPGTCDYMEKVKNNSEYWDKPNKTYTNSDLEKKCECKPQPPPPAPA
PTQSACEIVDDILNGKSATDYIEKCNGKYKYGRYPEWNCNSQIHRTHNGACMPPRRQK
LCVINLQYFKGKTTVDLREAFIKCAAVETFFLWHKYKEDNNGGEDLQNQLESGIIPDD
FKRQMFYTFGDYRDFLFGTDISKGHGIGSELAKKIDSLFKNIGGKNPGDLSRKDWWNE
NGPYIWKGMLCSLEKAWGKDTIKNKSNYNYHNVKFSDNRNGPDLETFAKRPQFLRWFT
EWGDEFCREQKKQLDILKKKCPKETCTNEGKKKECSDACKAYKEWLQTWKEHYEKQKI
KYENDKDSYTNDPDTKQSPQAYQYLNKKLEKICPSGNTSANCEYKCMKYPSSQNNNNM
PASLDDTPSDYKDTCECTKSQASSRNFSVRSEDGEDGPPPPRAPRQSLARSADNPSPR
PAPPGGPQPPSGTPDAGGARAETGPSPQQPPKPPAGNGGVARILQPIARVDQDEEEDE
DEEDDDEESGSEEGEGEDVDDSDSSEDENDEEDEDDSHHVDGGHQEEEPPDETEVVEE
TVAAPEVKPACEIVKELFNDTNKFKDACNLKYGGNNSRLGWKCIPSGDSTTTSSVNGD
RSQRHRRAAGEATGKSDASGSICVPPRRRRLYVGKLTQWASQRTQGGTSSQIVGKTAS
QPNSHPTLSPSSNPRDDGLRDAFIQSAAVETFFLWDRYKKLNTKKPDATLGGLPQIPL
AMGAINGYVPSGDDNNPQKKLEEGEIPEEFKRQMFYTLGDYRDILFGKNDIVIGNTGS
GASDKEMKAKEEKIKETIDKVFPNSVSTPPPTPATKPSDEKRKTWWEANGEHIWKGMI
YALTYKDNGEKKIVKDNEVYKKLWDEANKKPKETKYQYKNVKLEENSGAKPTQPPSPS
GDNTPTTLTNFISRPPYFRYLEEWGETFCRERKKRLEEVRKECRGEYPGEKYCGGDGH
DCTENGELKHTNMFADLDCRDCHKQCRKYRKWIDIKFEEYEKQKDKYQGELDKLNGNS
NGNNNCCKEIKKHTSASEFLKELKHCKDGQNSEDDTDKSEEDKKNNKIDFNKPLETFN
PSTYCETCPSNKVNCNGSGRGTRGKDPCTPHNEKGKSWESVFNANGGNSTEITVEMID
RREPLIKNYSKILEESGNSSDSLFKTSRLFKSVRDQQWECRYKDEKTDICKLKNFNDK

IDLNQYTTFKVFLEYWLQDFIEGYYILKKRKIIEQCKENGGETCNENSKNDCACVKGW
VAQKTTEWNQIKDHYNKKEYGNGYDMSHKVKNYFEKNENELRKWIDNYDVLKNNEEYE
VCNNGDKNCNFEGKKRKKDMVTLLLSRLQNEIKTCQNPPPSDANLLSAQNPAQCQESS
PVGDVEEDLLLEEENTENTVEAKKNMMPTICKDVVPQEPKAEDESGCKTDAPQPDVKE
EEEEKEEEKDKGDEEQSGAPSSPSPSEGTEEPPPAPEAPPSTPRPQPLPSDNTSDILK
TTIPFGIALALTSIALLFLKKKPKSPVDLIRVLDVHKGDYGMPTLESKNRYIPYRSGT
YKGKTYIYMEGDTSGDDDKYMFLSDTTDITSSESEYEEMDINDIYVPGSPKYKTLIEV
VLEPSKSNGNTPSKGDGNTLGDDMIPTTNTFTDEEWNELKHDFISQYIQSRLPMDVPQ
YDVSTELPMNITEVNVLDVGINEKPFITSIHDRDLYSGEEINYNINMSTNTNNDIPKY
VSNNVYSGIDLINDTLSGNKHIDIYDEVLKRKENELFGTNHTKNTSNNSVAKNTNSDS
IMNQLDLLHKWLDRRRNMCEKWEKHNKEELLDKLNEQWNKDNDGINVPSDNRSLNTDV
SIQIDMDENKGKKEFSNMDTNVDTPTMDNILDDLETCNEPFYDIYEDDIYYDVNDENP
SVDDIPMDHNKVDVPKKVHVEMKILNNTSNGSLEPEFPISDVWNI
>PFD0615c, 2209 bases, B21A2120 checksum.
MAPGSTGTQDDDAKNMFDRIGQQVYDEIMKKDDADAKKYIKELKGKLSFASILGESAG
TDDPCQLESKYTELISGSGSGVAARGHPCGNVSGKGEDVSRFSKERVSKYDEKKIGCS
NSEGACAPYRRLSLCNKNFQKINNYSSKAKHNLLLDVCLAANHEGQSIKTHLKQYDAE
YPSGSGHTTCTALARSFADIGDIIRGKDLYRRDKGEKKKLEEHLKTIFGKIHSDVTSS
GSNKEALQERYNGDKENYYKLREDWWTANRETVWEAITCDDDDKLANASYFRATCSDS
DGKGSFSQANDKCRCKDKKGKNTDQVPTYFDYVPQYLRWFEEWAEDFCRKKKKYVNIV
KTYCRKKDNSSEERYCSRNGFDCEKTKRAIGKLRYGKGCTDCFFACYPYEKWIDNKKK
EFLKQKEKYINVINGTSSSSRKTRAARGSNVNGYEKIFYEKLKEGNVGNLDAFLGLLN
NEKACQDIKDDKEGGKINFKDDHGDINNNNKDEGTFYRSKYCQPCPYCGVKKNNNGGS
GGGNKWEEKHESDKCTRIKLYKPRSGQGGTPIKILKSGEGEKEIKEKIDDFCTKTQNG
TGDSNIDSSLCDPWKCYEIDELTKEGQEGEDDVDDRYYDELVETGGGLCILKKEKKEQ
EKEKSDAKSQNDPDEIQKTFYDFFYYWVAHMLKDSIYWRTKKLDKCLQNGNKKCGKKI
CNGDCECFQRWVEKKKTEWTNIKDHFVKQKGIPEGCYFTTLEGVLQIEFLNEGSAQDK
QNSLDAKEIQHLKQIKKILDEEKQKNQEETAGGCGPGVASDNKKETIMDKLIDYEKKI
ATECIEKHKCPDPPREGAGRSDTSRDSPSSRPAQEVGDSEEDEDEDEDDEEEHPDDGK
GDANEEEAENHSNDQEDKDTLDAVVENTEVGPSGPATPVDDKVCDIVSKLFSGNDFGD
ACGTKYDKYGREKFPNWKCIPSGDKTAPSSDSNQGSICVPPRRRRLYVGGLTKWASGN
TVVSGQAQTPQGDTTSPSDNKLRDAFIQSAAIETFFLWHKYKMDKEIEKKQQQKNGLV
ANTSNVGKEHQEKLEQSGIIPEDFKRQMFYTLGDYRDILFGKDISGDKNMDTIEEKIN
GILPKNGTPSPAKKNTPTEWWSQNGEHIWNAMICALTYDTNTASGDKPTQNEKVKEAL
WDEQNNKPKNDYQYSSVTIGGEGAEGQLQSTDSKDAARGEKTPLDSFIKRPPYFRYLE
EWGQNFCKERKKRLKDIKYECRGDENITRYGSGYGEDCKNNLPENPSTFKDLEYPTCA
KYCRFYKKWINTKKTEYEKQEKIYVQQKKDATSDNGNKYDSNCDGKLKQYASIESFLE
KLVQCKKDNGEGTIKFNGGQTFQHTEDCKSCSKFRIKCDNDKCSGGNTKVKCDGKTPI
DAKEIANMINSPQEVTMLVSDNGATGFKGDDLKEACEGKGIFEGIRKEQWKCDNVCGY
VVCKPKEGNRETVRGEKNDDKHIITIRALVTHWVQNFLDDYKKIKHKISHCTKTDQGS
TCQNKCQNKCKCVGEWIPKKREEWQQIKDRFLKQYKNDKLDEDFNLRSCLETFLVQIG
AAYGEDKFKKVIKLSVFDQSCGCSAIASSQKKNGEYKDAIECMLKKLEEKANKCKEDH
SSGEQTEKECQESPSVEDEDDTLHEETEVKAPEICKDVIKAPTEPEEKGACDPAPTTP
KETSPATDSGKETNTEPVTPQDQSPDTKTPKEKGPKPPKSLPQPPRQKREFTPSDWWK
VMSASAFPWTVGVAFMALSYWVMKKKSKPRVDLFSVMEIPQNDYGMPTLKSKNRYVPY
SSGKYRGKTYLYVEGDSGTDSGYTDHYSDITSSSESEYEELDINDIYPYQSPKYKTLI
EVVLEPSKRETNSGDIPNDNTPANKPITDEEWNTLKDEFISNMLQSTQNTEPNILHDN
MHYNTHPNTLYFDKHEEKPFIMLIQDRNLLIREEYSYNMSTNSGGNGSYSGISPISDN
PDSLSDKNGPTSGNHNLYSGTDLINDALSGDYDIYDEMLKRKENELFGTNHTKHTTTN
RVATQICDDPITNQLNLFHKWLDRHRNMCEKWENHHERLDKLKELWDNETHSGNKHNG
IQSNIPSSDIHPSDIHSGKLSDTPSDNNIHSDIPYVLNSDVSIQIHMDNPKPTNEDNV

VDCNPVGNNIYVDNNPNQTFPSNPNPVENNTYVNAPTNVQIEMDVNNHKVVKEKYPIS
DMLDI
>PFD0625c, 2277 bases, A473F6DB checksum.
MGPPSTAPDYSSAKDAKDLLDMIGKDVHDQVEKEAAGRGGSELKGLLSLAKGSGVELA
AFPEPCGLIKDKGDELLGDSGERHPCGNTTGKEDVDRFSVKQQAEYDNKKMKCSYGSN
GTDVGACAPYRRLFLCNKNMEKMGRTSTTKHDLLLDVCMAANYEAQSLIRYHDKHQLT
NEGSQICTVLARSFADIGDIIRGKDLYLGKKKKKKTETERDQLESKLKKIFGDIYNEL
TNGRNGVKDHYQDDNGGNYFQLREDWWTANRATVWKAITCKADTGNAYFRPTCSDSDG
KGSFSQANDKCRCKDKKGKNTDQVPTYFDYVPQYLRWFEEWAEDFCRKKKIYVGIVKT
YCREKYKSGNEPRYCSRNGYDCTKTKRAIGKYRMGNQCISCLYACNPYVDWINNQKEQ
FDKQKQKYDKEIKIYKNGASGIRRQKRGTTTKYEGYEKKFYDKLEKNNYGTVGEFLGL
LNNEKACKEVKDGGTIDFKQVNSTSGGTAVSASGASSTSGGSGAASGGTSDTSGTNNA
SQGTFYRSEYCQPCPHCGVRKANNGNFVKKSDSEQCKNINLYRPKKPEEGTKIEILKS
GEGETEIKEKLEQFCQTQNATGGVANGSGSGTSGSQKLYEDWKCYNDVEKDGQDGVDD
DDDLEYDRLVNSSGGLCILQKKNGEENGKKQKTYNDFFNFWVAHMLKDSIHWRTKKIK
GCLKNGKAIKCTDKCKGDCKCFERWVEQKREEWTNIKEHFGKQTDIPTGLTPDALLEG
VLEKGVLLTSIKEAYGDAKETEHIKQLLDETAVAGGVVVGAKDNTTIDKLLEQELKDA
NRCKNCEQRKPPGEEGGAARNLPGVDTTVDDANEDDLDDEDDEDEDDDGGGSDVGGSD
VGEVEEETAKEATEETTTPLDVCNTVKTALEGDLGEACRQKYEYGREKFPNWKCISGD
NTRGSESESAGPSRSKRHTESSDSAVTATGSSGEATGKSGDKDGAICVPPRRRKLYLG
GFKRLTDGTAVSSEATQAGTPSQSPKGDALLLTAFVETAAVETFFLWHKYKQEKKKPK
NEVGGAAGVLQTIGGTLENSGEQNPQKKLQESGEIPDDFLRQMFYTLGDYRDILVRGV
ADDKNGGNNIILNASGNKEDMEKMKKIQQEIDKILEKSGSEAASGAQKNSGISREKWW
DKHAPSIWDGMVCALTYNTDTASGTAPTQIQEVKNALLDGEGKKPKQNGTNGKDYTYG
GVRLEDENSGTQALSPNAPASTASQTTQSSSTSENTPTTLTNPKLKDFVLRPTYFRYL
EEWGQNFCKERKKRLDQIYRECKVDEDGPRDGKKCSGYGEDCKDNLSKKYDTLPSLEC
PGCGRHCSFYKKWIKIKKDEYEKQQKAYNEQRTNYTNKNKVSESNNHDKEFCTNLETK
YTDAANFLQRLKDGPCKNNSEEDQKVNGYIKFDDISKDKTFGHENYCDPCSKFTVNCN
RNDHCDNSNGNNCKDNKITAEKIGNGVDSTVLDMRVIDDSATGFKGDGLEEACGSANI
FKGIRKEQWKCGKVCGYNVCKPKEGNRETVRGEKNDDKHIITIRALVTHWVQNFLEDY
KKIKHKISHCTKTDQGSTCQNKCQNKCKCVGEWIKLKQQEWEEIKKRFLNQYKMDSDE
YYPVRSVLETFLVQIGAANANNDVKKLIKLSEFYKSCGCSAKTNSENNKNEDAIDCML
DKLGKKAEKCHDQHSDNPQEKCDEPPPELDEEDLLLEEEQNPKNMRPGFCPQNDTTEQ
QEEEENICTPAETVKKEEEEKEEQEEEEPDEKVPPPRAPEASKPKKEKPSQPPRPRRT
LELLDNPHVQTALVTSTLAWSVGIGFAAFTYFYLKKKTKASVGNLFQILQIPKSDYDI
PTLKSSNRYIPYASDRYKGKTYIYMEGDSSGDEKYAFMSDTTDVTSSESEYEELDIND
IYVPGSPKYKTLIEVVLEPSGNNTPTSDIPSDIPNSDTPPPITDDEWNQLKKDFISNM
LQNTQNTEPNILHDNVDNNTHPTMSRHNMDQKPFIMSIHDRNLFSGEEYNYDMFNSGN
NPINISDSTNSMDSLTSNNHSPYNDKNDLYSGIDLINDALSGNHIDIYDEMLKRKENE
LFGTQHHPKNITSNRVVTQTSSDDPITNQINLFHKWLDRHRDMCEKWKNNHERLPKLK
ELWENETHSGDINSGIPSGNHVLNTDVSIQIDMDNPKTMNEFTNMDTNPDKSTMDTIL
DDLEKYNEPYYYDFYKHDIYYDVNDDKASEDHINMDHNKMDNNNSDVPTNVQIEMNVI
NNQELLQNEYPISHM
>PFD0635c, 2326 bases, 12A4625E checksum.
MAPTSGGGGTKDESAKHALDSIGKRVHAQVQNEAKQRSNGDLKGLLTSATLSGGESAF
TENPCELIKEKREKLLGARGERNPCGNGSASEKRFSEVSGGECDDKKIEGNGRNNGGA
CAPYRRLSLCNKNFQKINNYDSSKAKHNLLVDVCMAANYEAQSLIPYHDQYDATYPGS
DFSMCTMLARSFADIGDIIRGKDLYRGKKKKKQNGKETETERDQLESKLKEIFGDIYN
ELTNGRNGVKDHYQDTTDYFQLREDWWTANRHTVWKAITCGTHEGDTYFRPTCSNRQG
PSQAHHYCRCNGDKPDDDKPNTDPPTYFDYVPQYLRWFEEWAEDFCRKKKKYVDIVKK
YCRGVYNDVPRYCSRNGYDCEQTIYKKGYFVIDKGCINCLYACNPYVEWIEKQKEQFD

KQKKKYTDEINEASRSSRRQKRGARSTGSSSNYDGYESKFYNILKDDYGTVDDFLKLL
NKEKSCQAVKDNDGGTINFTEKNDDKNNNNKDKGTFYRSEYCQVCPDCGVKYNGSGWE
EKKKNDQCNIKLYKPKKDAPHTPIKILKSGEGKEEIEKKLEAFCDKKDGGNSDSSLYD
PWQCYQFDQLEKDEKEEGVDDRNYDNDVRTGGGLCILPNQKKNKEEGANTSEKDHDEI
QKTFNPFFYYWVAHMLKDSIHWKKKLEKCLQNGTKTRCRNNEKCNKECECFQRWVEKK
KTEWGKIKDHFKTQNIGDETNCDPIVTLEGVLKLQFLNEDSTQDKQNSLDSEELKHLK
HLSEMLQETSGDGLTCGASDNEKETLMDKLIEHEEGIAKECLRKQNECEKKAKPEGRS
DSHDDPQPPDDADNEDDLDDEDDEDEEEEVQVEDNTQEEGEQPVVPQQEEGSSSPTPA
PAGPDVCDIVSQLFSDPSQFSDACTLKYVTGKNYGWKCIPSGNTSDTTGSESEATGAR
QRRDTDSSGDTTGGKDGATGGLCIPPRRRRLYVGGLSQWASQRTQGETSSQSGENLLE
AFIQSAAIETFFLWHKYKAENTKTQGVGAGGADFLPATSSVATALAPGAVPSRPSLQL
LSGVGVPGEPGMAPGVKSIPVPPLGVGVGGIPGVGALGGGALGPGGPVGLDGVPGQAQ
PLTLLRPGVLGNGLQSPQSRLRTLDGHFAGGESEDKTPQQWLQQGHIPPDFLRLMFYT
LADYRDICIGGDRDIVGDTIVSNTEGSSSSKIKISEKIKEILNHDNKQEPAPKPSVEK
TTPTEWWSQNGQHIWNGMICALTYTDSGGSITEDKDVRDKLIDKDTGKPQKNGDNDYT
YEKVELKDDESGPKGNDTIQPATLKDFVEIPTYFRWLHEWGSDFCGKRARMLKDVKDN
CRNIDKAGHHYCSGDGYDCTRDVIERNDKFVDLNCLGCYKQCRKYKKWIDIKFVEYHN
QEKKYKDEYGKLTKDKSSDDKKLEGYKCAENFLKELKHCKPSEDNNDQDNKINFDKPE
KTFNPSKYCKACPVYGVKYIGGNYIPNEEKDYKSKKGRVKKENDTIPKNIEVLVLGRK
GEEKDKDKHLHDACKNTGLFEVARYEQWNCQKKKGIDQCKITKFANDIDFDKDIVFNE
FFQRWLRYFVQDYNKLKDKINPCTKKETEKEKEKSYKCTQGCNDKCECVKEWLSKKKQ
EWTQIKTLYKQYSKISDQEIAFRVKSYFVDQGLFDNDYKKAQEVVEKPCDKEKLWGCT
GDNLKEGEDPGKCHMGDFITNLISKLQKKIDDCNKNQAQNSVETQPSDENTAQCQDTH
PDDEEDLLLEENENQVAQPNICPNQVEDKKIEEEVEKCETAQTTAEETAAAGGERQTP
PAPAPAAPPSPPRPLPKPKPPKPDLPPALKNAMLSSTIMWSVGIGFAAFTYFFLKKKT
KASVGNLFQILQIPKGDYDIPTLKSSNRYIPYASDRYKGKTYIYMEGDSSGDEKYAFM
SDTTDVTSSESESYEELDVNDIYVPGSPKYKTLIEVVLEPSGNNTPTSDIPSDNTPTPQ
PITDDEWNQLKHDFISNMLQNTQNTEPNILHDNVDNNTHPTMSRHNMDQKPFIMSIHD
RNLFSGEEYNYDMFNSGNNPINISDSTNSMDSLTSNNHSPYNDKNDLYSGIDLINDAL
SGNHIDIYDEMLKRKENELFGTQHHPKNITSNRVVTQTSSDDPIHNQLNLFHTWLDRH
RDMCEKLKNDNERLAKLKEEWENETHSGDINSGIPSGNHVLNTDVSIQIHMDNPKPIN
EFTNMDTSPDKSTMDTIIDDLEKYNEPYYYDFYEYDIYYDVNDDDKTSMDNNNNLVNK
NNPVDSNSSTYNHHNPADINKTFVDINNHNQHPIEKPTKIQIEMNSNNREVDEQQYPI
ADIWNI
>PFD0995c, 2152 bases, EE782057 checksum.
MVPPGGRQGGSGEDGIDDKDAKHLLDSIGKIVHEQVKNDAKTYKGELEGKLSLATTIG
ELNYTKDPCIFDYSKLINGSGSGGVTARDDPCGNGSGKGEDVSRFSDKEGAQCANSKI
HGNSKGSNGGACAPLRRLHLCNKNMEKIATSTAKHDLLVDVCMAANYEAQSLIRDHPQ
HKRTNPDSKICTELARSFADIGDIVRGRDLYRGNKKENKQREKLEENLRKIFENIYEG
LSNNGVKARYEGDKENFYQLREDWWALNRDQVWKALTCSDDLKDASYFRPTCSDRKGS
CSQAKDNCRCDGSNTDQVPTYFDYVPQFLRWFEEWAEDFCRKKKKKLEKLEQQCRGVY
EGKERYCSRNGFDCEKTVNARGKVRMGKGCTDCFFACNPYIDWIEKQKEQFDKQKQKY
DEEIKKYTKVASSSSGGRAKRAARGGSNVNGYEKIFYKKLKDSGYESVGEFLDLLSKE
EVCKNFKEKEEGKIDFKTVKSSSAKNSDDSNKTFCRTKYCQPCPLCGMKKNNNGEWEH
KKKGKCTSGNLYRPIDGAIHTDINFLYSGDRHDDIEKKLNRFCDETNGDTINSVAGVS
GTGVVAGSNSRSKELYQEWKCYKGKDVEKVKDGKDEDDEDYEDDYHKEVENAGGLCIL
ENNDGKEKVNKQKTYNDFFYYWVAHMLKDSIHWKKKLEKCLKNGTKTRCKNNKCNREC
GCFQKWVEQKKKKEWGKIIEHFYKQEGIPPGTHDITLEGVLKKEVLLTSLQEGYGNAE
DIEHIKQLLEEEENEENEGTPGADNKNKNTIDKLLDHEEGIAKECQQKQNECPKKPPK
KTPGGPDRALKPEEVDASSEDHYDEEGGEDEEEEEDKEEEGEDGEDVQDDVAEEDTAK
EEGSSTTETQLPDACNIVKTLFESTKNFEDACRQKYGPKAPTSWKCVPTTGDKDGATG

KSDGSICVPPRRRRLYVGKLHDWASGNTQEDGKAQPQGDTPSQPDPLLKAFVESAAVE
TFFLWDRYKKLNTKRQGVGAGAPQLQEEDDNDEEEENPQQELEDGTIPDDFLRQMFYT
FGDYRDIFFGNNIGSGNDVEKVKPNIDKVFENSEKPTSGGHNSEQQRETWWEEYGPHI
WDGMICALSYNTKDKEIIKLAHHNLTSKEIYKYNNVTFIGGFNSDKNSKTETSATKLE
EFSRRPTFFRWLEEWGEDFCRKRNDKLKNVKKECRGKYPNGDKQYCSGDGHDCEKTYL
KHNDMFADLNCRRCGEQCRNYKQWIEKKLEEFHKQKSIYEKEIPKLKDNYNNHHYKNF
YEQIIEKNGYSSFKKFLESFNQRKVCQGNSDQTNNTDFNEPLKTFSLSTYCKTCPLNG
LNCGSKECNEVKGKGATWESVLNGKSKDYRRTTGINVEMINRRGKYIEKHTQDSLFKE
SSLLKSVRDQKWECTFINDKMDICKLNNYNESIDMNKYTTFKVFLENWLQDFLEGYYI
SKRKIEICTENGENKCIKGCKGKCECVKEWLNKKSTEWKQIKERYKVHHDSKGYDIAH
KVRSYFEKNESDVNKSIDNYEVLKKKDEYEDCIDSDTCGPKNNWKKKDMVSILLSELQ
HNMNKCKTLHDPSGKPEAQCVEPTNPLDDENPDPEDDTSTTSSRPKFCPEIPQPEPEP
EPFPEEDTEQEEKEEKLPAAPDNSEQEETSKEVVPEKKMPTPPPKKPEQGPKQRKKQK
RQLPTHTSILPEMLSISSFPLTVGVAFAALSYFLLKKKSKSTIDLLRVIDIPKGEYGI
PTSKSKNRYIPYASDRYKGKTYIYMEGDESDDYTYIGDISSSDITSSESEYEDIDINN
IYPYKSPKYKTLIDVVLEPSKRDTFNTQSDIPSDTSTNKFTDNEWNQLKQDFISNILQ
STQMDLPNENIIDDFMDKGIQPNNPVLDVNMAEKPFITSIHDRDLHNGEEVTYNINFD
VSKNINEITNTTDDSKYVSNNIYSGIDLINDSLNSDQHVDIYDELLKRKENEIFGTNH
TKHTTTNSIAKQTHTDPILNQLDLFHKWLDRHRNMCEQWNKNKKEELLDKLKEEWNKK
NNNNSDLTHTSSNIPSGENSIKNVLNTDVSIQIDMDDPKPINEFTYMDNIIDNLEKNS
EPYYDIDEDDIIYFDIDDEKTPMYHNNMDNNKSNVPTKVQIEMNVINKQELFQEEFPI
SDIWNI
>PFD1000c, 2201 bases, C010679E checksum.
MVPPGGRQGGSGEDGIDDKDAKHLLDSIGKIVHEQVKNDAKTYKGELEGKLSLATTIG
ELNYTKDPCIFDYSKLINGSGSGGVTARDDPCGNGSGKGEDVSRFSDKEGAQCANSKI
HGNSKGSNGGACAPLRRLHLCNKNMEKIATSTAKHDLLVDVCMAANYEAQSLIRDHPQ
HKRTNPDSKICTELARSFADIGDIVRGRDLYRGNKKENKQREKLEENLRKIFENIYEG
LSNNGVKARYEGDKENFYQLREDWWALNRDQVWKALTCSDDLKDASYFRPTCSDRKGS
CSQAKDNCRCDGSNTDQVPTYFDYVPQFLRWFEEWAEDFCRKKKKKLEKLEQQCRGVY
EGKERYCSRNGFDCEKTVNARGKVRMGKGCTDCFFACNPYIDWIEKQKEQFDKQKQKY
DEEIKKYTKVASSSSGGRAKRAARGGSNVNGYEKIFYKKLKDSGYESVGEFLDLLSKE
EVCKNFKEKEEGKIDFKTVKSSSAKNSDDSNKTFCRTKYCQPCPLCGMKKNNNGEWEH
KKKGKCTSGNLYRPIDGAIHTDINFLYSGDRHDDIEKKLNRFCDETNGDTINSVAGVS
GTGVVAGSNSRSKELYQEWKCYKGKDVEKVKDGKDEDDEDYEDDYHKEVENAGGLCIL
ENNDGKEKVNKQKTYNDFFYYWVAHMLKDSIHWKKKLEKCLKNGTKTRCKNNKCNREC
GCFQKWVEQKKKKEWGKIIEHFYKQEGIPPGTHDITLEGVLKKEVLLTSLQEGYGNAE
DIEHIKQLLEEEENEENEGTPGADNKNKNTIDKLLDHEEGIAKECQQKQNECPKKPPK
KTPGGPDRALKPEEVDASSEDHYDEEGGEDEEEEEDKEEEGEDGEDVQDDVAEEDTAK
EEGSSTTETQLPDACNIVKTLFESTKNFEDACRQKYGPKAPTSWKCVPTTGDKDGATG
KSDGSICVPPRRRRLYIHKVDDNVKDDASLRKWFIESSAVETFFLWHEYKMEKKREDI
EKQKANEKVVDTSNVGEELQNDLEGNGTIPEEFKRQMFYTLGDYKDIFEGKSIEVGDE
KDKQKMKEIEKKIQAHINSGSSSPPHGTPGQPNSVTTPQQTWWSRNAPSIWKGMICAL
TYKEDGEKSTDDKTTLKRNDDVYEKIFGKPPNNDNPQNPNNGTFHKKYHYDSVRLKDD
DDQSGDKLQSTSAPSDTPTLNNPKLSDFVLRPTYFRYLEEWGESFCGTRKRMLEQLEK
VCRSGETGKEHCSGDGHVCEKDYLNHNNMFADSYCPDCKKACRKYKKWIEKKLEEFQK
QKDKYKGELDKLTKDKSGGDKKFCEEIKNHSSAANFLKELKHGKDNQGNSDQDNKLDF
ENIPQTFSRSTYCKTCPPNKVNCSSGSKSRTSGGTNRCTEVKKNGETWEKVFDKIAKN
NGKTTTIDVHMIDRRAPFIKKYLENSKNSEESNNSLFKDSYLFKSVRDQNWECKFENE
NKDVCKLKNFNDKIDLNQYTTFKVLLIYWLEDFIEGYYILKKRKVFEQCKENGENTCS
EESKNYCACVKVWLEKKKNEWDQIKKHFKDRKSDDGDTVVSKVRNFLETLIPRIAPKK
NNGEVTELSDLEKSLGCNCAGRAENSKEDEKEDVVLCLLTKLEDKANKCKEDQKPNGE

NQAQTCEKSAPVEDEDDEPLEEENPVTQPNICPKVETTEETVDEGKCEEDTVTPSLGP
KDDSEKDEKKEENSEDTTAAEGEENGAPGSSGTPPPPPAAPPSTPAKTKESKKPKSTK
KPRIKTLNVLDHPAVIPALMSSTIMWSIGIGFATFTYFYLKKKTKSSVRNLFQILQIP
KGDYDIPTKLSPNRYIPYTSGKYRGKRYIYLEGDSGTDSGYTDHYSDITSSESEYEEM
DINDIYVPGSPKYKTLIEVVLEPSKSNGNTLGDDMVPTTNTFTDEEWNELKHDFISNM
LQNQPNDYKSEDIPLNTQPNTLYFNKPEEKPFITSIHDRNLYSGEEYSYNINMSTNSM
DDIPINRDNNVYSGIDLINDTLSGNQHIDIYDEVLKRKENELFGTKHHTKRTSTQNVA
KPARDDPIHNQLDLFHKWLDRHRDMCKKWNNKEGLLDKLKEEWNKDTNSGKLSDNIHS
DNKPGDIPSDNHVLNSDVSIQIDMGNPKYINQFTCVDSNPNLTLRSNPNLMGNQNPNL
NLVENNINPNHQNQNQVGDTNFVDTPTNPTNVQIEMDVNTKLVKEKYPIGDVWDI
>PFD1005c, 2181 bases, 4B98EF82 checksum.
MVARGGHQGGEDIDETSAKHLLDSIGEKVYKKVHGAALQRSNGALQGILSLATFEKKP
ETQQTPKDPCDLNHKYHTTVTSGYDKENPCKDRPEVRFSYTEGAECNKSKVKGNEGNS
EGACAPYRRLHVCDQNLELIKPKNITTHNLLVDVCLAAKFEAESLKTYRGKYQLTNHG
FHTNICTELARSFADIGDIIRGKDLYRGNNKEKDRLEDNLKKIFKQLYEELTKNNKNE
AIKTHYQDDDPNYYKLRNAWWEANRQEVWKAITCGAGGSKYFRHTCGTGTPTDDKCRC
AINDVPTYFDYVPQYLRWFEEWAEDFCRKRKHKLKDVKTNCRGENGTDRYCSGNGLDC
TKTIRAKYIYAIGSECTKCSFLCGFYKKWIDNQKKEFLKQKKKCENEMLSKSKKKQST
KYNVYEGYDKEFYKILKSNNVGNLDNFLDLLSNENECKNIDGKGGKIDFNNTDNKTFS
HSEYCEECPDCGVEKKDNGEFQKKEKNNGECKGGKRYEIPNHAKFNEINVLSFGDKGE
DRETKLKKFCETKNGSAGGGGGSNSEIKELTEQWKCYEEKDIKKLVENDLEYNKEVKG
SGGICILQKTNGEENGKKQKTFNDFFHFWVRHLLNDSIEWREKLDKCLKNGTKILCKN
GCNKNCKCYESWVEEKKKEWGEIKKHFDTQEDIKNSTGVDPIVTLEYVLEEFYFPLIQ
EAYGDAQAIEGIKKTLHSKENQQTDATDTKNKTLLDYLLHHEEQDADKCVRNNPDKDC
PKKPPTGGPGGVHSDTASEDITHHDSSHDESEGSEDEEEEEEEDKEEEGEDGEDVQDD
VAEEDTAKEEGSSTTETQLPDACNIVKTLFESTKNFEDACPTKYGPKAPTSWKCIPSG
DKAATSGEGDSGPSRKRRDTTGGESTTSSSATTGGKDGATGGSVCVPPRRRRLYIHKV
DDNVKDDASLRKWFIESSAVETFFLWHEYKMEKKREDIEKQKANEKVVDTSNVGEELQ
NDLEGNGTIPEEFKRQMFYTLGDYKDIFEGKSIEVGDEKDKQKMKEIEKKIQAHINSG
SSSPPHGTPGQPNSVTTPQQTWWSRNAPSIWKGMICALTYKEDGEKSTDDKTTLKRND
DVYEKIFGKPPNNDNPQNPNNGTFHKKYHYDSVRLKDDDDQSGDKLQSTSAPSDTPTL
NNPKLSDFVLRPTYFRYLEEWGESFCGTRKRMLEQLEKVCRSGETGKEHCSGDGHVCE
KDYLNHNNMFADSYCPDCKKACRKYKKWIEKKLEEFQKQKDKYKGELDKLTKDKSGGD
KKFCEEIKNHSSAANFLKELKHGKDNQGNSDQDNKLDFENIPQTFSRSTYCKTCPPNK
VNCSSGSKSRTSGGTNRCTEVKKNGETWEKVFDKIAKNNGKTTTIDVHMIDRRAPFIK
KYLENSKNSEESNNSLFKDSYLFKSVRDQNWECKFENENKDVCKLKNFNDKIDLNQYT
TFKVLLIYWLEDFIEGYYILKKRKVFEQCKENGENTCSEESKNYCACVKVWLEKKKNE
WDQIKKHFKDRKSDDGDTVVSKVRNFLETLIPRIAPKKNNGEVTELSDLEKSLGCNCA
GRAENSKEDEKEDVVLCLLTKLEDKANKCKEDQKPNGENQAQTCEKSAPVEDEDDEPL
EEENPVTQPNICPKVETTEETVDEGKCEEDTVTPSLGPKDDSEKDEKKEENSEDTTAA
EGEENGAPGSSGTPPPPPAAPPSTPAKTKESKKPKSTKKPRIKTLNVLDHPAVIPALM
SSTIMWSIGIGFATFTYFYLKKKTKSSVRNLFQILQIPKGDYDIPTKLSPNRYIPYTS
GKYRGKRYIYLEGDSGTDSGYTDHYSDITSSESEYEEMDINDIYVPGSPKYKTLIEVV
LEPSKSNGNTLGDDMVPTTNTFTDEEWNELKHDFISNMLQNQPNDYKSEDIPLNTQPN
TLYFNKPEEKPFITSIHDRNLYSGEEYSYNINMSTNSMDDIPINRDNNVYSGIDLIND
TLSGNQHIDIYDEVLKRKENELFGTKHHTKRTSTQNVAKPARDDPIHNQLDLFHKWLD
RHRDMCKKWNNKEGLLDKLKEEWNKDTNSGKLSDNIHSDNKPGDIPSDNHVLNSDVSI
QIDMGNPKYINQFTCVDSNPNLTLRSNPNLMGNQNPNLNLVENNINPNHQNQNQVGDT
NFVDTPTNPTNVQIEMDVNTKLVKEKYPIGDVWDI
>PFB0985c, 229 bases, DB6FFAD2 checksum.
MFHYIYKIYIFTIILCASNLFNNNVVEIGTYKLSYHNGGIQFRMLAQKNTNKKPFGNT

LTNILFKDKKKKNLDPQISSLVSLVDNMDITQEKKDKIKNLSLKYINSRDVKEKNESI
NELQKYSNNEECKEYMDSYLMHLRMQNDIKCLKRKNLWNNIWIVSTTLLLIIIMIACI
IVCTPETYTALYPAFILLIFIIHIVARYFPDMKIGFKKLKTKLNTFFQNKKQITK
>PFD1015c, 2193 bases, 723BC224 checksum.
MAPQSSGGSEEDDIDHKSVKHLLDSIGKKVHDKVKSEANQYKDDLKGNLQHAKDMGER
ASSNKTCDLVKEYYEHFNGDASDKRQPCKKDTNGNDVERFSVKQQAEYDNKKMKCSNG
SNGKNEGACASFRRLNLCNKNMENMDTNNNDGKAKHNLLAEVCLAAKYEGDSLKHYSK
KLNLTYTDSPSQLCTELARSFADIGDIVRGKDLFLGHKQRKKELEKRLVEMFKNIQGN
NSKLRTLSLNHVREYWWALNRDQVWKAITCDADTGNAYFRTTCSDSHRSGTFSQAKDK
CRCKDENGKNETDQVPTYFDYVPQFLRWFEEWAEDFCRKKKKYVDIVKTYCREKDKYG
NERYCSGNGYDCTKTIYKKGRIVVGYECTSCSVWCRMYEKWIDNQKKEFLKQRNKYQT
EIPVNGRKRRSTTSNYKGYEKQFYEEFKRNYGDVNKFLQLLNKEATCKTIGDEKEKID
FTKNVEDHKNINSQGTFYHSQYCEVCPGCGVKHNGSEWKEKNNGQCKGGKRYTIKDNA
PSTNIDVLFFGDKRDQIKSKMEKFCNQTNGSSGDCGGTNSDSSLCEPWQCYEGKHVQK
DQNAQEDDDDGEYDKDYTNIENAGGLCILQNKNRKEKKSEKEPEQFQKTFNEFFYFWI
VRFLNDSMYWRGKIGGCLKNKSEKCKNECNTNCKCFLKWIGKKKTEWGKIVEHFNTQK
NLPMDHVLILKFVLKLEDLFNNIKSGYGNVKELKGIKNMLKEEEEKNKEEEEAGASGG
ENKNTSIDKLLKHEEDEANKCLKTYKENCQQQENTSGGRTGEPRPAATDPTASTGSGD
DEDDDDDDDDEDDDDDDDDEDEEEAEAAEGDTEAENPEQGEEEEKGPSQEDPKVCETV
DKALTGDTTALKDACPTKYGPKAPTSWKCIPSGDKAATSGEGDSGPSRKRRDTTGGES
TTSSSATTGGKDGATGGSVCVPPRRRKLYVGGLTKWAEIQLKSQAGGDKATQEGDGNG
GSETQPQGGTPSQPDPKVELLKAFVESAAVETFFLWDRYKKEWMAQKLAEQGLTGGLP
GLSSSVLGEEEEQPPQSKLQQTGEIPPDFLRQMFYTLGDYRDILVGNSTHILEAVTIG
SNNETGKEIMKAIQEKIQQILPQNGDKPGKTSGTTPQALWSKYAEPIWNGMIYALTYK
DNTDSGAKGKPPTQDTQVKTKLWDENSKKPKKNAGPESNIDYTYENVVLKEDESGAKT
TGGDTPLTNFISRPTYFRYLEEWGETFCRQRTKRLDKIKEDCEVDDDDNKCDGDGFYC
TQKVTNEDETIKGFDCSTCARHCRFYKKWIERKKDEFTEQKNAYSKQKEKCKEGSESS
KQFCGTLKDDAAAFLNKLKSGPCKNESGEGPIKFDDQDKTFKHTEYCGPCSQFTVDCK
NCNGGHTKGKCKNKTITEEDIKTMNDHNHVVMGVIDNSAKGFDDGLEACRDKCIFEGI
RNDVWKCGKVCGLDVCGLKNANGKNYDQIILIRALFKRWVENFFDDYKKIKHKISHCI
NNGNGSICTSDCGKKCNCVKDWITKKKDEWKNIKERFREQYKKPDNYNVRSVLEEVIP
ENHLVNTKNKVIKISKFDNSCACSASAISTNGNEEDAIDCMIKNLEKKIDDCNRNHNP
SDKECNETLAQTPDETFDNDIEIEEAKKMVPKFCKIEEETKEAVDEEGECKASSPAEP
KVQEEKEEQKDKKDEETEQAAPTGDKGTKELPLTPRPRPGPQPKPPPQVEKNPWEHPI
VIPALVTSTLAWSVGIGFAAFTYFYLKKKTKSSVGNLFQILQIPKSDYDIPTKLSPNR
YIPYTSGKYRGKRYIYLEGDSGTDSGYTDHYSDITSSSESEYEELDINDIYAPRAPKY
KTLIEVVLEPSGNNTTASGNNTTASGKNTPSDTQNDIQNDGIPSSDTPMNKFTDEEWN
TLKDEFISQYLQSEQPNDVPNDYSSGDIPLNTQPNTLYFNKPEEKPFITSIHDRDLYT
GEEYSYNVNMVNSMDDPKYVSNNVYSGIDLINDALNGDYDIYDEILKRKENELFGTNH
TKKNTSTNSVAKNTNSDPILNQINLFHTWLDRHRDMCEKWENDNERLAKLKEEWENET
HSGNKHSDIPSGNHVLNTDVSIQIHMDDPKPINQFTNMDTILDDLEKYKEPYYDVQDD
IYYDVNDHDTSTVDTNAMDVPSKVQIEMDVNTKLVKEKYPIGDVWDI
>PFD1235w, 3553 bases, 58745069 checksum.
MGNASSSEGEAKTPSLTESHNSARNILEGYAESIKEQASKDAKIHGHHLKGDLAKAVF
RHPFSAYRPNYGNPCELDYRFHTNVWHRNAEDRNPCLFSRAKRFSNEGEAECNGGIIT
GNKGECGACAPYRRRHICDYNLHHINENNIRNTHDLLGNLLVMARSEGESIVKSHEYT
GYGIYKSGICTSLARSFADIGDIIRGKDLYRRDSRTDKLEENLRKIFANIYKELKNGK
KWAEAKEYYQDDGTGNYYKLREAWWALNRKDVWKALTCSAPRDAQYFIKSSVRDQTFS
NDYCGHGEHEVLTNLDYVPQFLRWFEEWAEEFCRIKKIKLGKVKEACRDDSKKLYCSH
NGYDCTKTIRNKDILSDNPKCTGCSVKCKVYELWLRNQRNEFEKQKKKYYKEIQTYTS
KDAKTDSNINNEYYKEFYDKLKNEGYETLNKFIKLLNEGRYCKEKISGERNIDFTMTG

DKDAFYRSDYCQICPECGVQCSGTTCTPKKVIHPNCKDKETYEPGDAKTTDITVLYSG
DEEGDIAQKLQDFCNDKNKENDENYEKWQCYYKSSEINKCQMTPSSHKVPKHGYIMSF
YAFFDLWVKNLLIDSINWKNDLTNCINNTNVTDCKNDCNTNCKCFENWAKTKENEWKK
VKTIYKNENGNTNNYYKKLNNHFQGYFFHVMKELNKEEKWYKLMEDLKEKIDSSNLKN
GTKDSEGAIKVLFDHLKDIAERCIDNNSKDSCPPSVDTKTNPCAKPPGSKPTKSVKQL
AEHMQQKAQKLLGTRGGESKLKGDATRGTYNLGGQGNTLNGDICKITKNHTNDSRPNG
EPCTGKDKVKNGFRLKIGTPWTNIVQKKKKKSYKDFYLPPRRQHMCTSNLENLSTSSK
GLSNGSFASHSLLGDVLLAAKFEAQKIILVYKNKNNINIRKRITDPNDQATVCRAIRY
SFADLGDIIRGKDMWNINSDAKDLQDRLEKIFKTINEKLPNEIQKRYTNRENKHLDLR
SDWWEANRHQVWRAMKCATKGISNNNCNGIPIEDYIPQRLRWMTEWAEWYCKKQSQEY
EKLEEKCGMCTGKGQGDGKDCTQKDKECSPCKKACDAYKKEIEKWEKQWKTVSAIYQI
LYAKARIVASNGGPGYYNTEVQKKDRSVYDFLYELHLQNGGKKGPPPATHPYKSVNTR
DKRDATDDTTPTVYSTAAGYVHQEAHIGDCKEQHVFCDNNGNKEKYAFKNPPNVYVEA
CKCMTREAPPPPTTPSTPNPCAETGGVHTIKTVTDVAKILQGEANETMLKNSSNGNDK
DESKLKGKAEEGDYSRGGTPSDFNNNLCGITQKHSNAHNDSQQPCYGKDQKRFNVGTE
WSFKDNHRKRTHPEAYMPPRREHICTSNLEYLIHKRKKPIIEGDPNKIIHSLLGDVLL
AAKYEAENIKKLYEENNNRKDQEGICRAMKYSFADIGDIIRGKDMWIENNDAKRLQTN
LKEIFTKIKEKTGGTTYNEDNDPYLKLRADWWEANRAKVWKAMKCKTNGVDITCDSDH
TPLDDYIPQRLRWMTEWAEWYCKAQSQEYKKLEEKCSQCKSKGKGGNECYRETKECND
CKQACEEYKRKIKTWADQWKVISNKYEDLYKKAQNPTNAVLKDNKDEKDKNVIDFLTQ
LQKANNGEKTGVHTVYSTAAGYIHQEARTRECQEQREFCDKKNGIDNTSYAFKDPPHG
YATACDCINRSQTEEPKKKEENVESACKIVEEVLSKPRDKTTGGIDHCNPKYYPRKEN
YPGWNCTPGQFKSGHAGACMPPRRIKLCVINLQYLNEKKSPEELRKAFIQCAAIETYW
LWQKYKKDKNGGVAQAKLNSGTIPDDFKRQMFYTFGDYRDLCLDTDISSKADTSTGVG
KVKINIDSVFQKIDITNVEQRKPWWGKNAEAIWDGMLCALSYNTTNKNMDYNAHTKLN
PTYGYNAIKSELEDFVNRPQFLRWFTEWSDEFCTERSIKIKELETKCNDCTVSESGTS
DATGNKTCDDKDKCDECKRACTTYKTWLKNWKTQYKTQSKKYFDDKRKELYKSIDDVA
SSTQAYQYLHAQLKKLCGNADCKCMDGESKETTGQPDNSHDSHMPASLDDEPEEVNGK
CNCKVKHRPQPPLALPPPAPSGPPAEDQIEHDNRGRSERGDQGPLPARPPPPPQAAQP
PQPKPKRTGEGLGRNLPPADRNTNLSDSEEEDDEDDDEVQEEEETPPSEAEEGEGHVE
TEEETKPVKEKTEGAGATEVTKQGSAPTATTPTVEDICATVAKALKGDKSLNAACALK
YGKNNSRLGWKCIPTSGDKTDTSENGAPRRARSAHGGKSDSEKGSICVPPRRRRLYIK
KIVDWAESQSKTVTSVNGDGNGSQEVVSVNGASESGGSGSGTESQASDVSQGNGASTS
PQVALLHAFVKSAAIETFFAWHKYKVDKEIEEKEKQAAQNHLVQRKTSENPQKKLEGG
EIPEDFKRQMFYTLGDYRDILVGDKTMIEALEKSGDTKIEDISEKIPKILDGENNKAA
GGGPKQPNSGKTPQEWWKENAKHIWHGMICALTYNTDSNGKDKKIQQVKATDNTDLFQ
KLKKDNDYETVSFGASGTGAKSNDDTKLKNFVVRPTYFRWLEEWGEEFCRKQKHKLYI
IKKDCRDNKFCSGDGLRCDEKVPDKKDIFKHFDCPSCARHCRSYRKWIERKKTEYEKQ
ESAYSKQKSNYVNGSNGDGGNNNDKEFYTKLETCTKATNFLESLKGQCIGNNNGGTDI
KFSNTNITFGSAEDCKPCSEFKVNCENGSCGSAKQKDCPNNTITSQNIKGLTDQVDMR
VSDNTESGFEGDLGICQGAGIFKGIRKDEWKCGDFCGIDICTLEKTNNGKESDKKYII
MKEFVKRWLEYFFEDYNRIQKKLKTCKENGKGSTCIRSCVDEWIKLKKDEWQKINSNY
LDQNTKENPEGNNLSSFLEDGPFKNEVDKAIKPCGNLTDFKKSKKCNGTSRSGNSEES
TKYDGVICLLDNLKNIIKTCQNVPSGKPDTPCQKSPAPVGDDDDPLEEENPVTQPNIC
PQTSVEEKKKEEEEKCDEKEEEEEKEEEKDKGDEEVKEEEKDKGDEEEEAEEEEEEEE
ETDSHIYEDYSDSDAEEDDEDEAVTESLSPSESQPKRLLREFPSPELKNAMLFSTILW
MVGIGFAAFTYFFLKKKPKSPVDLLRVLDIHKGDYGTPTPKSKNRYIPYVSDTYKGKT
YLYVEGDTDEEKYMFLSDTTDITSSESEYEELDINDIYVPGSPKYKTLIEVVLEPSKS
DGNTPGKGDGNTLGDDMVPTTNTFTDEEWNELKQDFVSQYIQSRLPMDVPQYDVSTES
PMNIGGNVLDDGMDEKPFITSIHDRDLNSGEEISYNIHMSTNTNNDIPKYVSNNVYSG
IDLINDTLSDNKHIDIYDEVLKRKENELFGTNYKKNTSNNSVAKNTNSDPIMNQLDLL

HKWLDRHRDICENWGKKEDILNKLNEQWNKDNDGGDIPNDNKKLNTDVSIQIDMDETK
GKKEFSNMDTILDDMEDDIYYDVNDENPSVDDIPMDHNKVDVPKKVHVEMKILNNTSN
GSLEQQFPISDVWNI
>PFD1245c, 2149 bases, 7CEBB869 checksum.
MAQKVGGGGKDDYKDAKDLLDKIGQQVHDKVKNGDAETYKGALKGNLSFATASGETGG
TNKPCDFGYDKLISGRGGVTARGDPCGKDGTGKEHRFSKERGAECDKNKISGSNDNEG
ACAPYRRLSLCNKNFPNMNSKDSLKAKNDLLADVCLAAKHEGNSINTHYQKYQAQYAS
SASSSQICTMLARSFADIGDIVRGKDLYSGNKKKKQTEREKLENNLKTIFGKIHSDVT
KSGNNKDALQERYQKDEKDGNFFQLREDWWYANRATIWEALTCNAQGNTYFRQTACAG
RSATRNQCRCDGANIVPTYFDYVPQYLRWFEEWAEDFCRKRKYKLENAKEQCREKDKY
YQERYCSGNGYDCTQTVRAQEEYSMENNCHKCFFACNPFVKWLGNQKLEFDKQKKKYT
QEIEKYKNGTKQATNGTTNNLYVKDFYDELKNKHENVRSFLELLSKETACQKHPKVEG
EGYFDFTKFELNEIFSLTEYCKPCPYCGGKFVDGVFRSEGDEEGKCLRLFSSYVPPEG
VDPTEIKVLISGEGHDNINKKLDAFCTNSNDNSLYEKWNCYHDKEGKDKCVWEYDEKD
KNKKKVKKFYDFFRFWVTHMLDDSMEWKEKLKGCLKNGTKIKCKNGCKTPCKCFEKWI
EQKKKEEWTKIRNHFYTQEGFGQEVGQGIPHYIILEQFLENEYFDGISDAYADPEQME
KIKEKLDEKKKERNDDPTNKETIIDFLLEHEGEDAKKCTNSHNDQECNQQKKQQQQET
PARSGTSPSSPRDPNHVDSHEEPDDNDDDSSDEDGEDDVVEEAEEQTHKTTEGTGEGP
KETTTQDTEVTDGPATDPSVEVCSIVDKLFSGNDFGDACGTKYDKYGKEKFPNWKCIS
DSTTKSGAPVKSGGSVCVPPRRRKLYIHDLQSLGVEVDKAPSQEDLLKWFVKSAAVET
FFLWDRYKKENTKRQSGSPLPLLEGAGGGEETPETSLKNGTIPLPFLRQMFYTLADYK
DILFSGDKDDNTKSSTYHDILKGDKEIKGREEKIQEQLKKFFQNSGNQSPPSGTLPPN
SVKDPSSWWKENAQHIWNGMICALTYKEIEVKNPDGKNTYKIEQIQDADKLLGKIKEE
KGEYHYSKVTLKDENSGTQAKSNDDTKLENFVERPPFFRWLHEWGSDFCGKRARMLKN
VKDNCRSGIHGERYSSGDGENCEKMLRDDYDTVPDLEYRDCGKYCRFYKKWIEKKKTE
YENQQKAYTGQKKKYEEESKGGVNGVCETLQENAAQFLERLKNGPCKNDNVDDSGKDK
TGNSHIKFDDHKTFGHENYCDPCPIFGIESKNSGWSEVTQKTCKDNAVITKDNIKTKI
KADQQVVLHVSDNSDHKFERGLDECKEADIFKGIRKEEWECDKLCNSVVCFLKKKNEN
GTDLKQYIEIRALLKRWVDNFFDDYNKIKHKISHCINSGNKSTCTNDCPNKCKCVRKW
IEKKKNEWEEIKKRFNDQYKKENNEGTSSNLNSFLETLIPQTDVDNVIGKVKTLSDLY
DSNECIDTDTSKKGQHEYNDVVECLLYKLQKKIDTYNTQTIEVTDPNSCVLKPTEDET
YDESPEDDTSTTSVVPEFCEQFVKPEAPPPKVPEIPKEEDKDKRDEEKPASPTDDVDT
DSAGTEKLPTPPAPAPVPRSRPKQRKKQKRQITPKEYRLTDVLLPSAFPLSVGIAFVA
LSYFVLKKKTKASVGNLFQILQIPKSDYGTPTPKSKNRYIPYVSDRYKGKTYIYMEGD
SSGDEKYAFMSDTTDVTSSESEYEELDINDIYAPRAPKYKTLIEVVLEPSGNNTTASG
KNTTASGKTQSGNNTTASGKNTPSDTQNDIQNDGIPSSDIPHTNKFTDDEWNTLKHDF
ISNMLQNEPNTEPNILRDNVDNNTNPKTLHVSMDEKPFIMSIHDRNLYSGEEYSYNVN
MSTNSMDDRQYVSNNVYSGIDLINDSLNSNNVDIYDELLKRKENELFGTNHVKQTSIH
SVAKPISDDPLLNQLELFHKWLDRHRDMCEKWENHHERLAKLKEKWENETHSGNTHPS
DSNKTLNTDVSIQIDMNNPKTTNEFTYVDSNPNQVDDTYVDSNPDNSSMDTILEDLDK
PFNEPYYYDMYDDDIYYDVNDHDASTVDSNNMDVPSKVQIEMDINTKLVKEKYPIGDV
WDI
>PFE0005w, 2207 bases, 2E213C1D checksum.
MGPPGITGTQGETAKHMFDRIGKQVYETVKNEAENYISELEGKLSQATLLGERVSSLK
TCQLVEDYRSKANGDVKRYPCANRSPVRFSDESRSQCTYNRIKDNETDDNACGACAPY
RRLHLCDYNLEKMGKTSTTKHDLLAEVCMAAKYEGDSIKTHYTIHKHTNNDSAAELCT
ELARSFADIGDIIRGKDLYLGDIKKKQNGKKTEREKLEENLKRIFGKIHEDVTNGKKE
VLKTRYKDINDPEFFKLREDWWTANRATVWKAITCHAGESDKYFRNTCNDSEHSGTFS
QPNKYCRCNGDKPGEDKANVDPPTYFDYVPQYLRWFEEWAEDFCRIRKHKLKNAKEQC
REKYKSGTDRYCSRNGYDCTQTIRGRNILVSDSECTNCSVVCTPFVKWIENKKLEFEK
QKGKYTKEIEKANGTSNGTTIRTQYGTINNMYRKDFYQQLQSGYGDVNAFLELLNKET

TCKDHPKVEEKSDIDFNEGTEKTFSHTEYCETCPWCATKKKGIDGNWEEQKYEEGCEN
YLMKPIDESKSTDIDLLVKDTSGTTMVEKLGGLCNDSSKRTVQMENWQCHYEKKSQYE
DGFDKDYCVLKDDKKKKPEHRTIKSYYTLFPNWINEMLKDSIDWSKELKTCINNEKPT
NCIRECKSKCDCFKKWVVQKEQEWKQLEEHYEKENFSGDFGPRISPYVTLEGNLQYSY
LEMIRKYYAQEKPVQEIEQIIEKNKNNFEVKEDDNSITKFLQQEKGIATKCIEKQEEC
KQQKKQQRQKQPADKVVSRSGASPDTASPDTKATEEKEEEEEEEEEEDLGEESEEPED
QAVVDQDGQGETTEKKVPATTEEGSPKETTTPEKSVDVCKTVAELFSNVDNLKEACTQ
KYGGNNSRLGWKCIPTSGGEKATGGSGESTGSDATTGGSICVPPRRRRLYVTPLTTWA
SGGTTQVETQASGGNTETSQVSGETTPQGQTPSESEAQTASQDPSEKLRTAFIESAAV
ETFFLWDRYKKENTKRQSVLPLLEPINGDTISDDNNPEKLLKVGKIPPDFLRLMFYTL
GDYRDILFSSSNTSDTTGKETPSSSNDNLKNIVIEAGGTDEKDKQKMKEIQQKIDKIL
KQSGTTPPTPVTHSPSSGTTPSSWWENNAKHIWKGMVCALTYKDNTNGGPPTQDNDLK
KALWDDKTNEPKKSEYKYDQVELKEENSGTGVTKGHAEAPGDNTPLSKFVLRPPYFRY
LEEWGETFCRERTRRLGKIKGECKVDDSGSGRGGVIKRQYSGDGEDCDKVHEDPTTLP
DLGSSCPKSCSSYRKWIKIKKDEYEKQQKAYEQQKKNCEKESEGVENKSDICDQKFLQ
NLKNYGSIDSFLDRLKKGPCSKTYNESGEDNNKIFEDTEKTFGHQNYCGPCSEFKINC
EKGNCDKTKGQECNGKKTIDAQNFEQMGQTAKEFVMRVSDNDTTKFEGHGLEDCNGAG
IFKGIKENKWKCDKVCGYVVCKPKNGNGKNEGTYIIQIRALVRRWVEYFLEDYNKIRK
KLKPCINDGKEPKCIKTCDKKCNCAGKWIKLKQDEWENIKNRFNEQYKNGNEGDYPVK
TILEELISRIAAATDKAERGSLDKLKTSLGCNCSDNSQKKNTDERDIVECLLEKLQVK
ATSCQSQHSDKTEQQCQEYTPEPDEEDLLLEEENENQVENQKVGNKAPAFCPPPEKPP
KEEPDEKCGDKEEEKEQEEEKDKGNEEQSGAPSSPSPSEGTKELPPPPEPPEEAKPPE
PVKPTPAPAPAAPPAAPAQPTLPADEPFNRDILATTIPFGIAFALGSIAFLFLKKKTK
STIDLLRVINIPKSDYDIPTKLSPNRYIPYTSGKYRGKRYIYLEGDSGTDSGYTDHYS
DITSSSESEYEEMDINDIYVPGSPKYKTLIEVVLEPSGNNTTASGNNTTASDTQNDIQ
NDGIPSNKFSDNEWNTLKDDFISQYLQSEQPNDLPNDYKSGNVTLNTEPNILYFDKPE
EKPFITSIHDRNLYTGEEYSYNINMSTNSMDDIPINRDNNVYSGIDLINDALNGDYDI
YDEMLKRKENELFGTNHVKQTSIHSVAKNTNSDPIHNQLELFHKWLDRHRDMCEQWEN
HHERLAKLKEEWENETHSGNTHPSDSNKTLNTDVSIQIDMDDGKPKKEFTNMDTILED
LDKYNEPYYDVQDDIYYDVNDHDTSTVNPNNMEKPSKVKIELDVNKKTIKEKYPISDV
WDI
>PFE1640w, 3164 bases, D990C2B0 checksum.
MGNEQSSSSSEGAKNPSIIESQNSPRNVLERYGEGIMIKEKDEARIYENSLKGQLKQA
KFRGGPSTSVDEQYYYYPYPCSLNHNEHTNIRHDNVDERHPCHGREQNRFGEGQISEC
SNSKIKGNENKSDGGACAPPRRRHMCDKNLEALTVENTQNCDDLLGNILVAAKYEGQS
IVNNYPDKNNSNNKSSICTALARSFADIGDIVRGKDLFLGAPNKEKIKLEENLKKIFD
NIKNENAELSKLSLEKVREYWWAIHRKELWEALTCNAPKGANYFVYKLDGPKFSSDRC
GHNYNGDPLTNLDYVPQYLRWFDEWGEEFCRKRKIKLENVEKACRKDSEKLYCRHNGY
DCTKTIKNENILFDDPKCTDCLIKCSLYEIWLDNQQKEFEKQKEKYEKEIQTYISNNE
ISNNNISNEYNKQISEKYTDKKYETLDKFLNLLKEGKYCKERMTGESSIDFNNDVDKT
FSYSKHCKVCPHCEVDCENGNCEVKNKPDGNCGKNVKYKPPYGVKPTEITVLYSGNEK
GDISKKLSEFCSNKNNINVKNNETWKCYYKNSDNNKCKMESNSENNKGAEKITSFHEF
FELWVKNLLKDTMKWENEIKDCINNTNITDCNDECNKNCVCFDKWVKQKEEEWKNVKK
VFENKKYIQDKYYLDINKLFESFLFKVISELDQGEAKWNQLKEELKKKIESSKANEGI
KDSESAIELLLDHLKESATTCKDNNANEACSSSQKSSPNPCADKSGGKLVSVKQIAQY
IKRQAYEQANYRSDGLHKLKGKAHEGKYKRKGRENYFKDKLCKIGKYHSNRDPFESKG
PCYGKDGNKLSFQIGTDWKGAEEEKMIYNDVYLPPRREHMCTSNLEHLVTDNKPLDGT
DGNPKLVNNSFLGDVLLSAKYEAQRTKEDYEPVSDEQSICRAVRYSFADLADIIRGRD
MWDKDDGAQKMERILKSIFKNIYETIGDKKGKYTNTDGKYLELREDWWEANRAKVWEA
MKCAIKGLNVTSSDGKLSDHCGYSDHTPLDDYIPQKLRWMAEWAEWYCKVQSQEYDKL
KKSCVMCMQKDNGKNCWKHNSECGVCKGACTAYQGKLLPWEYQWNVISHKYETLYEKA

RIAADNGGLDTSSGDIDPKDKTLVEFLFNLYVQNGGKIGTIGTTGDKDTRDTTPTVYS
TAAGYIHEEAHIGDCKEQKKFCEKKNGAKPPSGDLEVDNEYAFRDKPTDHDKVCDCES
GEKLQNKKSEGIVLVEPCNVVNTLLKDKNENSDIDQCIRKYKDGKEKYPGWDCTRNKI
KIGEEGAYMPPRRQKLCVDFLKQLKDQTDEKLRDAFIKSAAAETFLSWHKYKEDTQKE
KATHTADNDLNKGIIPEDFIRQMYYTFGDYRDLCMNKNIGNDVSNVESKIEGVFSKAD
GTSSSNLSREVWWKKHGPKIWEGMLCGLSHAGGNDAIKSNQDYQYSEVKIFKQSDGSG
ITLSQFAERPQFLRWITEWYDDYCHTRQKYLKDVKEKCKSNDQLKCDKECNNKCDEYK
KYMEGKKKEWDAQYKYYKEQRNKKEVVNDSKGIIVKDYVLANAKEYLKKKFTAICVTS
SGKAQNSATEEVKKNIELLSEGQYYDAKEHCGCTKFIHDGKYSKISGRSNCYGLNSDA
KKNKIKWRNGDEKDYAFLKKRNLSGEVFFPSRRLGICFRGLDGYYRYPEVKDKDTLRK
TLMEVAATEGYNLGQYYKEKKKANKEAYRYSYEVRPYSAMKYSFYDLRDIILGYDNLE
DNSTTTEKNLKEIFKNESKEDSQGRQTFWNNNKDCVWEAMKCGYNKSGETIPDECKNM
PRDSDYPIGSNRDEGTEYQFLRWFAEWGEDFCKHKEKELKKLVGACNDYNCGDNEDKK
KKCTSACTQYKQFISEWKPQYENQIKKYGKHKHKIYSEHPVAKEAKDAQEYLDKQLQK
SCNSGGKCDCMNKKSMSNRNNMPASLDDTPSEYKKKCNCPLPHKKPEAPLARAAAPPA
STKKKTPSRKTQPSQPAREMQADEPASPSRRASLKEKAVASKKEEKTARPTKPPKKVE
QPPTGIRAPTRTPRAAPQARTRRSTPTTTASDVATMVKAILSNEPDSRGGIEGCNPKT
YGQYPKWSCIVGKSKENENGTCMPPRRKKLCINNIQYLNYETVNKREKDIKEAFIKCA
AIETHFAWHRYKEDNKKAEDELKKGTIPDEFKRIMYYTYGDYKDMFFGTDIANDKKII
TITNSVTTILNENNKKKQDKKKDEELRKIFWEENKKFIWEGMIYGLTYHLKDENEKKK
IKDNYQYNDMTKLTPSLEEFVKRPQFLRWFTEWAEEFCNERKEQLKKLEAGCKEYECN
GSDDGKTQECAEACVTYQAFIKKWKTQYEVQREKFKKDKDNKKYKDYPSTERDIEKAT
YAHEYLNMKLKEFCGNKDCSCMQKPSSQLPKTKSPLQPSNGNDMPESLDYHPEEFNKC
ECPEISKNESKTHTKKIPAPKIPMNCVEKAAYYLSKEAENNIERTLKQKITHSNCVKE
TDNSFSSNNRCDPNKPYAPDKYIGRRNPCGNREQNRFKLDSEWKCYKNIKLYQEKKRV
CIPPRRENMCISNLDEIEIAKVNQSNYLLNMVRIAARNEGIDIIKNFNSQNGCAMNPI
CDTMKYSFADLGDIVRGTDMLRIGGYLPPVEIKLYKAFEYIYGKWRNKNKGRNKYNDV
QTFRSAWWDANRKDIWKAMTCKAPEDAKLFRKGRMDGFESITLIQDKCGHKDDPPVDD
YIPQRFRWMTEWSEYYCKALKVELEKLEKLEKSCDHCKTTNKCKNDYDKNKCEKCKTR
CQQYDNFILKWKTLFDIQSKKYKELYEPIDTKNSTYDHVENFVQKLKKYKNECSVESV
SEYLHETSKCLNYKFDENDGSSNIRSYAFEETPKSYKEACSCTLPSKNPLDNCPTDQN
KDVCKELQTFTFCSKNDYDNNLDNWNAYLVLNSSDDNKGVLIPPRRRHLCTRPITAYN
YRKGDKEILKKKLLTSAFSQGQLLGQKYKSEEELCFEAMKYSYADYSDIIKGTDMMDT
SLSEKIKKIFETSNQDTEDCKTWWEKNRSHVWHAMLCGYISKNKNENINPKWCNVPTE
DGTDQFLRWLIEWAMQACKEKKRVRDSLKTKCRCSNKDNFKASELLRQPGCQNDIRKY
ISLNILIQNSMENLNIKYKKFKDQSSGLGFRV
>PFF0020c, 1325 bases, 2DAE4B42 checksum.
MGSDYSSPGGNKSVNITESEKSARNVLEKIGRHIKDEINKNSNHTNKLKGTLSNAQFH
DGLHKAAGWGVRYGPANSCDLEHRFYTNINNGYLPARNPCHNRNQNRFDENAEAYCNS
DKIRGNENNSNAGACAPFRRQNLCDKNLEYLINENTKTTHDLLGNVLVTAKYEGDYIV
NNHPNRGSSEVCIALARSFADIGDIVRGKDMFKSNDNVENGLREVFKKIYEGFLDKGA
REHYKEVKNGNYIKLREDWWTANRDQVWKAMTCVAPENAYFRKTEADGIGISSLILPY
SKCGRDTDPPVVDYIPQRLRWMSEWSEYFCNVLNKEIDEMNNQCKDCEMSRRCNNDTE
GEKCKKCKEQCQIFKELVSKWKNEFDKQSMKYKELYIKASTNITKQNSSSPERGYRRN
HRRRGYDDDTNVQLFLKKVIENNECKVESLGKYLDKTSHCGNYNFNYDNIPGSNRPNA
FEIPPEKFKKACKCKIPNPLEKCPNEENKNVCTRFDKVSSCTSLFFKNDLIEWNNSGV
KNKENDNNGVLVPPRRRNLCINLFSKKDYKMKDENDFKEDLLNAAFSQGKLLGKKYSN
YSNEAYEAMKFSYADYSDIVKGTDMMNDLKKLNKELNTLLKETEKGDISVDRKTWWDD
NKNVVWNAMLCGYKTENENQQLNSSWCNVPDDDNIDQFLRWLTEWAQQYCKEKLIKAH
IINTKCKDIVEGRKHKSMVDITDVECKRLFIDYEEWFRYRYNQWKGLSEKYIKIKKSK
NSGVNIPSEECAASYVTKHCNGCICNLRDMEDIHKNINNQNELMKEMINIIKFDTDQY

RTQLQNISNSMEINPKSVKTAVDTTKDIVSYGLAGTMGVAAIGLQAGDFLGKKIQDLY
NEFMKPVEKKLDTSSKNLNIYEDPNIMVPAGIGVALTLGLLLFKMRRKAKRQVDMIRI
LQMSQNEYGIPTTKSPNKYVPYGSQRYKGKTYLYVEGDTDEEKYMFMSDTTDITSSES
EYEEMDINDIYVPGSPKYKTLIEVVLEPSKRDTQNDIPSDNTPSYKLTDEEWNQLKDD
FISQYLPNTEPNNNYRSGNSPTNTNNTTTSHDNMGEKPFIMSIHDRNLYTGEEISYNI
NMSTNTNNDIPKYVSNNVYSGIDLINDTLSGNKHIDIYDEVLKRKENELFGTNHVKQT
SIHSVAKNTYSDDAITNKINLFHKWLYRHRDMCEKWENHHERLAKLKEKWENDNDGGN
VPSDNHVLNTDVSIEIDMDNPKPINQFSNMDINVDTPTMDNMEDDIYYDVNDNDDDND
QPSVYDIPMDHNKVDVDVPKKVHIEMKILNNTSNGSLEQQFPISDVWNI
>PF07_0048, 2215 bases, D67739C6 checksum.
MARPGSGGGGSSQDAKHVLDEIGQQVHDQVEKEAKERSNGDLKGNLTISTIFDTETTG
TDDPCSSDYTTRFDARGDPCKKDGTGNDVERFSVKQGAECGNSKIHGNSKGGTGTEVG
ACAPYRRLNLCNKNLENINKYDNTKAKHDLLAEVCHAAKYEGASITLHYPQYQNKYDD
SGSTMCTMLARSFADIGDIIRGKDLYLGKKKKKQNGKETEREKLEDNLRKIFENIKKE
NNSKLKSLTDDQIREYWWTENRETVWKAMTCSEDLKNSSYFHATCIDGKNQSQARNQC
RCPKTSGNVNIVPTYFDYVPQYLRWFEEWAEDFCRKKKKKVQNLQKQCRDKYQGDDRY
CSRNGYDCEQTINKIGKLVIGKGCINCLYACNPYVDWINNQKEQFDKQVKKYGTEISD
GGSGSGAAGGGGGRKKRGTSTTNYDGYESKFYKIFKGKCGTVDAFLGLLNNEKACKEV
KDGGKIHFEKVNSTSGGTAGSNKTFYHSEYCQPCPLCGVKKKDGGNEWEKKKEDEKCN
IKLYKPKDGVVGTTINFLYSGDETNEIEKKLKKFCRTENGTGDSNSDPSLYDPWQCYH
VKQLEKDKEGVDDPVYEANVKNGGGLCILPNPKKNQDSGNNSSNEPEQFQKTFNNFFN
FWVAHMLKDSIYWRTKKLVKCLKNGNTIKCGMNCKDDCGCFKRWVEKKQQEWGEIKEH
FGKQDFGKQGENGVGEMLGVLMKSPSYVLKFVLEKDVLLTSIKSGYGKPEDIERIEEL
LKEEKKKNEEEAADGTDNENKTTIDKLLKHELTDAKDCQQKCDKKPPPPETPARNLPP
PDTQRDPNQEESEEEEEEEDEDAPDDGEVDGDKGDGQETEPEEEEDTEDAKVEPAKDT
TEDTEQDGQGPPVKKDEVNPCDIVSKLFQNPNNFSDACTLKYVTGKNYGWKCVSSGNG
TTSSEGRSPRVARSAPSGDKDGAICVPPRRRRLYVGRLTKWAESKQVTQPQEDGKAPL
APTPPTSSGSQSDPLLTAFVESAAIETFFLWHKYKVDKIREEKEQQELFTNTSTLGKE
LQDKLERGNIPTDFLRQMFYTLGDYRDILFSGSKDAKNGVNDIFSGDKEMKEKEEKIK
GAIQTFFENGASQPPSGKRNDKHEEWWKNHGEHIWNGMICALTYKDNDAKGQTPTQIQ
EVRDNLWDSGKNEPKKPQYQYQTAKLEEKNSGTKPTNQTPSSTSDNTPTTLTDFISRP
PYFRYLEEWGQNFCKERKKRLAQIKHECMDGDTQKYSGDGEYCEEIFSKKYNVLQDLS
SSCAKPCRLYKTWIEKKKTEYEKQKKAYEEQKSNYENEQKDKCQTQSNNNANEFSRTL
GASPTAAAFLNRLGSCKNDNVEDNGEDKLDFNNPEQTFRPATNCGPCPVIGVKCKNGN
CSDTSNGNMCKGGMITAENINNSTDDVSMLVSDNSTTGFNGLDEACQGAGVFEGIKED
KWKCGTVCGVDICTLEKKDTNGQEGDKKYITMKELLKRWLEYFFEDYNRIQKKLKPCT
KSENKSTCIKGCVEKWIDKKKEEWKNINNNYLQQYKYVGNTLTNFLEILIPKIDLTND
KKKIKDLPAFLKLYGCNCADNSQNSTQNDVVLCLLENLKTKAKKCEENHKPSGNQQQP
CQESPSVEDDEEDLTLEETEENTEEAKKKMMPTICETVVPTEPEEPGETCTPAAAGGG
HNPEQTPVLKPEEEAPTPEAETKKDKAPVKPPSQPTTPQIVDKTPALVTSTLAWSVGI
GFAAFTYFFLKKKTKASVGNLFQILQIPKSDYDIPTLKSKNRYIPYRSGTYKGKTYIY
MEGETSGDDDKYAFMSDTTDITSSESEYEELDINDIYVPGSPKYKTLIEVVLEPSGNN
TTASGKNTPSDTQNDIQNDGIPSSKITDNEWNTLKDDFISNMLQNEPNDIPNDYSSGD
IPFNTQPNTLYFDNNQEKPFIMSIHDRNLYTGEEYNYNVNMSTKNVDIPMSDKNDVYS
GIDLINDSLNSNNVDIYDEVLKRKENELFGTNHPKHTNTHSVTKSSNSDPIDNQLNLF
HKWLDRHRDMCEQWNNKEKVLDKLKEEWNKDNNSGDIHTSDSNKTLNTDVSIQIHMDN
PKPKNEFKNMDTTPNKSTMDTMLDDLEKYNEPYYYDFYKNDIYYDVNDDDKTSMDNNN
NLVDKNNPVDSNNSTYNHRNPADINKTFVDINNHNQHPIEKPTKIQIEMNSNNREVVE
QQYPIADIWNI
>PF07_0049, 2275 bases, 3869900C checksum.
MAPTSDGGGTKDESAKHVLDEFGQKVYEEIVGKKADAKTYKDELRGKLSLAPIWNESA

GTDDPCKLESEYTELISGSLASGGAARGHPCGNVSGNDGTGNDDLKRFSKERVSKYDE
KKIRGSNDGACPPYRRLSLCNKNLENIKTNIIDNKHDLLVDVCMAAKYEGESIKAHYP
KYDAEYSSGSGSDFPMCTMLARSFADIGDIVRGKDLYSGNKKKEKLEQNLQKIFKEIY
DKLNGAKDHYQKDGDKFFQLREDWWTANRHTVWKALTCKAEGAYFRPTCSDGNSQSQA
TKQCRCNDKPNAGKPKAGNGDVNIVPTYFDYVPQYIRWFEEWAEDFCRKKKKKVQNLQ
KQCRGTDASKEPRYCSRNGFDCEKTISRIGKVRMGKGCTDCFFACHRYENWIDNQRKQ
FLKQKNIYENEILGNSSRRRGAGGATTTNYEEYEKKFYLKLQSNGYGDVNDFLGLLSK
EKACEQITTQEEGRINFAEKHDDNNNDKEKGTFYRSKYCQPCPDCGVKPLGGGKFQDK
ETKRKKCEGEKLYEPKPNKEGTTITILKSGENHDDIETKLKAFCKTQNGGGVVLSGGR
DNEKKSDKDSLYEEWKCYEGKDVKKVKNGEEEDDEEDVQEVKDAGGLCILKNDKNKKE
EKTVNEPEQFQKTFHDLFYYWVAHMLKDSIYWETQKLDKCLQNGNKKCGKKICNSDCD
CFKRWVDQKKEQEWMKIRNHFYTQEIKGFQGELFKLSHDDLLKQVLKEEFYKEKSEDA
SAQDNQNSLDAEEAKELKQLSKIIESEENQEAGAGANGKKNIMDKLIDYEEKEAQKCL
EKRKQTCPPPEESPDRSQTPPASRSDSPRVDQKEEEEEEEEEDDEDEDEVEDTAEGET
QAETEQVEDQVNGEGEQPVEDHSDQVEEGTTQDNAEKPCEIVDKLFNNPDNFKEVACQ
QKYAKNNSRLGWKCIASDTKSVATVKSDASGSICVPPRRRRLYVGHLQKWAEKYNKVA
PQVGGGNTKAGESSQSSSGSETPSQPDPKVELRDAFVESAAIETFFLWDRYKKIKEKE
KQEEQQRQQENGGRLATLNGDTLSVEQTPENQLKSGIIPNDFLRLMFYTLADYKDILF
SGSKDDNTKSSTYNDIISGDKEIAQREKTIKGAISTYFSNSGTTPTPVTQPSGQKTTP
KDWWKENAKHIWEGMICALTYKESENGDKTIEKDEQVYEKIFGKDNNDKPGTTGTNTG
TTGTPTGTYNDRYKYETVKLDDQSETEAISNDNPTLEEFSKRPTYFRWLHEWGESFCR
ERKKRLKDIIYECRNIDKAGHHYCSGDGHDCTDKNLRHKNMSADLFCRDCHKQCRKYR
KWIDIKFEEFQNQKSIYQAEHGKLKANHNGDNNCCKEIHNRSTAAKFLEALKHCKNNE
GDEEKEEDKKNNKIDFGKPLETFRHSKYCETCPFNRVTCNSGRKSGTNGCNVNGNGET
WESVFNGIPENGGKTTTITVEMIDRRGPFIKEYLNNSQKSEKSNDLFNASRLFKGLRV
QNWKCKFNDQKMNVCHLINFNKDIDLNKYTTFKVFLLYWLEDFLYGYYISKKRKIVEK
CTQKGEKACSGDGNSKNDCACVKIWIEQKEKEWDQIKNYYDANFKTDSEHIYSRINSF
FEQQLFDSGIKKDKQKVTELRDLERSLGCECAENSKKIKDADKKDIVECIHKYLEKKI
GECTSQPSGENEAQCENSSPFEDDDEEDLLLVEDDKIIKQPGFCPPPEEKTEIEGTDD
KCEEASSPVVPEQPAKEDGDPAAQPEDDTEKKAPVKPTPTKPQRPRRPRRTLELLDNP
PFKTALMSSTIMWSIGIGFAAFTYFFLKKKTKSSVGNLFQILQIPKGDYDIPTLKSSN
RYIPYASDRYKGKTYIYMEGDSSGDEKYAFMSDTTDVTSSESEYEELDINDIYVPGSP
KYKTLIEVVLEPSKRDTQNDIHNDIPSDIPNTPSDTPPPITDDEWNQLKKDFISNMLQ
NTQNTEPNILHDNVDNNTHPTMSRHKVDQKPFIMSIHDRNLYIGEEYSYDMSTNSGQN
NVYSGIDPTSANHDSYSDKNDPISDNHHPYSGIDLINDVLNGDYDIYDEILKRKENEL
FGTYHTKKNTSTNSVAKNTNSDPILNQINLFHKWLDRHRYMCAKLKNKEDILNKLKEE
WNKENNNNSGKTYNSDNKPSHNHVLNTDVSIQIDMDNPKTKNEFKNMDTTPDKSTMNT
MLDDLEKYNEPYYYDFYKDDIYYDVNDDDKASVDHNKMDNNNSDVPTKVQIEMNVINN
QELLQNEYPISHM
>MAL7P1.50, 2267 bases, E423C7B8 checksum.
MASQSGGGSPQDAKHVLDEFGQQVHKEVKKEAERRSKGELKGLLTSAKLSGGEIAGTT
DPCSSDYTKHFEANSNRYPCGNTNVDRFPDNDGAECDNSKIKGNKGKEDNSEGACAPY
RRLSLCNKNFQNNNNDHSSNAKHDLLLDVCMAANYEAQSLITYHDKHELTNVGSQICT
VLARSFADIGDIVRGKDLYLGKKKKKKTETERERDQLEQKLKGIFKKIYEGLTGGVKD
HYQDTDNYYELREDWWALNRDQVWKAITCKADNSNRYFRATCDSADGKGSSVARNKCR
CKDEEGKNETNEVPTYFDYVPQYLRWFEEWAEDFCRKKKKYVDIVKTYCRGKYQGEER
YCSRNGFDCEKTVNARGKVRMGKGCTDCFFACNPYVDWIDNQRKQFLKQRNKYADEIK
IYTEGASGGSRQRRNVRSNYDNGYEKKFYKELKNRYGSVETFLVLLNNEKACTAVDDD
KGGKIDFKEVNTGGAASGGTSGTNVESQGTFYRSKYCQPCPLCGVERKGGSGWEEKDK
IEKCKSINLYKPRDDKVGTTIKILKSGEEEKDIETKLKAFCDNKDSGNSDPSLYDPWQ
CYQIGELTKDQKEGGEDDLDYEADVQTGGGLCILKKEKKTDNDPDEIQKTFNNFFYYW

VAHMLKDSIYWRMKKLERCLQNGNKKCGNQKCEKPCDCFQRWVQQKETEWGKIKTHFN
TQNIGALTGCNPIVTLEGVLKLQFLKDESTEDSEENSKNSLDSEELKHLKHLSEMLQG
TGVDGLTCSDSGTEQKTLMDKLIDYEEGIATECKKCDEPKPEGAAGGRSDGPPGSPPR
PAEKDEDHHSDESDEEEDEEHTEQGEEKTEGKTEEQPEEVKEHTETPEDEVNPCKIVE
TLFTSDDNNALNEACKQKYQYGKEKFPNWKCIPSGDNSTTSSEGGDRGGQSRAKRAAP
RESTTSSGDTTGGSICVPPRRRKLYIKKIVDWAESQSKTLTSVNGDGNGSQEVVSVNG
ASESGSQGSRSDSSSSSSSDSSQGTTSATSQSPNGDLLTAFVESAAIETFFLWDRYKK
IKDIEEKEIKEKNEIYGIYTSSVDEKPQNKLNGGDIPNDFLRLMFYTLGDYRDICIGV
KEDVIKALEASSDKNIETIKKAIDEILSKQSRNNQQSGQKSENPRDTWWKTNGEHIWN
AMVCALTYNEDTSGAKGKQPQQDQSLKDKLIDKKTGKPEGKYHYEKVTLENSDTQAIS
NDTINNPKLKDFVEIPPFFRWLHEWGSDFCGKRARMLKNVKHNCRNIDNPGHQYCSGD
GYDCEKIKPENYKNISDLDCRDCYKQCRKYRKWIEKKVEEFHKQEKKYKGEHGKLRND
NCSGNDNKEYCEQIKQKTSAADFLAALKHCKNDQIDGEQGNQEDKKNNEIKFEEPLKT
FGPLEYCKTCPFNGVNCTVRRGTNGCIENGQIWKKVFEGISGNGENSTITVDMIDRRG
PFIEKYLGDSNDLFKTSKLFKGIRKQNWTCKFNKDKNMDVCHLTNFKDNIDLNEYTTF
KVLLIYWLEDFLYGYYLLKKKKKIYLCTQNKEQTCSEESKNDCACVKEWIGKKRAEWK
TIRDRFNDQYKSENSGDTFPVRSFLEGLIPQIPVADVQGNVIKLSKFGNSCGCSFSAH
STNGKDDAIDCMIKKLEEKVTSCLSSTSGDNLAQCENSTPLEDDDEPFEEEENPENMR
PGFCPPPEEKTEIEGTDKKCEEAPTTPPEPAPPAKSGEGTEELPSAPEPPQDKALPKP
AAQPKDKKRPKRQPQNPWEHPIVIPSLATSTLMWTVGIGFATFTYFFLKKKTKSSVGN
LFQILQIPKGDYDIPTLKSSNRYIPYASERYKGKTYIYMEGDSSGDEKYAFMSDTTDV
TSSESEYEEMDINDIYAPRAPKYKTLIEVILEPSKRDTQNDIPSGDINSDIPNTPSDT
PSPITDEEWNTLKDDFISNMLQNEQEDIPQPDVSKELPLNTHPTPSHDTLDQKPFIMS
IHDRNLLNGEEYSYDIINNIGNNDLYSGFDPKSGDNVSYSGTIGSISDKTSPYSGIDL
INDSLNSGNHIDIYDEILKRKENELFGTNHVKHISTHSVAKLTNSDPIECQLNLFHKW
LDRHRDMCEKFSNNKEELLDKLKEQWENKTHSGNKHSDIPSGKLSDTPSDNNIHSDIH
PSDIPSGKQSDIPSDNNIHSDIPYVLNSDVSIQIDMDNPNQVDDNTYLDTYPDKSTMD
TIMDDLEKYNEPYYDIYYDVNDHDASNVDSNNMDVPSKVKIEMSVKNTQMMEGKYPIG
DVWDI
>PF07_0050, 2064 bases, 95B088E0 checksum.
MAAAGSGGQGARDVLEQFGEIVQKKVHNEVDAYREELKGKLWEAKFVGVSHVPSDPCQ
LNHEYHTNVTTGKDDPCDRRWPVRFSDESRSQCTKNRIKDSTSDTVGACAPYRRLHVC
DQNLEQIRPEQITSTDNLLADVCLAAKHEGESIIKNYPQDRNNNEVICTALARSFADI
GDIIRGKDLYRGDKGEKKKLEENLKNIFANIYEELKNGKTNGKKGEIETRYKKDDEDG
NFFQLREDWWNANRNDIWKALTCHAPPDAQYTKKGPHNHITESNKGQCRCFSGDPPTN
MDYVPQYLRWFEEWAEEFCRKRKKKLQNAQKFCRDESSKLYCSLNGYDCKATIRANDE
YTISQACAKCFFSCHRFVKWIDNQKLELEKQKKKYDEEINVTTETSKKTEHGRINIIY
AKKFYDILKEQYGKVDNFLELLSNQKECKNHPEVKGKNSIDFKNDNDTFSRTEICEPC
PWCGVKKQEDGNWKRLERGHPHCPKKPPYTPPDGAKCTEIDVLYTGERNNDILDKLKE
FCRSEGKNERKTDFKNEQWKCYYKDSYNNKCVLQPDRDLGIEKKVKNYVDFMMFWINH
MLKDSIDWRKYIKRCINNSASNECKKNMCFKNCQCFEKWINKKQDEWNKIKKHYNNEK
GFGEFGSYNTLEYILEEDFFKGISEAYGSVDAIERIKNITKNHVSTEEITNAENEEFA
IDILLKHELEEAQECKNNNPEDQKCNEPHDEEEYEDEHEEEVYVNNPCAKPSGNYPAL
AHNVAYQMHEVAKTQLRTRGGRNALRANAAEGKYKNNNKEFTFNGNICSINENHSNCN
HNYSSGPCGGKDGRNEMFEVKDGWKSGADVSKEHAEDVFLPPRRQHFCTSNLENLNTN
SKGLSDGTLASHSLLGDVLLSANKEAGFIKDKYKNQPTLGGFKDEATICRAMKYSFAD
IGDIIKGTDLWDGNKEETDTQRNLVTIFGKIKDKIRDEATKKKYSDAQKHLQLRKDWW
EANRDQVWKAMQCGNDNPCSGVSGVPLDDYIPQRLRWMTEWAEWFCKMQSQEYNKLME
ACTGCMKKGKDGEGCTQKTQECALCKAACDAYKKEIEKWQRQWNNMQVPYITLYEQAR
TVRDGTVIGDVSDQQVIAFFKELHQQNGGKKSVDTVYSTAAGYVHQEAIMNCEKQTQF
CKNKNGENSISGTQNNQKYAFMQPPKGYEKACSCEDYYKAADPQDDACTIVKQLLGNK

GAADDIQGCKKKYDGINPYPSWKCDKSLVDEDGVCMPPRRQNLCVHYLTQLDDKDDEN
KLREAFIKSAAAETFVLWQNYNSKNDNDAQLLDSGIIPPEFLRSMFYTFGDYRDICLD
TDISAKTPDGDLEQAKENIGKVFQKIGPKTANHKPPRGITREDWWKEYGSQIWKGMLC
ALSYDNETRIMNERVRKQLMDSIYNYHILKNHLENFAERHPFFRWFIEWTDEFCQEQK
KEYNDLVQKCTGCNEQTCDSECETCKSQCGQYEKFITQWKSQWEKQSGKYDKLYAEAK
VTSTPTTDPIETKLLKHLKELKDHSTNSDKYSTAGRYMKEEGYINDCELSKQNKFEKN
CNDGSNDNYAFRTYPNDHQTKCDCKKKEAPPPEPPANVVPKVIPKSRTPREAGSSHLQ
NALLSSTIMWSIGIGFAAFTYFFLKKKTKSSVGNLFQILQIPKSDYDIPTKLSPNRYI
PYTSGKYRGKRYIYLEGDSGTDSGYTDHYSDITSSSESEYEELDINDIYVPGSPKYKT
LIEVVLEPSGNNTTASGKNTPSDTQNDIQNDGIPSSKITDNEWNTLKDDFISQYLQSE
QPKDVPNDYSSGDIPFNTQPNTLYFDKPEEKPFIMSIHDRDLYSGEEYNYNVNMSTNS
MDDIPINSHNNVYSGIDLINDTLSGNKHIDIYDEVLKRKENELFGTNHVKQTSIHSVA
KLTNSDPIHNQLELFHKWLDRHRNMCEKWENHHERLAKLKEEWENETHSGNTHPSDSN
KTLNTDVSIQIHMDDPKPINQFTNMDTILEDLEKYNEPYYDVQDDIYYDVNDHDTSTA
DSNAMDVPSKVQIEMDVNTKLVKEKYPIADVWDI
>PFC1080c, 231 bases, FAD00D22 checksum.
MFHYIYKIYIFTIILCASNLFNNNVVEIGTYKLSYHNGGIQFRILAQKNTNKKSNGNT
LTNILLKDKGGKGSKKKNPDDQISSLVSLVDNMNITQEKKDKIKNLSLKYINSRDVKE
KNESINELQKYSNNEECKEYMDSYLMHLRMQNDIKCLKRKNLWNNIWINSITLTLIII
MIACVFSVEITSSSALYPAFILLIFIIYIYARYFPEMKIGYKGIKEACKYYFNKKSK
>PF07_0051, 2215 bases, 7C409B06 checksum.
MAPGGRQGDGGEDIDHQSAKHLLDSIGKIVHDQMKKEAEQRSKGELEGKLSFATVSGE
TGATDKPCNFDYDKLIGANDGKRHPCKKDGKGEEVPRFSDKQGAECANNRIQGNNKNS
HYKDFGACAPFRRLNLCNKNFPNMNSNDSSKAKHDLLVDVCLAAKYEGDSLKHYSEKL
NLTYTDSPSQLCTELARSFADIGDIIRGKDLYLGYNRKEKAQKEKLEQNLKKFFQNID
EKLPLKAKNYYTKEKDPNFLKLREDWWTANRETVWEALTCDAHGTYFHATCSDLNGDC
SQAHEKCRCPKTSGGKAIKAGGDVTIVPTYFDYVPQYLRWFEEWAEDFCRKKKKYVDI
VKTYCREQDKSGNQRYCSRNGYDCTKTVRARGKYRMGNQCTKCLFACNPYVEWIENQR
KQFDKQVKKYKTEISDGGGRQKRDAGGTTKYEGYEKKFYDELKGNYSDVNAFLGLLNN
EKACTAVKDNDGGTIHFETVGTTTTIGENGGTSDTSGTNNENEGTFYRSKYCQPCPHC
GVKREGNEWKEKKKNTDQCTSGKRYKPKNNAEATNIRILKSGDGQTEIENKLNEFCDQ
INLDTLNSGVNSAGGGGGGRSGSQELYQEWKCYKGDDVVKVENKDEEEDEEENVQKVK
DAGGLCILKNERSETNSQNHPYDIQKTYNDFFNFWVAHVLKDSIHWRTKKIKGCLSNG
AKIRCNKKNKCNNDCGCFERWVKQKKDEWKPIKQHFYTQEDIVKKGPFMTLTHDYVLE
GVLELEFLKGDSEEDFEADSSEDSQSRDEDAQETKRIKDMFEKKKKKNKDEVASNEET
IIDFMLDEELNDAKQCIEKHTCPPQEGLARSESDTQRDTHSDDVEAEEEDEEEEEDDV
GAEGESETVKDTEAPVETKKDVDVCKIVEQALNNMENLNAACTLKYGPKAPTSWKCIP
SGSDSSSSGAICVPPRRRRLYVGKLEQWANKHNTDASEARGSEAPSPSGTDTHTQPDP
QKALLKAFVESAAVETFFLWHRYKEEKKKPQEGGLPLLGTGDSVDGEEEEQPPQNELN
GGKIPNDFLRQMFYTLADYKDILDGKNIVADILNGSSGSDKDMVEREKTIKEKIKIFF
EQNGNKEAAPRGSTQTQPSEKLKSWWSQNGEYIWKGMVCALTYEENGARGTDGKTTLK
RNDDVYEKFFGKPSDSSILPVTGSTPTGTAATPNGTTGTYTTQYQYDSVTIGASGTGA
KSDDTINNPTLKDFVLRPPYFRYLEEWGETFCRQRTRMLEKIKDDCKVGQGNGKNGDK
VCSGYGEECKIEDISKEGLFADLKCPSCAISCSSYRKWIKGKKTQYEKQKDRYKTEID
QAKSDNEFCATLKSLSDAAQFLERLGPCKKDNASGEGKKFFENEGEAFTPATNCKPCP
EFKINCKNGKCKSGDGDTKEKCNEKTTIDVNDIENKTDVNNIVMLVSDDNKNKSETVL
EACIEAGIFKGIRKEEWKCRNECGLDVCGLKKGDNNGKLDDKQIILIRALIKRWLEYF
LEDYNKIRKKLKSCTEKGEGSPCIKDCKKKCDCVKAWINLKTKEWEEIKKPYLEQYKN
GYGENYNVKTILEKFQDQPEFKKAIGPCPNLGQFEDSIHCNGAARSENGKKRDIVECL
LQKLEKKAEKCQNQNETACDTPSTCDKNLTPDVEDDDEEPYEDLLLQETEEKPEEAKK
KMMPKICEGVIPKEEVKDKDGCKPAAAPSAETDKEKPVPKPPSQPTNPPNPFEHPAVI

PALMSSTIMWSVGIGFAAFTYFYLKKKTKASVGNLFQILQIPKGDYDIPTLKSSNRYI
PYASDRYKGKTYIYMEGDSSGDEKYAFMSDTTDVTSSESEYEELDINDMYVPGSPKYK
TLIEVVLEPSKRDTMSTQSDIPLNDKLDSNKLTDEEWNKLKQDFISILQNPQNDLPQN
NISGNIQMDTHPSVSILHDDMEEKPFITSIHDRDLHNGEEVTYNINLDDHKNMNFSTN
HDNIPPKNNQNDLYTGIDLINDSISGNHNVDIYDELLKRKENELFGTNHTKHTTTNSI
TKKTHNDPIVNQINLFHKWLDRHKNMCEQWDKHKKEELLDKLKKEWEQDYNNNSDDIH
TSDNNIVSTVNHVFNTDVSIQIDMDDPNPVNPFTNMYTNSDNSTMDNILNGMEKHREP
YFYDIYEDDITYFDIDDEKTPMGDIYVDHNNVNSNNMDVPNKVHIEMNIVNNKKEIFE
EEYPISDIWNI
>MAL7P1.55, 2239 bases, EC964FD checksum.
MAPPGGSVGAGSGGEEDAKHMFDRIGEEVYKEVKNGGAEKYFYELHGTLSKARFENEP
EGLQTQGNPCQLDHRYHTNATNVRSYPCRAGKEERFSQVHGGECDKNKIRDNEDDRVG
ACAPFRRLHLCVRNLENINIKKKIDNDNLLADVCLAAKYEGDLIKTHYTPYEHKYGDS
PSQLCTMLARSFADIGDIVRGKDLYLGNSREKEKLQTNLKSIFQNIYKNLKKPAQKHY
SDPHANFYKLREDWWYANRQQVWYAITCGAEGYQYFRKTACNGGKSSTPNKCRCTTHD
VPTYFDYVPQYLRWFEEWAEDFCRKRKKQLTDAIKNCREEDENGENRYCDLNGYDCKN
TAKGKNKYKHDQECIKCSSVCIPFGPWIKNQKQEFEKQKNKYENEILGKSRRKRSIGS
DTYDGYIEQFYDILKREYRDVDKFLDLLSKETACKQQPYDEPRTIDISFKNPEGIDIF
SSTKYCQACPWCGIEEQKVNGRWIAKGDEDCNNKEIIKFNNEDTTDISILSTDRGKTK
ILEKLGSFCRNGEKIKKDDWKCHYDDNDKSDNCILQNDNKNTKNQEIESFNSLFWHWI
NEMLKDSILWSKELDRCIKKDDKSKCISGCKKNCECFKNWVAQKQQEWKQIEQHYEKE
DFELSGPYGILEGNLQIYYLPMIQEAHPKDEAVQKMEDIIEKNKPNMLSVKEHDNSIT
KFLQHEEEDANKCTGIHNDAKCNQQKKQKTPGGGDDARSDTSHDGARPQPPPITNHHE
EHDDDEDDDPNKIRSIKFEDEKHIQPKFKEHPDEVKETEEAEETEEGTDGGGEIPQKE
TQPAEATTEDGTGETTQITKEDGVKPCDIVSKLFQNPNDFSDACTLKYVTGKNYGWKC
IPSGDKTATSSGNGERPSRSKRGAEPTSDGVPTTGGKDGATGGSICVPPRRRRLYVTP
LTRLAGGDGNTQAGETTQGNGASTETPEASLRRAFVESAAVETFFLWHKYKEEKKTPA
TQNGALPLPLAPDVSPPSENPQTKLEGGNIPEEFLRQMFYTLGDYRDILVRGGDVNSD
SEKKDGDSNSDRNIVLNAGGDKASMEKIQQEIDEIIKQSGNNKGTSGTTSRGPQIGSH
SPNPDKQQTEREKWWDKHAPSIWNGMICALTYEEKTNSASGSESKIEQNQKLKDKLWD
NDGNKPKNDNYTYDKVELKEENSGPKGQTESSSPSGDNTPLSHFISRPTYFRYLEEWG
QNFCKERKKRLEKIKEECRSGNYGKEHCSGDGHYCKTSDLKHHKMFQDFVCRDCYKQC
RKYKKWIDIKFDEYQNQKHKYQGEYDKLTKVDCPNNGDKKFCEEIKKHSSAAEFLKSL
KHCKDGQNNNDQNNIIDFENTKTTFGPLDYCKTCPLNGVNCNVIRGTNGCIENRQKWK
DVFDKIHKDNEKITENITVEMIDRRGINIDKKFEQLFKESYLFKSVREQNWECKVINN
DTDVCKLTNFDETIDLNDYTTFKVFLEYWLDDFLYGYYLLKKRKIIEKCTQKEGKTCN
ENSKNDCACVGKWVQQKGKEWESIKDHFQKRQYGNGDDIKSKVKMFLEKLQSLTELNK
IMKPCTSLDKFKASLKCNSTENSEKGGKDGKKSDIIECMLEDLDTKIKTGSCLTQPSG
EKQAECEKYTPPDDEDLLLEEENENQVEAPKICEGVIKPETKVVDEHACKTDAPQPDV
KEEEEEKEEEIVQPATTGDEGTKELPLTPPAPAPAAPSSTPATPNHQPLPSDNTSDIL
KTTIPFGIALALTSIALLFLKKKTKSSVGNLFQILQIPKSDYDIPTKLSPNRYIPYTS
GKYRGKRYIYLEGDSGTDSGYTDHYSDITSSESEYEEMDINDIYVPDSPKYKTLIEVV
LEPSGNNTTASGKNTPSDTQNDIQSDGIPSSKITDNEWNTLKKDFISNMLQNTQNTEP
NILRDNVDNNTHPTTSHHNVEEKPFIMSIHDRNLFSGEEYNYDMFNSGNNPINISDST
NSMDSLTSNNHSPYNDKNDLYSGIDLINDALSGNHIDIYDEMLKRKENELFGTNHVKH
TSTHSVAKNTNSDPILNQINLFHKWLDRHRDMCEKWKNNHERLPKLKELWENETHSGD
INSGIPSGNHVLNTDVSIQIHMDNPKPKNEFKNMDTTPNKSTMDTMLDDLEKYNEPYY
YDFYKNDIYYDVNDDDKTSMDNNNNLVDKNNPVDNNNSTYNHRNPADINKNFVHKNNQ
NQHPIEKPTKIQIEMNSNNREVVEQQYPIADIWNI
>MAL7P1.56, 2192 bases, 14751040 checksum.
MARDPRGGGSEEDDIDHKSVKHLLDSIGKIVHDQVKNGADGTAKKYIKELKGDLSKAT

FSSEETASSIETCYLVKEYYNNHVNGGDVSGERNPCRKEDVKRFSDKEGAECTNNKIN
CNKGGCGACAPYRRLHVCDKNMEKMGRTSMTTHKLLAEVCYAAKYEGASITLHYPQYQ
EKYDDSPSEMCTMLARSFADIGDIVRGKDLFIGYNQKDRKEKEQLQNKLKYIFKKIHE
KLDSEAQTRYNDATGNFYQLREDWWTANRATIWEAMTCSEDLKNSSYFRQTCSDERGG
AQANDKCRCPNGNNQVPTYFDYVPQYLRWFEEWAEDFCRKKKKYVDIVKKFCREGENG
KEKYCSLNGYDCTKTKLAVGKYRMGNQCTKCFFACYPYENWIEKQKEQFDKQKKIYDK
EIKIYTEGAPRSSSRKKRDAGGTTNYDGYIKKFYGELKNHGYGDVNNFLEKLSDEDVC
KKVQDTQGGKINFKNVKSSSASVPGGGNDVVAASGDRGKGASADSNSNKTFYRSEYCQ
PCPYCGMKKKSDGSGGWEKRSETDNCTRGNLYKPKGDAEATPIKILKSGKGHKDIEKK
LNEFCQEQNRSDGSSSGGGGKNSNNQELYEEWKCYNDVEKDGQDGVDDDDEEDVQKVK
NAGGLCILQKTNGKENVNKQKTFYDFFYYWVAHMLKDSIHWRTRRLRKCINDGTTMKC
INGCHGKCDCFQKWIEQKKKDEWKPIKDHFYKQEGFGEQGSEKLPHYMVLEIVLEEEF
LKEVSEDESENNSENPHEDAQETKRIKDMFEKKKKKNNDEVASNEETIIDFMLEEELK
DAKQCIEKHTCPPQEGLARSATDPQPRSEEKEEEEEDDDEDDEDGDEVEEEEPHTEDT
TEGSATEEKEAPKVVEPAVKKEEVNVCSIVGGILTGSGNLNDACSQKYGYPQRHWGWK
CIPSGDNTTTREGSGEATKSGATTGSGKDGATGGSICVPPRRRRLYVTPLTRLAGGDG
NTQAGETTQGNGASTETPEASLRRAFVESAAVETFFLWHKYKTVKQKELDEKKKQQQE
NVLSQLSGDTISGEQNPQSKLEKGEIPDDFKRLMFYTLGDYRDIVVRGVADDKNGGNN
IILNASGNKQDMDKIQKKIDKILKQSASKPGQEPNSKREEFWTTHGPDIWKGMVCALT
YKESKNGDKTIVKDGAVYDKFFGENNNDNPGSKPKTNGTYQENYDYNIVTLKEDESGG
GPKPAGVNEAPPKLSDFVLRPTYFRYLEEWGETFCSERMKRLKQIYEDCKVGENGDRR
RDGKKNPKCSCYGEDCEEIFSKKYDTVSSLECPNCAKYCRFYKRWIDRKRKEYDKQEK
IYVQQKSNYENESNNHDKGFCTKLKENYTDAAKFLERLKDGPCKNDSEEGKKGRDKLD
FNEPDETFKDADNCKPCSEFKINCKNGKCSDEEKRKCNGTTVITKDNIEKMKDSNGNV
DMLVIDKSGNGSQNDLKDCEGKGIFTGIRKEQWKCRNVCGYIVCGLKGDNGQKVNEKH
IIQIRALVTHWVQYFLEDYNRIKQKISHCIKNSDGSKCENKCNDKCNCASKWIDEKST
EWTNLKNLYLQQYGGNDSGESYPVKTILQELQPKTELNKAIKPCGDLHQFEESRHCNG
AASSENGKPQKKDIVECLLDRLKKKATSCPAPTSGENPTQCQEPPLVEDNDEAIEEEN
SVTQPNICPPQTPPKQEEKDTCEPAAEETEPAEEKSSEEPDKQKPDQDKSADTPARAP
AAPPSTPGPQPLPSDNTSDILKTTIPFGIALALTSIALLFLKKKTKHPVDLFSVINIP
KSDYDIPTKLSPNRYIPYTSGKYRGKRYIYLEGDSGTDSGYTDHYSDITSSSESEYEE
LDINDIYVPGSPKYKTLIEVVLEPSGNNTTASGKNTPSDTQNDIQNDGIPSSKITDNE
WNTLKDDFISNMLQNEPNTEPNILHDNLDNNTNTTMSRDNMEEKPFITSIHDRDLYTG
EEISYNINMSTNSMDDTKYVSNNVYSGIDLINDTLSGNAHIDIYDEVLKRKENELFGT
NHVKQTSIHSVAKLTNSDPIHNQLELFHTWLDRHRDMCEQWSNKEELLDKLKEEWENE
THSGNTHPSDSNKTLNTDVSIQIDMDHEKRMKEFTNMDTILEDLEKYNEPYYDVQDDI
YYDVNDHDASTVDSNAMDVPSKVQIEMDVNTKLVKEKYPIADVWDI
>PF08_0142, 2120 bases, E2B4BBD2 checksum.
MAAAGGGGKDKYKNAQDAKHLLDIIGEDIYKIANDAALKRSGSELKGLLSLAKFEKNP
PDKQTPEDPCDLDYKYHTNVTSNVIEPCNKRSGKRFSEVSGAECANNRIKGNKGSNGD
ACAPFRRLHVCDRNLEQIDPAKITATHNLLVDVCQAAKFEGQSITQDYPKYLATYNDS
PSQICTMLARSFADIGDIVRGKDLFRGYDDEEKNRRKKLEQKLKVIFGHIYEELKKHK
KLKEEAEERYKKDGDNYYKLREDWWALNRQEIWKAITCGHPGGTYFRQTACGGGTTPT
PNKCRCATNDVPTYFDYVPQYLRWFEEWAEDFCRKRKYKLENAIEKCRGKTKGEKYCD
LNGFDCTQTASGEKKFVKGHNCHNCSVTCIPFGPWIDNQKKEFLKQRNKYQNEISSNS
RKKRSTSNNNYKGYDEEFYKILKEDYGDVEQFLEKLSREGICQSQPTVGNQKADAANF
TKDNPAKTFSHTEYCQACPWCGVVCKSGNCTKNPEGSCTEQIRKKVYDDSNTTTIPVL
TPEKGKTSILQKYKTFCEKPEKHNQINNWECHYEKTDISNNCILGKWEKFQKGQEVMV
YHPFFWKWVTEMLDDSIKWRKELDNCLKNENKQCISKCNGKCDCYKRWVEQKKEKEWT
QIKDHFGKQEDMKEQIRGADPGIILEGVLDIEDLFENIKDTYGDVKEIDHIKKLLEEE
TTVDADNQNKTTIDKLLDHEDKDAKGCLQKQNECKEQERDGGARSDSQEPTPRSEVKP

DSEDLDDDDEDDPDEEKSEEVENPEDQGEEEGTKQGSGEKKVDGTEAVQETVAEVTPE
KKDEVNPCEIVKTLFEKPENLSDACGLKYGPGGKERYSQWKCIPTKPNSDNKGEVGSA
GRVARSAPSGEKGSICVPPRRRRLYVKDLETLGDSEVTQVQLRDAFIKCAAVETFFLW
DRYKKEKEKKKPQEGVLQLLGTVGTPPTDDEEDPPEKMLQKGEIPEEFKRQMFYTLAD
YKDILFGDQEVIKTLKDSGDENIKDISEKIKKTLNGDNNQESGSSPSLSGKKTTPKDW
WETYGKDIWEGMVCALTYKNSGDKKIEQVKTADDGEDLFQKLKTQYEYNTVTLKDENS
GTEGAKPFTPKTVSSSSGEKNPPKLSDFVLRPPYFRYLEEWGETFCRQRARMLDKIKK
DCNVEENDNRPGGGITKQYSGDGESCKDYLPDDPTTLPDLVSSCPKSCSSYRKWINKK
KDEFVEQQNAYTEQQNKCQSKSDKAKSDNGFYTRLQNLPDAAAFLKTLGSCSKNDIPE
YKIDFDVNGETFRYEKYCGTCPEFKINCTKVKCTSGDMQNGCKDNKINAANFKTMAQS
TEINMLVSDNSGNGSQNDLKDCKTSGIFKGFREDVWTCGKVCGYNVCKPKNVNGQNGD
GNQILLFNALLKRWVEYFLEDYKKIKHKISHCKNSSEGHTCIKNCVEQWISTKRTEWE
TIRGRFNDQYKSNDSDVYPVRSFLETWIPKIPVANANNDGKKLIKLSKFDNFCSCSAS
AHSPNGKDDAIDCMINRLQDKIDKCKEKHPQPSAENQTTCDESTLVEDVDDYEEQNPE
NKVGKPAICGNVDTTEPVKEEDEEECKAAESPAEPEQAAEEESVPAAETKDTENQPPQ
APDVGPPPLAPAPADQPLDPTILQTTIPFGIAIALTSIVFLFLKKKTKSTIDLLRVIN
IPKSDYDIPTKLSPNRYIPYTSGKYRGKRYIYLEGDSGTDSGYTDHYSDITSSSESEY
EELDINDIYVPRAPKYKTLIEVVLEPSGNNTTASGNNTTASDTQNDIQNDIPSDIPKT
PSDTPPPITDDEWNQLKDEFISQYLQSEQPKDVPNDYSSGDIPLNTQPNTLYFDKPQE
KPFITSIHDRNLYSGEEYNYDMFNSGKNGPYSDKNDLYSGNHDSLSGNRDPTSANHDS
YSGNHHPYSGIDLINDSISGNQHIDIYDELLKRKENELFGTNNPKRTSTYSVAKLTNS
DPIHNQLELFHTWLDRHRDMCEKWENHHERLAKLKEEWENETHSGNTHPSDSNKTLNT
DVSIQIDMDHEKRMKEFTNMDTILEDLDKPFNEPYYYDMYDDDIYYDVNDHDTSTVDS
NAMDVPSKVQIEMDVNTKLVKEKYPIGDVWDI
>PF08_0141, 2858 bases, CDCDBFBC checksum.
MGSQGSKPVDTSDVKNESHNSARNVFENIAEIIYNRVKEDAEKRGKSLKGDYKRAKFH
QPLLEAAKYVWYAPSNPCNFDFRFDTNAPDKGSYDRDPCHLRDTNRFSDKGDAICTNN
KINCNNGGCGACAPYRRIQLCDYNLEYINEYNINSTHDLLGNLLVMAKREGDSIVNSH
GHTGKGIYKSGICTSLARSFADIGDIIRGKDLFLGHNHKKKPLLDNLEKIFNRFQKIY
EDINNLPIDDIREYWWALNRNDVWEALTCSAPYYADYFKKKSGNTYNFTTEGYCGRNE
GAPPTNLDYVPQFLRWFDEWAEEFCRIKNIKIGNIKKSCTGESNNKHCSREGYDCNKT
NLRLNEIFMDLECPRCADDCKSYETWVENKKKEFNKQKEKYKEEINESKPGVNSEHGK
YNKAFYDKLRTIYRTNDKFFEILNKGQICENVDEKNKIDFNDLVKTFSRSEYCKSCPM
LGVTCKGEQCNSLDDITCTNDKGGPNKVTDKNNNTFVIDILLNDNKKKVLSNDLNDYK
ECDLFKRLRRQNWNCKYKCNLHVCELNNFNNEIDDERVISIKVFIKRWLESFLKDYNK
LKENLNPCINNENQLPCIKDCLENCDCVEKWIKKKGEEWKKIKERYLKDYKIEDNNSS
NSLNKFLQQNQFHSDVVKAIKPFENLRAFEDSSGCTGTVPSGIEECKSNDVIKILLSK
LKEKIELCKKNDVKKNKSNNCVTLLKPLNDEEDDEEQDDEPPAPKPLPTPNPCVNGGD
DTSGAQITSVTEIAEGMHMGAQKQMLERSGDKSGKGTENGESVLKGDIKKAKFKNGAS
PSSLEDVCGITDQHTKDSRRRRRLRRLRLLVLRFRRRLRRGHRDYKGPCTGKDGHKKM
FQVEKGWENGSKIGTENDVFLPPRREHFCTSNVEHLYRSASGLQGTTASHSLLGDVLL
AANKEAGFIKERYKTQKTSEGFKDEATVCRAIKYSFADIADIIKGTDLWKANSGKKNT
QDKLVKIFQKIKDNLPVNIKGKYNEDEKHLELRKDWWFANRDKVWEAMKCEQNGITCS
GPTPLDDYIPQRLRWMTEWAEWYCKAQKEAYDKLKVCEKCMDNGKCTQGNGECAKCKT
ACENYKKFINTWQPQWKQMEQKYESLYKEAQENGNSSHKSTTEQDKYMVEFLSQLQKA
NNGDKTGDDKVYSTAAGYVHQEATMNCEKQTQFCKNKNGVKAANDAEDVNYTFKDTPN
GYDVVCKCKDRPEQQIKKKEVEDACKIAQDIFKGKDENTTVGLCKKKDFGRTPYPDWK
CEKNSKLVTGNGECMPPRRQKLCLYFLAHKIETPNLNTQEDLRKAFIKCAAAETFFSW
YYFKKINDKLNKLDEKLKEGEIPPQFLRSMFYTFGDYKDICLDTDISLKTENGDITKA
KSNIDRIIPKKSAKNPDEERKIWWDGIKEDVWEGMLCGLSHAVSNNDKATVQKTLTTK
TIYDYDTVTFDGTTKLEDFSKRPQFLRWMTEWGEEFCKKRKEQLENLKDKCPDYTCSF

DTKKQECENHCKVYEEWLKGWKDQYKKQSEKFTTDKEKPEYKDDPDVASSENAHKYLS
KKLKQIFHNGSTTEKCDYTCMEYASRQPQTSACSQQQQQQNKSSTQNHFPEAFDYPPK
EIGDRCTCPKLPEPKYCVDKTAYDIRKESGKKSDNSLKGNGNTYNNNCNNSMREEYAN
QNGETCKFNETFWSKKKPSIEECDINAKERFQIQKYWDCNGKTPDGKNTFCIPPRRKD
MCLKQLEEINSADVNNSKKLLQKIQDVAKKEGDDIIKKLLPKYPCNEDVICKAMKYSF
ADLADIVRGIDKYKGPNGTNVLEKELKSVFETIYTKWKSENTNNKSKYTDVASFRSAW
WDANRKDIWNAMTCNAPYDAKIYITKEGGYISPLTFTKNKCGHNDDPPDYDYIPQPFR
WLSEWSETYCLAQKDLLETMKNCENCMKKNKNADCEQTQYGACRDCKRKCEEYKKFIE
IWKKQFETQNKAYQEIYRNATTTNRDNNNIVDENTKNFVKKLQENCRTDRNESLDTAD
KYLENASVCRRFKFGNKDSRHLNYAFHTDPPSYEEHCKCAKDFDPLDECPVDNNECKK
YGIGSCPKKNFHKKLEEWTNYVLNNKSNKNKSAIVPPRRRQLCLQNLTRNLSRLNKEK
SFKEGILISAASEAKMLTEQYRENPAKALQAIKYSFADIGNIIKGDDIIGNVISVQLN
KLINGNKKINTSTLWWEANKEKIWNAMMCYYTGDEKTATSCPSHGNIDKEDQFLLWFQ
EWGENFCARQKELYEHVQAECSHVICVNGTGNIDSKCTEACKNYSNFISETKNVYQSL
KKQYNDNHNSIKVGGKEVHHYMKEKCKDKCECLSEKFNSDNNWEKPYDTFDDTVLKNK
CQCKKPDSPILPVEPSVPAETEKEKKKDSPLKPHTEPKPAPPKKPEVPPPPPVQPPPA
NQPFNRDILEKTIPFGIALALGSIAFLFIKKKPKSPVDLIRVLDIHKGDYGMPTLKSK
NRYIPYASDTYKGKTYIYMEGDSDSGHYYEDTTDITSSESEYEEMDINDIYVPDSPKY
KTLIEVVLEPSKRDTPSSDAPMNKFTDDEWNQLKQDFISGILENEQKDLPKNNISGNT
PMNTQPNTLYFNKPEEKPFITSIHDRDLYSGEEINYNINMSTNSMDDTSYVSNNVYSG
IDLINDTLSGNQHIDIYDEVLKRKENELFGTNYKKNTSNNNVAKLTNSDPIMNQLDLL
HKWLDRHRDMCEKWKSKEDILHKLNEQWNKDNDGGNVPIDNRSLNTDVWIEIDMDDPK
GKKEFSNMDTILDDIEDDIYYDVNDDENPSVDNIPMDHNKVDVPKKVHVEMKILNNTS
NGSLEPEFPISDVWNI
>PF08_0140, 2980 bases, 7397299A checksum.
MVPPVRSPRAAGPAPAPASASTTYGSVKDLLEDIGKSIQKQAKDAAEKRSNGELQGDL
SKARFKDQKNTTTNPCQLNYRNHSNVRSSFENDNPCYGREEKRFSDSRSGQCTYNRIK
DSKEGDNKVGACAPYRRLHICDHNLENINDYKNINNHTLLVDVCLSAKHEGEMIANKL
KEYDKSNYESRICTVLARSFADIGDIVRGKDLYLGDQQEKLYLENNLKKIFAKIHENL
NDKIKSNYNDSEGNYFKLREDWWTANRDQVWKAITCNAPKDANYFEYNSGKFFKFSND
QCGHNNGGAPPTNLDYVPQFVRWFEEWADDFCRIRKHKLKNIKDVCRNESKPLYCSLN
GYDCTKTSWKNDFESREHYCTPCFSACSLYKIWVANQKKEFEKQKKKYEKEIPKYVSK
KVKTNSTINNYYYYDFYEKLQKNEYGNIDAFLNLLKEGKYCKENLPEEEVINFTKHDE
KKTFSLSNYCVVCPDCGVEYKNGRYTAKDQKYPDCRNEKYDPNNAETTDITVLYSGDV
GDFSEKLQDFCNDINNGKVKNYQIWQCYYENSEINKCQMTPSSHKVPKHGYIMSFFAF
FDLWVKNLLIDTINWKNELTNCINNTNVTDCKNDCNTNCKCFENWAKTKEKEWENVKT
IYKNENRNTNNYYKKLNDLFKGYFFHVMYELNNEEAKWNKLMKNLRTKIDSSRKNAGN
EDSEGAIKVLFDHLKDIAERCIDNNSIKPCNSHPKPTPNPCGKNNNGGKLVRVKRPAE
LKQQKAHIQLEKRGGESNLKGDATRGTYNLGCQGDQLDATFCNIDEKYSNRIPRKSEG
PCYGKNPQRFYTGKDWTHVVQEKTSYKDVFLPPRREHMCTSNLENLNLNSKGLSNSSI
ASNSLLGDVLLAAKYEADDIVKKNDSDKSSICNAMKYSFADIGDIIRGKDMWDLDSGS
KDMEKHLISIFEKIKENLDVIKYNSKYKDTQKFLDLRSDWWEANRHQVWRAMKCAIEN
DKDMKCNGIPIEDYIPQRLRWMTEWAEWYCKAQKKEYDELVKGCKGCMGNGQGCTKDS
SDGECKKCTEACNTYKQKIKTWENQWKKIKGKYEKLYEQATKNGETSGTPNEKDKDVV
DFLKQLLPRNSAAARNRVIRAAGSSAPGVTALTPITLYSSAAGYIHQELGKTVGCNTQ
KEFCKNGSGEKYAFKHPPKEYKDACSCNTRDKKSEAPVTKKEEACKIVDGILKGNDGI
NEISGCNPKESYPEWQCDKSKFKNGEEGACMPPRRQKLCVSGLTQGGKITTIEDIRTQ
FITCAAIETHFAWHKYKKDNVNAERELNSGIIPHEFKRQMYYTFGDYRDIFFGTDIST
HNHIPEVSQKVITILEKENLTKSEVKQKYKNELLDDWWNEHGKEIWDGMVCALCYDTK
TKEFKNEVRTQLTGPNSKYQYGTVSFGDKQNTILSKFAERPPFLRWFIEWGEEFCKKR
KEQVDQLVNVCKECNVSDSVISSGNKTCNDKEKCDACKKECTQYENWLQTWKGHYNKQ

KERYAKVKSTSPYNNDNDVNKSTHAYEYLSKKLTNIPCTNGNTTGKCDYKCMDQRTST
DGMPASLEYPPKEINGKCDCTKALPPQEPPSTPESRGRSLNPPPDSPQPPRGPEEQEE
SASYSEEEEEEEEEEEENDEENDGDEEEVKTEDGGSDDEDEDEEDDEEEEENEVDVDE
PQQEEVEEPPAKESAPTTQEPAVPEVKKQEVKVCDIVSKILTKDALQDACKLKYNGKY
YGWKCISGATTGGLCIPPRRRKLYVTPLTKWATTAVSSEATPAPSQSESDKLREAFIQ
SAAIETFFLWHKYKQEKKPPSEQNAGAALVLLQGVTPQEDPQNKLKSGTIPEEFKRQM
FYTLGDYADIFFGKNDILIGKTGTGSAKDEMADKESKIKEAIKKFFQNGDSQPLSGKP
VTQSSDKRTALWGDFAQDIWHGMICALTYTDSGEKGELPKEDDKVKDKLWDDTKKQPK
KEEYQYDKVKLDENSGDGPKTSQPPSAPSDTPTTLDSFIKRPPYFRYLEEWGQNFCKK
RTEMLGKIKGDCRVEDGSKNCSGYGEDCDDQLGEDPSTVPSLKCQSCGEECRKYKKWI
ERKKDEFTKHSNAYGDQKAKCQAKSGGGGNGFCGTLERCSKAAEFLKTLGSCKTNNGN
ESVEANKTIFDNIGETFKPATNCKPCSQFKIYCKNCKSSGDDTNVGCNCKENGNGYIT
SDHIKNEDNFTDPVDMLVSDDNTNGNKFADLQACGGAGIFKGIRKDQWKCRNVCGYVV
CKSEKGNGETVRGEKNDVKHIITIRALVTHWVEYFLDDYNKINKKLNRCMKDGEGYQC
IKDCVEKWIQNKREEWKKIKELYLQEYKNNNQPDYLVKIILEELHPQTQLNEAIKPCK
TFDDFKSFCGLNGAESSKKSKNSNDNDLVLCMLNKLEKKIDECKQKHQASGKNTAQCQ
EYPPLPDEEYENEEENDKKVEPPTFCEIKDTPEPADEDACKAAPKETAPSSEENQPET
PVIKPEEEAPAPEEPPPAPPPLAPSDESILHTTIPFGVALALGSIAFLFLKKKTKSTI
DLLRVINIPKSDYNIPTKLSPNRYIPYTSGKYRGKRYIYIEGDSGTDSGYTDHYSDIT
SSSESEYEELDINDIYVPGTPKYKTLIEVVLEPSGNNTTASGKNTPSDTQNDIQNDGI
PSSKITDNEWNTLKDEFISQYLQSEQPKDVPNDYKSGDIPFNTQPNTLYFDKPEEKPF
ITSIHDRNLYSGEEHSYNVNMVNSMDDIPINSHNNVYSGIDLINDTLSGNQHIDIYDE
LLKRKENELFGTNHVKQTSIHSVAKPISDDPIHNQLELFHKWLDRHRDMCEKWENHHE
RLAKLKEEWENDTSTSGNTHPSDSNKTLNTDVSIQIDMDNPKTTNEFTYVDSNPNQVD
DTYVDSNPDNSSMDTILEDLEKYKEPYYDVQDDIYYDVHDHDTSTVDTNAMDVPSKVQ
IEMDINTKLVKEKYPIGDVWDI
>PF08_0107, 2265 bases, A81DAC10 checksum.
MPPLGRQVGGSPQDDDAKNMFDRIGKDVYDQVKKGVAETYKEALTGQLSLATLLGVES
ASTTDPCGLDYSKLISGSGVAARGHPCGNESVSEKRFSKERVDEYDEKKIKDNKGNRG
NNEGECAPYRRLSLCNKNFQKINNIDSDKARHNLLVDVCLAAHYEGDSIKAHLEQYDA
TYPGSGSTTCTALARSFADIGDIVRGRDLYRGNKKKIKTETERDKLEQKLKEIFKNIK
KENNEKLKSLTDDQIREYWWTANRHTVWKAITCKADASSAYFHATCSDSHRSGTFSQA
NNYCRCNGDQPGNDKENIDPPTYFDYVPQYLRWFEEWAEDFCRKKKHKLNDAIQKCRG
EDKYGKDRYCDLNGYDCEKTKRGRNMYRWDHKCTGCFLSCSHFRTWIDNQKLEFLKQK
EKYTNEISGGKSRKKRAARSSSSSSYDNGYEKKFYDQLKAGGYNGVNSFLDLLSKEKT
CKDITDTEGGKIDFKNVNSDKNSYDDDSNKTFSHTEYCQACPLCGVKRNGRKWERKEV
MKDCPPINLYKPKKDAEGTLINFLYSGDETNEIAKKLKAFCAQANGDTTNGTGGNGTG
GSVAGGTGTSGSNELYQKWKCYEIDELTKDQKEGGEDDPVYDEDVKTGGGLCILENKN
KSKGSQSNSQKEPDEIQKTFNPFFYYWVVHMLKDSIHWRTKKLDKCINNSNESKACKN
NNKCKDDCGCFLKWVVQKKTEWENIKKHFKKQKDIPPGFTHDDVLEGVLEKEVLLTSI
KEGYGNEKDIKHIKKLLDEEEAAGVTDNENKTTIDKLLKHEKDEADKCKQIQEECNKQ
KQQERGGPGGRSADPSPPADLDEHSSDEGEEDDDDDEEEENANEETAEDATEDTQVDG
TEVVEETVAEVTEVTEVKPCEIVQKLFTNGDLQNTFKDACEQKYGYPQRHWGWKCISD
TTTGKSGDNTGSSGAICIPPRRRKLYIGKIKEWANSGNTQAAEPQEDGKAQTPQGQTP
SQSGKESSQSQKLREAFIQSAAIETFFLWDRYKKVKNKEIAEKKQRDSTNPFGFTQST
SGGMQALPVGGAVQGAQAQLPDGAGGSDQTPEKQLAGGKIPEVFLRQMFYTLGDYRDI
CVGNTPSGIDTNDKENMQKIQNKIKSVIEKSDSTPRTPGTHSPSSGTTPQALWDKISP
SIWEGMLCALTYKDGGEGKKIEKVNDANGDDLFQKLKDKYSDYKTVTLEEDNTSSAMS
TSPRTSETTSASSDNTPTLNNPKLSDFVLRPPYFRYLEEWGQNFCKKRTEMLEKIKEE
CRSGTGGHEYCSGDGHDCTDNDRKYNKMIADFHCPGCAKECMKYKTWIGKKFEEFKKQ
ENTYKVEHGKLTNNNCSGSDNTKFCQQIKNNSFDKFLELLRHCKDGKDDTDKDNELNF

SKPLQTFSRSTYCKACPIYGVKYNRGTYSAINESEYMSKNGISGENKNDKKPTEIKVL
LLGRKGEVRNNDEELKELKEVCNNAGFVEDYSLQKWNCQKKNGVDQCNLTNSVDNIDD
SDKIIPFNVFFQRWLRNFVHDYNKLKQKIHPCIKNEDGKEHKCIEGCKKKCECVEKWL
DKKSTEWDEIKKHYEKHSKDHNEGIPHSVKGYLVQAPFKDEVLKAIKPCDFDNFKTSC
GLNGTDNSQNGNNNDLVLCMIKNLEKKIEQCKKKHDENKKTPCDPLPPLPDENDETLE
ENPVAQPNICPQLPEEPKETCEEAVTPSEPKKAEEEPAAEPEAVPEEEAPLPPPPPAA
PPRESKEKAKPPPKPRIKTRHVLDHPAVIPALMSSTIMWSIGIGFAAFTYFFLKKKTK
SSVGNLFQILQIPKSNYDIPTKRSPNRYIPYTSGKYRGKRYIYLEGDSGTDSGYTDHY
SDITSSSESEYEEMDINDIYAPRAPKYKTLIEVVLEPSGNNTTASGKNTPSDTQNDIP
TSDTPPPITDDEWNTLKHDFISQYLPNTQNTEPNILHDNLDNNTHPTPSRHTLDQKPF
IMSIHDRDLYSGEEISYNIHMSTNSMDDPKYVSNNVYSGIDLINDTLSGNKHIDIYDE
VLKRKENELFGTNYKKNTSNNSVTKNINSDPIDNQLNLFHTWLDRHRDMCEQWNNKEE
LLDKLKEEWENETHSGNKHSDIPSGKLSDTPSDNNIHSDIHPSDIPSGKQSDIPSDNN
IHSDIPYVLNTDVSIQIHMDNPKTTNEFTYVDSNPNQVDDTYVDSNPDNSSMDTILDD
LEKYNEPYYDVQDDIYYDVNDHDTSTVDSNAMDIPSKVQIEMDVNTKLVKEKYPIGDV
WDI
>PF08_0106, 2231 bases, BB909D18 checksum.
MAASTTYSSAPDAKHLLDMIGKDVHDQVKKEAKERSKGELTGQLSFASILGERAYTTD
PCQLIKEKRENLIGDRGERHPCGNGSGKGEDVNRFSKERVDEYDNKKMKCSYGSNGKS
EGACAPFRRLHVCDKNIQQIKTENITTHNLLLDVCLAAKYEGESLKGYHEQYEVQYPS
SGSTMCTELARSFADIGDIVRGRDLYGGSKKEKEKRKQLDDKLKKIFENIKKENNEKL
KSLTDDQIREYWWTANRETVWKAITCDEENKLGGYLYFRGTCGDNEKSVTQARDKCRC
KKKDNTPDDQVPTYFDYVPQYLRWFEEWGEDFCRKKKKKVENAKKKCRGENNDKYCSR
NGCDCEKTVRAKGKLRYGNRCTDCLYACYPYVHWIDKKKEEFDKQVKKYDEEIKKYTK
VASGSSRKTRAATIKYEGYEKKFYEKLKESDYGKVDKFLEKLSKDKECEKVDDEEGGK
INFAEKHDNNNNDEKEGTFYRSKYCQPCPYCGMKKKVPGKEWEKKNDNCTSGKLYEPK
GDAEGTPIKILKSGENHDDIKQKIDDFCKTEDDESLYAAWKCYKHDEVQKVKREGEEE
DDDEEDYKNVKAAGGLCILENQKKKEEGKEKKSEKEPEQFQKTFNDFFTYWVAHMLKD
SIYWRTKKIKKCLENGKTMKCRNGCNNDCDCFESWVKQKKEKEWKPIKDHFGKQEDIK
QETGMDPGDFLEYYLKLQFFEEVSEEKSQTGDEDANEKKRIKEMFDKKNQRTDKVASN
EETIIDFMLEEELKDATKCKQDCEQRKPPGAGGGDVRSDTVPSRDPLTPGPTDGHEDS
EDEEEEEEEEDEEHGPDDKEGTEENVEETETVEDQVEEEKAKDNTDGKGEEEEDNQGE
TTQITTQDGVNPCDIVDKLFKDDNTLTNACPTKYEKGREKFPNWKCIPSGDKTDTRER
AGRSRRDTSGENTTTGSSGDTNGSVCVPSRRRKLYIQKLHDWAEKQVGTTQVDGKAAQ
GDAASKDPKVELRNAFIQSAAVETFFLWHKYKAENTKTQGGVGSLPLQTIDRNSANGD
DEDNPEKQLKEGTIPHGFLRQMFYTLGDYRDILVGKYVDNGKDMEEVKSNIDSFFSNG
EKPDDKKGVEQRKEWWKENAKHIWHGMICALTYDTNTASGDKHTLKQNKSLKEALWDE
QKKKPKKTDNGSDYTYESVVLKEDDEGGGPRGQTSSPSSGEKTTLNNPKLSDFVLRPP
YFRYLEEWGQNFCKERKKRLEKIEEECMEYGSRGKQKQKYSGDGEECEKVLVEDANIF
SDLSSSCATPCRKYRKWIERKKYEFTEQYNAYGGQKNNYVNEQKDKCQTQSNNHDNGF
CGTLEKDAAAFLNRLKNGPCKNNNDNGEEHKIDFNDTKKTFKHTEYCGPCSEFKVKCK
NGACGDSNTQKKCNGITPIDGNEIAKMITSTEEVVMRVSDNSGNRFDGDLKEACNGKG
IFKSIKENKWECRNVCGYVVCKPKEGNRETASGENKDQIITIRALVTHWVQNFLDDYK
KIKHKISQCTKTDQGSTCQNKCQNKCKCVGEWIPKKREEWEKIRKRFLEQYKIENDED
FNVRSCLENFLVQIGAANAKNKVIKLSKFGNSCGCSADASAQKNDGHKDAIDCLLQKL
QNKIDDCNKNQAQNSVETQPSDENPAQCQDTHPDDDLLLEEEQNPKNMRPGFCPEDDT
TEQQEEEQDTCTPAGTVKEEEEEKEEKKDKGDEEEESPSGNSAPDPPESPSAGPNHNQ
HPPAIPTPATPAADPPQADEPFNRDILEKTIPFGVALALGSIAFLFLKKKTKSSVGNL
FQILQIPKSDYDIPTLKSKNRYIPYKSGPYKGKTYIYMEGDSDEDKYAFMSDTTDITS
SESEYEEMDINDIYVPGSPKYKTLIEVVLEPSGNNTTASGKNTPSDTQNDIQNDGIPS
SKITDNEWNTLKHDFISNMLQNEPNTEPNILHDNLDNNTHPTMSRDNMEEKPFITSIH

DRNLYTGEEYNYNVNMVNSMDDTKYVSNNVYSGIDLINDTLSGNKHIDIYDEVLKRKE
NELFGTNHVKQTSIHSVAKLTNSDPIHKQLDLLHTWLDRHRHMCEKWNNKEELLDKLK
KEWNKENNTNSSLTHTSNIPSGENSIKNVLNTDVSIQIDMDNPKPKNEFTNMDTIPDK
STMDTILDDLEKYNEPYYYDFYKDDIYYDVNDDDKTSMDDIYVDHNNVTSNNMDVPTK
MHIEMNIVNNKNEIFEEEYPISDIWNI
>PF08_0103, 2109 bases, BC463864 checksum.
MSTLGGGTDKSAKHVLDEFGQQVYEQVKNGEAKTYFDELHGDLSEATYPGDENPNKTT
PPNPCLLQYDYNSNVTIGGGREYPCKDRPEVRFSDEYGGQCTDSKIKGNEDNKGGACA
PFRRLFLCDQHLSHMKAEKINNKHNLLLEVCLAAKYEGESLKGYHDKYNATYSDSRSQ
LCTVLARSFADIGDIIRGKDLFIGYDKKDRVQKKKLQDSLKNIFGNIYNELTTSGKNV
DKAKARYNDPKGDFFQLREDWWALNREKVWSAITCNAQGNKYFRPTCSGGESIAHNKC
TCINGDPPTYFDYVPQYLRWFEEWAEDFCRKRKHKLQNAKEQCRGKNGEDKYCDLNGY
DCKRTISAEKKLFPDSDCNKCSYSCIPFRTWIDNQKLEFLKQKNKYDKEKEKNNDTTK
TTRYGPINNLYAKDFYDKLKQQYITVDSFLELLNKEKECKNHPEVGDGKKTFVDFSNK
NVDETFSRTEICEPCPWCGIEKQEDGKWKRLNENAPECPQEIEKTYPESNTTDIPVLT
PEKGKTSILQKYRIFCQNAENNKQIKEWQCHYEKNDKSDDSDETHNSDNCILGKWENF
EKGQEFKSYYSFFYGSIIDMLKDSVDWRDKLNNCINNETKACKNGCNKNCDCYKRWVE
KKQQEWSNIKKHFGKQGDLLEEIKGEDPGKILEFYLKSIFLQDMKEAQGDPKAIKRFT
DLLQKKNNPGTDDTTKTIIDKFLQEELTDANRCKETHKDDCSQQEVTRLRSADPSPDT
VDSASEDEDEEDKDHQEETEDTAQDTGQGEEETATEKVAPTVVDVCATVAEALTKGDL
NAACTLKYGTPNRYWGWKCIGDKTATGEARAGRVARSPPETAPSSAKSGEPTGSICIP
PRRRRLYIQKLHEWASRGGDEATKSQSQAGGSEAQPQGGEKSPSGKVSSQSDKLRTAF
IQSAAIETFFLWHKYKAENTKTQSVGSPLLLLPQLPRSGSDDKDPETSLKSGTIPIDF
LRLMFYTIADYKDIFEGKNMEVVNLLKDGSPSDKEMQERESKIKDAIDKVFPNSDNKK
HSGVPSQTGNTTPQTLWSKYAEPIWNGMICALTYRDSEEKGGTPTQNNTVKTELYDKN
TKENGKYNYHTVTLEDDSDETRPKIGTSPSGEKTYLSKFVLRPPYFRYLEEWGETFCR
ERTRRLEKIQGDCTQGDDEYKCSGYGENCKDIREQDYSIISNFNCPDCGKSCRSYKKW
INIKKDEFTKHSNVYNEQKEKAKNNKDPESKSGNISDHEFVGKLDKDYASIDSFLEKL
GSCSKNNKDNGDGTINFKEPDVTFKPADNCKPCSEFKVNCRNGNCKGANGNTCNGETV
TAEEITKMSDSTVIDIRVSDNSENVFEDILDECQNAGIFEGIRKEQWTCGYVCGVDIC
EQTNVNVNQNDKEYIQIRALLKRWVDHFLEDYIKIKHKISHCIDNGKGNICKNKCNDK
CNCASKWIDEKRTEWKTIRDRYFEQYKGAQSDVYDVKGFLEDLQSQIPVTINKAIEPC
KDLGEFERSTHCNGAASSENGKPQKKDIIECLLDKLEKKTKKCKDDHPQPSAENQAQT
CENSAHVEDDDEPLEEEGDQNPVGKQQPSFCPPVEDKKKEEEGETCTPASPAPAPAPS
EDPPVPAPAGDQKEASTPKVAPRPKPPRVKPQQPGDDPWEPLKNAMLSSTIMWSIGIG
FATFTYFYLKKKTKSSVANLFQILQIPKGDYDIPTLKSSNRYIPYASDRYKGKTYIYM
EGDSSGDEKYAFMSDTTDVTSSESEYEELDINDIYVPGSPKYKTLIEVVLEPSKRDIP
SGDIPHTNKFTDNEWNQLKKDFISNMLQNTQNTEPNVLHDNVDNNTHPTMSRHNVDQK
PFIMSIHDRNLYIGEEYSYDMSTNSGENNLYSGIDPTSANHDSYSGIDLINDALNGDY
DIYDEILKRKENELFGTNHTKKNTSTNSVAKNTNSDPILNQINLFHKWLDRHRNMCEQ
WDKNKKEEFLDKLKKEWNKENNNNSGDINNRYENVLNTDVSIQIDMHNPKPKNEFTNM
DTNPDNFIKDTILNDLEKHREPYFYDIYDDDITYFDTDDVKPPMDDIHIKEQTEMNAL
HNNKMNELLEKEYPISDIWNI
>PFI0005w, 2270 bases, 24AEEC12 checksum.
MGPKTRAPDYSSAPDAKHLLDMIGKDVHEKAKSEAKNYIEELKGDLNTANGRSSETRY
TTDTCTLVKEYYTTRLGVNNERNPCKGLSGIIVERFSDTLGGQCTDHRIKGNNRNVTG
GACAPYRRLHVCDKNMEKIPTSKTSTDTLLAEVCYAAKYEGDSISGRYRQHQLTNKGS
QLCTVLARSFADIGDIVRGRDLYLGYDDKEKEKRDQLESKLKVIFGNIYGNLDKNNRY
NDDTPEYYKLREAWWDANRAKVWEAITCDAPDNAKYFRGTCGGDDEKTGTQAIHKCRC
TKSSGGKPGKNETDQVPTYFDYVPQFLRWFEEWAEDFCRKKKHKLKDVKTNCRNDEEQ
RYCSGNGFDCTKTIYKKGRIVIGEHCTKCSVWCRMYESWIDNQKKEFLKQKRKYKTEI

SGGDGGGSSRKKRVARSSSSSDDNGYESKFYKKLKEVGYQDVDSFLEKLNNEEICKKI
TDEKENIDFKTADNSLNKNINKEGTFYHSKYCEVCPGCGVERNGSGWEEKSGGTCDVK
KPYKIRDDAPFNDINVLSFGDKGNEIKQKIDKFCKTQNGNSGKASSSGSGDCGTNIDS
SLCEPWKCYKGEDVMKPEDDDDQQDYENMKNAGGLCILPNPKKHESEKKTADEPAEFQ
KTYNDFFYFWIRRFLNDSMYWRGKIGGCLKNKSEKCKNECNTNCDCFKKWIGKKKEEW
DKIKKHFKTQDFGSKGFLGQFGYDEVLKYVLNIDELFKDIKDGYGNAKELEGIKKLLE
EEERKNQVEAADGGASSGKDNTTIDKLLKHEEQDATKCKDCKPPEESLARAEVAGDNI
PKKEDSDEEEEEEEEGTEGTGEVEENVEEDNTENKDQEDVSGEGSKEAEKEPTAKKDE
AEKVCETVKSALTGDDLTKACQQKYQYGKEKFPNWKCIPSGDKTDTSSESESPPRQRR
DTTSGVVTTAPSSAKSGDTTGSICVPPRRRKLYIGKIKEWAGIQLKSQVEGGGTGKQS
SQLQQQQQQQEQQQQQQLKPQSPVASNSTLTTSAKQSPSNPRADDLRDAFIQSAAI
ETFFLWHRYKKIKEKEKKEKQEEAQGNVYTQTDENKNKDPQNELNGGKIPDGFLRQMF
YTLADYKDILEGKNIVVDMLSGNDKDTMKKIQAKIDEILPKNGTSPGSSSGNDPESWW
KNHGEHIWNAMVCALTYTDSEAKEQPPIQDKEVKKALLDKEGASNEPISQYQYKTVEL
KEESSDTKPQIASAPSDTPTLNNPKLTEFVEIPPFFRWLHEWGSDFCDKRARMLKNVK
KACREKGDDEYKCSGDGHDCTKNGNLGHHKMLEDPNCPRCYEQCRKYRKWIDIKFEEF
HNQKHKYQGEYDKLKVNSSVDYKKLQGYSTAADFLKELRHCKDGQTSEEKDNDNKIDF
KEPLKTFGHSKYCEMCPSYEVNCNGRRARSGGNDQCTPVNGNGNTWDSVFEGISGNGE
NSTITVEMIDRRGPFIKEYSKDLKNSEQSKDSNDLFKTSRLFKGIRKQEWTCKYKGEN
MDLCKLNNFNKVIDLNDYTTFKVLLIYWLEDFIEGYYILKKRKVFEQCKENGGNKCSE
ESKKNCACVKVWVDQKKEEWDQIRTHFNNRNQRKGDDDMKSSVKKILDPLIYRMDLKN
GKRKFDELTDFLKAYECKCVDNAGNSEKDVVECLLENLKTKATSCQTQHQPSGKPGQT
CENPITPPDDEDLVLEEEQNPENMRPGFCPPQTPAQQEETDGTCDAVAPGAAKKAEEE
SGIPPAAEPEADKGPEQADTEKKVPPKAAPARPKDKKRPKRQPRNVLEHPAVIPALMS
STIMWSIGIGFAAFTYFYLKKKTKSTIDLLRVINIPKSDYDIPTKLSPNRYIPYTSGK
YRGKRYIYLEGDSGTDSGYTDHYSDITSSSESEYEEMDINDIYVPRAPKYKTLIEVVL
EPSGKNTTASGKNTPIDTQNDIQSDGIPSSKITDNEWNTLKDEFISNMLQSEQPNDVP
NDYSSGDIPFNTQPNTLYFDKPEEKPFITSIHDRNLLSGEEYSYDMSTNTMDDPKYVS
NNVYSGIDLINDSLNSGNQHIDIYDELLKRKENELFGTNHVKQTSIHSVAKPISDDPI
HNQLNLFHTWLDRQRDMCQQWNNKEEVLDKLKEKWENETHSGDINSGIPSGKLSDTPS
DNNIHSDIHPSDIPSGKQSDIPSDNNIHSDIPYVLNTDVSIQIHMDNPKPINQFTNMD
TILEDLDKYNEPYYDVQDDIYYDVNDHDTSTVDTNAMDVPSKVQIEMDVNTKLVKEKY
PIADVWDI

(2) $\mathbb{S}^-$: 252 non-secretory proteins

>MAL13P1.164(Q8IE20_PLAF7)
MLKSKCIFLNSFTKSDKLKHTFERLNLMMEKRNMNNLLYNKAVECHIYIRRDRDTKET
NKKLNNMYNKYSCKCNITNKCSNKNKVYNIYKRQNNLVDNKDKFFCIFRQNFAIGIFE
RKKPHMNIGTIGHVDHGKTTLTAAITKVCSDLNRGVFKSYEEIDKTPEEQKRGITINA
THVEYETEKRHYSHIDCPGHLDYIKNMITGTSQMDGSILVVSAYDGLMPQTKEHVLLS
RQIGIEKMIVYLNKIDMCEDQELVDLVELEIRELLSFHKYDGDNIPFIKGSALKALNG
DQSEYGVPSILKLLDACDNYIEEPKRKTDLPFLMSIDDVLQISGKGTVATGKVEQGTL
KLNDQVEILGIKEKSIKTVITGIEMFRKILDTAQAGDQIGIMLKNVKRNDITRGMVVT
KAPNIKTFKKFESDIYVLKNEEGGRKNPFSSYYRPQAYIRTADVNCAVILNEDTQVAN
PGDNVKCVIELMYPLALTYGLRFSLREGGKTVASGVITKLL
>MAL13P1.166(Q8IE16_PLAF7)
MFEKKKKKVKNKNSIHLQCYRRLGIHRKKYFNFSNQLIYISLSGHIYTYPSYEGVLQA
DKYILDELCITKFGESSAITDIKLLYKKIYKNDYDPSKYTKQNFEHYYIPPSIIKAYN
ELGIHKLYKEQAECLCKIFLNDDIENKIKNGIYMNHGNNEEFVSNENTHCKSNVEVNT
DISMNLDFFCDMNIVDMKNIKYTPGKKEDIQIDEFTDLIKSDITFGKKKNVDICNHMD

NKMKVYTNEYTNEYTNEYTNEYTNEYTNEYTNECSNEDTNEYNLNNSHSKYDTLVNVE
NSSYKKRSQICYWSNNNNNNNNNKFYNNFLFKIPTGMGKTLIYDILIIRQVLYKGFRVI
LTLPTLSLINEKYEYYDKLFGDQTVSLNIKKFNSNDFTGYSYSLSTDLALCTFEQANT
ILSIIIKNNLKFNYLFILDEIHYINNQKRGFHIESLLTKIKYIQKNFSHIYNIKVYGF
SATISNIDQLAEWLDANVYESKTKLQRIKYLYKIDNSLYKDINKNEVERTLENCNTLD
PNHLGYLISEEFILKKNVLIFCPSKNKSEQTASFISSTLPYYLNKRDYKINMELQEKR
FKLLNDLKGLTIKVANVEKMIMNGIFYHHSGLNINEKSLIENSFRNNILFCLCCTTTL
SVGINMNIHTIIIRSLKLGKSFITKDEITQMAGRCGRSQKKLYDSTSSCKNFDESTNK
SYMTPDRRYTDSSGLPPILDYDYDCDGKVIIFVSNVEKHYLEKILYDEEEMSKLKTTL
NNIQMCKFLLDFIHLKLIKTKKDMFDFLYLYSIKFFKAKRVEGNTNDDDQNNGINMNI
NNNDKCIHNNNDKCCSNNNNNDKCCSNNNNNDKCCIHNNNDKCINNNNDKCINNNNDK
CIHNNNDKCCIHNNNKCSNNMNEKILIDVKQTFQYLFENKLITIPYEQEKNYYEHVFN
KIFHINFCDINKIFTFQYINQNINPNILMKYNITQKINIFKKLYHNYKGKNITTDKQT
SFFTLPFITILLIFKDIEFNKNLFQNLYVEFIKYIFLMNINLQQYDLFVYDILKDDDP
IACTELFSYVQSASSIMEFIFNYSLVQYIYVKGFLPDVLLMIFAFCINSEINLKIHFD
IYEQILMSHNKNIKNVFQFFGLNVEKLKNFELKNVDDLFDASANILKGKCELLKL
>MAL13P1.171(Q8IDZ5_PLAF7)
MKKILWLCLVIIFEIIGYNGLEIYIKMKGKKLKCIKERINKDTLVISKFKTNHKNALV
SLYIYDVDVNEKNFHTQPKLPIFETVNKHDIKTAFTTFYSSSYSFCAYNNSDKLIEVY
FEIKYGTEARDYAQVAKTEHLNQGTLYLKQILDQMNIFHVNLKRIRSDEDKEKKAHDK
LNDTLMWFSFLNIIIIVIAAIVQDFYFKKFFTSKKII
>MAL13P1.184(Q8IDW2_PLAF7)
MNHISYKLRGIKCYRTHLRGTIFKRNYFWEKVNRESGLYKIWNKLNLKNLVKKSCNKL
IIHDKKEKIYNNNNNNNNIINENIPSSCFYDYVGSTDILNDDEKNKIDKILEDINRRN
KEIGGYLLELNGIKNNGDGIIKCSYACIRSCDNILSKICKEENIFKIINMVDTVSNNL
CKLGDVLELLRNLHNNKNVIGKAHEALEKLTNYIDKINIDTDIYNFLKKKYNENIHLL
NHEHKEVLHNMIVSMETQGVHIKDQKERERYLELQSQEKYFAFHASSNYSDEFKGIYI
EKNKLLKYINQKCLKDYEDKLIPYIKNNQIKVEKKYPFNEYIYILQDSSFLMTILKNV
NDVEIINQVYTLLREPNHTFLNNILILQYYRNRLINYRNFKNYNEYSLKDCILNEPRR
VNYFLKNFLHKILPYFFKELQFIESYISLITLRKKEKKSVNTIKKNLENYNHKDEIPK
LNASNIYYYMNEIKKVRLKNIEAEMKNNLSLYDVIKFVITLLKNSYSLEMINVNPLKN
ELWDENIIKFEIKRGHYVYGYIYMDLFERENKNNSIAQYTVRCSKNMNYSLKYKWLEE
NADQCSFVYTGIVKDEYFKNDDKTKCAHNEKFKEKINNNNNNNNNNNNYYYYHNGDNT
YEYANSNKNINLNNNHENNSLLNSSYRQTTSTFLVCNFNVNICDKEKDNNYESYDNDD
NLFLNKKISFLLEKICMSIDKVNIFLHEFGHTLHCILSSTYLQHLSGNRSGVDFSEFS
SHFFEEYLNCYDALLLLYNNKKNKKNEEYMKSLLSNYMKNKNIICYYSIVQLTIQSII
DQIFYAFSSNSNNMIDRKESIEKQIKSYFEGIYYKDIHILDFFPQIHFSKTTHLVHYP
SNYYSYLYCSVLAKYIWNMTFKDNLFNTEKADKIVNFLQKGSVDSSLRNIISLVENDQ
SKIDYYTENPHKIPLNDFLDYYQENKEDKYTSRSSQFNQIMRFLIKNLLFFNIYNKKI
YYILSSYEKTCYNIMNYAINMKHSELINFVLLFFL
>MAL13P1.186(Q8IDW0_PLAF7)
MIFNYVFFKNFVPVVLYILLIIYINLNGMNNKNQIKTEKIYIKKLNRLSRKNSLCSSK
NKIACLFDIGNDDNRNTTYGYNVNVKNDDINSLLKNNYSNKLYMDKRKNINNVISTNK
ISGSISNICSRNQKENEQKRNKQRCLTQCHTYNMSHEQDKLANDNNRNNKKNFNLLFI
NYFNLKRMKNSLLNKDNFFYCKEKKLSFLHKAYKKKNCTFQNYSLKRKSNRDSHKLFS
GEFDDYTNNNALYESEKKEYITLNNNNKNNNNKNNDNKNNDNNDYNNNNSCNNLGERS
NHYDNYGGDNNNPCNNNNDKYDIGKYFKQINTFINIDEYKTIYGDEIYKEIYELYVER
NIPEYYERKYFSEDIKKSVLFDIDKYNDVEFEKAIKEEFINNGVYINNIDNTYYKKEN
ILIMKKILHYFPLLKLINNPSDLKKLKKQYLPLLAHELKIFLFFIVNITGGHFSSVLS
SLEIQLLLLYIFNQPYDNVIYDIGHQAYVHKILTGRKLLFLSLRNKKGISGFLNIFES
IYDKFGAGHSSTSLSAIQGYYEAEWQVKNKEKYGNGDIEISDNANVTNNERIFQKGIH

NDNNINNNINNNNYINPSDVVGRENTNVPNVRNDNHNVDKVHIAIIGDGGLTGGMALE
ALNYISFLNSKILIIYNDNGQVSLPTNAVSISGNRPIGSISDHLHYFVSNIEANAGDN
KLSKNAKENNIFENLNYDYIGVVNGNNTEELFKVLNNIKENKLKRATVLHVRTKKSND
FINSKSPISILHSIKKNEIFPFDTTILNGNIHKENKIEEEKNVSSSTKYDVNNKNNKN
NDNSEIIKYEDMFSKETFTDIYTNEMLKYLKKDRNIIFLSPAMLGGSGLVKISERYPN
NVYDVGIAEQHSVTFAAAMAMNKKLKIQLCIYSTFLQRAYDQIIHDLNLQNIPLKVII
GRSGLVGEDGATHQGIYDLSYLGTLNNAYIISPSNQVDLKRALRFAYLDKDHSVYIRI
PRMNILSDKYMKGYLNIHMKNESKNIDVNVDINDDVDKYSEEYMDDDNFIKSFIGKSR
IIKMDNENNNTNEHYSSRGDTQTKKKKVCIFNMGSMLFNVINAIKEIEKEQYISHNYS
FSIVDMIFLNPLDKNMIDHVIKQNKHQYLITYEDNTIGGFSTHFNNYLIENNYITKHN
LYVHNIYLSNEPIEHASFKDQQEVVKMDKCSLVNRIKNYLKNNPT
>MAL13P1.196(Q8IDU4_PLAF7)
MKLSSCITYVSILCNLIIVGRFLYSSFLFLFSENILNYFERNNPFIHRKKYLIHKRTQ
PILHSSKYRHPYEGSFKKKVIYDNNYFGIRKEINLVHYNKFSFYEYLFLLLFYKKGIH
KIFDYVYVYYYKTNIFDSLYNYNNEYVKNNLLFAFAKDIKKYFLIFNSNIKKLDVQLA
ILIKKKRRKKKKRIYEQKHLASYSSKLERTTNGNKYINTYDGNEEENIFVHEEINTAY
GNYEGKPVLNSKYCLIKKIKNILKKKTLRKSNINKLDVIHLPIDDESVENKNNVELNN
YVCKSCIIDIEEKSLYALKIREEEKEKDSNNISLYKEKSRHIQIYYDYEDMQEYIMKK
KKKNEKNEKNEKKKKKKIMMRNFNKSISGTKPIINTPYYEPLKNNYQMINFTKRLFLS
NPMKIQEKGFELNRYYPRRVNYPMKEKLGTGSYGEIWYAININKNSQYKDVVLKKFLI
TKDEETSELNAMREVYFGEILKNCDNISRFIEYFKEYEINETVNKKKEEYIYFWLVFV
NEGYSLSKHLFDTSSSTLGLVTPSALWWSIKKQNIGMLVLKDLLRQILNGIYIAHKKG
ITHRDIKMENIFVSSTTPFTVRIGDWGSAVEYKNEHFSFIPSKDEETNGYQPPESLFG
HMKNNFMRLPYYDMWSIGIVFLQFILGTKNPLEVKDKESERRLKKIFSKYPIDILKEA
IFLQSLSELCLTPWVKKSTHNLIRLQKKKKNKIIHECDRYDITINDVHDVSHNNNINP
YNRPNHIYRKHFFSTKSSYNSLYTNNNNNFIMNKLKYYITNDLINIKKQSFHNIYNII
SDKYKSLISLPSSPLCSDGMCLHKYDQAYDKKNITQPNDTGLICLNKYKLFFSENQKN
ILPNYTCDDEKFQNILKDRDPSGVGLPNKNARDLLRQLLNFDYESRITAEQALNHPWF
QEN
>MAL13P1.209(Q8IDS5_PLAF7)
MYGIALKNVGRIKKHGRKHLVSKNPYLRLLVKLYNFLARRTNANFNKIIAKRLIMPKR
YRPPLSLSKLQYHMANHPNDIAVVVGSITDDKRLFSLKQLKVCALRFTETARKRIEDA
GGECLTFDQLALKYPTGKKCVLLRGPTKARTAEKHFGKAPGKPKSKARPYVRSKGRKF
EKARGRRKSRAYKK
>MAL13P1.22(Q8IES4_PLAF7)
MNFLILVVLIKMVVCKIINYVRSDYNFMLSFLRTRKIFSHKKIYFNFKVSKNITKNSS
SNSYSDILKCFSFPRKGTNDKTKENETFEKGEVKEEEEEKDKNVKRKIINESSKNKRA
KVKNENDVIKKGSLFNCVVREDEKVNDLSSPKFNPVHFNINDLYLSEKDKEKHKFKDS
LLFTFLTNAFNQIEELKGSGTGSKKNVSIILSNIFRVLIYYSPNDLIPAVYITLNKVA
PDYLNVEAGVGEALILKTMSEAYSRTESSIKKDLQQIEDLGIIAENCSCKMRTIFPLP
RLTIQSVFHELKSIPNLIGSNSQQKKREVIKKLLVSAKTSEAKYIVRFLQQRLRIGVN
SATVVQALSYAFILTRPSIPDDIIKRGKEINENLINGKLDGTNNLINKSNNKTEEWLT
QSGDRNCIKEENLNDELLSKDDINKCENMDSLINEIRIRNEKINKPNIFYNLIEKVGD
TRLLPLFFKELKNIYCEENNDIDIFECMEKSVKSALCELPNIEIIIQNLLNGDDMNTL
SKKCTVKTGIPVQPMLAKPTKGVQEVLDRFNNVTFTCEYKYDGERAQIHYIDKDNIKI
FSRNLETMTEKYPDVIQIIKDQIGENVKECIIDSEVVAYDIVNNKILPFQVLTTRKRK
DVDIENIKVKVCLFPFDLICCNGIPVIKEPLAVRRKLLYSLLKSKDGVLSYATHSEMN
NIEDIDMFLQDAIENNCEGLMVKTLVENASYEPSRRSLNWLKVKKDYVEGLSDSVDLV
PIAGYYGKGKRSGVYGAFVLAAYNSETENFQTVCKAGTGFSDEILSTLYETLSEKIIP
NKKSYYEVSDKLNPDVWFDAHYVWEVKAADLSLSPVHTAAIGIYADDKGIGLRFPRFL
RLREDKNAEQATTTQQIVDFYEAQFSSNKNKNIDYNDDTESE

>MAL13P1.220 (Q8IDQ0_PLAF7)
MHFGIPSLFYLYILFSIIMRIKCVITKNLKKTKKRTCSYIPHGNMEKGIILNYIEKPN
PAYLKRGKNKNKNKNKKGDIYKLRNVEILLYANRYVHEGNENFSSTTKKLLLTPKVGN
KMPEGKKPDWFHVAAPTVAKYNKLKDDIKKLNLHTVCEEAQCPNIGECWNIGTATIML
LGDTCTRGCKFCSIKTSSNPLPPDINEPFNTAKAICEWNIDYVVLTSVDRDDLPDGGA
SHFAKTVELVKFSRPDILIECLVSDFQGNIDSVRKLAFSGLDVYAHNIETVKRLQKYV
RDKRANYDQSLFVLKTAKEINPQLYTKTSIMLGLGETKEEVIQTMYDARKNNIDVITF
GQYLRPTKNHLSIVQYISPQMFEYYKEEGLKMGFKYIASGPLVRSSYKAGEYFIKNLV
NQRNKDKKN
>MAL13P1.253(Q8IDJ7_PLAF7)
MATTNKKLQKIMTQPINQIFRFFTNKTVVQIWLYDKPDMRIEGIILGFDEYMNMVLDQ
TKEISVKKNTKKELGKILLKGDTITLIMEVKNEET
>MAL13P1.281(Q8IDD3_PLAF7)
MIFIYFFFVTLIIINLNTIESKYLFGYSYNGNPKCPINILYDKKYIYSFILNNNKRNT
YNSRIKCAKKSSIIPNEIVEGKVRLRFAPSPTGFLHVGGCRTFLYNYILSKQMNGSLI
LRLEDTDIKRNTKDSLDEIIKDLNWLNLTWDEGPDKVGEYGPYKQSEKIQLYKKIAHQ
FVNEGKAYFCFCSKEELQEIKEKSKMMKKKYIYNRKCRDMNNEQIKMKLEQNIAYTIR
FKSPLKRKIILKDILKGDIIDEVLEDFIILRSNELPTYNFSVSVDDYLMKITHVIRGV
EHISNTFKQILILETLNADIPHYAHIPVITTEEKKKISKRNNEYLIRNLREEGFKPEC
VVNYMTTLGWGSISKKEIYTMSELIDTFNIHKLNKSSVVFDIKKLKWMNKKYMLEQDN
ETYVREAEEYMINNNILSSNEYKEFVELCVDIFKYEVHNYSELKECILNALKYEHHVN
DPLNTDDIYLKQVSFLLYDWFRKNDVKDNTEYLEMKMIDDFDSLIDDIVKSTNLKKNQ
VLLKIRLLLTFQSKGIPFIYLIKLWASARKHNIPNYFSLKKRFLHLKNLFKF
>MAL13P1.301 (Q8IDA0_PLAF7)
MKTQTLSLMNINGKRKFLGTNNKIYRKVIINPTSEDDIQKFCRNYFRIYNFSLYNFIR
RLISFDAILVYSLFLTVYIFSEINHGETKKYLFIDTAISLFFNIILLIVIESLFELKK
LKDVKNANSQYYLRIVPKMSYFEKVMTKDIKVGNIIRIFQGDEFPADVVILYVKNNAN
AIVDSFKIDGLFRKSIKYAVDKYKIDKDYLKMLSEINGVIRCELPNKNIFCFQGNFKL
DKHPRSLLLNYENFALQSSVLKGAEYIDAVVVYTGADTKKNLNIPQKIEENKTFCIKM
NNIVYYLIFMYFVFVVLSIVIKTIFFHKKNSFQNSRDSFLSMLEDFVGLYILVLPIMM
YSEKSLIYIIQSLRIENDLRMRNTDSEKPKVFNKNKNDSLGNVDLLATSRNGVLVKRK
ELLVSCVINNVMYGKKDIICSRTNFKLPTLNILDSERKNVSNLLNLDERIFKDPENIF
FPTRDFYSFLKLFENKISSIYNPYSSSLSNLLKEKYKNYVNEEILNKNVKLTSFVKSQ
LTIGYNQICEDDELSYNCYEIKEDSQKENIQSVKIEDFILGLCGCNRIIIYNEKSLDI
SMNEYKSDNFMETYSKFETENEEYHENDHDEYHNMEHSDDENINSIEYEDICLYNIIR
NTGFSIYCYKNTLFLYNLMKECKVYFLTCYHDFLRSNKFSMCILKCGYSINNEKEGGI
LYVRGYDFNILPYISKEKNNIKKIKNVIKIYTLNYLKVIILCKKQISNEDIAKYIILK
SISKKLSFKFYDLIKLFFLYDLEVIGIIGLKNQLREGVKETFNDVINFDIKSWIFANE
CSKDTYLTALQCNLIVSSSNLFLINYYNLKNTHEEGANILFHNFISSLYKLKSNSYAV
VINDESIKNIMTNVESMKIFLCIAMRATVVLFCKLQNETKGKIIRTLYALTSPKLTVL
GIGTTLNDAYLLKYSSISVFLSLNEHVNILYNISDYVLQEFKFISELLILGRLNRFSL
CKVFLWIIYLKITVVSFYFFHNFDNYFSGSSASSILYTQTTFALLHYFLIIAFSAYEI
DLPYKFVRRLPYIYQLSRRKYFLNNNIILLTIIEAILISLTSYYILRLNVFHLITHRE
FTFHIFILNVFITTEKILLLSKTWHIYFFIMAVLIIGILLIYVNIFTLVDCIKNGKCE
FSLFQMENIYFWTSLFPILYINFIFDKLMKYIKNRIYPDISDYLRKYFLRRICCHNND
KFLSQRKIKGINKFVNFEKNDILLKYIPTPKIYKIKDDPTYYNKSKRSKFLYDTFRKV
IDINVKYRNQQLNLEYKTYEKGNKLKLRIIVILLFLIYIIIFSSQTIIDINTKSNIHY
ITMFYIIYFVLACVLLIYIRIRNKATSTFFFFLSRFLLICGFCIELYDNISNDILNVL
ITYSFTVSYIFFMSFKILEALLVCISILLLTFGVYYEKNKNMIDICTHFCSNPYLSIN
NLDHMNISCLCKKQIVIFLISLLSFTLICLSMKYYEIFYLKKKFLFRYKQKVNLAKQI
EILHTMLPNFLVEYLLISDPKNDGIMVGKNISGEDRGIISVIFCDIDDFQNMVSTLQP

HVLVETLDNLYLYFDKCIKYFNCIKIETVFESYLAASGLSEKKNNALDKIMYDTKCAI
KLAIAQLSAKYYISYKVLDTREHFSDNSTSYDKYINKNISLKIGIHTGKAISGVIGSV
KPQYALFGDTVNTASRMKSTSLPDHIHVSYDTYKYLKEDNTFIWKERKVFIKGKGKMK
TYLLVDILDDVKRKGESLNYYSSSNLLLSQLGSEAVSIYEEREDIKEGSMDIIKESSR
DIIKEDSRDIIKEISTNISKSSSRNISKSSSRSISDIKEGQIIDKEDLIFKINRMKNK
IDSRYSKRIDKESRDKISDKTNHVLDEVVKHSDIHLLNYEINNKRCKKMKGDTNNENK
LIGDIFNMYDKKIKYIYKKNYKSKSMENISFIKHYRNTKYKKSDYLLLDNKGESKKFK
RNTSYVLESPLHLIGDIVDNNIKRKKKKKEIKTIVSDDMFTSPVNIKEYNYNEQERKK
EIVGNLSYDKTKKIFPFIKFTKEGRIKKKKIEKKEKKEKKENNNNFLYNDDYSSYSSP
KYGDNENNFVIKYIRERKDFQKKFDHPNFNFSKFLHNYNPMKNKNKNKKNNKNVRRNE
YPNYTSSSKDGVSYNFLSDSLFSSDNEYSSDNEYSSDSEKYYKKRFKKNKKIIKFDDL
FTKIYIKKKRLLQMNNYDVKGKGKKLKNKGMERNKTKYKNVNEITKMKYFVNNENRDH
EVNKEDISKSMQKYFLHISKHKKEQIEDKKKTHKYFHKNVECVYPYAGNNINHNFSRN
EKRKYSINLYDHLDEQEKIKGKKKYFNKDKELIGSINKQTERKPKKKNKKNIENKKDK
KKIRMITNKTKEKHSNSIISVEEQNMNHNNSLKKKEVNFTGKNEEYLNRANTNCSLGI
KEMEEDVYEFHSNNIYYNNQTSYSDDINNTTKLKGMGNNTNDISKNKGKNKLGKKISF
FSMNNKYHESEIMNEEDNKNMLNLTQSQIINKDKYNYFTHCPSLKKKKSVFTKINNLF
KNYFKSIDVHEKFGFSKKFKFHSKDSDDIKGNNNKISKNRYNNNNNNNNSNYSNIDSG
KYSHNNKKNHHHNNNKYHHHNNNKYHHHNNNKYHHQNNNYEKHHHSNNSRVMLSKGEK
TEKNENVDYAYQFDNYDKKLLKKLTSNLQLNKKNVKNFNMFYYKFNDEELEEEYTRNY
YREIINIDLTKKLIIIFIFTEIFLSLCNIIELSFYEKKLRYNDSIVIIWLIRSIYLFI
ITYIWIILKTKLKEYKNNSSKMMWTIFILNIFLCSWGIILIDLSCIHYSMLLGNKNER
ALFFMKDASELIICIQLIFIKNMLFKHKFFFFVFFYIFLIYSFSKLFSIHTCQTHICC
SIILFISINILYFWYSEYLDRIQFLVKRKRNRMEKISQDFLTKILPRQVLEEYQNDNL
QLTYKHEKIAFLFADIVGFTKWSKTVSPKEVLKLLQKLISKIDKDTIKLGLYKLFTIG
DAYVATSQPNSSITDESEALEGILNILKLAKLILHNINTIKIQFNKHDFNMRIGLHYG
SCVGGIIGSVRIRYDMWGLDVLIANKIESNGIPGEIICSEQFRHFFIQNEPQA
>MAL13P1.322(Q8ID64_PLAF7)
MIAKSYVYRDSSSDGNKKNNDKIQTGFLYNEKKNEKTLEFKRRKVNKINKKLTTDDVK
SNIFKKIDTFEEASFKKDKVSKRYLNNNEKEKYYKDRDKNKIKKNNIKDKHVNQKYNI
YNSSTSPDNSDIDNLYNHKENKNNHKYNKSDYSEDSINISINIKNDESNKYNLSNKQH
HNRYSNKTFEKDEKQKKDYYKKNNDKILDEIWYTKEDEFVDSFYNMDIEATYEKEKKM
INNLKTRINSNTGKKVNQKNLDNNLWELNKLKQGGVTSTYNKLQLEKINEANVTNEIK
KIVLTRTVNPPFIDKFKSYNKRFVNIEQRNNFDDQKSEKKKSDNINNDKYYSNKPIQS
TSYSTVVKDETCDFVKAAKKGSEFLKYFKSENEKSKARDRYWEISNSKLGELLKLYKN
KKKNKNDNTSKEDYDNISYNSSENNKEDDNGSDVFDYKKDKIYSSLFNIENNNKDKKN
TLKDKEELLKLKESLPIYKSKHELLDAVYNNNIIIIVGETGSGKTTQIVQYLYEEGYH
RNGIICCTQPRRVAAVSVAYRVSYEMNVDIGSLVGYTIRFEDNTTKDTKIRYVTDGIL
LRETLNDKELDKYSVIIMDEAHERSINTDVLLGILKNICLKRNDLKLIVTSATIDAKK
FSAFFGNAPIYNIQGRTFKVHIEYLRTPCNDYIECAVQKAIQIHVSDNNYDNNFGDIL
IFMTGQEDINATCYLLSERFYEVYESYKESKNNKKDTINKIKNILNEDNNNNNNDSNI
KKKVDGDNNTNDHMIYPFYIFPIYSQLSSEQQSKIFKKYDLRKIIVSTNIAETSLTLD
GIKYVIDTGYCKLKVYNQTIGMDVLQVTPISQANANQRSGRAGRTGAGICYRLYTENT
FLCDLYQNNIPEIQRSNLSNVVLLLKSLHVQNLFEFDFIDVPSKESIINSLHELWVLG
AINNEGNLTDIGRKMVQFPLDPPLSKIVIYSQNFQCTKEILIIVSMLSSPSIFLESKE
NNESIESKKEKFTVPESDHLTLLNIYLQWRSHNYSYSWCTKNFIQYKALNKAKEVYSQ
LIDIIKTLNIKNVSCDNKWELIRKTICSGYFHNAAKLKSFSEYINLRTNVSCHVHPNS
SLYNIGYTPDYVIYQEIVFTTKEYMRNVTTVDPEWLCELGPLFFYMKNV
>MAL13P1.324(Q8ID61_PLAF7)
MPRLVIFYFLIFVFMKINCINYNSFSKRKINYHLIRDKQENIKYKYLREDEKLNLSRR
NRTKRTKETNGGTQRIKRIKRIKRIKRIKRNNLSNIFYNKNDYMIKKINVPRTYFIKN

MGNSSLKGCIYKEKKTIFNIYNIYDDKNIMNPKNFGNEDGNLLNKLGNDYSDKDSYNK
NMNDKKQIVLDNKNEKMYIENMKKNLSRTDEVTYKYVEEMELKKINIKGVDIQYRLYN
LEEGIYIVDKENYEKIKKLWDKDELKKAKENFEDTFDIKKQGIKDEDWIMLPFEYEEN
KNIKLVKADFDNPTYDYILTYYDKERNYYWEYKRRNYYKMFKNTVHETPPYKSDLEED
KRYYGKEIIIPKKKLDNDIFRQPMLSDVMTSGCNREPLGFESWRFVKYPYGNLIESQK
YSKLYCVKKNDKDKPSMKYHYLGNSNLAVSEICLGTMNFGNYVNEKLAHELFDYAFEE
FQVNFFDTAEIYPLPASENYYGHSEEILGNWLEAKGKANRHKFVIATKICGRTDKLPW
MKKYKIRTEQKNILNKRNADIYNDNNYKKDHYITNSKSNEYSHRNNNHMLDKEDNKNG
YDKLKELKEKEELYLKKDYDKINKFEEYEKERLDNSANLITLNKENIINSVDNCLKRL
KTSYIDLLQLHWPDRYYPDQSSGDFSHVLYDYNKYYDDFIPFIEQLQALDELKKKGKI
REWGLSNETPFGLLKFYELCKHLHISPPVSVQLEYNLLCRNDVEKGFPEICRPQNTNI
SILAYSPLCAGILTGKYLEYTDYTTKGRMQKFPSYMKRLRGSIATYIIRELYYLSQKY
YFPNLTVAALKWVYTRSFITSTIIGVSDFLQLRENLYSLTNEVLFTDKLEREINALHW
KFRDPIRIIQ
>MAL13P1.335(Q8ID40_PLAF7)
MSLFNLITLYYVMQKYAKTPKISILTKILNYLILIYYVIIMFLHFFSTSEVRTILRFL
NKNIEFHAVEKSYMENCNNLANISVRILKRKIEHSKNIYIYICIYDKIDWFVCAHLWG
WFAKGMIIRNFFLLNINSVIFELIELRFQHILPNFYECWWDHIFLDVLSCNLIGIVAS
ILFMKYFNIELYDWKIPDKIKPNKKNIIFPTIDKLCRKVFTNSSTLLILIFLSFITNI
IDLNVFFLKAEIQLHHVNLIVIARTFAIGFISGKACKEFYRFLKEGMTPKRAFYIFLE
IIILSLEFLLAIRWKDTLISDKSDLTGINMVWLFITSTLSSILLLLYVNESLI
>MAL13P1.338 (Q8ID37_PLAF7)
MSAIGMPQHILILFQARPMLEFYKPIKKKKPKEYSGLSDFLNYFEEGEAPPKIKVESF
KERKEKKKKEKMAYNELILKEKRKEYDPFKNEDLTSDPKKTLFIGRLSYEVSEQKLKK
EFESYGKIKTVKIIYDKNLKPRGYAFIEFEHTKSMNDAYKLADGKKIENRRILVDIER
GRTIKNWIPRRLGGGKGPARGSEEKKKIIHNINWTALINKDKYRNDKKRSDELYKNVP
LYNERNNDDDEDDGISSTMRDYKHHRSDDHRRGSKRDRRHRSRRSESSDRHHHKHRRR
DRSRDDRDRNYYDRHGRHDKHDRHDKYDRNDKYDRHDKYDRHDKYDKHDRYDRHDKYD
RHNRHDKYDRHDRHDKYDRHDKHHNSDNPYDNSTKDDIRKENEINDIQHNDYENNREH
NEGFVYESITSIEDCDD
>MAL13P1.35(Q8IEP6_PLAF7)
MSNNTTTHNINSINNVNNNDNINNDALNNPNDYLDKNTNFTNAHFKPSSYVNGAGKNM
DSAGNIIPNINNNIQYHMPIPNNMPYGVNPNYNIHNNKMINNPKNMYNAPYPLNMNTI
PINSYGGAEKFTHSVRIPIYPTPQNMLYNTMNYMNSKAYIKHLKYNKVIPTDPTIPPN
ETLYIKNLNDRVKTDEMKKNLKDLFNTYGEIKDLIVMKSFWRKGQAWVVYDDKECATK
ALNALQGYVLFGKIMQINFSHNKSDIHAKRDGTFVERSKEPKKPKKILEREQKQKEIF
EQMHKNYLEMQKNNFNMLNGNKEINKPEIIDLSQMDKQTLIAKAQAKANEDKNKKNEE
LPNNNIFSSYYQMNNIAPVQNYPVVMPYKILFVENVVENVDTQAFNDLFKNYAGFVEA
RIIPQRNVAFVDFTDETTATFAMKAVQNYELQGSKLKISYAKRYFFI
>MAL13P1.47(Q8IEL5_PLAF7)
MRYAFGTSYLTCFMGINKAMKKFVFKKNKYSSLHLFERNEINNFQINIMDRKYVHSIK
EEKKKEESEDYYLSMGDNIEKRYSLALYNVAKKQNKINEISNDMLFIKNHLLKDTTFQ
KFLHTPNIEKKEKIHFIKNECKTLNKFNIITENFIESLFDSKRISFLPKIIEEFEFLL
LKQRKEIKCVVYTANEIDNNYKQKIQDSILIKLNKKLIPIIQYKTDPYILGGLILQIG
NQVYDFSAKSKIDKIKTNFSQ
>MAL13P1.56(Q8IEK1_PLAF7)
MKLTKGCAYKYIIFTVLILANILYDNKKRCMIKKNLRISSCGIISRLLKSNSNYNSFN
KNYNFTSAISELQFSNFWNLDILQKDIFSNIHNNKNKPQSYIIHKRLMSEKGDNNNNN
HQNNNGNDNKKRLGSVVNNEENTCSDKRMKPFEEGHGITQVDKMNNNSDHLQQNGVMN
LNSNNVENNNNNNNSVVVKKNEPKIHYRKDYKPSGFIINNVTLNINIHDNETIVRSVLD
MDISKHNVGEDLVFDGVGLKINEISINNKKLVEGEEYTYDNEFLTIFSKFVPKSKFAF

SSEVIIHPETNYALTGLYKSKNIIVSQCEATGFRRITFFIDRPDMMAKYDVTVTADKE
KYPVLLSNGDKVNEFEIPGGRHGARFNDPHLKPCYLFAVVAGDLKHLSATYITKYTKK
KVELYVFSEEKYVSKLQWALECLKKSMAFDEDYFGLEYDLSRLNLVAVSDFNVGAMEN
KGLNIFNANSLLASKKNSIDFSYARILTVVGHEYFHNYTGNRVTLRDWFQLTLKEGLT
VHRENLFSEEMTKTVTTRLSHVDLLRSVQFLEDSSPLSHPIRPESYVSMENFYTTTVY
DKGSEVMRMYLTILGEEYYKKGFDIYIKKNDGNTATCEDFNYAMEQAYKMKKADNSAN
LNQYLLWFSQSGTPHVSFKYNYDAEKKQYSIHVNQYTKPDENQKEKKPLFIPISVGLI
NPENGKEMISQTTLELTKESDTFVFNNIAVKPIPSLFRGFSAPVYIEDNLTDEERILL
LKYDSDAFVRYNSCTNIYMKQILMNYNEFLKAKNEKLESFNLTPVNAQFIDAIKYLLE
DPHADAGFKSYIVSLPQDRYIINFVSNLDTDVLADTKEYIYKQIGDKLNDVYYKMFKS
LEAKADDLTYFNDESHVDFDQMNMRTLRNTLLSLLSKAQYPNILNEIIEHSKSPYPSN
WLTSLSVSAYFDKYFELYDKTYKLSKDDELLLQEWLKTVSRSDRKDIYEILKKLENEV
LKDSKNPNDIRAVYLPFTNNLRRFHDISGKGYKLIAEVITKTDKFNPMVATQLCEPFK
LWNKLDTKRQELMLNEMNTMLQEPNISNNLKEYLLRLTNKL
>MAL13P1.67(Q8IEI1_PLAF7)
MYVKCFFYVIQILFIIFLKCHCYKIKCFNILDLNKKKYYSFREHINCEHIRNSVNRNN
LSNVLLRRRTKNALVKELYVSKLKDNYKTHTNFIRTNNIFLEEDKKIQECNINNIINN
NVDIQENVEKYNILYKNQLDDINILYILLFNTLMIYKKKYDFFMNENYIRSYYYIYKN
NLGRDKKIYNTKNYFINTFSITWYNTIKPYMNNIFLEILNIIENTLGNKIFYIDIKQE
LNKNRDEIINTLYNIYKGNFRKRDKKPIKLLFIGSNEYSNLCFKIILLIIKRLRNDII
LDNVITKSPRRKGRNLILKKSNVEDEAIKNNINVFYYDKLKNNIHMLQNKKMDLCISI
SFGEIFNCNFFKTIKSNIFSLHPSLLPFYKGASPIQRSLLNNEILYGYSVFLTTLNID
SGNVIMKKPFWFNSNYNFNDIITILFTQGTLSLLKNISYLANYNKDIPHKNIYNNNIC
EETKNNLNQNHVQNKYDSEINIHNSNLENKNNSRNNILPLNINNVLNNYNNKMYIQND
YNINNNYAPKIKNDEKYVCFFCSTSLFIHNKIRSFINWPKAECTLFLLQNEVIKPLEI
KIIKSSYDLNNNYKFIKYDGLINTHDQHTCFDNIPRNFVYIQNDSLNILCKNNTLLKI
YKLQQKNKKIVDAMSFINSINKCSLLY
>MAL13P1.83(Q8IEF5_PLAF7)
MDELEVAILCLYGNEHSNINKNDAQKYCENFQNSADCWKYCMSKFLESNKLEVKFFCI
HVIVEKISTLKIEDMILIKNSLYGYIEKKYVNANEDSCVLNKIIQLYLYLIEFLYPHN
MNDAFKYLINLIMLNNDINIKTIHINFFLKLMNMFDSEYIDNVCSNKSIQTTTNIKEA
IKENDLPIIIECFYYIMNMNIPESTSLSIFTLSKYVPWIDINYVVNDKILTYIYQTLN
TTNSITEASYSFLTSLIRKGMNSANKIQFIESINIICILQNTPKITDLTFDVNKNIMT
KRGELINYICLELVESIFEINKLKDYQMNMLKYNEICTKAADMLFLVLPHALDIFSVN
DFYIASTVEKFFSLFFTKFKNVIDVGSCANSIMKSSSNDYESSKNNISNNINSSINSN
INNNNANSGYKISIDKLNVFINTLICTIVNKFEYPECIPDDYDEEEEDDDEFSTFFNF
RENIEKLYQRLILFDKLKAIEIIKNAIIYLNENYDNLKWNNIESKLYAFYVTTSIYCE
YKQGSTNTSNNLNNNMITNNQNKNNIIMNNSLEHFKNIKEANEVNIDYNNLLFDCLIE
LLKNRKILNSTNYHININLMEIFQRLNLFFIKNPNYIEYALHIFLTNGIRSNNNKIAK
KSVHIFKKFLKTNSSVISNYIKDILQLLESYLDVPYIYPKMDINNNILNNNNMILDDN
TIKYIYTFLYSNKNYNHEYQIDIYEIIGLLLLNYDFNKFKKLSKTDSGMSIQSNNNLS
NNLNLNNNDNSININNNNNNNTINSSNNNSNDGLNQNISEEMIANYKDRIFFFKGILN
KLLENLSAVKNLYLNSTKTQHDNICASFISSVIIKCIGALCKNVNINITDILLNDLDN
TLGIIISESLELFYNNYIVRDSVLFTYRILSNLFKDLSLNYTVKILPYFYNISYNMIM
NKLQKSSVDNQNANTFNMITPTSYQSVDGTTNNPMKTSNTHHEELKYLYNELNELSIL
VCHLISTHKEKSFDTFVNPYIYNITQIHMNIWKFINVQSLEMQREQNAVLSPLLLILY
NISVNIPATIHSFMSFEHIALNHKQFCDIFSNDDIIKSKIADAITSILLISLNYKNTS
DINICLYSAQTLSNMLNNATCMTNPSEVLSKYPIMQIIDTLCVTLKSLDYADPKTKRI
MQEVMNIFRLFCGFKVSANCLPNKIIESSQICLQNSLLSVFKNNPNDIAILIQAINAN
NQQQFRQILSNIVA
>MAL13P1.84(Q8IEF4_PLAF7)

MNKMKSTKIENPKYKLMKLIGKGTFGKVYSAIDMSTQEAVAIKRSPKWRNKVSREVDL
LKKMNGSTNIVKIKSVFYTTTKKGYRIQNIVFKYMTYSLGRYIRMKKQEKRENNQICI
GIKDLHKNDFAHRDLKPDNILIDLDSSNIKIEICDLGSAKKVQRNIISIPYICSRWYR
APELLCGSMFYTTEVDLWSLGCIIFELINLCPLFPGKFKKDEYSEECSQIINLIEVIG
SPQMSFFENIKDHTSKKNTLLIKELCELNIKPLCWDEILGNILEIENPNERLNIDAVL
SNPYFSTLNK
>MAL13P1.95(Q8IED5_PLAF7)
MNIVILLLILTFSIKHSNTYKLKNTYIPINYMYHNNKNILRSQKSKLFLNFLSNNQLA
NSNKQTCFFKSNIKSSISNIDNYDYIRKRYINTSNKNKLFYNITLRTNDGEKKIECNE
DEYILDASERQNVELPYSCRGGSCSTCAAKLVEGEVDNDDQSYLDEEQIKKKYILLCT
CYPKSDCVIETHKEDELHDM
>MAL7P1.113(Q8IBN8_PLAF7)
MNKHDQKKKRNKQHTDNVMGKKSKGFIKNKKNIGESGNEKKRNNNFNNIWKKKKRSGN
SEKDQVDILGLLKGDSENMNDDDDNNMNDDYNNNNIKGDYNNNNIKDDDVDDDDYDDD
DDDNFDENKNCNDNCSSKHKRNVPSKKEHDILELNNINFNETRKKMINYKNIFDGKFC
DLKYILSESLINTLEKNEFIKMTSIQKMSIPLFFKPNDIFLKSMTGSGKTLCYAIPSI
EKILNMKEKVKITRDMGIFVLVLSPTRELAIQINNLFCILTKPYPYIVASCITGGEKK
KSEKNRLKKGISILTCTPGRLLDHLENTKSLKLTFLKMVILDEADKIIYLGTQDKIKL
IYDMIRKIKQEEFSKVHKKKKKEENEVLDHINDTNMSDMNNISNDHSNDYEQFILDKF
QMIFISATLNHAMKTLANYCLTNNTMWIEKEKKNGINGGNKNDETKQKSNDMISCMNR
ENSPLNIHNNDDNDDNDDNDENNGDNNNNNDDNNNNNDDNNNKNNDDDNNNTYELPEQ
LKQYCILIDMKQKFICLIYMLLDCIEKKKKPVVFLSNHHSVEYLQILLKNIYWPTDVN
KKNIEVNKKLNEKITPVLEREDEKLLRKHLEQNILNNNYYNNNYNVGNISYKNINLEE
IQNEDELNDEPGNLYNINADKHKRIYLFNNVNIYILHGNLSKEDRLGNFMDFSKTNNS
ILLCTDIISRGIHFDSLSVVIQYDPPQILEEYIHKVGRTARLNKQGSAYLFLLKSQKQ
FLNILKNKNIQLKIILGNTIINHFKKFCIPNFLKSVGKDILNFLHNHMQTIVKSNNTL
MEKGTSAFLCTITSFYSTSKNLRSIFNAKDIHLGHLAYTFLLEKTPKQISKYKKEQNY
INIKKQTVLSKKEKRLLKSKQFQKKQKRK
>MAL7P1.118(Q8IBM9_PLAF7)
MKLLYRKRDNDKMIIGLITEEDDDLWGVYNLLSLNDEIESYTSRKVQKDIGNNSYVTE
IRKLMLTLCITKIDFDCENNSLRVSGKNVKANEYVKIGQYHTFDIGLNDKIKIMKKNW
DHIHREKLEECTNIKNNCEIAILLIDCGRANMYLLTQQLYKTVFSINKIIHKKKDKNN
SSSYKKSLENFFNIVLKNLYSSINFEKIKCIVLGGPGFFKNDFFSYLYEKSDMKNDKN
ILTLKNKFLIVKTSNIFKNSLNEILNDENMKKQILNLKVVSHVDILNKFYKIFEKNED
KICYGPDEVKYASKINAIDSLLITDKTFRSCDVKTRKEYVQVVQYVKNTGGQVYIFSD
NHTSGEQLNSLTGIAAILKFPIFYDINQAHEKGQSTKEDYIKREDTQNGEHMTNI
>MAL7P1.122(Q8IBM9_PLAF7)
MAPKKKEEEPPKLLLGRPKNTLKMGLVGLPNVGKSTTFNVLTKLNIPAENYPFCTIDP
HEAKVTVEDERFEWLVKHFNPKSNVHAYLSIFDIAGLVKNAHLGEGLGNNFLSNIAAV
DGIYHVVRAFENEDIIHTEGNINPVRDLEIINSELIYKDISHCEKNLEEVTKVLNRNK
KDKVKQNEHDVLTSVLNYLKEHKWIKDGTWKSNEIEVLNEYNFLTAKPVVYLVNMSEA
DFIRQKNKYLAKIYNWVQEKNKGTIIPYSAEVEQKILSMDEEEKKQYFETNNIKQSML
NKIIKTGYYEINLIHFFTCGHDEVKCWTIRKGTKAPQAAGVIHTDFEKGFICAEVYKY
TDLVEYKSEGEVKANGKYLQKGKDYVVEDGDIIFFKFNVSSGGKK
>MAL7P1.130(Q8IBK9_PLAF7)
MKRSFRSIKTLRVNYFMKNKRIYHSSSNNTYDEKEKRFFNEQDNEWWYDDDFDNENNT
QENKTCIKTRWKNIFDEIIGKNIYSLHDYNKKRFDFIFKNYEFLYYKNIKDHMNKKEI
NILDIGCGGGILCEYIKKNIFYFLLKNVDNVDLIKDIQINIDGIDVSEKLINVAIKRQ
QINRDTYKHLHINLNYMNCDLNEYVNIHNNNKLKKKYDIIISSEVIEHVPNNKKNIFV
SYINKLCTKNTLVVFTTINKNYLAYLYTIMLGEKIFRMMKKGTHDYDKFIDNKQLDKL
CRDYNLYNIKTEHVLYLPFFRNYFQTYKLNLLYLSSFIYSGNNL

>MAL7P1.150(Q8IBI5_PLAF7)
MKFLQIIKHLKLQNKKNALDNFVNCRTYEHISNINKLFLNNFSSTKEHSEHGQVKHEN
FLNSTLKYEENSQNGSTNNLKNGKYNMYVSEGNVNINEEKYKDNNISSNNTQYNNNSS
NSGSLNDEGPLWKEHIDDVVNENKKKKMNRFYLDSQATTMIDPRVLDKMLPYMTYIYG
NAHSRNHFFGWESEKAVEDARTNLLNLINGKNNKEIIFTSGATESNNLALIGICTYYN
KLNKQKNHIITSQIEHKCILQTCRFLQTKGFEVTYLKPDTNGLVKLDDIKNSIKDNTI
MASFIFVNNEIGVIQDIENIGNLCKEKNILFHTDASQAAGKVPIDVQKMNIDLMSMSG
HKLYGPKGIGALYIKRKKPNIRLNALIHGGGQERGLRSGTLPTHLIVGFGEAAKVCSL
EMNRDEKKVRYFFNYVKDYLTKHLDYIVFNGCQINRYYGNMNISFLFVEGESLLMSLN
EIALSSGSACTSSTLEPSYVLRSIGISEDIAHTSIRIGFNRFTTFFEVQQLCINLVKS
VERLRSISPLYEMELEKKNPSNDDIPKFIWT
>MAL7P1.151 (Q8IBI4_PLAF7)
MHKIKVLELYCGIGGLHYSLLQAFNNFVHANKITEKKCDTYKDGIHNHMSNNKSIEIH
KYHDCTLTCLNDLFCFISVDLNPVANQTYFHNFKDSTILLTQTRDVHKFFKWKCDSMD
KQSNGSTHNEHEKTQKKKNKKKNNDDDEKNNIFNINKNYIIQTDINNIMPEFLNNHHF
NILLISNPCQPYTRQNQKFKEINLDLLFCKNNEYKQNVNNNISNDNNSFYSNHNGDEN
HQFNVDNINIDELNNYIYNDKDERTKSFIHICTLLTKVDFKNLPEYIFIENVKNFELS
SSFIYFLYCIKNNYSFQTYLLSPLQFGIPNERLRFYCICKKKNYDFKHANNLSGINYI
KDKNLNLYTNSLIPKNYLHKNNIHEQKNNQGDNYNNISCENVIFYTPNLITYLDVNNN
FNITNNIWNHINIYNNYLDNYQVQNQVLQKNASYCFDIININKKTTTCCHVANYYHHH
HQKKKENVNNISPDEPTKHMNAKGNYAMCFTSNYGRYIKGSGSILYYNRKENSNCAEQ
KTKNKETNVLTKENNEYVHTSNYTCNSISNSDNDTYETRRKKNCMKKYEQNVRYFTPT
EISRLMGFKMKTNNKNINQNEKGKNTYGNVFWNIDHINHTCAYFSNVHYCDVQNKNAC
LLTYQNVTNLNNNHTYQNCHQKNCLCHEFVFPNFLTDRQKYKLIGNSVNVIVISYIFH
VHNIFEHIHI
>MAL7P1.159(Q8IBG7_PLAF7)
MDRYINIDVRNMNNISDTDGSPNDFTSIDTHELFNNKKILLISLPGAFTPTCSTKMIP
GYEEEYDYFIKENNFDDIYCITNNDIYVLKSWFKSMDIKKIKYISDGNSSFTESMNML
VDKSNFFMGMRPWRFVAIVENNILVKMFQEKDKQHNIQTDPYDISTVNNVKEFLKNNQ
L
>MAL7P1.20 (Q8IC21_PLAF7)
MSIFVECRKNIRGLNKCLFLYKGVRNIRNRYNLCSYVNKDVENYSRYFKNSCSLILSS
GTGGTEACDFCNMLYNMYIKYLCRIKNRKNIKYEVIDISKNEVGIKKVEIKIDGEYSF
YNFVSEKGIHRLVRNSPFNAQNKKMTSFVKVDVIPTLNFNDLNVINFLNTAEGVGSSS
SSKLTKESSDKKQNKNKHKQLKESDNNNNNMNNSYNGGNNKMNDFVINKSDLVIQTMR
SGGKGGQNVNKVETAVRILHKPTNISVKASSERTQLLNKRNALKRIYEKLLYLQTEAL
KNKKYELANKSLTHFGEQIRNYVLYGNKMIKDTRTNVFSTDIDKVLYQGEIDIFIDAY
QKNL
>MAL7P1.21(Q8IC17_PLAF7)
MLKNFEVNSPKKLIRKIEEETYEEDKEEYLITNLTENKKNMPSVIIRIPRDLNKFLSN
EETTIKYNENIKSSNNKMNKEQKLTSFKMDNKYDMNSEEKISAMDNIVLDSTYQSDST
HESISFFNKIKGQNYTIDNINNENENNNSSSNGDSDDDSNDDSDNNSYDNSDYYTDDD
KPIDESLLKSFKNKYVDVYESNVSNNEYITFIKNHTKNELEHFLQRRKLHHTNEMKVN
DIINIDYDKLNEKNIIPYFLTNVFDMEQKKKREKMKLELEKKRKIMEEKRKTKTKVDD
KAKTKNDTHINEKKKKKRNENNKVDTKKSVENFTKAANTQKIVKEGKMKTEDNDNSYD
EYEDDNSYENKNRRNKKNKTNSNENQKINEDNEESYSDSYNDEFDKEDIDINIYNDPT
LYGIEGSSDYSEDMKSDADNHTQEDDTGKEQITNNNSKTPKKKKGKKNIDEKNSEEEE
KLDDIVDACTQRLISYDYYSSLNIKEIVKPNNKIKSLSSYIPIQENLDNLDHIQKLQY
LIKNLPYTHIKEKRSLYHYNIKQFIKWKVYLLNNINICLYGIGSKFHLLNLFTNICLN
DGNKCIILGFEDEINFEEILVRILEYHYKYKSSKTLKSFDLLYELIQRVNDSNVPLYF
IIHNLDNTKLYPYYEYFSFLSQYENIYFVCSIDDVSFELNMNFKNISSINFFYIKCHT

WLDYRHEILRQWNKFLPEWVFNKKCEEIDIKKNIETILNALSINHKRLFKIIASIQLE
NLDKGIYGVEKESLLQDKRIFTVGASSIRINSLLVEFVSHNVITETRLKEGNTFLKIN
ADKEELKRISEEL
>MAL7P1.75(Q8IBU5_PLAF7)
MWKAANVSYTRYASEMADILRKCLKDPYSDIALERSKMHIRETIYKDGKPVSQELYEE
FEKAYKNLSRQKE
>MAL7P1.92(Q8IBR8_PLAF7)
MRNSDFFFFFFVLLNCFLNIQPYVSNATSNIIKEKNTSFVKKIYDDPYIKKDVTLQLK
NAKYNHINKKISKSKKNVKKKRGISFVSLNLNKILELQDKNPIKLISNIYAKDKSKFL
STCYENNKLNDINNCNRFTISKQICDTPVGFNLNDNFLVKETGGLSSTCLVDNKPEYW
KIYNNHIIVESGEYFDLKIRNETFLLQTSIIITKDICMNKTKIESSYFTYQNNHSKIG
NPILYKGEVIGMEIKNIKVKDDVEGLFNVCSCYLDNEHHAYNKCSETNKHYMQISSIR
IIKKIVHNINILTGKKLHINLQNYVTPYPIKSAFIIKKVEHYSCNNINNKSFNEIQEH
FYNNEKKLKFYNIFLYETDLQEFKEDIIFENPGMYLLCYTSNDSQSEYSAELSTILVN
GYDISKMNYLYLDLYENKLNLRNSVVLHRYNLNEKLEEIFFKKKETIQCSGEDIIYSN
NITETKSYKSDDNSDDDVIIDTIYIYNINLNAYDQILEICSKKYNEYSLIGYTTIKPY
ILHNYKDINHLTHFNIPPDHVRTYVVDKKSNILTFFPQLLNIFNQNDINDMYMSFSCY
SSKNEIIITYSFDKNMTPTFFKYLKISDASSSILYITNKGVYLYVLKKKSQLLFLYDI
TPEKVKKKKKIITNKKINDDYLELLLSYYLDYYDFTKCEQCLSPILMEPIYDEKQNLK
HIFLITSHPLAKLLIVGLDFKIIYKHDNNDVKNVVTTIRGFNFTSDVLKYSSFLITGI
SCGILKNESLDCFLIDQLNNTIIAIEYLQKQKTIILIDSFQGENKNELNYESINLNDF
VFSQSNSYLHTPRNAIAYSFGESYVIFVNENESNELNLLFYDKNKADNNLSYITKINN
TYINDGAIENIYKFYDHKDLINRNVLLIMKYYEGDVYFMYIPLRNIANKLELAYDYPT
VIQDNGNTYTMKIKSEEIKKMNALVNFQIEVHNVNKSKYVTIDKYDGTIEIKLSEFVG
DSVNLTAKLHGFFLELNVNITFTVICSNGMKALNGICIPCPLGSYNNINEYIKNNNIY
ECTLCHNNSTTKNEGSTSISQCLCLPGYELNNNDLCVPCKRGTWKTKLSNSPCIFHCY
PNSYSLVQGSRSEEESKCKCKRGFYFVSKDSINFCDNCNIGYFCPGGYKIGEKKCPKN
TTNITQNNFSISSCKCDVGFEPFDSSNLNAYNFKNDPIFNDYKDFLDDVKSSQICVPC
KEGFYKNTVSEEKCKRCSDNVYTDGLQSTSISNCKKCEKGYYLQSEDSCIICPDNHYC
PGAYINDPKYAIYENQKIPCGDKSLTIPPNNLNVSHLNCLCKKGFEFIKTDDNEFDCL
EVPKNYYKSHLGNKEKEPCPENSVTLYTQTKSKVKCLCMPGYYWDLKEFKCIKCPKGH
YCPGGYLKNCFNNQNLHSCKPQKKKCPLKNSTTQTKESFSQSSCLCDKGYTINKEELR
ECIPCPINTYKDVISNAECTKCLTPYTTDGQIGSTKEEDCTCSGGFFFLNHCLPCSDK
NTYCKGGKMIVNNKNKTIHYGPSKCPPNTTVSFETERPYNKGFCVCQKGFKHVYTTSD
FTKICAPCERGFFKTIIGDFSCESKCKPNSTSFVGTIHETHCFCLENYYFKNGICLNC
PDGAYCEGGFQKETLLYMKKNEYYLDTSKIKHIMPVPKENYALYKLKTNIYNTDWFIV
ECPIKEACLYNEKCHESMTNFLCGECKKGYTNNFSKLNLCIKCSGHIMNILHMIFVSI
FILLFTVIMAYLNVFTGANRKSVHSIVIKIAVNYFSCMKIFYVMGISELYFPVTFSSH
VNYILKNIKRLLKAKKNYGLYCILTNYFDLSHADAYFYGMVYHAFRPVFLAIILTLLM
FIVVEIYKYKVRNETNIKLNITDKIKELGKDKLHEEIMNELPSERALVLFRYIPIPGD
SRFKRIKNFLEDMIPMYVTLLFFIHTKTTYYMLTLLDCKALYYNDKFVEQYMSYVPSI
KCDLSKSYSKFFILGLTGLIVWGIGIPLMSYLVLYKNRKQLHSENILFKYGFLNNGFN
FQFWYWESIVFLRKILVLLISTVPIFKNAKIFGTTMWLFTIISSIFLTLQVILQPFDS
RNYHILNKLETYSMVAWTMTLIIFVFLTISNTNVTINFYVLLFLLFFNFVFIAKILIS
LCYSYIENLRHMKKRIKLPFLRIFFEKMSKIAEEKDYKDPVVSLNTHDNSIQFTRKYK
KKYISCSFKNNMLTTEEKNYFLDVISNFIYFGVLNLNFSVFHSYFMEFMLRLSIIDNE
MLYRKGGNGILKLIAKDPNKIDKWIKIKEREINKKTFFERHKKILNLFSKSLFVIQNN
IKSIVYKGDKHTIISDYEVLINVLKYDEDFITDFKFLYDDNAVKSGLILSDLHLSFTK
IKMKDKELIKQLFSLFIAKKNIVQFERDIQLKNKIEQLTSLYDILIKSSEKKKLTFRK
NVEDAVKGDPFDYKILENELKSVNDRINNLIENYRKLKDVDYFEGMDNIDEEKMDLNN
DSEFFNNKLLELSFKELKDDITENEQNEDISKKEKEKIYDETTNDDQTK

>MAL8P1.104(Q8IAU8_PLAF7)
MDERTKIVDVWANNLEEEFERIRDIVEKHPYVAIDTEFPGIVARPTGNVLDYNYQTIK
CNVDLLKVIQLGVTFSNGKGEMPNVSTWQFNFKFDLDSDMYAQNSIDFLKLSGINFEK
HQSLGIELLHFGEVIMSSGLVMNEDVKWISFHGCYDFAYLLKILTCSALPHNEAAFFE
LLNDFFPSLYDIKYLLLNLNIKQLSRTFSLQKISEILSVKRIGRQHQAGSDSLVTCKT
FFKLMEMYFDNKIDDKKYSGIIYGLGSTIKNYNPKLDDHNNNRYHNHHNNYNNHNYVK
NSNNNNNNNKYNNNSNNSNNSNNNNNNNNNNNNNNNNSSNNNNNSNNNNNNNSINSHH
SHNNNNMLMNAPNNNPMSNYIDAKEYHPTGFNKEIPKLHNKNDVMFHMYADNNQNSYN
NISSNINNIHGTNNYITNAIYSSDTVNNYVLNKFVNNSPPSPSSSLSSPPHPPHLPHP
PQLLNNTVNKNVRLSLNTSSYMNSKSTDPIGINNHNNNNNLSSSSRSYMASYPSSGIK
NSLDTSKIMGPIYNDIPRNITGNLNSDLNNIANENMMNINGLTNGNNLSPTLSANNGI
MSPSLSNANNGLLNLNNTNLNNSINNTINGSLILGGMNNNGHTNNNINNNHNNNGNAI
NMNMNGMGIMNSSMNISGLSTNLNMNNLYGDIRSLGGSTSNINLNNINSMNELNNLKS
GSISNISNCDENMIGLNKNIYSTNNGPLIGNMINNNVYNANINYNNYNYNSINTNNSS
NNSFLNSVNYNTAYAHGNNPSISPGGNNNMSTNNYYSIQNKYSNFGKYSSNLNYVNPL
SQSINEVKSMAGEDYCDIDKRSIDNLGSSKNSFANNKNLLGKINNNNNNNIISSSSST
SHHINFKNTISNNNSNINNNNLNINSTTSVHPMNNPNMNDTNNLNIKRNMKNESFISD
LSTNKNELNNMGDHVGMENLNMLVQNMNIKRDEKYKDEKINNNNNMSKNMMLNLNNNS
SISNVNNLNNMMNSNNDLIKNNNTTYMKNINMSISNINNGVTNTVLNTDLKVSSNLNQ
NGINVTNSTTYISANSLDNNNNNNNLNSGNNINDHGNNSGKGINHTNPTIINNDNKIN
VASHNKGNTSIDGNWINGPINNTNSSNKYSEGKDKDANTEDNKIGYKANDNNNNSSNN
NRNNNGKVLSNNKNSNKGNNKNSKNKSKNNNDPNKKASGDNLNEPDYKENNMNNNNNN
NNNNNNNNNNNNNNNNNNNNNNNNSNNNNSNNNNSNNNNNSNNNNNNNNNNNNSNNNN
NNSNNNNNNNNNNNNNNNNSNNNNYNNSNNNNNNHSTLISNNDNTIVSNNDNEDNSNH
TTNNNEQDKDDKNNNMKTEGNHSKFSKQHGNMNTRNKNNDKNNFVNNSNLVNSNVEDV
NNFNNIINQRNHCNKNYINFIKEDQQEVDQKELHASNISHMLNEDNSNEIMDKNTSYN
NPYNNNANYYKSDNIKLYRGFEKNSSHQLILKNNFSNNSNMTENEGIIMHDMNKMNNP
NNADILLGNENLLGIKNLNNYMNPNVHNTERNNELNNNNTNMITNKKNVNSCNNDMLH
KQINNFNIININKVHNNISNSLFSNNMSNYNVVSENVNINNNSNTNNTANNNFMNKKN
SKANLNVFNQSLINNLNLNLNNFYLNNENSFNKINMNYSNINSTSYYFNTNDNNMNNT
QHYYSSFIPEHFATDKIVTKQSSENNNNINNSSTMYKEKKEEPNLNFKEKNFVDKNLP
TEKYPGAVLETVDNNFGYPNYEYKTKDHKYFYDN
>MAL8P1.140 (Q8IAP0_PLAF7)
MNISIFFFLFLYSGSICAIHLWRHSEWRGRNVLGGGKCRNNDNMLWLDLIKKNKKVNK
KKNKNNNNNIISSSCCCCCCCCIYNDKYCFLFVNKKIDNNYKRIKKKEKHNCKKRLKS
LNNEGGFSNTYHNDVKKNTLDYCEERLPSYHRYIDNLKTRKIIHPSIRIRNLDKKFMK
CKESYNKLYSHLKENDLFENFSYVGRQKKGILSPTYRLPKYIERPNYHKTGIPIYVPY
DKEKKNNKTNSDHNNKYDHYNCNYDYNYYDNIKSDKDIQIIKENCKFARELMDDVSYI
ICEGITTNDIDIYILNKCINNGFYPSPLNYHNFPKSSCISINEILCHGIPDNNLLYLN
DVVKIDISLFRNGYHADMCESFIVPKLSKNEKKKRKKFYDFIYLNNSFKTKYTKYILK
YHYDLTKNKVVRRGKSFVTKKIKYAAPNSKEQNDDLCNNDFDDNTTNVMNTSQKYCFN
DIYEDKIPSHTNMHTNMHTNTQHTNMHTHTHNRNNSYYPNNQMHNNEKNTFNMSNNV
LQQEDDELEYFHKYYDQKIIFNKENNEIYEDIQKFIYQKSLNKTIGKKRFDFFDNTKM
STNDIKNFMYQKNLDLIKTAYECTMAGISVCKDGTPFNKIAEAMDNYIKQVNKKNNKT
YSIVPHLCGHNIGKNFHEEPYIIHTLNNDQRKMCSNMVFTIEPIISESSTNFILWPDN
WTISNTKYHFSAQFEHTILIQKNGAQILTDKRDISPKYLWQQIN
>MAL8P1.37(Q8IB70_PLAF7)
MKVGKLKKRKNSGLLYPYFKNKSFRLNRYIFIKPIKSVKLNYKKKKMNLTHEICILNC
SEKLIDYKLAFQLQNILHHSKIIMKNKNEVQISNHLELKKFKNFKENMEKYDFCFILQ
HTPCYTLGSVANCSDILLDKENYYIEELGDIYNNLYSNEIIHLMNKCETIQDKINQSD
IYNENTNYFNNFLKHCRQRKIPIYRVNRGGKATYHGPGQLVLYFIFNLKNYPSNYNER

IINKHYKYTNKENFPSKTSEYEKNNIYTNSNSKENISSIERTFDLRTTINNFQKIGME
TLQKFNIKTHCKKDTIGIFYKDKKIISIGLKITKYISMHGLSLNFNLDNNFLKYLLSC
GMNHNDYISMHEINEMKKKNYIYQKGKIASSSNILNELTLNITESLKKVFNVKVRNIK
DIREMFY
>MAL8P1.48 (Q8IB57_PLAF7)
MTLTVGKAGPASDFRKFMEKRLQIYLNGNRQVVGILRGYDTFMNLVLDNTMEIKKDEQ
IDIGVVVIRGNSISYWECLDKVNIK
>MAL8P1.76(Q8IB05_PLAF7)
MATLPASKSASKVALTSEVEDEGIKEHTFQEIEKLQDLGINAADINKLKGSGYCTILS
LIQTTKKELCNVKGISEAKVDKILEVASKIENCSSFITANELVQKRSKVLKITTGSTV
FDQTLGGGIESMCITELFGENRCGKTQVCHTLAVCYIDTEGTFRPEKVCKIAERYGLD
GEAVLDNILYARAFTHEHLYQLLAISAAKMCEEPFALLVVDSIISLFRVDFSGRGELS
ERQQKLNKTMSILSKLSEQFNIAILITNQVMSDPGATMTFIANPMKPVGGHVIGHAST
IRLSLRKGKGDQRVCKVYDAPNLPEVECIFQLSDKGVIDATD
>MAL8P1.9(Q8IAV7_PLAF7)
MSSINIESYLENEILVITNDSRIFTGKLKGFDQTTNIILGNCHERIYKESMEKISLGV
YIIRGDTVTLIGEIDEDVDKNILHQKIKPQMLKPVN
>PF07_0071 (Q8IBS6_PLAF7)
MSEENDATLIKKRKNEKINEEQHNTTNNNYEEPLVYDFLTKEDHENYNNFVRFCKDNF
AYVDMKDINRVIHELRKGPNKDYFSNYISSSDDSDMDDNEKIEYMDDDDDKNKSKVDC
IYDNDKPCNRYKKKKIKDYRKNLKLINIKKNKRARINLIVIKKKYLNDDNNNNNNNNH
NIYNSNNFCSGERVQKNTIILSPFFMPVGTKCCIKGLTLEDVNDICDYIILSNTYHLS
NIYDMSIFEYNKDINNLIKFPNAMLTDSGGFQMVSLSKRIKILEEGILFNNIYNSEVI
KKNIKACNVGDVVKKTYESVCSDMHNDNNNDNVVVRDNVVVRDNVVVRDNVVVRDNVV
AHDIFDEINKNVCDETYNNTNEENSCKTKKYVDMGEDILLSPEISIRLQNFIGSDIIM
ALDDVRSSLEEDKNKIEEATHRTNRWLKRCIDIHKKKEEQSLFGIVQGGLHIDLRNIS
MDYILRQKLNGYAVGGLCGGEKKKKFIEIIHHCSNEKNKKYNYLPTNKCRYIMGIGYI
VDIIFCSLFGYDMYDCVYPSRTARFNTAVSFDGTIKLKQAKYKYDFSRLENNCKCYVC
LKYTKAALHYLISKRNTITNVLLTLHNIYFTLYMCHLMRVAIFSNKLNQFITTFLYNH
FVVGVKNGNYKIPCADDKKMNDDKKMNDEKKMNDDKMMNDEKNINDEKNINDEKNIND
EKNINDDKMMKSDKKHEQGSENLNMKSKNMIEELKKNLPQWAIQALEYADIELMF
>PF07_0073 (Q8IBS3_PLAF7)
MVLDINLFRKEKGGNPDKIKESERKRYHDENNVDKVIEYDDKWRKCIFELEELKKNIN
MINKEIGNKKKVDKNADVEDLKKKSLNIKEEIPKYQLKEKELLKERNKYISKIGNLLN
IKVVCSDNEDNNKIVKTWGECKILPACEENDNSIHDNVVNSNNIKRETLNNEVDNKKK
IKYYYHYDLLRKIGGANFKKGIQVAGHRGYYLTGAGFLLHNAILQYALNFLVNKKYIP
VYPPFFMKKNIMEECAELDDFEETLYKIPSTSNSTLSSQQVSTSPTKISSQADIKDDT
TCNSQKKTNIPSNEDLTRDDLFLIATSEQPLCALHKDETIESKRLPLKYAGFSSCFRK
EAGAHGKDIRGILRVHQFDKVEQFCIALPQHSNKIHEEMIQTCEEFYQSLNIPYRIVS
IVSGALNNAASIKYDLEGFFPTSNQYRELVSCSNCTDYQSINLNIRYSDSSIKINDLN
KNTNLNDEMDSEYEHFLTNFNTENKYHVHLLNGTMVAAQRFLCCLLENYQNGEGIVVP
EKLRPYMNNMDFIPFME
>PF07_0103(Q8IBK8_PLAF7)
MWPVVMLLFGGGVLFVKKGLNYVKNQGIQLNGKRSFFPSGFNKNLNNLFLKNDLKGFE
RNMSKSEAFKILNINPTTNKEKIREVHKQLMLKNHPDNGGSTYIAAKVNEAKDILLK
>PF07_0115 (Q8IBH9_PLAF7)
MRSKYNILIYKKKKKHLRLDVLLFFVYLIFLNLILQNKKFEAQPKDYEYIEILKNKNV
NEETFVHVKRFEPTSSDLGNIGKEKILEQNNYNVKSINNVELRNDNMNVINNEGVYNK
YVKENEEIIGDNEKKEKRAKTFENNDFKNEKSFKPIIVGKYNLIYIFYSIEFICLLLF
IFLHFLLFLLSQWNIKINLFISYKSLYSKNKEKYLYNLKNFCTHVYIEPCIIKSEEDN
GIYDKNRYNMDCNFYKPKSELIELKKIDNDIYFFYKQKKYIFNYETFIFESLKHFDNF

NLSFYLNWKGLLDYPKGKDINKMDIYNNKMMRDIKDRVTCNNIESVIKKLDILYALHN
DKKGNLNIDEKVDDVNFNCNDFISKEKNVDNVYDMNKKNMINIKYRGKTNKGNNNFID
VLKNDNMCEKDINKNDIFGDYNKDDMDDTHNHNVDRNIICSNKKKVDSIKNSIHNDVV
RDDNVNNNFYNFYYKKSCFEIPYDIFVHNNVEKYGENIYDIPCPCFKELLYESMLSPF
FIFQFFSIVLWMLDSYWYFGIFSIFILIIILESQLINKRIREFNMINGMKVDPQDVYVY
RNLRWTIMKSNKLLPGDIYILTNDMTATDNNICTCETLLIDGTCITDESILTGESVPL
IKACIDKSVINNKSNNNNNKNENNNNKNENNNKKKDNNKNENNNKKKDNNKNENNNKK
KDNNKNNDNNKNNSNNNSYRFIGDDNVERLNYKNFENNENEFIKDKECDYESSNYCNS
LFCNRLDIKNKHKKHIVYAGTNILMTKNENNKFNGKKLPVNGCIGIVLRSGFSTYQGK
LVRTIINTSEKVNSSSIDSIIFLMILLLFSICSSAYVVYSVLKTNEERNLYKLLLSVS
HIITAVIPPEFPITLSLAVTISIVYLYNMKIYCTEPFRLPFSGKTNICAFDKTGTLTE
DNMIVLGLFGLDDNLKRINEINESIINKQKIPFFSLSVIAGCHSICTLNNKLLGDPLE
KNSFLKLKCVMKSLNHTYVYTNNINNNNNNNNNNNINNDNVVDKKYHKNNEKKIKNQS
LENFQIVKRFFFSSELQRMTCIILHEGSQHDWYGDEYETDTCDSDEQNEEQYKNTKQH
VLRNNGHEIYKTTPYKESAQMLRKLKRKNNNEYDTESDDHADQNCDIHNNDIHNNDIP
NNDIHNKDIHNKDIHNKDIRHNDVHHNDICYKHTEKEKKKKNRIKNILFVKKKKENKD
KIVKQYLVVSKGSPEIMKRFLKKVPEHYDEVLNSLSIKGYRVLCLAVNILDNNMYKEN
ISREEVEKDLYFCGFLTFICPIKVTTPNYILHIKNAGIKNIMITGDNALTACQVSQDV
NIVPKVTCKDILILKMNEVISYDLIGEKRTNMINMIDIMTTNNNHTCDTNQSDDVCKN
KINNVVKDLRYDHAIFKNDDIKDCIEFLTCLKNKETSVILKNHVENLIRIIEVNYNKC
SNILYFMNRENKKILPFIHNIEYIKVCSEIFSLCITGDIIDYFLEVYKNNLHIFNELI
RGVHIFCRMSPKNKEIIIKTLNKIGYITIMCGDGTNDMAALKAAHVGVSLLSIKISYK
NRDGNRKSVLNDDRKSLLNNHNNMRMMNMYGDGRVKSVYDNLRASYSEARNIINNNSN
NLGGINFRRSYEQMKLYNEKKKELDKMLQSLDDSLPLIKLGEASIASPFTYKGNDIKC
VKEIISCGRCALSKVIMMYKLMIINSLITAFSVSILTLDGVKLSDAQTTIISLLYTCL
IVLISKTSPLKNITNYSPPNSLFNFSVIISLLSQIIIHFSILIYGWKLACVYREINYI
PDIKGDFIPNLVNTCIYYLIYCINLSIFSCNYEGLPFMVPIHKNKEIVYIFAVNFFFL
FVLVMDIFPFLNYFFSLVSFPNIRFKFFFFFLMLVDIFLPYLVTNLFKSLRFYIFHKY
QINI
>PF07_0121 (Q8IBG6_PLAF7)
MNGKKDDELIIEGEHNDKMKSPGKSLIPCIKEKFNETNLKMKIVKKVTFIDEHEDYIK
NKKEIECESESNSYCKKEDENDIKKKEDIDEKESILEQLNGNISNEKNGYHTYESKNC
DFNFKYDDTDNINMYEENIENDIHLNKNNYYHYNKYMNLDYKDNITNEKVIYEKYDKR
EYSENHIHPSTIMNGFSSLKNQEENYSTYENNNIIDNINENLNISYYNQMNGNNDLST
NKEINEYINEKDEEKRKGILKNESPDFNKSYNDSLNNYLSSSFVDILNTLKDVKNENN
DGSYINSNGSYINNPENSNSNNNDDTNLRYISCILCGDSIKANTSKMCHNCILQNIES
NSININKDTYLIYYCRECKRYLHNRWVYCELESKELLALCLKKVNKLKKLKILDAKFL
YTEPHSKRLKVHVSVQEELINNFISEMEIIIHYVIKYTQCDDCKKKYTPYTYNTCVSV
RQKVEHKKTLLFLENLLLKCNMNENIINIVSNPDGLDFHFLSRTDALKFCDFILSKTM
SKCKNSKHLINHDANNNTYNYLYTFSIDICPICKYDLIFFPKGLANKYGIKNVFYLCI
HVSIFIILINPFSSANHVYVSPERYNKNPFLPLLSKADAKVFLILNIEYINDDVNTNN
HKNNNMNPSISNNDNINLNGHTNICKKKNKKRKNKRKKGINTKDDDSDSIEEDNHLLS
NDVNNNHYNYSTNKNNLKRKKKDSNNLEDINKNSFVFSSSDNEEENLHKPNDFDKNSD
IYTDGKSAKSSSRKVKLDKLVYAFVELYDESNGTTILTKTCNAKHLRPGDYVNAYDLR
KHSFDNEINLFLEKNDNYNIIIIDKVKTKEKEKIENYLQVQNNNIETLKRTNEQDIFN
RILLNNCKALENMAITST
>PF08_0011(Q8IBA7_PLAF7)
MFFKFVRFLIYIFLYSLCYQHVNNVFALHINKYKNVFLFESPSVLIKNRRNYKRKIYS
HDEEKGKYDFHRIEEKWQTIWNSKRLLDKDFEQYNKINLGRKEKKEKRHKDKQNGFID
KPYNDVYEKGDNTYKYNKKKFYILDMFPYPSSEGLHVGHILCFTITDIISKFKKMNNY
CVFHPIGWDSFGLPCDRLSMKKKIDPREIIHKNISNFKNQLIKLGFLFNWESEINTCD

ENYYKWTQWIIIQLFLNNLSYKKRSYVNWSNELRCVISNDELRNEINLQYLNIQKIKL
LQWYLKITKYANRLIKDLNMIHWPMKIKNMQINWIGKKTGIFLKARIISLDKFLNNTH
FNFVNIKKSYNNNMINVFYNNIFNHLFFIFINYILSIFTFHMYINNPNEQKQQQKKNT
HIKELNNKIQKYNYINKYDFFFSYHQNDLFLFFSFFFNYYHNRNIDQMKKQKLNLPTE
PFKNEQIRVSHNKENNNNSSNLETNWVSFNIQNCNDMIINILFNNMETYNENYFLYPL
KDATKGKNYINGHNSNDNMFNDNMFNDNMFDDNMFDDNMLKDQNSYVQIFLNKNEVIL
QNDKLIISVNHPNIRNIVNHNPFLLKFIDKMVLENDTVRLKNEELYFTGSVIYFPIMN
KMIPVFLCAYILDNNKNFLFLKKEMTQTQDKNSNTNISLLYKMLQDSNYSKKKNIYNL
KDWLFSRQRYWGEPFPFLYKMKDKNGYMDNTKIKNDDHMNENNHNNNMLSDCKDINNF
SNYNDQNNNKKIRIRKVINSPNNNIYIDDIPLKLPNFNKKIYEIDPHNEQDVNNISSV
LSRFKKWMITKKYNMLYKRESDIMPQWAGSSWYFLRYIDSKNKKRIFNKKKINSWLPV
DLYVGGSEHAVLHLLYSRFFHKFLYDLKLTKHKEPFQKLFNQGLLLNTTSFYLYTTLD
NKPVSFDQINEKKINTNAYDYTNRGTNHHIQLNKKENEKKKKTYSQNVCETTNLLTSQ
VLPDHASFCSGDQNGNINKELKNERNENMKEKDIDILRKSKINDDMKEKAIDMLGKNR
INDDMKEKAIDMLGKNKINDDMKENVIDMLGKNNYTIVDNKYKKFLIEEDYVKEGKDQ
KYYLKSFPSIEVQPNYEKMSKSKGNTINPLDIVKTYGSDCLRLHILFLGPVDQNKKWT
TKGIKGTFKFLNNLYNLFIQRCDIKNNKESNKKNVCTRVVDNSMNDMMFHKDNIEKSA
NHILSNNGYEKKKKINLENGEKKIEYMICKNCKRKSSNKKLILNFLKNKYGFIIANEK
YKKVNDYVNIILKNDSHVFNKLSDKIKKIKIEDIENEKKKKVNYYIEKITNCINDIKL
NTAVSFFMKFYNEIKTWDIVPLKIFIIFVKLLYPFCPHICEEFWFYYLKRYKIKKRKK
LCYFCNSNLLYYGKWPSLFEIKQNKLVNISIKLNNKHITFLQRDVSSTSDITEEATNL
IKHKIENEMKKGKKIVNIVNIPNKVINFIIK
>PF08_0014(Q8IBA7_PLAF7)
MLIYFTNISIYVSKIHHHGLNKKIYNLNFVSNVFKYNARRRGNSLNVLINSPAQVKLS
EMEDAKDRSGKYCIIEICGKIRWVEEGRFYDVFRIKQEENRNIYLNRILFYSNEEGNI
FFGRPFLDNVRIHATVLNHFRGHKIYRLKYKPKKNYKRFYGHRQEMTRIKINKIEYNN
ELLGQEKRIYNFFKDDSLYYILNRIHNIVRPSLELKHLKKNFIEYINSFCSLKFETFY
KHKGNYKKEKMLRNILKTKKLSKRPEVIEEVKKIEEEKKKQRLTKCDPLDDFDPVVNE
FMIKEHFYS
>PF08_0055 (O77370_PLAF7)
MLRRNIRLRKEYLYLKKVEDEKKKYAEKIKSIKESYDKNKKIRGDLKDEESELRKNMN
LYDEKSFDRKVDDEYFFCGLENPRVLITTSRNPSSTLENFAKELKLIIPNSEKINRGS
YFIKDILNFARKNNITDVIILHEYKGIPRNLIICHLPFGPTLFCTIKDCKMRCEFNEK
IDNISLCTPHLIFHNFHSDLGKRIMNIFKYLFPPVTMRTNKRKMPKHNSQIVKDNKLN
KNININTNNNDTNQMDQMDQMNKIKFVTHTTQDDEDNNDHLQIYFKNNEYLNLQKYEN
NRVIVFFNKNDIIYFRHYNWEKNQTNEIVLKEIGPRFSFVVYKINKETLDSLNEDYEY
IYRPFMNSRKALLT
>PFC0831w (O77370_PLAF7)
MLKTLVLLSFYSLYTVFQKSTNCLTIYSSFRKKGKECYVLISSTYILKNKSKKLSRDT
CRILKKFERNNSSDKKFIKTYCIENKNDYMENILTHDEQKKKKKKKKILIANWKCYLS
KEEAYKLIDSLTRIKYSNYVDVILSLNLLYIPYLLQKIKENNSKIYACSQDVSLVNGF
GPFTGETTAKLIQDFGNEYTLIGHSERRQGFYKNGETIEEIVLKVYNAINSKLKVILC
TGDDYKNCDNPSSSYKMMELLRLIKTKISKDEMKNIIIAFEPRFAIGTGQPVSYDILN
KYYYELKRDIAKEIDKSTSEEMMIVYGGSISKSNMKHYVDNTHVDGFLIGKASLNEDF
IDIIRYVDH
>PFC1020c (O97313_PLAF7)
MAVGKNKRTSKGKKGGKKKVTDVFTKKEWYDLKAPKMFLVRNFGKTLVTKTIGKKLAT
DSLKGRIYEVNLADLNNDEDQAHKKIKLSCDHIINRDCYTDFCGLSITRDKLCSLIRK
GYTLIEGHTDVKTLDNYHLRMFCIAFTKKRQNQTKSTCYAQTSQIKKIRKKMVDIMTA
EASKVLLKDLVKKFIPESIGKEIEKQCKKIYPLQNVLIRKVKILKRPKLDISKLMELH
TDPKEESGKNVNALPESKEATNILTAELKH

>PFD0230c(Q8I1Y2_PLAF7)
MILIFQLFLINILFLNFIKCDIPVHCLSRHVEGKWEIHLGLLKKKKNSKQKNVEGVIL
NDKVTGGNVWNISGDIKKNETTAYDNNYDNNYDYQCGYKRPDNADYHDDLNPENEDTK
ERFEEKEKRYIVFNEDRSLNILNEEGDINSRYSGYWKIIYDEGLYIEVYKEDDSKEVY
FSFFKFKQKGDVSYSYCNNLIMGVMNKYSLDNNIWDMRYEENIEENIRSDDKNNNIHM
IKDEKKSVLNIDEKNREISNNISHIYMIRKNVTFFGLRNNKIKKPKRKKMKDKIIISK
GNNNINEIQNDDNYNKIIKEKNFFQLIDYYNDNYISSGRFNIDKFCWYGKKVQEHSEQ
PTNKIPVQNISPLSVNADEYNKLYDEKKKIINNLQKSQDDQLSHSSDNNNNNDNNDNN
DNNDNNDNNDNNDNNNNVQNVVYLRRHKDVFRKYNKGLLLMNRNNKYEKRNSLFYKYI
DKNIDLKNFDWNNKEDIKMRLGHYIKILDDAIDQKDCGSCYANSASFIINSRVRIKYN
YIKNIDSLFFSNEQLILCDIFNQGCNGGYIYLSLKYAYENYLYTQKCFEKYKKKINYN
TDDLEIKSSLMSQDDNSLLCDQFDVFKIKNEKKKNNEINIKEQITMNINNDSNKNQEY
TNNDHDHLLILSYQIQTDPNKKDDNKKNINIYGLNEDYILIDEQKYNNNHNNNDDDEE
YDIFKSNSCDVKINVSKFEYLDIQDEELLKKYIYYNGPVAAAIEPSSEFIGYKKGIIL
GNFIKMYDGTKNNAYIWNKVDHAVVIVGWGEDTLPNFVKKNKLSKETMEHILTTWKEN
NKDNGDSNNNGDSNNNGDSNNNGDSNNNGDSNNNGDSNNNGDNKDNGDSNDNGDNNND
NGDNNDNGDNNIINNTNNVIIKYWKVLNSWGTNWGNSGYFYILRNNNSFNIKSYILAC
DVNLFVKQKET
>PFD0260c(Q8I1Y2_PLAF7)
MEKGSILSFIFFCSVVIFIRFIGYFFCNRYMTEEPYNNIFEIIKPENLYSSLLLLSNE
KENDFPSSTNCNGYMKCIPFYNNVSERWKRYNFIQLYIIRSALNIHVMSKYNMLNKYN
KETNRLLKRNNNVENRINNISNHYLCSGFKKENRLFFLLFYKTIKMMKLYIRNLFMKY
IKIYYKTKHFEKNIETNKKVVYVERDNLFDIERNNLFDILYMLKRIDSYVKNIYSIIS
NNFLYVIRIIFLPFEKIYFSLKSLIMIKKMNMSSSYYYYYVNMFSLYKKNYNKYEEIF
IHEQRVIYPNEYLKNEMLDKYRRVIRILSGQHDNPFIDSLLINPEKIEKDDLDVKQKK
KKIIEELKKKKENTNTNTNTSTNTSANTNTSTNTSANTNTSTKESHILDESKLETFYR
DELDKMGKEEIETYFKGNIDKKSLDEFHKILLEELNKMDKDELYEMYREELNRIEQEK
IRNMNKEEINKTYKDEINNMNSDQVDKIHREELEKIEKEKINKMDKDEIDKIYREELD
KMDRDAIYSMYIEDISNKNIKDLIKNEKETNKDKNKKKDIDINKKKKKDIDIDVDIDK
DIHKDHVEELYGEVKNKLSKEELDRMDRDALYRVYLEELDRMNRDELYRVYLEELEKI
DKEEKEKIHREKLHKIEKEKINKMDKDQIDKIYEEELNKMDSDEIQHVRRAILQDIQK
EKIQNLELEEIDRLYKEELDRMDREARYEIPMRNLSRNEKDNIIHRNIKNESNQKNKK
ENVNVFIIHDNNDSNNNNNNNNRDVNNLNNKHTNNNYNENVEVELVVRNLDKDKGAKI
EDIIDYFNKEIKKDKNVNVSNIVNFLNSKVGKDNTPIQHKKENQVDVVRKNIQIIQED
NIKNKGQKDNTEMLDNNKEITNIDIKNVDDIKNVGDIKSVGDIKSVDDINNVDGIKNV
DGIKNVDGIKNVDGINNVGDINNAGDTNNAGDINNVGDINNSVDIYNVEHIDEAEKKP
NLDNPKKFDWTQVFKDKVTEKIKNEEKFNNSKENIQNDIRDKEIHKDDRIKGITSREK
NAEEINNNEKKDKFVYEFYTSNKKENIDKEEENNIDDKNIKIEIEPNYEINNNFEEEN
KNEINVIIDKEAKNNMDKDDSNNNNNIQKNNIIIKDNTNVSEEVHITESSKEIAEFFN
NIIKNSNILDMCSKMNASDSEKGFICINGNNYIINPGTYHIINIKYPDYNNVRKKWYD
SMDCISINNKDENNNNKEHNYYNKNDDDLYLKKSVEEFIPGFLSNINKVDDLARIFTP
SFIQNDIFLNCIYKYRNDFDKNNNIYSFPMKIFLRKNSTKIKGCSFQIDEDPLLYKDY
SEKESFLSNKIILNNSNRNTECVLHASNEIVGFQCGPPYKSYDNIQYRHLTNKSNDIQ
KNIFGIYSNNNSSYSHLFKNIFNNEHKLYNVGGYFQTEPINCFEFVNDNINVEDILPG
AVPFPRFDLIHHDLDVNQTRYILLNETNQDKTISCTCNYFTEPNIVYTGKIIIKVEEE
KIYKTKKLQTEFNDIINNKKEIYHEKKMNHIIKEKKDEDENDMSFNKNYVNNYNENFK
INDINNFYNRNNHPNNNYHNDYHNNHSSKGNHTNKIHDTFLKNKYNISFNNLKFYNIK
HENKNNQDVINYEYNVDDYHEVQDGQDESFKEEEDFIDFKENIINDNTNHNNTDLDDD
KYNKYHNNNNNNNNNSSFKSIESNLDLQRSILQSGDTQQVVVINKSKNVNVLFPDKKK
NHTHPNEEKRTFPKYISLVYKEKNKDNEKIDKTLTFIKEFYPPLLRGGKDSDPENKQT
GVEINNGVEKKNDVQIKNDVEIKNDVEIKNDVEINDDVEIKNDVEIKNDVEIKNDVEI

NDDVEINDDVEINNGVEINDGVENKDNIHEGNNNLENDSFNEDTIEEPFENIFDFINE
ETSSNENSEIILDSADSIKRKLGHNFLDIISAGKFKIRHKEKKTKNKKKK
>PFD0462w (Q8ILW9)
MLALRILRRKVCSEHFLFERSFFTQSIKGKNGCLVTRYDKNKLLFYYKRNINTSRKCL
NQDPYTVLGLSRNATTNDIKKQFRLLAKKYHPDINPSPDAKQKMASITAAYELLSDPK
KKEFYDKTGMTDDSNYQNHSSNFEGAFSGFGDASFMFTDFAEMFTNMAGGNKNTSTRG
EDIQSEITLKFMEAIKGCEKNIRLNVKVSCNNCNGSGKKPGTNLTICKVCNGSGIQRM
ERGPIIIGVPCRNCSGNGQIINNPCKHCSGSGVKFQTKNITLDIPPGIKKGMQMRIPN
QGHCGYRGGKSGHLFVTINIEPHKIFKWVDDNIYVDVPLTIKQCLLGGLVTVPTLNGD
MDLLIKPKTYPNSEKILKGKGPCKVDSHNNGDLIIKFSLKIPEKLTPRQVELIEEFNT
IELNLPNPQTNVKQKKNIYETKGNINENIFSMNNTYNNMKGPEGETSNTQAKSMKNQN
WNNEKSVNNKGTISKDEKKLNMKNNHINEKSNLKNSSHMDTNKNEENMSDDEKKKIKK
IIPEPPMPHTHKIVNNLESKNSCNIPIPPPPPKSSSKPISENQNISNREHNGVTNNSA
KLDNNINMNYSCDPYKNVTQNDLNNNDNIKNKIYKDNTNISNHHIFKNDNINQQQFHC
ADNSSENNNESDMNTTSTFSFAKKWISDKLKPKN
>PF10_0053(Q8I716_PLAF7)
MKIYFIFILFLYLNFFILIIEILSKDIIKTRRSFPSYIKDGEKRKSNKIKRKRIKNDA
YINVYRKNILDSINIKGDQNKIKEKWLQNLKKKKAFDKISVRLSNYNDDKKEIFDITF
VNNKEDRNDDNYDNKSCIKKVCNAEEYLLISTPVYYSNDRPHIGHAYCNILCDVLYKY
EKLKGVEKKKKGIIFFSGMDEHGLKIEKKCNKNKMKIIEYIDDISNYYKDMNKKLSVD
VNLFYRTSYSFHKMFVQNVWKYLVHNNYIYKDTYKGYYNINEERYISEQELREGKYKI
DNENIIYVEEENSYFFNILKFKDYLIDFYEKNENFIYPPYLRKQVIYTLKNELRNICI
SRYNTKWAIQIPNEAEGTIYVWFDALLSYVSSMLYLHKIKKNNNNNNNNNNKNNNNNN
NKNNNNKNNKNNNNVINCTNGTKDVNISSTCDTDSPICSYDDILNIVNLNANNNNTQS
NILPHISSNICDEKKNEKREKNIPFNKVIGKKFWNPFIQVIGKDILNFHAILYICLLK
SLNLELPQKILCHGLIKNENIKMSKSLNNVVNPFDLLKKYNPDVLRLYFMGCGSIYED
KNYKEQNIESFELFLRNNVGNLLYRVVSLCIENNYNMVPVIKTNDYDSNIILNEWKDN
IKNKLIPYLDNMEYIQFLELIMTLIKNVNKFFVHNTPWNYKKDTQHFHTTIYVTLECM
KYFSILMFPFIPNISLAILRNIGFDDIDEENISLDMLETRTTKFMLNGLIKIV
>PF10_0149(Q8IJP3_PLAF7)
MDNANKLPKWNQPSKEGKKITNLFVNNSLTHSKVEFIPQEGNKIKWYACGPTVYDAAH
LGHARTYVSFDIIRRILVNYFKYDVFMVINITDIDDKIIKRSVEEKIGFTELARKWEY
EFWEDMKSLNVLLPTAITRVSEYVGDIVKYIEKIIENKYAYVSEEGSVYFDIDEFKKS
EKHFYARMEPLSVKDENKILEGEGDLGVISKKKKNAYDFALWKSSKPNEPHWDSPWGK
GRPGWHIECSTMASNILGDVLDIHSGGIDLRFPHHDNELAQSEAFFDHSQWVNYFLHS
GHLHIEGLKMSKSLKNFITIKNMLTKYTSNQIRILFLLNKWDNFMNYSPNGESMVQCI
EIDKSFTNFFAIILMKIKNFDLNSCNLYWSDADNKLNLLFRQTKNKIHEHFLDNFNTP
DALLAIQKLITEINIYMDKEKIQIGLLLEIKHYINFIFDTFGLIYGDAPKGKYDKFDE
LLQTLGTYRRNIRINLQSNAKLIRNILKEKNKKNDLDPVAAQEKSELLHSEFINNIKA
NNELLLKECDLLRDQHLLNMGILIDDRPNNEFVIKIIDDNQLQQEKNKREQELSKKMA
GDQKKGNQNEKRE
>ABRA_PLAF7
MMNMKIVLFSLLLFVIRWNIISCNKNDKNQGVDMNVLNNYENLFKFVKCEYCNEHTYV
KGKKAPSDPQCADIKEECKELLKEKQYTDSVTYLMDGFKSANNSANNGKKNNAEEMKN
LVNFLQSHKKLIKALKKNIESIQNKKHLIYKNKSYNPLLLSCVKKMNMLKENVDYIQK
NQNLFKELMNQKATYSFVNTKKKIISLKSQGHKKETSQNQNENNDNQKYQEVNDEDDV
NDEEDTNDDEDTNDEEDTNDDEDTNDDEDTNDEEDTNDEEDHENNNATAYELGIVPVN
DVLNVNMKNMITGNNFMDVVKNTLAQSGGLGSNDLINFLNQGKEIGENLLNITKMNLG
DKNNLESFPLDELNMLKDNLINYEFILDNLKTSVLNKLKDLLLRLLYKAYVSYKKRKA
QEKGLPEPTVTNEEYVEELKKGILDMGIKLLFSKVKSLLKKLKNKIFPKKKEDNQAVD
TKSMEEPKVKAQPALRGVEPTEDSNIMNSINNVMDEIDFFEKELIENNNTPNVVPPTQ

SKKKNKNETVSGMDENFDNHPENYFKEEYYYDENDDMEVKVKKIGVTLKKFEPLKNGN
VSETIKLIHLGNKDKKHIEAINNDIQIIKQELQAIYNELMNYTNGNKNIQQIFQQNIL
ENDVLNQETEEEMEKQVEAITKQIEAEVDALAPKNKEEEEKEKEKEKEEKEKEEKEKEKE
EKEKEEKEKEEKEKEEKEKEEEKKEKEEEQEEEEEEEIVPENLTTEESK
>ABRA_PLAFC
MMNMKIVLFSLLLFVIRWNIISCNKNDKNQGVDMNVLNNYENLFKFVKCEYCNEHTYV
KGKKAPSDPQCADIKEECKELLKEKQYTDSVTYLMDGFKSANNSANNGKKNNAEEMKN
LVNFLQSHKKLIKALKKNIESIQNKKHLIYKNKSYNPLLLSCVKKMNMLKENVDYIQK
NQNLFKELMNQKATYSFVNTKKKIISLKSQGHKKETSQNQNENNDNQKYQEVNDEDDV
NDEEDTNDDEDTNDEEDTNDDEDTNDDEDTNDEEDTNDEEDHENNNATAYELGIVPVN
DVLNVNMKNMITGNNFMDVVKNTLAQSGGLGSNDLINFLNQGKEIGENLLNITKMNLG
DKNNLESFPLDELNMLKDNLINYEFILDNLKTSVLNKLKDLLLRLLYKAYVSYKKRKA
QEKGLPEPTVTNEEYVEELKKGILDMGIKLLFSKVKSLLKKLKNKIFPKKKEDNQAVD
TKSMEEPKVKAQPALRGVEPTEDSNIMNSINNVMDEIDFFEKELIENNNTPNVVPPTQ
SKKKNKNETVSGMDENFDNHPENYFKEEYYYDENDDMEVKVKKIGVTLKKFEPLKNGN
VSETIKLIHLGNKDKKHIEAINNDIQIIKQELQAIYNELMNYTNGNKNIQQIFQQNIL
ENDVLNQETEEEMEKQVEAITKQIEAEVDALAPKNKEEEEKEKEKEKEKEEKEKEEKE
KEEKEKEKEEKEKEKEEKEEEKKEKEEEQEEEEEEIVPENLTTEESK
>ABRA_PLAFF
HYKKRKAQEKGLPEPTVTNEEYVEELKKGILDMGIKLLFSKVKSLLKKLKNKIFPKKK
EDNQAVDTKSMEEPKVKAQPALRGVEPTEDSNIMNSINNVMDEIDFFEKELIENNNTP
NVVPPTQSKKKNKNETVSGMDENFDNHPENYFKEEYYYDENDDMEVKVKKIGVTLKKF
EPLKNGNVSETIKLIHLGNKDKKHIEAINNDIQIIKQELQAIYNELMNYTNGNKNIQQ
IFQQNILENDVLNQETEEEMEKQVEAITKQIEAEVDALAPKNKEEEEKEKEKEKEKEE
KEKEEKEKEKEEKEKEEKEKEEKEEKEEEKK
>ABRA_PLAFG
QYTDSVTYLMDGFKSANNSANNGKKNNAEEMKNLVNFLQSHKKLIKALKKNIESIQNK
KHLIYKNKSYNPLLLSCVKKMNMLKENVDYIQKNQNLFKELMNQKATYSFVNTKKKII
SLKSQGHKKETSQNQNENNDNQKYQEVNDEDDVNDEEDTNDDEDTNDEEDTNDDEDTN
DDEDTNDEEDTNDEEDHENNNATAYELGIVPVNDVLNVNMKNMITGNNFMDVVKSTLA
QSGGLGSNDLINFLNQGKEIGENLLNITKMNLGDKNNLESFPLDQLNMLKDNLINYEF
ILNNLKTSVLNKLKDLLLRLLYKAYVSYKKRKAQEKGLPEPTVTNEEYVEELKKGILD
MGIKLLFSKVKSLLKKLKNKIFPKKKEDNQAVDTKSMEEPKVKAQPALRGVEPTEDSN
IMNSINNVMDEIDFFEKELIENNNTPNVVPPTQSKKKNKNETVSGMDENFDNHPENYF
KEEYYYDENDDMEVKVKKIGVTLKKFEPLKNGNVSETIKLIHLGNKDKKHIEAINNDI
QIIKQELQAIYNELMNYTNGNKNIQQIFQQNILENDVLNQETEEEMEKQVEAITKQIE
AEVDALAPKNKEEEEKEKEK
>ACT1_PLAF7
MGEEDVQALVVDNGSGNVKAGVAGDDAPRSVFPSIVGRPKNPGIMVGMEEKDAFVGDE
AQTKRGILTLKYPIEHGIVTNWDDMEKIWHHTFYNELRAAPEEHPVLLTEAPLNPKGN
RERMTQIMFESFNVPAMYVAIQAVLSLYSSGRTTGIVLDSGDGVSHTVPIYEGYALPH
AIMRLDLAGRDLTEYLMKILHERGYGFSTSAEKEIVRDIKEKLCYIALNFDEEMKTSE
QSSDIEKSYELPDGNIITVGNERFRCPEALFQPSFLGKEAAGIHTTTFNSIKKCDVDI
RKDLYGNIVLSGGTTMYEGIGERLTRDITTLAPSTMKIKVVAPPERKYSVWIGGSILS
SLSTFQQMWITKEEYDESGPSIVHRKCF
>ACT1_PLAFA
GEEVVQALVVDNGSGNVKAGVAGDDAPRSVFPSIVGRPKNPGIMVGMEEKDAFVGDEA
QTKRGILTLKYPIEHGIVTNWDDMEKIWHHTFYNELRAAPEEHPVLLTEAPLNPKGNR
ERMTQIMFESFNVPAMYVAIQAVLSLYSSGRTTGIVLDSGDGVSHTVPIYEGYALPHA
IMRLDLAGRDLTEYLMKILHERGYGFSTSAEKEIVRDIKEKLCYIALNFDEEMKTSEQ
SSDIEKSYELPDGNIITVGNERFRCPEALFQPSFLGKEAAGIHTTTFNSIKKCDVDIR

KDLYGNIVLSGGTTMYEGTGERLTRDITTLAPSTMKIKVVAPPERKYSVWIGGSILSS
LSTFQQMWITKEEYDESGPSIVHRKCF
>ACT2_PLAF7
MSEEAVALVVDNGSGMVKSGLAGDDAPKCVFPSIVGRPKMPNIMIGMEQKECYVGDEA
QNKRGILTLKYPIEHGIVTNWDDMEKIWHHTFYNELRVSPEEHPVLLTEAPLNPKTNR
EKMTQIMFETFDVPAMYVSIQAILSLYASGRTTGIVLDSGDGVSHTVPIYEGYVLPHA
INRIDMAGRDLTYHMMKLFTERGHTFTTTAEREIVRDIKEKLCYIAMDYDEELKRSEE
HSDEIEEIYELPDGNLITVGSERFRCPEALFNPTLIGRECPGLHITAYQSIMKCDIDI
RKELYNNIVLSGGTTMYNNIGERLTKEMTNLAPSSMKIKVIAPPERKYSVWIGGSILS
SLSTFQQMWITKEEYEDSGPSIVHRKCF
>ACT2_PLAFA
MSEEAVALVVDNGSGMVKSGLAGDDAPKCVFPSIVGRPKMPNIMIGMEQKECYVGDEA
QNKRGILTLKYPIEHGIVTNWDDMEKIWHHTFYNELRVSPEEHPVLLTEAPLNPKTNR
EKMTQIMFETFDVPAMYVSIQAILSLYASGRTTGIVLDSGDGVSHTVPIYEGYVLPHA
INRIDMAGRDLTYHMMKWFTERGHTFTTTAEREIVRDIKEKLCYIAMDYDEELKRSEE
HSDEIEEIYELPDGNLITVGSERFRCPEALFNPTLIGRECPGLHITAYQSIMKCDIDI
RKELYNNIVLSGGTTMYNNIGERLTKEMTNLAPSSMKIKVIAPPERKYSVWIGGSILS
SLSTFQQMWITKEEYEDSGPSIVHRKCF
>ALF_PLAF7
MAHCTEYMNAPKKLPADVAEELATTAQKLVQAGKGILAADESTQTIKKRFDNIKLENT
IENRASYRDLLFGTKGLGKFISGAILFEETLFQKNEAGVPMVNLLHNENIIPGIKVDK
GLVNIPCTDEEKSTQGLDGLAERCKEYYKAGARFAKWRTVLVIDTAKGKPTDLSIHET
AWGLARYASICQQNRLVPIVEPEILADGPHSIEVCAVVTQKVLSCVFKALQENGVLLE
GALLKPNMVTAGYECTAKTTTQDVGFLTVRTLRRTVPPALPGVVFLSGGQSEEEASVN
LNSINALGPHPWALTFSYGRALQASVLNTWQGKKENVAKAREVLLQRAEANSLATYGK
YKGGAGGENAGASLYEKKYVY
>ALF_PLAFA
MAHCTEYMNAPKKLPADVAEELATTAQKLVQAGKGILAADESTQTIKKRFDNIKLENT
IENRASYRDLLFGTKGLGKFISGAILFEETLFQKNEAGVPMVNLLHNENIIPGIKVDK
GLVNIPCTDEEKSTQGLDGLAERCKEYYKAGARFAKWRTVLVIDTAKGKPTDLSIHET
AWGLARYASICQQNRLVPIVEPEILADGPHSIEVCAVVTQKVLSCVFKALQENGVLLE
GALLKPNMVTAGYECTAKTTTQDVGFLTVRTLRRTVPPALPGVVFLSGGQSEEEASVN
LNSINALGPHPWALTFSYGRALQASVLNTWQGKKENVAKAREVLLQRAEANSLATYGK
YKGGAGGENAGASLYEKKYVY
>AMA1_PLAF8
MRKLYCVLLLSAFEFTYMINFGRGQNYWEHPYQKSDVYHPINEHREHSKEYEYPLHQE
HTYQQEDSGEDENTLQHAYPIDHEGAEPAPQEQNLFSSIEIVERSNYMGNPWTEYMAK
YDIKEVHGSGIRVDLGEDAEVAGTQYRLPSGKCPVFGKGIIIENSNTTFLKPVATGNQ
DLKDGGFAFPPTNPLISPMTLDHMRDFYKNNEYVKNLDELTLCSRHAGNMNPDNDKNS
NYKYPAVYDYNDKKCHILYIAAQENNGPRYCNKDESKRNSMFCFRPAKDKSFQNYTYL
SKNVVDNWEKVCPRKNLENAKFGLWVDGNCEDIPHVNEFSANDLFECNKLVFELSASD
QPKQYEQHLTDYEKIKEGFKNKNASMIKSAFLPTGAFKADRYKSRGKGYNWGNYNRKT
QKCEIFNVKPTCLINNSSYIATTALSHPNEVEHNFPCSLYKDEIKKEIERESKRIKLN
DNDDEGNKKIIAPRIFISDDIDSLKCPCDPEIVSNSTCNFFVCKCVEKRAEVTSNNEV
VVKEEYKDEYADIPEHKPTYDKMKIIIASSAAVAVLATILMVYLYKRKGNAEKYDKMD
EPQDYGKSNSRNDEMLDPEASFWGEEKRASHTTPVLMEKPYY
>AMA1_PLAFC
MRKLYCVLLLSAFEFTYMINFGRGQNYWEHPYQNSNVYHPINEHREHPKEYQYPLHQE
HTYQQEDSGEDENTLQHAYPIDHEGAEPAPQEQNLFSSIEIVERSNYMGNPWTEYMAK
YDIEEVHGSGIRVDLGEDAEVAGTQYRLPSGKCPVFGKGIIIENSNTTFLKPVATGNQ
DLKDGGFAFPPTEPLISPMTLNGMRDFYKNNEYVKNLDELTLCSRHAGNMNPDKDENS

NYKYPAVYDDKDKKCHILYIAAQENNGPRYCNKDESKRNSMFCFRPAKDKSFQNYTYL
SKNVVDNWEKVCPRKNLENAKFGLWVDGNCEDIPHVNEFSANDLFECNKLVFELSASD
QPKQYEQHLTDYEKIKEGFKNKNASMIKSAFLPTGAFKADRYKSHGKGYNWGNYNRKT
HKCEIFNVKPTCLINNSSYIATTALSHPIEVENNFPCSLYKNEIMKEIERESKRIKLN
DNDDEGNKKIIAPRIFISDDKDSLKCPCDPEMVSNSTCRFFVCKCVERRAEVTSNNEV
VVKEEYKDEYADIPEHKPTYDNMKIIIASSAAVAVLATILMVYLYKRKGNAEKYDKMD
QPQDYGKSTSRNDEMLDPEASFWGEEKRASHTTPVLMEKPYY
>AMA1_PLAFF
MRKLYCVLLLSAFEFTYMINFGRGQNYWEHPYQKSDVYHPINEHREHPKEYQYPLHQE
HTYQQEDSGEDENTLQHAYPIDHEGAEPAPQEQNLFSSIEIVERSNYMGNPWTEYMAK
YDIEEVHGSGIRVDLGEDAEVAGTQYRLPSGKCPVFGKGIIIENSNTTFLTPVATGNQ
YLKDGGFAFPPTEPLMSPMTLDEMRHFYKDNKYVKNLDELTLCSRHAGNMIPDNDKNS
NYKYPAVYDDKDKKCHILYIAAQENNGPRYCNKDESKRNSMFCFRPAKDISFQNYTYL
SKNVVDNWEKVCPRKNLQNAKFGLWVDGNCEDIPHVNEFSAIDLFECNKLVFELSASD
QPKQYEQHLTDYEKIKEGFKNKNASMIKSAFLPTGAFKADRYKSHGKGYNWGNYNTET
QKCEIFNVKPTCLINNSSYIATTALSHPIEVEHNFPCSLYKNEIMKEIERESKRIKLN
DNDDEGNKKIIAPRIFISDDKDSLKCPCDPEIVSNSTCNFFVCKCVERRAEVTSNNEV
VVKEEYKDEYADIPEHKPTYDKMKIIIASSAAVAVLATILMVYLYKRKGNAEKYDKMD
EPQHYGKSNSRNDEMLDPEASFWGEEKRASHTTPVLMEKPYY
>AMA1_PLAFG
MRKLYCVLLLSAFEFTYMINFGRGQNYWEHPYQKSGVYHPINEHREHPKEYEYPLHQE
HTYQQEDSGEDENTLQHAYPIDHEGAEPAPQEQNLFSSIEIVERSNYMGNPWTEYMAK
YDIEEVHGSGIRVDLGEDAEVAGTQYRLPSGKCPVFGKGIIIENSNTTFLKPVATGNQ
DLKDGGFAFPPTNPLISPMTLNGMRDFYKNNEYVKNLDELTLCSRHAGNMNPDNDKNS
NYKYPAVYDYNDKKCHILYIAAQENNGPRYCNKDQSKRNSMFCFRPAKDKLFENYTYL
SKNVVDNWEEVCPRKNLENAKFGLWVDGNCEDIPHVNEFSANDLFECNKLVFELSASD
QPKQYEQHLTDYEKIKEGFKNKNASMIKSAFLPTGAFKADRYKSHGKGYNWGNYNRET
QKCEIFNVKPTCLINNSSYIATTALSHPIEVEHNFPCSLYKDEIKKEIERESKRIKLN
DNDDEGNKKIIAPRIFISDDKDSLKCPCDPEMVSNSTCRFFVCKCVERRAEVTSNNEV
VVKEEYKDEYADIPEHKPTYDNMKIIIASSAAVAVLATILMVYLYKRKGNAEKYDKMD
QPQHYGKSTSRNDEMLDPEASFWGEEKRASHTTPVLMEKPYY
>AMA1_PLAFH
MRKLYCVLLLSAFEFTYMINFGRGQNYWEHPYQNSDVYRPINEHREHPKEYEYPLLQE
HTYQQEDSGEDENTLQHAYPIDHEGAEPAPQEQNLFSSIEIVERSNYMGNPWTEYMAK
YDIEKVHGSGIRVDLGEDAEVAGTQYRLPSGKCPVFGKGIIIENSKTTFLTPVATENQ
DLKDGGFAFPPTEPLISPMTLDQMRHLYKDNEYVKNLDELTLCSRHAGNMNPDNDKNS
NYKYPAVYDYEDKKCHILYIAAQENNGPRYCNKDESKRNSMFCFRPAKDKLFENYTYL
SKNVVDNWEEVCPRKNLENAKFGLWVDGNCEDIPHVNEFSANDLFECNKLVFELSASD
QPKQYEQHLTDYEKIKEGFKNKNASMIKSAFLPTGAFKADRYKSRGKGYNWGNYNTET
QKCEIFNVKPTCLINNSSYIATTALSHPNEVENNFPCSLYKDEIKKEIERESKRIKLN
DNDDEGNKKIIAPRIFISDDKDSLKCPCDPEIVSNSTCNFFVCKCVEKRAEVTSNNEV
VVKEEYKDEYADIPEHKPTYDNMKIIIASSAAVAVLATILMVYLYKRKGNAEKYDKMD
EPQDYGKSTSRNDEMLDPEASFWGEEKRASHTTPVLMEKPYY
>AMP1_PLAFQ
MKLTKGCAYKYIIFTVLILANILYDNKKRCMIKKNLRISSCGIISRLLKSNSNYNSFN
KNYNFTSAISELQFSNFWNLDILQKDIFSNIHNNKNKPQSYIIHKRLMSEKGDNNNNN
HQNNNGNDNKKRLGSVVNNEENTCSDKRMKPFEEGHGITQVDKMNNNSDHLQQNGVMN
LNSNNVENNNNNNSVVVKKNEPKIHYRKDYKPSGFIINNVTLNINIHDNETIVRSVLD
MDISKHNVGEDLVFDGVGLKINEISINNKKLVEGEEYTYDNEFLTIFSKFVPKSKFAF
SSEVIIHPETNYALTGLYKSKNIIVSQCEATGFRRITFFIDRPDMMAKYDVTVTADKE
KYPVLLSNGDKVNEFEIPGGRHGARFNDPHLKPCYLFAVVAGDLKHLSATYITKYTKK

KVELYVFSEEKYVSKLQWALECLKKSMAFDEDYFGLEYDLSRLNLVAVSDFNVGAMEN
KGLNIFNANSLLASKKNSIDFSYARILTVVGHEYFHNYTGNRVTLRDWFQLTLKEGLT
VHRENLFSEEMTKTVTTRLSHVDLLRSVQFLEDSSPLSHPIRPESYVSMENFYTTTVY
DKGSEVMRMYLTILGEEYYKKGFDIYIKKNDGNTATCEDFNYAMEQAYKMKKADNSAN
LNQYLLWFSQSGTPHVSFKYNYDAEKKQYSIHVNQYTKPDENQKEKKPLFIPISVGLI
NPENGKEMISQTTLELTKESDTFVFNNIAVKPIPSLFRGFSAPVYIEDNLTDEERILL
LKYDSDAFVRYNSCTNIYMKQILMNYNEFLKAKNEKLESFNLTPVNAQFIDAIKYLLE
DPHADAGFKSYIVSLPQDRYIINFVSNLDTDVLADTKEYIYKQIGDKLNDVYYKMFKS
LEAKADDLTYFNDESHVDFDQMNMRTLRNTLLSLLSKAQYPNILNEIIEHSKSPYPSN
WLTSLSVSAYFDKYFELYDKTYKLSKDDELLLQEWLKTVSRSDRKDIYEILKKLENEV
LKDSKNPNDIRAVYLPFTNNLRRFHDISGKGYKLIAEVITKTDKFNPMVATQLCEPFK
LWNKLDTKRQELMLNEMNTMLQEPNISNNLKEYLLRLTNKL
>AN32_PLAF7
MEKAIRDRVEKWKKEHTIDGKDATNEHAYESLNELILDGKKLTSIKNEEKELLKNFKN
LERLCLNQTGIQTLENIPSIATLNVLELTDNHLSSVEVLKYIVQNFPNIKTLEIGGNH
FKNINDFETLKELKNLVRLGVQFNPFADNPNYRKELFEFLPNVKIIDCYNKEGMEVLS
SDEEEEEEYEEDNTLKNFYEADFKDEDDEDEEFVPNDNEDDDEDDELDDDLEDEDMED
LDKEDLDKEDYDIDTKETEGVNKDEKSNKRKQDALDNTNDMDLKKTKLE
>ARF1_PLAFA
GLYVSRLFNRLFQKKDVRILMVGLDAAGKTTILYKVKLGEVVTTIPTIGFNVETVEFR
NISFTVWDVGGQDKIRPLWRHYYSNTDGLIFVVDSNDRERIDDAREELHRMINEEELK
DAIILVFANKQDLPNAMSAAEVTEKLHLNTIRERNWFIQSTCATRGDGLYEGFDWLTT
HLNNAK
>ARF1_PLAFO
GLYVSRLFNRLFQKKDVRILMVGLDAAGKTTILYKVKLGEVVTTIPTIGFNVETVEFR
NISFTVWDVGGQDKIRPLWRHYYSNTDGLIFVVDSNDRERIDDAREGLHRMINEEELK
DAIILVFANKQDLPNAMSAAEVTEKLHLNTIRERNWFIQSTCATRGDGLYEGFDWLTT
HLNNAK
>ARP2_PLAFA
MNNEIVNSTIDGVLNTKLHIQNIPPHITDVHLRSLLGNVGFIKDICYFNKSKKQMNNN
NFANKKIYNTALVTFNTHEEALNVLKNIKNLIDTSGEERNIDAKFAVPNVSINNNNNN
NNSNTFFQKNNMNNTNFSQGSTNYGSNYNSENFQGNNNMNNYNFYNNNSSNNNNNNQT
NTQNNFMNRNMKNKNMNNNNNNNNSNNNMMMNMNFNNNQQMNNNPMLNQNNFMLNNNN
NYNNNAKNVNDMYRDGEMSPNHLNNNNNNINNNNNNNNNNNNNNNVMFRQNNSHLAQM
YQANDNSLEDVENVDGLSLWEMYKDKNNNIFYYNNLTKHANGINLFTQTNYSSITIMK
KQNKMDLVEVTYLFSTYLVNGQTLIYSNISVVLVILYHQKFKETVLGRNSGFGFVSYD
NVISAQHAIQFMNGYFVNNKYLKVQLKKGETVENTNS
>ARP_PLAFA
YNNNNKNNNNNDDGNINYQNTNEFKDNKKNMNFKNQYNNNYKFDENMNNSNTMHSRNS
NVEEHLRNNSIDMNNSNINNYTNQQTRFSSFMENENENENKNYHTGGMNNNIHFKNKY
DNNNSSMKNTDNNKTDTSYNMKGTINNDNNNMDYLRNINNINEYKGSAKNKFYTNYMN
KNNLKFTQNNNDNMNINEDNNNNNNNNNNNNGVFSNYQNNNMNRNNSINIKRNLNNNN
NINNNMNKMGSQDKNQNSNNNFYMNYNYQNRKNSMNNNMNNNMNNNMNHNMNNNNMNHN
MNNNMNHNMNNNNMNHNMNNNNMNNINSLDSDMSPNYHAHVKMSMMNYNNNESNTANPNQ
MNFEQTNNDNMKRENNNMNNYGYDDNTVHVNNNTPSTDFFSRAVGYNNNYLNNNNNMN
SAVNNNSSNGNNMKNENSENKNVADNNDSLNNNKNNNNNINMNESINNNNTLNNNNEY
NNQNNNEDEDDDDWGELGEDKYIDINSIMKKKNVILNQLEADLNDLSKKGNDGKNKKK
NKMKKDDLFVLPHTN
>ASP_PLAFS
MYLFIYIFFFFFFFFFFVIVQKDIEQLDIKCAHEQMNIQKQYDEKKKPLFEKRDEIIQ
KIPGFWANTLRKHPALSDIVPEDIDILNHLVKLDLKDNMDNNGSYKITFIFGEKAKEF

MEPLTLVKHVTFDNNQEKVVECTRIKWKEGKNPIAAVTHNRSDLDNEIPKWSIFEWFT
TDELQDKPDVGELIRREIWHNPLSYYLGLEEFDEFDDDFDEEFDDDDDDDDDDDDDDD
DDDKDDDLDGDDDGNNDDNDD
>ATC_PLAFK
MEEVIKNAHTYDVEDVLKFLDVNKDNGLKNEELDDRRLKYGLNELEVEKKKSIFELIL
NQFDDLLVKILLLAAFISFVLTLLDMKHKKIEICDFIEPLVIVLILILNAAVGVWQEC
NAEKSLEALKELQPTKAKVLRDGKWEIIDSKYLYVGDIIELSVGNKTPADARIIKIYS
TSLKVEQSMLTGESCSVDKYAEKMEDSYKNCEIQLKKNILFSSTAIVCGRCIAVVINI
GMKTEIGHIQHAVIESNSEDTQTPLQIKIDLFGQQLSKIIFVICVTVWIINFKHFSDP
IHGSFLYGCLYYFKISVALAVAAIPEGLPAVITTCLALGTRRMVKKNAIVRKLQSVET
LGCTTVICSDKTGTLTTNQMTTTVFHLFRESDSLTEYQLCQKGDTYYFYESSNLTNDI
YAGESSFFNKLKDEGNVEALTDDGEEGSIDEADPYSDYFSSDSKKMKNDLNNNNNNNN
NSSRSGAKRNIPLKEMKSNENTIISRGSKILEDKINKYCYSEYDYNFYMCLVNCNEAN
IFCNDNSQIVKKFGDSTELALLHFVHNFDILPTFSKNNKMPAEYEKNTTPVQSSNKKD
KSPRGINKFFSSKNDNSHITSTLNENDKNLKNANHSNYTTAQATTNGYEAIGENTFEH
GTSFENCFHSKLGNKINTTSTHNNNNNNNNNSNSVPSECISSWRNECKQIKIIEFTRE
RKLMSVIVENKKKEIILYCKGAPENIIKNCKYYLTKNDIRPLNETLKNEIHNKIQNMG
KRALRTLSFAYKKLSSKDLNIKNTDDYYKLEQDLIYLGGLGIIDPPRKYVGRAIRLCH
MAGIRVFMITGDNINTARAIAKEINILNKNEGDDEKDNYTNNKNTQICCYNGREFEDF
SLEKQKHILKNTPRIVFCRTEPKHKKQIVKVLKDLGETVAMTGDGVNDAPALKSADIG
IAMGINGTEVAKEASDIVLADDNFNTIVEAIKEGRCIYNNMKAFIRYLISSNIGEVAS
IFITALLGIPDSLAPVQLLWVNLVTDGLPATALGFNPPEHDVMKCKPRHKNDNLINGL
TLLRYIIIGTYVGIATVSIFVYWFLFYPDSDMHTLINFYQLSHYNQCKAWNNFRVNKV
YDMSEDHCSYFSAGKIKASTLSLSVLVLIEMFNALNALSEYNSLFEIPPWRNMYLVLA
TIGSLLLHVLILYIPPLARIFGVVPLSAYDWFLVFLWSFPVIILDEIIKFYAKRKLKE
EQRTKKIKID
>ATX1_PLAFA
MHLMCTGLRNEKLINDRKILYGECNLNIKSDSFIILLFKEIMNPFFIFQIFAMIVWSL
DNYIEYTISILFITSISIILELKNTIKNQKKIKNMLNYTCPINVYRYNTSYIISSSEL
VPGDIYEIKNNMTIPCDTIILSGSVTMSEHMLTGESVPIHKERLPFEGNAIINKNNKY
DSNDEKDDYLRIYNNHASINMIKRNHLIEETLGKKDREYKSNTHDLCSMNKLCYINNT
YDDVHMKNNKMDYNNNNNNKKKKKINNLNFVKGTYINSNDLLYDDKIGVNIFEDDVNN
MKHKFNQRNINYYNKDTNNLEYNNKHRYIYDCLLKKVEAISQKNKIIYSNEDINKYML
YGGTYVLSLYNINKIKYNNKEENRILGLVIKTGFITTKGKIVNNILYHKKKELNLIND
SYKFLIILIIYALFSVFILLYITLSNNEYTNHIIIKCLDIITDAIPPALPTTLTVGIS
IAISRLKKKFSISCLCPHKINIAGQINTMVFDKTGTLTENNLQFIGIITQNKKNKNML
SDFIHIKEMNTESYIHSKDDNMIHNKNSIISEYYIKDNMKNLHTSSKKKSITKERSNF
LVQTIKSCLLKDHYIKEKKKEYYTNNTYCNDLHINDSTCSSYLLNSETKDAYCEYYNI
DHLCDINKKNMDINSKNELMGKYSKNELMGKTIKNELMGKYSKNELMGKYSKNELMGK
YSKNELMGKYSKNELMGKYSKNELMGKTIKNQVGVDTNIYHMNCDNDYNYDYPCDYNC
NNCNDTYHRLEYHNINKDNSFNIPPEKNKSYNNISEHIKINYPLLFEALACCHTLSKV
NNKIMGDVLEILMFNFTNCDMLINNNSFIIKEKKKNCSYDFQKIDGDKNIGANDERCH
LNNNLVSYNILKRFEFQSRLQRMSVIVKSTYGNNNDDNNDDDNNNDDDNNDDNNNDDN
NNDDNNDDNNNNNYYYNIFCKGSPEKIKELCLKSKIPNNYDEILNKYTKQGMRILSIS
YKRVKSKNINLLNVKRSFVESNLHFLGFLIFTNNMKKNAPDIIHNLQTSGCQCIMSTG
DNVLTSIHVAKKCGIINSNVESIIIGDVIPVVGKNNKQKKKLVWYNHKNDTYLKGHDK
TCIDNEFTSIQSQMNSDNICGDNICGDNIYGDNICGDNIYGDNINGDNINGDNIYGDN
INGDNINGDNINTYDNIYGDNYNLDYCPTEYHKCTYNNSILYRNNFLYKKENKKDKNY
KNISTLYEHRTNDIQFDKLCDILINKDPRNVNIVLTGKAFIFLKKKFYSFHLPYYEEC
KNIVHYIMKKKHKKIKNIINNHNSNLYYHYNIIDTFVKRMNKEYMCFNKLLYKIQQKL
LYNLIHNLYKKKKYMNNYYDIDEVHLIGNNNNNNNKNNSKEKKLPLKNKMKHIRKNES

NDNITFNTYTSNNIHLSKYKYVHHKNYYYPDSCTNLRKKKNSLFYNLKKYIYYEKKKY
LQHCLLKHDNYKKVELPRIKDINYSYQMESIKTRNFIHSLSEQFAFSNLILSFYIIKN
DDNNVYNKNYIYNKNYIYNKNSICNKNYICNKNYIYNKNNIYNKNNIYNKKNILTHAK
SVLLSGSSKKFLKFFSNIIRRHKLKEKKNKKNIKRYKMNHVNNTSKGHIILNMCTHGF
KKDYSSLKNKYRIVNNKRYMLKNDNVYDRHMYNLTDMYRGTQYGCSKKKNKNIYMNNN
NNILKNKINRFLEHLLVDKCKRNICHKYTDIKNIKLSIYEYILRTCTVYARMKPKDKS
DLILSLKKLPNNSYVGMCGDGANDCLALSCADIGISLCNNNESSICSSFTSNKLCLHS
IVHILIEGRASLVNSFQLFKFISLYSIMQCSQVLILYSISNKLTDNQYIFIDIVTILP
LSIFMCWTSASEKLSKNIPIGKLFSFPILISIYGQIIIQLFFVMISLVVLMNLSFYKY
DKNKVMKEKSDDTYLYKAQKYTLIYSLLFSKFVYVYIFKYKE
>CALM_PLAF7
ADKLTEEQISEFKEAFSLFDKDGDGTITTKELGTVMRSLGQNPTEAELQDMINEIDTD
GNGTIDFPEFLTLMARKLKDTDTEEELIEAFRVFDRDGDGYISADELRHVMTNLGEKL
TNEEVDEMIREADIDGDGQINYEEFVKMMIAK
>CALM_PLAFA
ADKLTEEQISEFKEAFSLFDKDGDGTITTKELGTVMRSLGQNPTEAELQDMINEIDTD
GNGTIDFPEFLTLMARKLKDTDTEEELIEAFRVFDRDGDGYISADELRHVMTNLGEKL
TNEEVDEMIREADIDGDGQINYEEFVKMMIAK
>CATC_PLAF7
MAKRIFSVSFLLVLLNVLHICIKFSVADLPTHVETKNLLGKWKILRTKTSPNLTTCGS
SQPNKNTYNVGITDYKKYLLENNYEFVSELNVILSDDYVLYGDIYNTQDNEHRSKWKV
LAVYDENKRVIGTWTTICDEGFEIKIGNETYAALMHYEPNGKCGPVSDEDSLDSNGDT
DCYTTSFSKIRYGWLDVENEKNEHLHGCFYAERIFDNVNEIKHLDSFTIDKDSQNVLQ
TFTYDTKLNNILNSNNMLYKFGNLQKPTFTKRNNTNVQFNSELNWHRMKHHGKKKPLK
KSMLDASRQTYACPCNANEVVDNVINKGDSDNPVSPTLIQLNNNLKNTTQTGNKDTNE
MDLENYEDTLNSPKRELEINELPKNFTWGDPWNKNTREYEVTNQLLCGSCYIASQLYA
FKRRIEVALTKKLDRKYLNNFDDQLSIQTVLSCSFYDQGCNGGFPYLVSKLAKLQGIP
LNVYFPYSATEETCPYNISKHPNDMNGSAKLREINAIFNSNNNMSTYNNINNDHHQLG
VYANTASSQEQHGISEENRWYAKDFNYVGGCYGCNQCNGEKIMMNEIYRNGPIVSSFE
ASPDFYDYADGVYFVEDFPHARRCTIEPKNDGVYNITGWDRVNHAIVLLGWGEEEING
KLYKYWIGRNSWGNGWGKEGYFKILRGQNFSGIESQSLFIEPDFSRGAGKILLEKMQK
ELGN
>CDAT_PLAF7
MKVRKMHYPLFSFTEYLSCYIILYFFVFLPNYSYAININNYHNKNNLWNINNKWNNQQ
SNHFSNNRIGHFLWGSKIRKNPLASISTSITTKKLENQKKKTNKNESYSNVNDKYEHN
NNTAHFVQINYKNDQSNDTLSTYKNNNEMTVEGKPNNLASGEGKKEIHNTTQKILEKV
DYFKEREKITNSYNNNNNNNKVIVTHDNKNHTNYINKVENKQSDDKIKNLNNKYIKKF
KSHILQNDILGTISTMFWSGKKNNNGNVKKGIKNVPMDEKSYSPNDHDNNSNNSNNNN
NNDNNNSNNNNNNNNGGKNNSYYNEKTQKGVNDKETNFLLKALDSGKFPTYCLVENID
ENLDNFDIYMSKEKMDELNINDGATVLLKGKKKREMLGIARLDRSLKKHYVVISFAMK
KNLRLMHNDIIKIHPFMNAKRIRNVVLSPFSDTIPNLSREELEKAVIHPYLKNSYKPL
RVNSNIYIYYKNNKIEFKVLKIISEESENEEFGCIGEHSQLTLAEEYLKREDYEENND
DITYEDLGGMKKQLNKIRELIELPLKYPEIFMSIGISAPKGVLMHGIPGTGKTSIAKA
IANESNAYCYIINGPEIMSKHIGESEQKLRKIFKKASEKTPCIIFIDEIDSIANKRSK
SNNELEKRVVSQLLTLMDGLKKNNNVLVLAATNRPNSIDPALRRFGRFDREIEIPVPD
EQGRYEILLTKTKKMKLDPDVNLRKIAKECHGYVGADLAQLCFEAAIQCIKEHIHFLD
LDEEDFIEFMKISVDEDKKNMGNEPYGSSHTNNSNYINHLTESSNKLSYTNMFPLNRK
NTLLQNDKNEMNKDSSYDKKTDALDNYKNDSTIDMEKKKNKKKSNFFFSNDDEETKNK
NKTNVNQKKKKNPNDKLDKNERRIPAYILNKLTIKAKHFQHALNICNPSSLRERQVQI
PTVTWNDIGGMNEVKEQLKETILYPLEYKHLYNKFNSNYNKGILLYGPPGCGKTLLAK
AIANECKANFISVKGPELLTMWFGESEANVRDLFDKARAASPCIIFFDEIDSLAKERN

SNTNNDASDRVINQILTEIDGINEKKTIFIIAATNRPDILDKALTRPGRLDKLIYISL
PDLKSRYSIFKAILKNTPLNEDVDIHDMAKRTEGFSGADITNLCQSAVNEAIKETIHL
LNIRKKEQEEQRKKNKNSFKIDDTDTYDPVPTLSKKHFDLAFKNARISIQPEDVLKYE
KFKEKLSLQDF
>CDC2H_PLAF7
MEKYHGLEKIGEGTYGVVYKAQNNYGETFALKKIRLEKEDEGIPSTTIREISILKELK
HSNIVKLYDVIHTKKRLVLVFEHLDQDLKKLLDVCEGGLESVTAKSFLLQLLNGIAYC
HDRRVLHRDLKPQNLLINREGELKIADFGLARAFGIPVRKYTHEVVTLWYRAPDVLMG
SKKYSTTIDIWSVGCIFAEMVNGTPLFPGVSEADQLMRIFRILGTPNSKNWPNVTELP
KYDPNFTVYEPLPWESFLKGLDESGIDLLSKMLKLDPNQRITAKQALEHAYFKENN
>CDC2H_PLAFK
MEKYHGLEKIGEGTYGVVYKAQNNYGETFALKKIRLEKEDEGIPSTTIREISILKELK
HSNIVKLYDVIHTKKRLVLVFEHLDQDLKKLLDVCEGGLESVTAKSFLLQLLNGIAYC
HDRRVLHRDLKPQNLLINREGELKIADFGLARAFGIPVRKYTHEVVTLWYRAPDVLMG
SKKYSTTIDIWSVGCIFAEMVNGTPLFPGVSEADQLMRIFRILGTPNSKNWPNVTELP
KYDPNFTVYEPLPWESFLKGLDESGIDLLSKMLKLDPNQRITAKQALEHAYFKENN
>CDPK1_PLAF7
GCSQSSNVKDFKTRRSKFTNGNNYGKSGNNKNSEDLAINPGMYVRKKEGKIGESYFKV
RKLGSGAYGEVLLCREKHGHGEKAIKVIKKSQFDKMKYSITNKIECDDKIHEEIYNEI
SLLKSLDHPNIIKLFDVFEDKKYFYLVTEFYEGGELFEQIINRHKFDECDAANIMKQI
LSGICYLHKHNIVHRDIKPENILLENKHSLLNIKIVDFGLSSFFSKDNKLRDRLGTAY
YIAPEVLRKKYNEKCDVWSCGVILYILLCGYPPFGGQNDQDIIKKVEKGKYYFDFNDW
KNISEEAKELIKLMLTYDYNKRITAKEALNSKWIKKYANNINKSDQKTLCGALSNMRK
FEGSQKLAQAAILFIGSKLTTLEERKELTDIFKKLDKNGDGQLDKKELIEGYNILRSF
KNELGELKNVEEEVDNILKEVDFDKNGYIEYSEFISVCMDKQILFSEERLRDAFNLFD
TDKSGKITKEELANLFGLTSISEQMWNEVLGEADKNKDNMIDFDEFVNMMHKICDNKS
S
>CDPK1_PLAFK
GCSQSSNVKDFKTRRSKFTNGNNYGKSGNNKNSEDLAINPGMYVRKKEGKIGESYFKV
RKLGSGAYGEVLLCREKHGHGEKAIKVIKKSQFDKMKYSITNKIECDDKIHEEIYNEI
SLLKSLDHPNIIKLFDVFEDKKYFYLVTEFYEGGELFEQIINRHKFDECDAANIMKQI
LSGICYLHKHNIVHRDIKPENILLENKHSLLNIKIVDFGLSSFFSKDNKLRDRLGTAY
YIAPEVLRKKYNEKCDVWSCGVILYILLCGYPPFGGQNDQDIIKKVEKGKYYFDFNDW
KNISEEAKELIKLMLTYDYNKRITAKEALNSKWIKKYANNINKSDQKTLCGALSNMRK
FEGSQKLAQAAILFIGSKLTTLEERKELTDIFKKLDKNGDGQLDKKELIEGYNILRSF
KNELGELKNVEEEVDNILKEVDFDKNGYIEYSEFISVCMDKQILFSEERLRDAFNLFD
TDKSGKITKEELANLFGLTSISEQMWNEVLGEADKNKDNMIDFDEFVNMMHKICDNKS
S
>CDPK2_PLAF7
GNHLSVNKLKRKKKKKSFLNIYGKNTNENTSKQSNDYKYDINTSCISREGTTTLERKN
LILCHSGKLEDKYIIDEKLGQGTYGCVYKGIDKVTNQLYAIKEEKKDRLKNINRFFQE
IEIMKKLDHPNIVKLYETYENDNYIYLIMELCSGRELFDSIIENGSFTEKNAATIMKQ
IFSAIFYLHSLNIVHRDLKPENFLFQSENKDSLLKIIDFGLSKNLGTGEFTTTKAGTP
YYVAPQVLDGKYDKKCDIWSSGVIMYTLLCGYPPFYGDTDNEVLKKVKKGEFCFYEND
WGSISSDAKNLITKLLTYNPNERCTIEEALNHPWITQMTKSHEHVELSSTLLKNLKNF
KKENELKKIALTIIAKHLCDVEINNLRNIFIALDVDNSGTLSSQEILDGLKKIGYQKI
PPDIHQVLRDIDSNASGQIHYTDFLAATIDKQTYLKKEVCLIPFKFFDIDGNGKISVE
ELKRIFGRDDIENPLIDKAIDSLLQEVDLNGDGEVNKNYNHEKG
>CDPK2_PLAFK
GNHLSVNKLKRKKKKKSFLNIYGKNTNENTSKQSNDYKYDINTSCISREGTTTLERKN
LILCHSGKLEDKYIIDEKLGQGTYGCVYKGIDKVTNQLYAIKEEKKDRLKNINRFFQE

IEIMKKLDHPNIVKLYETYENDNYIYLIMELCSGRELFDSIIENGSFTEKNAATIMKQ
IFSAIFYLHSLNIVHRDLKPENFLFQSENKDSLLKIIDFGLSKNLGTGEFTTTKAGTP
YYVAPQVLDGKYDKKCDIWSSGVIMYTLLCGYPPFYGDTDNEVLKKVKKGEFCFYEND
WGSISSDAKNLITKLLTYNPNERCTIEEALNHPWITQMTKSHEHVELSSTLLKNLKNF
KKENELKKIALTIIAKHLCDVEINNLRNIFIALDVDNSGTLSSQEILDGLKKIGYQKI
PPDIHQVLRDIDSNASGQIHYTDFLAATIDKQTYLKKEVCLIPFKFFDIDGNGKISVE
ELKRIFGRDDIENPLIDKAIDSLLQEVDLNGDGEIDFHEFMLMMSKKK
>CDPK3_PLAF7
MNDLIIKNNKKGSCDVIIKYKCKKSDENIKRRKSSHKYIKNKSVVLGRSIMTNKKEKL
KGALKYKGSKKEIKICNKKSMIKNDKDENTTLKSMKSDNFKFSRRGFILSFTGNLEDF
YNLSKEPLGKGTYGCVYKATDKLLKISRAVKVVSKKKLKNIPRFRQEIDIMKNLDHPN
VVKLLETFEDSNQIYLVMELCTGGELFDKIVKKGCFVETFASFIMKQIFSVLNYLHIR
NICHRDIKPENFLFYDMTPESLIKIIDFGLASYFTHNNYEMKTKAGTPYYVAPQVLTG
SYNYKCDMWSSGVLFYILLCGYPPFFGESDHEILSMVKKGKYQFKGKEWNNISEEAKD
LIKRCLTMDADKRICASEALQHPWFKKKKYAFNMDMKMDIHVLENFKNYGLLLKFQKL
AMTIIAQQSNDYDVEKLKSTFLVLDEDGKGYITKEQLKKGLEKDGLKLPYNFDLLLDQ
IDSDGSGKIDYTEFIAAALDRKQLSKKLIYCAFRVFDVDNDGEITTAELAHILYNGNK
KGNITQRDVNRVKRMIRDVDKNNDGKIDFHEFSEMMKLKF
>CDPK4_PLAF7
GQEVSSVNNTKNEHHKTNKKSLKGGNERHEMKESSVGISKKIVENSFNNSKLRPGMFI
QNSNVVFNEQYKGIKILGKGSFGEVILSRDKHTGHEYAIKVISKKHVKRKTDKESLLR
EVELLKMLDHINIMKLYEFFEDNNYYYLVSDVYTGGELFDEIISRKRFYEIDAARIIK
QILSGITYMHKNNVVHRDLKPENILLETKNKEDMIIKIIDFGLSTHFEYSKKMKDKIG
TAYYIAPDVLHGTYDEKCDIWSCGVILYILLSGCPPFNGSNEYDILKKVEAGKYTFDL
PQFKKISDKAKDLIKKMLMYTSAVRISARDALEHEWIKMMTSKDNLNIDIPSLELSIA
NIRQFQSTQKLAQAALLYMGSKLTTIDETKELTKIFKKMDKNGDGQLDRNELIIGYKE
LLKLKGEDTSDLDNAAIEYEVDQILNSIDLDQNGYIEYSEFLTVSIDRKLLLSTERLE
KAFKLFDKDGSGKISANELAQLFGLSDVSSECWKTVLKEVDQNNDGEIDFKEFRDMLV
KLCNY
>CH60_PLAFG
MRMKRIHILFVVIFLLCLRYGYSIKKKRSPNNKNRLFINKRLKYINSKIISRRKENYV
KMKMTENKVKGKDIIYGNECRNELLKGILTVSDVVKLTLGPRGRNVLLEKEYGSPLII
NDGVTIAKNISLKDRKKNNGVKLMQESTNISNDKAGDGTSSTALMTATITKKGIEQVN
RNHNPIPIQRGIQLASKMIIEKIKSLSTPIKTYKDILNIATIASNNDVHMGQIIANAY
DKLGKNAAIILDDNADINDKLEFTEGYNFDRGIINPYLLYNENKDYIEYSNVSTLITD
QNIDNIQSILPILEIFAKNKQPLCIIADDFSNEVLQTLIINKLKGAIKVLCIVTNSKY
ISADVGLDLNNLHNNMSSFDNNYLSLLGSANTLIVKKDRTSLITKEEYKKEIDERINV
LKKEYEETTSKYDKEKLNERIAALSGGIAKILIGGNSETEQKERKFKYEDATNAVKSA
IDIGYVPGGGVTYLEIIKSNFIQEIHKKIEEDLQISSNNDEKKYLELIGNLESEMELQ
KMGANIVVSSLDVITKQIADNAGVNGDNVVKIILNSKDKYGFGYDVNTNKFVNMVEKG
IIDSTNVIISVIKNSCSIASMVLTTECMMVDHEKKDKGILDSSINSPNYLSKHRRTYK
HKLHDDEDTDEDDEEDEDDEDDEDDLDDDDYDDEDEEDEEDEEDEDDEDDEDSMNDEY
NYDE
>COX1_PLAFA
MVLNRYSLITNCNHKTLGLYYLWFSFLFGSYGFLLSVILRTELYSSSLRIIAQENVNL
YNMIFTIHGIIMIFFNIMPGLFGGFGNYFLPILCGSPELAYPRINSISLLLQPIAFVL
VILSTAAEFGGGTGWTLYPPLSTSLMSLSPVAVDVIIFGLLVSGVASIMSSLNFITTV
MHLRAKGLTLGILSVSTWSLIITSGMLLLTLPVLTGGVLMLLSDLHFNTLFFDPTFAG
DPILYQHLFWFFGHPEVYILILPAFGVISHVISTNYCRNLFGNQSMILAMGCIAVLGS
LVWVHHMYTTGLEVDTRAYFTSTTILISIPTGTKVFNWICTYMSSNFGMIHSSSLLSL
LFICTFTFGGTTGVILGNAAIDVALHDTYYVIAHFHFVLSIGAIIGLFTTVSAFQDNF

FGKNLRENSIVILWSMLFFVGVILTFLPMHFLGFNVMPRRIPDYPDALNGWNMICSIG
STMTLFGLLIFK
>CRA_PLAFA
MKILSVFFLALFFIIFNKESLAEKTNKGTGSGVSSKKKNKKGSGEPLIDVHDLISDMI
KKEEELVEVNKRKSKYKLATSVLAGLLGVVSTVLLGGVGLVLYNTEKGRHPFKIGSSD
PADNANPDADSESNGEPNADPQVTAQDVTPEQPQGDDNNLVSGPEH
>CSP_PLAFA
MMRKLAILSVSSFLFVEALFQEYQCYGSSSNTRVLNELNYDNAGTNLYNELEMNYYGK
QENWYSLKKNSRSLGENDDGNNNNGDNGREGKDEDKRDGNNEDNEKLRKPKHKKLKQP
GDGNPDPNANPNVDPNANPNVDPNANPNVDPNANPNANPNANPNANPNANPNANPNAN
PNANPNANPNANPNANPNANPNANPNANPNANPNVDPNANPNANPNANPNANPNANPN
ANPNANPNANPNANPNANPNANPNANPNANPNANPNANPNANPNANPNANPNANPNKN
NQGNGQGHNMPNDPNRNVDENANANNAVKNNNNEEPSDKHIEQYLKKIKNSISTEWSP
CSVTCGNGIQVRIKPGSANKPKDELDYENDIEKKICKMEKCSSVFNVVNSSIGLIMVL
SFLFLN
>CSP_PLAFL
EALFQEYQCYGSSSNTRVLNELNYDNAGTNLYNELEMNYYGKQENWYSLKKNSRSLGE
NDDGNNNNGDNGREGKDEDKRDGNNEDNEKLRKPKHKKLKQPADGNPDPNANPNVDPN
ANPNVDPNANPNVDPNANPNANPNANPNANPNANPNANPNANPNANPNANPNANPNAN
PNVDPNANPNANPNANPNANPNANPNANPNANPNANPNANPNANPNANPNANPNANPN
ANPNANPNANPNANPNANPNANPNANPNANPNKNNQGNGQGHNMPNDPNRNVDE
NANGNNAVKNNNNEEPSDQHIEKYL
>CSP_PLAFO
MMRKLAILSVSSFLFVEALFQEYQCYGSSSNTRVLNELNYDNAGTNLYNELEMNYYGK
QENWYSLKKNSRSLGENDDGNNEDNEKLRKPKHKKLKQPADGNPDPNANPNVDPNANP
NVDPNANPNVDPNANPNANPNANPNANPNANPNANPNANPNANPNANPNANPNANPNA
NPNANPNANPNANPNANPNANPNVDPNANPNANPNANPNANPNANPNANPNANPNANP
NANPNANPNANPNANPNANPNANPNANPNANPNANPNANPNKNNQGNGQGHNMPNDPN
RNVDENANANSAVKNNNNEEPSDKHIKEYLNKIQNSLSTEWSPCSVTCGNGIQVRIKP
GSANKPKDELDYANDIEKKICKMEKCSSVFNVVNSSIGLIMVLSFLFLN
>CSP_PLAFT
MMRKLAILSVSSFLFVEALFQEYQCYGSSSNTRVLNELNYDNAGTNLYNELEMNYYGK
QENWYSLKKNSRSLGENDDGNNNNGDNNREGKDEDKRDGNNEDNETLRKPKHKKLKQP
GDGNPDPNANPNVDPNANPNVDPNANPNVDPNANPNANPNANPNANPNANPNANPNAN
PNANPNANPNANPNANPNANPNANPNANPNANPNANPNANPNANPNANPNANPNANPN
ANPNANPNANPNANPNANPNANPNANPNANPNANPNANPNANPNANPNANPNANPNAN
PNANPNANPNKNNQGNGQGHNMPNDPNRNVDENANANNAVKNNNNEEPSDKHIEQYLK
KIQNSLSTEWSPCSVTCGNGIQVRIKPGSANKPKDELDYENDIEKKICKMEKCSSVFN
VVNSSIGLIMVLSFLFLN
>CSP_PLAFW
MMRKLAILSVSSFLFVEALFQEYQCYGSSSNTRVLNELNYDNAGTNLYNELEMNYYGK
QENWYSLKKNSRSLGENDDGDNDNGNNNNGNNNNGDNGREGKDEDKRDGNNEDNEKLR
KPKHKKLKQPGDGNPDPNANPNVDPNANPNVDPNANPNANPNANPNANPNANPNANPN
ANPNANPNANPNANPNANPNANPNANPNANPNANPNVDPNANPNANPNANPNANPNAN
PNANPNANPNANPNANPNANPNANPNANPNANPNANPNANPNANPNANPNANPNANPN
ANPNANPNANPNANPNANPNANPNKNNQGNGQGHNMPNDPNRNVDENANANNAV
KNNNNEEPSDKHIEQYLKKIQNSLSTEWSPCSVTCGNGIQVRIKPGSADKPKDQLDYE
NDIEKKICKMEKCSSVFNVVNSSIGLIMVLSFLFLN
>CYB_PLAFA
MNFYSINLVKAHLINYPCPLNINFLWNYGFLLGIIFFIQIITGVFLASRYTPDVSYAY
YSIQHILRELWSGWCFRYMHATGASLVFLLTYLHILRGLNYSYMYLPLSWISGLILFM

IFIVTAFVGYVLPWGQMSYWGATVITNLLSSIPVAVIWICGGYTVSDPTIKRFFVLHF
ILPFIGLCIVFIHIFFLHLHGSTNPLGYDTALKIPFYPNLLSLDVKGFNNVIILFLIQ
SLFGIIPLSHPDNAIVVNTYVTPSQIVPEWYFLPFYAMLKTVPSKPAGLVIVLLSLQL
LFLLAEQRSLTTIIQFKMIFGARDYSVPIIWFMCAFYALLWIGCQLPQDIFILYGRLF
IVLFFCSGLFVLVHYRRTHYDYSSQANI
>CYSP_PLAFA
MVAIKEMKELAFARPSLVETLNKKKFLKKKEKRTFVLSIYAFITFIIFCIGILYFTN
KSSAHNNNNNKNEHSLKKEEIELLRVLLEKYKKQKDGILNESSNEEDEEKYTLNSETY
NNKNNVSNIKNDSIKSKKEEYINLERILLEKYKKFINENNEENRKELSNILHKLLEIN
KLILREEKDDKKVYLINDNYDEKGALEIGMNEEMKYKKEDPINNIKYASKFFKFMKEH
NKVYKNIDEQMRKFEIFKINYISIKNHNKLNKNAMYKKKVNQFSDYSEEELKEYFKTL
LHVPNHMIEKYSKPFENHLKDNILISEFYTNGKRNEKDIFSKVPEILDYREKGIVHEP
KDQGLCGSCWAFASVGNIESVFAKKNKNILSFSEQEVVDCSKDNFGCDGGHPFYSFLY
VLQNELCLGDEYKYKAKDDMFCLNYRCKRKVSLSSIGAVKENQLILALNEVGPLSVNV
GVNNDFVAYSEGVYNGTCSEELNHSVLLVGYGQVEKTKLNYNNKIQTYNTKENSNQPD
DNIIYYWIIKNSWSKKWGENGFMRLSRNKNGDNVFCGIGEEVFYPIL
>DPOD_PLAFK
MEELKTCPFTNVIPYGLLYDKLKKEKNNDVPENYVIEEFDKLLKNYERPNVYDEIGNA
TFKNDEDLITFQIDLDYTVENIFKNMIYNESGSNNSILNDIYMPYRILLSKDKNYVSV
PIIRIYSLRKDGCSVLINVHNFFPYFYVEKPDDFDNEDLIKLEMLMNENLNLNSQYKI
YEKKILKIEIVKTESLMYFKKNGKKDFLKITVLLPKMVPSLKKYFEGIVHVNNKSIGG
IVYEANLPFILRYIIDHKITGSSWINCKKGHYYIRNKNKKISNCTFEIDISYEHVEPI
TLENEYQQIPKLRILSFDIECIKLDGKGFPEAKNDPIIQISSILYFQGEPIDNCTKFI
FTLLECASIPGSNVIWFNDEKTLLEAWNEFIIRIDPDFLTGYNIINFDLPYILNRGTA
LNLKKLKFLGRIKNVASTVKDSSFSSKQFGTHETKEINIFGRIQFDVYDLIKRDYKLK
SYTLNYVSFEFLKEQKEDVHYSIMNDLQNESPESRKRIATYCIKDGVLPLRLIDKLLF
IYNYVEMARVTGTPFVYLLTRGQQIKVTSQLYRKCKELNYVIPSTYMKVNTNEKYEGA
TVLEPIKGYYIEPISTLDFASLYPSIMIAHNLCYSTLIKSNHEVSDLQNDDITTIQGK
NNLKFVKKNVKKGILPLIVEELIEARKKVKLLIKNEKNNITKMVLNGRQLALKISANS
VYGYTGASSGGQLPCLEVAVSITTLGRSMIEKTKERVESFYCKSNGYEHNSTVIYGDT
DSVMVKFGTNNIEEAMTLGKDAAERISKEFLSPIKLEFEKVYCPYLLLNKKRYAGLLY
TNPNKHDKMDCKGIETVRRDFCILIQQMMETVLNKLLIEKNLNSAIEYTKSKIKELLT
NNIDMSLLVVTKSLGKTDYETRLPHVELAKKLKQRDSATAPNVGDRVSYIIVKGVKGQ
AQYERAEDPLYVLDNNLAIDYNHYLDAIKSPLSRIFEVIMQNSDSLFSGDHTRHKTIL
TSSQTALSKFLKKSVRCIGCNSSIKKPPLCNHCKENKEFSIYMQKIKDFKNKQNEFFQ
LWTECQRCQGNLHVDVICMNRDCPIFYRRAKIKKDIANLQEQVTSLRMDW
>DRTS_PLAFK
MMEQVCDVFDIYAICACCKVESKNEGKKNEVFNNYTFRGLGNKGVLPWKCNSLDMKYF
RAVTTYVNESKYEKLKYKRCKYLNKETVDNVNDMPNSKKLQNVVVMGRTNWESIPKKF
KPLSNRINVILSRTLKKEDFDEDVYIINKVEDLIVLLGKLNYYKCFIIGGSVVYQEFL
EKKLIKKIYFTRINSTYECDVFFPEINENEYQIISVSDVYTSNNTTLDFIIYKKTNNK
MLNEQNCIKGEEKNNDMPLKNDDKDTCHMKKLTEFYKNVDKYKINYENDDDDEEEDDF
VYFNFNKEKEEKNKNSIHPNDFQIYNSLKYKYHPEYQYLNIIYDIMMNGNKQSDRTGV
GVLSKFGYIMKFDLSQYFPLLTTKKLFLRGIIEELLWFIRGETNGNTLLNKNVRIWEA
NGTREFLDNRKLFHREVNDLGPIYGFQWRHFGAEYTNMYDNYENKGVDQLKNIINLIK
NDPTSRRILLCAWNVKDLDQMALPPCHILCQFYVFDGKLSCIMYQRSCDLGLGVPFNI
ASYSIFTHMIAQVCNLQPAQFIHVLGNAHVYNNHIDSLKIQLNRIPYPFPTLKLNPDI
KNIEDFTISDFTIQNYVHHEKISMDMAA
>EBA1_PLAFC
MKCNISIYFFASFFVLYFAKARNEYDIKENEKFLDVYKEKFNELDKKKYGNVQKTDKK
IFTFIENKLDILNNSKFNKRWKSYGTPDNIDKNMSLINKHNNEEMFNNNYQSFLSTSS

LIKQNKYVPINAVRVSRILSFLDSRINNGRNTSSNNEVLSNCREKRKGMKWDCKKKND
RSNYVCIPDRRIQLCIVNLSIIKTYTKETMKDHFIEASKKESQLLLKKNDNKYNSKFC
NDLKNSFLDYGHLAMGNDMDFGGYSTKAENKIQEVFKGAHGEISEHKIKNFRKEWWNE
FREKLWEAMLSEHKNNINNCKNIPQEELQITQWIKEWHGEFLLERDNRSKLPKSKCKN
NTLYEACEKECIDPCMKYRDWIIRSKFEWHTLSKEYETQKVPKENAENYLIKISENKN
DAKVSLLLNNCDAEYSKYCDCKHTTTLVKSVLNGNDNTIKEKREHIDLDDFSKFGCDK
NSVDTNTKVWECKNPYILSTKDVCVPPRRQELCLGNIDRIYDKNLLMIKEHILAIAIY
ESRILKRKYKNKDDKEVCKIINKTFADIRDIIGGTDYWNDLSNRKLVGKINTNSKYVH
RNKKNDKLFRDEWWKVIKKDVWNVISWVFKDKTVCKEDDIENIPQFFRWFSEWGDDYC
QDKTKMIETLKVECKEKPCEDDNCKSKCNSYKEWISKKKEEYNKQAKQYQEYQKGNNY
KMYSEFKSIKPEVYLKKYSEKCSNLNFEDEFKEELHSDYKNKCTMCPEVKDVPISIIR
NNEQTSQEAVPEENTEIAHRTETPSISEGPKGNEQKERDDDSLSKISVSPENSRPETD
AKDTSNLLKLKGDVDISMPKAVIGSSPNDNINVTEQGDNISGVNSKPLSDDVRPDKKE
LEDQNSDESEETVVNHISKSPSINNGDDSGSGSATVSESSSSNTGLSIDDDRNGDTFV
RTQDTANTEDVIRKENADKDEDEKGADEERHSTSESLSSPEEKMLTDNEGGNSLNHEE
VKEHTSNSDNVQQSGGIVNMNVEKELKDTLENPSSSLDEGKAHEELSEPNLSSDQDMS
NTPGPLDNTSEETTERISNNEYKVNEREDERTLTKEYEDIVLKSHMNRESDDGELYDE
NSDLSTVNDESEDAEAKMKGNDTSEMSHNSSQHIESDQQKNDMKTVGDLGTTHVQNEI
SVPVTGEIDEKLRESKESKIHKAEEERLSHTDIHKINPEDRNSNTLHLKDIRNEENER
HLTNQNINISQERDLQKHGFHTMNNLHGDGVSERSQINHSHHGNRQDRGGNSGNVLNM
RSNNNNFNNIPSRYNLYDKKLDLDLYENRNDSTTKELIKKLAEINKCENEISVKYCDH
MIHEEIPLKTCTKEKTRNLCCAVSDYCMSYFTYDSEEYYNCTKREFDDPSYTCFRKEA
FSSMIFKFLITNKIYYYFYTYKTAKVTIKKINFSLIFFFFFSF
>EF1A_PLAFK
MGKEKTHINLVVIGHVDSGKSTTTGHIIYKLGGIDRRTIEKFEKESAEMGKGSFKYAW
VLDKLKAERERGITIDIALWKFETPRYFFTVIDAPGHKDFIKNMITGTSQADVALLVV
PADVGGFDGAFSKEGQTKEHVLLAFTLGVKQIVVGVNKMDTVKYSEDRYEEIKKEVKD
YLKKVGYQADKVDFIPISGFEGDNLIEKSDKTPWYKGRTLIEALDTMQPPKRPYDKPL
RIPLQGVYKIGGIGTVPVGRVETGILKAGMVLNFAPSAVVSECKSVEMHKEVLEEARP
GDNIGFNVKNVSVKEIKRGYVASDTKNEPAKGCSKFTAQVIILNHPGEIKNGYTPLLD
CHTSHISCKFLNIDSKIDKRSGKVVEENPKAIKSGDSALVSLEPKKPMVVETFTEYPP
LGRFAIRDMRQTIAVGIINQLKRKNLGAVTAKAPAKK
>EFTU_PLAFA
MNNKLFLRNKQHINLGTIGHVDHGKTTLTTAISYLLNLQGLSKKYNYSDIDSAPEEKI
RGITINTTHIEYETLTKHCAHIDCPGHSDYIKNMIIGATQMDIAILVISIIDGIMPQT
YEHLLLIKQIGIKNIIIFLNKEDLCDDVELIDFIKLEVNELLIKYNFDLNYIHILTGS
ALNVINIIQKNKDYELIKSNIWIQKLNNLIQIIDNIIIPTRKINDYFLMSIEDVFSIT
GRGTVVTGKIEQGCINLNDEIEILKFEKSSPNLTTVIGLEMFKKQLTQAQSGDNVGIL
LRNIQKKDIKRGMILATPNKLKVYKSFIAETYILTKEEGGRHKPFNIGYKPQFFIRTV
DVTGEIKNIYLNENVQKVAIPGDKITLHIELKHYIVLTLNMKFSIREGGKTIGAGIIT
EIKN
>ENO_PLAF7
MAHVITRINAREILDSRGNPTVEVDLETNLGIFRAAVPSGASTGIYEALELRDNDKSR
YLGKGVQKAIKNINEIIAPKLIGMNCTEQKKIDNLMVEELDGSKNEWGWSKSKLGANA
ILAISMAVCRAGAAANKVSLYKYLAQLAGKKSDQMVLPVPCLNVINGGSHAGNKLSFQ
EFMIVPVGAPSFKEALRYGAEVYHTLKSEIKKKYGIDATNVGDEGGFAPNILNANEAL
DLLVTAIKSAGYEGKVKIAMDVAASEFYNSENKTYDLDFKTPNNDKSLVKTGAQLVDL
YIDLVKKYPIVSIEDPFDQDDWENYAKLTAAIGKDVQIVGDDLLVTNPTRITKALEKN
ACNALLLKVNQIGSITEAIEACLLSQKNNWGVMVSHRSGETEDVFIADLVVALRTGQI
KTGAPCRSERNAKYNQLLRIEESLGNNAVFAGEKFRLQLN
>ENO_PLAFA

MAHVITRINAREILDSRGNPTVEVDLETNLGIFRAAVPSGASTGIYEALELRDNDKSR
YLGKGVQKAIKNINEIIAPKLIGMNCTEQKKIDNLMVEELDGSKNEWGWSKSKLGANA
ILAISMAVCRAGAAPNKVSLYKYLAQLAGKKSDQMVLPVPCLNVINGGSHAGNKLSFQ
EFMIVPVGAPSFKEALRYGAEVYHTLKSEIKKKYGIDATNVGDEGGFAPNILNANEAL
DLLVTAIKSAGYEGKVKIAMDVAASEFYNSENKTYDLDFKTPNNDKSLVKTGAQLVDL
YIDLVKKYPIVSIEDPFDQDDWENYAKLTAAIGKDVQIVGDDLLVTNPTRITKALEKN
ACNALLLKVNQIGSITEAIEACLLSQKNNWGVMVSHRSGETEDVFIADLVVALRTGQI
KTGAPCRSERNAKYNQLLRIEESLGNNAVFAGEKFRLQLN
>ENO_PLAFG
MAHVITRINAREILDSRGNPTVEVDLETNLGIFRAAVPSGASTGIYEALELRDNDKSR
YLGKGVQKAIKNINEIIAPKLIGMNCTEQKKIDNLMVEELDGSKNEWGWSKSKLGANA
ILAISMAVCRAGAAANKVSLYKYLAQLAGKKSDQMVLPVPCLNVINGGSHAGNKLSFQ
EFMIVPVGAPSFKEALRYGAEVYHTLKSEIKKKYGIDATNVGDEGGFAPNILNANEAL
DLLVTAIKSAGYEGKVKIAMDVAASEFYNSENKTYDLDFKTPNNDKSLVKTGAQLVDL
YIDLVKKYPIVSIEDPFDQDDWENYAKLTAAIGKDVQIVGDDLLVTNPTRITKALEKN
ACNALPLKVNQIGSITEAIEACLLSQKNNWGVMVSHRSGETEDVFIADLVVALRTGQI
KTGAPCRSERNAKYNQLLRIEESLGNNAVFAGEKFRLQLN
>ERD2_PLAF7
MNIFRLIGDILHLVSMYILIMKLKKSKNCIGISCRMQELYLIVFLCRYIDLFFVFVSF
YNTVMKITFILTIAYTIYLIRLKLPISQTYNRKVDNFKSEKYLIPPCLVLSLLTCKTY
NLYNILWSFSIWLESVAILPQLVLLEKQREVENITSHYVITMGLYRAFYILNWIYRYF
FDDKPYINVVGWIGGLIQTLLYIDFFYYFALAKWYGKKLVLPFNGEV
>ERD2_PLAFA
MNIFRLIGDILHLVSMYILIMKLKKSKNCIGISCRMQELYLIVFLCRYIDLFFVFVSF
YNTVMKITFILTIAYTIYLIRLKLPISQTYNRKVDNFKSEKYLIPPCLVLSLLTCKTY
NLYNILWSFSIWLESVAILPQLVLLEKQREVENITSHYVITMGLYRAFYILNWIYRYF
FDDKPYINVVGWIGGLIQTLLYIDFFYYFALAKWYGKKLVLPFNGEV
>EXP1_PLAFA
MKILSVFFLVLFFIIFNKESLAEKTNKETGSGVSSKKKNKKGSGEPLIDVHDLISDMI
KKEEELVEVNKRKSKYKLATSVLAGLLGVVSTVLLGGVGLVLYNTEKGRHPFKIGSSD
PADNANPDADSESNGEPNADPQVTAQDVTPEQPQGDDNNLVSGPEH
>FIRA_PLAFF
VTGSCVVTGSCVVTDSCVVTGSCGNTRTVTTQESVTTQEPVTIEEPVTTQEPVTIEEP
VTTQEPVTIEEPVTTQEPVTTQEPVTTQEPVTTQEPVTTQEPVTVEEHIDEKKGSEGD
NISLSSLSEETEEKSHTKKKKSSWLKFGRGNKNDKKSKNEKKPSLESVKQNADEQKEQ
PTDSQISVNAQDSVTIQEPTATQEPPTTQELTATQEPTTTQETVTEQEPTTTQETVTA
QEPITTQEPVTAQEPVTTQELIATQEPSTTQEHADEKKASEGDNISLSRLSEETEEKS
HTKKKSSWLKFGRGNKNDKK
>G6PI_PLAFA
MNMEITNLKSYKELVTLSAEEKTKDLKDYLNDKNRSESLIKKFKNFYMDLSRQRYSEK
TLNKLVEYAEEVELKKKVEKTFMGEKVNMTENRSVLHTALRIPIEKINTHKIIIDNKN
VLEDVHGVLKKIEKYSDDIRNGVIKTCKNTKFKNVICIGIGGSYLGTEFVYEAMKYYY
YNMELNKNEKDQVNNFNNNYDQDNVFNVRFLANVDPNDVNRAIQNLDQYDTLVIIISK
TFTTAETMLNARSIKKWLSLKIKDDENLSKHMVAVSTNLKLTDEFGISRDNVFEFWDW
VGGRFSVTSSVGILPLSIAFGYKNMRNFLNGCHDMDEHFLHADLKENIPVLLALTSFY
NSHFFDYKNVAILPYFQNLLKFSAHIQQLSMESNGKSVDRNNQPIHYNTCQVYFGEPG
TNGQHSFYQLIHQGQVIPVELIGFKHSHFPIKFDKEVVSNHDELMTNFFAQADALAIG
KTYEQVKEENEKNKMSPELLTHKVFNGNRPSTLLLFDELNFYTCGLLLSLYESRIVAE
GFLLNINSFDQWGVELGKVLAKEVRNYFNDTRNQKKSDNTYNFNESTKNFIKLLLVQI
KKKKKINTNLK
>GARP_PLAFF

MNVLFLSYNICILFFVVCTLNFSTKCFSNGLLKNQNILNKSFDSITGRLLNETELEKN
KDDNSKSETLLKEEKDEKDDVPTTSNDNLKNAHNNNEISSSTDPTNIINVNDKDNENS
VDKKKDKKEKKHKKDKKEKKEKKDKKEKKDKKEKKHKKEKKHKKDKKKKENSEVMSLY
KTGQHKPKNATEHGEENLDEEMVSEINNNAQGGLLLSSPYQYREQGGCGIISSVHETS
NDTKDNDKENISEDKKEDHQQEEMLKTLDKKERKQKEKEMKEQEKIEKKKKKQEEKEK
KKQEKERKKQEKKERKQKEKEMKKQKKIEKERKKKEEKEKKKKKHDKENEETMQQPDQ
TSEETNNEIMVPLPSPLTDVTTPEEHKEGEHKEEEHKEGEHKEGEHKEEEHKEEEHKK
EEHKSKEHKSKGKKDKGKKDKGKHKKAKKEKVKKHVVKNVIEDEDKDGVEIINLEDKE
ACEEQHITVESRPLSQPQCKLIDEPEQLTLMDKSKVEEKNLSIQEQLIGTIGRVNVVP
RRDNHKKKMAKIEEAELQKQKHVDKEEDKKEESKEVQEESKEVQEDEEEVEEDEEEEE
EEEEEEEEEEEEEEEEEEEEEEEDEDEEDEDDAEEDEDDAEEDEDDAEEDDDEEDD
DEEDDDEDEDEDEEDEEEEEEEEEESEKKIKRNLRKNAKI
>GBPH_PLAFB
MRISKASNIESTGVSNCKNFNSKNCSKYSLMEVQNKNEKKRSLTSFHAKNITLIFGII
YVALLGVYICASQYKQAADYSFRESRVLAEGKSTSKKNAKTALRKTKQTTLTSADPEG
QIMKAWAADPEYRKHLNVLYQILNNTDPNDELETSADPEGQIMKAYAADPEYRKHLNV
LYQILNNTDPNDEVESSADPEGQIMKAYAADPEYRKHVNVLYQILNNTDPNDELETSA
DPEGQIMKAYAADPEYRKHVNVLYQILNHTDSSEVETSADPEGQIMKAYAADPEYRKH
VNVLYQILNHTDSSEVETSADPEGQIMKAYAADPEYRKHVNVLYQILNNTDPNDELET
SADPEGQIMKAYAADPEYRKHVNVLYQILNNTDPNDELETSADPEGQIMKAYAADPEY
RKHVNVLYQILNNTDPNDESS
>GBP_PLAF7
MRLSKVSDIKSTGVSNYKNFNSKNSSKYSLMEVSKKNEKKNSLGAFHSKKILLIFGII
YVVLLNAYICGDKYEKAVDYGFRESRILAEGEDTCARKEKTTLRKSKQKTSTRTVATQ
TKKDEENKSVVTEEQKVESDSEKQKRTKKVVKKQINIGDTENQKEGKNVKKVIKKEKK
KEESGKPEENKHANEASKKQEPKASKVSQKPSTSTRSNNEVKIRAASNQETLTSADPE
GQIMREYAADPEYRKHLEIFYKILTNTDPNDEVERRNADNKEDLTSADPEGQIMREYA
SDPEYRKHLEIFYKILTNTDPNDDVERRNADNKEDLTSADPEGQIMREYAADPEYRKH
LEVFHKILTNTDPNDEVERRNADNKEDLTSADPEGQIMREYAADPEYRKHLEVFHKIL
TNTDPNDEVERRNADNKELTSSDPEGQIMREYAADPEYRKHLEVFHKILTNTDPNDEV
ERRNADNKEDLTSADPEGQIMREYAADPEYRKHLEVFHKILTNTDPNDEVERRNADNK
EDLTSADPEGQIMREYAADPEYRKHLEIFHKILTNTDPNDEVERRNADNKEDLTSADP
EGQIMREYAADPEYRKHLEIFYKILTNTDPNDEVERRNADNKEELTSSDPEGQIMREY
AADPEYRKHLEIFHKILTNTDPNDEVERRNADNKEDLTSADPEGQIMREYAADPEYRK
HLEIFYKILTNTDPNDEVERRNADNKEDLTSADPEGQIMREYASDPEYRKHLEIFYKI
LTNTDPNDDVERRNADNKEDLTSADPEGQIMREYAADPEYRKHLEVFHKILTNTDPND
EVERQNADNNEA
>GBP_PLAFG
MRLSKVSDIKSTGVSNYKNFNSKNSSKYSLMEVSKKNEKKNSLGAFHSKKILLIFGII
YVVLLNAYICGDKYEKAVDYGFRESRILAEGEDTCARKEKTTLRKSKQKTSTRTVATQ
TKKDEENKSVVTEEQKVESDSEKQKRTKKVVKKQINIGDTENQKEGKNVKKVIKKEKK
KEESGKPEENKHANEASKKKEPKASKVSQKPSTSTRSNNEVKIRAASNQETLTSADPE
GQIMREYAADPEYRKHLEIFYKILTNTDPNDEVERRNADNKEDLTSADPEGQIMREYA
SDPEYRKHLEIFYKILTNTDPNDDVERRNADNKEDLTSADPEGQIMREYAADPEYRKH
LEVFHKILTNTDPNDEVERRNADNKEDLTSADPEGQIMREYAADPEYRKHLEIFHKIL
TNTDPNDEVERRNADNKEDLTSADPEGQIMREYAADPEYRKHLEVFHKILTNTDPNDE
VERRNADNKELTSSDPEGQIMREYAADPEYRKHLEIFHKILTNTDPNDEVERRNADNK
EDLTSADPEGQIMREYAADPEYRKHLEIFYKILTNTDPNDEVERRNADNKEELTSSDP
EGQIMREYAADPEYRKHLEIFHKILTNTDPNDEVERRNADNKEDLTSADPEGQIMREY
AADPEYRKHLEIFYKILTNTDPNDEVERRNADNKEDLTSADPEGQIMREYASDPEYRK
HLEIFYKILTNTDPNDDVERRNADNKEDLTSADPEGQIMREYAADPEYRKHLEIFHKI

LTNTDPNDEVERQNADNNEA
>GRP78_PLAFA
EFFNGKEPNRGINPDEAVAYGAAIQAGIILGEELQDVVLLDVTPLTLGIETVGGIMTQ
LIKRNTVIPTKKSQTFSTYQDNQPAVLIQVFEGERALTKDNHLLGKFELSGIPPAQRG
VPKIEVTFTVDKNGILHVEAEDKGTGKSRGITITNDKGRLSKEQIEKMINDAEKFADE
DKNLREKVEAKNNLDNYIQSMKATVEDKDKLADKIEKEDKNTILSAVKDAEDWLNNNS
NADSEALKQKLKDLEAVCQPIIVKLYGQPGGPSPQPSGDEDVDSDEL
>GRP78_PLAFO
MNQIRPYILLLIVSLLKFISAVDSNIEGPVIGIDLGTTYSCVGVFKNGRVEILNNELG
NRITPSYVSFVDGERKVGEAAKLEATVHPTQTVFDVKRLIGRKFDDQEVVKDRSLLPY
EIVNNQGKPNIKVQIKDKDTTFAPEQISAMVLEKMKEIAQSFLGKPVKNAVVTVPAYF
NDAQRQATKDAGTIAGLNIVRIIINQPTAAALAYALDKKEETSILVYDLGGGTFDVSI
LVIDNGVFEVYATAGNTHLGGEDFDQRVMDYFIKMFKKKNNIDLRTDKRAIQKLRKEV
EIAKRNLSVVHSTQIEIEDIVEGHNFSETLTRAKFEELNDDLFRETLEPVKKVLDDAK
YEKSKIDEIVLVGGSTRIPKIQQIIKEFEFFNGKEPNRGINPDEAVAYGAAIQAGIIL
GEELQDVVLLDVTPLTLGIETVGGIMTQLIKRNTVIPTKKSQTFSTYQDNQPAVLIQV
FEGERALTKDNHLLGKFELSGIPPAQRGVPKIEVTFTVDKNGILHVEAEDKGTGKSRG
ITITNDKGRLSKEQIEKMINDAEKFADEDKNLREKVEAKNKLDNYIQSMKATVEDKDK
LADKIEKEDKNTILSAVKDAEDWLNNNSNADSEALKQKLKDLEAVCQPIIVKLYGQPG
GPSPQPSGDEDVDSDEL
>GSHR_PLAF7
VYDLIVIGGGSGGMAAARRAARHNAKVALVEKSRLGGTCVNVGCVPKKIMFNAASVHD
ILENSRHYGFDTKFSFNLPLLVERRDKYIQRLNNIYRQNLSKDKVDLYEGTASFLSEN
RILIKGTKDNNNKDNGPLNEEILEGRNILIAVGNKPVFPPVKGIENTISSDEFFNIKE
SKKIGIVGSGYIAVELINVIKRLGIDSYIFARGNRILRKFDESVINVLENDMKKNNIN
IVTFADVVEIKKVSDKNLSIHLSDGRIYEHFDHVIYCVGRSPDTENLNLGKLNVETNN
NYIVVDENQRTSVNNIYAVGDCCMVKKSKEIEDLNLLKLYNEETYLNKKENVTEDIFY
NVQLTPVAINAGRLLADRLFLKKTRKTNYKLIPTVIFSHPPIGTIGLSEEAAIQIYGK
ENVKIYESKFTNLFFSVYDIEPELKEKTYLKLVCVGKDELIKGLHIIGLNADEIVQGF
AVALKMNATKKDFDETIPIHPTAAEEFLTLQPWMK
>GSHR_PLAFK
VYDLIVIGGGSGGMAAARRAARHNAKVALVEKSRLGGTCVNVGCVPKKIMFNAASVHD
ILENSRHYGFDTKFSFNLPLLVERRDKYIQRLNNIYRQNLSKDKVDLYEGTASFLSEN
RILIKGTKDNNNKDNGPLNEEILEGRNILIAVGNKPVFPPVKGIENTISSDEFFNIKE
SKKIGIVGSGYIAVELINVIKRLGIDSYIFARGNRILRKFDESVINVLENDMKKNNIN
IVTFADVVEIKKVSDKNLSIHLSDGRIYEHFDHVIYCVGRSPDTENLKLEKLNVETNN
NYIVVDENQRTSVNNIYAVGDCCMVKKSKEIEDLNLLKLYNEERYLNKKENVTEDIFY
NVQLTPVAINAGRLLADRLFLKKTRKTNYKLIPTVIFSHPPIGTIGLSEEAAIQIYGK
ENVKIYESKFTNLFFSVYDIEPELKEKTYLKLVCVGKDELIKGLHIIGLNADEIVQGF
AVALKMNATKKDFDETIPIHPTAAEEFLTLQPWMK
>H2A_PLAFA
MSAKGKTGRKKASKGTSNSAKAGLQFPVGRIGRYLKKGKYAKRVGAGAPVYLAAVLEY
LCAEILELAGNAARDNKKSRITPRHIQLAVRNDEELNKFLAGVTFASGGVLPNIHNVL
LPKKSQLKAGTANQDY
>HGXR_PLAFG
MPIPNNPGAGENAFDPVFVNDDDGYDLDSFMIPAHYKKYLTKVLVPNGVIKNRIEKLA
YDIKKVYNNEEFHILCLLKGSRGFFTALLKHLSRIHNYSAVETSKPLFGEHYVRVKSY
CNDQSTGTLEIVSEDLSCLKGKHVLIVEDIIDTGKTLVKFCEYLKKFEIKTVAIACLF
IKRTPLWNGFKADFVGFSIPDHFVVGYSLDYNEIFRDLDHCCLVNDEGKKKYKATSL
>HGXR_PLAFK
MPIPNNPGAGENAFDPVFVKDDDGYDLDSFMIPAHYKKYLTKVLVPNGVIKNRIEKLA

YDIKKVYNNEEFHILCLLKGSRGFFTALLKHLSRIHNYSAVEMSKPLFGEHYVRVKSY
CNDQSTGTLEIVSEDLSCLKGKHVLIVEDIIDTGKTLVKFCEYLKKFEIKTVAIACLF
IKRTPLWNGFKADFVGFSIPDHFVVGYSLDYNEIFRDLDHCCLVNDEGKKKYKATSL
>HRP1_PLAFA
MVSFSKNKVLSAAVFASVLLLDNNNSAFNNNLCSKNAKGLNLNKRLLHETQAHVDDAH
HAHHVADAHHAHHAHHAADAHHAHHAADAHHAHHAADAHHAHHAADAHHAHHAADAHH
AHHAADAHHAHHAADAHHAHHAADAHHAHHAADAHHAHHAAYAHHAHHASDAHHAADA
HHAAYAHHAHHAADAHHAADAHHAAYAHHAHHAADAHHAADAHHATDAHHAHHAADAH
HATDAHHAADAHHAADAHHATDAHHAADAHHATDAHHAADAHHAADAHHATDSHHAHH
AADAHHAAAHHATDAHHAAAHHATDAHHAAAHHEAATHCLRH
>HRP2_PLAFA
MVSFSKNKVLSAAVFASVLLLDNNNSEFNNNLFSKNAKGLNSNKRLLHESQAHAGDAH
HAHHVADAHHAHHVADAHHAHHAANAHHAANAHHAANAHHAANAHHAANAHHAANAHH
AANAHHAANAHHAANAHHAANAHHAANAHHAANAHHAANAHHAADANHGFHFNLHDNN
SHTLHHAKANACFDDSHHDDAHHDGAHHDDAHHDGAHHDDAHHDGAHHDGAHHDGAHH
NATTHHLHH
>HRP3_PLAFS
MLNHRYHHYFHRHHHLNHHLYHRHHHHRHHHHRHHHRHQILHQNRHQIHQILLSLNNK
IMGYAIFLFLSILLHLVYLVIHRL
>HRP_PLAFF
MVSFSKNKILSAAVFASVLLLDNNNSEFNNNLFSKNAKGLNSNKRLLHESQAHAGDAH
HAHHVADAHHAHHAANAHHAANAHHAANAHHAANAHHAANAHHAANAHHAANAHHAAN
AHHAANAHHAANAHHAANAHHAANAHHAADANHGFHFNLHDNNSHTLHHAKANACFDD
SHHDDAHHDDAHHDGAHHDGAHHDGAHHDGAHHNATTHHLHH
>HSP70_PLAFA
MASAKGSKPNLPESNIAIGIDLGTTYSCVGVWRNENVDIIANDQGNRTTPSYVAFTDT
ERLIGDAAKNQVARNPENTVFDAKRLIGRKFTESSVQSDMKHWPFTVKSGVDEKPMIE
VTYQGEKKLFHPEEISSMVLQKMKENAEAFLGKSIKNAVITVPAYFNDSQRQATKDAG
TIAGLNVMRIINEPTAAAIAYGLHKKGKGEKNILIFDLGGGTFDVSLLTIEDGIFEVK
ATAGDTHLGGEDFDNRLVNFCVEDFKRKNRGKDLSKNSRALRRLRTQCERAKRTLSSS
TQATIEIDSLFEGIDYSVTVSRARFEELCIDYFRDTLIPVEKVLKDAMMDKKSVHEVV
LVGGSTRIPKIQTLIKEFFNGKEACRSINPDEAVAYGAAVQAAILSGDQSNAVQDLLL
LDVCSLSLGLETAGGVMTKLIERNTTIPAKKSQIFTTYADNQPGVLIQVYEGERALTK
DNNLLGKFHLDGIPPAPRKVPQIEVTFDIDANGILNVTAVEKSTGKQNHITITNDKGR
LSQDEIDRMVNDAEKYKAEDEENRKRIEARNSLENYCYGVKSSLEDQKIKEKLQPAEI
ETCMKTITTILEWLEKNQLAGKDEYEAKQKEAESVCAPIMSKIYQDAAGAAGGMPGGM
PGGMPGGMPSGMPGGMNFPGGMPGAGMPGNAPAGSGPTVEEVD
>HSP73_PLAFA
EFRETAGGVMTKLIERNTTIPTKKNQIFTTYADNQPGVLIQVYEGERAMTKDNNLLGK
FQLEGIPPAPRSVPQIEVTFDIDANGILNVTALDKGTGKQNQITITNDKGRLSKDDID
RMVNDAEKYKEEDEQNKNRIEARNNLENYCYNVKNTLQDENLKTKIPKDDSEKCMKTV
KSVLDWLEKNQTAETEEYNEKEKDTEF
>HSP90_PLAFP
KDFDGKKLKCCTKEGLDIHHSEEAKKDFETLKAEYEGLCKVIKDVLHKKVEKVVVGQR
ITDSPCVLVTSEFGWSANMERIMKAQALRDNSMTSYMLSKKIMEINARHPIISALKQK
ADADKSDKTVKYLIWLLFDTSLLTSGFALEEPTTFSKRIHRMIKLGLSIDEEENNDID
LPPLEETVDATDSKMEEVD
>HXK_PLAFA
MSEYDIAKNDVTYTKLDTIECDIPINEELSWRINKFVNQLRISYSTLEEFVDNFVYEL
KKGLEAHRKHPNLWIPHECSFKMLDSCIANIPTGQEKGTYYAIDFGGTNFRAVRASLD
GKGKIKRDQETYSLKFTGSYSHEKGLLDKHATASQLFDHFAERIKYIMGEFNDLDNKE

VKSVGFTFSFPCTSPSINCSILIDWTKGFETGRATNDPVEGRDVCKLMNDAFVRAAIP
AKVCCVLNDAVGTLMSCAYQKGRGTPPCYIGIILGTGSNGCYYEPEWKKYKYAGKIIN
IEFGNFDKDLPTSPIDLVMDWYSANRSRQLFEKMISGAYLGEIVRRFMVNVLQSACSK
KMWISDSFNSESGSVVLNDTSKNFEDSRKVAKAAWDMDFTDEQIYVLRKICEAVYNRS
AALARGTIAAIAKRIKIIEHSKFTCGVDGSLFVKNAWYCKRLQEHLKVILADKAENLI
IIPADDGSGKGAAITAAVIALNADIPQLP
>IPYR_PLAF7
MGSKLINVEGGNNQDDNKYNSNNVISINNKVNKNDYFIETNKELKINLNFQNNNIISN
IFSNINIYDKISNIFINNKKTYMLKYNNNINEENFFISYFEKKDDNFVPISPWHHIDL
KNDDGTYNMIVEITKYNYIKLEIQLREKFNVIKQDKKKGKLRYYHNSIYWNYGALPQT
YEYPKHIYQNKSKKNKEALLFTGDNDPLDILDIGSACLKIGQVVPVKILGAFTLIDEG
ELDWKIIAINKEDKHYEDINSLSDIEKYYPHTLSLLLEWFRSYKMADTKKLNLISKQL
YDKKESEDLIMKTHHYYLEFREDVKKLKEEHSKETIKEHDYVNAQNIQFNYDKLNNND
DEPMENNLLEDINITYYKSDSAYKPDLNIWTP
>ISPF_PLAF7
MFLKGYTSNVVLIILTFFILLTKEEKNIKNNISGYCFLNFGLKKNAIIKKREKQNLKL
FCYNGIRIGQGYDIHKIKVLDEEYNTYANNDFNKNEQSFKTLTLGGVKINNVLVLSHS
DGDIIYHSIVDSILGALGSLDIGTLFPDKDEKNKNKNSAIFLRYARLLIYKKNYDIGN
VDINVIAQVPKISNIRKNIIKNISTVLNIDESQISVKGKTHEKLGVIGEKKAIECFAN
ILLIPKNS
>ISPF_PLAFX
MFLKGYTSNVVLIILTFFILLTKEEKNIKNNISGYCFLNFGLKKNAIIKKREKQNLKL
FCYNGIRIGQGYDIHKIKVLDEEYNTYANNDFNKNEQSFKTLTLGGVKINNVLVLSHS
DGDIIYHSIVDSILGALGSLDIGTLFPDKDEKNKNKNSAIFLRYARLLIYKKNYDIGN
VDINVIAQVPKISNIRKNIIKNISTVLNIDESQISVKGKTHEKLGVIGEKKAIECFAN
ILLIPKNS
>KC1_PLAF4
MEIRVANKYALGKKLGSGSFGDIYVAKDIVTMEEFAVKLESTRSKHPQLLYESKLYKI
LGGGIGVPKVYWYGIEGDFTIMVLDLLGPSLEDLFTLCNRKFSLKTVRMTADQMLNRI
EYVHSKNFIHRDIKPDNFLIGRGKKVTLIHIIDFGLAKKYRDSRSHTSYPYKEGKNLT
GTARYASINTHLGIEQSRRDDIEALGYVLMYFLRGSLPWQGLKAISKKDKYDKIMEKK
ISTSVEVLCRNASFEFVTYLNYCRSLRFEDRPDYTYLRRLLKDLFIREGFTYDFLFDW
TCVYASEKDKKKMLENKNRFDQTADQEGRDQRNN
>KC1_PLAF7
MEIRVANKYALGKKLGSGSFGDIYVAKDIVTMEEFAVKLESTRSKHPQLLYESKLYKI
LGGGIGVPKVYWYGIEGDFTIMVLDLLGPSLEDLFTLCNRKFSLKTVLMTADQMLNRI
EYVHSKNFIHRDIKPDNFLIGRGKKVTLIHIIDFGLAKKYRDSRSHTHIPYKEGKNLT
GTARYASINTHLGIEQSRRDDIEALGYVLMYFLRGSLPWQGLKAISKKDKYDKIMEKK
ISTSVEVLCRNASFEFVTYLNYCRSLRFEDRPDYTYLRRLLKDLFIREGFTYDFLFDW
TCVYASEKDKKKMLENKNRFDQTADQEGRVKQN
>KNOB_PLAFA
MKSFKNKNTLRRKKAFPVFTKILLVSFLVWVLKCSNNCNNGNGSGDSFDFRNKRTLAQ
KQHEHHHHHHHQHQHQHQAPHQAHHHHHHGEVNHQAPQVHQQVHGQDQAHHHHHHHHH
QLQPQQLQGTVANPPSNEPVVKTQVFREARPGGGFKAYEEKYESKHYKLKENVVDGKK
DCDEKYEAANYAFSEECPYTVNDYSQENGPNIFALRKRFPLGMNDEDEEGKEALAIKD
KLPGGLDEYQNQLYGICNETCTTCGPAAIDYVPADAPNGYAYGGSAHDGSHGNLRGHG
NKGSEGYGYEAPYNPGFNGAPGSNGMQNYVPPHGAGYSAPYGVPHGAAHGSRYSSFSS
VNKYGKHGDEKHHSSKKHEGNDGEGEKKKKSKKHKDHDGEKKKSKKHKDNEDAESVKS
KKHKSHDCEKKKSKKHKDNEDAESVKSKKSVKEKGEKHNGKKPCSKKTNEENKNKEKT
NNLKSDGSK
>KNOB_PLAFD

AFSEECPYTVNDYSQENGPNIFALRKRFPLGMNDEDEEGKEALAIKDKLPGGLDEYQN
QLYGICNETCTTCGPAAIDYVPADAPNGYAYGGSAHDGSHGNLRGHDNKGSEGYGYEA
PYNPGFNGAPGSNGMQNYVPPHGAGYSAPYGVPHGAAHGSRYSSFSSVNKYGKHGDEK
HHSSKKHEGNDGEGEKKKKSKKHKDHDGEKKKSKKHKDNEDAESVKSKKHKSHDCEKK
KSKKHKDNEDAESVKSKKSVKEKGEKHNGKKPCSKKTNEEKKKKK
>KNOB_PLAFG
MKSFKNKNTLRRKKAFPVFTKILLVSFLVWVLKCSNNCNNGNGSGDSFDFRNKRTLAQ
KQHEHHHHHHHQHQHQHQAPHQAHHHHHHGEVNHQAPQVHQQVHGQDQAHHHHHHHHH
QLQPQQLQGTVANPPSNEPVVKTQVFREARPGGGFKAYEEKYESKHYKLKENVVDGKK
DCDEKYEAANYAFSEECPYTVNDYSQENGPNIFALRKRFPLGMNDEDEEGKEALAIKD
KLPGGLDEYQNQLYGICNETCTTCGPAAIDYVPADAPNGYAYGGSAHDGSHGNLRGHG
NKGSEGYGYEAPYNPGFNGAPGSNGMQNYVPPHGAGYSAPYGVPHGAAHGSRYSSFSS
VNKYGKHGDEKHHSSKKHEGNDGEGEKKKKSKKHKDHDGEKKKSKKHKDNEDAESVKS
KKHKSHDCEKKKSKKHKDNEDAESVKSKKSVKEKGEKHNGKKPCSKKTNEENKNKEKT
NNLKSDGSKAHEKKENETKNTAGENKKVDSTSADNKSTNAATPGAKDKTQGGKTDKTG
ASTNAATNKGQCAAEGATKGATKEASTSKEATKEASTSKGATKEASTTEGATKGASTT
AGSTTGATTGANAVQSKDGTADKNAANNGEQVMSRGQAQLQEAGKKKKKRGCCG
>KNOB_PLAFN
MKSFKNKNTLRRKKAFPVFTKILLVSFLVWVLKCSNNCNNGNGSGDSFDFRNKRTLAQ
KQHEHHHHHHHHHHHQHQAPHQAPHQAHHHHHHGEVNHQAPQVHQQVHGQDQAHHHHH
HHHHHLHPQQPQGTVANPPSNEPVVKTQVFREARPGGGFKAYEEKYESKHYKLKENVV
DGKKDCDEKYEAANYAFSEECPYTVNDYSQENGPNIFALRKRFPLGMNDEDEEGKEAL
AIKDKLPGGLDEYQNQLYGICNETCTTCGPAAIDYVPADAPNGYAYGGSAHDGSHGNL
RGHDNKGSEGYGYEAPYNPGFNGAPGSNGMQNYVHPWSGYSAPYGVPHGAAHGSRYSS
FSSVNKYGKHGDEKHHSSKKHEGNDGEGEKKKKSKKHKDHDGEKKKSKKHKDNEDAES
VKSKKHKSHDCEKKKSKKHKDNEDAESVKSKKVLKKREKSIMEKNHAAKKLTKKIKIK
KKTNNSKSDGSKAHEKKENETKNTAGENKKVDSTSADNKSTNAATPGAKDKTQGGKTD
KTGASTNAATNKGQCAAEGATKGATKEASTSKEATKEASTSKEATKEASTSKEATKEA
STSKGATKEASTTEGATKGASTTAGSTTGATTGANAVQSKDETADKNAANNGEQVMSR
GQAQLQEAGKKKKKRGCCG
>KPK2_PLAFK
MEKRYQQLFKGKRIDFPLATGAASHVSLTYDEKKNPYLLCWTYIYQEKPEFTVPLKGC
RIINNITEIGPCIHIITSNEEYQFQCRSKEEFDEMSQFFNMLGYPILGFKNVYVLNKK
IGKGSFSTAYIGTNILYGNRVVVKEVDKSKVKESNVYTEIEVLRKVMHKYIIKLISAY
EQEGFVYLVLEYLKGGELFEYLNNNGPYTEQVAKKAMKRVLIALEALHSNGVVHRDLK
MENLMLENPNDPSSLKIIDFGLASFLNSPSMNMRCGSPGYVAPEILKCASYGTKVDIF
SLGVILFNILCGYPPFRGNNVKEIFKKNMRCHISFNTKHWINKSESVKEIILWMCCKN
PDDRCTALQALGHQWFLPKLTDMHMTANINELKRNEAIVHKSNDQQDMCKKCKHFNNT
QNDDIYNNNNNNNNQLDPNKNHKNNYNDYKNYFDTMLKIDDKYSENLIKDKTSMDSISL
NKKDYDAYLVHSNEHDTVVLHGKCQTTKNSSSLLSYKCSRRSPPQN
>LDH_PLAFA
LLVYDNLLLNDKNEMNKDSSYDKKTNALDNYKNDSTIDMEKK
>LDH_PLAFD
MAPKAKIVLVGSGMIGGVMATLIVQKNLGDVVLFDIVKNMPHGKALDTSHTNVMAYSN
CKVSGSNTYDDLAGADVVIVTAGFTKAPGKSDKEWNRDDLLPLNNKIMIEIGGHIKKN
CPNAFIIVVTNPVDVMVQLLHQHSGVPKNKIIGLGGVLDTSRLKYYISQKLNVCPRDV
NAHIVGAHGNKMVLLKRYITVGGIPLQEFINNKLISDAELEAIFDRTVNTALEIVNLH
ASPYVAPAAAIIEMAESYLKDLKKVLICSTLLEGQYGHSDIFGGTPVVLGANGVEQVI
ELQLNSEEKAKFDEAIAETKRMKALA
>MDR_PLAFF
MGKEQKEKKDGNLSIKEEVEKELNKKSTAELFRKIKNEKISFFLPFKCLPAQHRKLLF

ISFVCAVLSGGTLPFFISVFGVILKNMNLGDDINPIILSLVSIGLVQFILSMISSYCM
DVITSKILKTLKLEYLRSVFYQDGQFHDNNPGSKLRSDLDFYLEQVSSGIGTKFITIF
TYASSFLGLYIWSLIKNARLTLCITCVFPLIYVCGVICNKKVKLNKKTSLLYNNNTMS
IIEEALMGIRTVASYCGEKTILNKFNLSETFYSKYILKANFVEALHIGLINGLILVSY
AFGFWYGTRIIINSATNQYPNNDFNGASVISILLGVLISMFMLTIILPNITEYMKALE
ATNSLYEIINRKPLVENNDDGETLPNIKKIEFKNVRFHYDTRKDVEIYKDLSFTLKEG
KTYAFVGESGCGKSTILKLIERLYDPTEGDIIVNDSHNLKDINLKWWRSKIGVVSQDP
LLFSNSIKNNIKYSLYSLKDLEAMENYYEENTNDTYENKNFSLISNSMTSNELLEMKK
EYQTIKDSDVVDVSKKVLIHDFVSSLPDKYDTLVGSNASKLSGGQKQRISIARAIMRN
PKILILDEATSSLDNKSEYLVQKTINNLKGNENRITIIIAHRLSTIRYANTIFVLSNR
ERSDNNNNNNNDDNNNNNNNNNNKINNEGSYIIEQGTHDSLMKNKNGIYHLMINNQKI
SSNKSSNNGNDNGSDNKSSAYKDSDTGNDADNMNSLSIHENENISNNRNCKNTAENEK
EEKVPFFKRMFRRKKKAPNNLRIIYKEIFSYKKDVTIIFFSILVAGGLYPVFALLYAR
YVSTLFDFANLEYNSNKYSIYILLIAIAMFISETLKNYYNNKIGEKVEKTMKRRLFEN
ILYQEMSFFDQDKNTPGVLSAHINRDVHLLKTGLVNNIVIFSHFIMLFLVSMVMSFYF
CPIVAAVLTFIYFINMRVFAVRARLTKSKEIEKKENMSSGVFAFSSDDEMFKDPSFLI
QEAFYNMHTVINYGLEDYFCNLIEKAIDYKNKGQKRRIIVNAALWGFSQSAQLFINSF
AYWFGSFLIKRGTILVDDFMKSLFTFIFTGSYAGKLMSLKGDSENAKLSFEKYYPLMI
RKSNIDVRDDGGIRINKNLIKGKVDIKDVNFRYISRPNVPIYKNLSFTCDSKKTTAIV
GETGSGKSTFMNLLLRFYDLKNDHIILKNDMTNFQDYQNNNNNSLVLKNVNEFSNQSG
SAEDYTVFNNNGEILLDDINICDYNLRDLRNLFSIVSQEPMLFNMSIYENIKFGREDA
TLEDVKRVSKFAAIDEFIESLPNKYDTNVGPYGKSLSGGQKQRIAIARALLREPKILL
LDEATSSLDSNSEKLIEKTIVDIKDKADKTIITIAHRIASIKRSDKIVVFNNPDRNGT
FVQSHGTHDELLSAQDGIYKKYVKLAK
>MSA2_PLAF1
MKVIKTLSIINFFIFVTFNIKNESKYSNTFINNAYNMSIRRSMTESKTPTPTGAGAGA
SGSAGSGDGASGSASGSASGSASGSAGASGSASGSAGASGSASGSAGAEGSPSTPATT
TTTTTTNDAEASTSTSSENPNHNNAKTNPKGNGGVQKPNQANKETQNNSNVQQDSQTK
SNVPPTQDADTKSPTAQPEQAENSAPTAEQTESPELQSAPENKGTGQHGHMHGSRNNH
PQNTSDSQKECTDGNKENCGAATSLLNNSSNIASINKFVVLISATLVLSFAIFI
>MSA2_PLAF2
MKVIKTLSIINFFIFVTFNIKNESKYSNTFINNAYNMSIRRSMAESKPPTGDGAVASA
GNGAVASAGNGAVASAGNGAVASAGNGAGNGAGNGAGNGAGNGAGNGAGNGAGNGAGN
GAGNGAGNGAGNGAGNGAGNGAGNGAGNGAVASAGNGAGNGAVASAGNGAVAERSSST
PATTTTTTTTNDAEASTSTSSENSNHNNAETNPKGNGEVQPNQANKETQNNSNVQQDS
QTKSNVPRTQDADTKSPTAQPEQAENSAPTAEQTESPELQSAPENKGTGQHGHMHGSR
NNHPQNTSDSQKECTDGNKENCGAATSLLNNSSNIASINKFVVLISATLVLSFAIFI
>MSA2_PLAF6
MKVIKTLSIINFFIFVTFNIKNESKYSNTFINNAYNMSIRRSMAESKPPTGTGASGSA
GSGAGASGSAGSGDGAVASARNGANPGADAEGSSSTPATTTTTTTTTTTTTNDAEAS
TSTSSENPNHNNAETNPKGKGEVQKSNQANKETQNNSNVQQDSQTKSNVPPTQDADTK
SPTAQPEQAENSAPTAEQTESPELQSAPENKGTGQHGHMHGSRNNHPQNTSDSQKECT
DGNKENCGAVTSLLSNSSNIASINKFVVLISAKLVLSFAIFI
>MSA2_PLAF7
MKVIKTLSIINFFIFVTFNIKNESKYSNTFINNAYNMSIRRSMAESKPSTGAGGSAGG
SAGGSAGGSAGGSAGGSAGSGDGNGADAEGSSSTPATTTTTKTTTTTTTTNDAEASTS
TSSENPNHKNAETNPKGKGEVQEPNQANKETQNNSNVQQDSQTKSNVPPTQDADTKSP
TAQPEQAENSAPTAEQTESPELQSAPENKGTGQHGHMHGSRNNHPQNTSDSQKECTDG
NKENCGAATSLLNNSSNIASINKFVVLISATLVLSFAIFI
>MSA2_PLAF8
MKVIKTLSIINFFIFVTFNIKNESKYSNTFINNAYNMSIRRSMAESNPSTGAGGSGSA

GGSGSAGGSGSAGGSGSAGGSGSAGSGDGNGANPGADAERSPSTPATTTTTTTTNDAE
ASTSTSSENPNHNNAETNPKGKGEVQKPNQANKETQNNSNVQQDSQTKSNVPPTQDAD
TKSPTAQPEQAENSAPIAEQTESPELQSAPENKGTGQHGHMHGSRNNHPQNTSDSQKE
CTDGNKENCGAAPSLLSNSSNIASINKFVVLISATLVLSFAIFI
>MSA2_PLAF9
MKVIKTLSIINFFIFVTFNIKNESKYSNTFINNAYNMSIRRSMEESKPPTGAVAGSGA
GAGSGAGAVAGSGAGAVAGSGAGAVAGSGAGAVAGSGAGAVAGSGAGAVAGSGAGNGA
NPGADAERSPSTPATTTTTTTTNDAEASTSTSSENRNHNNAETNPKGKGEVQKPNQAN
KETQNNSNVQQDSQTKSNVPRTQDADTKSPTAQPEQAENSAPTAEQTESPELQSAPEN
KGTGQHGHMHGSRNNHPQNTSDSQKECTDGNKENCGAATSLLNNSSNIASINKFVVLI
SATLVLSFAIFI
>MSA2_PLAFC
MKVIKTLSIINFFIFVTFNIKNESKYSNTFINNAYNMSIRRSMAESKPPTGTGGSGSA
GSGAGASAGNGANPGADAERSPSTPATPATPATTTTTTTTNDAEASTSTSSENPNHKN
AETNPKGKGEVQKPNQANKETQNNSNVQQDSQTKSNVPPTQDADTKSPTAQPEQAENS
APTAEQTESPELQSAPENKGTGQHGHMHGSRNNHPQNTSDSQKECTDGNKENCGAATS
LLNNSSNIASINKFVVLISATLVLSFAIFI
>MSA2_PLAFF
MKVIKTLSIINFFIFVTFNIKNESKYSNTFINNAYNMSIRRSMANEGSNTNSVGANAP
NADTIASGSQRSTNSASTSTTNNGESQTTTPTAADTIASGSQRSTNSASTSTTNNGES
QTTTPTAADTPTATESISPSPPITTTESSKFWQCTNKTDGKGEESEKQNELNESTEEG
PKAPQEPQTAENENPAAPENKGTGQHGHMHGSRNNHPQNTSDSQKECTDGNKENCGAA
TSLLSNSSNIASINKFVVLISATLVLSFAIFI
>MSA2_PLAFG
MKVIKTLSIINFFIFVTFNIKNESKYSNTFINNAYNMSIRRSMTESNPPTGASGSAGG
SAGGSAGGSAGGSAGGSAGGSAGGSAGGSAGGSAGGSAGGSAGGSAGSGDGNGANPGA
DAERSPSTPATTTTTTTTNDAEASTSTSSENPNHNNAETNQANKETQNNSNVQQDSQT
KSNVPPTQDADTKSPTAQPEQAENSAPTAEQTESPELQSAPENKGTGQHGHMHGSRNN
HPQNTSDSQKECTDGNKENCGAATSLLNNSSNIASINKFVVLISATLVLSFAIFI
>MSA2_PLAFH
MKVIKTLSIINFFIFVTFNIKNESKYSNTFINNAYNMSIRRSMEESNPPTGASGRAGA
GASGRAGAGASGRAGAGAGAVASAGSGDGAVASAGNGANPGADAKRSTSTPATTTTTT
TTNDAEASTSTSSENPNHNNAKTNPKGKEVQEPNKANTETQNNSNVQQDSQTKSNVPP
TQDADTKSPTAQPEQAENSAPTAEQTESPELQSAPENKGTGQHGHMHGSRNNHPQNTS
DSQKECTDGNKENCGAATSLLNNSSNIASINKFVVLISATLVLSFAIFI
>MSA2_PLAFI
MKVIKTLSIINFFIFVTFNIKNESKYSNTFINNAYNMSIRRSMEESKPPTGAVAGSGA
GAGSGAGAVAGSGAGAVAGSGAGAVAGSGAGAVAGSGAGAVAGSGAVAGSGAGNGANP
GADAERSPSTPATTTTTTTTNDAEASTSTSSENRNHNNAETNPKGKGEVQKPNQANKE
TQNNSNVQQDSQTKSNVPRTQDADTKSPTAQPEQAENSAPTAEQTESPELQSAPENKG
TGQHGHMHGSRNNHPQNTSDSQKECTDGNKENCGAATSLLNNSSNIASINKFVVLISA
TLVLSFAIFI
>MSA2_PLAFJ
MKVIKTLSIINFFIFVTFNIKNESKYSNTFINNAYNMSIRRSMANEGSNTNSVGANAP
NADTIASGSQRSTNSASTSTTNNGESQTTTPTAADTIASGSQRSTNSASTSTTNNGES
QTTTPTAADTPTTTESNSPSPPITTTESSKFWQCTNKTDGKGEESEKQNELNESTEEG
PKAPQEPQTAENENPAAPENKGTGQHGHMHGSRNNHPQNTSDSQKECTDGNKENCGAA
TSLLNNSSNIASINKFVVLISATLVLSFAIFI
>MSA2_PLAFK
MKVIKTLSIINFFIFVTFNIKNGSKYSNTFINNAYNMSIRRSMANEGSNTKSVGANAP
KADTIASGSQSSTNSASTSTTNNGESQTTTPTAADTPTATESNSRSPPITTTESNSRS

PPITTTESNSRSPPITTTESNSRSPPITTTESNSRSPPITTTESSSSGNAPNKTDGKG
EESEKQNELNESTEEGPKAPQEPQTAENENPAAPENKGTGQHGHMHGSRNNHPQNTSD
SQKECTDGNKENCGAATSLLNNSSNIASINKFVVLISATLVLSFAIFI
>MSA2_PLAFZ
MKVIKTLSIINFFIFVTFNIKNESKYSNTFINNAYNMSIRRSMEESKPPTGAVAGSGA
GAGSGAGAVAGSGAGAVAGSGAGAVAGSGAGAVAGSGAGAVAGSGAVAGSGAGNGANP
GADAERGPSTPATTTTTTTTNDAEASTSTSSENRNHNNAETNPKGKGEVQKPNQANKE
TQNNSNVQQDSQTKSNVPRTQDADTKSPTAQPEQAENSAPTAEQTESPELQSAPENKG
TGQHGHMHGSRNNHPQNTSDSQKECTDGNKENCGAATSLLNNSSNIASINKFVVLISA
TLVLSFAIFI
>MSP1_PLAF3
MKIIFFLCSFLFFIINTQCVTHESYQELVKKLEALEDAVLTGYSLFQKEKMVLKDGAN
TQVVAKPADAVSTQSAKNPPGATVPSGTASTKGAIRSPGAANPSDDSSDSDAKSYADL
KHRVQNYLFTIKELKYPELFDLTNHMLTLCDNIHGFKYLIDGYEEINELLYKLNFYFD
LLRAKLNDVCANDYCQIPFNLKIRANELDVLKKLVFGYRKPLDFIKDNVGKMEDYIKK
NKTTIANINELIEGSKKTIDQNKNADNEEGKKKLYQAQYDLFIYNKQLQEAHNLISVL
EKRIDTLKKNENIKKLLEDIDKIKIDAEKPTTGVNQILSLRLEKESRHEEKIKEIAKT
IKFNIDRLFTDPLELEYYLREKNKKVDVTPKSQDPTKSVQIPKVPYPNGIVYPLPLTD
IHNSLAADNDKNSYGDLMNPHTKEKINEKIITDNKERKIFINNIKKQIDLEEKNINHT
KEQNKKLLEDYEKSKKDYEELLEKFYEMKFNNNFNKDVVDKIFSARYTYNVEKQRYNN
KFSSSNNSVYNVQKLKKALSYLEDYSLRKGISEKDFNHYYTLKTGLEADIKKLTEEIK
SSENKILEKNFKGLTHSANASLEVSDIVKLQVQKVLLIKKIEDLRKIELFLKNAQLKD
SIHVPNIYKPQNKPEPYYLIVLKKEVDKLKEFIPKVKDMLKKEQAVLSSITQPLVAAS
ETTEDGGHSTHTLSQSGETEVTEETEETVGHTTTVTITLPPKEVKVVENSIEHKSNDN
SQALTKTVYLKKLDEFLTKSYICHKYILVSNSSMDQKLLEVYNLTPEENELKSCDRLD
LLFNIQNNIPAMYSLYDSMNNDLQHLFFELYQKEMIYYLHKLKEENHIKKLLEEPKQI
TGTSSTSSPGNTTVNTAQSATHSNSQNQQSNASSTNTQNGVAVSSGPAVVEESHDPLT
VLSISNDLKGIVSLLNLGNKTKVPNPLTISTTEMEKFYENILKIMIPIFNDDIKQFVK
SNSKVITGLTETQKNALNDEIKKLKDTLQLSFDLYNKYKLKLDRLFNKKKELGQDKMQ
IKKLTLLKEQLESKLNSLNNPHNVLQNFSVFFNKKKEAEIAETENTLENTKILLKHYK
GLVKYYNGESSPLKTLSEVSIQTEDNYANLEKFRVLSKIDGKLNDNLHLGKKKLSFLS
SGLHHLITELKEVIKNKNYTGNSPSENNKKVNEALKSYENFLPEAKVTTVVTPPQPDV
TPSPLSVRVSGSSGSTKEETQIPTSGSLLTELQQVVQLQNYDEEDDSLVVLPIFGESE
DNDEYLDQVVTGEAISVTMDNILSGFENEYDVIYLKPLAGVYRSLKKQIEKNIFTFNL
NLNDILNSRLKKRKYFLDVLESDLMQFKHISSNEYIIEDSFKLLNSEQKNTLLKSYKY
IKESVENDIKFAQEGISYYEKVLAKYKDDLESIKKVIKEEKEFPSSPPTTPPSPAKTD
EQKKESKFLPFLTNIETLYNNLVNKIDDYLINLKAKINDCNVEKDEAHVKITKLSDLK
AIDDKIDLFKNPYDFEAIKKLINDDTKKDMLGKLLSTGLVQNFPNTIISKLIEGKFQD
MLNISQHQCVKKQCPQNSGCFRHLDEREECKCLLNYKQEGDKCVENPNPTCNENNGGC
DADAKCTEEDSGSNGKKITCECTKPDSYPLFDGIFCSSSNFLGISFLLILMLILYSFI
>MSP1_PLAFC
MKIIFFLCSFLFFIINTQCVTHESYQELVKKLEALEDAVLTGYGLFHKEKMILNEEEI
TTKGASAQSGTSGTSGTSGTSGTSGTSGTSAQSGTSGTSAQSGTSGTSAQSGTSGTSG
TSGTSPSSRSNTLPRSNTSSGASPPADASDSDAKSYADLKHRVRNYLFTIKELKYPEL
FDLTNHMLTLCDNIHGFKYLIDGYEEINELLYKLNFYFDLLRAKLNDVCANDYCQIPF
NLKIRANELDVLKKLVFGYRKPLDNIKDNVGKMEDYIKKNKTTIANINELIEGSKKTI
DQNKNADNEEGKKKLYQAQYDLSIYNKQLEEAHNLISVLEKRIDTLKKNENIKELLDK
INEIKNPPPANSGNTPNTLLDKNKKIEEHEEKIKEIAKTIKFNIDSLFTDPLELEYYL
REKNKKVDVTPKSQDPTKSVQIPKVPYPNGIVYPLPLTDIHNSLAADNDKNSYGDLMN
PDTKEKINEKIITDNKERKIFINNIKKQIDLEEKKINHTKEQNKKLLEDYEKSKKDYE
ELLEKFYEMKFNNNFDKDVVDKIFSARYTYNVEKQRYNNKFSSSNNSVYNVQKLKKAL

SYLEDYSLRKGISEKDFNHYYTLKTGLEADIKKLTEEIKSSENKILEKNFKGLTHSAN
ASLEVYDIVKLQVQKVLLIKKIEDLRKIELFLKNAQLKDSIHVPNIYKPQNKPEPYYL
IVLKKEVDKLKEFIPKVKDMLKKEQAVLSSITQPLVAASETTEDGGHSTHTLSQSGET
EVTEETEETEETVGHTTTVTITLPPKEVKVVENSIEHKSNDNSQALTKTVYLKKLDEF
LTKSYICHKYILVSNSSMDQKLLEVYNLTPEEENELKSCDPLDLLFNIQNNIPAMYSL
YDSMNNDLQHLFFELYQKEMIYYLHKLKEENHIKKLLEEQKQITGTSSTSSPGNTTVN
TAQSATHSNSQNQQSNASSTNTQNGVAVSSGPAVVEESHDPLTVLSISNDLKGIVSLL
NLGNKTKVPNPLTISTTEMEKFYENILKNNDTYFNDDIKQFVKSNSKVITGLTETQKN
ALNDEIKKLKDTLQLSFDLYNKYKLKLDRLFNKKKELGQDKMQIKKLTLLKEQLESKL
NSLNNPHNVLQNFSVFFNKKKEAEIAETENTLENTKILLKHYKGLVKYYNGESSPLKT
LSEVSIQTEDNYANLEKFRVLSKIDGKLNDNLHLGKKKLSFLSSGLHHLITELKEVIK
NKNYTGNSPSENNKKVNEALKSYENFLPEAKVTTVVTPPQPDVTPSPLSVRVSGSSGS
TKEETQIPTSGSLLTELQQVVQLQNYDEEDDSLVVLPIFGESEDNDEYLDQVVTGEAI
SVTMDNILSGFENEYDVIYLKPLAGVYRSLKKQIEKNIFTFNLNLNDILNSRLKKRKY
FLDVLESDLMQFKHISSNEYIIEDSFKLLNSEQKNTLLKSYKYIKESVENDIKFAQEG
ISYYEKVLAKYKDDLESIKKVIKEEKEKFPSSPPTTPPSPAKTDEQKKESKFLPFLTN
IETLYNNLVNKIDDYLINLKAKINDCNVEKDEAHVKITKLSDLKAIDDKIDLFKNHND
FEAIKKLINDDTKKDMLGKLLSTGLVQNFPNTIISKLIEGKFQDMLNISQHQCVKKQC
PENSGCFRHLDEREECKCLLNYKQEGDKCVENPNPTCNENNGGCDADAKCTEEDSGSN
GKKITCECTKPDSYPLFDGIFCSSSNFLGISFLLILMLILYSFI
>MSP1_PLAFD
MKIIFFLCSFLFFIINTQCVTHESYQELVKKLEALEDAVLTGYSLFQKEKMVLNEGTS
GTAVTTSTPGSKGSVASGGSGGSVASGGSVASGGSGNSRRTNPSDNSSDSDAKSYADL
KHRVRNYLLTIKELKYPQLFDLTNHMLTLCDNIHGFKYLIDGYEEINELLYKLNFYFD
LLRAKLNDVCANDYCQIPFNLKIRANELDVLKKLVFGYRKPLDNIKDNVGKMEDYIKK
K
>MSP1_PLAFF
MKIIFFLCSFLFFIINTQCVTHESYQELVKKLEALEDAVLTGYSLFQKEKMVLNEGTS
GTAVTTSTPGSSGSVTSGGSVASVASVASGGSGGSVASGGSGNSRRTNPSDNSSDSNT
KTYADLKHRVQNYLFTIKELKYPELFDLTNHMLTLSKNVDGFKYLIDGYEEINELLYK
LNFYYDLLRAKLNDACANSYCQIPFNLKIRANELDVLKKIVFGYRKPLDNIKDNVGKM
EDYIKKNKTTIANINELIEGSKKTIDQNKNADNEEGKKKLYQAQYNLFIYNKQLQEAH
NLISVLEKRIDTLKKNENIKKLLEDIDKIKTDAENPTTGSKPNPLPENKKKEVEGHEE
KIKEIAKTIKFNIDSLFTDPLELEYYLREKNKKVDVTPKSQDPTKSVQIPKVPYPNGI
VYPLPLTDIHNSLAADNDKNSYGDLMNPDTKEKINEKIITDNKERKIFINNIKKQIDL
EEKNINHTKEQNKKLLEDYEKSKKDYEELLEKFYEMKFNNNFDKDVVDKIFSARYTYN
VEKQRYNNKFSSSNNSVYNVQKLKKALSYLEDYSLRKGISEKDFNHYYTLKTGLEADI
KKLTEEIKSSENKILEKNFKGLTHSANASLEVSDIVKLQVQKVLLIKKIEDLRKIELF
LKNAQLKDSIHVPNIYKPQNKPEPYYLIVLKKEVDKLKEFIPKVKDMLKKEQAVLSSI
TQPLVAASETTEDGGHSTHTLSQSGETEVTEETEVTEETVGHTTTVTITLPPKEESAP
KEVKVVENSIEHKSNDNSQALTKTVYLKKLDEFLTKSYICHKYILVSNSSMDQKLLEV
YNLTPEEENELKSCDPLDLLFNIQNNIPAMYSLYDSMNIDLQHLFFELYQKEMIYYLH
KLKEENHIKKLLEEQKQITGTSSTSSPGNTTVNTAQSATHSNSQNQQSNASSTNTQNG
VAVSSGPAVVEESHDPLTVLSISNDLKGIVSLLNLGNKTKVPNPLTISTTEMEKFYEN
ILKNNDTYFNDDIKQFVKSNSKVITGLTETQKNALNDEIKKLKDTLQLSFDLYNKYKL
KLDRLFNKKKELGQDKMQIKKLTLLKEQLESKLNSLNNPHNVLQNFSVFFNKKKEAEI
AETENTLENTKILLKHYKGLVKYYNGESSPLKTLSEVSIQTEDNYANLEKFRALSKID
GKLNDNLHLGKKKLSFLSSGLHHLITELKEVIKNKNYTGNSPSENNKKVNEALKSYEN
FLPEAKVTTVVTPPQPDVTPSPLSVRVSGSSGSTKEETQIPTSGSLLTELQQVVQLQN
YDEEDDSLVVLPIFGESEDNDEYLDQVVTGEAISVTMDNILSGFENEYDVIYLKPLAG
VYRSLKKQIEKNIITFNLNLNDILNSRLKKRKYFLDVLESDLMQFKHISSNEYIIEDS

FKLLNSEQKNTLLKSYKYIKESVENDIKFAQEGISYYEKVLAKYKDDLESIKKVIKEE
KEKFPSSPPTTPPSPAKTDEQKKESKFLPFLTNIETLYNNLVNKIDDYLINLKAKIND
CNVEKDEAHVKITKLSDLKAIDDKIDLFKNTNDFEAIKKLINDDTKKDMLGKLLSTGL
VQNFPNTIISKLIEGKFQDMLNISQHQCVKKQCPENSGCFRHLDEREECKCLLNYKQE
GDKCVENPNPTCNENNGGCDADATCTEEDSGSSRKKITCECTKPDSYPLFDGIFCSSS
NFLGISFLLILMLILYSFI
>MSP1_PLAFK
MKIIFFLCSFLFFIINTQCVTHESYQELVKKLEALEDAVLTGYSLFHKEKMILNEEEI
TTKGASAQSGTSGTSGTSGPSGPSGTSPSSRSNTLPRSNTSSGASPPADASDSDAKSY
ADLKHRVRNYLLTIKELKYPQLFDLTNHMLTLCDNIHGFKYLIDGYEEINELLYKLNF
YFDLLRAKLNDVCANDYCQIPFNLKIRANELDVLKKLVFGYRKPLDNIKDNVGKMEDY
IKKNKKTIENINELIEESKKTIDKNKNATKEEEKKKLYQAQYDLSIYNKQLEEAHNLI
SVLEKRIDTLKKNENIKELLDKINEIKNPPPANSGNTPNTLLDKNKKIEEHEKEIKEI
AKTIKFNIDSLFTDPLELEYYLREKNKNIDISAKVETKESTEPNEYPNGVTYPLSYND
INNALNELNSFGDLINPFDYTKEPSKNIYTDNERKKFINEIKEKIKIEKKKIESDKKS
YEDRSKSLNDITKEYEKLLNEIYDSKFNNNIDLTNFEKMMGKRYSYKVEKLTHHNTFA
SYENSKHNLEKLTKALKYMEDYSLRNIVVEKELKYYKNLISKIENEIETLVENIKKDE
EQLFEKKITKDENKPDEKILEVSDIVKVQVQKVLLMNKIDELKKTQLILKNVELKHNI
HVPNSYKQENKQEPYYLIVLKKEIDKLKVFMPKVESLINEEKKNIKTEGQSDNSEPST
EGEITGQATTKPGQQAGSALEGDSVQAQAQEQKQAQPPVPVPVPEAKAQVPTPPAPVN
NKTENVSKLDYLEKLYEFLNTSYICHKYILVSHSTMNEKILKQYKITKEEESKLSSCD
PLDLLFNIQNNIPVMYSMFDSLNNSLSQLFMEIYEKEMVCNLYKLKDNDKIKNLLEEA
KKVSTSVKTLSSSSMQPLSLTPQDKPEVSANDDTSHSTNLNNSLKLFENILSLGKNKN
IYQELIGQKSSENFYEKILKDSDTFYNESFTNFVKSKADDINSLNDESKRKKLEEDIN
KLKKTLQLSFDLYNKYKLKLERLFDKKKTVGKYKMQIKKLTLLKEQLESKLNSLNNPK
HVLQNFSVFFNKKKEAEIAETENTLENTKILLKHYKGLVKYYNGESSPLKTLSEESIQ
TEDNYASLENFKVLSKLEGKLKDNLNLEKKKLSYLSSGLHHLIAELKEVIKNKNYTGN
SPSENNTDVNNALESYKKFLPEGTDVATVVSESGSDTLEQSQPKKPASTHVGAESNTI
TTSQNVDDEVDDVIIVPIFGESEEDYDDLGQVVTGEAVTPSVIDNILSKIENEYEVLY
LKPLAGVYRSLKKQLENNVMTFNVNVKDILNSRFNKRENFKNVLESDLIPYKDLTSSN
YVVKDPYKFLNKEKRDKFLSSYNYIKDSIDTDINFANDVLGYYKILSEKYKSDLDSIK
KYINDKQGENEKYLPFLNNIETLYKTVNDKIDLFVIHLEAKVLNYTYEKSNVEVKIKE
LNYLKTIQDKLADFKKNNNFVGIADLSTDYNHNNLLTKFLSTGMVFENLAKTVLSNLL
DGNLQGMLNISQHQCVKKQCPQNSGCFRHLDEREECKCLLNYKQEGDKCVENPNPTCN
ENNGGCDADAKCTEEDSGSNGKKITCECTKPDSYPLFDGIFCSSSNFLGISFLLILML
ILYSFI
>MSP1_PLAFM
MKIIFFLCSFLFFIINTQCVTHESYQELVKKLEALEDAVLTGYSLFQKEKMVLNEGTS
GTAVTTSTPGSSGSVTSGGSVASVASVASGGSGGSVASGGSGNSRRTNPSDNSSDSNT
KTYADLKHRVQNYLFTIKELKYPELFDLTNHMLTLSKNVDGFKYLIDGYEEINELLYK
LNFYYDLLRAKLNDACANSYCQIPFNLKIRANELDVLKKIVFGYRKPLDNIKDNVGKM
EDYIKKNKTTIANINELIEGSKKTIDQNKNADNEEGKKKLYQAQYNLFIYNKQLQEAH
NLISVLEKRIDTLKKNENIKKLLEDIDKIKTDAENPTTGSKPNPLPENKKKEVEGHEE
KIKEIAKTIKFNIDSLFTDPLELEYYLREKNKKVDVTPKSQDPTKSVQIPKVPYPNGI
VYPLPLTDIHNSLAADNDKNSYGDLMNPDTKEKINEKIITDNKERKIFINNIKKQIDL
EEKNINHTKEQNKKLLEDYEKSKKDYEELLEKFYEMKFNNNFDKDVVDKIFSARYTYN
VEKQRYNNKFSSSNNSVYNVQKLKKALSYLEDYSLRKGISEKDFNHYYTLKTGLEADI
KKLTEEIKSSENKILEKNFKGLTHSANASLEVSDIVKLQVQKVLLIKKIEDLRKIELF
LKNAQLKDSIHVPNIYKPQNKPEPYYLIVLKKEVDKLKEFIPKVKDMLKKEQAVLSSI
TQPLVAASETTEDGGHSTHTLSQSGETEVTEETEVTEETVGHTTTVTITLPPKEESAP
KEVKVVENSIEHKSNDNSQALTKTVYLKKLDEFLTKSYICHKYILVSNSSMDQKLLEV

YNLTPEEENELKSCDPLDLLFNIQNNIPAMYSLYDSMNNDLQHLFFELYQKEMIYYLH
KLKEENHIKKLLEEQKQITGTSSTSSPGNTTVNTAQSATHSNSQNQQSNASSTNTQNG
VAVSSGPAVVEESHDPLTVLSISNDLKGIVSLLNLGNKTKVPNPLTISTTEMEKFYEN
ILKNNDTYFNDDIKQFVKSNSKVITGLTETQKNALNDEIKKLKDTLQLSFDLYNKYKL
KLDRLFNKKKELGQDKMQIKKLTLLKEQLESKLNSLNNPHNVLQNFSVFFNKKKEAEI
AETENTLENTKILLKHYKGLVKYYNGESSPLKTLSEVSIQTEDNYANLEKFRALSKID
GKLNDNLHLGKKKLSFLSSGLHHLITELKEVIKNKNYTGNSPSENNKKVNEALKSYEN
FLPEAKVTTVVTPPQPDVTPSPLSVRVSGSSGSTKEETQIPTSGSLLTELQQVVQLQN
YDEEDDSLVVLPIFGESEDNDEYLDQVVTGEAISVTMDNILSGFENEYDVIYLKPLAG
VYRSLKKQIEKNIITFNLNLNDILNSRLKKRKYFLDVLESDLMQFKHISSNEYIIEDS
FKLLNSEQKNTLLKSYKYIKESVENDIKFAQEGISYYEKVLAKYKDDLESIKKVIKEE
KEKFPSSPPTTPPSPAKTDEQKKESKFLPFLTNIETLYNNLVNKIDDYLINLKAKIND
CNVEKDEAHVKITKLSDLKAIDDKIDLFKNTNDFEAIKKLINDDTKKDMLGKLLSTGL
VQNFPNTIISKLIEGKFQDMLNISQHQCVKKQCPENSGCFRHLDEREECKCLLNYKQE
GDKCVENPNPTCNENNGGCDADATCTEEDSGSSRKKITCECTKPDSYPLFDGIFCSSS
NFLGISFLLILMLILYSFI
>MSP1_PLAFN
MKIIFFLCSFLFFIINTQCVTHESYQELVKKLEALEDAVLTGYSLFQKEKMVLNEEEI
TTKGASAQSGASAQSGASAQSGASAQSGTSGPSGPSGTSPSSRSNTLPRSNTSSGASP
PADASDSDAKSYADLKHRVRNYLFTIKQLKYPESLDLPNHMLTLCDNIHGFKYLIDGY
EEINELLYKLNFYFSLLRAKLNDVCANDYCQIPFNLKIRANELDVLKKLVFGYRKPLD
NIKDNVGKMEDYIKKNKTTIANINELIEGSKKTIDQNKNADNEEGKKKI
>MSP1_PLAFP
MKIIFFLCSFLFFIINTQCVTHESYQELVKKLEALEDAVLTGYGLFHKEKMILNEEEI
TTKGASAQSGTSGTSGTSGTSGTSGTSGTSAQSGTSGTSAQSGTSGTSAQSGTSGTSG
TSGTSPSSRSNTLPRSNTSSGASPPADASDSDAKSYADLKHRVRNYLFTIKELKYPEL
FDLTNHMLTLCDNIHGFKYLIDGYEEINELLYKLNFYFDLLRAKLNDVCANDYCQIPF
NLKIRANELDVLKKLVFGYRKPLDNIKDNVGKMEDYIKKNKTTIANINELIEGSKKTI
DQNKNADNEEGKKKLYQAQYDLSIYNKQLEEAHNLISVLEKRIDTLKKNENIKELLDK
INEIKNPPPANSGNTPNTLLDKNKKIEEHEEKIKEIAKTIKFNIDSLFTDPLELEYYL
REKNKKVDVTPKSQDPTKSVQIPKVPYPNGIVYPLPLTDIHNSLAADNDKNSYGDLMN
PDTKEKINEKIITDNKERKIFINNIKKQIDLEEKKINHTKEQNKKLLEDYEKSKKDYE
ELLEKFYEMKFNNNFDKDVVDKIFSARYTYNVEKQRYNNKFSSSNNSVYNVQKLKKAL
SYLEDYSLRKGISEKDFNHYYTLKTGLEADIKKLTEEIKSSENKILEKNFKGLTHSAN
ASLEVYDIVKLQVQKVLLIKKIEDLRKIELFLKNAQLKDSIHVPNIYKPQNKPEPYYL
IVLKKEVDKLKEFIPKVKDMLKKEQAVLSSITQPLVAASETTEDGGHSTHTLSQSGET
EVTEETEETEETVGHTTTVTITLPPKEVKVVENSIEHKSNDNSQALTKTVYLKKLDEF
LTKSYICHKYILVSNSSMDQKLLEVYNLTPEEENELKSCDPLDLLFNIQNNIPAMYSL
YDSMNNDLQHLFFELYQKEMIYYLHKLKEENHIKKLLEEQKQITGTSSTSSPGNTTVN
TAQSATHSNSQNQQSNASSTNTQNGVAVSSGPAVVEESHDPLTVLSISNDLKGIVSLL
NLGNKTKVPNPLTISTTEMEKFYENILKNNDTYFNDDIKQFVKSNSKVITGLTETQKN
ALNDEIKKLKDTLQLSFDLYNKYKLKLDRLFNKKKELGQDKMQIKKLTLLKEQLESKL
NSLNNPHNVLQNFSVFFNKKKEAEIAETENTLENTKILLKHYKGLVKYYNGESSPLKT
LSEVSIQTEDNYANLEKFRVLSKIDGKLNDNLHLGKKKLSFLSSGLHQLITELKEVIK
NKNYTGNSPSENNKKVNEALKSYENFLPEAKVTTVVTPPQPDVTPSPLSVRVSGSSGS
TKEETQIPTSGSLLTELQQVVQLQNYDEEDDSLVVLPIFGESEDNDEYLDQVVTGEAI
SVTMDNILSGFENEYDVIYLKPLAGVYRSLKKQIEKNIFTFNLNLNDILNSRLKKRKY
FLDVLESDLMQFKHISSNEYIIEDSFKLLNSEQKNTLLKSYKYIKESVENDIKFAQEG
ISYYEKVLAKYKDDLESIKKVIKEEKEKFPSSPPTTPPSPAKTDEQKKESKFLPFLTN
IETLYNNLVNKIDDYLINLKAKINDCNVEKDEAHVKITKLSDLKAIDDKIDLFKNHND
FDAIKKLINDDTKKDMLGKLLSTGLVQNFPNTIISKLIEGKFQDMLNISQHQCVKKQC

PENSGCFRHLDEREECKCLLNYKQEGDKCVENPNPTCNENNGGCDADAKCTEEDSGSN
GKKITCECTKPDSYPLFDGIFCSSSNFLGISFLLLILMLILYSFI
>MSP1_PLAFW
MKIIFFLCSFLFFIINTQCVTHESYQELVKKLEALEDAVLTGYSLFQKEKMVLNEGTS
GTAVTTSTPGSKGSVASGGSGGSVASGGSVASGGSVASGGSVASGGSGNSRRTNPSDN
SSDSDAKSYADLKHRVRNYLLTIKELKYPQLFDLTNHMLTLCDNIHGFKYLIDGYEEI
NELLYKLNFYFDLLRAKLNDVCANDYCQIPFNLKIRANELDVLKKLVFGYRKPLDNIK
DNVGKMEDYIKKNKKTIENINELIEESKKTIDKNKNATKEEEKKKLYQAQYDLSIYNK
QLEEAHNLISVLEKRIDTLKKNENIKELLDKINEIKNPPPANSGNTPNTLLDKNKKIE
EHEKEIKEIAKTIKFNIDSLFTDPLELEYYLREKNKNIDISAKVETKESTEPNEYPNG
VTYPLSYNDINNALNELNSFGDLINPFDYTKEPSKNIYTDNERKKFINEIKEKIKIEK
KKIESDKKSYEDRSKSLNDITKEYEKLLNEIYDSKFNNNIDLTNFEKMMGKRYSYKVE
KLTHHNTFASYENSKHNLEKLTKALKYMEDYSLRNIVVEKELKYYKNLISKIENEIET
LVENIKKDEEQLFEKKITKDENKPDEKILEVSDIVKVQVQKVLLMNKIDELKKTQLIL
KNVELKHNIHVPNSYKQENKQEPYYLIVLKKEIDKLKVFMPKVESLINEEKKNIKTEG
QSDNSEPSTEGEITGQATTKPGQQAGSALEGDSVQAQAQEQKQAQPPVPVPVPEAKAQ
VPTPPAPVNNKTENVSKLDYLEKLYEFLNTSYICHKYILVSHSTMNEKILKQYKITKE
EESKLSSCDPLDLLFNIQNNIPVMYSMFDSLNNSLSQLFMEIYEKEMVCNLYKLKDND
KIKNLLEEAKKVSTSVKTLSSSSMQPLSLTPQDKPEVSANDDTSHSTNLNNSLKLFEN
ILSLGKNKNIYQELIGQKSSENFYEKILKDSDTFYNESFTNFVKSKADDINSLNDESK
RKKLEEDINKLKKTLQLSFDLYNKYKLKLERLFDKKKTVGKYKMQIKKLTLLKEQLES
KLNSLNNPKHVLQNFSVFFNKKKEAEIAETENTLENTKILLKHYKGLVKYYNGESSPL
KTLSEESIQTEDNYASLENFKVLSKLEGKLKDNLNLEKKKLSYLSSGLHHLIAELKEV
IKNKNYTGNSPSENNTDVNNALESYKKFLPEGTDVATVVSESGSDTLEQSQPKKPAST
HVGAESNTITTSQNVDDEVDDVIIVPIFGESEEDYDDLGQVVTGEAVTPSVIDNILSK
IENEYEVLYLKPLAGVYRSLKKQLENNVMTFNVNVKDILNSRFNKRENFKNVLESDLI
PYKDLTSSNYVVKDPYKFLNKEKRDKFLSSYNYIKDSIDTDINFANDVLGYYKILSEK
YKSDLDSIKKYINDKQGENEKYLPFLNNIETLYKTVNDKIDLFVIHLEAKVLNYTYEK
SNVEVKIKELNYLKTIQDKLADFKKNNNFVGIADLSTDYNHNNLLTKFLSTGMVFENL
AKTVLSNLLDGNLQGMLNISQHQCVKKQCPQNSGCFRHLDEREECKCLLNYKQEGDKC
VENPNPTCNENNGGCDADAKCTEEDSGSNGKKITCECTKPDSYPLFDGIFCSSSNFLG
ISFLLLILMLILYSFI
>MYOA_PLAF7
MAVTNEEIKTASKIVRRVSNVEAFDKSGSVFKGYQIWTDISPTIENDPNIMFVKCVVQ
QGSKKEKLTVVQIDPPGTGTPYDIDPTHAWNCNSQVDPMSFGDIGLLNHTNIPCVLDF
LKHRYLKNQIYTTAVPLIVAINPYKDLGNTTNEWIRRYRDTADHTKLPPHVFTCAREA
LSNLHGVNKSQTIIVSGESGAGKTEATKQIMRYFASSKSGNMDLRIQTAIMAANPVLE
AFGNAKTIRNNNSSRFGRFMQLVISHEGGIRYGSVVAFLLEKSRIITQDDNERSYHIF
YQFLKGANSTMKSKFGLKGVTEYKLLNPNSTEVSGVDDVKDFEEVIESLKNMELSESD
IEVIFSIVAGILTLGNVRLIEKQEAGLSDAAAIMDEDMGVFNKACELMYLDPELIKRE
ILIKVTVAGGTKIEGRWNKNDAEVLKSSLCKAMYEKLFLWIIRHLNSRIEPEGGFKTF
MGMLDIFGFEVFKNNSLEQLFINITNEMLQKNFVDIVFERESKLYKDEGISTAELKYT
SNKEVINVLCEKGKSVLSYLEDQCLAPGGTDEKFVSSCATNLKENNKFTPAKVASNKN
FIIQHTIGPIQYCAESFLLKNKDVLRGDLVEVIKDSPNPIVQQLFEGQVIEKGKIAKG
SLIGSQFLNQLTSLMNLINSTEPHFIRCIKPNENKKPLEWCEPKILIQLHALSILEAL
VLRQLGYSYRRTFEEFLYQYKFVDIAAAEDSSVENQNKCVNILKLSGLSESMYKIGKS
MVFLKQEGAKILTKIQREKLVEWENCVSVIEAAILKHKYKQKVNKNIPSLLRVQAHIR
KKMVAQ
>MYOA_PLAFB
MAVTNEEIKTASKIVRRVSNVEAFDKSGSVFKGYQIWTDISPTIENDPNIMFVKCVVQ
QGSKKEKLTVVQIDPPGTGTPYDIDPTHAWNCNSQVDPMSFGDIGLLNHTNIPCVLDF

LKHRYLKNQIYTTAVPLIVAINPYKDLGNTTNEWIRRYRDTADHTKLPPHVFTCAREA
LSNLHGVNKSQTIIVSGESGAGKTEATKQIMRYFASSKSGNMDLRIQTAIMAANPVLE
AFGNAKTIRNNNSSRFGRFMQLVISHEGGIRYGSVVAFLLEKSRIITQDDNERSYHIF
YQFLKGANSTMKSKFGLKGVTEYKLLNPNSTEVSGVDDVKDFEEVIESLKNMELSESD
IEVIFSIVAGILTLGNVRLIEKQEAGLSDAAAIMDEDMGVFNKACELMYLDPELIKRE
ILIKVTVAGGTKIEGRWNKNDAEVLKSSLCKAMYEKLFLWIIRHLNSRIEPEGGFKTF
MGMLDIFGFEVFKNNSLEQLFINITNEMLQKNFVDIVFERESKLYKDEGISTAELKYT
SNKEVINVLCEKGKSVLSYLEDQCLAPGGTDEKFVSSCATNLKENNKFTPAKVASNKN
FIIQHTIGPIQYCAESFLLKNKDVLRGDLVEVIKDSPNPIVQQLFEGQVIEKGKIAKG
SLIGSQFLNQLTSLMNLINSTEPHFIRCIKPNENKKPLEWCEPKILIQLHALSILEAL
VLRQLGYSYRRTFEEFLYQYKFVDIAAAEDSSVENQNKCVNILKLSGLSESMYKIGKS
MVFLKQEGAKILTKIQREKLVEWENCVSVIEAAILKHKYKQKVNKNIPSLLRVQAHIR
KKMVAQ
>NMT_PLAF7
MNDDKKDFVGRDLYQLIRNAKDKIKIDYKFWYTQPVPKINDEFDENVNEPFISDNKVE
DVRKEEYKLPSGYAWCVCDITKENDRSDIYNLLTDNYVEDDDNVFRFNYSSEFLLWAL
SSPNYVKNWHIGVKYESTNKLVGFISAIPIDMCVNKNIIKMAEVNFLCVHKSLRSKRL
APVLIKEITRRINLESIWQAIYTAGVYLPKPISTARYFHRSINVKKLIEIGFSCLNTR
LTMSRAIKLYRIDDTLNIKNLRLMKKKDIDGLQKLLNEHLKQYNLHAIFSKEDVAHWF
TPIDQVIYTYVNEENGEIKDLISFYSLPSKVLGNNKYNILNAAFSFYNITTTTTFKNL
IQDAICLAKRNNFDVFNALEVMDNYSVFQDLKFGEGDGSLKYYLYNWKCASCHPSKIG
IVLL
>OAT_PLAF7
MDFVKELKSSQDYMNNELTYGAHNYDPIPVVLKRGKGVFVYDIEDRRYYDFLSAYSSV
NQGHCHPDILNAMINQAKKLTICSRAFFSDSLGVCERYLTNLFGYDKVLMMNTGAEAS
ETAYKLCRKWGYEVKKIPENSAKIIVCNNNFSGRTLGCVSASTDKKCKNNFGPFVPNF
LKVPYDDLEALEKELQDPNVCAFIVEPVQGEAGVIVPSDSYFPGVASLCKKYNVLFVA
DEVQTGLGRTGKLLCTHHYGVKPDVILLGKALSGGHYPISAILANDDVMLVLKPGEHG
STYGGNPLAAAICVEALKVLINEKLCENADKLGAPFLQNLKEQLKDSKVVREVRGKGL
LCAIEFKNDLVNVWDICLKFKENGLITRSVHDKTVRLTPPLCITKEQLDECTEIIVKT
VKFFDDNL
>OAT_PLAFD
MDFVKELKSSQDYMNNELTYGAHNYDPIPVVLKRGKGVFVYDIEDRRYYDFLSAYSSV
NQGHCHPDILNAMINQAKKLTICSRAFFSDSLGVCERYLTNLFGYDKVLMMNTGAEAS
ETAYKLCRKWGYEVKKIPENSAKIIVCNNNFSGRTLGCVSASTDKKCKNNFGPFVPNF
LKVPYDDLEALEKELQDPNVCAFIVEPVQGEAGVIVPSDSYFPGVASLCKKYNVLFVA
DEVQTGLGRTGKLLCTHHYGVKPDVILLGKALSGGHYPISAILANDDVMLVLKPGEHG
STYGGNPLAAAICVEALKVLINEKLCENADKLGAPFLQNLKEQLKDSKVVREVRGKGL
LCAIEFKNDLVNVWDICLKFKENGLITRSVHDKTVRLTPPLCITKEQLDECTEIIVKT
VKFFDDNL
>ORA_PLAFN
KKVLSFSHSLNTYEGTGVPEKIYNEEKNNGKFRLLGLYGNNSTNWLITDCACMISGVT
TLVMHSKFSIDIIIDILNNTKLEWLCLDLDLVEGLLCRKNELPYLKKLIILDNLTKRS
EMKIENEEKSNGSRKSSNKQKYNESDKREDISLCALECDKEKIEKINSLKEKAKTLGL
SIIVFDNMTENKIANVTVQNEDPNFIASIVYTSGTSGKPKGVMLSNRNLYNGVIPPCD
CNIIKKYPLTTHLSYLPVSHIYERVIFFIALFLGVKINIWSRDIKFLNTDICNSKAEI
ILGVPKVFNRMYATIMTKINNLSRCKKWIAKQAINLRKGKNNGNFSKVVEGITNISRK
IKDKINPNMDVILNGGGKLSPEVAEGLSVLLNVKYYQGYGLTESTGPIFLQDVDDCNT
ESMGVAVSPSTRYKVRTWEIYKATDTIPKGELLIKSDSMFSGYFLEKESTEHAFTNDG
YFKTGDIVQINDNGSLTFLDRSKGLVKLSQGEYIETEMINNLYSQIPFVNFCVAYGDD
SMDGPLGIISVDKHKLFTFLKNDNMLKTTGVDEKNFSEKLIDETLNDPIYVDYVKGKM

MEIYKKTNLNRYNVINDIYLTSKPWDTTNYLTPTLKIRRFNVFKDFSFFIDEVKKKYE
EKLSGSSTGSMNNGKSGSKSDIKGGSKDDIKSGSKDDIKSGSKADIKSGSKDDIKSGS
KDHIK
>OS25_PLAFO
MNKLYSLFLFLFIQLSIKYNNAKVTVDTVCKRGFLIQMSGHLECKCENDLVLVNEETC
EEKVLKCDEKTVNKPCGDFSKCIKIDGNPVSYACKCNLGYDMVNNVCIPNECKNVTCG
NGKCILDTSNPVKTGVCSCNIGKVPNVQDQNKCSKDGETKCSLKCLKENETCKAVDGI
YKCDCKDGFIIDNESSICTAFSAYNILNLSIMFILFSVCFFIM
>PCNA_PLAF7
MLEAKLNNASILKKLFECIKDLVNDANVDADESGLKLQALDGNHVSLVSLHLLDSGFS
HYRCDRERVLGVNIASLNKVFKLCGANESVVISSKDDEDNLNFVFENNKEDKVTNFSL
KLMSIELDSLNIPDCEEGFDAEVELSSKELTNIFRNLSEFSDTVFIEIDSNCIKFTTK
GIVGDAEVALKPRDSTSEDDIGVTIKSKKKIKQSFAIKYLNLFSKSNILADVVVLGLS
DSRPIEFKYEIKDTSPDSDTLKIGFVKFFLAPKMDDDMDNKD
>PCNA_PLAFK
MLEAKLNNASILKKLFECIKDLVNDANVDADESGLKLQALDGNHVSLVSLHLLDSGFS
HYRCDRERVLGVNIASLNKVFKLCGANESVVISSKDDEDNLNFVFENNKEDKVTNFSL
KLMSIELDSLNIPDCEEGFDAEVELSSKELTNIFRNLSEFSDTVFIEIDSNCIKFTTK
GIVGDAEVALKPRDSTSEDDIGVTIKSKKKIKQSFAIKYLNLFSKSNILADVVVLGLS
DSRPIEFKYEIKDTSPDSDTLKIGFVKFFLAPKMDDDMDNKD
>PCY1_PLAFK
MDSSNYFHDCKTMLSEHNESIESSNNDINGKQKEHIKKGNSENQDVDPDTNPDAVPDD
DDDDDDNSNDESEYESSQMDSEKNKGSIKNSKNVVIYADGVYDMLHLGHMKQLEQAKK
LFENTTLIVGVTSDNETKLFKGQVVQTLEERTETLKHIRWVDEIISPCPWVVTPEFLE
KYKIDYVAHDDIPYANNQKKKKKKKSKGKSFSFDEENEDIYAWLKRAGKFKATQRTEG
VSTTDLIVRILKNYEDYIERSLQRGIHPNELNIGVTKAQSIKMKKNLIRWGEKVTDEL
TKVTLTDKPLGTDFDQGVENLQVKFKELFKIWKNASNKLITDFTRKLEATSYLTSIQN
IIDYEIENDDYASSNFDDETSS
>PEBP_PLAFA
MTIPTISELKKDRIIPHVFPNDKIDLNVDLFISFKAGKEVNHGNVLDIAGTGSVPRNI
KFSEEPPDGYCFVLFMVDPDYPSRLRPDGKEYIHWVVSGIKTKELIKGTQKNCVTILP
YVGPSIKKGTGLHRISFIISLIKEEDKDNITGLPHYKGEKYITRVKFNNYESVHNIAQ
INNMKIVGYNWCQIEG
>PF12_PLAFA
MIKLSKKYCLGISFVLYILLSVCEGHKNLTCDFNDVYKLEFHPNQQTSVTKLCNVTPN
VLEKVTIKCGSDKLNYNLYPPTCFEEVYASRNMMHLKKIKEFVIGSSMFMRRSLTPNK
INEVSFRIPPNMMPEKPIYCFCENKKTITINGSNGNPSSKKDIINRGIVEIIIPSLNE
KVKGCDFTTSESTIFSKGYSINEISNKSSNNQQDIVCTVKAHANDLIGFKCPSNYSVE
PHDCFVSAFNLSGKNENLENKLKLTNIIMDHYNNTFYSRLPSLISDNWKFFCVCSKDN
EKKLVFTVEASISSSNTKLASRYNTYQDYISNSSFLTLSSYCAFITFIITSFLSFIL
>PF2L_PLAFP
MILKKEKKRIYILNNNIKNLNLKENVDVLNKRHKKNIIFQTDIYICSTYKGNKNIKKK
SRTIIFIIKVNV
>PGK_PLAF7
MLGNKLSISDLKDIKNKKVLVRVDFNVPIENGIIKDTNRITATLPTINHLKKEGASKI
ILISHCGRPDGLRNEKYTLKPVAETLKGLLGEEVLFLNDCVGKEVEDKINAAKENSVI
LLENLRFHIEEEGKGVDANGNKVKANKEDVEKFQNDLTKLADVFINDAFGTAHRAHSS
MVGVKLNVKASGFLMKKELEYFSKALENPQRPLLAILGGAKVSDKIQLIKNLLDKVDR
MIIGGGMAYTFKKVLNNMKIGTSLFDEAGSKIVGEIMEKAKAKNVQIFLPVDFKIADN
FDNNANTKFVTDEEGIPDNWMGLDAGPKSIENYKDVILTSKTVIWNGPQGVFEMPNFA
KGSIECLNLVVEVTKKGAITIVGGGDTASLVEQQNKKNEISHVSTGGGASLELLEGKE

LPGVLALSNK
>PLM1_PLAF7
MALSIKEDFSSAFAKNESAVNSSTFNNNMKTWKIQKRFQILYVFFFLLITGALFYYLI
DNVLFPKNKKINEIMNTSKHVIIGFSIENSHDRIMKTVKQHRLKNYIKESLKFFKTGL
TQKPHLGNAGDSVTLNDVANVMYYGEAQIGDNKQKFAFIFDTGSANLWVPSAQCNTIG
CKTKNLYDSNKSKTYEKDGTKVEMNYVSGTVSGFFSKDIVTIANLSFPYKFIEVTDTN
GFEPAYTLGQFDGIVGLGWKDLSIGSVDPVVVELKNQNKIEQAVFTFYLPFDDKHKGY
LTIGGIEDRFYEGQLTYEKLNHDLYWQVDLDLHFGNLTVEKATAIVDSGTSSITAPTE
FLNKFFEGLDVVKIPFLPLYITTCNNPKLPTLEFRSATNVYTLEPEYYLQQIFDFGIS
LCMVSIIPVDLNKNTFILGDPFMRKYFTVFDYDNHTVGFALAKKKL
>PLM1_PLAFA
MALSIKEDFSSAFAKNESAVNSSTFNNNMKTWKIQKRFQILYVFFFLLITGALFYYLI
DNVLFPKNKKINEIMNTSKHVIIGFSIENSHDRIMKTVKQHRLKNYIKESLKFFKTGL
TQKPHLGNAGDSVTLNDVANVMYYGEAQIGDNKQKFAFIFDTGSANLWVPSAQCNTIG
CKTKNLYDSNKSKTYEKDGTKVEMNYVSGTVSGFFSKDIVTIANLSFPYKFIEVTDTN
GFEPAYTLGQFDGIVGLGWKDLSIGSVDPVVVELKNQNKIEQAVFTFYLPFDDKHKGY
LTIGGIEDRFYEGQLTYEKLNHDLYWQVDLDLHFGNLTVEKATAIVDSGTSSITAPTE
FLNKFFEGLDVVKIPFLPLYITTCNNPKLPTLEFRSATNVYTLEPEYYLQQIFDFGIS
LCMVSIIPVDLNKNTFILGDPFMRKYFTVFDYDNHTVGFALAKKKL
>PLM2_PLAFA
MDITVREHDFKHGFIKSNSTFDGLNIDNSKNKKKIQKGFQILYVLLFCSVMCGLFYYV
YENVWLQRDNEMNEILKNSEHLTIGFKVENAHDRILKTIKTHKLKNYIKESVNFLNSG
LTKTNYLGSSNDNIELVDFQNIMFYGDAEVGDNQQPFTFILDTGSANLWVPSVKCTTA
GCLTKHLYDSSKSRTYEKDGTKVEMNYVSGTVSGFFSKDLVTVGNLSLPYKFIEVIDT
NGFEPTYTASTFDGILGLGWKDLSIGSVDPIVVELKNQNKIENALFTFYLPVHDKHTG
FLTIGGIEERFYEGPLTYEKLNHDLYWQITLDAHVGNIMLEKANCIVDSGTSAITVPT
DFLNKMLQNLDVIKVPFLPFYVTLCNNSKLPTFEFTSENGKYTLEPEYYLQHIEDVGP
GLCMLNIIGLDFPVPTFILGDPFMRKYFTVFDYDNHSVGIALAKKNL
>PRI1_PLAF7
MKMEIVGDIKDSIVNENDLIFYYRSLCPINDLYNWLNYKNDIKGKYTKLNDPHFFSKR
EFSFTCKKSDQGKEEIYIRWLSFSNPEEFKNKLLSDLVPIKFDIGAIYNFPVSQKDQK
GDIFLPVQKELIFDIDMNDYDDIRTCCTDKKVCKLCWKFLTVAIVLLDTALREDFSFE
HILWVYSGRRGIHCWVADESCRYYTTDARAALADYLNILSGSDTKKKKVSIWGKDKYP
MFERAFDICYKYFDVLMEEQDFFKKGSPHVQKLIDYLPYASGKVTDPLKAMKLNELKE
YINNNNFNSREIFEKFSSIYNFLTPSNYFKRKNVSGNINMPSFVKEIVFHFTYPRLDI
NVSKEINHLLKSPFCIHNSTGRVCVPLDIKNINNFNPQSVPTLKLLREQFDDPKNSHI
EAENRTSLKPYIDYFRRHFIENILLSCVEKKKRLNENSKYVDYNNI
>PRI1_PLAFK
MKMEIVGDIKDSIVNENDLIFYYRSLCPINDLYNWLNYKNDIKGKYTKLNDPHFFSKR
EFSFTCKKSDQGKEEIYIRWLSFSNPEEFKNKLLSDLVPIKFDIGAIYNFPVSQKDQK
GDIFLPVQKELIFDIDMNDYDDIRTCCTDKKVCKLCWKFLTVAIVLLDTALREDFSFE
HILWVYSGRRGIHCWVADESCRYYTTDARAALADYLNILSGSDTKKKKVSIWGKDKYP
MFERAFDICYKYFDVLMEEQDFFKKGSPHVQKLIDYLPYASGKVTDPLKAMKLNELKE
YINNNNFNSREIFEKFSSIYNFLTPSNYFKRKNVSGNINMPSFVKEIVFHFTYPRLDI
NVSKEINHLLKSPFCIHNSTGRVCVPLDIKNINNFNPQSVPTLKLLREQFDDPKNSHI
EAENRTSLKPYIDYFRRHFIENILLSCVEKKKRLNENSKYVDYNNI
>PVA_PLAFA
ETGESKETGESKETGESKETGESKETGESKETGESKETGESKETGESKETGESKETGE
SKETGESKETGESKETGESKETGESKETGESKETGESKETGESKETRIYEETKYNKIT
SEFRETENVKITEESKDREGNKVSGPYENSENSNVTSESEETKKLAEKEENEGEKLGE
NVNDGASENSEDPKKLTEQEENGTKESSEETKDDKPEENEKKADNKKKKK

>PYRD_PLAF7
MISKLKPQFMFLPKKHILSYCRKDVLNLFEQKFYYTSKRKESNNMKNESLLRLINYNR
YYNKIDSNNYYNGGKILSNDRQYIYSPLCEYKKKINDISSYVSVPFKINIRNLGTSNF
VNNKKDVLDNDYIYENIKKEKSKHKKIIFLLFVSLFGLYGFFESYNPEFFLYDIFLKF
CLKYIDGEICHDLFLLLGKYNILPYDTSNDSIYACTNIKHLDFINPFGVAAGFDKNGV
CIDSILKLGFSFIEIGTITPRGQTGNAKPRIFRDVESRSIINSCGFNNMGCDKVTENL
ILFRKRQEEDKLLSKHIVGVSIGKNKDTVNIVDDLKYCINKIGRYADYIAINVSSPNT
PGLRDNQEAGKLKNIILSVKEEIDNLEKNNIMNDESTYNEDNKIVEKKNNFNKNNSHM
MKDAKDNFLWFNTTKKKPLVFVKLAPDLNQEQKKEIADVLLETNIDGMIISNTTTQIN
DIKSFENKKGGVSGAKLKDISTKFICEMYNYTNKQIPIIASGGIFSGLDALEKIEAGA
SVCQLYSCLVFNGMKSAVQIKRELNHLLYQRGYYNLKEAIGRKHSKS
>RAN_PLAFA
MDSQEYIPQYKLILVGDGGVGKTTFVKRHLTGEFEKKYIPTLGVEVHPLKFQTNFGKT
QFNVWDTAGQEKFGGLRDGYYIKSDCAIIMFDVSSRITYKNVPNWYRDITRVCETIPM
VLVGNKVDVKDRQVKSRQIQFHRKRNLQYYDLSARSNYNFEKPFLWLARRLSNQPNLV
FVGEHAKAPEFQIDLNIVREAEKELEQAAAVAIDEEDIEN
>RESA_PLAFF
MRPFHAYSWIFSQQYMGTKNVKEKNPTIYSFDDEEKRNENKSFLKVLCSKRGVLPIIG
ILYIILNGNLGYNGSSSSGVQFTDRCSRNLYGETLPVNPYADSENPIVVSQVFGLPFE
KPTFTLESPPDIDHTNILGFNEKFMTDVNRYRYSNNYEAIPHISEFNPLIVDKVLFDY
NEKVDNLGRSGGDIIKKMQTLWDEIMDINKRKYDSLKEKLQKTYSQYKVQYDMPKEAY
ESKWTQCIKLIDQGGENLEERLNSQFKNWYRQKYLNLEEYRRLTVLNQIAWKALSNQI
QYSCRKIMNSDISSFKHINELKSLEHRAAKAAEAEMKKRAQKPKKKKSRRGWLCCGGG
DIETVEPQQEEPVQTVQEQQVNEYGDILPSLRASITNSAINYYDTVKDGVYLDHETSD
ALYTDEDLLFDLEKQKYMDMLDTSEEESVEENEEEHTVDDEHVEEHTADDEHVEEPTV
ADDEHVEEPTVADEHVEEPTVAEEHVEEPTVAEEHVEEPASDVQQTSEAAPTIEIPDT
LYYDILGVGVNADMNEITERYFKLAENYYPYQRSGSTVFHNFRKVNEAYQVLGDIDKK
RWYNKYGYDGIKQVNFMNPSIFYLLSSLEKFKDFTGTPQIVTLLRFFFEKRLSMNDLE
NKSEHLLKFMEQYQKEREAHVSEYLLNILQPCIAGDSKWNVPIITKLEGLKGSRFDIP
ILESLRWIFKHVAKTHLKKSSKSAKKLQQRTQANKQELANINNNLMSTLKEYLGSSEQ
MNSITYNFENINSNVDNGNQSKNISDLSYTDQKEILEKIVSYIVDISLYDIENTALNA
AEQLLSDNSVDEKTLKKRAQSLKKLSSIMERYAGGKRNDKKSKNFDTKDIVGYIMHGI
STINTEMKNQNENVPEHVQHNAEENVEHDAEENVEHDAEENVEHDAEENVEHDAEENV
EHDAEENVEENVEEVEENVEENVEENVEENVEEVEENVEENVEENVEENVEENVEENV
EENVEENVEENVEEYDEENVEEVEENVEENVEENVEENVEENVEEVEENVEENVEENV
EENVEENVEENVEEYDEENVEEHNEEYDE
>RESA_PLAFN
EFRYSQYKVQYDMPKEAYESKWTQCIKLIDQGGENLEERLNSQFKNWYRQKYLNLEEY
RRLTVLNQIAWKALSNQIQYSCRKIMNSDISSFKHINELKSLEHRAAKAAEAEMKKRA
QKPKKKKSRRGWLCCGGGDIETVEPQQEEPVQTVQEQQVNEYGDILPTLRASITNSAI
NYYDTVKDGVYLDHETSDALYTDEDLLFDLEKQKYMDMLDTSQEESVEENEEEHTVDD
EHVEEHTADDEHVEEPTVADDEHVEEPTVADEHVEEPTVAEEHVEEPTVAEEHVEEPA
SDVQQTSEAAPTIEIPDTLYYDILGVGVNADMNEITERYFKLAENYYPYQRSGSTVFH
NFRKVNEAYQVLGDIDKKRWYNKYGYDGIKQVNFMNPSIFYLLSSLEKFKDFTGTPQI
VTLLRFFFEKRLSMNDLENKSEHLLKFMEQYQKEREAHVSEYLLNILQPCIAGDSKWN
VPIITKLEGLKGSRFDIPILESLRWIFKHVAKTHLKKSSKSAKKLQQRTQANKQELAN
INNNLMSTLKEYLGSSEQMNSITYNFENINSNVDNGNQSKNISDLSYTDQKEILEKIV
SYIVDISLYDIENTALNAAEQLLSDNSVDEKTLKKRAQSLKKLSSIMERYAGGKRNDK
KAKKYDTQDVVGYIMHGISTINKEMKNQNENVPEHVQHNAEANVEHDAEENVEHDAEE
NAEENVEENVEEVEENVEENVEENVGEKKMRREEKKKRVQEPIKIDEIQVYDIIKNEK
KKKTEF

>RESA_PLAFP
NSITYNFENINSNVDNGNQSKNISDLSYTDQKEILEKIVSYIVDISLYDIENTALNAA
EQLLSDNSVDEKTLKKRAQSLKKLSSIMERYAGGKRNDKKAKKYDTQDVVGYIMHGIS
TINKEMKNQNENVPEHVQHNAEANVEHDAEENVEHDAEENVEHDAEENAEENVEENVE
EVEENVEENVEENVEENVEENVEENVEENVEEVEENVEENVEENVEENVEENVEENVE
ENVEENVEENVEENVEEYDEENVEEVEENVEENVEENVEENVEENVEENVEENVEENV
EEYDEENVEEHNGI
>RHOA_PLAFA
VDILEEKTKDQDLEIELYKYMGPLKEQSKSTSAASTSDELSGSEGPSTESTSTGNQGE
DKTTDNTYKEMEELEEAEGTSNLKKGLEFYKSSLKLDQLDKEKPKKKKSKRKKKRDSS
SDRILLEESKTFTSENEL
>RIR1_PLAF4
MYVLNRKGEEEDISFDQILKRIQRLSYGLHELVDPARVTQGVINGMYSGIKTCELDEL
AAQTCAYMATTHPDFSILAARITTDNLHKNTSDDVAEVAEALYTYKDVRGRPASLISK
EVYDFILLHKDRLNKEIDYTRDFNYDYFGFKTLERSYLLRINNKIIERPQHLLMRVSI
GIHIDDIDKALETYHLMSQKYFTHATPTLFNSGTPRPQMSSCFLLSMKADSIEGIFET
LKQCALISKTAGGIGVAVQDIRGQNSYIRGTNGISNGLVPMLRVFNDTARYVDQGGGK
RKGSFAVYIEPWHSDIFEFLDLRKNHGKEELRARDLFYAVWVPDLFMKRVKENKNWTL
MCPNECPGLSETWGEEFEKLYTKYEEENMGKKTVLAQDLWFAILQSQIETGVPYMLYK
DSCNAKSNQKNLGTIKCSNLCCEIIEYTSPDEVAVCNLASIALCKFVDLEKKEFNFKK
LYEITKIITRNLDKIIERNYYPVKEAKTSNTRHRPIGIGVQGLADTFMLLRYPYESDA
AKELNKRIFETMYYAALEMSVELASIHGPYESYQGSPASQGILQFDMWNAKVDNKYWD
WDELKAKIRKHGLRNSLLLAPMPTASTSQILGNNESFEPYTSNIYYRRVLSGEFFVVN
PHLLKDLFDRGLWDEDMKQQLIAHNGSIQYISEIPDDLKELYKTVWEIKQKNIIDMAA
DRGIFIDQSQSLNIYIQKPTFAKLSSMHFYGWEKGLKTGAYYLRTQAATDAIKFTVDT
HVAKNAVKLKNADGVQITREVSRETISTESTVTQNVCPLRRNNDEQCLMCSG
>RIR1_PLAFG
MYVLNRKGEEEDISFDQILKRIQRLSYGLHKLGEYPACVTQGVINGMYSSIKTCELDE
LAAQTCAYMATTHPDFSILAARITTDNLHKNTSDDVAEVAEALYTYKDGRGRPASLIS
KEVYDFILLHKVRLNKEIDYTTHFNYDYFGFKTLERSYLLRINNKIIERPQHLLMRVS
IGIHIDDIDKALETYHLMSQKYFTHATPTLFNSGTPRPQMSSCFLLSMKADSIEGIFE
TLKQCALISKTAGGIGVAVQDIRGQNSYIRGTNGISNGLVPMLRVFNDTARYVDQGGG
KRKGSYAVYIEPWHSDIFEFLDLRKNHGKEELRARDLFYAVWVPDLFMKRVKENKNWT
LMCPNECPGLSETWGEEFEKLYTKYEEENMGKKTVLAQDLWFAILQSQIETGVPIYLY
KDSCNAKPIKNLGTIKCSNLCCEIIEYTSPDEVAVCNLASIALCKFVDLEKKEFNFKK
LYEITKIITRNLDKIIERNYYPVKEAKTSNTRHRPIGIGVQGLADTFMLLRYLYESDA
AKELNKRIYETMYYAALEMSVDWLQSGPYESYQGSPGSQGILQFDMWNAKVDNKYWDW
DELKLKIAKTGLRNLLLLAPMPTASTSQILGNNESFEPYTSNIYYRRVLSGEFFVVNP
HLLKDLFDRGLWDEDMKQQLIAHNGSIQYISEIPDDLKELYKTVWEIKQKNIIDMAAD
RGYFIDQSQSLNIYIQKPTFAKLSSMHFYGWEKGLKTGAYYLRTQAATDAIKFTVDTH
VAKNAVKLKNADGVQITREVSRETIQLNQRYSKCVSFKSNNDEQCLMCSG
>RIR2_PLAF4
MADVINISRIPIFSKQEREFSDLQKGKEINEKILNKESDRFTLYPILYPDVWDFYKKA
EASFWTAEEIDLSSDLKDFEKLNENEKHFIKHVLAFFAASDGIVLENLASKFLREVQI
TEAKKFYSFQIAVENIHSETYSLLIDNYIKDEKERLNLFHAIENIPAVKNKALWAAKW
INDTNSFAERIVANACVEGILFSGSFCAIFWFKKQNKLHGLTFSNELISRDEGLHTDF
NCLIYSLLDNKLPEQIIQNIVKEAVEVERSFICESLPCDLIGMNSRLMSQYIEFVADR
LLECLGCSKIFHSKNPFNWMDLISLQGKTNFFEKRVADYQKSGVMAQRKDQVFCLNTE
F
>RIR2_PLAFG
MRRILNKESDRFTLYPILYPDVFPFYKKAEACFWTAEEIDYSSDLKDFEKLNENEKHF

IKHVLAFFAASDGIVLENLAVSFLREVQITEAKKFYSFQIAVENIHSETYSLLIDNYI
KDEKERLNLFHAIENIPAVKNKALWAAKWINDTNSFAERIVANACVEGILFSGSFCAI
FWFKKQNKLHGLTFSNELISRDEGLHTDFNCLIYSLLDNKLPEQMVQNIVKEAGGVEV
EKSFICESLPCDLIGMNSRLMSQYIEFVADRLLECLGCSKIFHSKNPFNWMDLISLQG
KTNFFEKRVADYQKSGVMAQRKDHVFCLNTEF
>RK18_PLAF7
MYISFILFSIIFIFLGVIENFIINKRVLYKPNFLLYSEKKNKKKSTPEQVTTRVNKDL
KEKKRKRPRSKILECLLKEKVEKVEKVEKNSDENQCSNVDKEIREGKRVPRLRVRNTN
NHIYASIIDDYKKYVLCSTCSRDATLSKILGTYRRKATNRVINNGRTIKSAWEIGKII
GKKALSKGIFKVRFDRARHPYAGKVEALAEGARAVGLLL
>RL37A_PLAF7
MSRRTKKVGLTGKYGTRYGSSLRKQIKKIELMQHAKYLCTFCGKTATKRTCVGIWKCK
KCKRKVCGGAWSLTTPAAVAAKSTIIRLRKQKEEAQKS
>RL44_PLAF7
VNVPKTRKTYCSNKCKKHTMHKVSQYKKGKERLSSLGRRRYDMKQKGFGGQTKPVFKK
KAKTTKKIVLKLECTKCKKKRFQTMKRCKTFEMGADKKKKGGAVY
>RLA0_PLAF8
MAKLSKQQKKQMYIEKLSSLIQQYSKILIVHVDNVGSNQMASVRKSLRGKATILMGKN
TRIRTALKKNLQAVPQIEKLLPLVKLNMGFVFCKDDLSEIRNIILDNKSSSHPARLGV
IAPIDVFIPPGPTGMDPSHTSFLESLGISTKIVKGQIEIQEHVHLIKQGEKVTASSAT
LLRKFNMNPSYGVDVRTVYDDGVIYDAKVLDITDEDILEKFSKGVSNVAALSRATGVI
TEASYPHVFVEAFKNIVALIIDSDYTFPLMKILKKWVENPEAFAAVAAPASAAKADEP
KKEEAKKVEEEEEEEEDGFMGFGMFD
>RLA2_PLAFA
MAMKYVAAYLMCVLGGNENPSTKEVKNVLGAVNADVEDEVLNNFIDSLKGKSCHELIT
DGLKKLQNIGGGVAAAPAGAAAVETAEAKKEDKKEEKKEEEEEEEDDLGFSLFG
>RPB1_PLAFD
MTVDLNIPYSACELKRVKRLELGVLDPEIIKKISVCEIVNVDIYKDGFPREGGLNDIR
MGTIDYRTLCGTCNMNVKYCPGHFGHIELAKPMYHYGFMNVVLNVLRCVCYHCGRLLC
NVNSSKVKYIEKIKVNSLRLRKLAELCLGIRACDHSVEEEGLNINDNSLNNFYNNDLS
NLNMNQQMLLNKSNYTNIFEMVSKEDVDCGCVQPKYSREGPNMYIQFLHSSEEDIDES
KRKLSAEEALEILKKIRKEEMSILGFNSDRCVPASLILTCIPIPPPCARPYVQYGNQR
SEDDLTLKLLDIVKTNIQLKRQTDRGAKSHVLQDLCSLLQFHITTLFDNDIPGMPIAT
TRSKKPIKAIRTRLKGKEGRLRGNLMGKRVDFSARTVITGDPNLNIDYIGVPKSVAMT
LTFCETVTPLNYDNLKKLVERGPYEWPGAKYIIRDNGTKYDLRHVRRNSEKELEYGYK
VERHMTDEDYILFNRQPSLHKMSIMGHKAKILPYSTFRLNLSVTSPYNADFDGDEMNL
HLAQSHETRSEIKHLMIVQRQIVSPQGNKPVMGIVQDSLLAIRKFTRRDNFLTKEEVM
SLLIWIPYWNHVIPTPAIIKPRALWTGKQIFSMLLQFDDMNIEDDKNDTANNKVGRDV
NTNVNKDSSKMNTSGNYYYGNSTNDNTDDYLEKGNAYSRSGNNHPNSPLSIGDNINVG
NVQQNDMSSPNNNNNNNNNNNSNNNNNNNIGGGINSFKRFNMVKINLMRDSSTSSKDD
NPYCSINDGKVIIKNNELLSGIICKRTVGSSSGSLIHVLWHEMGPDKTKDFLSALQKV
TNNWLEYVGFTVSCSDIIASNKVLGKVREILDKSKSEVSKLVEKAQKGELECQPGKSL
YESFETRVNNELNCAREMAGKVASESLDERNNIFSMVASGSKGSIINISQIISCVGQQ
NVEGKRIPFGFNHRSLPHFIKFDYGPESRGFVSNSYLSGLTPQEVFFHAMGGREGIID
TACKTSETGYIQRRLIKAMEDVMVQYDRTVRNSYGDIIQFLYGEDGMAGEYIEDQIID
LMKLDNKEINKLYKYNFDEEPFGKDYYIGNKNDGSRNTTYIDYNKQNILNQEFEELYK
CKNYLCKEIFPDGDIRQHLPINMNRLIEYAKSQFPCIPFVSNNNSTNNNNNNNNNNNI
SNSRKLMDKGNLSSTHNHKENKKRRKRRRRKNKFDKFKNENNELMSEIKKEYENNDLN
NMMISKGDQSPFKGMNEFHMGVADNDMGSDLGNNNNYNNDDFVDDDYVDDDDYDDDDY
DDDDYDDDDLDDDENYSDNINIGGNRKYYGNTLKNNYDENSMLNPIDVVHKVNNFLEK
LVIIKQINSNDTLSVEAQNNATILLKAHLRTYLNSKLLTQTHKVSVKGLDWLLQEIEK

IFYKSLCHPGECVGALAAQSIGEPATQMTLNTFHFAGVGSKNVTLGVPRLKELINIVK
NVKTPSTTIYLDDMVSNDQQKAKDILTKLEYTTLKQLTSHAQIIYDPNTTTTILEEDK
SWVNEFYEFPDEDDTQYSLGEWVLRIQLTNIHVNEKKLTMKEIVYIIYSVFSSDELDI
IYTDDNSEDLVLRIRVKYLNGEYNFMNYDVVDNANEQVDEQEEDEEHLVANDRGNYDE
TKNSTHPHHDYNNNTTNIFKSKVKNNISSDINTKNEDSISINSSNNEQVKNINSSPVS
NNMHNNNNNNNNNDSSNINDIKVKNIKKEDGNEGALRGGGDSNTSALFGNKNSQKEDNI
VNNNNDNNDDDDEEEEEEDFLFGDHNVSPKNTKDGKNKNTNNKSNNNENKNKKSGNNN
SNNSNTYDDGDVDNDNDDDNDDNKSDITIKEDNDVAFMKTSTKNAEEDLELKNKNHIE
HNISREDTEDTFLKKLMEQCLSTLKLRGIENITKVYMREESKITYDSDNGKFVRSSHW
VLDTDGCNLENIFCAPQVDFKKTVSNDIVEIFEVLGIEAVRRALLKELRTVISFDSSY
VNYRHLSILCDVMTQKGYLMSITRHGINRVDKGPLIKCSFEETVEILLEAAAFAQVDN
LRGITENIM
LGQLCKIG
TGSFDIIIDNQKLNDANQNLETIQDLTSAGFTTPDSLHVITPDGLQSPVAINTINSPL
PFSPTYNANLLSPTAPIDNVNNLLSPQYNLQNYGDNVMSPTSKDINNLDTLKLGGKFS
PTQSPKSPTSVMHSPFSPFDHQNQQPVDATNLLFSPKNNNIMNYNVFSPKPNINNNVI
QSPNIYSPNPMLDIFSPKPQINHNIYSPSYSPTSPTYNANNAYYSPTSPKNQNDQMNV
NSQYNVMSPVYSVTSPKYSPTSPKYSPTSPKYSPTSPKYSPTSPKYSPTSPKYSPTSP
KYSPTSPKYSPTSPVAQNIASPNYSPYSITSPKFSPTSPAYSISSPVYDKSGVVNAHQ
PMSPAYILQSPVQIKQNVQDVNMFSPIQQAHVDEAKNDDPFSPMPYNIDEDEMKENM
>RPC1_PLAFA
MMKKKNIDIEELKRLIEESSMKKRFVKDIKRNCEIKSIRFGIMSKEDIIKYSEVKIMN
REMYKNNSGIPYPYGVLDLKLGAHKSNSVCETCNKKLINCSGHFGYIELNYPVFHIGY
YKYIIHILYCICKYCSSLLLSKEKIDFYCNLKKKSTDDSFYKKHLFKRILNNCKKVNK
CYICGNPQGVIKKIIKPSLDQFMKLKHILKVKENGKMIIKEEDLNSLYVLKLFKNINP
YHVKLLNIENPEKLIITALLVPPNTIRPSVIIDEHGTAEDDLTCILSEITQLNNTIYN
QCTNGYQTNQFLGNVEFLQLQITRFINSDSPAVSQLLATQNISKPGRGICQRLKGKEG
RFRCNLSGKRVDFSSRTVISPDPNISIDEVVIPKIIAMRLTYPETVNKYNIDKLKMLI
KNGCNKWPGANYIIKKSKKGTDPYSDISTSYNNNSNNISSIGCSNIFNVVNNYINNNC
KNVRYNIKDVNNNVLLKDMCDINNMNNDINNNINNIYKNTSETNLCNVNNHNNNNNIY
CNNQTQDNEEERKNSQFNKISLKYANKNHVIQNLNIGDVVERHICDGDIVLFNRQPSL
HRMSIMCHKAKIMDFKTFRFNECVCSPYNADFDGDEMNLDVPQTEEARAEALYLMNVK
HNLITPKNGEVIIALTQDFLSASYIITNKDTFLDRDTFCLLCSYFSDASLYIELPIPA
ILKPKELWTGKQLISVLIKPNKKENTIINFEIQEREYSNKFGDLKHLCLNDSYVCFYK
SELICGSLGKKVLGSSKYGLFYYLIHHNSSHIALKIMNRLSKLTSRYFSNKGMTIGID
DVRPSQTLTEKKKDLLLKGYEKVNNEIILYNEKKMQIQPGCTLEETLEIKVKSILDDL
RNDAGKTCNQYLHYLNKPLIMFNSGAKGALINIAQMIACVGQQNVAGQRIQNGFINRT
LPHFHFHCKDSESRGFVQNSFYTGLSPTEFFFHTMSGREGLVDTAVKTAETGYMQRRL
MKALEDLSIHYDYSVRSCDKQIVQFIYGDDALNPSYIDNNNTYLDQFDKVFDHIVSIS
SSHLLLSYKNKIPYLPHVQHQNKTSNMNNIYNNMNNINNNDSNRSIIYNNDSNMNNIN
NNDSNMNSIHNNNSNMNNIHNNDSNRSIIHNNDSNMNSIHNNDSNMNSIHNNNSNMNN
IHNNDSNRSIIHNNDSNMNSIHNNDSNNNNNYKDCTHNPYICNESLIIRNIMNRLIYQ
NIAQEDLFIPLEHDEFLVNKIMESYTDQECNYEDIIRSLDLNKNVSYIHNDQGKHLSL
QMCAEEHITINNTNNDNTYVEQIEMKELSKNKTKEKQSFKGTIRDMHEDSEEQMNKFI
TKKAKFFIEKKKGKMHECNDDIEYNNTQYDNIQYNNISCNYIKSQNLENTHHQVNNDL
SFIKNNVILPPKEYHSIFHFVNDYRNVVEIKNLMDKKKIFLNNSEKNVVQSKYNRMSK
NLKKKIEIINNIYRNEKKKLNRWKTKMDNDDNYWSSDDDSIIAKKIIKIKNKEKRKYH
PKEEKENFDRNNYKMITDNNNNDNNNNNNDNNNNDNNNNNNNSNNNNYYYNLHDDVNN
LGVTNYNTNIYPNDCNGIYEKETNNNELTTNSNMCDKNNDFSDEFFNNINENDLLYDN
KYYRQIFKNVIGFVSVFEYVESYKQHYILFPYEIIKWTSFLLEYLTEIIPTNIFLHTK
LSKKEKPTHQKNTGKMKIYIEEIKKWLFIKAINIYKYFSFKKSIELIKKKDYFNYIIK

NYDISHRYIIHDYSFINLKQLYLFIFFNIYKYFKYISTPGDAVGSISAQSIGEPGTQM
TLKTFHFAGVASMNVTLGVPRIKEIINASNSIQTPILNIPLEVNDNYNFALMMKSKLE
KTTIRDICMYIKEDYTSRGVFLSVKFNEELIQKLFLNINAYNIKDIILKQSHINKIKI
NKIHINVINKYKLHISLKNDEFIFFQMESLKKGLLDLLIYGDKDIKRCIIKKEDIEVT
DNEDEICDDMDEYYNVSQGTELYERKCNSKEENKNAIRVKKEEIDDNLEKEENIIYVS
EKDSVNQLK
SEKKKDIN
DDNNNNDDNNNNNDDDNKINDTIFNDDIDSDRNNLKENGSKLENVGEHIIERLSYKMK
EKNVKKEHIKKEPNLINLDTINLDTLNFDEINVHNINNEKIEFYDEHLNICQGNKKHI
QKKKKKKTVYSILVEGNSLNYVLGLEGVDFKHIISNHVINVFQVLGIEAARITIINEI
KKCVEAYSIDIDIRHIMLLADIMAFTGDILGINRFGIQKARQSTLMLASFEETNEHLF
VSSFFKNVDEINNISESIIVGKNIPIGTGAFQLLYDYKLEKETKNLTLLEKAERETAM
NY
>RPO2J_PLAF7
MSVPTLSNKPETVDLLVLAPGEKKVTCTISDKGDCNIFVIKLEDHTIGNLIKIQLCQD
PKVLFAAYRQPHPLQNAIEITIKPKGYAGVKLLSDNVNNILSQVATLKENFAKKIQKY
KESNSYYEDY
>RPOB_PLAFA
MIYIVNPILVKNNYIISNLYLLLIQEIIYNLRYYILFLNNNINVKFNFIYYKIIILLT
NININSIDTIQNINNLLKIILTLKLNFININKIIKFNILIFILPFIYNNIIILNGLYK
TCIQLFKKNNKIFIIKFKNNNKNIIYVYIYISLGLRIIFKISKLNIDCYFNNFKFNFL
ILLLYLNNIYINKNISLFIYNNIINKKILIYNYIKFIYSKYNNINNIISLKLFIIKLN
KFNNIYINLLNILFSIKLNFSYYSDFYINNIYNKKFYSIIDNLLIKSKKYLKIFKYQL
LNINRNIYNNITLLLNNKKYINIILENININPLVQYSDQVNNLSEINQKFKINMITTG
LNSKFILNNDLRELPRNILGYISLINTNEGLTCGLVNYLTTNIFLNLKYLFVIYYKHI
FYNRYNFKLLLNIFNKNFYNISFNNIYLKKNINFNKTTILTINKNTFKICNITQNIIY
IPFNYLLSFIENLIPFIHYNDSIRNLMSIKMHTQIVPIIYPNLSNIITNYNFILNKYL
NHLIISYQEGIVIYVSCIKIIIRDLFNRQIIYYLNNYKKINQNILLIYKPIVWVGEKV
NIGQILAINSNLLNSEYSLGNNLLVGYGSYLGYEYEDAIIISRKILYNNLYTSLHLNI
YEISLNIINNIPEICSINLSKMYYKNIKHLDKYGIIKEGTYILANNILISKLMFMPFI
FNNKSLINIINFLFGSKLRIFKNKPIISTIHDIGRVIKIEILPNHLYNKTEKNNIYLK
FRIYIGIQKYLQLGDKICNRHGHKGIISYISEINDIPYLNNKIQPDIFISAISIPSRI
NIGQIFEGIYGLNSLYLNTRYIISNNLNKNYYNNYNHIFNYYKYNYNNNFNINSKMSY
NYNKYYLKNPFTGNMINNSICLNNIYYYKLIHMIKDKFRYRFIGLYSELTQQPIKGNT
KQGGQRFGEMEVWALEAFGASYLFKEFFTYKSDDIKSRKILKNYLFNNYKIKNTFISE
TFKLILKELQSLAINIEAFCIFNDTNNLLENLPINIIY
>RPOC_PLAFA
MIIHNNINFIGLKLNILNPKQIIKWSSIFYKNKIIIGEVLIPNTINFNTGLPILNGLF
CEKIFDYMYKWNCNCNKKMYNINNFSFFLYCKFCKNKLIININRKYKLGFIFLNIPIL
HLWYLTGPLKVASLLLNKNVFYLKFLIYYKYFFSNIKYKQYFYYNKLFSKINLYKKKY
KNIIQYLFSHNILYKKLQNINLLTELLNNKELLLINNKYYNKKYLYKKINLFNLFILN
NIKPNWIFLDLLPILPAGLRPYFYINNSTYIISTINENYRLIILKNNKLKYWLYLRNN
IFFIFEIIEKRLLQQLIDYLLINKLILKNNNTFFNFSKTFQGKYSTIKYKLLGKRVDF
SGRSVITVNPSIIYNNIGLPYYISINLFKPFLINILKYNSKLNIIFKSLLINKNLFII
QKFLNRLLQNQFIIINRAPTLHRMNLQSFKPLLTEGYSLKFYPLGCTSFNADFDGDQM
SIFLPLIKTSKFESNINLNFDKNIISPSNNKNLFSNLQYYKLGINTLLILNYNNELNI
FYFNSIEKIYEYYNNNILFIFNLVWIKYINNNNIFYILTSINRIIINLYMYIY
>RS12_PLAF7
MSDVESADNNVVVEEKAVFDNVTAIQKVIKNAHVHDGLKIGIREVIKSIESQEAKVCF
LSDVCSEPAYKKLITTLCAEKNIPLFMVQNDSKDLGHWAGLFKLDNEGNARKIIGASS
VAVVDFGEDSAEKDFLLSQNQTVTA

>RS30_PLAF7
MGKVHGSLARAGKVKNQTPKVPKLDKKKRLTGRAKKRQLYNRRFSDNGGRKKGPNSKA
>S230_PLAF7
MKKIITLKNLFLIILVYIFSEKKDLRCNVIKGNNIKDDEDKRFHLFYYSHNLFKTPET
KEKKNKKECFYKNGGIYNLSKEIRMRKDTSVKIKQRTCPFHKEGSSFEMGSKNITCFY
PIVGKKERKTLDTIIIKKNVTNDHVVSSDMHSNVQEKNMILIRNIDKENKNDIQNVEE
KIQRDTYENKDYESDDTLIEWFDDNTNEENFLLTFLKRCLMKIFSSPKRKKTVVQKKH
KSNFFINSSLKYIYMYLTPSDSFNLVRRNRNLDEEDMSPRDNFVIDDEEEEEEEEEE
EEEEEEEEEEEEEEYDDYVYEESGDETEEQLQEEHQEEVGAESSEESFNDEDEDSVEA
RDGDMIRVDEYYEDQDGDTYDSTIKNEDVDEEVGEEVGEEVGEEVGEEVGEEVGEEVG
EEVGEEVGEEEGEEVGEGVGEEVGEEEGEEVGEEEGEYVDEKERQGEIYPFGDEEEKD
EGGESFTYEKSEVDKTDLFKFIEGGEGDDVYKVDGSKVLLDDDTISRVSKKHTARDGE
YGEYGEAVEDGENVIKIIRSVLQSGALPSVGVDELDKIDLSYETTESGDTAVSEDSYD
KYASNNTNKEYVCDFTDQLKPTESGPKVKKCEVKVNEPLIKVKIICPLKGSVEKLYDN
IEYVPKKSPYVVLTKEETKLKEKLLSKLIYGLLISPTVNEKENNFKEGVIEFTLPPVV
HKATVFYFICDNSKTEDDNKKGNRGIVEVYVEPYGNKINGCAFLDEDEEEEKYGNQIE
EDEHNEKIKMKTFFTQNIYKKNNIYPCYMKLYSGDIGGILFPKNIKSTTCFEEMIPYN
KEIKWNKENKSLGNLVNNSVVYNKEMNAKYFNVQYVHIPTSYKDTLNLFCSIILKEEE
SNLISTSYLVYVSINEELNFSLFDFYESFVPIKKTIQVAQKNVNNKEHDYTCDFTDKL
DKTVPSTANGKKLFICRKHLKEFDTFTLKCNVNKTQYPNIEIFPKTLKDKKEVLKLDL
DIQYQMFSKFFKFNTQNAKYLNLYPYYLIFPFNHIGKKELKNNPTYKNHKDVKYFEQS
SVLSPLSSADSLGKLLNFLDTQETVCLTEKIRYLNLSINELGSDNNTFSVTFQVPPYI
DIKEPFYFMFGCNNNKGEGNIGIVELLISKQEEKIKGCNFHESKLDYFNENISSDTHE
CTLHAYENDIIGFNCLETTHPNEVEVEVEDAEIYLQPENCFNNVYKGLNSVDITTILK
NAQTYNINNKKTPTFLKIPPYNLLEDVEISCQCTIKQVVKKIKVIITKNDTVLLKREV
QSESTLDDKIYKCEHENFINPRVNKTFDENVEYTCNIKIENFFNYIQIFCPAKDLGIY
KNIQMYYDIVKPTRVPQFKKFNNEELHKLIPNSEMLHKTKEMLILYNEEKVDLLHFYV
FLPIYIKDIYEFNIVCDNSKTMWKNQLGGKVIYHITVSKREQKVKGCSFDNEHAHMFS
YNKTNVKNCIIDAKPKDLIGFVCPSGTLKLTNCFKDAIVHTNLTNINGILYLKNNLAN
FTYKHQFNYMEIPALMDNDISFKCICVDLKKKKYNVKSPLGPKVLRALYKKLNIKFDN
YVTGTDQNKYLMTYMDLHLSHKRNYLKELFHDLGKKKPADTDANPESIIESLSINESN
ESGPFPTGDVDAEHLILEGYDTWESLYDEQLEEVIYNDIESLELKDIEQYVLQVNLKA
PKLMMSAQIHNNRHVCDFSKNNLIVPESLKKKEELGGNPVNIHCYALLKPLDTLYVKC
PTSKDNYEAAKVNISENDNEYELQVISLIEKRFHNFETLESKKPGNGDVVVHNGVVDT
GPVLDNSTFEKYFKNIKIKPDKFFEKVINEYDDTEEEKDLESILPGAIVSPMKVLKKK
DPFTSYAAFVVPPIVPKDLHFKVECNNTEYKDENQYISGYNGIIHIDISNSNRKINGC
DFSTNNSSILTSSVKLVNGETKNCEININNNEVFGIICDNETNLDPEKCFHEIYSKDN
KTVKKFREVIPNIDIFSLHNSNKKKVAYAKVPLDYINKLLFSCSCKTSHTNTIGTMKV
TLNKDEKEE
EDFKTAQG
IKHNNVHLCNFFDNPELTFDNNKIVLCKIDAELFSEVIIQLPIFGTKNVEEGVQNEEY
KKFSLKPSLVFDDNNNDIKVIGKEKNEVSISLALKGVYGNRIFTFDKNGKKGEGISFF
IPPIKQDTDLKFIINETIDNSNIKQRGLIYIFVRKNVSENSFKLCDFTTGSTSLMELN
SQVKEKKCTVKIKKGDIFGLKCPKGFAIFPQACFSNVLLEYYKSDYEDSEHINYYIHK
DKKYNLKPKDVIELMDENFRELQNIQQYTGISNITDVLHFKNFNLGNLPLNFKNHYST
AYAKVPDTFNSIINFSCNCYNPEKHVYGTMQVESDNRNFDNIKKNENVIKNFLLPNIE
KYALLLDDEERQKKIKQQQEEEQQEQILKDQDDRLSRHDDYNKNHTYILYDSNEHICD
YEKNESLISTLPNDTKKIQKSICKINAKALDVVTIKCPHTKNFTPKDYFPNSSLITND
KKIVITFDKKNFVTYIDPTKKTFSLKDIYIQSFYGVSLDHLNQIKKIHEEWDDVHLFY
PPHNVLHNVVLNNHIVNLSSALEGVLFMKSKVTGDETATKKNTTLPTDGVSSILIPPY
VKEDITFHLFCGKSTTKKPNKKNTSLALIHIHISSNRNIIHGCDFLYLENQTNDAISN

NNNNSYSIFTHNKNTENNLICDISLIPKTVIGIKCPNKKLNPQTCFDEVYYVKQEDVP
SKTITADKYNTFSKDKIGNILKNAISINNPDEKDNTYTYLILPEKFEEELIDTKKVLA
CTCDNKYIIHMKIEKSTMDKIKIDEKKTIGKDICKYDVTTKVATCEIIDTIDSSVLKE
HHTVHYSITLSRWDKLIIKYPTNEKTHFENFFVNPFNLKDKVLYNYNKPINIEHILPG
AITTDIYDTRTKIKQYILRIPPYVHKDIHFSLEFNNSLSLTKQNQNIIYGNVAKIFIH
INQGYKEIHGCDFTGKYSHLFTYSKKPLPNDDDICNVTIGNNTFSGFACLSHFELKPN
NCFSSVYDYNEANKVKKLFDLSTKVELDHIKQNTSGYTLSYIIFNKESTKLKFSCTCS
SNYSNYTIRITFDPNYIIPEPQSRAIIKYVDLQDKNFAKYLRKL
>S230_PLAFO
MKKIITLKNLFLIILVYIFSEKKDLRCNVIKGNNIKDDEDKRFHLFYYSHNLFKTPET
KEKKNKKECFYKNGGIYNLSKEIRMRKDTSVKIKQRTCPFHKEGSSFEMGSKNITCFY
PIVGKKERKTLDTIIIKKNVTNDHVVSSDMHSNVQEKNMILIRNIDKENKNDIQNVEE
KIQRDTYENKDYESDDTLIEWFDDNTNEENFLLTFLKRCLMKIFSSPKRKKTVVQKKH
KSNFFINSSLKYIYMYLTPSDSFNLVRRNRNLDEEDMSPRDNFVIDDEEEEEEEEEE
EEEEEEEEEEEEEEYDDYVYEESGDETEEQLQEEHQEEVGAESSEESFNDEDEDSVEA
RDGDMIRVDEYYEDQDGDTYDSTIKNEDVDEEVGEEVGEEVGEEVGEEVGEEVGEEVG
EEVGEEVGEEEGEEVGEGVGEEVGEEEGEEVGEEEGEYVDEKERQGEIYPFGDEEEKD
EGGESFTYEKSEVDKTDLFKFIEGGEGDDVYKVDGSKVLLDDDTISRVSKKHTARDGE
YGEYGEAVEDGENVIKIIRSVLQSGALPSVGVDELDKIDLSYETTESGDTAVSEDSYD
KYASNNTNKEYVCDFTDQLKPTESGPKVKKCEVKVNEPLIKVKIICPLKGSVEKLYDN
IEYVPKKSPYVVLTKEETKLKEKLLSKLIYGLLISPTVNEKENNFKEGVIEFTLPPVV
HKATVFYFICDNSKTEDDNKKGNRGIVEVYVEPYGNKINGCAFLDEDEEEEKYGNQIE
EDEHNEKIKMKTFFTQNIYKKNNIYPCYMKLYSGDIGGILFPKNIKSTTCFEEMIPYN
KEIKWNKENKSLGNLVNNSVVYNKEMNAKYFNVQYVHIPTSYKDTLNLFCSIILKEEE
SNLISTSYLVYVSINEELNFSLFDFYESFVPIKKTIQVAQKNVNNKEHDYTCDFTDKL
DKTVPSTANGKKLFICRKHLKEFDTFTLKCNVNKTQYPNIEIFPKTLKDKKEVLKLDL
DIQYQMFSKFFKFNTQNAKYLNLYPYYLIFPFNHIGKKELKNNPTYKNHKDVKYFEQS
SVLSPLSSADSLGKLLNFLDTQETVCLTEKIRYLNLSINELGSDNNTFSVTFQVPPYI
DIKEPFYFMFGCNNNKGEGNIGIVELLISKQEEKIKGCNFHESKLDYFNENISSDTHE
CTLHAYENDIIGFNCLETTHPNEVEVEVEDAEIYLQPENCFNNVYKGLNSVDITTILK
NAQTYNINNKKTPTFLKIPPYNLLEDVEISCQCTIKQVVKKIKVIITKNDTVLLKREV
QSESTLDDKIYKCEHENFINPRVNKTFDENVEYTCNIKIENFFNYIQIFCPAKDLGIY
KNIQMYYDIVKPTRVPQFKKFNNEELHKLIPNSEMLHKTKEMLILYNEEKVDLLHFYV
FLPIYIKDIYEFNIVCDNSKTMWKNQLGGKVIYHITVSKREQKVKGCSFDNEHAHMFS
YNKTNVKNCIIDAKPKDLIGFVCPSGTLKLTNCFKDAIVHTNLTNINGILYLKNNLAN
FTYKHQFNYMEIPALMDNDISFKCICVDLKKKKYNVKSPLGPKVLRALYKKLNIKFDN
YVTGTDQNKYLMTYMDLHLSHKRNYLKELFHDLGKKKPADTDANPESIIESLSINESN
ESGPFPTGDVDAEHLILEGYDTWESLYDEQLEEVIYNDIESLELKDIEQYVLQVNLKA
PKLMMSAQIHNNRHVCDFSKNNLIVPESLKKKEELGGNPVNIHCYALLKPLDTLYVKC
PTSKDNYEAAKVNISENDNEYELQVISLIEKRFHNFETLESKKPGNGDVVVHNGVVDT
GPVLDNSTFEKYFKNIKIKPDKFFEKVINEYDDTEEEKDLESILPGAIVSPMKVLKKK
DPFTSYAAFVVPPIVPKDLHFKVECNNTEYKDENQYISGYNGIIHIDISNSNRKINGC
DFSTNNSSILTSSVKLVNGETKNCEININNNEVFGIICDNETNLDPEKCFHEIYSKDN
KTVKKFREVIPNIDIFSLHNSNKKKVAYAKVPLDYINKLLFSCSCKTSHTNTIGTMKV
TLNKDEKEE
EDFKTAQG
IKHNNVHLCNFFDNPELTFDNNKIVLCKIDAELFSEVIIQLPIFGTKNVEEGVQNEEY
KKFSLKPSLVFDDNNNDIKVIGKEKNEVSISLALKGVYGNRIFTFDKNGKKGEGISFF
IPPIKQDTDLKFIINETIDNSNIKQRGLIYIFVRKNVSENSFKLCDFTTGSTSLMELN
SQVKEKKCTVKIKKGDIFGLKCPKGFAIFPQACFSNVLLEYYKSDYEDSEHINYYIHK
DKKYNLKPKDVIELMDENFRELQNIQQYTGISNITDVLHFKNFNLGNLPLNFKNHYST

AYAKVPDTFNSIINFSCNCYNPEKHVYGTMQVESDNRNFDNIKKNENVIKNFLLPNIE
KYALLLDDEERQKKIKQQQEEEQQEQILKDQDDRLSRHDDYNKNHTYILYDSNEHICD
YEKNESLISTLPNDTKKIQKSICKINAKALDVVTIKCPHTKNFTPKDYFPNSSLITND
KKIVITFDKKNFVTYIDPTKKTFSLKDIYIQSFYGVSLDHLNQIKKIHEEWDDVHLFY
PPHNVLHNVVLNNHIVNLSSALEGVLFMKSKVTGDETATKKNTTLPTDGVSSILIPPY
VKEDITFHLFCGKSTTKKPNKKNTSLALIHIHISSNRNIIHGCDFLYLENQTNDAISN
NNNNSYSIFTHNKNTENNLICDISLIPKTVIGIKCPNKKLNPQTCFDEVYYVKQEDVP
SKTITADKYNTFSKDKIGNILKNAISINNPDEKDNTYTYLILPEKFEEELIDTKKVLA
CTCDNKYIIHMKIEKSTMDKIKIDEKKTIGKDICKYDVTTKVATCEIIDTIDSSVLKE
HHTVHYSITLSRWDKLIIKYPTNEKTHFENFFVNPFNLKDKVLYNYNKPINIEHILPG
AITTDIYDTRTKIKQYILRIPPYVHKDIHFSLEFNNSLSLTKQNQNIIYGNVAKIFIH
INQGYKEIHGCDFTGKYSHLFTYSKKPLPNDDDICNVTIGNNTFSGFACLSHFELKPN
NCFSSVYDYNEANKVKKLFDLSTKVELDHIKQNTSGYTLSYIIFNKESTKLKFSCTCS
SNYSNYTIRITFDPNYIIPEPQSRAIIKYVDLQDKNFAKYLRKL
>SAHH_PLAF7
MVENKSKVKDISLAPFGKMQMEISENEMPGLMRIREEYGKDQPLKNAKITGCLHMTVE
CALLIETLQKLGAQIRWCSCNIYSTADYAAAAVSTLENVTVFAWKNETLEEYWWCVES
ALTWGDGDDNGPDMIVDDGGDATLLVHKGVEYEKLYEEKNILPDPEKAKNEEERCFLT
LLKNSILKNPKKWTNIAKKIIGVSEETTTGVLRLKKMDKQNELLFTAINVNDAVTKQK
YDNVYGCRHSLPDGLMRATDFLISGKIVVICGYGDVGKGCASSMKGLGARVYITEIDP
ICAIQAVMEGFNVVTLDEIVDKGDFFITCTGNVDVIKLEHLLKMKNNAVVGNIGHFDD
EIQVNELFNYKGIHIENVKPQVDRITLPNGNKIIVLARGRLLNLGCATGHPAFVMSFS
FCNQTFAQLDLWQNKDTNKYENKVYLLPKHLDEKVALYHLKKLNASLTELDDNQCQFL
GVNKSGPFKSNEYRY
>SANT_PLAF7
MNRILSVTLCLFFIYLYIYKTYGKVKNTDEGLSNIYGAKYYLRSGLFNEKNGKGQKYE
DLEEEKEGENDDEEDSNSEESNNDEENELIKGQEGVEQETHGSEDEVSNGREDKVSNG
GEDEVSNGGEDEVSNGREDKVSNGGEDEVSNGREDKVSNGGEDEVSNGREDKVSNGGE
DEVSNGREDKVSNGGEDEVSNGREDKVSNGREDKVSNGGEDEVSNGREDKVSNGREDK
VSNGGEDEVSNGREDKVSNGGEDEVSNGREDKVSNGGEDEVSNGREDKVSNGREDEVS
NGREDKVSNGGEDEVSNGREDKVSNGGEDEVSNGREDKVSNGREDKVSNGGEDEVSNG
REDKVSNGGEDEVSNGREDKVSNGREDKVSNGREDEVSNGREDKVSNGGEDEVSNGRE
DKVSNGREDKVSNGGEDEVSNGREDKVSNGGEDEVSNGREDKVSNGREDKVSNGREDK
VSNGGEDEVSNGGEDEVSNGREDKVSNGGEDEVSNGREDKVSNGGEDEVSNGREDKVS
NGGEDEVSNGREDKVSNGREDEVSNGREDKGGAGTDGELSHNSESHTKNKKSKNSIIN
MLIGM
>SANT_PLAFF
MNRILSVTFYLFFIYLYIYETYGKVKNTDKELSDIYGTKYYLRSGFFNSKKCKGHKYE
DLQAEGEGENDKEEDSNNEEMNIDEENGLIEGQGESEDPAKASQGGLEDPAKASQGGL
EDPAKASQGGLEDPAKASQGGLEDPAKASQGGLEDPAKASQGGLEDPAKASQGGLEDP
AKASQGGLEDPAKASQGGLEDPAKASQGGLEDPAKASQGGLEDPAKASQGGLEDPAKA
SQGGAEGHGKHAPNKENKNKNKESIKNIMNMFI
>SANT_PLAFN
MNRILSVSFYLFFLYLYIYKTYGKVKNTDQEISNIYGTNYYLRNGFLNGKNGKGNKYE
DLQEEGEGENDDEEHSNSEESDNDEENEIIVGQDEAPKSDEAEALKSDEAEALKSDEA
EARKSDEAEALKSDEAEARKSDEAEALKSDEAEALKSDEAEARKSDEAEALKSDEAEA
LKSDEAEARKSDEAEARKSDEAEARKSDEAEARKSDEAEALKSDEAEARKSDEAEARK
SDEAEALKSDEAEARKSDEAEARKSEAGTEGPKGTGGPGSEAGTEGPKGTGGPGSGGE
HSHNKKKSKKSIMNMLILM
>SANT_PLAFP
EFRPNSDGDKGPNSDGDKGPNSDGDKGPNSDGDKGPNSDGDKGPNSDGDKGPNSDGDK

GPNSDGDKGPNSDGDTEF
>SANT_PLAFV
MNRILSVSFYLFFLYLYIYKTYGKVKNTDQEISNIYGTNYYLRNGFLNGKNGKGNKYE
DLQEEGEGENDDEEHSNSEESDNDEENEIIVGQDGSDQEVKVQKEQGGPGSEGPKGTG
GPGSEGPKGTGGPGSEGPKGTGGPGSEGPKGTGGPGSEGPKGTGGPGSEGPKGTGGPG
SEGPKGTGGPGSEGPKGTGGPGSEGPKGTGGPGSEGPKGTGGPGSEGPKGTGGPGSEG
PKGTGGPGSEGPKGTGGPGSEGPKGTGGPGSEGPKGTGGPGSEGPKGTGGPGSESPKG
TGGPGSEGPKGTGGPGSEGPKGTGPKGTGGPGSEAGTEGPKGTGGPGSEAGTEGPKGT
GGPGSGGEHSHNKKKSKKSIMNMLIGV
>SANT_PLAFW
MNRILSVSFYLFFLYLYIYKTYGKVKNTDHELSNIYGIKYYLRNGLSDKKNGKGQKYQ
DLEEDENDDEEDSNSEESNNDEENKLIEGQEGPNSDGDKGPNSDGDKGPNSDGDKGPN
SDGDKGPNSDGDKGPNSDGDKGPNSDGDKGPNSDGDKGPNSDGDKGPNSDGDKGPNSD
GDKGPNSDGDKGPNSDGDKGPNSDGDKGPNSDGDKGPNSDGDKGPNSDGDKGPNSDGD
KGPNSDGDKGPNSDGDKGPNSDGDKGPNSDGDKGPNSDGDKGPNSDGDKGPNSDGDKG
PNSDGDKGPNSDGDKGPNSDGDKGPNSDGDKGPNSDGDKGPNSDGDKGPNSDGDKGPN
SDGDKGPNSDGDKGPNSDGDKGPNSDGDKGPNSDGDKGPNSDGDKGPNSDGDKGPNSD
GDKGPNSDGDKGPNSDGDKGPNSDGDKGPNSDGDKGPNSDGDKGPNSDGDKGPNSDGD
KGPNSDGDKGPNSDGDKGPNSDGDKGPNSDGDKGPNSDGDKGPNSDGDKGPNSDGDKG
PNSDGDKGPNSDGDKGPNSDGDKGPNSDGDKGPNSDGDKGPNSDGDKGPNSDGDKGPN
SDGDKGPNSDGDKGPNSDGDKGPNSDGDKGPNSDGEHSRSKNDNKKKKSKKNIINMFI
GM
>SERA_PLAF7
MKSYISLFFILCVIFNKNVIKCTGESQTGNTGGGQAGNTGGDQAGSTGGSPQGSTGAS
PQGSTGASPQGSTGASQPGSSEPSNPVSSGHSVSTVSVSQTSTSSEKQDTIQVKSALL
KDYMGLKVTGPCNENFIMFLVPHIYIDVDTEDTNIELRTTLKKTNNAISFESNSGSLE
KKKYVKLPSNGTTGEQGSSTGTVRGDTEPISDSSSSSSSSSSSSSSSSSSSSSSSSSS
SESLPANGPDSPTVKPPRNLQNICETGKNFKLVVYIKENTLILKWKVYGETKDTTENN
KVDVRKYLINEKETPFTNILIHAYKEHNGTNLIESKNYAIGSDIPEKCDTLASNCFLS
GNFNIEKCFQCALLVEKENKNDVCYKYLSEDIVSKFKEIKAETEDDDEDDYTEYKLTE
SIDNILVKMFKTNENNDKSELIKLEEVDDSLKLELMNYCSLLKDVDTTGTLDNYGMGN
EMDIFNNLKRLLIYHSEENINTLKNKFRNAAVCLKNVDDWIVNKRGLVLPELNYDLEY
FNEHLYNDKNSPEDKDNKGKGVVHVDTTLEKEDTLSYDNSDNMFCNKEYCNRLKDENN
CISNLQVEDQGNCDTSWIFASKYHLETIRCMKGYEPTKISALYVANCYKGEHKDRCDE
GSSPMEFLQIIEDYGFLPAESNYPYNYVKVGEQCPKVEDHWMNLWDNGKILHNKNEPN
SLDGKGYTAYESERFHDNMDAFVKIIKTEVMNKGSVIAYIKAENVMGYEFSGKKVQNL
CGDDTADHAVNIVGYGNYVNSEGEKKSYWIVRNSWGPYWGDEGYFKVDMYGPTHCHFN
FIHSVVIFNVDLPMNNKTTKKESKIYDYYLKASPEFYHNLYFKNFNVGKKNLFSEKED
NENNKKLGNNYIIFGQDTAGSGQSGKESNTALESAGTSNEVSERVHVYHILKHIKDGK
IRMGMRKYIDTQDVNKKHSCTRSYAFNPENYEKCVNLCNVNWKTCEEKTSPGLCLSKL
DTNNECYFCYV
>SERA_PLAFD
MKSYISLFFILCVIFNKNVIKCTGESQTGNTGGGQAGNTVGDQAGSTGGSPQGSTGAS
QPGSSEPSNPVSSGHSVSTVSVSQTSTSSEKQDTIQVKSALLKDYMGLKVTGPCNENF
IMFLVPHIYIDVDTEDTNIELRTTLKETNNAISFESNSGSLEKKKYVKLPSNGTTGEQ
GSSTGTVRGDTEPISDSSSSSSSSSSSSSSSSSSSSSSSSSSSSSSSSSSSSESLPANG
PDSPTVKPPRNLQNICETGKNFKLVVYIKENTLIIKWKVYGETKDTTENNKVDVRKYL
INEKETPFTSILIHAYKEHNGTNLIESKNYALGSDIPEKCDTLASNCFLSGNFNIEKC
FQCALLVEKENKNDVCYKYLSEDIVSNFKEIKAETEDDDEDDYTEYKLTESIDNILVK
MFKTNENNDKSELIKLEEVDDSLKLELMNYCSLLKDVDTTGTLDNYGMGNEMDIFNNL
KRLLIYHSEENINTLKNKFRNAAVCLKNVDDWIVNKRGLVLPELNYDLEYFNEHLYND

KNSPEDKDNKGKGVVHVDTTLEKEDTLSYDNSDNMFCNKEYCNRLKDENNCISNLQVE
DQGNCDTSWIFASKYHLETIRCMKGYEPTKISALYVANCYKGEHKDRCDEGSSPMEFL
QIIEDYGFLPAESNYPYNYVKVGEQCPKVEDHWMNLWDNGKILHNKNEPNSLDGKGYT
AYESERFHDNMDAFVKIIKTEVMNKGSVIAYIKAENVMGYEFSGKKVQNLCGDDTADH
AVNIVGYGNYVNSEGEKKSYWIVRNSWGPYWGDEGYFKVDMYGPTHCHFNFIHSVVIF
NVDLPMNNKTTKKESKIYDYYLKASPEFYHNLYFKNFNVGKKNLFSEKEDNENNKKLG
NNYIIFGQDTAGSGQSGKESNTALESAGTSNEVSERVHVYHILKHIKDGKIRMGMRKY
IDTQDVNKKHSCTRSYAFNPENYEKCVNLCNVNWKTCEEKTSPGLCLSKLDTNNECYF
CYV
>SERA_PLAFG
MKSYISLFFILCVIFNKNVIKCTGESQTGNTGGGQAGNTVGDQAGSTGGSPQGSTGAS
QPGSSEPSNPVSSGHSVSTVSVSQTSTSSEKQDTIQVKSALLKDYMGLKVTGPCNENF
IMFLVPHIYIDVDTEDTNIELRTTLKETNNAISFESNSGSLEKKKYVKLPSNGTTGEQ
GSSTGTVRGDTEPISDSSSSSSSSSSSSSSSSSSSSSSSSSSSSSSSSSSSESLPANG
PDSPTVKPPRNLQNICETGKNFKLVVYIKENTLIIKWKVYGETKDTTENNKVDVRKYL
INEKETPFTSILIHAYKEHNGTNLIESKNYALGSDIPEKCDTLASNCFLSGNFNIEKC
FQCALLVEKENKNDVCYKYLSEDIVSNFKEIKAETEDDDEDDYTEYKLTESIDNILVK
MFKTNENNDKSELIKLEEVDDSLKLELMNYCSLLKDVDTTGTLDNYGMGNEMDIFNNL
KRLLIYHSEENINTLKNKFRNAAVCLKNVDDWIVNKRGLVLPELNYDLEYFNEHLYND
KNSPEDKDNKGKGVVHVDTTLEKEDTLSYDNSDNMFCNKEYCNRLKDENNCISNLQVE
DQGNCDTSWIFASKYHLETIRCMKGYEPTKISALYVANCYKGEHKDRCDEGSSPMEFL
QIIEDYGFLPAESNYPYNYVKVGEQCPKVEDHWMNLWDNGKILHNKNEPNSLDGKGYT
AYESERFHDNMDAFVKIIKTEVMNKGSVIAYIKAENVMGYEFSGKKVQNLCGDDTADH
AVNIVGYGNYVNSEGEKKSYWIVRNSWGPYWGDEGYFKVDMYGPTHCHFNFIHSVVIF
NVDLPMNNKTTKKESKIYDYYLKASPEFYHNLYFKNFNVGKKNLFSEKEDNENNKKLG
NNYIIFGQDTAGSGQSGKESNTALESAGTSNEVSERVHVYHILKHIKDGKIRMGMRKY
IDTQDVNKKHSCTRSYAFNPENYEKCVNLCNVNWKTCEEKTSPGLCLSKLDTNNECYF
CYV
>SSSP_PLAFO
MNIRKFIPSLALMLIFFAFANLVLSDANDKAKKPAGKGSPSTLQTPGSSSGASLHAVG
PNQGGLSQGLSGKDSADKMPLETQLAIEEIKSLSNMLDKKTTVNRNLIISTAVTNMIM
LIILSGIVGFKVKKTKNADDDKGDKDKDKDNTDEGDEGDDS
>TBA_PLAFK
MREVISIHVGQAGIQVGNACWELFCLEHGIQPDGQMPSDKASRANDDAFNTFFSETGA
GKHVPRCVFVDLEPTVVDEVRTGTYRQLFHPEQLISGKEDAANNFARGHYTIGKEVID
VCLDRIRKLADNCTGLQGFLMFSAVGGGTGSGFGCLMLERLSVDYGKKSKLNFCCWPS
PQVSTAVVEPYNSVLSTHSLLEHTDVAIMLDNEAIYDICRRNLDIERPTYTNLNRLIA
QVISSLTASLRFDGALNVDVTEFQTNLVPYPRIHFMLSSYAPVVSAEKAYHEQLSVSE
ITNSAFEPANMMAKCDPRHGKYMACCLMYRGDVVPKDVNAAVATIKTKRTIQFVDWCP
TGFKCGINYQPPTVVPGGDLAKVMRAVCMISNSTAIAEVFSRMDQKFDLMYAKRAFVH
WYVGEGMEEGEFSEAREDLAALEKDYEEVGIESNEAEGEDEGYEADY
>TBB_PLAF7
MREIVHIQAGQCGNQIGAKFWEVISDEHGIDPSGTYCGDSDLQLERVDVFYNEATGGR
YVPRAILMDLEPGTMDSVRAGPFGQLFRPDNFVFGQTGAGNNWAKGHYTEGAELIDAV
LDVVRKEAEGCDCLQGFQITHSLGGGTGSGMGTLLISKIREEYPDRIMETFSVFPSPK
VSDTVVEPYNATLSVHQLVENADEVQVIDNEALYDICFRTLKLTTPTYGDLNHLVSAA
MSGVTCSLRFPGQLNSDLRKLAVNLIPFPRLHFFMIGFAPLTSRGSQQYRALTVPELT
QQMFDAKNMMCASDPRHGRYLTACAMFRGRMSTKEVDEQMLNVQNKNSSYFVEWIPHN
TKSSVCDIPPKGLKMAVTFVGNSTAIQEMFKRVSDQFTAMFRRKAFLHWYTGEGMDEM
EFTEAESNMNDLVSEYQQYQDATAEEEGEFEEEEGDVEA
>TBB_PLAFA

MREIVHIQAGQCGNQIGAKFWEVISDEHGIDPSGTYSGDSDLQLERVDVFYNEATGGR
YVPRAILMDLEPGTMDSVRAGPFGQLFRPDNFVFGQTGAGNNWAKGHYTEGAELIDAV
LDVLRKEAEGCDCLQGFQITHSLGGGTGSGMGTLLISKIREEYPDRIMETFSVFPSPK
VSDTVVEPYNATLSVHQLVENADEVQVIDNEALYDICFRTLKLTTPTYGDLNHLVSAA
MSGVTCSLRFPGQLNSDLRKLAVNLIPFPRLHFFMYGFAPLTSRGSQQYRALTVPELT
QQMFDAKNMMCTSDPRHGRYLTACAMFRGRMSTKEVDEQMLNVQNKNSSYFVEWIPHN
TKSSVCDIPPLGLKMAVTFVGNSTAIQEMFKRVSDQFTAMFRRKAFLHWYTGEGMDEM
EFTEAESNMNDLVSEYQQYQDATAEEEGEFEEEEGDVEA
>TBB_PLAFK
MREIVHIQAGQCGNQIGAKFWEVISDEHGIDPSGTYCGDSDLQLERVDVFYNEATGGR
YVPRAILMDLEPGTMDSVRAGPFGQLFRPDNFVFGQTGAGNNWAKGHYTEGAELIDAV
LDVVRKEAEGCDCLQGFQITHSLGGGTGSGMGTLLISKIREEYPDRIMETFSVFPSPK
VSDTVVEPYNATLSVHQLVENADEVQVIDNEALYDICFRTLKLTTPTYGDLNHLVSAA
MSGVTCSLRFPGQLNSDLRKLAVNLIPFPRLHFFMIGFAPLTSRGSQQYRALTVPELT
QQMFDAKNMMCASDPRHGRYLTACAMFRGRMSTKEVDEQMLNVQNKNSSYFVEWIPHN
TKSSVCDIPPKGLKMAVTFVGNSTAIQEMFKRVSDQFTAMFRRKAFLHWYTGEGMDEM
EFTEAESNMNDLVSEYQQYQDATAEEEGEFEEEEGDVEA
>TBG_PLAFO
MPREIITLQCGQCGNQIGVEFWKQLCNEHNIDQEGILKNNNFLNEDRKDIFFYQADDE
HFIPGALLFDLEPRVINSIQTSEYRNLYNPENMFISKEGGGAGNNWGCGYSQGHKVEE
EIIDMIDREVDNSDNLEGFILSHSIAGGTGSGMGSYLLELLNDNYSKKMIQTFSVFPL
LTNESSDVVVQPYNSILTLKRLILSTDSVVVIDNTSLNRIFVERLKLNNPTFQQTNTI
ISNVMSASTTTLRYPGSMNNDMISLISSLIINPKCHFLITSYTPITIDKHISNVQKTT
VLDVMKRLLHTKNIMVSAPVRRGMYISILNIIRGETDPTQVHKGLQRIRDRKLVNFIK
WNPASIQVTLAKQSPHVVSQHKVCGLMMANHTSISTLFERCVTQFDRLYKRRAFLENY
KKESMFSSADGQGNFEEMESSKEITQNLIDEYKSAERDDYFTNTYI
>TBP_PLAFA
MNFLEQDQLFLENINQDNVVSAHYTSEYDNNEKEKSDDLKNKLVHKNISLNIHNIISS
ANLCIDINLRLVAVSIRNAEYNPSKINTLIIRLNKPQCTALIFKNGRIMLTGTRTKKD
SIMGCKKIAKIIKIVTKDKVKFCNFKIENIIASANCNIPIRLEVLAHDHKEYCNYEPE
LFAGLVYRYKPTSNLKSVILIFVSGKIIITGCKSVNKLYTVFQDIYNVLIQYKN
>TCPH_PLAF7
MSHLMSLPIVLLKEGTDTAQGRSQIIRNINACQIIVDIVKTTLGPRGMDKLIYTERDV
TITNDGATVMNLLNISHPAASILVDIAKSQDDEVGDGTTSVVVVAGELLNEAKGLLND
GIEPNMIIDGFRNACNVAINKLNELSLNFSNKNEEEKRSILLKCAQTALNSKLVSNHK
EFFGELVVNAVYKLGDNLDKSNIGIKKVTGGSCLDTQLIYGVAFKKTFSYAGFEQQPK
KFINPKILLLNVELELKAEKENAEVRIENPNEYNSIVQAEWDIIFKKLNLIKDCGANI
VLSKLPIGDIATQFFADHDIFCAGRVEDADLKRTANATGALVQTSLFNLNDDVLGTCG
VFEEVQIGNERYNIFKECLKTKSVTIILRGGAKQFIEEVERSINDAIMIVLRCITNSE
IVPGAGSIEMQLSKYLRIYSRSICNKEQIVLFSFAKALESIPRHLSHNAGYDSTDILN
KLRKKHSEQTSDIWYGVDCMEGDIINAYDNCIFEVTKIKRNVIYSATEAACLILSIDE
TIKNPSSAAGTQRSPYS
>TCTP_PLAF7
MKVFKDVFTNDEVCSDSYVQQDPFEVPEFREIAFEVKSNKRIKGNEDYGIADNSEDAV
EGMGADVEHVIDIVDSFQLTSTAFSKKEYSAYIKNYMQKVAKYLEEKKPDRVEIFKTK
AQPFIKHILTNFDDFEFYMGESLDMEAGIIYSYYKGEEITPRFVYISDGLFEEKY
>THIO_PLAF7
MVKIVTSQAEFDSIISQNELVIVDFFAEWCGPCKRIAPFYEECSKTYTKMVFIKVDVD
EVSEVTEKENITSMPTFKVYKNGSSVDTLLGANDSALKQLIEKYAA
>TIP_PLAF7
MYNFLSCKKKSIILQVLLIICTYNILLNFVNIFVNNNEKNHKNKYENRIKSFYVEAYN

WNFLEKWKSINTNEKLEYKINYNIGLNIDAEIGDFGDYNSDVKTDLILFKYDKDKLLS
TIFIYVFSVKENKFIYHTEVSFEGKIMNVTAIDLNFDGALDVLVLFKDNKDSSKSNKY
YVAAFLQNDNDQLEEIWNSKKKEQNDESITDNEEDNIYYTNIHPLICDINNDGLPDII
GQQSGGPDGFFRFIWINTRNGFKSFLWKNINIFKYSELDEITNPNSSAIVDINGDCKS
DLVFTVYNSYEKRIGLEIWLNKIIDGKSFYVKYSQDYLLPPNSLQVLFGDFNGDGSID
LVVPTCVKSSFCNYCCVSDDKIYFIPNIQKKICDSSWKKPDETKCRPASNLCSESDFE
FQQNLTDDFISVVDTSGLHLSGNADYPYYLSVGDIDDDGYLDLLITLKNDKGQKYVRI
YKNELKIHYEENSLEVRGFYNFYQFVTSPEESVTDVYNAAFFDIFENGVLDILIFGKY
ITSNKKTKYAAVGFIRNNETDSLFLKSTALNGICVNDCYKEKDKITTKTLGGNAHGPT
FKITVIDVNGVKSSRIGVQKSQSAHFPLQLPYVLFGLGRTSNYVEEFYVGMPTHEQKY
YNMWVSIIPNSHIIVIPYPLNNSNKWQIQLSVNPSKKFYSILYITLICLSVIGVLIFI
LDRKEKVEDSKEELGFKSHFVIG
>TOP2_PLAFK
MAKNKTIEERYQKKSQIEHILLRPDTYIGSVEMHTQLLWVWNKEKNRMVQKNITYVPG
LYKIFDEIIVNAADVKAREKEKSENPMTCIKIEINKENKRISVYNDGEGIPVDIHKEM
NIYVPHMIFGELLTSDNYDDAEDRITGGRNGFGAKLTNIFSKEFIVQCGDSSRKKEFK
MTWSDNMSKFSEPHIKNYNGKDYVKVTFKPDLNKFGMTEMDDDIESLLFKRVYDLAGT
CSVRVYLNGQRLAVKDFKSYVDLYLKDNSNDNKNNKGQNDNNNNNNNNNDENANQNND
NLDVSLSNEPADGTPTKNNNNNNNNNDEDEIVKIHEKQHRWEIVVSKSDGSQFQQVSF
VNSICTTKGGSHVNYIVEQLLSSLSKKANAKNKGGMEIKSGHIRNHLWVFVNCLIVNP
TFDSQTKETLTTKPVKFGSKCILSDKTINNVLKSPILSNILLWAQAKAQVELKKKMKA
GSSKARERIIGIPKLEDANDAGSKYSQECTLILTEGDSAKTSCLAGLSIVGRDKYGVF
PLKGKLLNVRDASFKQLMDNKEIQNIFRIMGLDITDKNKDDIKGLRYGSLMIMTDQDY
DGSHIKGLLINMIHKFWPSLLKHKGFLSEFVTPIVKVQKGSQEYSFFTIAEYEQWKEN
TNLLGWKIKYYKGLGTSTDREFKQYFSDIKNHKIMFLWTGDRDGDSIDMAFSKKRIED
RKLWLQNFILGSYVDHKEKDLSYYDFVNKELIYYSRYDTERSIPNIMDGWKPGQRKVL
YGCFKRNLRNECKVAQLVGYIAEHSAYHHHGESSLQQTIINMAQTFVGSNNINFLEPC
GQFGSRKEGGKDASAARYIFTKLASSTRSIFNEYDDPILKYLNEEGQKIEPQYYIPVI
PTILVNGCEGIGTGYSSFIPNYNYKDIIDNIKRYINKEPLIPMVPWYKDFKGRIESNG
KTGYETIGIINKIDNDTLEITELPIKKWTQDYKEFLEELLTDEKHQLILDYIDNSSHE
DICFTIKMDPAKLQKAEEEGLEKVFKLKSTLTTTNMTLFDPNLKLQRYSTELDILKEF
CYQRLKAYENRKSYLISKLEKEKRIISNKTKFILAIVNNELIVNKKKKKVLVEELYRK
GYDPYKDINKIKKEEIFEQELLDAADNPEDNEEIIAGITVKDYDYLLSMPIFSLTLEK
VEDLLTQLKEKERELEILRNITVETMWLKDIEKVEEAIEFQRNVELSNREESNKFKVA
RKQGPSSMKKKKKKKKLSSDEESEGGDTSDSSEFLVNTLNIKKNTNKKTTTSSNNVNN
SKKRLRKADDLNSNELDNTLSVSKTFDDNNNLTDNTPLINRLNDENNEFSSNNVDNKS
TNKNSRKKKPKIADSTNDNNSELNSSIQINDNVNDDINITISPNKTINVNEFSSIKNK
LLELGI
>TPIS_PLAF7
MARKYFVAANWKCNGTLESIKSLTNSFNNLDFDPSKLDVVVFPVSVHYDHTRKLLQSK
FSTGIQNVSKFGNGSYTGEVSAEIAKDLNIEYVIIGHFERRKYFHETDEDVREKLQAS
LKNNLKAVVCFGESLEQREQNKTIEVITKQVKAFVDLIDNFDNVILAYEPLWAIGTGK
TATPEQAQLVHKEIRKIVKDTCGEKQANQIRILYGGSVNTENCSSLIQQEDIDGFLVG
NASLKESFVDIIKSAM
>TPIS_PLAFA
MARKYFVAANWKCNGTLESIKSLTNSFNNLDFDPSKLDVVVFPVSVHYDHTRKLLQSK
FSTGIQNVSKFGNGSYTGEVSAEIAKDLNIEYVIIGHFERRKYFHETDEDVREKLQAS
LKNNLKAVVCFGESLEQREQNKTIEVITKQVKAFVDLIDNFDNVILAYEPLWAIGTGK
TATPEQAQLVHKEIRKIVKDTCGEKQANQIRILYGGSVNTENCSSLIQQEDIDGFLVG
NASLKESFVDIIKSAM
>TPPC5_PLAF7

MDKSKSSIEKELNRIKQDVSLSAFSILFSEMVQYCLYKSKRGYRIEDCLHEMGLRVGY
KLNEYLTYKNKVKRSINIINILTFISKHVWKYLFQHSSDLLKSQDSIYEYMICDKNIL
LNKFINVPKDYGNINCAAFAAGIVEGFLCSSEFQADVTAHTIHEGDDNYNTTIFIKFY
PEVVEREKNH
>TPPC5_PLAFA
MDKSKSSIEKELNRIKQDVSLSAFSILFSEMVQYCLYKSKRGYRIEDCLHEMGLRVGY
KLNEYLTYKNKVKRSINIINILTFISKHVWKYLFQHSSDLLKSQDSIYEYMICDKNIL
LNKFINVPKDYGNINCAAFAAGIVEGFLCSSEFQADVTAHTIHEGDDNYNTTIFIKFY
PEVVEREKNH
>TRAP_PLAFA
MNHLGNVKYLVIVFLIFFDLFLVNGRDVQNNIVDEIKYSEEVCNDQVDLYLLMDCSGS
IRRHNWVNHAVPLAMKLIQQLNLNDNAIHLYVNVFSNNAKEIIRLHSDASKNKEKALI
IIRSLLSTNLPYGRTNLTDALLQVRKHLNDRINRENANQLVVILTDGIPDSIQDSLKE
SRKLSDRGVKIAVFGIGQGINVAFNRFLVGCHPSDGKCNLYADSAWENVKNVIGPFMK
AVCVEVEKTASCGVWDEWSPCSVTCGKGTRSRKREILHEGCTSEIQEQCEEERCPPKW
EPLDVPDEPEDDQPRPRGDNSSVQKPEENIIDNNPQEPSPNPEEGKDENPNGFDLDEN
PENPPNPDIPEQKPNIPEDSEKEVPSDVPKNPEDDREENFDIPKKPENKHDNQNNLPN
DKSDRNIPYSPLPPKVLDNERKQSDPQSQDNNGNRHVPNSEDRETRPHGRNNENRSYN
RKYNDTPKHPEREEHEKPDNNKKKGESDNKYKIAGGIAGGLALLACAGLAYKFVVPGA
ATPYAGEPAPFDETLGEEDKDLDEPEQFRLPEENEWN
>TRXR_PLAF5
MCKDKNEKKNYEHVNANEKNGYLASEKNELTKNKVEEHTYDYDYVVIGGGPGGMASAK
EAAAHGARVLLFDYVKPSSQGTKWGIGGTCVNVGCVPKKLMHYAGHMGSIFKLDSKAY
GWKFDNLKHDWKKLVTTVQSHIRSLNFSYMTGLRSSKVKYINGLAKLKDKNTVSYYLK
GDLSKEETVTGKYILIATGCRPHIPDDVEGAKELSITSDDIFSLKKDPGKTLVVGASY
VALECSGFLNSLGYDVTVAVRSIVLRGFDQQCAVKVKLYMEEQGVMFKNGILPKKLTK
MDDKILVEFSDKTSELYDTVLYAIGRKGDIDGLNLESLNMNVNKSNNKIIADHLSCTN
IPSIFAVGDVAENVPELAPVAIKAGEILARRLFKDSDEIMDYSYIPTSIYTPIEYGAC
GYSEEKAYELYGKSNVEVFLQEFNNLEISAVHRQKHIRAQKDEYDLDVSSTCLAKLVC
LKNEDNRVIGFHYVGPNAGEVTQGMALALRLKVKKKDFDNCIGIHPTDAESFMNLFVT
ISSGLSYAAKGGCGGGKCG
>TRXR_PLAF7
MCKDKNEKKNYEHVNANEKNGYLASEKNELTKNKVEEHTYDYDYVVIGGGPGGMASAK
EAAAHGARVLLFDYVKPSSQGTKWGIGGTCVNVGCVPKKLMHYAGHMGSIFKLDSKAY
GWKFDNLKHDWKKLVTTVQSHIRSLNFSYMTGLRSSKVKYINGLAKLKDKNTVSYYLK
GDLSKEETVTGKYILIATGCRPHIPDDVEGAKELSITSDDIFSLKKDPGKTLVVGASY
VALECSGFLNSLGYDVTVAVRSIVLRGFDQQCAVKVKLYMEEQGVMFKNGILPKKLTK
MDDKILVEFSDKTSELYDTVLYAIGRKGDIDGLNLESLNMNVNKSNNKIIADHLSCTN
IPSIFAVGDVAENVPELAPVAIKAGEILARRLFKDSDEIMDYSYIPTSIYTPIEYGAC
GYSEEKAYELYGKSNVEVFLQEFNNLEISAVHRQKHIRAQKDEYDLDVSSTCLAKLVC
LKNEDNRVIGFHYVGPNAGEVTQGMALALRLKVKKKDFDNCIGIHPTDAESFMNLFVT
ISSGLSYAAKGGCGGGKCG
>UTP11_PLAF7
MSNFKNIIPKRTYLERGQAKHRLHLGELEKKVDYGKRREIYKKKKKIENVLKEKIMTK
NPDEFHTGMVHSRVTEDNVLVREEKVLKKEVQLKNKRQELKEQTNDLYNKLKKINKRL
SNYQMNIPLRYVFNNSHELYNENEIYTLKAENKKLKKRGDLIQKKYNGLINMKKNLLD
QIRKLDNKYITTYHKVDGYNIVTDKGKTPYRLYQPRLK
>VATA_PLAF7
MTKVAVEKEEPGVVYKVAGSLVIAENMSGTRMYELAKVGWNKLVGEIIRLEGNYAYIQ
VYEDTSGLSVGDPVIKTGNALSVELGPGILDNIYDGIQRPLERIANVCGDVYIYKGID
MTSLDHDKQWQFYADKKLKLNDIVTGGDIFGFVDENKLFKEHKIMAPPNAKGRLTYIA

PDGSYTLKDKIFELEYQGKKYTYGLSHLWPVRDPRPVLEKVTGDTLLLTGQRVLDSLF
PTVQGGTCAIPGAFGCGKTCVSQALSKYSNSEVIIYVGCGERGNEMAEILSDFPELTT
KVDNEDVGIMQRTCLVANTSNMPVAAREASIYTGITLCEYFRDMGYNATMMADSTSRW
AEALREISGRLAEMPADSGYPAYLGARLASFYERAGKVKCIGSPSRIGSITIVGAVSP
PGGDFSDPVTTATMSIVQAFWGLDKKLAQRKHFPSVNWSTSFSKYVRQLEQYFDNFDQ
DFLSLRQKISDILQQESDLNDIVQLVGKDSLSEDQKVVMEVAKIIREDFLQQNAFSDY
DYMCPLQKTVGMMRIICHFYAQCLRTLQEYDSRERKIGWGSIYNTLRPTINKITHMKF
ENPKNSDEYFKKYFKALEEEITVGLRNLMEK
>VATA_PLAFA
MTKVAVEKEEPGVVYKVAGSLVIAENMSGTRMYELAKVGWNKLVGEIIRLEGNYAYIQ
VYEDTSGLSVGDPVIKTGNALSVELGPGILDNIYDGIQRPLERIANVCGDVYIYKGID
MTSLDHDKQWQFYADKKLKLNDIVTGGDIFGFVDENKLFKEHKIMAPPNAKGRLTYIA
PDGSYTLKDKIFELEYQGKKYTYGLSHLWPVRDPRPVLEKVTGDTLLLTGQRVLDSLF
PTVQGGTCAIPGAFGCGKTCVSQALSKYSNSEVIIYVGCGERGNEMAEILSDFPELTT
KVDNEDVGIMQRTCLVANTSNMPVAAREASIYTGITLCEYFRDMGYNATMMADSTSRW
AEALREISGRLAEMPADSGYPAYLGARLASFYERAGKVKCIGSPSRIGSITIVGAVSP
PGGDFSDPVTTATMSIVQAFWGLDKKLAQRKHFPSVNWSTSFSKYVRQLEQYFDNFDQ
DFLSLRQKISDILQQESDLNDIVQLVGKDSLSEDQKVVMEVAKIIREDFLQQNAFSDY
DYMCPLQKTVGMMRIICHFYAQCLRTLQEYDSRERKIGWGSIYNTLRPTINKITHMKF
ENPKNSDEYFKKYFKALEEEITVGLRNLMEK
>VATB_PLAFA
MSKEVVNTKAEASRVNALAAVRNYKVCPRLEYKTISGVQGPLVIIEDVKFPKYSEIVT
IHLSDNTTRQGQILEVCGKKAVIQVFEGTSGIDNKNSYVEVSGDILKMPMSDEMLGRV
FNGSGKPIDKGPNILADDYLDINGNPINPQCRVYPKEMIQTGISTIDVMNSIVRGQKI
PLFSAAGLPHNEIGAQICRQASLVQGKDVLDHSDDNFAVVFGAMGVNMETARYFRQDF
EENGKMERVCLFLNLANDPTIERILTPRIALTTAEYLAFEKEMHVFVILTDMSSYADA
LREVSSAREEVPGRRGYPGYMYSDLSTIYERAGRVEGRNGSITQFPILTMPNDDITHP
IPDLTGYITEGQIFVDRNLYNRQIYPPINVLPSLSRLMKSGIGHNMTRIDHPYVSDQL
YSNYAIAQDVKAMKAVIGEEALSNDDILYLEFLDKFEKRFITQNTYECRDIYQSLDIA
WELLRIFPEDMLKKIKTDILSKYYPRHHAN
>VPS26_PLAF7
MLSTIFGSVCSIDLKIDADDNKKFAFLRKDKKGEKCPIFSDGEDINGTATISLKPGKK
FEHYGIKLELIGQINILNDKANSYDFFSISKDLEPPGFLVESKQFKWKFSAVDKQHES
YFGTNVQLRYFVRLNIIKGYSGNIQKEIDFIVQNLCIPPEINNTIKMEVGIEDCLHIE
FEYDKSKYHLKDVVVGKVYFLLVRIKIKHMELDIIKMETSGVGKNYTTETVTLSKFEI
MDGSPIKSECIPVRLYLSGFDLTPTYKNIQNKFSVKYYINLIIVDEEERRYFKKQEIF
LWRKKMG
>YCA1_PLAFA
MVNKEEAQRLKELGNKCFQEGKYEEAVKYFSDAITNDPLDHVLYSNLSGAFASLGRFY
EALESANKCISIKKDWPKGYIRKGCAEHGLRQLSNAEKTYLEGLKIDPNNKSLQDALS
KVRNENMLENAQLIAHLNNIIENDPQLKSYKEENSNYPHELLNTIKSINSNPMNIRII
LSTCHPKISEGVEKFFGFKFTGEGNDAEERQRQQREEEERRKKKEEEERKKKEEEEMK
KQNRTPEQIQGDEHKLKVMN
>YCA2_PLAFA
EFLEKVQIIKNTNLSNSLCILSIPCTVDIDTVITETINKYDSIIDGILLSGLGYDESN
ETRTNAFKNILNILPNNKLKFIQLSNGNPIEILHAIYHGIDVIEPNFPYYLAKNGKAI
NMNLKMDNLQDGNEYNLQDKNNDNIYDINLLDFKNDVNFIIDLNNPKYVLDHSTITCN
SPRKESKSYIHHLLKCHELTAHVILTYHNLYIYRSFFQEIQLHIKENNFLSYINWFIE
KNELNKKEE
>YDH1_PLAFS
DPYKERIKSDIRQINESQYLKSLAYKYISGEDYTQYLLLNEVLKDDQDYCTCTRRTIY

EESMDNTVEFAKKMYELSA
>YDH2_PLAFS
MVLVTCNRALAQGDFCLLALIFCHQTCRTPEKHKASQSSAKLVSINISLITSHHRLRH
PRRRQHHHRNNFAPTNWYWG
>YDH3_PLAFS
PQYQFVGAKLFRWWCWRRRGWRRRWWLVIKLMLIETSFALDCEALCFSGVRQV
